# Iron‐Catalyzed Tunable Double Bond Migration and Geometrical Isomerization in Olefins via a Spin‐Accelerated Alkyl Mechanism

**DOI:** 10.1002/anie.202519729

**Published:** 2025-10-16

**Authors:** Abdul‐Halim Obeid, Christian Herrero, Régis Guillot, Jérôme Hannedouche

**Affiliations:** ^1^ Université Paris‐Saclay CNRS Institut de Chimie Moléculaire et des Matériaux d'Orsay (ICMMO), Bâtiment Henri Moissan 17 avenue des Sciences Orsay 91400 France

**Keywords:** Alkenes, DFT calculations, Iron, Isomerization, Mechanistic studies

## Abstract

Despite the central relevance of metal‐catalyzed alkene isomerization in academia and industry and the overarching role of iron in sustainable catalysis, highly efficient iron‐catalyzed positional and geometrical isomerizations of alkenes remain long‐sought‐after goals. Herein, we disclose the development of an easily accessible, well‐defined anilido‐aldimine iron(II) alkyl catalyst that efficiently and selectively performs, for the first time, tunable olefin migration over one and multiple carbon–carbon bonds and configurational alkene isomerization. This singular catalyst behavior also unraveled the implementation of productive regiodivergent and regio‐ and stereoconvergent olefin isomerization processes. Notably, with only 1 equiv. of Cy_2_NH·BH_3_ (relative to Fe) under mild conditions, these reactions exhibited a broad substrate scope, including (hetero)aryl‐substituted and aliphatic alkenes of different substitution patterns and with diverse functionalities. They also displayed high regio‐ and stereoselectivities. Using experimental and computational approaches, a detailed mechanistic study of the positional isomerization regime suggested a redox‐neutral alkyl‐type mechanism via an isolable iron(II)‐hydride species. This study indicated the stereo‐ and rate‐determining β‐hydride elimination step occurs through a two‐state reactivity resulting in spin acceleration and a stereoselectivity correlated to these spin state changes. This work also elucidated the peculiar and critical role of the anilido‐aldimine skeleton on the reactivity.

## Graphical abstract

A readily accessible, well‐defined iron(II) catalyst that demonstrates high efficiency and versatility in positional and geometrical alkene isomerization is presented. A comprehensive mechanistic study suggests an alkyl mechanism via an isolable iron hydride species and indicates the stereo‐ and rate‐determining β‐hydride elimination step exhibits a two‐state reactivity resulting in spin acceleration and dictating the stereoselectivity outcome.

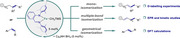

## Introduction

**Scheme 1:**
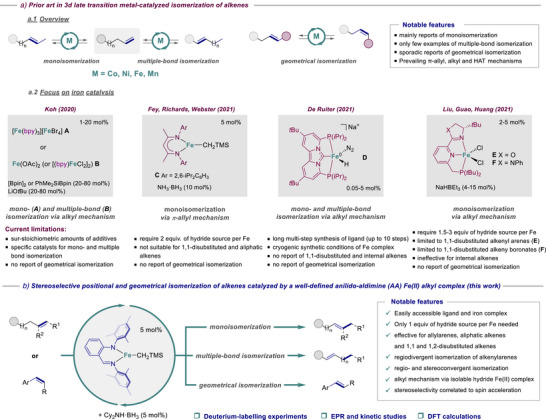
Prior art in 3d late transition metal‐catalyzed isomerization of alkenes with a focus on iron catalysis (A) and this work (B). bpy = 2,2′‐bipyridine.

Alkenes are of great importance in organic chemistry due to their prevalence in natural and biologically active molecules and their wide spectrum of applications and synthetic uses. Over the years, metal‐catalyzed carbon–carbon double (C═C) bond isomerization has emerged as an appealing atom‐economical approach to provide valuable internal alkenes from readily accessible terminal analogues.^[^ ^1^, ^2^, ^3^, ^4^, ^5^, ^6^, ^7^, ^8^, ^9^ ^]^ This approach poses two major challenges: i) control of the regioselectivity of the C═C bond displacement over the carbon scaffold and/or ii) control of the stereoselectivity, which consists in setting the configurational geometry of the reformed alkene. For decades, alkene migration relied on noble transition metal‐based catalysts to tackle these selectivity issues with notable achievements.^[^ ^10^, ^11^, ^12^, ^13^, ^14^, ^15^, ^16^, ^17^, ^18^, ^19^, ^20^, ^21^ ^]^ For economic and environmental concerns, earth‐abundant 3d transition metals have been investigated as sustainable substitutes. In addition, thanks to a rich landscape of variable redox and spin states, these metals could potentially exhibit discrete and complementary reactivities, opening the door for further synthetic applications.^[^ ^22^ ^]^ In this vein, Co,^[^ ^4^, ^7^, ^8^, ^23^, ^24^, ^25^, ^26^, ^27^, ^28^, ^29^, ^30^, ^31^, ^32^, ^33^, ^34^, ^35^, ^36^, ^37^ ^]^ Ni,^[^ ^4^, ^38^, ^39^, ^40^, ^41^, ^42^, ^43^, ^44^, ^45^, ^46^ ^]^ Fe,^[^ ^4^, ^9^, ^47^, ^48^, ^49^, ^50^, ^51^, ^52^, ^53^, ^54^, ^55^, ^56^, ^57^ ^]^ and more recently Mn^[^ ^58^ ^]^ have been explored to promote selective alkene isomerization. While remarkable advances were achieved with Co and Ni, most of the reports were dedicated to the positional isomerization over one position of terminal alkenes with only sporadic examples of stereocontrolled double bond transposition‐only processes over multiple positions^[^ ^37^, ^39^, ^41^, ^44^ ^]^ (Scheme 1, a.1). Moreover, only scarce reports dealt with geometrical isomerization that allows to set the C═C bond configuration without a bond shift.^[^ ^26^, ^27^, ^41^ ^]^ Despite its greater potential for sustainability, it is only in the last few years that iron catalysis has arisen in the field thanks to the advent of a few well‐defined bespoke iron complexes (Scheme 1, a.2).^[^ ^53^, ^54^, ^55^, ^56^, ^57^ ^]^ Although notable developments were made with these complexes, they are far from ideal for broad synthetic applications, and they have some shortcomings that need to be addressed. Indeed, they required either the use of sur‐stoichiometric amounts of additives^[^ ^53^ ^]^ (**A**, **B**) or a hydride source^[^ ^54^, ^56^, ^57^ ^]^ (**C**, **E**, **F**) (relative to Fe) or a very sophisticated bespoke ligand‐Fe‐complex synthesis^[^ ^55^ ^]^ (**D**). They also suffered from limitations or restrictions in terms of alkene substitution patterns. Complex **C** was not suitable for 1,1‐disubstituted and aliphatic alkenes, while complexes **E** and **F** were limited to either 1,1‐disubstituted alkenyl arenes or 1,1‐disubstituted alkenyl boronates and ineffective for internal alkenes.^[^ ^54^, ^56^, ^57^ ^]^ Complex **D** was only unveiled for the isomerization of monosubstituted alkenes. Moreover, only one report displayed concomitantly mono‐ and multiple‐bond isomerization using the same, but difficult to access, catalyst (**D**)^[^ ^55^ ^]^ and none of them has disclosed the geometrical alkene isomerization. Therefore, it is highly desirable to conceive an efficient catalytic system that overcomes these shortfalls in olefin transposition and that could also be applied to geometrical alkene isomerization.

Herein, we report the synthesis of a readily accessible, well‐defined low‐coordinate anilido‐aldimine (AA) Fe(II) alkyl catalyst that effectively accomplished, for the first time, controlled positional isomerization over one and multiple‐bonds and geometrical isomerization of alkenes stereoselectively (Scheme 1, B.). These reactions operated under mild conditions in the presence of only 1 equiv. of hydride source (relative to Fe) and accommodated diverse functional groups. They also tolerated alkenes of different substitution patterns, such (hetero)aryl‐substituted and aliphatic terminal alkenes and 1,1‐ and 1,2‐disubstituted aliphatic alkenes. This catalyst singularity offered the opportunity to perform selectively i) regiodivergent isomerization on alkenes of extended carbon skeletons into a single regioisomer and ii) regio‐ and stereoconvergent alkene migration to convert isomeric alkene mixtures into a single isomer. We performed in‐depth mechanistic investigations through deuterium‐labeling, ^1^H NMR, EPR, kinetic, and stoichiometric experiments, and DFT analysis. The stoichiometric experiments included the isolation and characterization of the catalytically competent iron(II) hydride species. Overall, these investigations provide a comprehensive picture of the underlying alkyl‐type mechanism along with the factors governing the stereoselectivity and the singular and pivotal role of the anilido‐aldimine scaffold on the reactivity.

## Results and Discussion

### Complex Synthesis and Reaction Optimization

**Scheme 2:**
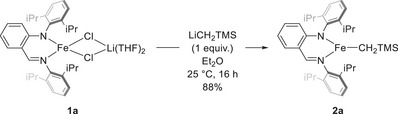
Salt metathesis synthesis of complex **2a**.

**Figure 1:**
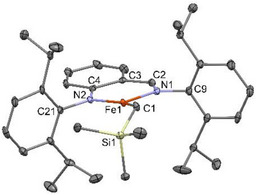
ORTEP drawing of complex **2a**. Thermal ellipsoids are shown at the 30% level. Only one molecule of the asymmetric unit is depicted.

We started our study with the synthesis of novel AA Fe(II) alkyl complex **2a** from our previously reported Fe(II) chloro‐ate complex **1a** (Scheme 2).^[^ ^59^ ^]^ Salt metathesis of **1a** with LiCH_2_TMS affords **2a** in 88% yield as a red air‐sensitive crystalline solid after cold crystallization in hexane. The molecular structure of **2a**, confirmed by single crystal X‐ray diffraction (Figure 1),^[^ ^60^ ^]^ showed a planar, three‐coordinate geometry around the Fe center with unequal Fe─N bond lengths (average ΔFe─N = 0.065 Å over the 2 molecules per asymmetric unit). The Fe─N and Fe─CH_2_TMS bond lengths are similar to those observed in a recently reported three‐coordinate isoelectronic amido‐imidazolin‐2‐imine Fe(II) alkyl complex and in previously reported three‐ and four‐coordinate β‐diketiminate (BDI) Fe(II) alkyl complexes.^[^ ^61^, ^62^, ^63^, ^64^, ^65^, ^66^, ^67^ ^]^ However, in contrast with the latter, the two C─N bonds of the ligand backbone have markedly different bond lengths (average C─N_amido_ 1.368 Å, average C─N_imine_ 1.298 Å), which hence induce dissimilar values for the two angles of the N‐aryl groups with the backbone (average C_Ar_─N─C_amine_ 119.1°, average C_Ar_─N─C_imine_ 117.5°). These differences reveal a more localized electron‐donor AA ligand over its parent BDI, which provides an asymmetric steric environment around Fe. The two N‐aryl groups are roughly perpendicular to the plane of the chelate backbone. The Evans magnetic moment of **2a** in a THF‐*d_8_* solution at 300 K was 5.2 μ_B_, a value that agrees with the spin‐only value expected for Fe(II) with a 3d^6^ configuration in an S = 2 high‐spin state.

With complex **2a** in hand, we first investigated its potential reactivity as a catalyst in the isomerization of allylbenzene **3a** in the presence of a hydride source. After variation of several reaction parameters, we were able to effectively perform the stereoselective monoisomerization of **3a** using only 5 mol% of **2a** and 5 mol% of Cy_2_NH·BH_3_ as a hydride source, affording the C═C transposed product **4a** in 98% yield and with *E*:*Z* ratio of 28:1 after 48 h at 60 °C (entry 1, Table 1). Representative results of our optimization study are shown in Tables 1 and S1. For instance, reducing the reaction time to 24 h decreased the relative *E*:*Z* stereoselectivity to 8:1 despite full conversion (entry 2, Table 1). In contrast to **C** (Scheme 1),^[^ ^54^ ^]^ 2 equiv. of hydride source related to the catalyst was not needed in our system, as the same outcome was noticed with 10 mol% of Cy_2_NH·BH_3_ instead of 5 mol% (entry 3). Worth mentioning is that, under our optimized conditions, **C** only afforded 10% conversion and 8% yield of **4a** (entry 4). The employ of other secondary or primary amine‐borane complexes (E·BH_3_, E = *i*Pr_2_NH, Me_2_NH, morpholine, *t*BuNH_2_, NH_3_) afforded diminished efficiency, while no isomerization was observed with Me_3_N·BH_3_ or Ph_3_P·BH_3_ (entries 1–7, Table S1). The use of 5 mol% of amine‐free hydride source 9‐borabicyclo(3.3.1)nonane (H‐9‐BBN) instead of Cy_2_NH·BH_3_ exhibited lower activity and stereoselectivity (entry 5). However, using the amine‐containing complex Me_2_NH·H‐9‐BBN restored good catalytic activity with similar selectivity, highlighting the key role played by the amine in the catalyst activity (entry 6). These compelling results underscore the crucial importance of the presence and the nature of the amine associated with the hydridoborane on the efficiency of the isomerization reaction of **3a** catalyzed by AA‐based complex **2a**. Investigations of the solvent effect on the reaction outcome led to the finding that the reaction performed equally well in toluene‐*d*_8_, methyl *tert*‐butyl ether (MTBE), *tert*‐amyl methyl ether (TAME), hexane, or heptane, providing **4a** in >95% yield (entries 10–13, Table S1).

**Table 1:** Reaction optimization for positional isomerization of allylbenzene.

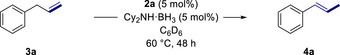				
Entry	Variations from optimized conditions^a)^	Conv (%)^b)^	Yield (%)^b)^	*E*:*Z* ^b)^
1	None	99	98	28:1
2	24 h instead of 48 h	99	99	8:1
3	10 mol% of Cy_2_NH·BH_3_	99	92	28:1
4	**C** instead of **2a**	10	8	−
5	H‐9‐BBN instead of Cy_2_NH·BH_3_	30	29	5:1
6	Me_2_NH·H‐9‐BBN instead of Cy_2_NH·BH_3_	95	88	7:1
7^c)^	[**2b**]_2_ ^d)^ instead of **2a** and Cy_2_NH·BH_3_	99	99	8:1
8	[**2b**]_2_ ^d)^ instead of **2a** and Cy_2_NH·BH_3_	99	99	29:1

^a)^ Reactions run on a 0.5 mmol scale at 60 °C for 48 h with **3a** (1.0 equiv.), **2a** (5 mol%), and Cy_2_NH·BH_3_ (5 mol%) in benzene (0.8 M) unless otherwise stated.

^b)^ Determined by ^1^H NMR using 1,3,5‐trimethoxybenzene as internal standard unless otherwise stated.

^c)^ 24 h.

^d)^ 2.5 mol%.

**Scheme 3:**
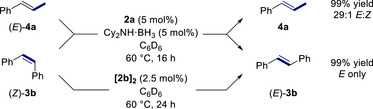
Catalytic reactivity of (*E*)‐**4a** and (*Z*)‐**3b** in geometrical isomerization.

Next, we examined the ability of our catalytic system to perform geometrical isomerization of stereochemically defined 1,2‐disubstituted alkenes (Schemes 3 and S2; Tables S2 and S3). To our delight, under the optimized catalytic conditions, *E*‐to‐*Z* interconversion and highly productive *Z*‐to‐*E* geometrical isomerization were observed as evidenced by the conversion of pure stereoisomer (*E*)‐**4a** into **4a** in *E*:*Z* ratio of 29:1 and the full conversion of stereo‐defined stilbene (*Z*)‐**3b** into stereoisomer (*E*)‐**3b**, respectively. These results highlight that our catalytic system performs efficiently not only the stereoselective position isomerization of terminal olefins but also the *E*‐to‐*Z* and *Z*‐to‐*E* geometrical isomerization of internal alkenes. To our knowledge, apart from stilbene, this is the first report of iron‐catalyzed geometrical isomerization of alkenes.^[^ ^47^, ^48^ ^]^

### Kinetic and Stereoselectivity Profiles

**Scheme 4:**
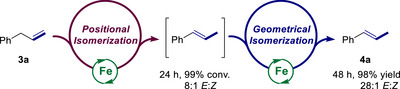
Overview of the tandem process occurring in the isomerization of **3a** catalyzed by **2a**.

To rationalize the enhancement of the stereoselectivity of the isomerization of **3a** catalyzed by **2a**/Cy_2_NH·BH_3_ (5 mol%) between 24 and 48 h (entries 1 and 2, Table 1), the kinetic and stereoselectivity profiles of the reaction were scrutinized. Monitoring reaction progress over a 48 h‐reaction time showed that the conversion and *E*:*Z* stereoselectivity (ratio (*E*)‐**4a**:(*Z*)‐**4a**) are correlated (Figures S1 and S2). Indeed, the selectivity remains constant at an *E*:*Z* ratio of 8:1 until the conversion reaches around >95% after 24 h. At this time, it increases gradually to attain the optimum value of 28:1 after around 33 h, a value that stays constant afterwards. This experiment stressed that a tandem process^[^ ^68^ ^]^ might operate as illustrated in Scheme 4. At first, a stereoselective Fe‐catalyzed positional isomerization performs until full conversion, then a more selective Fe‐catalyzed *Z*‐to‐*E* geometrical isomerization occurs, enhancing the overall stereoselectivity. To our knowledge, this is the first time that such an operating process was brought to light in alkene isomerization.

### Stoichiometric Reactivity of Complex 2a

**Scheme 5:**
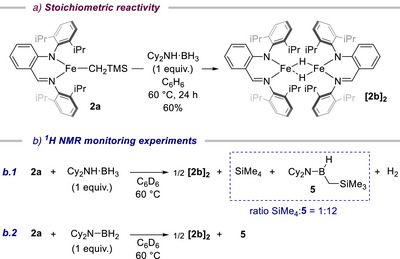
Stoichiometric reactivity of **2a** with Cy_2_NH·BH_3_ (A), ^1^H NMR monitoring experiments of the stoichiometric reaction of **2a** and Cy_2_NH·BH_3_ (B.1) and CyN‐BH_2_ (B.2).

**Figure 2:**
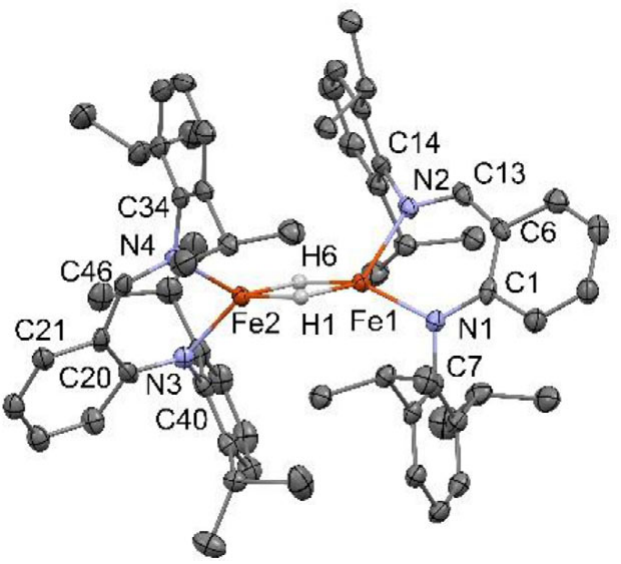
ORTEP drawing of complex **[2b]_2_**. Thermal ellipsoids are shown at the 30% level (hydrogen atoms of ligands are omitted for clarity). Fe(1)─Fe(2) distance = 2.6659(8) Å.

In order to get some insights into the molecular structure of the Fe intermediate generated from the reaction of **2a** and Cy_2_NH·BH_3_ and the underlying pathway of its formation, stoichiometric reactivity studies between these 2 partners were performed under conditions relevant to catalysis (Scheme 5). Before that, cyclic voltammetry of **2a** in THF was conducted and displays one reversible 1‐electron reduction peak at −1.82 V versus Ag/AgCl associated with an oxidation peak at −1.57 V versus Ag/AgCl (Figure S36). This low reduction potential value rules out the likely reduction of Fe(II)‐**2a** into Fe(I) by the amine‐borane reductant, as this was noticed by Webster and coworkers for BDI Fe(II) complex **C** (*E*_p,R_ = −1.37 V versus Ag/AgCl).^[^ ^54^ ^]^ First, stoichiometric addition of Cy_2_NH·BH_3_ to a stirred solution of **2a** in benzene led to the isolation of dimeric iron(II) hydride complex **[2b]_2_** as a brown crystalline solid in 60% yield after heating for 24 h at 60 °C (Scheme 5, A.).^[^ ^69^, ^70^ ^]^ X‐ray diffraction analysis of a single crystal of **[2b]_2_** revealed that each four‐coordinate Fe(II) atom adopts, in a solid state, a similar geometry that lies between a tetrahedral and a square planar (τ_4_(Fe1) = 0.5, τ_4_(Fe2) = 0.6) (Figure 2).^[^ ^60^, ^71^ ^]^ This akin coordination environment between the two Fe centers is in contrast to what was observed for previously reported dinuclear BDI Fe(II)‐H complexes bearing analogous *N*‐diisopropylphenyl or smaller *N*‐dimethylphenyl substituents, for which both Fe atoms reside in a distinct geometry.^[^ ^72^, ^73^, ^74^, ^75^ ^]^ Analysis of the metrical parameters reveals that the Fe─Fe distance [2.666 Å] is significantly longer than that found in hydride‐bridged BDI Fe(II) complexes [average 2.529 Å]^[^ ^72^, ^73^, ^74^, ^75^, ^76^, ^77^, ^78^, ^79^, ^80^ ^]^ or other related dimeric, four‐coordinate Fe(II) hydride^[^ ^61^, ^81^ ^]^ complexes.

Second, the in situ ^1^H NMR monitoring experiment of the stoichiometric reaction of **2a** and Cy_2_NH·BH_3_ at 60 °C in benzene‐*d*_6_ showed, in the paramagnetic region, the slow disappearance of the resonance signals of **2a** and the gradual appearance of a new set of peaks unambiguously assigned to **[2b]_2_** (Scheme 5, B.1 and Figures S3–S5). Full conversion was noticed after 300 min, reflecting a relatively slow process. Close examination of the diamagnetic region revealed, inter alia, the presence of characteristic signals that could not be attributed to the expected amino‐borane Cy_2_N‐BH_2_ product resulting from dehydrocoupling of Cy_2_NH·BH_3_ by **2a**, as confirmed by independent synthesis. Subsequent monitoring of the reaction of **2a** and amino‐borane CyN‐BH_2_ (1 equiv.) at 60 °C by ^1^H NMR showed a fast decay of **2a** signals and the emergence of new signals that match perfectly the resonances that would be expected from an equimolar mixture of **[2b]_2_** and **5** observed in the previous monitoring experiment (Scheme 5, B.2 and Figure S6). Consequently, the molecular structure of **5** was undoubtedly assigned as the product of σ‐bond metathesis of **2a** and the amino‐borane Cy_2_N‐BH_2_. In contrast to what was anticipated from previous works,^[^ ^74^ ^]^ the main pathway of **[2b]_2_** formation from **2a** under the catalysis conditions is not by amine‐borane dehydrocoupling but by σ‐bond metathesis of **2a** and the imine‐borane arising from the dehydroupling of Cy_2_NH·BH_3_ (see DFT calculations for more details), as evidenced by the 1:12 ratio of SiMe_4_/**5** measured in the ^1^H NMR monitoring experiment (Scheme 5, B.2).

### Catalytic Reactivity of Hydride‐Bridged Complexes

To our delight, under identical reaction conditions, the use of isolated **[2b]_2_** as a catalyst (2.5 mol%) afforded equal efficiency and stereoselectivity as **2a**/Cy_2_NH·BH_3_ in the transposition of **3a** and geometrical isomerization of stereodefined (*Z*)‐**3b** either after a 24 or 48 h reaction time (entries 7–8, Table 1 and Scheme 3). This underscores that **[2b]_2_** (or more likely its monomer) is a competent catalytic species of this tandem process in both positional and subsequent geometrical isomerization regimes. In line with previous finding,^[^ ^54^ ^]^ and in contrast to **[2b]_2_**, [(^Me^BDI^iPr^)Fe‐H]_2_ was not a competent catalyst to isomerize **3a** under these conditions, as only 13% and 32% yields of **4a** were obtained after reaction times of 48 h and 7 days, respectively.^[^ ^70^ ^]^

### Deuterium‐Labeling and EPR Experiments

**Scheme 6:**
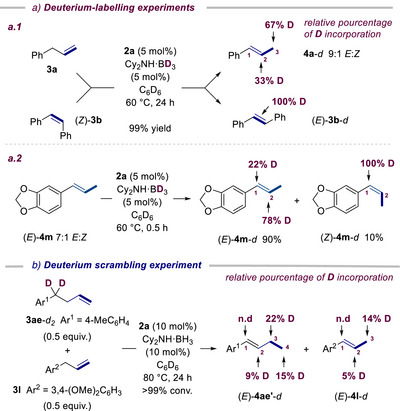
Deuterium‐labeling and scrambling experiments.

Performing the isomerization of **3a** and (*Z*)‐**3b** catalyzed by **2a** (5 mol%) in the presence of Cy_2_NH·BD_3_ (5 mol%) led to full conversion after 24 h and the observation of full deuterium (D)‐incorporation on both isomerized products, **4a** *‐d* (9:1 *E*:*Z*) and (*E*)‐**3b** *‐d* (Scheme 6, a.1). D‐incorporation occurred exclusively at carbons C_2_ and C_3_ of **4a** *‐d* and preferentially at the terminal C_3_ in a relative ratio of D‐incorporation at C_2_/C_3_ of 33/67. These experiments suggest the involvement of iron‐hydride (deuteride) species, which participates in a regioselective hydrometallation (deuterometallation) step. In addition, a scrambling experiment between deuterated compound **3ae**‐*d*_2_ (0.5 equiv.) and **3l** (0.5 equiv.) revealed both intra‐ and intermolecular H/D scrambling during the catalytic isomerization process (Scheme 6, B.). Collectively, these D‐labeling (including a complementary experiment in Figures S14 and S15) and crossover experiments are in favor of a metal‐hydride‐promoted alkyl‐type mechanism and argue against an allyl mechanism.^[^ ^82^ ^]^ Furthermore, measuring the D‐incorporation at the initial stage (0.5 h reaction time) of the *E*‐to‐*Z* interconversion of (*E*)‐**4m** (7:1 *E*:*Z*) catalyzed by **2a**/Cy_2_NH·BD_3_ (5 mol%) underlined that the degree of D‐incorporation at *C*_1_ and *C*_2_ is dependent on the stereoisomer formed (Scheme 6, A.2 and Figure S17 for other complementary experiments). Indeed, for (*E*)‐**4m** *‐d*, (90% yield), the incorporation occurred preferentially at *C*_2_ (*C*_1_/*C*_2_ ratio = 22/78) while for (*Z*)‐**4m** *‐d* (10% yield), it took place only at *C*_1_. This suggests the *E*:*Z* selectivity of the subsequent β‐H elimination step is correlated to the regioselectivity of the hydrometallation (see DFT calculations for more details). In situ EPR studies of the reactivity of **2b** (priorly isolated or in situ generated from **2a** and Cy_2_NH BH_3_) with respectively **3a** (20 equiv.) and (*Z*)‐**3b** (20 equiv.) at 25° and 60 °C were next conducted (Figures S20–S25).^[^ ^70^ ^]^ These EPR experiments evidence i) **[2b]_2_** (or its monomer) evolves through a hydrometallation in the presence of **3a** at 25° or 60 °C or in the presence of (*Z*)‐**3b** only at 60 °C, and ii) only traces of Fe(I), accounting for less than 1% of the total amount of iron species, were detected with no traces of Fe(III) species. This is in stark contrast to the reactivity of **C** with NH_3_·BH_3_ that shows an EPR signal of Fe(I) supporting an allyl‐type mechanism.^[^ ^54^ ^]^

### Kinetic Studies

To get some insights on the kinetic behavior of this Fe‐catalyzed isomerization of **3a**, several experiments were performed at different initial concentrations of **3a** and **2a**/Cy_2_NH·BH_3_. Thanks to the successive positional and geometrical regimes of the isomerization reaction, both regimes could be individually and independently monitored by ^1^H NMR spectroscopy. Variable Time‐Normalized Analyses (VTNA) of the progress concentration profiles of **3a** (for the positional regime) and (*Z*)‐**4a** (for the geometrical regime) at different excess experiments indicated for both regimes: i) a zeroth‐order rate dependence in alkene concentration (Figures S27 and S32) and ii) a nearly first‐order (1.2 and 1.1 for the positional and geometrical regimes, respectively) in catalyst concentration (Figures S28 and S33).^[^ ^83^, ^84^ ^]^ No catalyst deactivation or product inhibition over the entirety of the double‐bond migration manifold was also observed (Figure S26). The first‐order in catalyst is consistent with a monomeric iron species involved in the turnover‐limiting step, while the zeroth‐order in alkene supports a β‐H elimination, rather than a migratory insertion, as a rate‐determining step in the putative alkyl mechanism of both regimes. With the orders in alkene and catalyst in hand, rate constant (*k*_r_) values of 0.03 and 0.003 min^−1^ at 333 K could next be extracted for positional and geometrical regimes, respectively (Figures S29 and S34). Eyring analyses for the isomerization of **3a** over the two regimes provide the following activation parameters: Δ*H* ^‡^ = +15.85 kcal mol^−1^, Δ*S* ^‡^ = −18.3 cal mol^−1^ K^−1^ for the positional regime (Figures S30 and S31) and Δ*H* ^‡^ = +16.88 kcal mol^−1^, Δ*S* ^‡^ = −19.6 cal mol^−1^ K^−1^ for the *Z*‐to‐*E* interconversion regime (Figure S35). The magnitude of the negative Δ*S* ^‡^ values is reminiscent of a highly ordered structure of the transition states accompanying the rate‐limiting step.

### DFT Calculations

To further consolidate our mechanistic understanding of the plausible pathways of formation of competent species **[2b]_2_** from **2a** and Cy_2_NH·BH_3_ and its involvement in the isomerization of **3a** leading to **4a**, DFT calculations were performed. Systematic evaluation of the relative stability of **2a** in the singlet, triplet, and quintet spin states identifies the latter as the ground state, in agreement with what we observed experimentally and it was chosen as reference.

**Figure 3:**
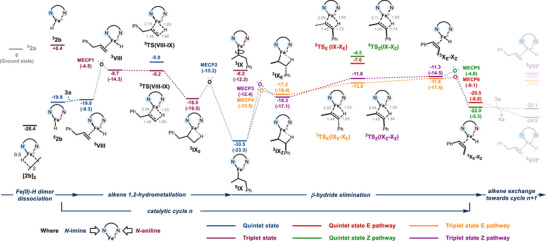
Calculated Gibbs energy profiles for the positional isomerization of **3a** from **[2b]_2_**. Energies are in kcal mol^−1^ at the B3PW91‐D3(C_6_H_6_)/TZVP//BP86/SDD/6‐31G** level of theory and relative to ^5^ **2a** and numbers in parentheses are in electronic energy. MECPs were identified using the Harvey et al.'s method and are expressed by means of electronic energy.

On the basis of ^1^H NMR monitoring experiments (Scheme 5, B.), two pathways for the formation of **[2b]_2_** from **2a** and Cy_2_NH·BH_3_ under the catalytic conditions could be proposed. The first and less dominant one arose from the anticipated dehydrocoupling of amine‐borane Cy_2_NH·BH_3_ by **2a** leading to the release of amino‐borane Cy_2_NBH_2_ concomitantly with **[2b]_2_** formation. The second and more prevailing one is the reaction of the resulting amino‐borane Cy_2_NBH_2_ and **2a** affording **[2b]_2_** and **5**.^[^ ^85^ ^]^ DFT calculations of these two pathways (Figure S39) confirmed that the second pathway of **[2b]_2_** formation is faster than the first one (ΔΔ*G* ^‡^ = 1.7 7 kcal mol^−1 [^ ^86^ ^]^) as noticed experimentally.^[^ ^70^ ^]^ Next, we turned our efforts toward the catalytic isomerization of **3a** catalyzed by **[2b]_2_** to shed light on the mechanistic pathway and origin of the observed stereoselectivity. On the basis of the collected experimental evidence, we postulated that the reaction proceeds by an alkyl‐type mechanism involving migratory 2,1‐insertion/β‐H elimination transition states. The DFT‐assessed Gibbs energy profile of the surveyed reaction is portrayed in Figure 3. First, exergonic dissociation of **[2b]_2_** followed by alkene coordination upon addition of **3a** results in the formation of intermediate **VIII**, which is more stable in the quintet (**^5^VIII**, Δ*G* = −19.0 kcal mol^−1^) than in the triplet spin state (**^3^VIII**, Δ*G* = −8.7 kcal mol^−1^). Subsequently, **^5^VIII** undergoes a regioselective migratory 2,1‐insertion of the C═C bond into the Fe─H bond, providing ^5^ **IX** that lies at −33.5 kcal mol^−1^.^[^ ^33^ ^]^ Interestingly, the triplet transition state **^3^TS(VIII‐IX)**, which adopts a pseudo square planar geometry at iron, was found to be more accessible by 4.2 kcal mol^−1^ than the quintet **^5^TS(VIII‐IX),** indicative of a two‐state reactivity^[^ ^87^, ^88^, ^89^, ^90^, ^91^ ^]^ with spin state acceleration.

Further progress along the triplet surface toward the key stereo‐determining β‐hydride elimination step delivers stereoisomeric intermediates **^3^IX*_E_*** and **^3^IX*_Z_***. These intermediates feature β‐agostic interactions, which pave the way for the subsequent β‐hydride elimination step. The latter is likely operating at the triplet surface as evidenced by the lower total energy barriers for the *E*‐selective **^3^TS*_E_* (IX*_E_*‐X*_E_*)** (Δ*G* ^‡^ = 20.5 kcal mol^−1^ relative to **^5^IX**) and *Z*‐selective **^3^TS*_Z_* (IX*_Z_*‐X*_Z_*)** (Δ*G* ^‡^ = 21.7 kcal mol^−1^ relative to **^5^IX**) in the triplet surface compared to those on the quintet surface. These triplet transition states adopt a pseudo square planar geometry at the metal center with likely an empty in‐plane d orbital to accept the two electrons of the incipient hydride. This is mandatory for β‐H elimination to occur.^[^ ^92^, ^93^ ^]^ This quintet to triplet spin surface cross unravels more accessible intermediates **^3^IX*_E_*** and **^3^IX*_Z_*** and low‐lying transition states **^3^TS*_E_* (IX*_E_*‐X*_E_*)** and **^3^TS*_Z_* (IX*_Z_*‐X*_Z_*)** inducing fast and stereo‐controlled catalysis. Such spin‐acceleration was previously reported for β‐hydride elimination promoted by BDI Fe(II) and Co(II) alkyl complexes and for Fe(I)‐catalyzed alkene transposition proceeding by an allyl‐type mechanism during the oxidative addition step.^[^ ^23^, ^52^, ^92^, ^93^, ^94^ ^]^ To our knowledge, this is the first illustration of spin‐accelerated alkyl mechanism in Fe‐catalyzed alkene isomerization.^[^ ^95^ ^]^ The energy gap between **^3^TS*_E_* (IX*_E_*‐X*_E_*)** and **^3^TS*_Z_* (IX*_Z_*‐X*_Z_*)** (ΔΔ*G* ^‡^ = 1.2 kcal mol^−1^), which reflects the *E*:*Z* stereoselectivity of the transposition step, corresponds to a 7:1 *E*:*Z* ratio and compares well with the experimentally observed 8:1 ratio during the positional isomerization phase of this tandem process. This finding indicates the outcome of the positional isomerization of **3a** is kinetically governed, specifically through the β‐hydride elimination step, which is the stereo‐ and rate‐determining step. Moreover, the magnitude of this stereoselectivity value is directly related to the spin state of transition states, as dissimilar stereoselectivity would have been noticed if the reaction went through the quintet manifold. With the report of Holland and coworkers on Co catalysis,^[^ ^23^ ^]^ this is a rare example of stereoselectivity being directly correlated to spin state changes. After **^3^TS*_E_* (IX*_E_*‐X*_E_*)** and **^3^TS*_Z_* (IX*_Z_*‐X*_Z_*)**, subsequent alkene‐hydride complexes **^3^IX*_E_*** (Δ*G* = −11.8 kcal mol^−1^) and **^3^IX*_Z_*** (Δ*G* = −11.3 kcal mol^−1^) decay into the quintet state. Further alkene exchanges liberate (*E*)‐**4a** and (*Z*)‐**4a** isomers and generate ^5^VIII*, ready for another catalytic cycle. To note, the DFT‐assessed overall barrier of 20.5–21.7 kcal mol^−1^ for turnover‐limiting β‐hydride elimination matches the empirically determined Eyring activation parameter Δ*G* ^‡^ = 21.9 kcal mol^−1^.

The migratory‐insertion and β‐hydride elimination steps for the **3a** isomerization catalyzed by [(^Me^BDI^iPr^)Fe‐H]_2_ were also assessed by DFT calculations (Figures S49 and S50). With this BDI dimer, a two‐state reactivity energetically facilitates the 2,1‐hydrometallation pathway over the spin‐hindered 1,2‐hydrometallation, that is ineffective for efficient transposition (Figure S49).^[^ ^70^ ^]^ This is in strong contrast to **2a** where spin crossover also gives access to the 1,2‐hydrometallation intermediate **^5^IX**, mandatory for subsequent productive isomerization (Figure 3). Stoichiometric reactivity of [(^Me^BDI^iPr^)Fe‐H]_2_ and **2a** led to the isolation and solid‐state characterization^[^ ^60^ ^]^ of 2,1‐hydrometallation intermediate **2i** in 81% with no detectable trace of its 1,2‐regioisomer **2j** (Figure S46).^[^ ^96^ ^]^ In situ ^1^H NMR monitoring of the reaction of [(^Me^BDI^iPr^)Fe‐H]_2_ (2.5 mol%) and **2a**, under conditions relevant to catalysis, supports that **2i** is likely the catalyst resting state (Figure S48).

Moreover, these DFT studies highlight the 1,2‐hydrometallation transition state, and the subsequent β‐H elimination transition states for both isomers are lower by at least 5 kcal mol^−1^ for [**2b**]_2_ compared to those for [(^Me^BDI^iPr^)Fe‐H]_2_ on the more accessible spin state surface (Figures 3 and S50).^[^ ^70^ ^]^ Collectively, these investigations suggest that, to a higher extent than BDI, the AA skeleton has the ability to i) substantially lower the energy of the triplet spin state in both alkene insertion and β‐H elimination transition states, making them energetically accessible, and ii) access and stabilize pseudo square planar geometry at the Fe(II) center.^[^ ^97^ ^]^ This ability opens the door to the 1,2‐hydrometallation pathway, which is compulsory for productive alkene transposition of terminal alkenes, and favors spin‐acceleration in the rate‐ and stereo‐determining β‐H elimination step. This feature emphasizes the singularity of the AA over the BDI ligand and the novel or enhanced reactivity patterns that this ligand can embrace in iron catalysis, as we demonstrated in C(sp^3^)─C(sp^2^) and C(sp^3^)─C(sp^3^) Suzuki–Miyaura cross‐coupling.^[^ ^59^ ^]^

**Scheme 7:**
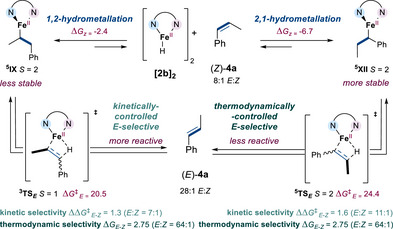
A comprehensive overview of the origin of the stereoselectivity in the geometrical isomerization regime. Energies are in kcal mol^−1^ at the B3PW91‐D3(C_6_H_6_)/TZVP//BP86/SDD/6‐31G** level of theory.

Ultimately, we set out to streamline the selectivity resulting from the subsequent geometrical isomerization. We hypothesized the observed increase in stereoselectivity (from 7:1 to 28:1 *E*:*Z*) upon complete depletion of **3a** might be ascribed to the emergence of a competitive pathway that is more *E*‐selective than the pathway of formation of (*E*)‐**4a** from 1,2‐hydrometallation intermediate **^5^IX** (Figure 3 and Scheme 7). To probe this hypothesis, the two pathways of geometrical isomerization of (*Z*)‐**4a** into (*E*)‐**4a** through 1,2‐ and 2,1‐hydrometallation were studied by DFT calculations (Figures S44 and S45 for profiles).^[^ ^70^ ^]^ The study highlights that intermediate **^5^XII**, arising from 2,1‐hydrometallation of (*Z*)‐**4a** by **^5^2b**, was more stable than **^5^IX**, arising from 1,2‐hydrometallation, by means of 4.3 kcal mol^−1^ (Scheme 7). Nevertheless, the subsequent reversible and selective‐determining β‐H elimination operated through a substantially lower energy barrier (ΔΔ*G* ^‡^ *_E_* = 3.9 kcal mol^−1^) from less stable intermediate **^5^IX** than more stable intermediate **^5^XII**.^[^ ^98^ ^]^ Moreover, as determined for the positional isomerization of **3a** (Figure 3), the stereoselectivity of the β‐H elimination from **^5^IX** is under kinetic control, affording (*E*)‐**4a** with a 7:1 *E*:*Z* ratio (ΔΔ*G* ^‡^ *_E_*_‐_ *_Z_* = 1.3 kcal mol^−1^ at the more accessible triplet surface). In contrast, the DFT calculations and the observation of a 28:1 *E*:*Z* selectivity at the end of the geometrical isomerization regime suggested the β‐H elimination stereoselectivity from the second regioisomer **^5^XII** is thermodynamically driven, resulting in an *E*:*Z* distribution of a 64:1 ratio for a Δ*G_E_*_‐_ *_Z_* value of 2.75 kcal mol^−1^ (at the more accessible quintet surface). This calculated value is of the same order of magnitude as that previously reported.^[^ ^44^ ^]^ In summary, the stereoselectivity outcome of the geometrical isomerization of (*Z*)‐**4a** from 7:1 to 28:1 *E*:*Z* arose from the concomitant and unbalanced contribution of the less selective and stable, but more reactive, 1,2‐regioisomer **^5^IX** and the more selective and stable, but less reactive, 2,1‐regioisomer **^5^XII**.

**Scheme 8:**
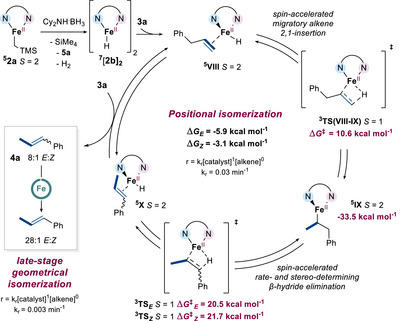
Proposed mechanism based on experimental and computational studies.

To summarize our experimental and theoretical findings thus far, the herein reported Fe(II)‐catalyzed isomerization likely involves an in situ generated high‐spin Fe(II)‐hydride **2b** (from **2a** and Cy_2_NH.BH_3_) as active species and proceeds via a redox‐neutral alkyl‐type mechanism, for which reversible, migratory 2,1‐alkene insertion and rate‐ and stereo‐determining β‐H elimination steps feature a two‐state reactivity leading to spin‐acceleration (Scheme 8). During the course of the positional isomerization regime, which occurs until full conversion of **3a** into **4a**, β‐H elimination step from **^5^IX** at the triplet manifold led to a moderate *E*:*Z* ratio of 8:1. Subsequently, a late‐stage geometrical isomerization regime, also promoted by **2b**, enhanced the selectivity, affording the ultimate *E*:*Z* ratio of 28:1 at the end of the reaction.

### Substrate Scope

**Scheme 9:**
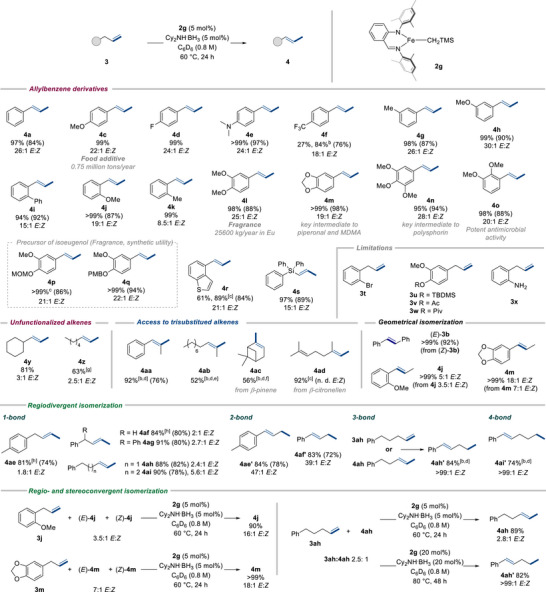
Scope of Fe‐catalyzed isomerization.[a] [a] reactions run on a 0.5 mmol scale at 60 °C for 24 h with alkene (1.0 equiv.), Cy_2_NH.BH_3_ (5 mol%), **2g** (5 mol%) in benzene (0.8 M) unless otherwise stated. Yield and *E:Z* determined by ^1^H NMR using 1,3,5‐trimethoxybenzene as an internal standard and isolated yield in parentheses. [b] Reactions run on a 0.5 mmol scale at 80 °C for 24 h with alkene (1.0 equiv.), Cy_2_NH.BH_3_ (20 mol%), **2g** (20 mol%) in benzene (0.8 M). [c] Run with Cy_2_NH.BH_3_ (10 mol%), **2g** (10 mol%). [d] 48 h. [e] 76% conversion. [f] 20% of the side product is detected. [g] Run with **2a**. [h] 3 h instead of 24 h.

Next, we set out to explore the generality and limitations of our methodology (Scheme 9). Priorly, catalyst structure optimization (Table S7) led to the identification of **2g** as a four‐fold more active catalyst than **2a** without any loss of stereoselectivity (Figure S41). Indeed, under our optimized conditions, **2g** afforded full conversion of **3a** into (*E*)‐**4a** in only 24 h (*k*_r_ = 0.12 min^−1^) versus 48 h for **2a** (*k*_r_ = 0.03 min^−1^) and with a 26:1 *E*:*Z* ratio. With a more active catalyst in hand, we first examined a range of allylbenzene analogues with a diverse set of functional groups under our general reaction conditions. Allylbenzene derivatives with an electron‐donating (**3c**, **3e**, **3h**), electron‐withdrawing (**3d**, **3f**) or electron‐neutral (**3g**) mono‐substituent in *para*‐ or *meta*‐ position relative to the allyl moiety afforded styrenyl products (**4c**, **4d**, **4e**, **4f**, **4g**, **4h**) in excellent yields (>98%, except **4f** 84%) and stereoselectivities (>22:1 *E*:*Z*, except **4f** 18:1 *E*:*Z*). Sterically constrained *ortho*‐substituted derivatives (**3i**, **3j**, **3k**) also furnished very high yields of isomerized products (**4i**, **4j**, **4k**) despite diminished selectivities (up to 19:1 *E*:*Z*). Synthetically and industrially relevant poly‐substituted allylbenzenes, including methyl isoeugenol **4l**, isosafrole **4m**, isoelemicin **4n**, methyl iso‐*ortho*‐eugenol **4o**, and MOM‐ and PMB‐protected iso‐eugenol **4p** and **4q** (that could potentially lead to catalyst inhibition or deactivation), were also prepared in excellent yields (95%→99%) and *E*:*Z* ratios ranging from 19:1 to 28:1, highlighting the versatility of our methodology. Moreover, our protocol was not limited to allylbenzene derivatives, as 4‐allylbenzo[*b*]thiophene **3r** and allyl triphenylsilane **3s** were successfully converted into **4r** and **4s** in 89% (21:1 *E*:*Z*) and 97% yield (15:1 *E*:*Z*), respectively. Nevertheless, it was not compatible with *ortho*‐bromide‐substituted allylbenzene (**3t**), TBDMS‐, acetyl‐, or pivaloyl‐protected iso‐eugenol (**3u**, **3v**, **3w**) or 2‐allylaniline (**3x**).^[^ ^70^ ^]^ Unfunctionalized non‐aryl substituted olefins such as allyl cyclohexane **3y** and 1‐octene **3z** provided selectively monoisomerized products **4y** and **4z** in 81% and 63% yield, respectively, regardless of lower stereoselectivities.

Trisubstituted alkenes are common building blocks in natural products and pharmaceuticals.^[^ ^99^ ^]^ In contrast to Co and Ni,^[^ ^24^, ^25^, ^28^, ^30^, ^33^, ^35^, ^39^, ^40^, ^41^ ^]^ the application of alkene isomerization to access trisubstituted analogues is largely unexplored under Fe catalysis.^[^ ^53^, ^56^, ^57^ ^]^ To evaluate the underlying potential of our methodology, monoisomerization of 1,1‐disubstituted olefins into challenging trisubstituted alkenes was next assessed. The use of a 20 mol% catalyst loading in **2g** in conjunction with 20 mol% of Cy_2_NH**^.^** BH_3_ was effective to furnish **4aa** in excellent yields (92%) after 48 h at 80 °C. Under these conditions, isomerization of 2‐methylundec‐1‐ene (**3ab**) and β‐pinene (**3ac**) was successful, providing **4ab** and α‐pinene **4ac**, respectively, in moderate yields. Milder reaction conditions could be applied to convert β‐citronellen (**3ad**) into the desired trisubstituted product **4ad** in excellent yield (92%) after only 24 h at 60 °C. To our delight, our iron‐based methodology could be efficiently applied not only to isomerize stereo‐defined stilbene (*Z*)‐**3b** into its purely *E*‐isomer but also to stereoisomerically enrich an alkenic compound with poor stereoselectivity. Indeed, configurational isomerization of **4j** (3.5:1 *E*:*Z*) and commercially available isosafrole **4m** (7:1 *E*:*Z*) resulted in an *E*:*Z* stereoselectivity increase of up to 18:1 for **4m**. To our knowledge, this is the first report of iron‐catalyzed geometric isomerization of aryl‐alkyl disubstituted olefins.

The precise control of the C═C bond relocation over one or multiple positions on the constitutional skeleton is another longstanding challenge to tackle in positional isomerization. To our knowledge, only two reports disclosed the controlled alkene transposition over one‐ and over multiple‐positions in Fe‐catalyzed isomerization‐only processes.^[^ ^53^, ^55^ ^]^ However, they either required the use of two distinct catalytic systems or displayed moderate stereoselectivity (<30:1 *E*:*Z*) with the use of a highly sophisticated catalyst. In contrast, our catalyst **2g** has the unique ability i) to perform both one‐bond and multiple‐bond translocation with *E*:*Z* selectivity up to >99:1, and ii) to distinguish between monoisomerization and chain‐walking, offering a simple approach for regiodivergent isomerization. Indeed, terminal alkenes **3ae**, **3af**, **3ag**, **3ah**, and **3ai** bearing distinctive extended carbon chains could be selectively monoisomerized into internal alkenes **4ae**, **4af**, **4ag**, **4ah**, and **4ai** in good yields (81%–91% yields) and *E*:*Z* ratios up to 5.6:1. However, when changing the reaction conditions, a selective two‐double bond shift was attained for **3ae** and **3af**, affording **4ae’** and **4af’**, respectively, while a selective three‐ and four‐bond migration was reached for **3ah** and **3ai** leading to chain‐walking products **4ah’** and **4ai’**, respectively (Scheme 9). All of these multiple‐bond translocations led to good yields of isomerized products with high selectivities (39:1 to >99:1 *E*:*Z*). It is pertinent to note that **4ah’** could also be obtained with the same efficiency starting from internal alkene **4ah,** emphasizing the compatibility of our methodology with internal alkene substrates. The competency of **2g** to promote both positional and geometrical isomerization offers the opportunity to convert regio‐ and stereoisomeric alkene mixtures into a single regio‐ and stereoisomer. This could be a valuable asset to transform raw isomeric alkene mixtures into a high‐value single isomer. In this respect, stoichiometric mixtures of **3j** and (*E/Z*)‐**4j** (3.5:1 *E*:*Z*) and **3m** and (*E/Z*)‐**4m** (7:1 *E*:*Z*) were successfully converted into the corresponding internal alkenes **4j** (16:1 *E*:*Z*) and **4m** (18:1 *E*:*Z*) in 90% and >99% yield, respectively. Interestingly, such a process could also be effectively applied to alkene **3ah** having an extended carbon chain, with control of the isomerization regime as either monoisomerization or chain‐walking. Indeed, depending on the reaction conditions, a mixture of **3ah** and **4ah** could be transformed either into monoisomerized product **4ah** or into chain‐walking product **4ah’** with high control of the alkene relocation.

## Conclusion

In summary, we have developed a readily accessible, well‐defined anilido‐aldimine Fe(II) alkyl catalyst that demonstrates high efficiency and versatility in alkene isomerization. For the first time, we accomplish a single catalyst that can promote tunable and stereocontrolled C═C bond migration over one and multiple positions along the carbon chain, and not previously reported with iron catalysis—stereocontrolled geometrical alkene isomerization. Remarkably, the protocol requires only 1 equiv. of a hydride source (relative to Fe), operates under mild reaction conditions, and isomerizes the following functionalized alkenes with high regio‐ and stereoselectivities: (hetero)aryl‐substituted terminal alkenes, aliphatic terminal alkenes, and 1,1‐ and 1,2‐disubstituted aliphatic alkenes. Thanks to the peculiar catalyst ability, regiodivergent and regio‐ and stereoconvergent olefin isomerization processes were also successfully implemented, underlining the great potential of our methodology to reach molecular diversity and to convert low‐cost olefin feedstocks into high‐value single isomers.

A comprehensive mechanistic study of the isomerization of allylbenzene (D‐labeling, in situ ^1^H NMR, EPR, kinetic, and stoichiometric experiments and DFT calculations) advocates in favor of a redox‐neutral alkyl mechanism involving an active and isolable Fe(II)‐H species. This species is mainly generated from an unexpected and unprecedented σ‐bond metathesis of catalyst **2a** and the amino‐borane arising from the dehydrocoupling of Cy_2_NH·BH_3_. Notably, the reaction performs via an original tandem process encompassing two independent and consecutive regimes. First, a selective one‐bond shift occurs until full completion, then a more selective *Z*‐to‐*E* geometrical isomerization operates, which improves significantly the stereoselectivity of the overall transformation. The transposition regime proceeds through a stereo‐ and rate‐determining β‐hydride elimination step that exhibits a two‐state reactivity, where the spin state changes lower the energy of the transition states (spin‐acceleration) and dictate the stereoselectivity outcome. To our knowledge, this current work is the first illustration of spin‐accelerated alkyl mechanism in Fe‐catalyzed alkene isomerization^[^ ^95^ ^]^ and a rare example of stereoselectivity correlated to spin crossover^[^ ^23^ ^]^ in a well‐defined metal‐catalyzed process. The increase in selectivity during the geometrical isomerization regime arises from the relative contribution of the stereo‐determining β‐hydride elimination steps of the two hydrometallation regioisomers. One of these is kinetically controlled, while the other is thermodynamically controlled. This study elucidated the central role of the anilido‐aldimine framework in this spin‐accelerated process, stressing its singularity and benefits over structurally‐related β‐diketiminates. Current efforts are focused on exploiting these ligand peculiarity and spin‐accelerated alkyl mechanism in other related iron catalytic processes.

## Conflict of Interests

The authors declare no conflict of interest.

## Supporting information



Supporting Information

Supporting Information

Supporting Information

## Data Availability

The data that support the findings of this study are available in the supplementary material of this article.
