# Supplementary material for: Iron‐Catalyzed Tunable Double Bond Migration and Geometrical Isomerization in Olefins via a Spin‐Accelerated Alkyl Mechanism
Source: Angew Chem Int Ed Engl. 2025 Oct 16;64(50):e202519729. doi: 10.1002/anie.202519729 (PMC12684360; doi:10.1002/anie.202519729)

Iron-Catalyzed Tunable Double Bond Migration and Geometrical Isomerization in Olefins via a Spin-accelerated Alkyl Mechanism

Abdul Halim Obeid, Christian Herrero, Régis Guillot, and Jérôme Hannedouche**

Université Paris Saclay, CNRS
Institut de Chimie Moléculaire et des Matériaux d'Orsay (ICMMO), UMR 8182
Bâtiment Henri Moissan, 17 avenue des Sciences, 91400 Orsay, France

E-mail: abdul-halim.obeid@universite-paris-saclay.fr
jerome.hannedouche@universite-paris-saclay.fr

Supporting Information

Table of Contents

1-General considerations **2**

2-Synthesis of Anilido-Aldimine (AA) ligands **3**

3-Synthesis of Anilido-Aldimine (AA) Fe(II) chloro-ate complexes **8**

4-Synthesis of Anilido-Aldimine (AA) Fe(II) alkyl and Hydride complexes **9**

5-Optimization studies and control experiments **13**

5a-Optimization studies 13

5b-Control experiments 14

6-Mechanistic studies **16**

6a-Kinetic and stereoselectivity profiles 16

6b-Stoichiometric reactivity of complex **2a** 18

6c-Catalytic reactivity of hydride complexes 20

6d-Radical-trapping experiments 22

6e-Deuterium-labelling experiments 25

6f-EPR experiments 35

6g-Kinetic studies 39

6h-Cyclic Voltammetry 47

6i-DFT calculations 49

6i- Mechanistic insights on (^{Me}BDI^{iPr})Fe(II)-catalyzed isomerization of allylbenzene **3a** 57

7-Catalyst structure optimization **63**

8-Time course studies with complex 2g **64**

9-Catalytic Procedures for Fe-catalyzed isomerization of alkene and characterization data **66**

10-X-ray Single Crystal Data of Fe complexes **77**

12-References **89**

13-NMR spectra **91**

1-General considerations

All manipulations were carried out under an inert atmosphere by using standard Schlenk-line techniques or in an Ar-filled glovebox (O_2 level <2.0 ppm). All glassware was stored in a pre-heated oven prior to use. THF and diethyl ether were distilled from sodium benzophenone ketyl, degassed by freeze-pump-thaw method and stored over activated 3Å molecular sieves. Benzene-d_6 was dried over CaH_2, degassed by freeze-pump-thaw method and transferred under vacuum. Toluene, *tert*-amyl methyl ether (TAME), methyl *tert*-butyl ether (MTBE), heptane, and hexane were distilled over CaH_2, degassed by freeze-pump-thaw method and stored over activated 3Å molecular sieves. Anhydrous iron(II) chloride (beads, ~10 mesh, 99.99%) was purchased from Sigma-Aldrich and used as received. Solid $LiCH_2SiMe_3$ was obtained by cold-recrystallization from a 1M pentane solution purchased from Sigma-Aldrich. *n*-BuLi (1.6 or 2.5 M in hexanes) was purchased from Sigma-Aldrich. 2-fluorobenzaldehyde (97%) were purchased from Fluorochem, 2-fluoro-4-methoxybenzaldehyde (97%) and 2-fluoro-5-methoxybenzaldehyde (98%) from ABCR, 2,5-difluorobenzaldehyde (98%) from Sigma-Aldrich, 2,6-diisopropylaniline (90%) from TCI, 2,4,6-trimethylaniline (98%) from Sigma-Aldrich and were used as received. Allylbenzene **3a** (TCI, >98%), (*Z*)-1,2-diphenylethene **3b** (Sigma-Aldrich, 96%), 1-allyl-4-methoxybenzene **3c** (Sigma-Aldrich, 98%), 1-allyl-4-fluorobenzene **3d** (Sigma-Aldrich, 97%), 1-allyl-3-methylbenzene **3g** (Sigma-Aldrich, 97%), 1-allyl-3-methoxybenzene **3h** (BLDpharm, 97%), 1-allyl-2-methoxybenzene **3j** (Sigma-Aldrich, 96%), 1-allyl-2-methylbenzene **3k** (Sigma-Aldrich, 97%), 5-allylbenzo[*d*][1,3]dioxole **3m** (Sigma-Aldrich, >97%), allyltriphenylsilane **3s** (Sigma-Aldrich, 98%), 1-allyl-2-bromobenzene **3t** (Sigma-Aldrich, 95%), 4-allyl-2-methoxyphenyl acetate **3v** (BLDpharm, 98%), allylcyclohexane **3y** (TCI, >97%), 1-octene **3z** (Fluka), (2-methylallyl)benzene **3aa** (Fischer Scientific Chemicals, 99%), 2-methylundec-1-ene **3ab** (Sigma-Aldrich, 97%), 2,6,6-trimethylbicyclo[3.1.1]hept-2-ene **3ac** (Alfa Aesar, 99%), 3,7-dimethylocta-1,6-diene **3ad** (Fluka), 4-phenyl-1-butene **3af** (TCI, >98%), isosafrole **4m** (Fluka, mixture of isomers 7:1), *cis*-1,2,3,3a,4,6a-allylboronic acid pinacol ester **3aj** (Sigma-Aldrich, 97%), 1-methyl-4-(prop-1-en-2-yl)cyclohex-1-ene **3al** (Fluka), 3-(allyloxy)prop-1-ene **3am** (Sigma-Aldrich, 98%) and diallyldimethylsilane **3an** (Sigma-Aldrich, 98%) were purchased, dried over CaH_2 and transferred under vacuum prior storage in the glovebox. 4-allyl-*N*,*N*-dimethylaniline **3e**, 1-allyl-4-(trifluoromethyl)benzene **3f**, 2-allyl-1,1'-biphenyl **3i**, and 4-allylbenzo[*b*]thiophene **3r** were prepared according to literature procedure,[1] dried over CaH_2, entered in the glovebox and passed through a Millipore filter prior to storage. Syntheses of 4-allyl-1,2-dimethoxybenzene **3l**,[2] 5-allyl-1,2,3-trimethoxybenzene **3n**,[2] 1-allyl-2,3-dimethoxybenzene **3o**,[2] 1-methoxy-2-(prop-1-en-1-yl)benzene **4j**,[2] 4-allyl-2-methoxy-1-(methoxymethoxy)benzene **3p**,[3] 4-allyl-2-methoxy-1-((4-methoxybenzyl)oxy)benzene **3q,**[3] (4-allyl-2-methoxyphenoxy)(tert-butyl)dimethylsilane **3u**,[1] (but-3-en-1-yl)-4-methylbenzene **3ae**,[4] but-3-ene-1,1-diyldibenzene **3ag**,[5] pent-4-en-1-ylbenzene **3ah**,[4] and hex-5-en-1-ylbenzene **3ai**[4], 2-allylaniline **4x**[6a] and 2-(hex-5-en-1-yl)-2-methyl-1,3-dioxolane

3ak[6b] were adapted from literature procedures, and these compounds were dried over CaH_2, entered in the glovebox and passed through a Millipore filter prior to storage. 4-allyl-2-methoxyphenyl pivalate **3w**[7] was prepared from eugenol by *O*-acylation, dried over P_2O_5 prior to storage in the glovebox. **(^{Me}BDI^{iPr})Fe-CH$_2$TMS** (complex **C**) and **[(^{Me}BDI^{iPr})Fe-H]$_2$** were prepared according to literature procedure[8] and the latter was isolated from a benzene solution by recrystallisation at room temperature. Silica gel (Merck, type 60, 0.04–0.063 mm) was used for column chromatography.
Amine borane (Sigma-Aldrich, 95%), dimethylamine borane (Sigma-Aldrich, 95%), *tert*-butylamine borane (Sigma-Aldrich, 97%), trimethylamine borane (Sigma-Aldrich, 97%), Phosphine borane (Sigma-Aldrich, 97%) 9-borabicyclo[3.3.1]nonane (Fluorochem, 95%) and pinacol borane (Sigma-Aldrich, 97%) were purchased and used as received. The syntheses of dicyclohexylamine borane, diisopropylamine borane and morpholine borane were adapted from a literature procedure.[10] Dicyclohexylamino borane was synthetized according to a literature procedure, extracted by cold hexane then dried under reduced pressure prior to use.[11] Dimethylamine 9-borabicyclo(3.3.1)nonane complex was prepared from a procedure adapted from literature.[12]

^{1}H, ^{13}C and ^{19}F NMR data were recorded on Bruker AV 600, Bruker AV 400, Bruker AV 300 or Bruker DPX 250 spectrometers at 22 °C (unless otherwise stated). Chemical shifts for ^{1}H NMR spectra were referenced internally according to the residual protiated solvent resonances in $CDCl_3$ (δ 7.26 ppm) and in benzene-d_6 (δ 7.16 ppm). Chemical shifts for ^{13}C NMR spectra were referenced internally according to the residual solvent resonances in $CDCl_3$ (δ 77.16 ppm) and in benzene-d_6 (δ 128.06 ppm). ^{13}C NMR recorded spectra of paramagnetic AA Fe(II) complexes showed an inadequate number of peaks and were not described. Coupling constants were reported in hertz (Hz) and multiplicities were abbreviated as following: br (broad signal), s (singlet), d (doublet), t (triplet), q (quadruplet), quint (quintet), sp (septet), m (multiplet), dd (doublet of doublets), dt (doublet of triplets), td (triplet of doublets), tt (triplet of triplets), dq (doublet of quadruplets), tdd (triplet of doublet of doublets). Elemental analyses were performed by the analytische Laboratorien GMBH in Lindlar, Germany and the CHN analysis service from Laboratoire de Chimie de coordination (LCC) in CNRS, Toulouse, France. High-resolution mass spectra were recorded on MicrOTOFq Bruker spectrometer by electrospray ionization (ESI) or atmospheric pressure chemical ionization (APCI).

2-Synthesis of Anilido-Aldimine (AA) ligands

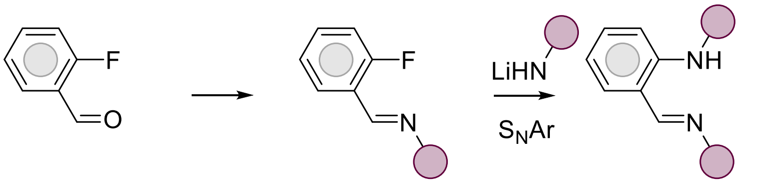

I-2a–I-2g **AA-2a–AA-2g**

Scheme S1. Overview of the synthesis of AA Ligands **AA-2a-AA-2g**

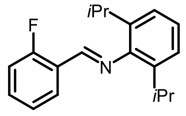

I-2a

(*E*)-*N*-(2-fluorobenzylidene)-2,6-diisopropylaniline (I-2a): 2,6-diisopropylaniline (11.2 mL, 59.8 mmol) was added to a stirred solution of 2-fluorobenzaldehyde (6 mL, 57 mmol) and $MgSO_4$ (2.0 g) in hexane (30 mL) at room temperature. The solution was stirred for 16 h. Then, the solution was filtered, concentrated under reduced and cooled to –20 °C to afford **I-2a** as yellow crystalline solid (15.2 g, 53.7 mmol, 94%). **^{1}H NMR** (400 MHz, $CDCl_3$, RT) δ 8.58 (s, 1H, ArC*H*N), 8.29 (td, 1H, *J* = 7.5, 1.8 Hz, *H* Ar), 7.57-7.51 (m, 1H, *H* Ar), 7.33 (t, 1H, *J* = 7.5 Hz, *H* Ar), 7.24-7.15 (m, 3H, *H* Ar), 3.02 (sp, 2H, *J* = 6.9 Hz, $(Me)_2CH$), 1.24 (d, 12H, *J* = 6.9 Hz, $(Me)_2CH$). **^{13}C{^{1}H} NMR** (100 MHz, $CDCl_3$, RT) δ 162.9 (d, J_{CF} = 253 Hz), 155.8 (d, J_{CF} = 5 Hz), 149.4, 137.7, 133.1 (d, J_{CF} = 9 Hz), 127.9 (d, J_{CF} = 2.6 Hz), 124.7 (d, J_{CF} = 3.4 Hz), 124.5, 123.8 (d, J_{CF} = 9 Hz), 123.2, 116.1 (d, J_{CF} = 21 Hz), 28.1, 23.6. **HRMS** (ESI) *m/z* calcd for $C_{19}H_{23}NF$ $[M+H]^+$ 284.1809. Found: 284.1798. These data are in agreement with our previously reported data.[9]

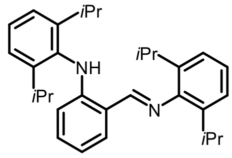

AA-2a

(*E*)-*N*-(2-((2,6-diisopropylphenyl)amino)benzylidene)-2,6-diisopropylaniline (AA-2a): n-BuLi (7 mL, 17.5 mmol) was added to a stirred solution of 2,6-diisopropylaniline (3.3 mL, 17.5 mmol) in THF (20 mL) at -78 °C. The solution was stirred overnight and then, was cannulated to a stirred solution of (*E*)-*N*-(2-fluorobenzylidene)-2,6-diisopropylaniline (**I-2a**) (3.3 g, 11.6 mmol) in THF (15 mL) at room temperature. The solution was stirred for 2 h. Then, water (20 mL) was added. The aqueous layer was separated and extracted with Et_2O (3 x 30 mL). Organics layers were combined, dried over anhydrous $MgSO_4$, filtered and concentrated under reduced pressure to give a light brown oil. The brown oil obtained was recrystallized in ethanol at room temperature to afford **AA-2a** as pale yellow crystalline solid (3 g, 6.8 mmol, 59%). **^{1}H NMR** (400 MHz, $CDCl_3$, RT) δ 10.46 (br s, 1H, N*H*), 8.29 (s, 1H, ArC*H*N), 7.29-7.03 (m, 8H, *H Ar*), 6.63 (t, 1H, *J*= 7.4 Hz, *H Ar*), 6.21 (d, 1H, *J* = 8.4 Hz, *H Ar*), 3.14 (sp, 2H, *J* = 6.9 Hz, $(CH_3)_2CH$), 3.03 (sp, 2H, *J* = 6.9 Hz, $(CH_3)_2CH$), 1.10 (d, 12H, *J* = 6.9 Hz, $(CH_3)_2CH$), 1.08 (d, 6H, *J* = 6.9 Hz, $(CH_3)_2CH$), 1.06 (d, 6H, *J* = 6.9 Hz, $(CH_3)_2CH$), **^{13}C{^{1}H} NMR** (100 MHz, $CDCl_3$, RT) δ 165.9, 150.1, 148.8, 147.7, 138.3, 134.7, 134.6, 132.3, 127.6, 124.5, 123.9, 123.2, 116.3, 115.3, 112.1, 28.7, 28.2, 24.7, 23.7, 23.2. **HRMS** (ESI) *m/z* calcd for $C_{31}H_{41}N_2$ $[M+H]^+$ 441.3264. Found: 441.3247. These data are in agreement with our previously reported data.[9]

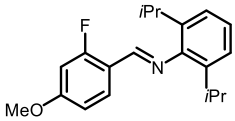

I-2d

(*E*)-*N*-(2-fluoro-4-methoxybenzylidene)-2,6-diisopropylaniline (I-2d): In a Dean-Stark apparatus, *p*-toluene sulphonic acid (0.032 g, 0.168 mmol) was added to a stirred solution of 2-fluoro-4-methoxybenzaldehyde (2.5 g, 16.2 mmol) and 2,6-diisopropylaniline (3.4 mL, 17.8 mmol) in toluene (40 mL). The solution was heated at 150 °C overnight. Then, the solution was cooled at room temperature and a saturated solution of Na_2CO_3 (20 mL) was added. The aqueous layer was separated and extracted with Et_2O (3 x 20 mL). Organics layers were combined, dried over anhydrous $MgSO_4$, filtered and the solvent was removed under reduced pressure to obtain light yellow oil. This oil was dissolved in hexane and cooled to –20 °C to afford **I-2d** as yellow crystalline solid (4.2 g, 13.4 mmol, 83%). **^{1}H NMR** (400 MHz,

$CDCl_3$, RT) δ 8.40 (s, 1H, ArC*H*N), 8.16 (t, *J* = 8.6 Hz, 1H, *H Ar*), 7.17-7.08 (m, 3H, *H Ar*), 6.84 (dd, 1H, *J* = 2.4, 8.8 Hz, *H Ar*), 6.67 (dd, 1H, *J* = 2.4, 12.3 Hz, *H Ar*), 3.88 (s, 3H, *Me*O), 2.97 (sp, *J* = 6.9 Hz, 2H, $(Me)_2CH$), 1.18 (d, *J* = 6.9 Hz, 12H, $(Me)_2CH$). **$^{13}C\{^1H\}$ NMR** (100 MHz, $CDCl_3$, RT) δ 164.1 (J_{CF} = 253.3 Hz), 163.8 (d, J_{CF} = 11.3 Hz), 155.2 (d, J_{CF} = 3.6 Hz), 149.7, 137.9, 128.8 (d, J_{CF} = 4.3 Hz), 124.2, 123.2, 116.9 (d, J_{CF} = 9.9 Hz), 111.3 (d, J_{CF} = 2.1 Hz), 101.4 (d, J_{CF} = 24.7 Hz), 56.0, 28.1, 23.6. **HRMS** (ESI) *m/z* calcd for $C_{20}H_{25}NFO$ $[M+H]^+$ 314.1915. Found: 314.1903. These data are in agreement with our previously reported data.[9]

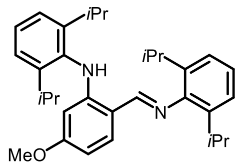

AA-2d

(*E*)-*N*-(2,6-diisopropylphenyl)-2-(((2,6-diisopropylphenyl)imino)methyl)-5-methoxyaniline (AA-2d) : (*E*)-*N*-(2-fluoro-4-methoxybenzylidene)-2,6-diisopropylaniline (**I-2d**) (3.3 g, 10.5 mmol) was added to lithium (2,6-diisopropylphenyl)amide (31.5 mmol) in suspension in toluene (80 mL) at room temperature. The solution was heated at 90 °C and stirred for 72 h. Then, the solution was cooled at room temperature and a saturated aqueous solution of Na_2CO_3 (40 mL) was added. The aqueous layer was separated and extracted with Et_2O (3 x 20 mL). Organics layers were combined, dried over anhydrous $MgSO_4$, filtered and the solvent was removed under reduced pressure to give a light brown oil. The brown oil obtained was recrystallized in ethanol at room temperature to afford **AA-2d** as pale-yellow crystalline solid (3.6 g, 7.7 mmol, 73%). **^1H NMR** (400 MHz, $CDCl_3$, RT) δ 10.73 (s, 1H, N*H*), 8.28 (s, 1H, ArC*H*N), 7.34-7.10 (m, 7H, *H Ar*), 6.30 (dd, *J* = 2.4, 8.5 Hz, 1H, *H Ar*), 5.77 (d, 1H, *J* = 2.4 Hz, *H Ar*), 3.66 (s, 3H, *Me*O), 3.24 (sp, 2H, *J* = 6.9 Hz, $(Me)_2CH$), 3.14 (sp, 2H, *J* = 6.9 Hz, $(Me)_2CH$), 1.19 (d, 12H overlap with 6H, *J* = 6.9 Hz, $(Me)_2CH$), 1.15 (d, 6H, *J* = 6.9 Hz, $(Me)_2CH$). **$^{13}C\{^1H\}$ NMR** (100 MHz, $CDCl_3$, RT) δ 164.8, 163.4, 151.9, 149.1, 147.5, 138.5, 136.4, 134.4, 127.7, 124.2, 123.9, 123.1, 110.9, 102.7, 95.9, 55.3, 28.7, 28.2, 24.7, 23.7, 23.3. **HRMS** (ESI) *m/z* calcd for $C_{32}H_{43}N_2O$ $[M+H]^+$ 471.3370. Found: 471.3364. These data are in agreement with our previously reported data.[9]

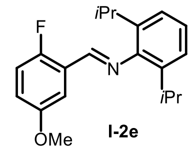

I-2e

(*E*)-*N*-(2-fluoro-5-methoxybenzylidene)-2,6-diisopropylaniline (I-2e): In a Dean-Stark apparatus, *p*-toluene sulphonic acid (0.032 g, 0.168 mmol) was added to a stirred solution of 2-fluoro-5-methoxybenzaldehyde (2.5 g, 16.2 mmol) and 2,6-diisopropylaniline (3.4 mL, 17.8 mmol) in toluene (40 mL). The solution was heated at 150 °C overnight. Then, the solution was cooled at room temperature and a saturated aqueous solution of Na_2CO_3 (20 mL) was added. The aqueous layer was separated and extracted with Et_2O (3 x 20 mL). Organics layers were combined, dried with $MgSO_4$, filtered and the solvent was removed under reduced pressure to obtain a slightly yellow oil. This oil was dissolved in hexane and cooled to –20 °C to afford **I-2e** as yellow crystalline solid (3.2 g, 10.2 mmol, 63%). **^1H NMR** (400 MHz, $CDCl_3$, RT) δ 8.48 (s, ArC*H*N, 1 H), 7.71 (dd, *J* = 3.0 Hz, *J* = 5.4 Hz, 1H, *H Ar*), 7.18-7.01 (m, 5H, *H Ar*), 3.89 (s, 3H, *Me*O), 2.96 (sp, 2H, *J* = 6.9 Hz, $(Me)_2CH$), 1.19 (d, 12H, *J* = 6.9 Hz, $(Me)_2CH$).**$^{13}C\{^1H\}$ NMR** (100 MHz, $CDCl_3$, RT) δ 157.7(d, J_{CF} = 246.5 Hz), 156.2, 155.7 (d, J_{CF} = 4.1 Hz), 149.3, 137.7, 124.5, 124.0 (d, J_{CF} = 10.6 Hz), 123.2, 120.1 (d, J_{CF} = 8.3 Hz), 117.0 (d, J_{CF} = 23.1 Hz), 110.1, 56.1, 28.1, 23.6. **HRMS** (ESI) m/z calcd for

$C_{20}H_{25}NFO$ [M+H]+ 314.1915. Found: 314.1905. These data are in agreement with our previously reported data.[9]

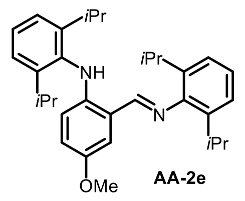
AA-2e

(*E*)-*N*-(2,6-diisopropylphenyl)-2-(((2,6-diisopropylphenyl)imino)methyl)-4-methoxyaniline (AA-2e) : (*E*)-*N*-(2-fluoro-5-methoxybenzylidene)-2,6-diisopropylaniline (**I-2e**) (3.0 g, 9.6 mmol) was added to lithium (2,6-diisopropylphenyl)amide (28.7 mmol) in suspension in toluene (70 mL) at room temperature. The solution was heated at 90 °C and stirred for 72 h. Then, the solution was cooled at room temperature and a saturated aqueous solution of Na_2CO_3 (35 mL) was added. The aqueous layer was separated and extracted with Et_2O (3 x 20 mL). Organics layers were combined, dried over anhydrous $MgSO_4$, filtered and the solvent was removed under reduced pressure to obtain a slightly brown oil. The brown oil obtained was recrystallized in ethanol at room temperature to afford **AA-2e** as pale yellow crystalline solid (2.7 g, 5.8 mmol, 61%). **^{1}H NMR** (400 MHz, $CDCl_3$, RT) δ 10.19 (s, 1H, N*H*), 8.35 (s, 1H, ArC*H*N), 7.34-7.12 (m, 6H, *H Ar*), 6.91 (d, 1H, *J* = 2.9 Hz, *H Ar*), 6.85 (dd, 1H, *J* = 2.9 Hz, *J* = 9.0 Hz, *H Ar*), 6.26 (d, 1H, *J* = 9.0 Hz, *H Ar*), 3.80 (s, 3H, *Me*O), 3.23 (sp, 2H, *J* = 6.9 Hz, $(Me)_2CH$), 3.12 (sp, 2H, *J* = 6.9 Hz, $(Me)_2CH$), 1.2 (d, 12H, *J* = 6.9 Hz, $(Me)_2CH$), 1.16 (d, 6H, *J* = 6.9 Hz, $(Me)_2CH$), 1.15 (d, 6H, *J* = 6.9 Hz, $(Me)_2CH$). **^{13}C{^{1}H} NMR** (100 MHz, $CDCl_3$, RT) δ 165.4, 150.1, 148.9, 147.8, 145.0, 138.2, 135.1, 127.4, 124.5, 123.9, 123.2, 120.2, 117.9, 116.0, 113.3, 56.1, 28.7, 28.2, 24.7, 23.7, 23.2. **HRMS** (ESI) *m/z* calcd for $C_{32}H_{43}N_2O$ [M+H]+ 471.3370. Found: 471.3360. These data are in agreement with our previously reported data.[9]

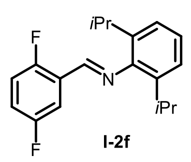
I-2f

(*E*)-1-(2,5-difluorophenyl)-*N*-(2,6-diisopropylphenyl)methanimine (I-2f): 2,6-diisopropylaniline (8.4 mL, 45 mmol) was added to a stirred solution of 2,5-difluorobenzaldehyde (6.39 g, 45 mmol) and $MgSO_4$ (1.60 g) in hexane (40 mL) at room temperature. The solution was stirred for 16 h. Then, the solution was filtered, concentrated under reduced and cooled to –20 °C to afford **I-2f** as yellow crystalline solid (5.8 g, 19.2 mmol, 43%). **^{1}H NMR** (400 MHz, $CDCl_3$, RT) δ 8.47 (d, 1H, *J* = 2.5 Hz, ArC*H*N), 7.95-7.89 (m, 1H, *H* Ar), 7.20-7.09 (m, 5H, *H* Ar), 2.93 (sp, 2H, *J* = 6.9 Hz, $(CH_3)_2CH$), 1.19 (d, 12H, *J* = 6.9 Hz, $(CH_3)_2CH$). **^{13}C{^{1}H} NMR** (100 MHz, $CDCl_3$, RT) δ 159.1 (dd, J_{CF} = 15.6, 243.7 Hz), 154.8, 149.0, 137.6, 124.7, 123.2, 119.8 (dd, J_{CF} = 8.8, 25.0 Hz), 117.5 (dd, J_{CF} = 8.2, 24.0 Hz), 113.6 (dd, J_{CF} = 2.4, 25.0 Hz), 28.1, 23.6. **HRMS** (ESI) m/z calcd for $C_{19}H_{22}NF_2$ [M+H]+ 302.1715. Found: 302.1707.

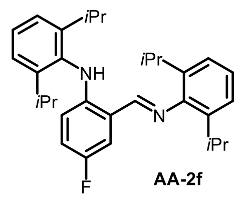
AA-2f

(*E*)-*N*-(2,6-diisopropylphenyl)-2-(((2,6-diisopropylphenyl)imino)methyl)-4-fluoroaniline (AA-2f) : *n*-BuLi (7.1 mL, 11.4 mmol) was added to a stirred solution of 2,6-diisoproopylaniline (1.8 mL, 10.4 mmol) in THF (10 mL) at -78 °C. The solution was stirred 2 h at rt and then, was cannulated to a stirred solution of (*E*)-1-(2,5-difluorophenyl)-*N*-(2,6-diisopropylphenyl)methanimine **I-2f** (2.85 g, 9.5 mmol) in THF (10 mL) at room temperature. The solution was stirred overnight. Then, water (10 mL) was added. The aqueous layer was separated and extracted with Et_2O (3 x 10 mL). Organics layers

were reunified, dried over $MgSO_4$, filtered and the solvent was removed under reduced pressure to obtain a brown oil. A recrystallization in ethanol at room temperature affords **AA-2f** as a pale-yellow crystalline solid (1.95 g, 4.25 mmol, 45%). **^{1}H NMR** (400 MHz, $CDCl_3$, RT) δ 10.32 (br s, 1H, N*H*), 8.30 (s, 1H, ArC*H*N), 7.34-7.11 (m, 6H, *H Ar*), 7.06 (dd, 1H, *J* = 2.9, 8.9 Hz, *H Ar*), 6.91 (dt, 1H, *J* = 2.9, 8.6 Hz, *H Ar*), 6.22 (dd, 1H, *J* = 4.5, 9.2 Hz, *H Ar*) 3.19 (sp, 2H, *J* = 7.0 Hz, $(CH_3)_2CH$), 3.10 (sp, 2H, *J* = 7 Hz, $(CH_3)_2CH$), 1.19 (d, 12H, *J* = 7 Hz, $(CH_3)_2CH$), 1.15 (d, 6H, *J* = 7 Hz, $(CH_3)_2CH$), 1.14 (d, 6H, *J* = 7 Hz, $(CH_3)_2CH$). **^{13}C{^{1}H} NMR** (100 MHz, $CDCl_3$, RT) δ 164.8, 153.9 (d, J_{CF} = 231.5 Hz), 148.6, 147.7, 146.8, 138.2, 134.7, 127.8, 124.7, 124.0, 123.3, 119.7 (d, J_{CF} = 22.7 Hz), 119.3 (d, J_{CF} = 22.1 Hz), 115.9 (d, J_{CF} = 6.1 Hz), 113.1 (d, J_{CF} = 6.6 Hz), 28.7, 28.3, 24.7, 23.7, 23.2. **HRMS** (ESI) *m/z* calcd for $C_{31}H_{40}N_2F$ $[M+H]^+$ 459.3170. Found: 459.3148.

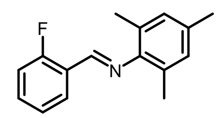

I-2g

(*E*)-*N*-(2-fluorobenzylidene)-2,4,6-trimethylaniline (I-2g): 2,4,6-trimethylaniline (10.0 mL, 95.0 mmol) was added to a stirred solution of 2-fluorobenzaldehyde (13.2 mL, 95.0 mmol) and $MgSO_4$ (2.0 g) in hexane (60 mL) at room temperature. The solution was stirred for 16 h. Then, the solution was filtered, concentrated under reduced and cooled to −20 °C to afford **I-2g** as yellow crystalline solid (15.5 g, 64 mmol, 68%). **^{1}H NMR** (250 MHz, $CDCl_3$, RT) δ 8.54 (s, 1H, ArC*H*N), 8.29 (td, 1H, *J* = 7.6, 1.8 Hz, *H* Ar), 7.51-7.42 (m, 1H, *H* Ar), 7.26 (t, 1H, *J* = 7.6 Hz, *H* Ar), 7.13 (ddd, 1H, *J* = 10.4, 8.4, 1.1 Hz, *H* Ar), 6.89 (s, 2H, *H* Ar), 2.29 (s, 3H, *p-Me*), 2.13 (s, 6H, *o-Me*). **HRMS** (ESI) *m/z* calcd for $C_{16}H_{17}NF$ $[M+H]^+$ 242.1340. Found: 242.1334. These data are in agreement with our previously reported data.[9]

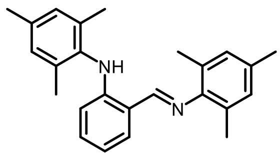

AA-2g

(*E*)-*N*-(2-(mesitylamino)benzylidene)-2,4,6-trimethylaniline (**AA-2g**): *n*-BuLi (12 mL, 30.0 mmol) was added to a stirred solution of 2,4,6-trimethylaniline (4.2 mL, 30.0 mmol) in THF (40 mL) at −78 °C. The solution was stirred for 2 h and then, was cannulated to a stirred solution of (*E*)-*N*-(2-fluorobenzylidene)-2,4,6-trimethylaniline (**I-2g**) (4.8 g, 20 mmol) in THF (20 mL) at room temperature. The solution was stirred for 16 h. Then, water (20 mL) was added. The aqueous layer was separated and extracted with hexane (3 x 30 mL). Organics layers were combined, dried over anhydrous $MgSO_4$, filtered and concentrated under reduced pressure to obtain a slight brown oil. The brown oil obtained was recrystallized in ethanol at −20 °C to afford **AA-2g** as pale yellow crystalline solid (3.9 g, 10.9 mmol, 55%). **^{1}H NMR** (250 MHz, $CDCl_3$, RT) δ 10.47 (s, 1H, N*H*), 8.35 (s, 1H, ArC*H*N), 7.32 (dd, 1H, *J*= 7.5, 1.6 Hz, *H Ar*), 7.15 (ddd, 1H, *J*= 8.4, 7.5, 1.6 Hz, *H Ar*), 6.96 (s, 2H, *m-H Ar*), 6.91 (s, 2H, *m-H Ar*), 6.69 (td, 1H, *J*= 7.5, 1.0 Hz, *H Ar*), 6.28 (d, 1H, *J*= 8.4 Hz, *H Ar*), 2.32 (s, 3H, *p-Me*), 2.29 (s, 3H, *p-Me*), 2.19 (s, 6H, *o-Me*), 2.16 (s, 6H, *o-Me*). **^{13}C{^{1}H} NMR** (100 MHz, $CDCl_3$, RT) δ 166.2, 148.8, 148.5, 136.6, 136.0, 134.9, 134.6, 133.2, 132.2, 129.2, 128.9, 127.7, 116.8, 115.2, 111.8, 21.1, 20.9, 18.6, 18.4. **HRMS** (ESI) *m/z* calcd for $C_{25}H_{29}N_2$ $[M+H]^+$ 357.2325. Found: 357.2318. These data are in agreement with our previously reported data.[9]

3-Synthesis of Anilido-Aldimine (AA) Fe(II) chloro-ate complexes

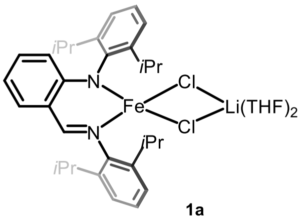

1a

Complex 1a: *n*-BuLi (1.4 mL, 3.5 mmol) was added to a stirred solution of **AA-2a** (1.5 g, 3.40 mmol) in THF (15 mL) at -78 °C. The cooling was removed 30 min after addition and the resulting light orange solution was stirred for 2 h at room temperature. Then, $FeCl_2$ was added (0.431 g, 3.4 mmol). The solution was stirred overnight and then, the solvent was removed under reduced pressure from the dark red solution. The dark red solid was washed with hexane (10 mL). Then the solid was extracted with Et_2O (15 ml). The dark red solution was concentrated and cooled to −20 °C to afford **1a** as red crystalline solid (1.91 g, 2.66 mmol, 78%). **Elemental analysis** calcd for $C_{39}H_{53}Cl_2FeLiN_2O_2$: C 65.46; H 7.47; N 3.91, found: C 64.91; H 7.37; N 3.95. **^{1}H NMR** (250 MHz, C_6D_6-50 µL THF-d_8, RT) δ 91.4 (s, 1H), 83.3 (s, 1H), 23.3 (s, 2H), 14.5 (s, 2H), 4.5 (br s, 8H, *THF*), 4.3 (br s, 6H), 1.8 (br s, 8H, *THF*), 1.4 (br s, 6H), −8.6 (s, 1H), −16.4 (br s, 6H), −19.1 (br s, 6H), −29.5 (br s, 2H), −34.5 (s, 1H), −39.5 (s, 1H), −42.1 (br s, 1H), −51.6 (br s, 2H), −56.5 (s, 1H, *p-H Ar*). **^{7}Li NMR** (117 MHz, C_6D_6-50 µL THF-d_8, RT) δ 269.8 (br s). These data are in agreement with our previously reported data.[9]

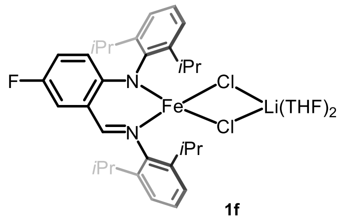

1f

Complex 1f: n-BuLi (1.2 mL, 1.91 mmol) was added to a stirred solution of **AA-2f** (0.9 g, 1.91 mmol) in THF (9 mL) at -78 °C. The cooling was removed 30 min after addition and the resulting light orange solution was stirred for 2 h at room temperature. Then, $FeCl_2$ was added (0.242 g, 1.91 mmol). The solution was stirred overnight and then, the solvent was removed under reduced pressure from the dark red solution. The dark red solid was washed with hexane (10 mL). Then the solid was extracted with Et_2O (30 ml). The dark red solution was concentrated and cooled to −20 °C to afford **1f** as dark red crystalline solid (1.24 g, 1.69 mmol, 88%). **Elemental analysis** calcd for $C_{39}H_{52}Cl_2FFeLiN_2O_2$: C 63.86; H 7.15; N 3.82, found: C 62.46; H 7.33; N 3.67. **^{1}H NMR** (300 MHz, C_6D_6-50 µL THF-d_8, RT) δ 91.4 (s, 1H), 84.9 (br s, 1H), 24.0 (s, 2H), 15.0 (s, 2H), 5.0 (s, 8H), 4.2 (br s, 6H), 1.9 (s, 8H), 1.2 (br s, 6H), -15.6 (s, 6H, CHMe*Me*), -18.0 (s, 6H, CH*Me*Me), -27.0 (br s, 1H, ArC*H*N), -33.8 (s, 1H, *p-H Ar*), -46.0 (br s, 1H, *p-H Ar*), -48.6 (br s, 1H), -55.2 (s, 1H). **^{7}Li NMR** (140 MHz, C_6D_6-50 µL THF-d_8, RT) δ 256.2 (br s).

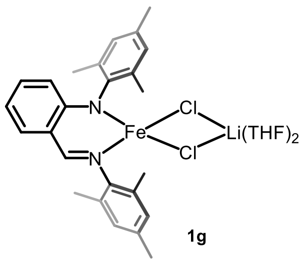

1g

Complex 1g: *n*-BuLi (1.2 mL, 3.0 mmol) was added to a stirred solution of **AA-2g** (1.0 g, 2.8 mmol) in THF (7 mL) at −78 °C. The cooling was removed 30 min after addition and the resulting light orange solution was stirred for 2 h at room temperature. Then, $FeCl_2$ was added (0.356 g, 2.8 mmol). The solution was stirred overnight and then, the solvent was removed under reduced pressure from the dark red solution. The dark red solid was washed with hexane (10 mL). Then the solid was extracted with Et_2O (20 ml). The dark red solution was concentrated and cooled to −20 °C to afford **1g** as red-orange crystalline

solid (1.12 g, 1.77 mmol, 63%). **Elemental analysis** calcd for $C_{33}H_{41}Cl_2FeLiN_2O_2$: C 62.78; H 6.55; N 4.44, found: C 62.54; H 6.68; N 4.47. **^{1}H NMR** (250MHz, C_6D_6-50 µL THF-d_8, RT) δ 95.8 (br s, 1H), 85.7 (br s, 1H), 58.4 (s, 3H), 34.6 (s, 3H), 24.5 (s, 2H), 15.2 (br s, 8H), 4.6 (s, 8H, *THF*), 1.8 (s, 8H, *THF*), 0.5 (br s, 6H), –39.9 (s, 1H), –44.8 (br s, 1H). **^{7}Li NMR** (117 MHz, C_6D_6-50µL THF-d_8, RT) δ 238.2 (br s). These data are in agreement with our previously reported data.[9]

4-Synthesis of Anilido-Aldimine (AA) Fe(II) alkyl and Hydride complexes

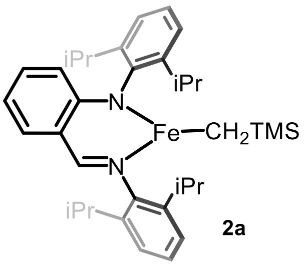

2a

Complex 2a: $LiCH_2TMS$ (236 mg, 2.5 mmol) was added as a solid to a Schlenk tube containing a stirred solution of **1a** (1.8 g, 2.5 mmol) in Et_2O (12.5 mL), at room temperature. Immediately after addition, a white precipitate appeared. The mixture was stirred overnight, then Et_2O was removed under reduced pressure until dryness. The solid was extracted with hexane. The extract was filtered, concentrated under reduced pressure then cooled to -20°C to afford complex **2a** as a red-orange crystalline solid (1.28 g, 2.2 mmol, 88%). **Elemental analysis** calcd for $C_{35}H_{50}FeN_2Si$: C 72.14; H 8.65; N 4.81, found: C 71.39; H 7.78; N 4.86. **Evans μ_{eff}** (THF-d_8, 300 K) 5.2 μ_B. **^{1}H NMR** (400 MHz, C_6D_6, RT) δ 109.5 (br s, 1H, *m-H* Ar), 104.1 (br s, 1H, *m-H* Ar), 51.5 (br s, 9H, Si(*Me*)$_3$), 44.5 (br s, 1H, ArC*H*N), 14.9 (s, 1H, *p-H* Ar), 2.2 (s, 6H, (*Me*)$_2$CH), −6.3 (s, 2H, *m-H* Ar), −10.4 (s, 2H, *m-H* Ar), −19.4 (s, 6H, (*Me*)$_2$CH), −49.9 (s, 1H, *p-H* Ar), −63.3 (br s, 6H, (*Me*)$_2$CH), −84.7 (s, 1H, *p-H* Ar), −109.4 (br s, 6H, (*Me*)$_2$CH).

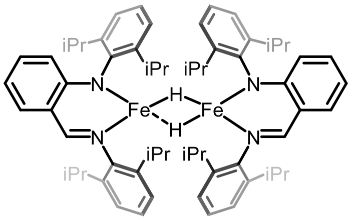

[2b]$_2$

Complex [2b]$_2$: Dicyclohexylamine borane (322 mg, 1.6 mmol) was added as a solid to a Schlenk tube containing a stirred solution of **2a** (874 mg, 1.5 mmol) in benzene (6 mL), at room temperature. The mixture was allowed to warm up to reflux and stirred for 24 h under an argon atmosphere. Then, the solution was concentrated under reduced pressure until dryness. The solid was dissolved in hexane, then cooled to -20°C to afford the complex as a brown crystalline solid (450 mg, 0.45 mmol, 60%). This latter was washed twice with cold hexane to remove traces of borane side products. **Elemental analysis** calcd for $C_{62}H_{80}Fe_2N_4$: C 74.99; H 8.12; N 5.64, found: C 72.23; H 7.81; N 5.34. **Evans μ_{eff}** (Toluene-d_8, 300 K) 6.2 μ_B. **^{1}H NMR** (400 MHz, C_6D_6, 60 °C) δ 42.3 (s, 2H), 40.6 (s, 2H), 15.6 (s, 4H), 12.9 (br s, 4H), 9.2 (s, 4H), 8.2 (s, 12H), 2.9 (s, 12H), −3.1 (s, 2H), −12.4 (s, 2H), −12.9 (s, 12H), −17.9 (s, 2H), −21.7 (s, 12H overlap with 4H), -28.6 (br s, 2 H).

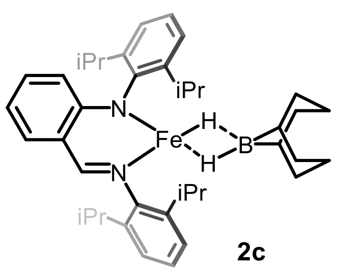

2c

Complex 2c: 9-BBN dimer (244 mg, 1 mmol) was added as a solid to a Schlenk tube containing a stirred solution of **2a** (583 mg, 1 mmol) in benzene (6 mL), at room temperature. The mixture was allowed to warm up to reflux and stirred for 16h under an argon atmosphere. Then, the solution was concentrated under reduced pressure until dryness. The solid was dissolved in hexane, then cooled to -20°C to afford the complex as a yellow-brown crystalline solid (325 mg, 0.52 mmol, 52%). This latter was washed twice with cold hexane to remove traces of borane side products. **Elemental analysis** calcd for $C_{39}H_{55}BFeN_2$: C 75.73; H 8.96; N 4.53, found: C 75.01; H 8.81; N 4.39. **Evans** $\boldsymbol{\mu_{eff}}$ (Toluene-d_8, 300 K) 5.4 μ_B. **^{1}H NMR** (400 MHz, C_6D_6, RT) δ 169.1 (s, 4H), 70.3 (br s, 2H), 57.1 (s, 1H), 44.9 (s, 2H), 43.3 (s, 1H), 42.2 (s, 4H), 33.2 (s, 2H), 22.7 (s, 2H), 13.5 (s, 2H), 4.7 (s, 6H), 0.5 (br s, 1H), −5.2 (s, 6H), −12.0 (br s, 2H), -22.0 (s, 1H), -23.7 (s, 6H), -32.8 (s, 1H), -43.5 (s, 6H), -66.7 (s, 1H), -73.1 (br s, 2 H).

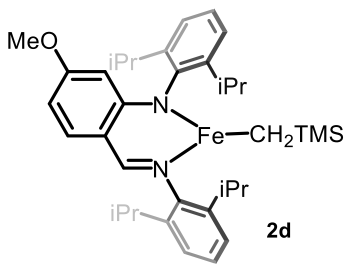

2d

Complex 2d: *n*-BuLi (1.6 mL, 5 mmol, 2.5 M in hexane) was added to a stirred solution of **AA-2d** (1.90 g, 4.0 mmol) in THF (8 mL) at −78 °C. The cooling bath was removed 30 min after the addition and the solution was stirred for 4 h at 25 °C. Then, $FeCl_2$ (0.51 g, 4.0 mmol) was added. The solution was stirred overnight and the solvent was evaporated under reduced pressure. The residual solid was washed with hexane or pentane (10 mL) then extracted with Et_2O (4 x 5 mL). After filtration of ethereal solution, $LiCH_2TMS$ (380 mg, 4.0 mmol) was added as a solid at room temperature. Immediately after addition, a white precipitate appeared. The mixture was stirred overnight, then Et_2O was removed under reduced pressure until dryness. The solid was extracted with hexane. The extract was filtered, concentrated under reduced pressure then cooled to -20°C to afford complex **2d** as an orange crystalline solid (1.0 g, 1.6 mmol, 41%). **Elemental analysis** calcd for $C_{36}H_{52}FeN_2OSi$: C 70.57; H 8.55; N 4.57, found: C 69.86; H 7.78; N 4.65. **Evans** $\boldsymbol{\mu_{eff}}$ (THF-d_8, 300 K) 5.2 μ_B. **^{1}H NMR** (400 MHz, C_6D_6, RT) 99.1 (br s, 1H, *m-H Ar*), 49.8 (br s, 9H, Si(*Me*)$_3$), 43.7 (br s, 1H, ArC*H*N), 26.7 (s, 3 H, *Me*O), 19.6 (s, 1H, *p-H Ar*), 0.3 (s, 6H, (*Me*)$_2$CH), -5.5 (s, 2H, *m-H Ar*), −9.5 (s, 2H, *m-H Ar*), −17.3 (s, 6H, (*Me*)$_2$CH), −48.1 (s, 1H, *p-H Ar*), −61.9 (br s, 6H, (*Me*)$_2$CH), −80.8 (s, 1H, *p-H Ar*), −105.4 (br s, 6H, (*Me*)$_2$CH).

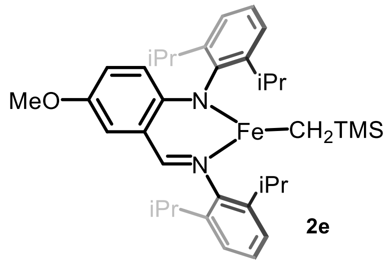

2e

Complex 2e: n *n*-BuLi (1.6 mL, 5 mmol, 2.5 M in hexane) was added to a stirred solution of **AA-2e** (1.90 g, 4.0 mmol) in THF (8 mL) at −78 °C. The cooling bath was removed 30 min after the addition and the solution was stirred for 4 h at 25 °C. Then, $FeCl_2$ (0.51 g, 4.0 mmol) was added. The solution was stirred overnight and the solvent was evaporated under reduced pressure. The residual solid was washed with

hexane (10 mL) then extracted with Et_2O (4 x 5 mL). After filtration of ethereal solution, $LiCH_2TMS$ (380 mg, 4.0 mmol) was added as a solid at room temperature. Immediately after addition, a white precipitate appeared. The mixture was stirred overnight, then Et_2O was removed under reduced pressure until dryness. The solid was extracted with hexane. The extract was filtered, concentrated under reduced pressure then cooled to -20°C to afford complex **2d** as an orange crystalline solid (1.2 g, 2.0 mmol, 50%). **Elemental analysis** calcd for $C_{36}H_{52}FeN_2OSi$: C 70.57; H 8.55; N 4.57, found: C 70.55; H 8.48; N 4.83. **Evans μ_{eff}** (THF-d_8, 300 K) 5.4 μ_B. **^{1}H NMR** (400 MHz, C_6D_6, RT) δ 107.6 (br s, 2H, *m-H Ar*), 51.2 (br s, 9H, Si(*Me*)$_3$), 43.5 (br s, 1H, ArC*H*N), 35.6 (s, 3H, *Me*O), 2.7 (s, 6H, (*Me*)$_2$CH), −4.7 (s, 2H, *m-H Ar*), −9.6 (s, 2H, *m-H Ar*), −19.4 (s, 6H, (*Me*)$_2$CH), −50.9 (s, 1H, *p-H Ar*), −64.7 (br s, 6H, (*Me*)$_2$CH), −85.1 (s, 1H, *p-H Ar*), −107.1 (br s, 6H, (*Me*)$_2$CH).

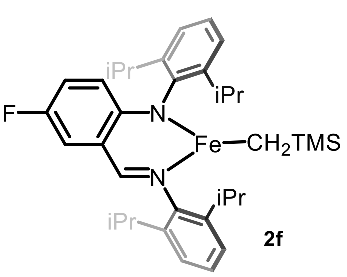

2f

Complex 2f: $LiCH_2TMS$ (113 mg, 1.2 mmol) was added as a solid to a Schlenk tube containing a stirred solution of **1f** (0.9 g, 1.2 mmol) in Et_2O (6 mL) at room temperature. Immediately after addition, a white precipitate appeared. The mixture was stirred overnight, then Et_2O was removed under reduced pressure until dryness. The solid was extracted with hexane. The extract was filtered, concentrated under reduced pressure then cooled to -20°C to afford complex **2f** as a red crystalline solid (380 mg, 0.63 mmol, 53%). **Elemental analysis** calcd for $C_{35}H_{49}FFeN_2Si$: C 69.98; H 8.22; N 4.66, found: C 67.67; H 8.13; N 4.48. **Evans μ_{eff}** (THF-d_8, 300 K) 5.2 μ_B. **^{1}H NMR** (400 MHz, C_6D_6, RT) δ 112.7 (br s, 1H, *m-H Ar*), 107.6 (br s, 1H, *m-H Ar*), 52.0 (br s, 9H, Si(*Me*)$_3$), 39.6 (br s, 1H, ArC*H*N), 2.6 (s, 6H, (*Me*)$_2$CH), −5.8 (s, 2H, *m-H Ar*), −10.1 (s, 2H, *m-H Ar*), −19.1 (s, 6H, (*Me*)$_2$CH), −50.9 (s, 1H, *p-H Ar*), −63.6 (br s, 6H, (*Me*)$_2$CH), −85.3 (s, 1H, *p-H Ar*), −107.0 (br s, 6H, (*Me*)$_2$CH).

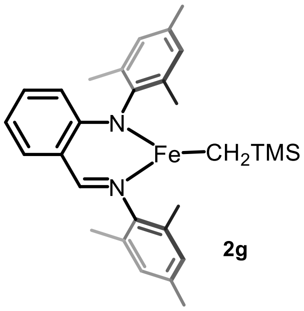

2g

Complex 2g: $LiCH_2TMS$ (443 mg, 4.7 mmol) was added as a solid to a Schlenk tube containing a stirred solution of **1g** (3 g, 4.7 mmol) in Et_2O (24 mL) at room temperature. Immediately after addition, a white precipitate appeared. The mixture was stirred overnight, then Et_2O was removed under reduced pressure until dryness. The solid was extracted with hexane. The extract was filtered, concentrated under reduced pressure then cooled to -20°C to afford complex **2g** as a brown-red crystalline solid (1.5 g, 3.0 mmol, 64%). **Elemental analysis** calcd for $C_{29}H_{38}FeN_2Si$: C 69.86; H 7.68; N 5.62, found: C 69.45; H 7.66; N 5.69. **Evans μ_{eff}** (Toluene-d_8, 300 K) 5.9 μ_B. **^{1}H NMR** (400 MHz, C_6D_6, RT) δ 105.1 (br s, 1H, *m-H Ar*), 99.3 (br s, 1H, *m-H Ar*), 49.5 (s, 3H, *p-Me*), 45.5 (br s, 1H, ArC*H*N), 35.8 (br s, 9H, Si(*Me*)$_3$), 28.4 (s, 3H, *p-Me*), 16.7 (s, 1H, *p-H Ar*), -5.0 (s, 2H, *m-H Ar*), -8.5 (s, 2H, *m-H Ar*), -45.0 (br s, 6H, *o-Me*), -83.4 (br s, 6H, *o-Me*).

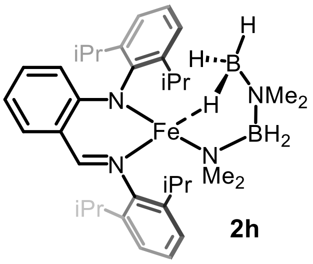

2h

Complex 2h: In an argon-filled glovebox, **2a** (146 mg, 0. 25 mmol) was dissolved in benzene and added to an oven-dried 10 mL screw-capped tube equipped with a magnetic stir bar containing $Me_2NH.BH_3$ (29.5 mg, 0.5 mmol). The mixture was allowed to warm up to 60 °C and stirred for 5 h under an argon atmosphere. Then, the solution was concentrated under reduced pressure until dryness. The solid was dissolved in pentane:Et_2O (1:1), then cooled to -20°C to afford the complex **2h** as a red orange crystalline solid (64 mg, 0.1 mmol, 40%). **Evans μ_{eff}** (Toluene-d_8, 300 K) 5.9 μ_B. **^{1}H NMR** (300 MHz, C_6D_6, RT) δ 170.5 (br s, 6H), 79.6 (s, 1H), 69.7 (s, 1H), 60.3 (br s, 3H), 45.2 (br s, 3H), 20.0 (s, 6H), 11.8 (s, 6H), -8.2 (br s, 4H), -13.3 (s, 6H), -16.2 (s, 6H), -24.4 (s, 1H), -27.0 (br s, 1H), -37.4 (br s, 1H), -38.4 (s, 1 H), -40.5 (s, 1H), -76.0 (br s, 1H).

5-Optimization studies and control experiments

5a-Optimization studies

In an argon-filled glovebox, **2a** (14.6 mg, 0.025 mmol) was dissolved in deuterated benzene and added to a 10 mL screw-capped tube, equipped with a magnetic stir bar, containing $Cy_2NH.BH_3$ (4.9 mg, 0.025 mmol) unless otherwise stated. Next, allylbenzene **3a** (59.1 mg, 0.5 mmol) was added, and the reaction was stirred at 60 °C for 48 h (unless otherwise stated). Upon completion, the reaction mixture was exposed to air, and 1,3,5-trimethoxybenzene (42 mg, 0.25 mmol, 0.5 equiv.) was added to the reaction vessel. Spectroscopic yield and stereoselectivity given by *E*:*Z* were determined by ^1H NMR analysis using 1,3,5-trimethoxybenzene as internal standard.

Table S1. Reaction optimization for Fe-catalyzed isomerization of **3a**

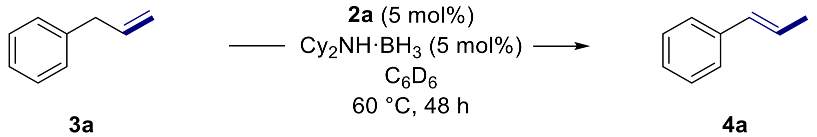

Entry	Variations from optimized conditions[a]	Conv. [%][b]	Yield [%][b]	*E*:*Z*[b]
1[c]	10 mol% *i*$Pr_2NH·BH_3$ instead of $Cy_2NH·BH_3$	97	86	8:1
2[c]	10 mol% $Me_2NH·BH_3$ instead of $Cy_2NH·BH_3$	100	81	24:1
3[c]	10 mol% $tBuNH_2·BH_3$ instead of $Cy_2NH·BH_3$	80	66	6:1
4[c]	10 mol% $NH_3·BH_3$ instead of $Cy_2NH·BH_3$	>99	82	25:1
5[c]	10 mol% morpholine·BH_3 instead of $Cy_2NH·BH_3$	>99	85	21:1
6[c]	10 mol% $Me_3N·BH_3$ instead of $Cy_2NH·BH_3$	0	-	-
7[c]	10 mol% $Ph_3P·BH_3$ instead of $Cy_2NH·BH_3$	0	-	-
8[c]	10 mol% HBpin instead of $Cy_2NH·BH_3$	88	82	7:1
9[d]	10 mol% H-9-BBN instead of $Cy_2NH·BH_3$	0	-	-
10	Toluene-*d₈* instead of C_6D_6	>99	98	15:1
11	MTBE or TAME instead of C_6D_6	>99	98	11:1
12	hexane instead of C_6D_6	>99	98	25:1
13	heptane instead of C_6D_6	>99	95	23:1
14	Without **2a**	0	-	-
15	Without $Cy_2NH·BH_3$	0	-	-
16	Without **2a** and $Cy_2NH·BH_3$	0	-	-
17[d]	25 °C instead of 60 °C	13	13	7:1
18	**1a** instead of **2a**	0	-	-
19	$FeCl_2$ instead of **2a**	0	-	-
20[d]	Addition of PPh_3 (0.0125 mmol)	70	67	7:1

[a] reactions run on a 0.5 mmol scale at 60 °C for 48 h with **3a** (1.0 equiv), **2a** (5 mol%) and $Cy_2NH·BH_3$ (5 mol%) unless otherwise stated [b] determined by ^1H NMR using 1,3,5-trimethoxybenzene as internal standard unless otherwise stated [c] 16 h instead of 48 h [d] 24 h instead of 48 h.

5b-Control experiments

Geometrical isomerization of (*Z*)-1,2-diphenylethene (*Z*)-3b (Table S2): In an argon-filled glovebox, **2a** (14.6 mg, 0.025 mmol) was dissolved in deuterated benzene and added to a 10 mL screw-capped tube, equipped with a magnetic stir bar, containing $Cy_2NH.BH_3$ (4.9 mg, 0.025 mmol) unless otherwise stated. Next, (*Z*)-1,2-diphenylethene (*Z*)-**3b** (90.1 mg, 0.5 mmol) was added, and the reaction was stirred at 60 °C for 24 h (unless otherwise stated). Upon completion, the reaction mixture was exposed to air, and 1,3,5-trimethoxybenzene (42 mg, 0.25 mmol, 0.5 equiv.) was added to the reaction vessel. Conversion, spectroscopic yield and stereoselectivity given by *E*:*Z* were determined by ^{1}H NMR analysis using 1,3,5-trimethoxybenzene as internal standard.

Table S2. Control experiment for Fe-catalyzed geometrical isomerization of (*Z*)-**3b**

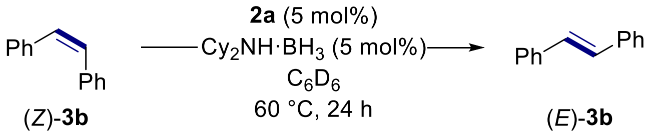

Entry	Variations from optimized conditions[a]	Conv. [%][b]	Yield [%][b]
1	none	>99	>99
2	Without **2a** and $Cy_2NH{\cdot}BH_3$	0	0
3	$FeCl_2$ instead of **2a**	0	0
4	Addition of 2.5 mol% of PPh_3	>99	>99

[a] reactions run on a 0.5 mmol scale at 60 °C for 24 h with (*Z*)-**3b** (1.0 equiv), **2a** (5 mol%) and $Cy_2NH{\cdot}BH_3$ (5 mol%) unless otherwise stated [b] determined by ^{1}H NMR using 1,3,5-trimethoxybenzene as internal standard.

Geometrical isomerization of (*E*)-prop-1-en-1-ylbenzene (*E*)-4a (Table S3): In an argon-filled glovebox, **2a** (14.6 mg, 0.025 mmol) was dissolved in deuterated benzene and added to a 10 mL screw-capped tube, equipped with a magnetic stir bar, containing $Cy_2NH.BH_3$ (4.9 mg, 0.025 mmol) unless otherwise stated. Next, (*E*)-prop-1-en-1-ylbenzene (*E*)-**4a** (90.1 mg, 0.5 mmol) was added, and the reaction was stirred at 60 °C for 24 h (unless otherwise stated). Upon completion, the reaction mixture was exposed to air, and 1,3,5-trimethoxybenzene (42 mg, 0.25 mmol, 0.5 equiv.) was added to the reaction vessel. Spectroscopic yield and stereoselectivity given by *E*:*Z* were determined by ^{1}H NMR analysis using 1,3,5-trimethoxybenzene as internal standard.

Table S3. Control experiments for Fe-catalyzed geometrical isomerization of (*E*)-**4a**

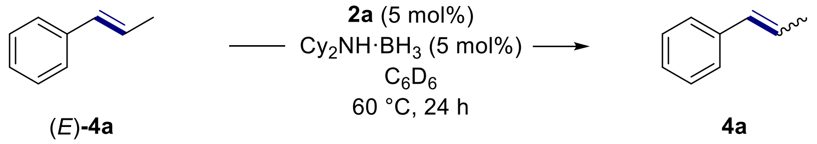

Entry	Variations from optimized conditions[a]	Yield [%][b]	*E*/*Z*[b]
1	none	>99	29/1
2	36 h instead of 24 h	>99	28/1
3	Without **2a** and $Cy_2NH{\cdot}BH_3$	0	-
4	Without $Cy_2NH{\cdot}BH_3$	0	-

[a] reactions run on a 0.5 mmol scale at 60 °C for 24 h with (*E*)-**4a** (1.0 equiv), **2a** (5 mol%) and $Cy_2NH{\cdot}BH_3$ (5 mol%) unless otherwise stated [b] determined by ^{1}H NMR using 1,3,5-trimethoxybenzene as internal standard.

Blank experiment for geometrical isomerization (Scheme S2): In an argon-filled glovebox, a solution of 5-(prop-1-en-1-yl)benzo[d][1,3]dioxole **4m** (81.1 mg, 0.5 mmol, *E*:*Z* 7:1) in deuterated benzene was stirred at 60 °C for 24 h. The reaction mixture was then subjected to ^{1}H NMR spectroscopy analysis to determine the *E*:*Z* stereoselectivity outcome. No geometrical isomerization was observed indicating that the geometrical interconversion is not thermally promoted.

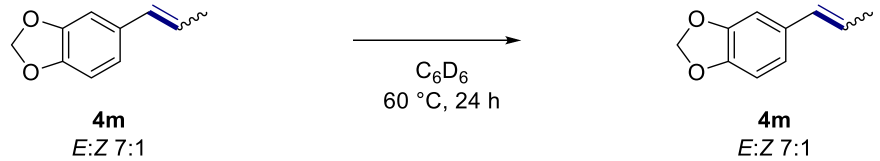

Scheme S2. Blank experiment for geometrical isomerization of 5-(prop-1-en-1-yl)benzo[d][1,3]dioxole **4m** under thermal conditions.

6-Mechanistic studies

6a-Kinetic and stereoselectivity profiles

General procedure to establish kinetic and stereoselectivity profiles of Fe-catalyzed isomerization of 3a: In an argon-filled glovebox, **2a** (14.6 mg, 0.025 mmol) was dissolved in deuterated benzene and added to a J-Young tap NMR tube containing $Cy_2NH.BH_3$ (4.9 mg, 0.025 mmol). Next, allylbenzene **3a** (59.1 mg, 0.5 mmol) was added, the reaction vessel was removed from the glovebox and monitored in a Bruker 400 MHz spectrometer for 48 h at 60 °C with measurements taken every 5 min. Kinetic data were analyzed using Microsoft Excel, and standard errors were calculated based on the sum of squared residuals (SSR) derived from the best-fit curve.

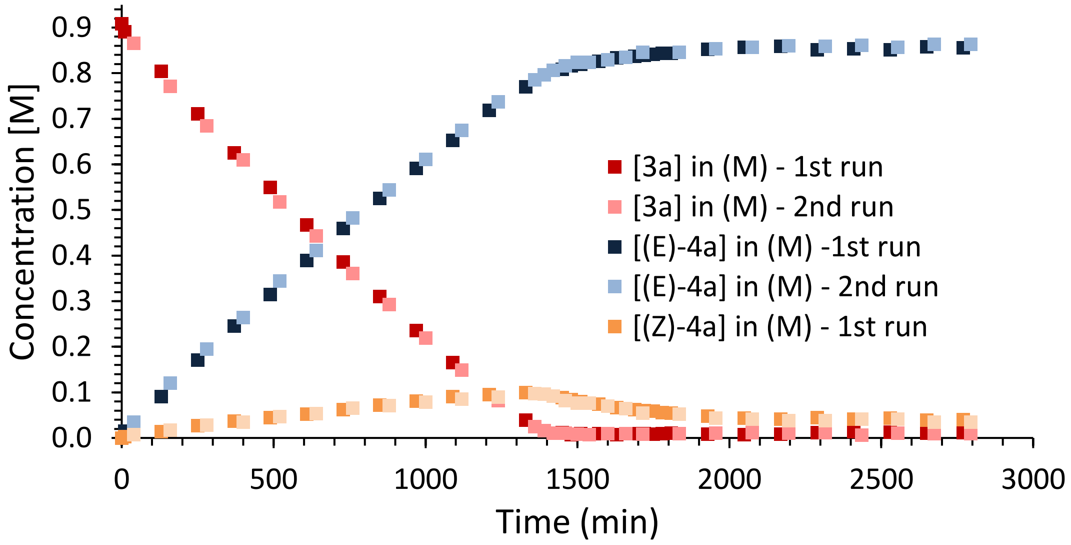

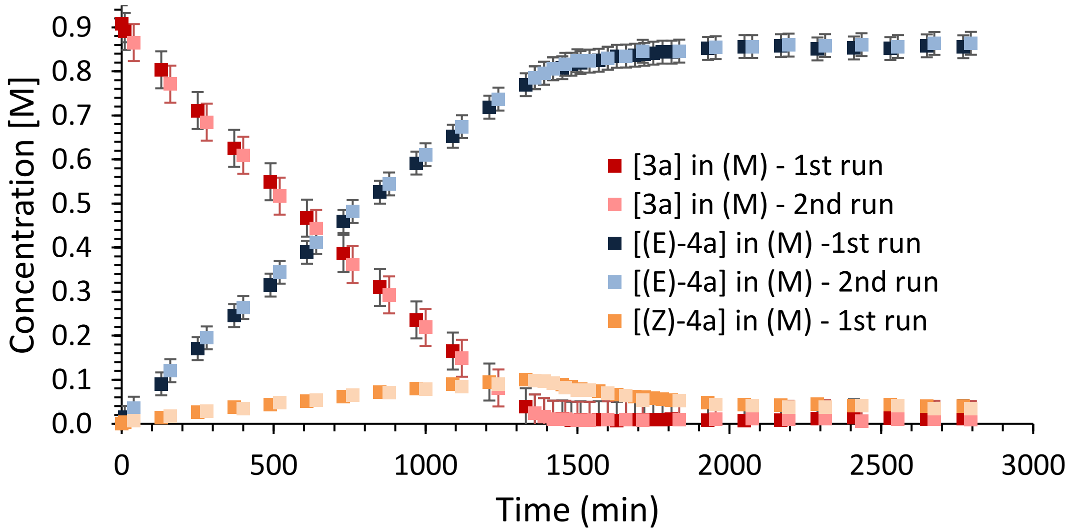

Figure S1. Reaction profile of Fe-catalyzed isomerization of **3a** under optimized conditions without (top) and with (bottom) standard errors bars.

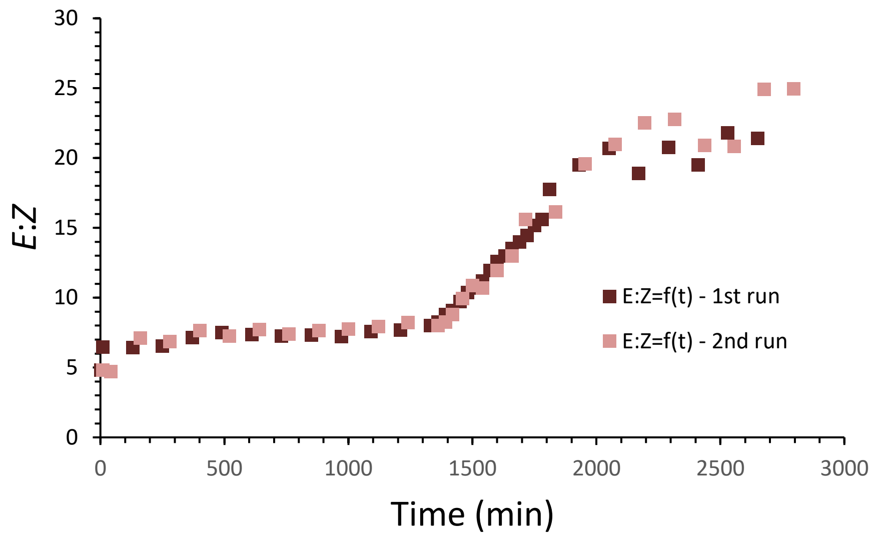

Figure S2. Stereoselectivity profile of Fe-catalyzed isomerization of **3a** under optimized conditions.

6b-Stoichiometric reactivity of complex 2a

Stoichiometric experiment between complex 2a and $Cy_2NH{\cdot}BH_3$ (Scheme 5, B.1): In an argon-filled glovebox, **2a** (14.6 mg, 0.025 mmol) was dissolved in deuterated benzene and added to a J-Young tap NMR tube containing $Cy_2NH.BH_3$ (4.9 mg, 0.025 mmol). The reaction vessel was then removed from the glovebox and monitored in a Bruker 400 MHz spectrometer at 60 °C. Signals for paramagnetic species were recorded within a spectral width from 120 to -120 ppm and assigned according to those obtained for isolated **2a** and **[2b]₂**. The formation of $SiMe_4$ was recognized by the emergence of a singlet signal at 0.0 ppm. A peak which corresponds to H_2 in solution was also detected at 4.5 ppm. Finally, relevant peaks that allow identification of **5** are described as follows. **^{1}H NMR** (400 MHz, C_6D_6, 60 °C) *δ* 4.88 (m, 1H, B*H*), 3.46 (t, 1H, *J*= 12.0 Hz, NC*H*), 2.75 (t, 1H, *J*= 12.0 Hz, NC*H*), 1.7-0.9 (m, 20 H), 0.53 (d, 2H, *J* = 4 Hz, C*H*$_2$TMS), 0.17 (s, 9H, Si*Me*$_3$). **^{11}B NMR** (300 MHz, C_6D_6, 60 °C) 40.1 ppm.

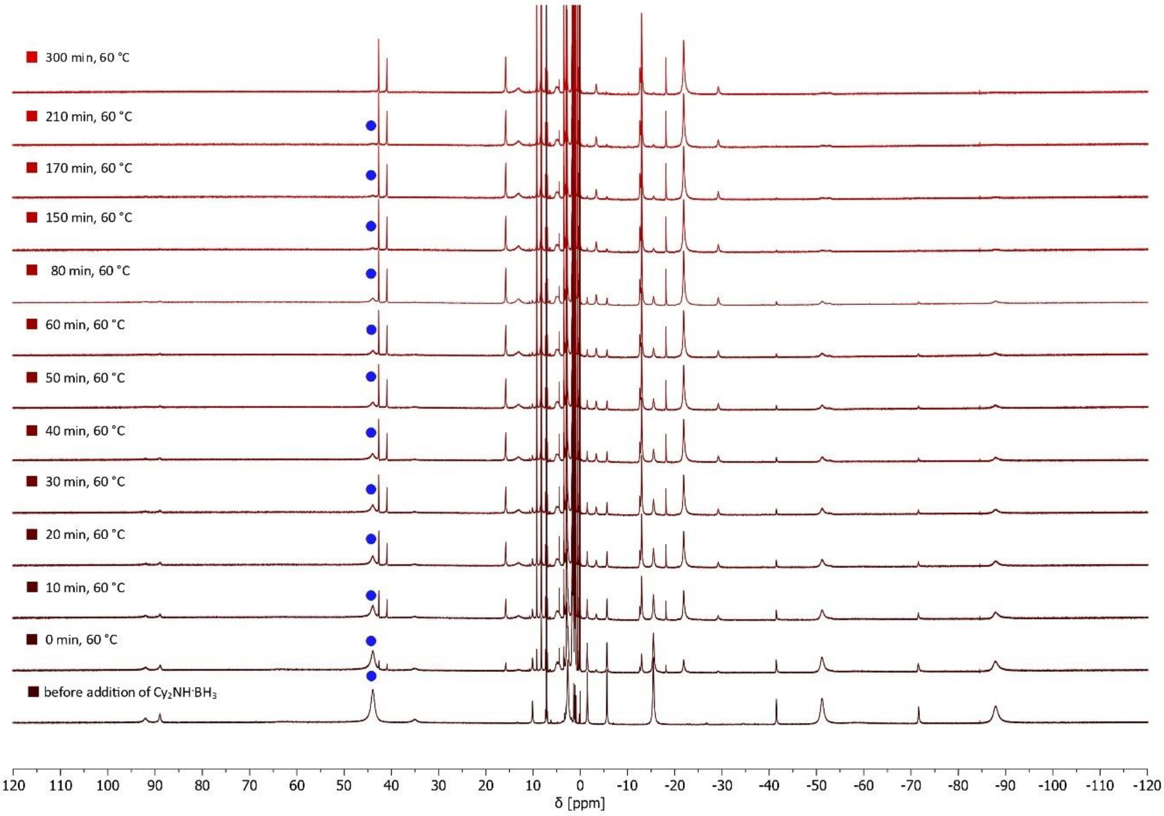

Figure S3. ^{1}H NMR trace of the stoichiometric experiment between **2a** and **$Cy_2NH{\cdot}BH_3$** at 60 °C (spectral width is from 120 to -120 ppm). Relevant Me signal from $SiMe_3$ (**2a**) is indicated by a blue dot.

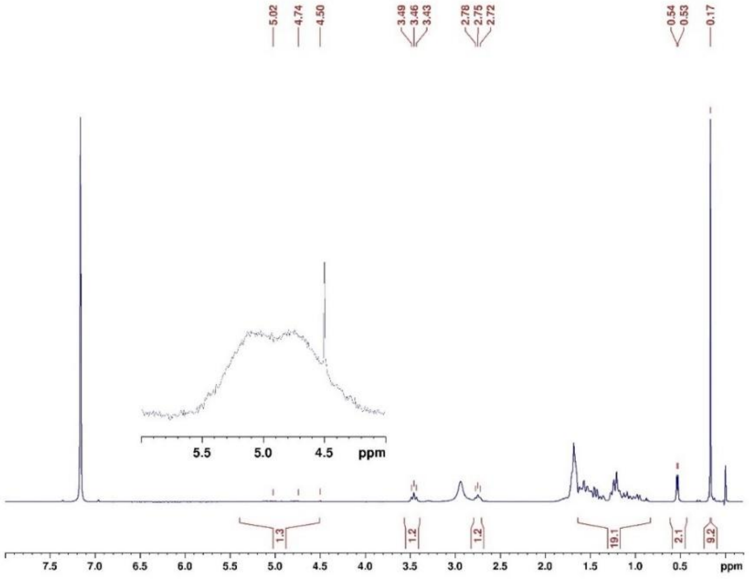

Figure S4. ^{1}H NMR trace of the stoichiometric experiment between **2a** and **$Cy_2NH^{\cdot}BH_3$** at 60 °C (spectral width is from 8.0 ppm to -0.2 ppm).

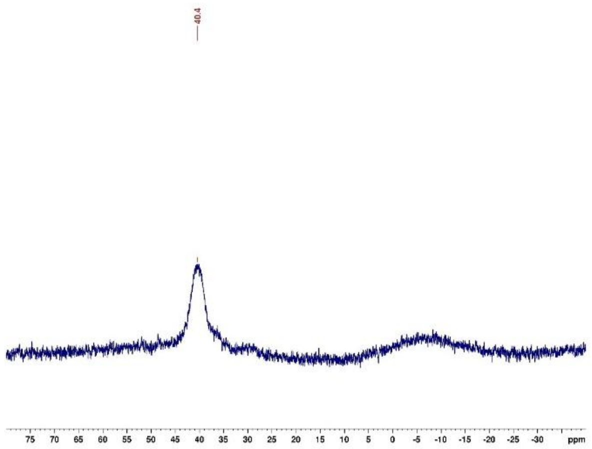

Figure S5. ^{11}B NMR trace of the stoichiometric experiment between **2a** and **$Cy_2NH^{\cdot}BH_3$** at 60 °C

Stoichiometric experiment between complex 2a and $Cy_2N^{\cdot}BH_2$ (Scheme 5, B.2): In an argon-filled glovebox, **2a** (14.6 mg, 0.025 mmol) was dissolved in deuterated benzene and added to a J-Young tap NMR tube containing $Cy_2N.BH_2$ (4.8 mg, 0.025 mmol). The reaction vessel was then removed from the glovebox and monitored in a Bruker 400 MHz spectrometer at 60 °C. Signals for paramagnetic species were recorded within a spectral width from 120 to -120 ppm and assigned according to those obtained for isolated **2a** and **$[2b]_2$**.

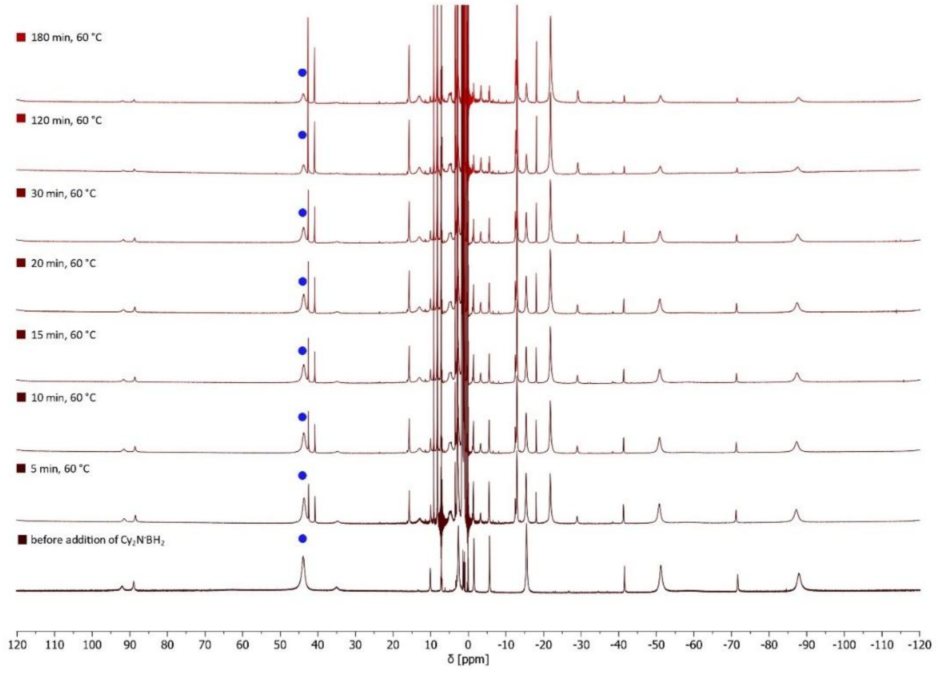

Figure S6. ^{1}H NMR trace of the stoichiometric experiment between **2a** and **$Cy_2N{\cdot}BH_2$** at 60 °C (spectral width is from 120 to -120 ppm). Relevant Me signal from $SiMe_3$ (**2a**) indicated by a blue dot.

6c-Catalytic reactivity of hydride complexes

Catalytic reactivity of $[2b]_2$ with allylbenzene 3a (entries 7-8, Table 1)

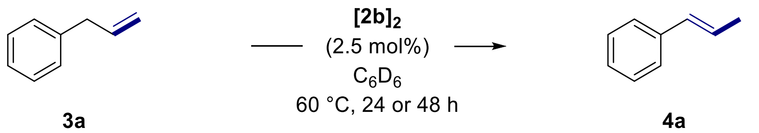

3a **4a**

In an argon-filled glovebox, **$[2b]_2$** (12.4 mg, 0.0125 mmol) was dissolved in deuterated benzene and added to an oven-dried 10 mL screw-capped tube equipped with a magnetic stir bar. Next, allylbenzene **3a** (59.1 mg, 0.5 mmol) was added at room temperature. The color of the solution turns immediately from brown to red orange. Then, the reaction was stirred at 60 °C for 24 h or 48 h. Upon completion, the reaction mixture was exposed to air and 1,3,5-trimethoxybenzene (42 mg, 0.25 mmol, 0.5 equiv.) was added to the reaction vessel. ^{1}H NMR analysis using 1,3,5-trimethoxybenzene as internal standard revealed **>99% conv., 99% yield** of prop-1-en-1-ylbenzene **4a**, and *E*:*Z* of **8:1** (for a 24-h reaction time) or **29:1** (for a 48-h reaction time)

Catalytic reactivity of $[2b]_2$ with (*Z*)-1,2-diphenylethene (*Z*)-3b (Scheme 3)

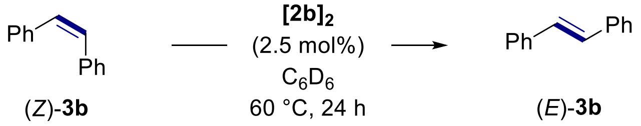

(*Z*)-**3b** (*E*)-**3b**

In an argon-filled glovebox, **[2b]₂** (12.4 mg, 0.0125 mmol) was dissolved in deuterated benzene and added to an oven-dried 10 mL screw-capped tube equipped with a magnetic stir bar. Next, (*Z*)-1,2-diphenylethene **(*Z*)-3b** (90.1 mg, 0.5 mmol) was added at room temperature. Upon heating at 60 °C, the color of the solution turns immediately from brown to red. Then, the reaction was stirred at 60 °C for 24 h. Upon completion, the reaction mixture was exposed to air and 1,3,5-trimethoxybenzene (42 mg, 0.25 mmol, 0.5 equiv.) was added to the reaction vessel. ^{1}H NMR analysis using 1,3,5-trimethoxybenzene as internal standard revealed **>99% conv., 99% yield** of (*E*)-1,2-diphenylethene **(*E*)-3b**.

Catalytic reactivity of 2c with allylbenzene 3a

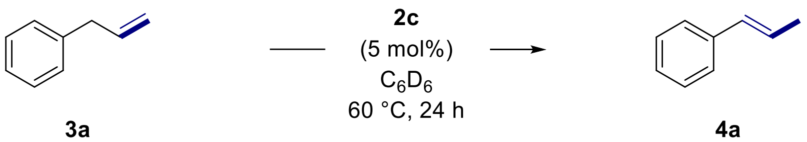

3a 4a

In an argon-filled glovebox, **2c** (15.5 mg, 0.025 mmol) was dissolved in deuterated benzene and added to an oven-dried 10 mL screw-capped tube equipped with a magnetic stir bar. Next, allylbenzene **3a** (59.1 mg, 0.5 mmol) was added at room temperature. Then, the reaction was stirred at 60 °C for 24 h. Upon completion, the reaction mixture was exposed to air and 1,3,5-trimethoxybenzene (42 mg, 0.25 mmol, 0.5 equiv.) was added to the reaction vessel. ^{1}H NMR analysis using 1,3,5-trimethoxybenzene as internal standard revealed **no conversion**.

Catalytic reactivity of 2h with allylbenzene 3a

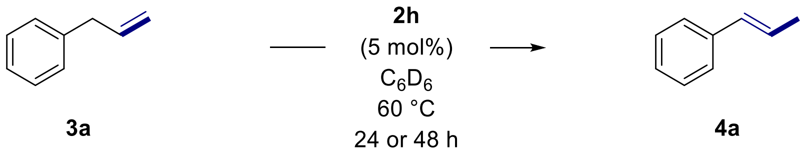

3a 4a

In an argon-filled glovebox, **2h** (7.7 mg, 0.0125 mmol) was dissolved in deuterated benzene and added to an oven-dried 10 mL screw-capped tube equipped with a magnetic stir bar. Next, allylbenzene **3a** (29.6 mg, 0.25 mmol) was added at room temperature. Then, the reaction was stirred at 60 °C for 24 h or 48 h. Upon completion, the reaction mixture was exposed to air and 1,3,5-trimethoxybenzene (42 mg, 0.25 mmol, 1 equiv.) was added to the reaction vessel. ^{1}H NMR analysis using 1,3,5-trimethoxybenzene as internal standard revealed **99% conv., 99% yield** of prop-1-en-1-ylbenzene **4a,** and *E*:*Z* of **13:1** (for a 24-h reaction time) or **32:1** (for a 48-h reaction time).

Catalytic reactivity of [(^{Me}BDI^{iPr})Fe-H]$_2$ with allylbenzene 3a

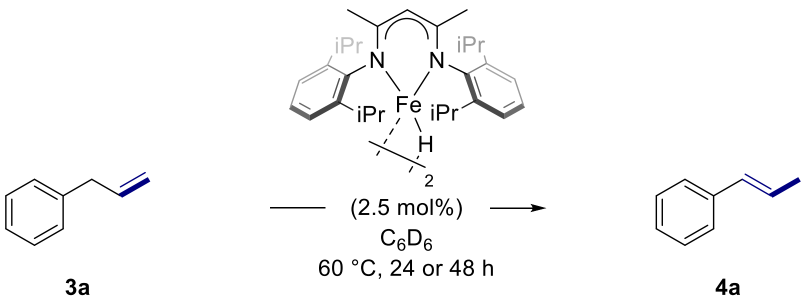

In an argon-filled glovebox, **[(^{Me}BDI^{iPr})Fe-H]$_2$** (11.8 mg, 0.0125 mmol) was dissolved in deuterated benzene and added to an oven-dried 10 mL screw-capped tube, equipped with a magnetic stir bar. Next, allylbenzene **3a** (59.1 mg, 0.5 mmol) was added at room temperature. Upon heating at 60 °C, the color of the solution turns from brown-yellow to clear yellow. Then, the reaction was stirred at 60 °C for 24 h. Upon completion, the reaction mixture was exposed to air and 1,3,5-trimethoxybenzene (42 mg, 0.25 mmol, 0.5 equiv.) was added to the reaction vessel. ^{1}H NMR analysis using 1,3,5-trimethoxybenzene as internal standard revealed **11% conv., 11% yield** of prop-1-en-1-ylbenzene **4a**. In addition, the reaction was conducted for an extended reaction time. ^{1}H NMR analysis using 1,3,5-trimethoxybenzene as internal standard revealed: **13% conv., 13% yield** after 48 h and **40% conv., 32% yield** after 7 days.

6d-Radical-trapping experiments

In an argon-filled glovebox, **2a** (14.6 mg, 0.025 mmol) was dissolved in deuterated benzene and added to an oven-dried 10 mL screw-capped tube, equipped with a magnetic stir bar, containing $Cy_2NH{\cdot}BH_3$ (4.9 mg, 0.025 mmol). Next, allylbenzene **3a** (59.1 mg, 0.5 mmol) was added followed by the radical trap (0.25 mmol, 0.5 equiv.) and the reaction was stirred at 60 °C for 24 h or 48 h. Upon completion, the reaction mixture was exposed to air and 1,3,5-trimethoxybenzene (42 mg, 0.25 mmol, 0.5 equiv.) was added to the reaction vessel. Spectroscopic yield and stereoselectivity given by *E*:*Z* were determined by ^{1}H NMR analysis using 1,3,5-trimethoxybenzene as internal standard.

Table S4. Radical trap experiments for Fe-catalyzed isomerization of **3a**

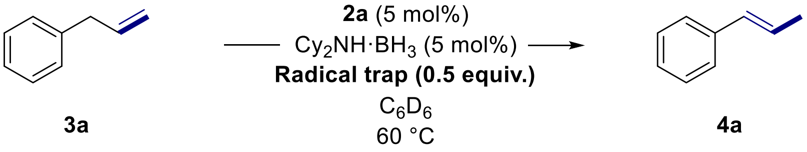

Entry	Radical trap	t (h)	Conv. [%][b]	Yield [%][b]	*E*:*Z*[b]
1	1,10-dihydroanthracene	24	88	88	8:1
2	1,10-dihydroanthracene	48	>99	99	24:1
3	1,1-diphenylethene	24	65	65	8:1
4	1,1-diphenylethene	48	>99	99	19:1

Catalytic isomerisation of **3a** in the presence of 1,10-dihydroantrancene proceeds without any significant loss of activity or selectivity in 24 h (88% yield, *E*:*Z* 8:1) or to competition in 48 h (>99% yield, E:Z 24:1) (entries 1-2, Table S4). In this case, no traces of the radical trapping product, anthracene, were detected by either ^{1}H NMR or GC, indicating that the radical pathways are not involved in the positional or geometric isomerisation regimes. The isomerisation of allylbenzene **3a** was also performed in the presence of 1,1-diphenylethylene as other radical trapping reagent (entries 3-4, Table S4). Although a modest decrease in activity or selectivity was noted within 24 h and 48 h respectively, which might be attributed to partial/reversible inhibition of the catalyst by competitive interaction with 1,1-diphenylethylene, no radical trapping product was observed, consistent with a radical-free regime.

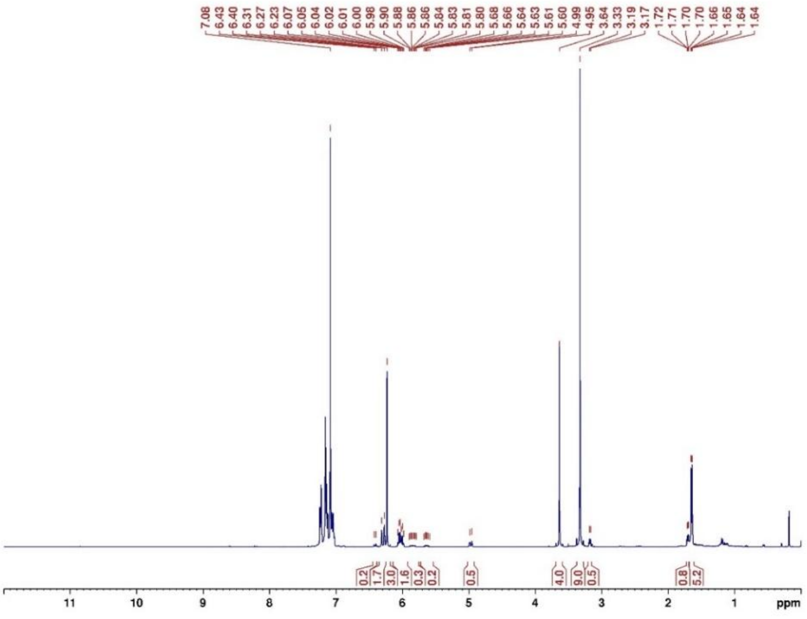

Figure S7. ^{1}H NMR spectrum of radical trap experiment with 9,10-dihydroanthracene (24 h)

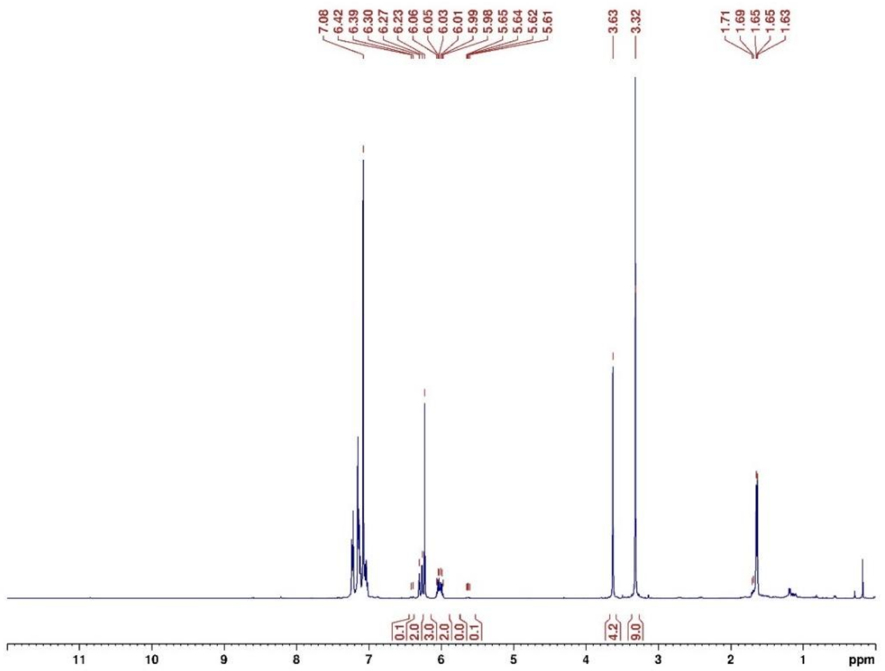

Figure S8. ^{1}H NMR spectrum of radical trap experiment with 9,10-dihydroanthracene (48 h)

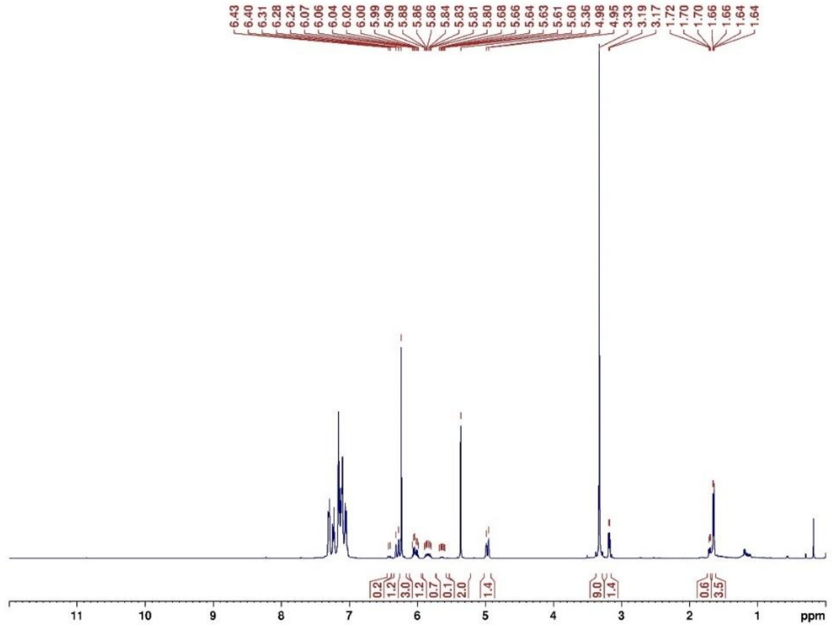

Figure S9. ^{1}H NMR spectrum of radical trap experiment with 1,1-diphenylethene (24 h)

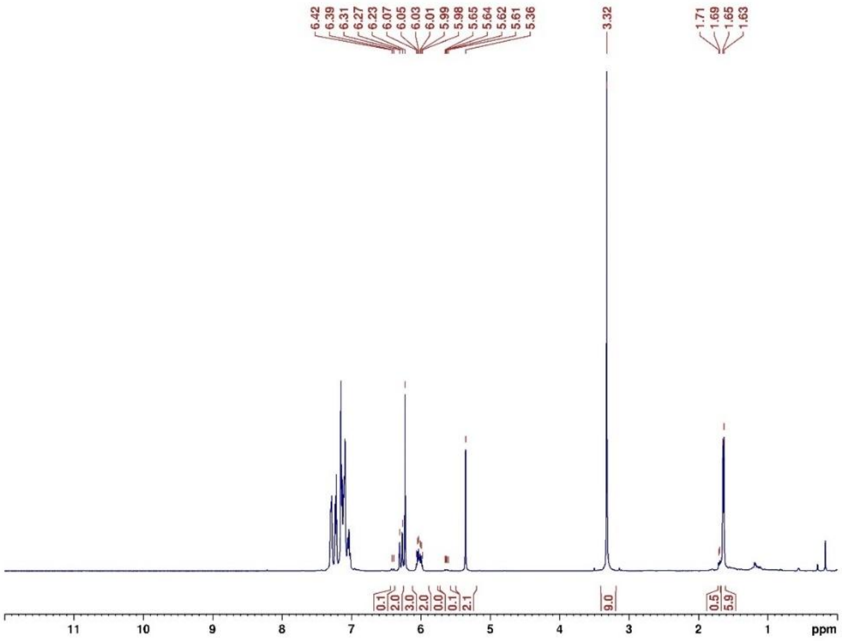

Figure S10. ^{1}H NMR spectrum of radical trap experiment with 1,1-diphenylethene (48 h)

6e-Deuterium-labelling experiments

Synthesis of deuterated dicyclohexylamine borane

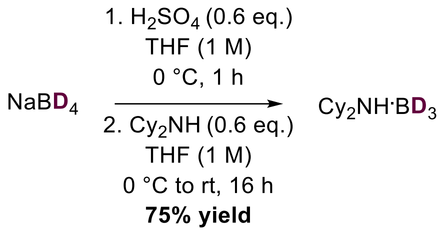

To a suspension of sodium borodeuteride $NaBD_4$ (0.5 g, 12.2 mmol) in THF (5 mL) was added H_2SO_4 (0.72 g, 0.4 mL, 7.3 mmol) dropwise at 0 °C over a period of 1 h, followed by dicyclohexylamine Cy_2NH (1.3 g, 7.3 mmol). The reaction mixture was then stirred vigorously for 16 h at room temperature. Upon completion, THF and volatiles were evacuated under reduced pressure until dryness. The solid was extracted by DCM (2 x 5 mL) and recombined organic extracts was washed by water (3 x 10 mL), dried over $MgSO_4$, filtered and subjected to evaporation under reduced pressure to afford deuterated dicyclohexylamine borane $Cy_2NH{\cdot}BD_4$ as white crystalline solid (1.1 g, 5.5 mmol, 75%). **^{1}H NMR** (400 MHz, $CDCl_3$, RT) δ 2.94 (br s, 1H, N*H*), 2.80 (tt, 2H, *J* = 3.3, 11.6 Hz, 2 x NC*H*), 1.90-1.76 (m, 8H, Cy-*H*), 1.70-1.53 (m, 6H, Cy-*H*), 1.32-1.06 (m, 6H, Cy-*H*). **^{11}B NMR** (96 MHz, $CDCl_3$, RT) δ -19.4 (br s). **^{13}C{^{1}H} NMR** (100 MHz, $CDCl_3$, RT) δ 60.5, 60.4, 30.8, 30.7, 29.5, 29.5, 25.6, 25.4, 25.2.

Synthesis of 1-(but-3-en-1-yl-1,1-*d₂*)-4-methylbenzene (3ae-*d₂*)

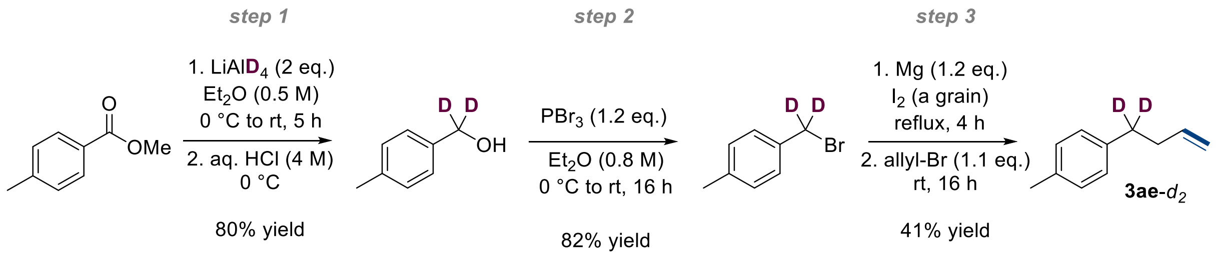

Step 1: $LiAlD_4$ (2.5 g, 60.0 mmol) was added portion wise to a stirred solution of methyl 4-methylbenzoate (4.5 g, 30.0 mmol) in Et_2O (60 mL) at 0 °C. The cooling was removed 30 min after addition and the reaction mixture was allowed to stir for 5 h at room temperature, then quenched by dropwise addition of an aqueous solution of HCl (10 mL, 4 M), at 0 °C. Filtration was performed through a pad of Celite, and the aqueous layer was extracted with Et_2O (3 x 30 mL). Organic extracts were recombined, dried over $MgSO_4$, filtered and organic solvent was removed under reduced pressure to afford *p*-tolylmethan-d_2-ol as white crystalline solid (3.0 g, 24.0 mmol, 80%). **^{1}H NMR** (400 MHz, $CDCl_3$, RT) δ 7.26 (d, 2H, J = 8.0 Hz), 7.18 (d, 2H, J = 8.0 Hz), 2.35 (s, 3H), 1.55 (br s, 1H). **^{13}C{^{1}H} NMR** (100 MHz, $CDCl_3$, RT) δ 137.8, 137.5, 129.3, 127.2, 29.7 (m), 21.2.

Step 2: PBr_3 (7.8 g, 28.8 mmol) was added dropwise to a stirred solution of *p*-tolylmethan-d_2-ol (3.0 g, 24.0 mmol) in Et_2O (30 mL) at 0 °C. The cooling was removed 30 min after addition and the reaction mixture was allowed to stir for 16 h at room temperature, then quenched by dropwise addition of H_2O (30 mL), at 0 °C. Aqueous layer was extracted with Et_2O (3 x 30 mL). Organic extracts were recombined, dried over $MgSO_4$, filtered and organic solvent was removed under reduced pressure. Purification by flash chromatography on silica gel using pentane as eluant yielded 1-(bromomethyl-d_2)-4-methylbenzene as white crystalline solid (3.7 g, 19.8 mmol, 82%). **^{1}H NMR** (400 MHz, $CDCl_3$, RT) δ 7.20 (d, 2H, J = 8.0 Hz), 7.06 (d, 2H, J = 8.0 Hz), 2.26 (s, 3H). **^{13}C{^{1}H } NMR** (100 MHz, $CDCl_3$, RT) δ 138.4, 134.7, 129.5, 129.0, 33.4 (m), 21.3.

Step 3: To a stirred suspension of Mg (722.0 mg, 29.7 mmol) and I_2 (a grain) in THF (15 mL), at room temperature, was added dropwise a solution of 1-(bromomethyl-d_2)-4-methylbenzene (3.7 g, 19.8 mmol) in THF (5 mL), at room temperature. The reaction mixture was stirred for 1 h at room temperature then refluxed for an additional 4 h. The resulting slurry was allowed to cool down to room temperature, at which allyl bromide (3.4 g, 28.1 mmol) was added dropwise. Next, the reaction was allowed to stir for an additional 16 h. Upon completion, a saturated aqueous solution of NH_4Cl (20 mL) was added at 0 °C. Aqueous layer was extracted with Et_2O (3 x 30 mL). Organic extracts were recombined, dried over $MgSO_4$, filtered and organic solvent and volatiles were removed under reduced pressure. Purification by flash chromatography on silica gel using pentane as eluant yielded 1-(but-3-

en-1-yl-1,1-d_2)-4-methylbenzene as color less oil (1.2 g, 8.1 mmol, 41%). **^{1}H NMR** (400 MHz, $CDCl_3$, RT) δ 7.16 (m, 4H), 5.93 (tdd, 1H, J = 6.6, 10.2, 17.1 Hz), 5.11 (d, 1H, J = 17.1 Hz), 5.04 (d, 1H, J = 10.2 Hz), 2.41 (d, 2H, J = 6.6 Hz), 2.39 (s, 3H). **^{13}C{^{1}H } NMR** (100 MHz, $CDCl_3$, RT) δ 138.7, 138.2, 135.2, 129.0, 128.3, 114.8, 35.5, 34.2, 21.0.

Deuterium-labelling experiment with allylbenzene 3a using $Cy_2NH·BD_3$ (Scheme 6, A.1)

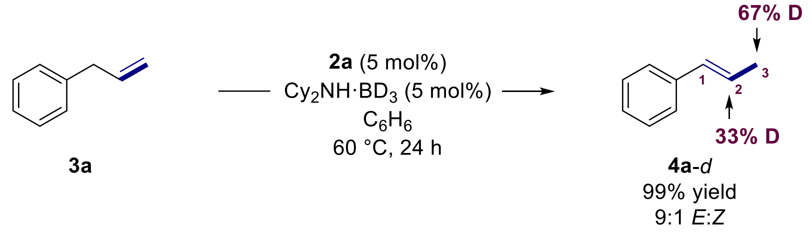

In an argon-filled glovebox, **2a** (14.6 mg, 0.025 mmol) was dissolved in deuterated benzene and added to an oven-dried 10 mL screw-capped tube, equipped with a magnetic stir bar, containing $Cy_2NH.BD_3$ (5.0 mg, 0.025 mmol). Next, allylbenzene **3a** (59.1 mg, 0.5 mmol) was added and the reaction was stirred at 60 °C for 24 h. Upon completion, the reaction mixture was exposed to air and 1,3,5-trimethoxybenzene (42 mg, 0.25 mmol, 0.5 equiv.) was added to the reaction vessel. ^{1}H NMR analysis using 1,3,5-trimethoxybenzene as internal standard revealed **99% yield** of prop-1-en-1-ylbenzene **4a**-*d*, and *E*:*Z* of **9:1**. ^{2}H NMR analysis displays the exclusive D-incorporation onto **C_2** and **C_3** with relative percentages of **33%** and **67%** respectively.

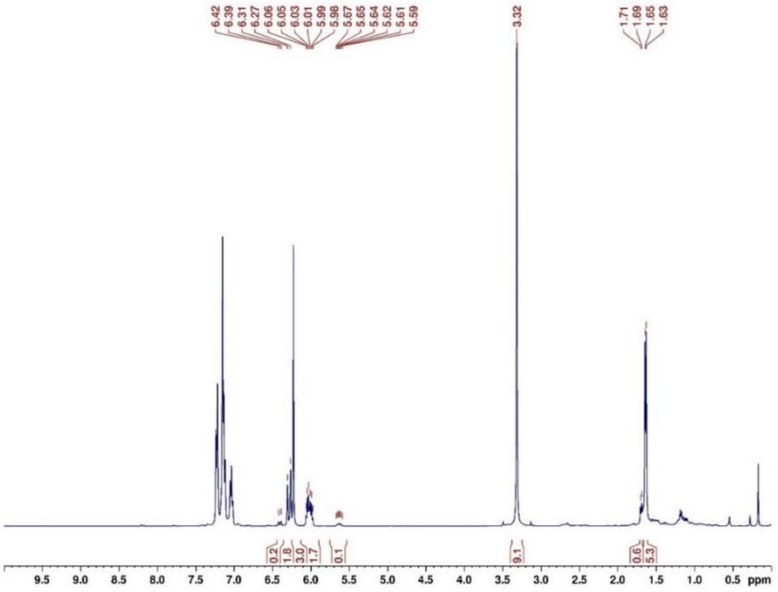

Figure S11. ^{1}H NMR spectrum of deuteriation experiment with allylbenzene **3a**

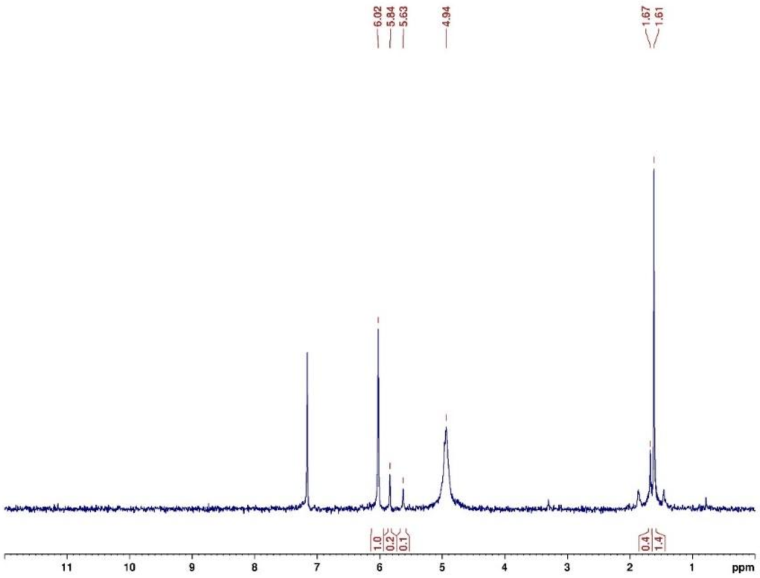

Figure S12. ^2H NMR spectrum of deuteriation experiment with allylbenzene **3a**

Deuterium-labelling experiment with (*Z*)-1,2-diphenylethene (*Z*)-3b using $Cy_2NH{\cdot}BD_3$ (Scheme 6, A.1)

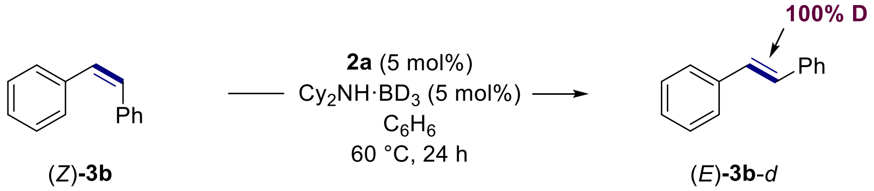

(*Z*)-**3b** (*E*)-**3b**-*d* 99% yield

In an argon-filled glovebox, **2a** (14.6 mg, 0.025 mmol) was dissolved in deuterated benzene and added to an oven-dried 10 mL screw-capped tube, equipped with a magnetic stir bar, containing $Cy_2NH.BD_3$ (5.0 mg, 0.025 mmol). Next, (*Z*)-1,2-diphenylethene **(*Z*)-3b** (90.1 mg, 0.5 mmol) was added and the reaction was stirred at 60 °C for 24 h. Upon completion, the reaction mixture was exposed to air and 1,3,5-trimethoxybenzene (42 mg, 0.25 mmol, 0.5 equiv.) was added to the reaction vessel. ^1H NMR analysis using 1,3,5-trimethoxybenzene as internal standard revealed **99% yield** of (*E*)-1,2-diphenylethene **(*E*)-3b**. ^2H NMR analysis displays D-incorporation onto (*E*)-1,2-diphenylethene **(*E*)-3b** (*c.a.* 100%).

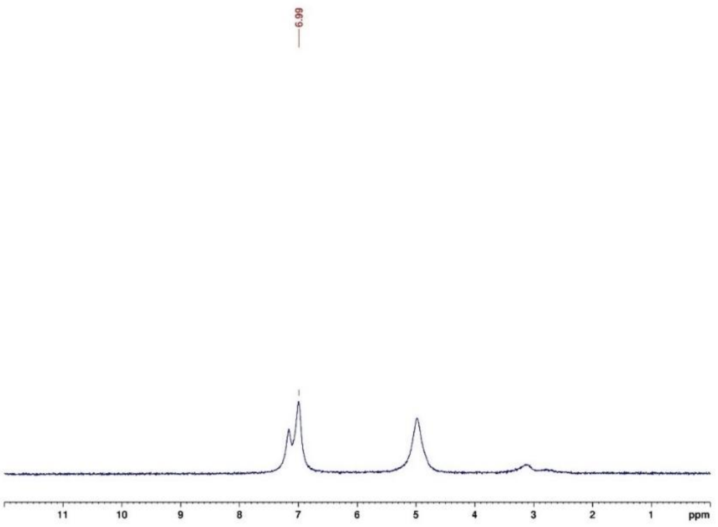

Figure S13. ^2H NMR spectrum of deuteriation experiment with (*E*)-1,2-diphenylethene **(*E*)-3b**

Deuterium-labelling experiment with 1-(but-3-en-1-yl-1,1-d$_2$)-4-methylbenzene 3ae-*d$_2$*

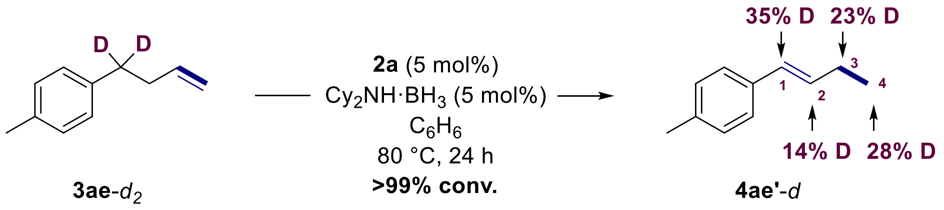

In an argon-filled glovebox, **2a** (14.6 mg, 0.025 mmol) was dissolved in benzene and added to an oven-dried 10 mL screw-capped tube, equipped with a magnetic stir bar, containing $Cy_2NH.BH_3$ (4.9 mg, 0.025 mmol). Next, 1-(but-3-en-1-yl-1,1-d2)-4-methylbenzene **3ae** (74.1 mg, 0.5 mmol) was added and the reaction was stirred at 80 °C for 24 h. Upon completion, the reaction mixture was exposed to air and the crude was subjected to NMR analysis. ^1H NMR analysis revealed >**99% conversion**. ^2H NMR analysis displays the D-incorporation onto **C_1**, **C_2**, **C_3** and **C_4** with percentages of **35%**, **14%**, **23%** and **28%** respectively.

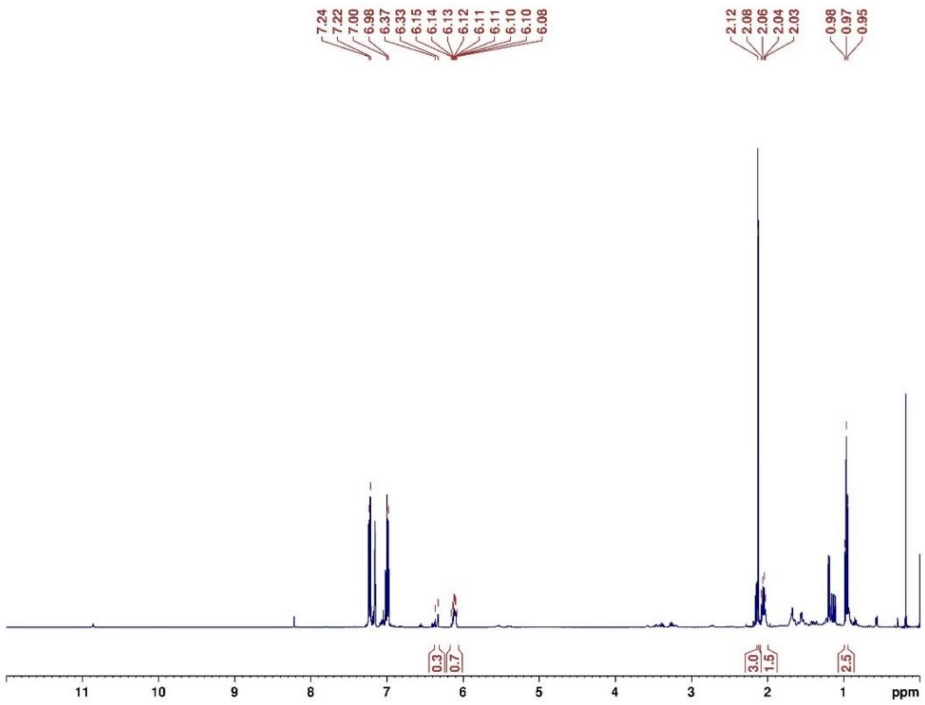

Figure S14. ^{1}H NMR spectrum of deuteriation experiment with 1-(but-3-en-1-yl-1,1-d_2)-4-methylbenzene **3ae**-d_2

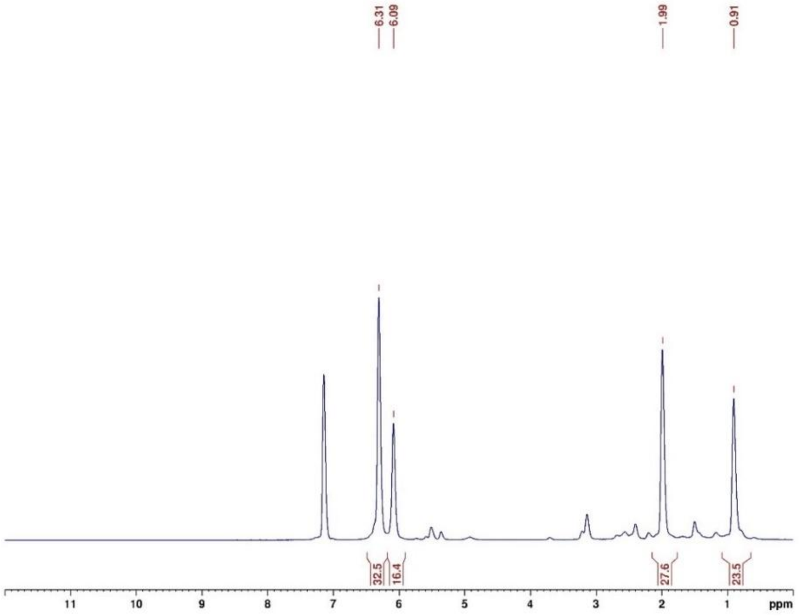

Figure S15. ^{2}H NMR spectrum of deuteriation experiment with 1-(but-3-en-1-yl-1,1-d_2)-4-methylbenzene **3ae**-d_2

Deuterium-labelling experiment with (*E*)-5-(prop-1-en-1-yl)benzo[d][1,3]dioxole (*E*)-4m using $Cy_2NH{\cdot}BD_3$ (Scheme 6, A.2)

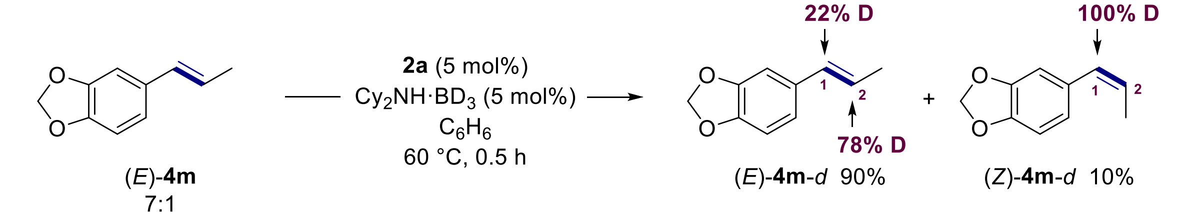

In an argon-filled glovebox, **2a** (14.6 mg, 0.025 mmol) was dissolved in benzene and added to an oven-dried 10 mL screw-capped tube, equipped with a magnetic stir bar, containing $Cy_2NH.BD_3$ (5.0 mg, 0.025 mmol). Next, (*E*)- 5-(prop-1-en-1-yl)benzo[d][1,3]dioxole (*E*)-**4m** (81.1 mg, 0.5 mmol) was added and the reaction was stirred at 60 °C for 0.5 h. Upon completion, the reaction mixture was exposed to air and the crude was subjected to ^{2}H NMR analysis. For (*E*)-**4m**-*d*, the incorporation occurred preferentially at $\mathbf{C_2}$ ($\mathbf{C_1}/\mathbf{C_2}$ ratio =22/78) while for (*Z*)-**4m**-*d*, it took place predominantly at $\mathbf{C_1}$ ($\mathbf{C_1}/\mathbf{C_2}$ ratio = 100/0).

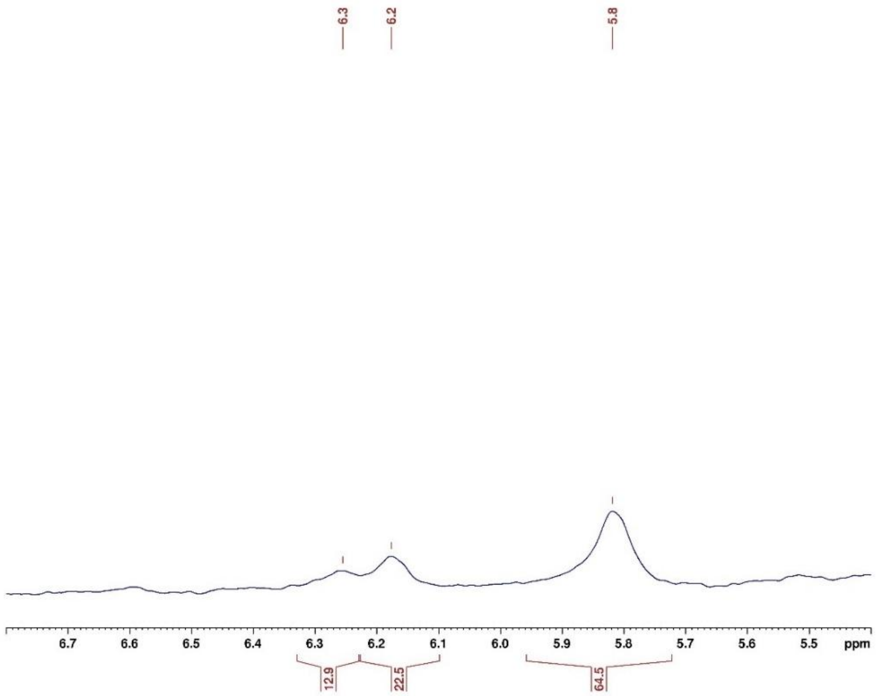

Figure S16. ^{2}H NMR spectrum of deuteriation experiment with (*E*)-5-(prop-1-en-1-yl)benzo[d][1,3]dioxole (*E*)-**4m**

Deuterium labelling experiment with (*E*)-prop-1-en-1-ylbenzene (*E*)-4a using $Cy_2NH{\cdot}BD_3$

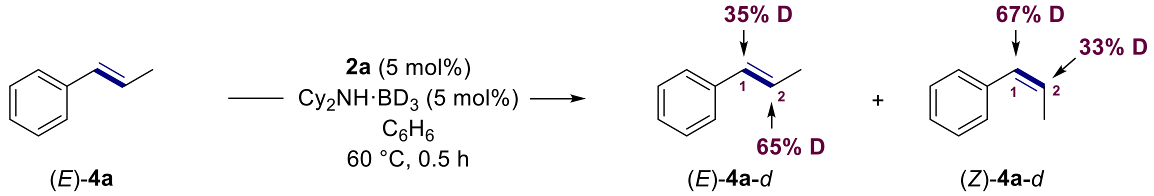

In an argon-filled glovebox**, 2a** (14.6 mg, 0.025 mmol) was dissolved in dry and O_2-free benzene and added to an oven-dried 10 mL screw-capped tube, equipped with a magnetic stir bar, containing $Cy_2NH.BD_3$ (5.0 mg, 0.025 mmol). Next, (*E*)-prop-1-en-1-ylbenzene (*E*)-**4a** (59.1 mg, 0.5 mmol) was added, and the reaction was allowed to proceed at 60 °C for 0.5 h. Upon completion, the reaction mixture was exposed to air and crude was subjected to ^{2}H NMR analysis. for (*E*)-**4a**-*d*, the incorporation occurred preferentially at $\mathbf{C_2}$ ($\mathbf{C_1}$/$\mathbf{C_2}$ ratio = 35/65) while for (*Z*)-**4a**-*d*, it took place predominantly at $\mathbf{C_1}$ ($\mathbf{C_1}$/$\mathbf{C_2}$ ratio = 67/33).

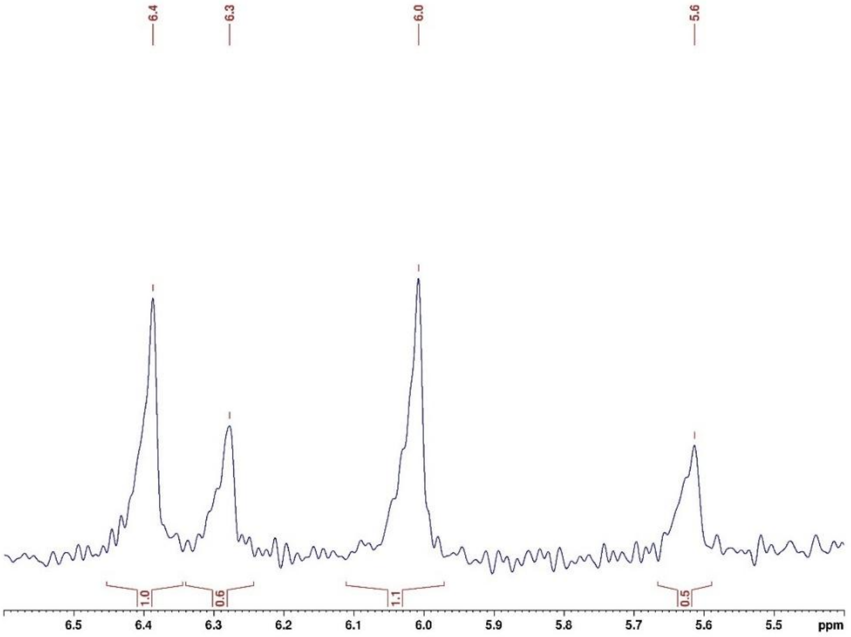

Figure S17. ^{2}H NMR spectrum of deuteriation experiment with (*E*)-prop-1-en-1-ylbenzene (*E*)-**4a**

Deuterium scrambling experiment (Scheme 6, B.)

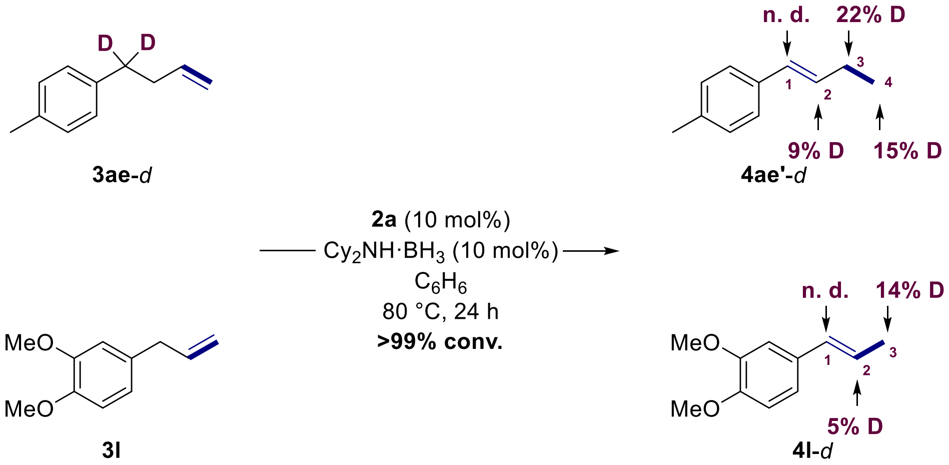

In an argon-filled glovebox, **2a** (14.6 mg, 0.025 mmol) was dissolved in benzene and added to an oven-dried 10 mL screw-capped tube, equipped with a magnetic stir bar, containing $Cy_2NH.BH_3$ (4.9 mg, 0.025 mmol). Next, 1-(but-3-en-1-yl-1,1-d2)-4-methylbenzene **3ae** (37.0 mg, 0.25 mmol) and 4-allyl-1,2-dimethoxybenzene **3l** (44.6 mg, 0.25 mmol) were added and the reaction was stirred at 80 °C for 24 h. Upon completion, the reaction mixture was exposed to air and the crude was subjected to NMR analysis. ^{1}H NMR analysis revealed >**99% conversion**. ^{2}H NMR analysis displays the D-incorporation onto **4ae’** and **4l** indication of a D-scrambling between substrates.

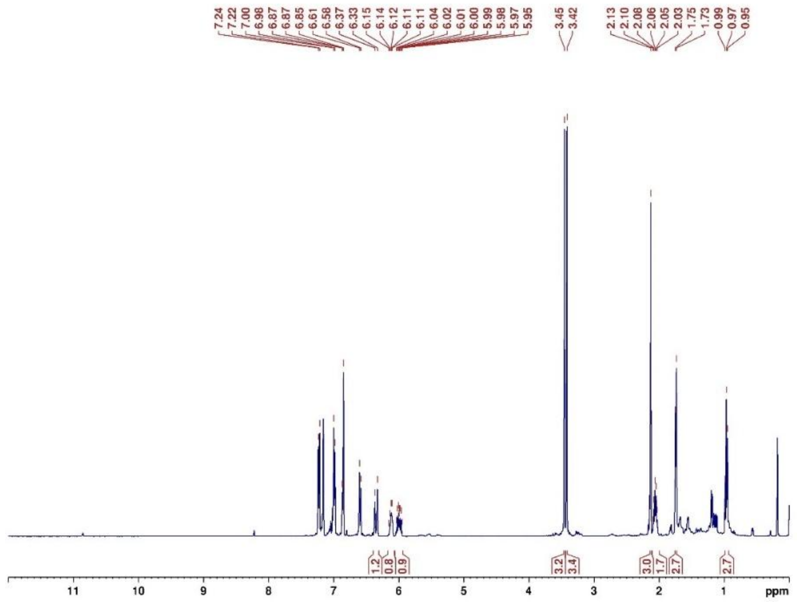

Figure S18. ^{1}H NMR spectrum of scrambling experiment with **3ae**-d_2 and **3l**

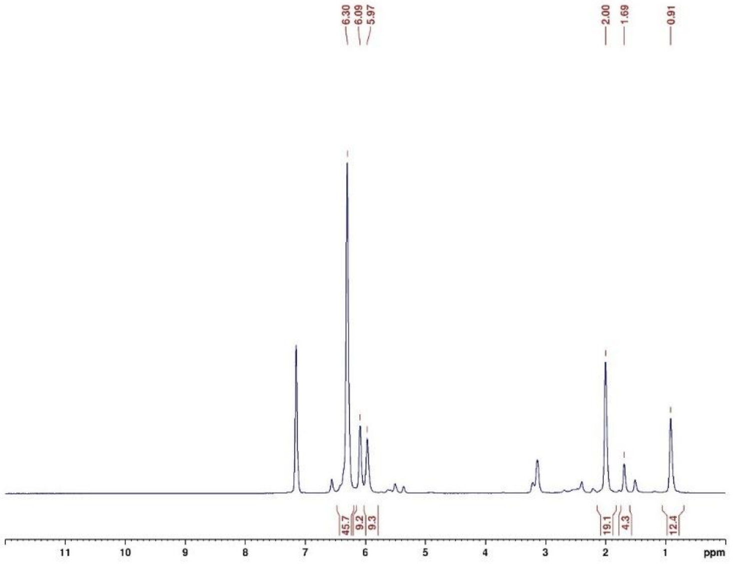

Figure S19. ^{2}H NMR spectrum of crossover experiment with **3ae**-d_2 and **3l**

6f-EPR experiments

X-band EPR spectra were recorded on a Bruker ELEXSYS 500 spectrometer equipped with a Bruker ER 4116DM X-band resonator and an Oxford Instruments continuous flow ESR 900 cryostat and ITC 503 temperature control system. The conditions used were:

Microwave frequency for perpendicular mode = 9.635 GHz, microwave frequency for parallel mode = 9.382 GHz, microwave power = 4.0 mW, modulation amplitude = 8 Gauss, modulation frequency = 100 KHz, Gain = 50 db and temperature =90 K or 10 K.

Quantification of the obtained signals was performed by plotting the area of the signal into a calibration curve obtained by using solutions of three different concentrations (0.5, 1, 2 mM) of $CuSO_4$ as reference standard.

In situ EPR studies of the reactivity of **[2b]₂** with respectively **3a** (20 equiv) and (*Z*)-**3b** (20 equiv.) at 25 °C and 60 °C were next conducted (Figure S20 and Figure S21). EPR spectra of **2a** (50 mM in benzene-d_6) in parallel mode shows a signal at g=7 characteristic of a Fe(II) species (Figure S20, black trace).In line with a previous work,[15] isolated hydride-bridge iron(II) dimer **$[2b]_2$** is EPR silent (Figure 20, red trace). Addition of 20 equiv. of alkene **3a** to isolated **$[2b]_2$** at 25 °C and immediate analysis yields a partial recovery of Fe(II) signal (Figure S20, blue trace), which is alike the signal of iron(II) alkyl complex **2a** (black trace). This signal persists after stirring for 60 min at 60 °C (pink trace). This Fe(II) signal could certainly be ascribed to the formation of the hydrometallation product of **3a** by **$[2b]_2$** (or more likely its monomer).

The same experiment was next carried out with stilbene (*Z*)-**3b** instead of **3a**. The addition of 20 equiv. of (*Z*)-**3b** to **$[2b]_2$** gives no Fe(II) signal at 25 °C (Figure S20, green trace) but the resurgence of a Fe(II)-alkyl signal was observed after stirring for 60 min at 60 °C (navy blue trace), ascribed to an hydrometallation process taking place. These experiments evidence **$[2b]_2$** (or its monomer) evolves through an hydrometallation in the presence of **3a** at 25 °C or 60 °C or (*Z*)-**3b** at 60 °C. The same behavior and reactivity were observed when **$[2b]_2$** was generated in situ from **2a** and $Cy_2NH\ BH_3$ (Figure S22 and Figure S23). It is pertinent to note that, in contrast to a previous work on ($^{Me}BDI^{iPr}$) Fe(II)-CH_2TMS,[8] only traces of Fe(I), that accounts for less than 1% of the total amount of iron species, was detected in perpendicular mode in these experiments with no traces of Fe(III) species.

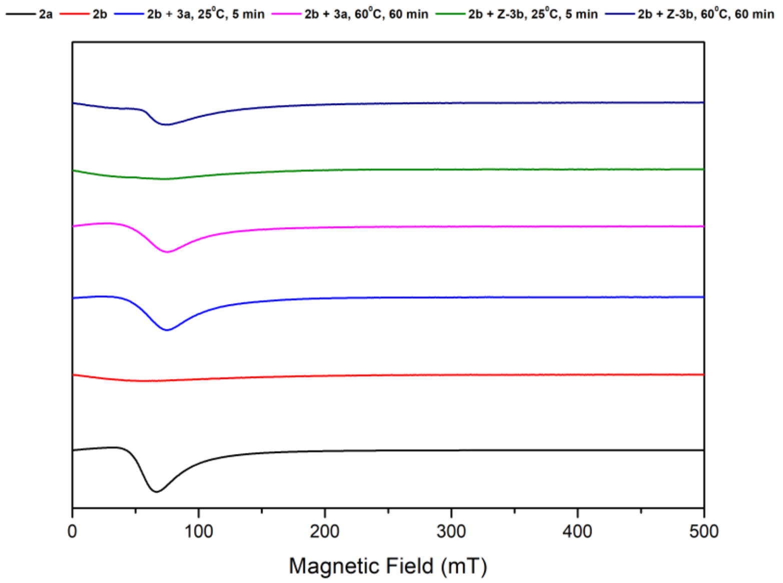

Figure S20. EPR spectra of **2a** and isolated **[2b]$_2$** and of reactions of **[2b]$_2$** with **3a** and (*Z*)-**3b** at 25 °C and 60 °C as shown in parallel mode. Conditions: MW freq. 9.382 GHz, MW power 4 mW, modulation amplitude 8 Gauss, gain 50 dB, temperature 90 K (except **2a** at 10 K). The relative signal intensity of **2a** was divided by a 10-factor.

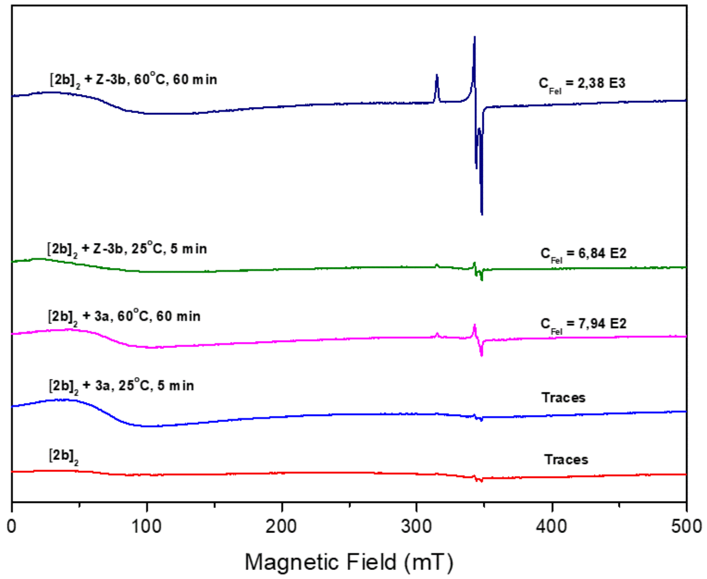

Figure S21. EPR spectra of **[2b]$_2$** and of reactions of **[2b]$_2$** with **3a** and (*Z*)-**3b** at 25 °C and 60 °C in perpendicular mode. Conditions: MW freq. 9.635 GHz, MW power 4 mW, modulation amplitude 8 Gauss, gain 50 dB, temperature 90 K. Concentrations in Fe(I) are in ppm.

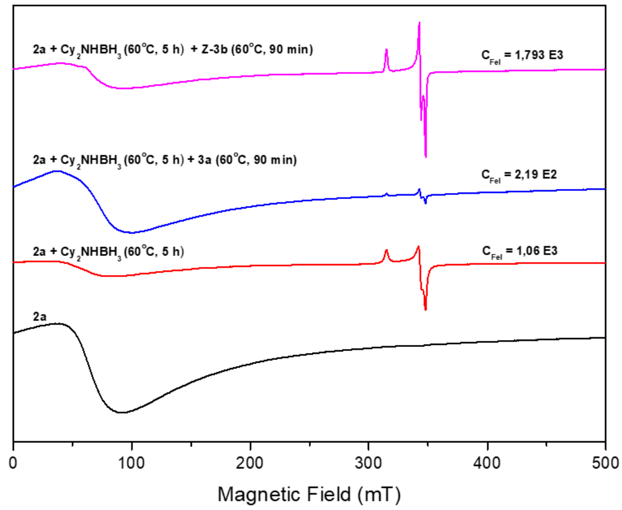

Figure S22. EPR spectra of **2a** and of reactions of **2a** with $Cy_2NH\ BH_3$ (5 h), $Cy_2NH\ BH_3$ (5 h) then **3a** (90 min) and $Cy_2NH{\cdot}BH_3$ (5 h) then (*Z*)-**3b** (90 min) at 60 °C in perpendicular mode. Conditions: MW freq. 9.382 GHz, MW power 4 mW, modulation amplitude 8 Gauss, gain 50 dB, temperature 10 K. Concentrations in Fe(I) are in ppm.

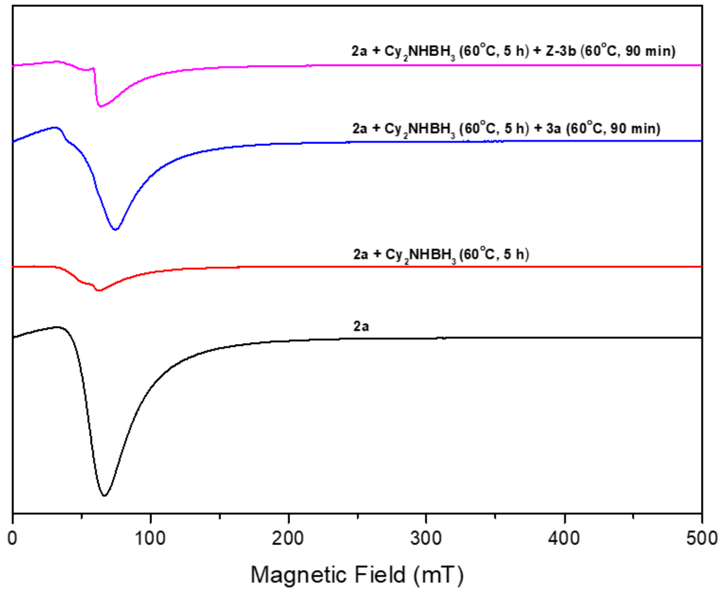

Figure S23. EPR spectra of **2a** and of reactions of **2a** with $Cy_2NH\ BH_3$ (5 h), $Cy_2NH\ BH_3$ (5 h) then **3a** (90 min) and $Cy_2NH{\cdot}BH_3$ (5 h) then (*Z*)-**3b** (90 min) at 60 °C in parallel mode. Conditions: MW freq. 9.635 GHz, MW power 4 mW, modulation amplitude 8 Gauss, gain 50 dB, temperature 10 K.

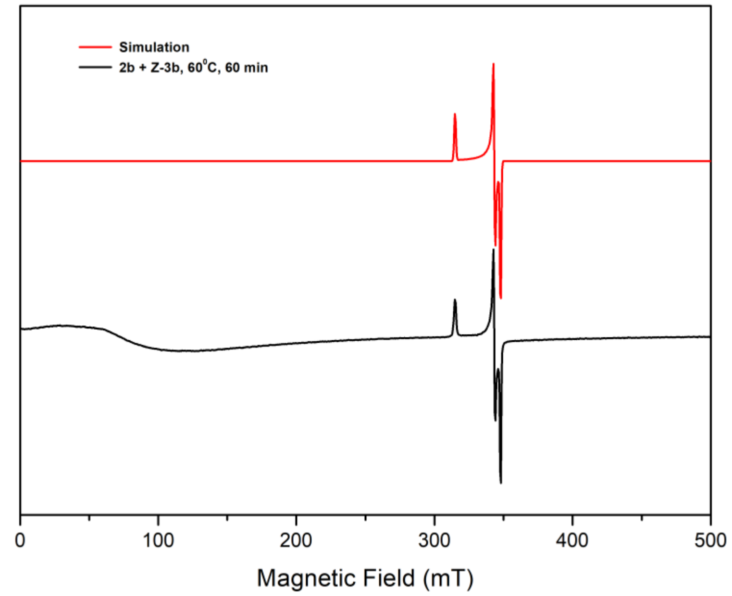

Figure S24. EPR spectrum of reaction of **[2b]$_2$** with (*Z*)-**3b** at 60 °C in perpendicular mode and the corresponding simulation. Conditions: MW freq. 9.635 GHz, MW power 4 mW, modulation amplitude 8 Gauss, gain 50 dB, temperature 90 K.

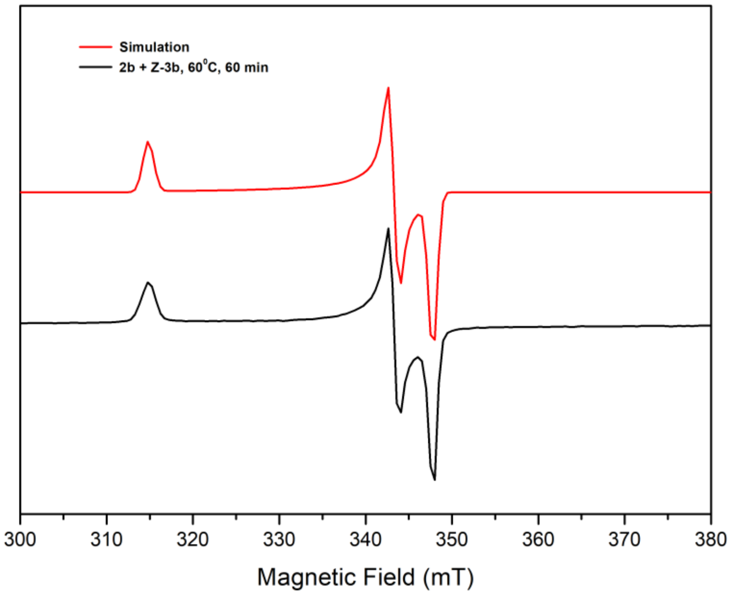

Figure S25. Zoom of EPR spectrum of reaction of **[2b]$_2$** with (*Z*)-**3b** at 60 °C in perpendicular mode and the corresponding simulation. Conditions: MW freq. 9.635 GHz, MW power 4 mW, modulation amplitude 8 Gauss, gain 50 dB, temperature 90 K.

6g-Kinetic studies

General procedure for kinetic studies: Reaction were performed on a 0.5 mmol scale. In an argon-filled glovebox, **2a** was dissolved in deuterated benzene and added to a J-Young tap NMR tube containing $Cy_2NH.BH_3$. **First, the solution was stirred for 20 minutes at 60°C**. Upon addition of allylbenzene **3a**, the reaction vessel was removed from the glovebox and monitored in a Bruker 400 MHz spectrometer at 60 °C unless otherwise stated with measurements taken every 5 min. Data relevant for kinetic were analyzed using Microsoft Excel, standard errors were calculated based on the sum of squared residuals (SSR) derived from the best-fit curve and kinetic analysis was performed according to Variable Time Normalization Analysis (VTNA) method.[13,14]

Table S5. Relevant reactions for kinetic studies of Fe-catalyzed isomerization of **3a**

Experiment	[**3a**] (M)	[**2a**] (M)	$Cy_2NH{\cdot}BH_3$ (M)
1	0.9	0.043	0.043
2	**0.6**	0.043	0.043
3	0.9	**0.054**	**0.054**

Experiment **1** represents the standard profile for VTNA method. For the following VTNA plots, orders are determined by the best fit of the appropriate reaction sets against a normalized time axis.

Kinetic studies in positional isomerization

Time-shift analysis: to probe a catalyst deactivation or product inhibition over the course of isomerization of allylbenzene **3a**, time-shift analysis was performed by considering experiments **1** and **2**. After appropriate time-shifting of 450 min of the profile of the reaction **2**, a good graphical fit of both curves was observed. This good overlay over the entirely of the double-bond migration regime evidences the absence of catalyst deactivation or product inhibition processes.

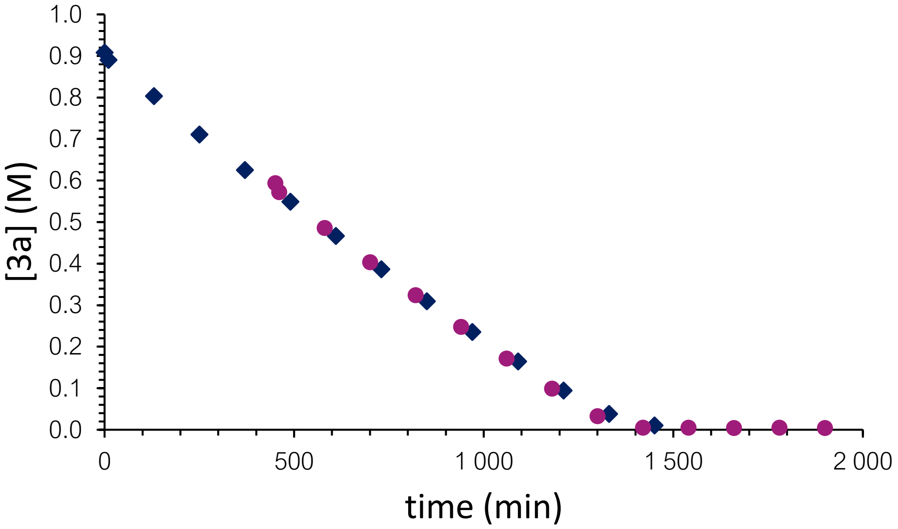

Figure S26. Time-shift analysis

Order in allylbenzene 3a: Considering experiments **1** and **2**, the best fit is obtained for an order in **[3a]** of 0.

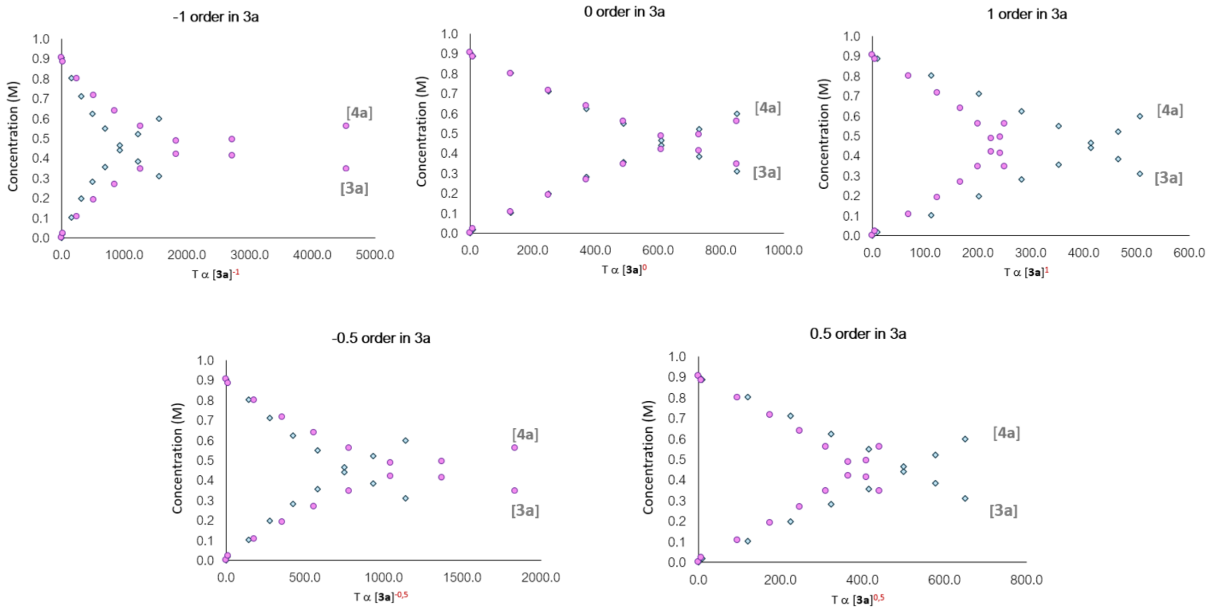

Figure S27. Variable Time Normalization Analysis to determine order in substrate **3a**

Order in complex 2a: Considering experiments **1** and **3**, the best fit was obtained for an order in **[2a]** of 1.2.

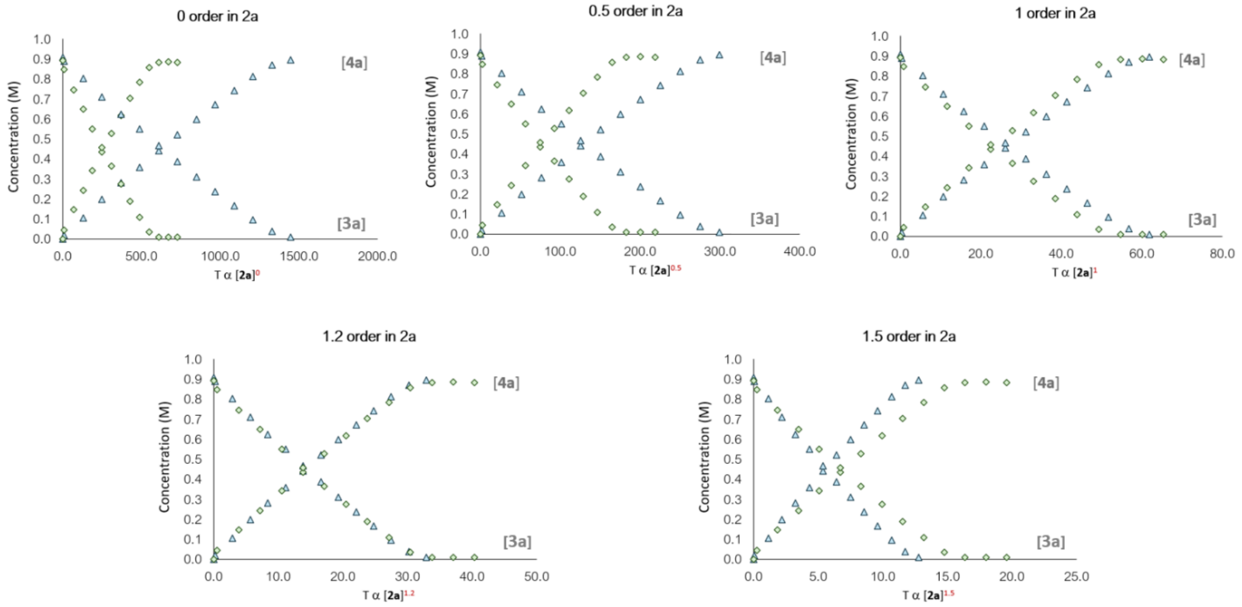

Figure S28. Variable time Normalization Analysis to determine order in catalyst **2a**

Determination of k_r: Having established the respective orders of the positional isomerization components, sequential normalization of the time scale in **3a** and catalyst **2a** leads to the overlay of all reaction profiles in a straight line with a slope that corresponds to k_r. The average value of k_r is 0.03 min^{-1}.

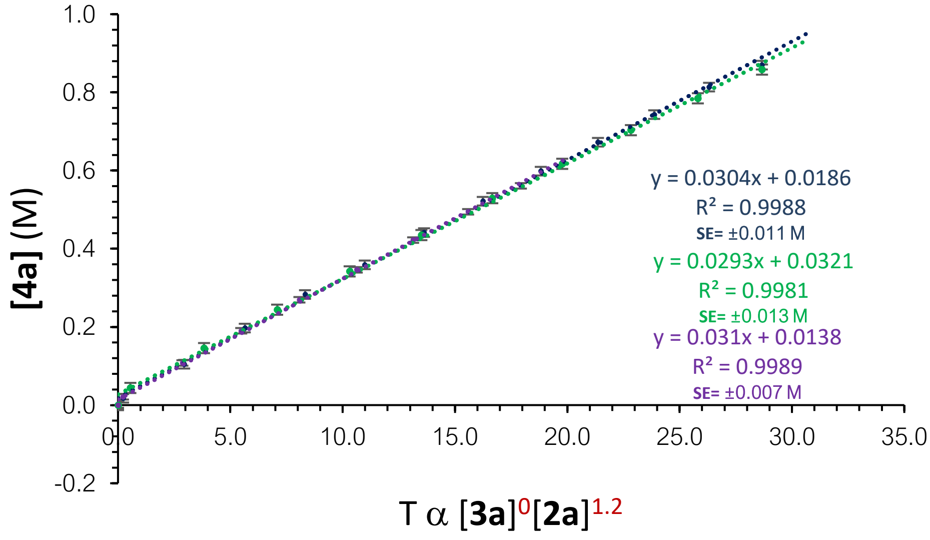

Figure S29. Time Normalized Analysis to determine k_r

Eyring plot: Experiments were performed as per the reaction setup in experiment 1 and monitored at stated temperature (50-70 °C). $\mathbf{k_r}$ (min^{-1}) were determined using Time Normalized analysis.

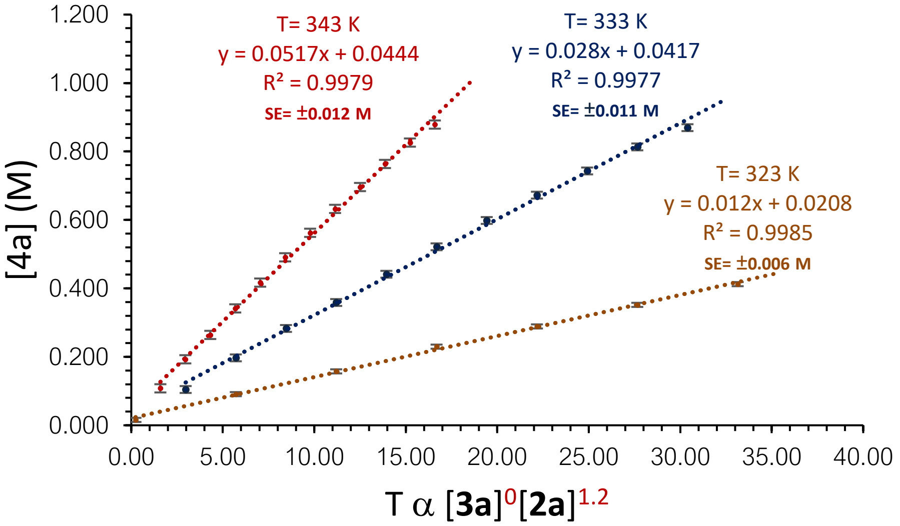

Figure S30. Time Normalized Analysis to determine $\mathbf{k_r}$ at different temperatures

**T (K)**	323	333	343
$\mathbf{k_r}$ **(min^{-1})**	0.012	0.030	0.052

The $\mathbf{k_r}$ (min^{-1}) values obtained in the experiments were used in Eyring plot to determine the activation parameters for positional isomerization of allylbenzene **3a** catalyzed by complex **2a**.

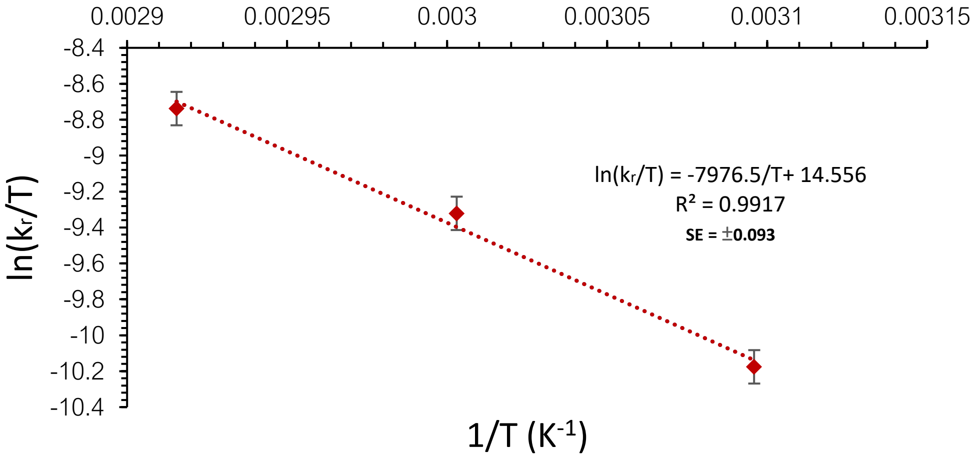

Figure S31. Eyring plot for Fe-catalyzed positional isomerization of **3a**

The sum of squared residuals (**SSR**) is 0.0087.

This leads to a standard error (**SE**) of ±0.093 with an Average percentage error of 0.5%

the Eyring equation is given by $\mathbf{ln(k_r/T) = -\Delta \mathit{H}^{\#}/R{*}T + \Delta \mathit{S}^{\#}/R + 23.76}$

The slope is $\mathbf{-\Delta \mathit{H}^{\#}/R}$ = -7976.5 K then $\Delta \mathbf{\mathit{H}}^{\#}$ = 15.85 kcal/mol

The Y-intercep is $\mathbf{\Delta \mathit{S}^{\#}/R}$ + 23.76 = 14.556 then $\Delta \mathbf{\mathit{S}}^{\#}$ = -18.29 cal/mol*K

Given $\mathbf{\Delta \mathit{G}^{\#} = \Delta \mathit{H}^{\#} - T\, \Delta \mathit{S}^{\#}}$ then $\Delta \mathbf{\mathit{G}}^{\#}_{333\ K}$ = 21.94 kcal/mol

Kinetic studies in geometrical isomerization:

The starting point of geometrical isomerization was identified as being the inflection point of the curve (*Z*)-**4a** = f(t), from which the sum of the concentration of (*E*)-**4a** and (*Z*)-**4a** was found to be constant along the reaction course of the magnitude of the initial concentration [**3a**]$_0$.
At this point:

1. The initial concentrations of (*E*)-**4a** and (*Z*)-**4a** are denoted [(*E*)-**4a**]$_0$ and [(*Z*)-**4a**]$_0$, respectively.
2. To ensure that variation of concentration of (*E*)-**4a** and (*Z*)-**4a** during geometrical isomerization is strictly associated to a *Z*-to-*E* interconversion process, it was carefully verified that the increase in the concentration of product (*E*)-**4a**, arises from the same order decrease in the concentration of (*Z*)-**4a**. Therefore**,** we define two new parameters ΔZ(t) = [(*Z*)-**4a**]$_t$ - [(*Z*)-**4a**]$_0$ and ΔE(t) = [(*E*)-**4a**]$_t$ - [(*E*)-**4a**]$_0$. Then along the course of geometrical isomerization ΔE(t) = -ΔZ(t)
3. In these following equations, for clarity, (*E*)-**4a** and (*Z*)-**4a** was quoted (*E*) and (*Z*) respectively

$$\frac{\mathbf{d}[(E)]_t}{\mathbf{d}\boldsymbol{t}} = -\frac{\mathbf{d}[(Z)]_t}{\mathbf{d}\boldsymbol{t}} = \mathbf{k'}_r[\mathbf{2a}]^{\boldsymbol{\alpha}}[(Z)]^{\boldsymbol{\beta}}$$

Given that [(*Z*)]$_0$ and [(*E*)]$_0$ are constant values, let:

$$\Delta Z(\mathrm{t}) = [(Z)]_t - [(Z)]_0 \qquad and \qquad \Delta E(\mathrm{t}) = [(E)]_t - [(E)]_0$$

Then

$$-\frac{\mathbf{d}\Delta Z(\mathrm{t})}{\mathbf{d}\boldsymbol{t}} = -\frac{\mathbf{d}([(Z)]_t - [(Z)]_0)}{\mathbf{d}\boldsymbol{t}} = -\frac{\mathbf{d}[(Z)]_t}{\mathbf{d}\boldsymbol{t}}$$

$$\frac{\mathbf{d}\Delta E(\mathrm{t})}{\mathbf{d}\boldsymbol{t}} = \frac{\mathbf{d}([(E)]_t - [(E)]_0)}{\mathbf{d}\boldsymbol{t}} = \frac{\mathbf{d}[(E)]_t}{\mathbf{d}\boldsymbol{t}}$$

Therefore, the rate law can be expressed by

$$\frac{\mathbf{d}\Delta E(\mathrm{t})}{\mathbf{d}\boldsymbol{t}} = -\frac{\mathbf{d}\Delta Z(\mathrm{t})}{\mathbf{d}\boldsymbol{t}} = \mathbf{k'}_r[\mathbf{2a}]^{\boldsymbol{\alpha}}[(Z)]^{\boldsymbol{\beta}}$$

Reaction profiles for VTNA could be confidently expressed by means of ΔZ(t) and ΔE(t) as function of the normalized time.

Order in (*Z*)-**4a:** Considering experiments **1** and **2**, the best fit is obtained for an order in **[**(*Z*)-**4a]** of 0.

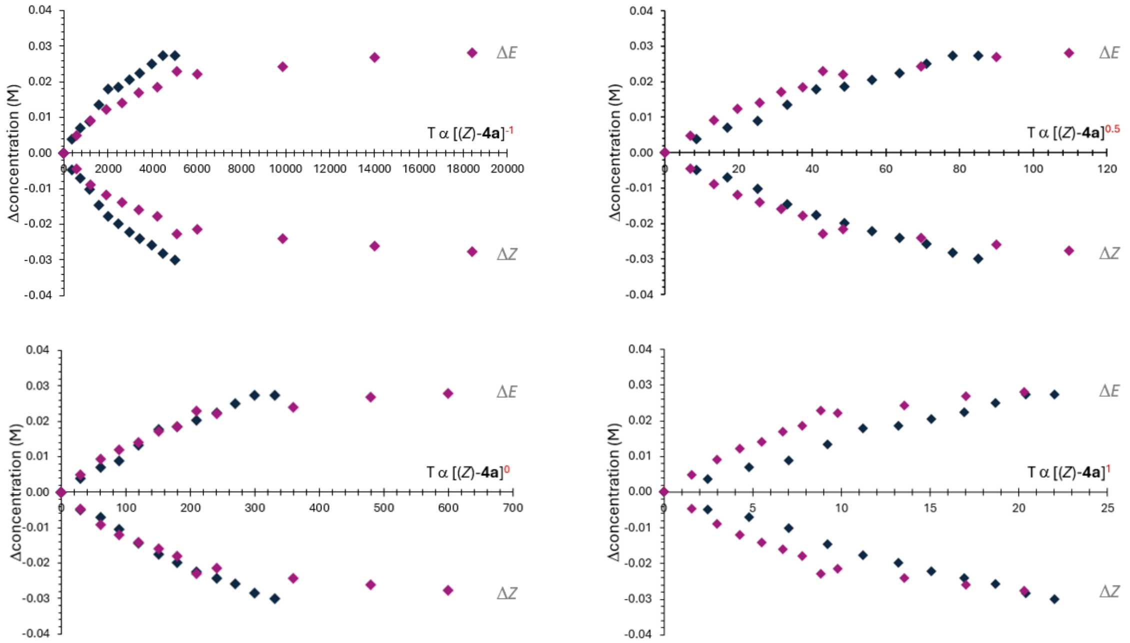

Figure S32. Variable Time Normalization Analysis to determine order in (*Z*)-**4a** in *Z*-to-*E* interconversion of **4a**

Order in 2a: Considering experiments **1** and **3**, the best fit is obtained for an order in **2a** of 1.1.

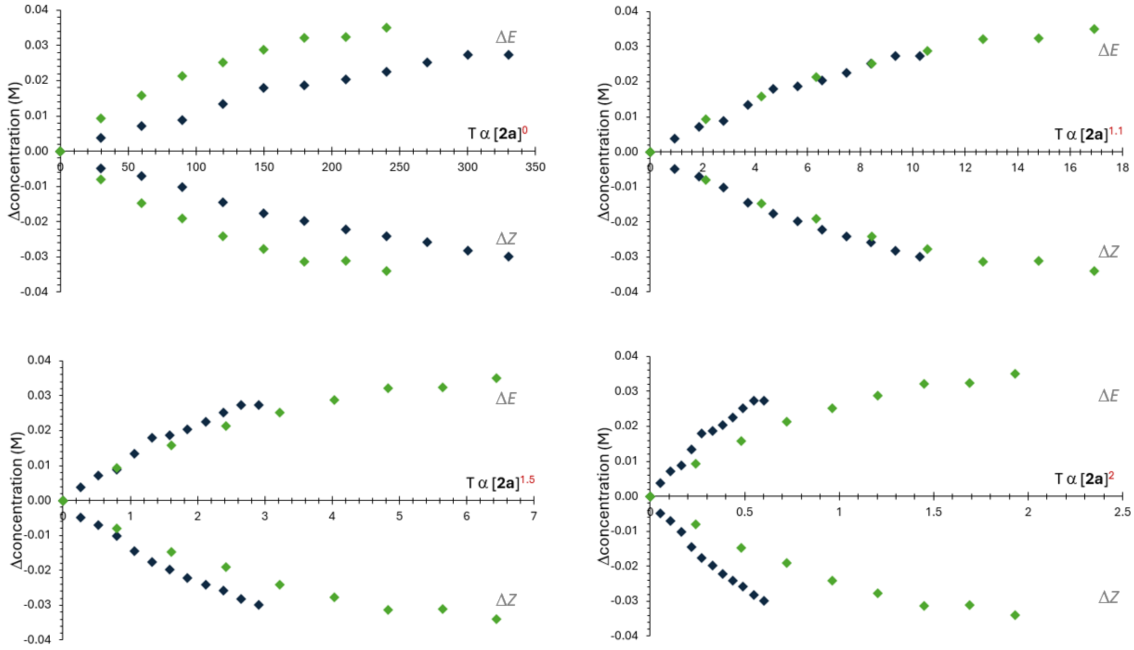

Figure S33. Variable Time Normalization Analysis to determine order in **2a** in *Z*-to-*E* interconversion of **4a**

Determination of k'$_r$: Having established the respective orders of the geometrical isomerization components, sequential normalization of the time scale in (*Z*)-**4a** and catalyst **2a** leads to the overlay of all reaction profiles in a straight line with a slope that corresponds to **k'$_r$**. The average value of **k'$_r$** is 0.003 min^{-1}.

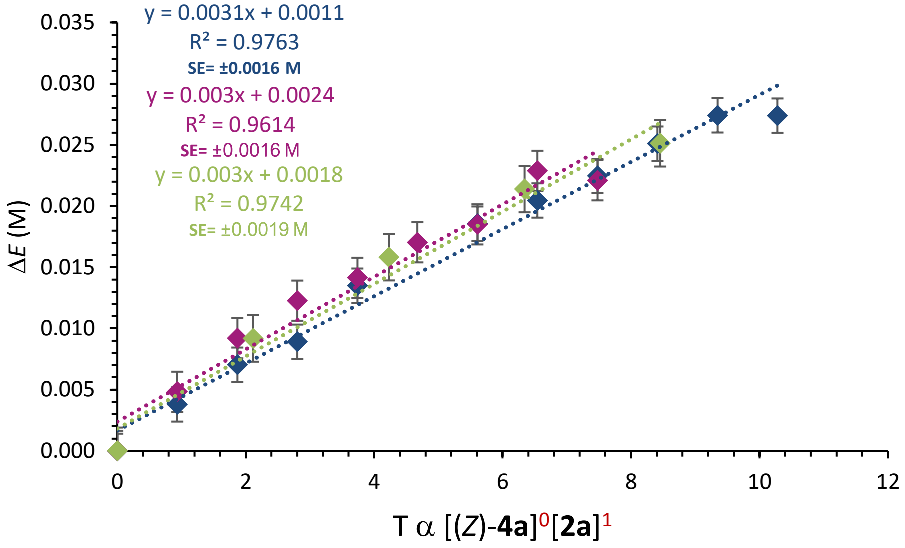

Figure S34. Time Normalized Analysis to determine **k'$_r$**

Eyring plot: Experiments were performed as per the reaction setup in experiment 1 and monitored at stated temperature (60-70 °C). **k'$_r$** (min^{-1}) were determined using Time Normalized analysis.

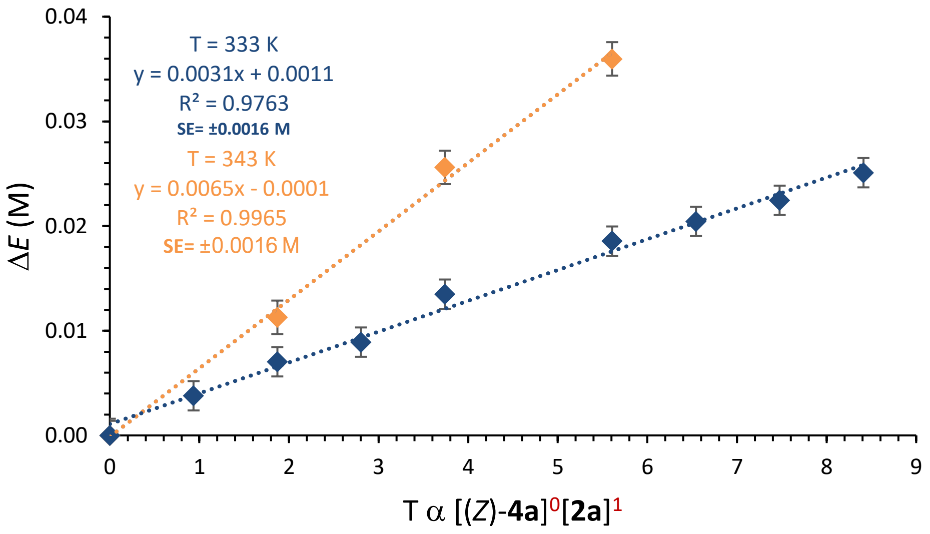

Figure S35. Time Normalized Analysis to determine **k'$_r$** at different temperatures

**T (K)**	333	343
**k'r (min^{-1})**	0.003	0.0065

The **k'$_r$** (min^{-1}) values obtained in the experiments were used to determine the activation parameters for *Z*-to-*E* interconversion of allylbenzene **4a** catalyzed by complex **2a**.

the Eyring equation is given by **ln(k'$_r$/T) = -Δ*H*$^{\#}$/R*T + Δ*S*$^{\#}$/R + 23.76**

-Δ*H*$^{\#}$/R = -8367.1 K   then **Δ*H*$^{\#}$** = 16.63 kcal/mol

Δ*S*$^{\#}$/R + 23.76 = 13.52   then **Δ*S*$^{\#}$** = -20.35 cal/mol*K

Given   **Δ*G*$^{\#}$ = Δ*H*$^{\#}$ - TΔ*S*$^{\#}$**   then **Δ*G*$^{\#}$$_{333\ K}$** = 23.40 kcal/mol

6h-Cyclic Voltammetry

Cyclic voltammetry (CV) measurements were recorded under an argon atmosphere at room temperature with a Metrohm Autolab PGSTAT320 potentiostat, using a standard 3-electrode setup fitted with Glassy Carbon working electrode (diameter = 0.3 cm), a platinum wire counter electrode and a 0.1 M Ag/AgCl electrode as reference. The working electrode was carefully polished with diamond paste (1 μm), sonicated with ethanol, washed with ethanol and acetone, and dried under vacuum overnight prior to use. [*n*-Bu_4N][PF_6] (Aldrich, >99% purity) was dried under vacuum prior to storage in an argon filled glovebox and used as supporting electrolyte at a concentration of 0.1 mM in THF. The scan rate is 100 mV/s.

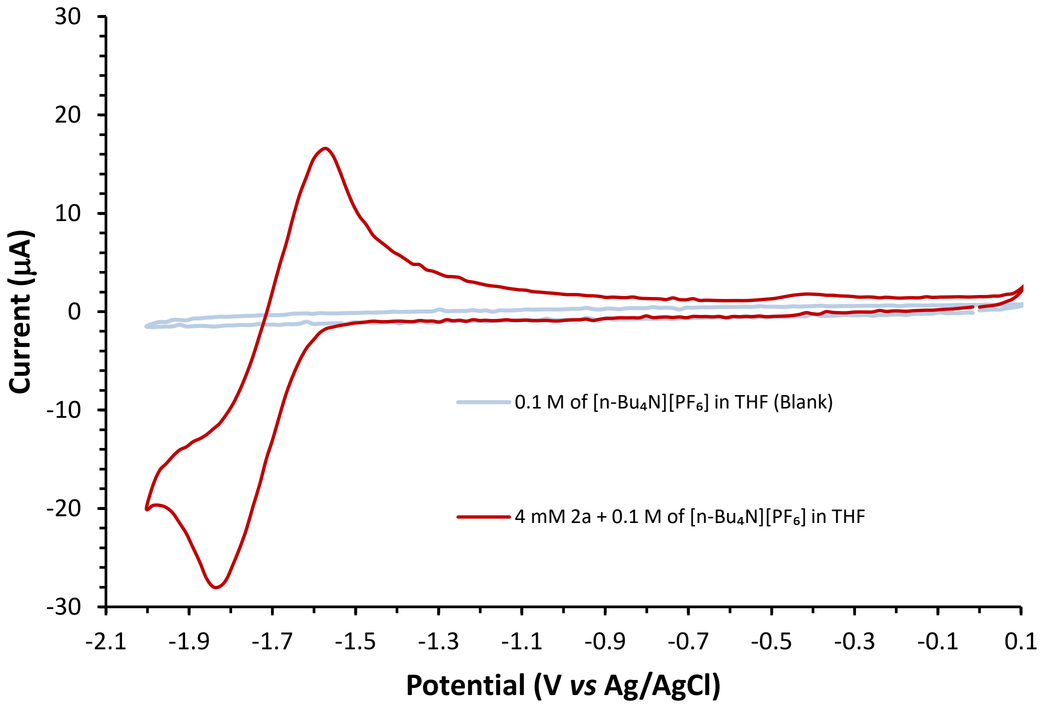

Figure S36. Cyclic voltammogram of 0.1 M [*n*-Bu_4N][PF_6] in THF (Blue curve), 4 mM **2a** + 0.1 M [*n*-Bu_4N][PF_6] in THF (red curve) (Potential are *vs* 0.1 M Ag/AgCl with a scan rate of 100 mV/s).

Cyclic voltammetry studies of **2a** (red curve) exhibit a sole quasi-reversible reduction/oxidation process at -1.70 V *vs* Ag/AgCl with a peak-to-peak separation of ΔE_p of 250 mV. The reduction wave appears at $E_{p,R}$ = -1.82 V *vs* Ag/AgCl associated with a re-oxidation wave at $E_{p,O}$ = -1.57 V *vs* Ag/AgCl. These features rule out an effective reduction of **2a** in the presence of co-catalytic amount of amine borane complex and align with our earlier results suggesting the reaction of **2a** with either $Me_2NH{\cdot}BH_3$ or $Cy_2NH{\cdot}BH_3$ is more likely to proceed via a redox neutral transformation leading to an iron (II) species rather than undergoing reduction to Fe(I) species.

6i-DFT calculations

Density functional theory calculations were carried out using the Gaussian 16.0 software package.[16] In line with a recent computational study dealing with low-valent Fe(II) chemistry,[17] geometry optimizations were performed using the BP86 density functional,[18, 19] with SDD basis sets for Fe atoms,[20] and double-ζ plus polarization 6-31G** for other atoms.[21, 22] The ultrafine integral grid option was employed in this purpose. Frequency calculations were undertaken at the same level of theory to furnish free energy. Ground states along the energy profiles have no negative frequencies. Transition states were confirmed to have one negative frequency that corresponded to the relevant bond formation/breaking process. Single point energies of optimized structures were obtained in benzene with Polarizable Continuum Model (PCM),[23] at the B3PW91-D3/TZVP level of theory.[24,25,26] $<S^2>$ eigenvalues have been carefully inspected at convergence to ensure no spin contamination. Minimum Energy Crossing Points (MECP) were calculated using easymecp package, which follows the method of Harvey et al.,[27] and reported as electronic energies with respect to the former ground states. All energies profiles are reported as free-energy in kcal/mol at the B3PW91-D3(C_6H_6)/TZVP//BP86/SDD/6-31G** level of theory, with respect to the pre-catalyst species **2a**, unless otherwise stated.

First, complex **2a** was computed in the singlet, triplet and quintet spin states. In line with solution magnetic data, **2a** in the quintet spin state was found to be more stable by 19.9 kcal/mol and 41.3 kcal/mol with respect to the triplet and singlet spin states respectively.

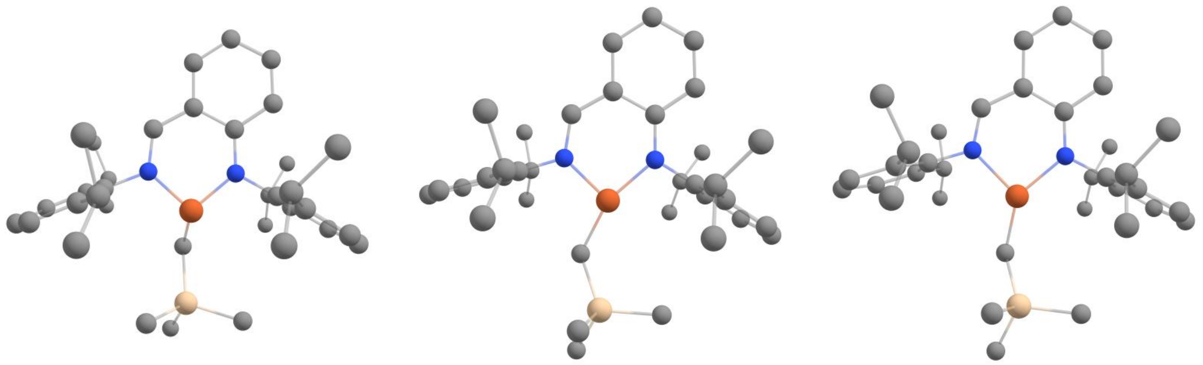

Figure S37. Computationally optimized structures for complex **2a** in the singlet, triplet and quintet spin states. Energies are in kcal mol^{-1} relative to ^{5}**2a** at the B3PW91-D3(C_6H_6)/TZVP//BP86/SDD/6-31G** level of theory. Hydrogen atoms bonded to carbons are omitted for clarity.

Comparison of relevant bond lengths and angles demonstrates that only calculated values from optimized structure of **2a** in the quintet spin state (denoted ^{5}**2a**) are in perfect accord with

experimental data. Quantitative evaluation of the structural deviation of optimized structures with respect to the XRD structure of **2a** is established by means of Root mean square deviation (RMSD). The calculated value for non-H ^{5}**2a** of 0.44 Å is reasonable, and such a deviation from the solid-state structure could arise from unconstrained structural optimization at 333 K.

Table S6. Calculated and experimentally determined bond distances and angles for complex **2a**

Element	Experimental value	Calculated value		
		^{1}**2a**	^{3}**2a**	^{5}**2a**
Fe-N_{imine}	2.027-2.018 Å	1.791 Å	1.868 Å	**2.004 Å**
Fe-N_{amido}	1.952-1.961 Å	1.792 Å	1.902 Å	**1.959 Å**
Fe-C	2.029 Å	1.935 Å	1.988 Å	**1.997 Å**
N_{imine}-Fe-N_{amido}	91.84°-92.30°	97.66°	94.10°	**93.60°**
RMSD	-	0.60 Å	0.40 Å	**0.44 Å**

The solution effective magnetic moment of complex of **[2b]$_2$** was 6.2 μ_B at 300K (in toluene-d_8), value which is lower than the expected spin-only value (μ_{eff} = 6.93 μ_B) for a dimer with two uncoupled, paramagnetic high-spin (S = 2) Fe(II) atoms.[28] This indicates the presence of antiferromagnetic coupling between the two nearby iron centers. The evaluation of the relative stability of complex **[2b]$_2$** at the triplet, quintet, septet and nonet spin states underlines the septet as the ground state, which is consistent with the measured magnetic susceptibility of complex **[2b]$_2$**. Optimized structures in the nonet, quintet and triplet spin states were respectively found to be +4.1 kcal/mol, +22.2 kcal/mol and +42.7 kcal/mol higher in energy. Root mean square deviation (RMSD) can be also calculated for computed non-H ^{7}**[2b]$_2$** structure for which a deviation of 0.34 Å indicates a satisfactory match of the calculated and solid-state structure.

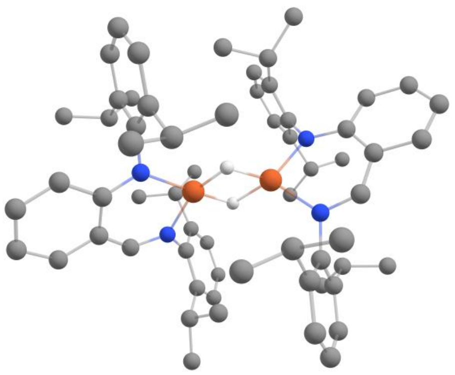

Figure S38. Computationally optimized structures for complex ^{7}**[2b]$_2$** in the septet spin states. Hydrogen atoms bonded to carbons are omitted for clarity.

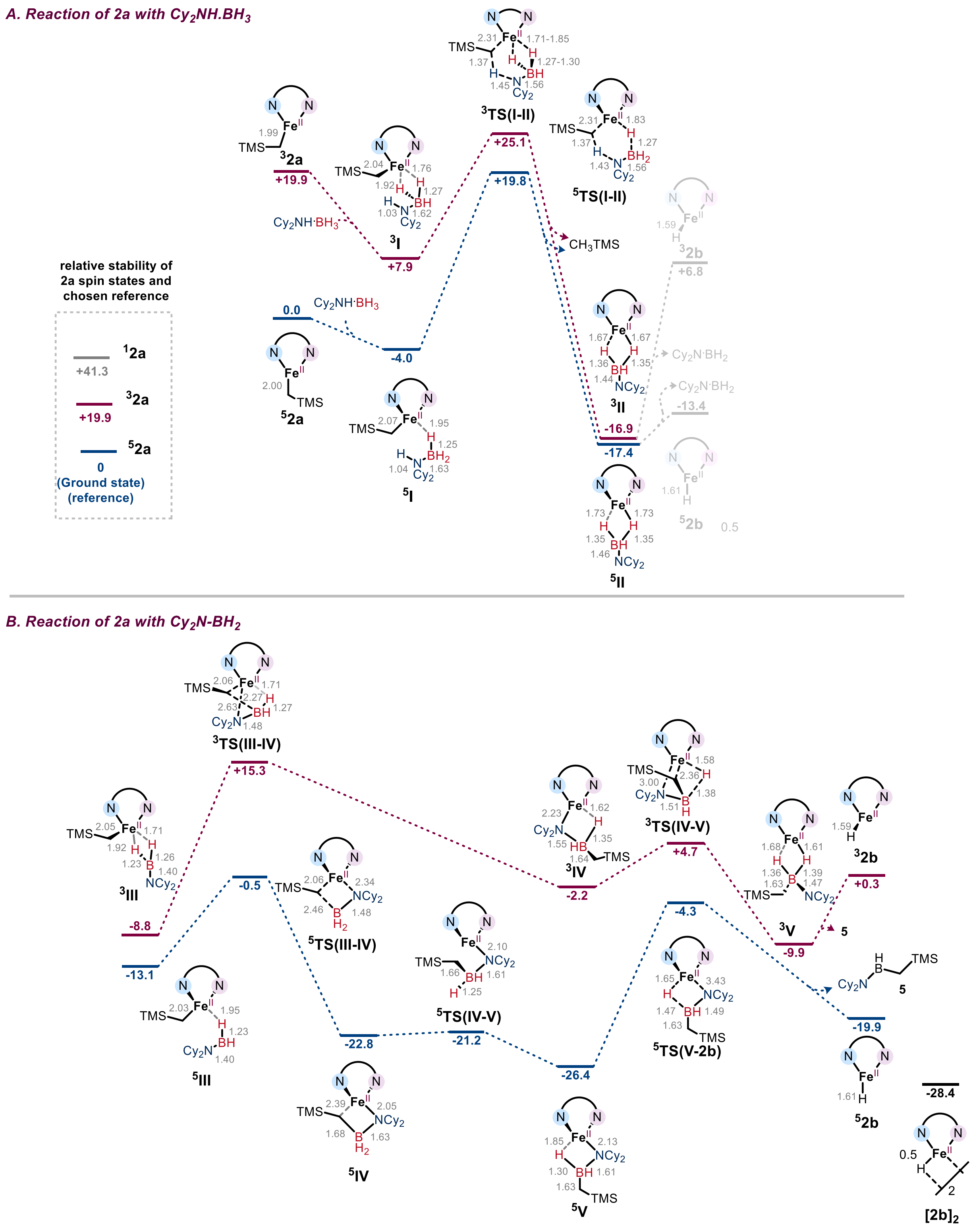

Figure S39. Calculated Gibbs energy profiles for the minor pathway of [**2b**]$_2$ formation from the reaction of **2a** and amine borane $Cy_2NH{\cdot}BH_3$ (A) and for the major pathway of [**2b**]$_2$ formation from the reaction of **2a** and amino-borane $Cy_2N\text{-}BH_2$ (B). Energies are in kcal mol^{-1} at the B3PW91-D3(C_6H_6)/TZVP//BP86/SDD/6-31G** level of theory and relative to ^{5}**2a**.

In both pathways, the quintet state was found to prevail energetically throughout the reaction profiles. For the minor pathway of **[2b]$_2$** formation (Figure S39, A), initial coordination of $Cy_2NH·BH_3$ to **^{5}2a** generates four-coordinate σ-adduct **^{5}I** and is exergonic by 4 kcal mol^{-1} on the more accessible quintet surface. This results in a slight increase in Fe-C and B-H bond lengths to 2.07 Å and 1.25 Å respectively (in comparison to 2.00 Å and 1.22 Å for **^{5}2a**), while the B-N bond length decreases by 0.04 Å. At this stage, **^{5}I** is subjected to protonolysis of its polar Fe−C bond with concomitant hydride transfer onto Fe center. This step is rate-determining and irreversible and occurs via **^{5}TS (I−II)** with a relatively high, but accessible, energy barrier of 23.8 kcal mol^{-1} leading to the formation of four-coordinate adduct **^{5}II** and release of CH_3TMS. This adduct has a relative free energy of -17.4 kcal mol^{-1} and features a B−H σ-interaction with coordinated imine borane Cy_2NBH_2. Subsequent endergonic dissociation of imine-borane affords monomeric Fe(II)-H species **^{5}2b** that lies at a -13.4 kcal mol^{-1} Gibbs free energy (from **^{5}2a**). Then, this monomer undergoes thermodynamically favourable dimerization into **[2b]$_2$,** as this step is downhill by 8.5 kcal mol^{-1}. The thermodynamic prevalence of **[2b]$_2$** over its monomer might account for the retention of dimeric nuclearity of isolated **[2b]$_2$** in solution observed experimentally. Regarding the main pathway of **[2b]$_2$** formation from **2a** and in situ generated Cy_2NBH_2[86] (Figure 39, B. .), the amino-borane initially interacts with **^{5}2a** via σ-bonding to give intermediate **^{5}III** that lies at a relative Gibbs energy of -13.1 kcal mol^{-1} along the lowest-lying energy profile. Regarding the main pathway of **[2b]$_2$** formation from **2a** and in situ generated Cy_2NBH_2[86] (Figure 3, A.), the amino-borane initially interacts with **^{5}2a** via σ-bonding to give intermediate **^{5}III** that lies at a relative Gibbs energy of -13.1 kcal mol^{-1} along the lowest-lying energy profile. Interestingly, a subsequent alkyl migration from Fe to B is operating being kinetically accessible on the quintet surface with a modest energy barrier of 12.6 kcal mol^{-1} via **^{5}TS (III−IV)**. The latter decays into amidoborate adduct ^{5}**IV** (ΔG =-22.8 kcal mol^{-1}) that features an agostic interaction with B-C bond. Subsequent exergonic (ΔG = 3 .6 kcal mol^{-1}) and almost barrierless ($\Delta G^{‡}$ = 1.6 kcal mol^{-1}) rotation across the B-N of ^{5}**IV** delivers ^{5}**V**. Thereafter, ^{5}**V** undergoes a reversible, rate-limiting β-hydride transfer from B to Fe through **^{5}TS(V−2b)** and with an energy barrier of 22.1 kcal mol^{-1}. This step leads to the formation of **^{5}2b** and **5**. Then, it is followed by downhill dimerization of **^{5}2b**. These DFT calculations stressed, among the two pathways for **[2b]$_2$** formation, the one arising from the reaction of **2a** and amino-borane is predicted to be faster as a result of the assessed kinetic gap between the two rate-determining steps of these pathways, namely the **^{5}I →**^{5}**II** protonolysis step (Figure S34) and the **^{5}V→**^{5}**2b** β-H transfer step ($\Delta\Delta G^{‡}$ = 1.7 kcal mol^{-1}). This is consistent with the experimental observation of this pathway being predominant in the stoichiometric reactivity studies of **2a** and the ΔΔG‡ value difference is in accordance with the experimentally observed 1:12 ratio of CH_3SiMe_3 and **5** by ^{1}H NMR experiments.

Reaction of 2b with $Cy_2NH{\cdot}BH_3$

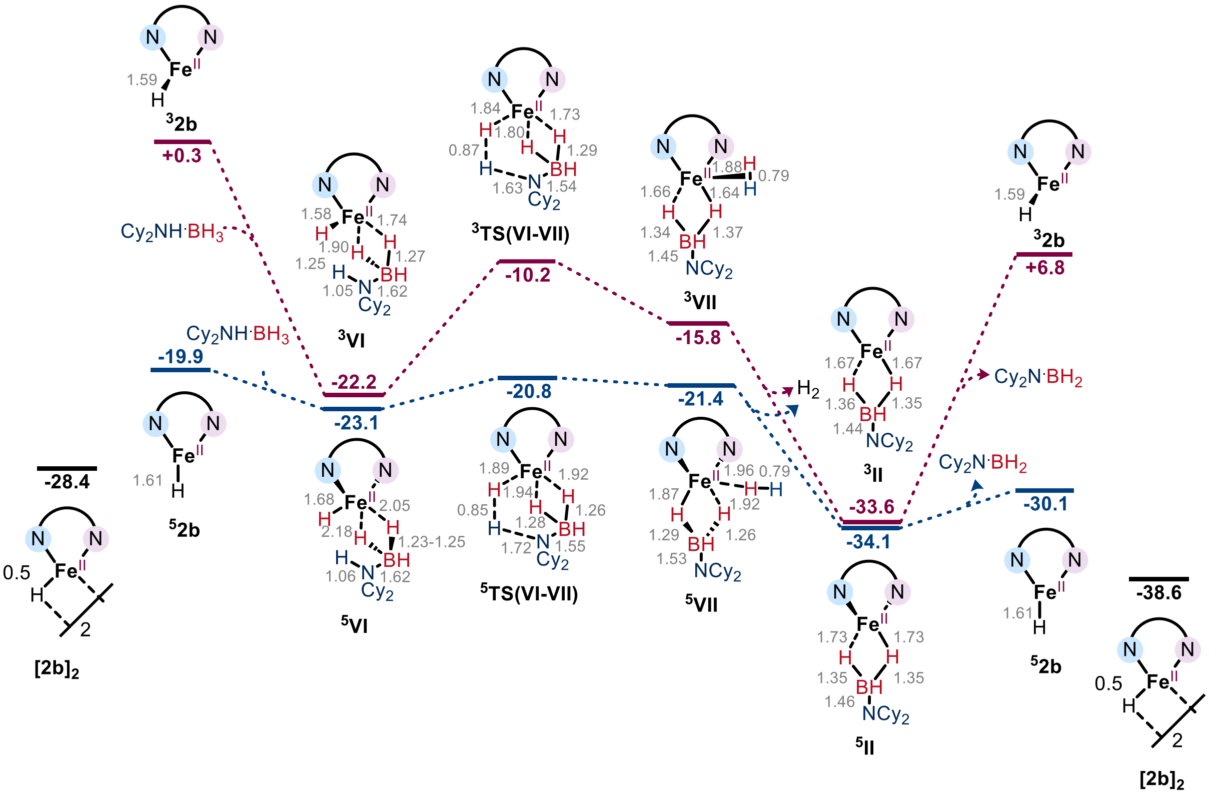

Figure S40. Calculated Gibbs energy profiles for the concurrent Fe-H mediated dehydro-coupling of $Cy_2NH{\cdot}BH_3$. Energies are in kcal mol^{-1} at the B3PW91-D3(C_6H_6)/TZVP//BP86/SDD/6-31G** level of theory

Stoichiometric reaction of **2a** with $Cy_2NH{\cdot}BH_3$ is further complemented by a concurrent Fe-H mediated dehydro-coupling of $Cy_2NH{\cdot}BH_3$ (Figure S40). This transformation proceeds along the quintet spin state surface and results in the formation $Cy_2N{\cdot}BH_2$ with concomitant hydrogen release. Starting from monomeric **2b**, coordination of $Cy_2NH{\cdot}BH_3$ is energetically downhill by 3.2 kcal/mol and generates a pentacoordinate Fe(II) σ–adduct (⁵**VI**, -23.1 kcal/mol). This results in a slight increase in Fe-H and B-H bond lengths to 1.68 Å and 1.25 Å respectively (in comparison to 1.61 Å and 1.22 Å for ⁵**2a**). Protonolysis of polar Fe-H bond with concomitant hydride transfer from B to Fe-center occurs via the ⁵**TS(VI-VII)** with an energy barrier of 2.3 kcal/mol. This barrierless transitions state, in contrast to its equivalent from **2a**, indicates that dehydro-coupling of $Cy_2NH{\cdot}BH_3$ is facilitated by the involvement of **2b**. Subsequent optimized intermediates lies at -21.4 kcal/mol and features σ–interaction with dihydrogen molecule. The release of this latter is exergonic by 12.7 kcal/mol and results in intermediates ⁵**II** at -34.1 kcal/mol. Finally, decoordination of $Cy_2N{\cdot}BH_2$ followed by dimerization delivers **[2b]₂** at -38.6 kcal/mol.

The positional isomerization of 3a from $[2b]_2$: stereoselective-determining β-H elimination

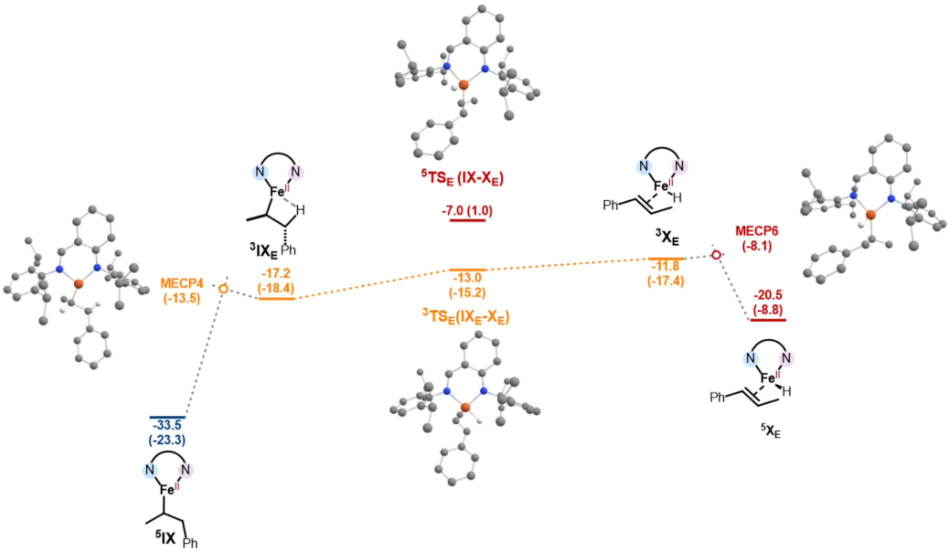

Figure S41. Calculated Gibbs energy profiles for the (*E*)-selective β-hydride elimination. Transitions states and MECPs optimized structures are represented. Free energies are in kcal mol^{-1} at the B3PW91-D3(C_6H_6)/TZVP//BP86/SDD/6-31G** level of theory. MECPs electronic energies are in kcal mol^{-1} at the BP86/SDD/6-31G**.

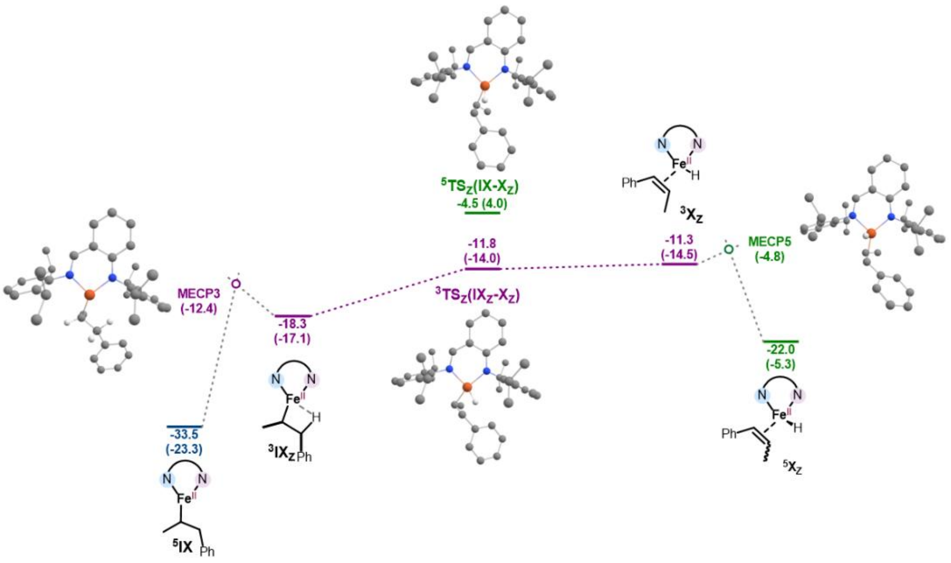

Figure S42. Calculated Gibbs energy profiles for the (*Z*)-selective β-hydride elimination. Transitions states and MECPs optimized structures are represented. Free energies are in kcal mol^{-1} at the B3PW91-D3(C_6H_6)/TZVP//BP86/SDD/6-31G** level of theory. MECPs electronic energies are in kcal mol^{-1} at the BP86/SDD/6-31G**.

Alternative profile for the positional isomerization of 3a from [2b]$_2$

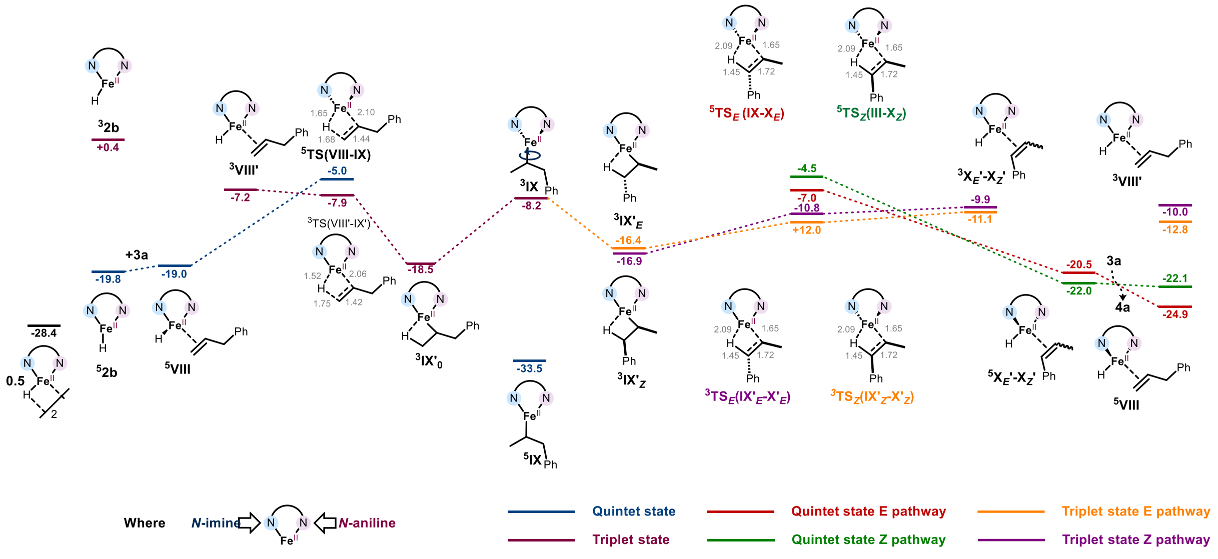

Figure S43. Calculated Gibbs energy profiles for the positional isomerization of **3a** from **[2b]$_2$**. Energies are in kcal mol^{-1} at the B3PW91-D3(C_6H_6)/TZVP//BP86/SDD/6-31G** level of theory.

In the manuscript, the postulated Gibbs energy profile of the positional isomerization of **3a** from **[2b]$_2$** displays alkene coordination trans to the **^{3}Fe-N$_{amido}$** bond at the triplet surface. An alternative energy profile with alkene coordination trans to the **^{3}Fe-N$_{imine}$** may be accessible, albeit transition state energies are, overall, somewhat superior in energy by 1 to 1,5 kcal/mol (Figure S43). No additional efforts have been made to locate the MECPs along the alternative energy profile, although they might reasonably be expected to resemble the geometric features of those in the ascendant one.

The geometric isomerization of (*Z*)-4a from [2b]$_2$

To gain a further insight into the origin of the stereoselectivity arising from geometrical isomerization, DFT calculations were undertaken to model the *Z*-to-*E* interconversion **4a** from **[2b]$_2$**. Deuteration experiments demonstrates that the degree of incorporation of D into C_1 and C_2 at the initial stage (0.5 h of reaction) of the *E*-to-*Z* interconversion of (*E*)-**4a** catalyzed by **2a** / $Cy_2NH{\cdot}BD_3$ (5 mol%) depends on the stereoisomer formed. This suggests the *E*:*Z* selectivity of the subsequent β-H elimination step is correlated to the regioselectivity of the hydrometallation. On this basis, we postulate that migratory insertion of (*Z*)-**4a** into Fe-H results in either intermediate **^{5}IX** or **^{5}XII** (Figures S44-S45). **^{5}IX** lies at +4.3 kcal/mol above **^{5}XII** and proceeds into (*E*)-**4a** with an energy barrier of 20.5 kcal/mol. In line with experimental results, we can assume that the **^{5}IX** mediated route is less *E*-selective. β-H elimination from the more stable **^{5}XII** is kinetically less accessible by 24.4 kcal/mol and lead to a thermodynamic

distribution with an *E*:*Z* of up to 68:1. Although its reactivity is somewhat hindered, ^{5}**XII** is more *E*-selective and contributes to the increase in the *E*:*Z* ratio.

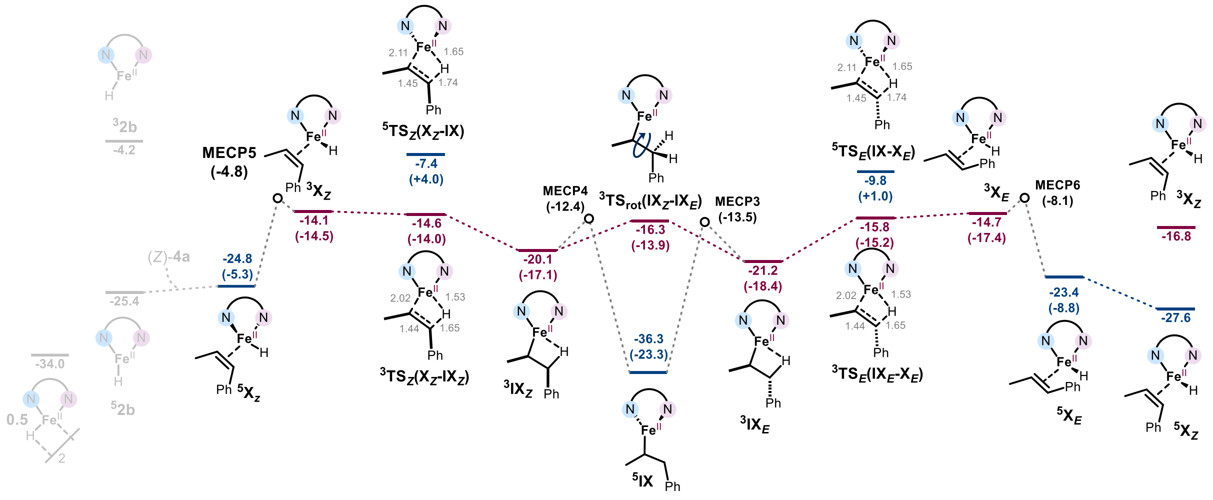

Figure S44. Calculated Gibbs energy profiles for the ^{5}**IX** mediated geometrical isomerization of (*Z*)-**4a**. Energies are in kcal mol^{-1} at the B3PW91-D3(C_6H_6)/TZVP//BP86/SDD/6-31G** level of theory and relative to ^{5}**2a**. Electronic energies are reported in parenthesis relative to ^{5}**2a**. MECPs were identified using Harvey *et al.* method and are expressed by means of electronic energy relative to ^{5}**2a**.

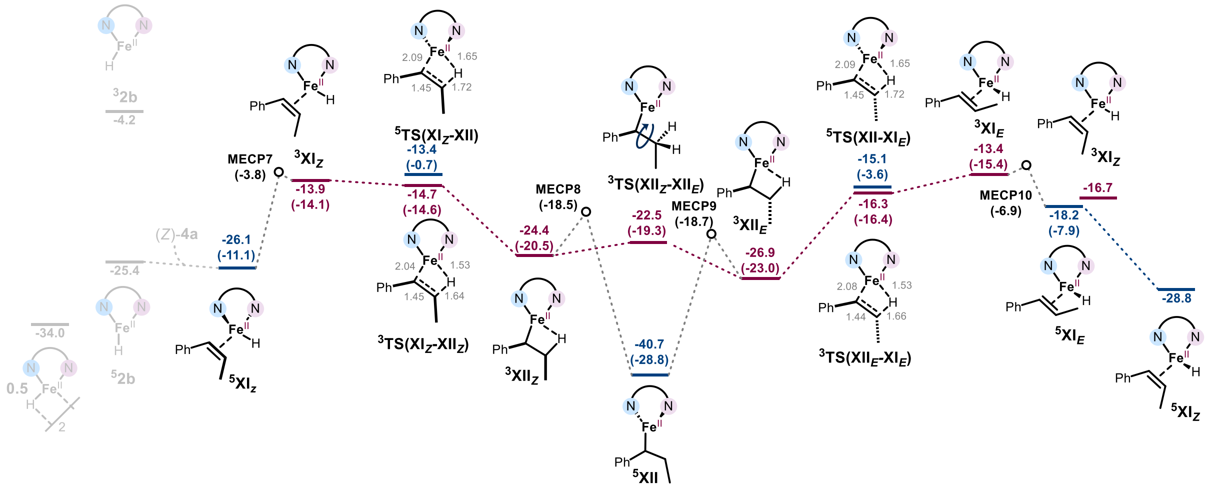

Figure S45. Calculated Gibbs energy profiles for the ^{5}**XII** mediated geometrical isomerization of (*Z*)-**4a**. Energies are in kcal mol^{-1} at the B3PW91-D3(C_6H_6)/TZVP//BP86/SDD/6-31G** level of theory and relative to ^{5}**2a**. Electronic energies are reported in parenthesis relative to ^{5}**2a**. MECPs were identified using Harvey *et al.* method and are expressed by means of electronic energy relative to ^{5}**2a**.

6i- Mechanistic insights on (^{Me}BDI^{iPr})Fe(II)-catalyzed isomerization of allylbenzene 3a

Catalytic reactivity of (^{Me}BDI^{iPr})Fe-CH$_2$TMS (C) with allylbenzene 3a under optimized conditions

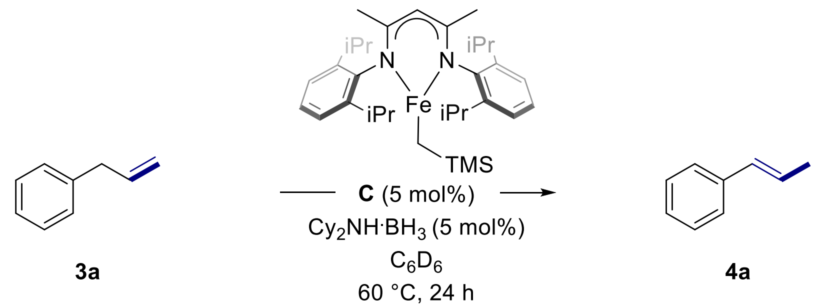

In an argon-filled glovebox, **(^{Me}BDI^{iPr})Fe-CH$_2$TMS** (14.1 mg, 0.025 mmol) was dissolved in deuterated benzene and added to an oven-dried 10 mL screw-capped tube, equipped with a magnetic stir bar. Next, allylbenzene **3a** (59.1 mg, 0.5 mmol) was added at room temperature. Then, the reaction was stirred at 60 °C for 24 h. Upon completion, the reaction mixture was exposed to air and 1,3,5-trimethoxybenzene (42 mg, 0.25 mmol, 0.5 equiv.) was added to the reaction vessel. ^{1}H NMR analysis using 1,3,5-trimethoxybenzene as internal standard revealed **10% conv., 8% yield** of prop-1-en-1-ylbenzene **4a**.

Stoichiometric reactivity of $[(^{Me}BDI^{iPr})Fe\text{-}H]_2$ with allylbenzene 3a:

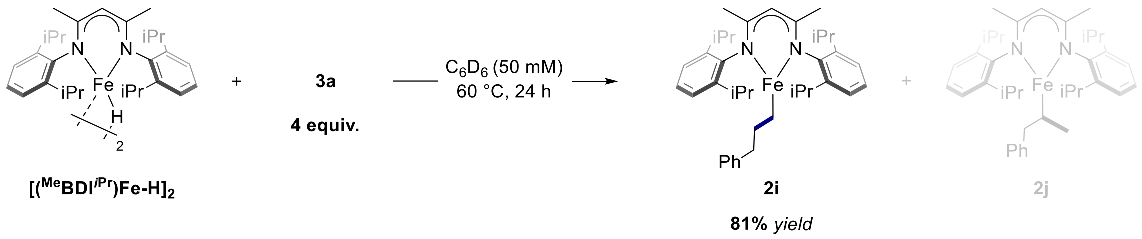

In a argon filled glovebox, allylbenzene (59.1 mg, 0.5 mmol, 4 equiv.) was added to a stirred solution of $[(^{Me}BDI^{iPr})Fe\text{-}H]_2$ (119 mg, 0.125 mmol, 1 equiv.) in benzene (5 mL) at room temperature. The mixture was allowed to warm up to 60 °C and stirred for 24. Then, the solution was concentrated under reduced pressure until dryness. The solid was dissolved in pentane and cooled to -20°C to afford the complex **2i** as a yellow crystalline solid (120 mg, 0.20 mmol, 81%). **^{1}H NMR** (400 MHz, C_6D_6, RT) δ 123.6 (br s, 1H, α-C*H*), 94.1 (br s, 2H, γ-CH_2), 65.8 (s, 6H, 2 x *Me-backbone*), 41.1 (s, 2H, Ph-*o* or *m*-C*H*), 18.3 (s, 2H, Ph-*o* or *m*-C*H*), 13.7 (s, 1H, Ph-*p*-C*H*), -10.2 (s, 4H, *m-H Ar*), -18.3 (s, 12H, (*Me*)$_2$CH), -73.2 (s, 2H, *p-H Ar*), -114.1 (br s, 12H, (*Me*)$_2$CH), -119.6 (br s, 4H, (Me)$_2$C*H*). **Evans μ_{eff}** (Toluene-d_8, 300 K) 5.4 μ_B.

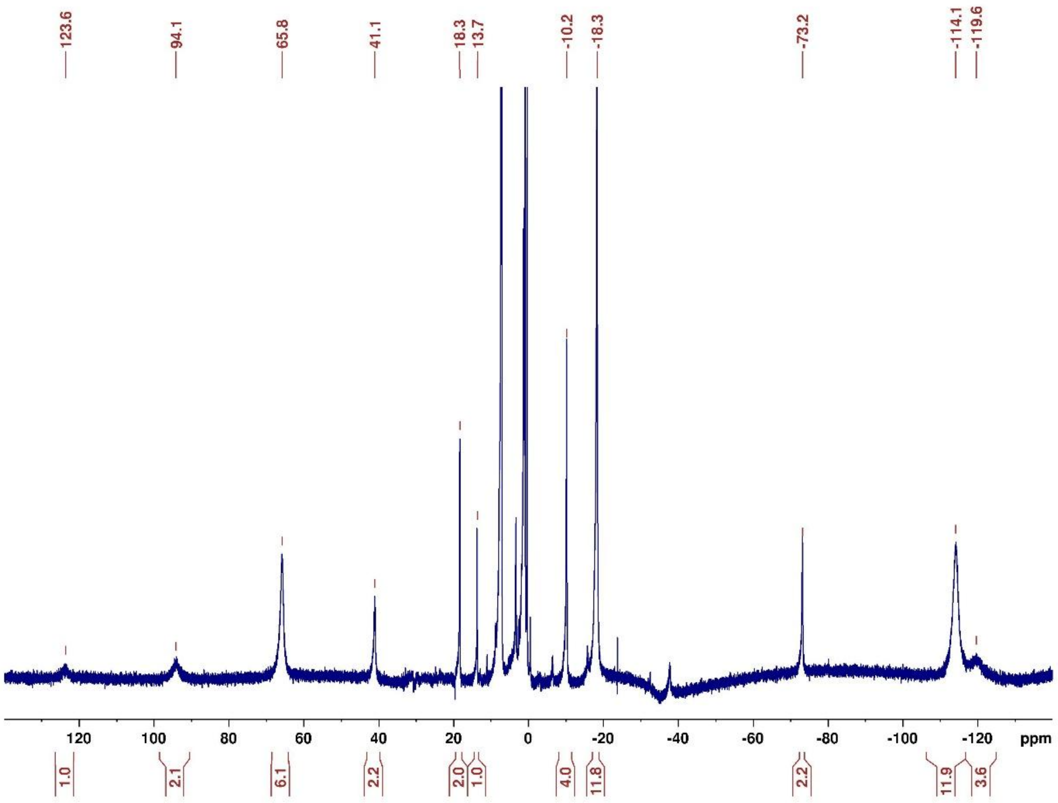

Figure S46. ^{1}H NMR spectrum of complex **2i** (400 MHz, C_6D_6, RT)

Probing alkyl migration in 2i:

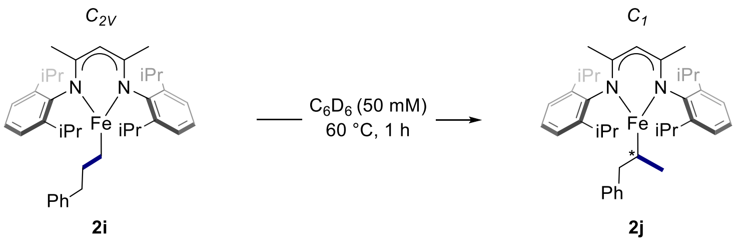

In an argon-filled glovebox, **2i** (14.8 mg, 0.025 mmol) was dissolved in deuterated benzene (0.5 mL) and added to a J-Young tap NMR tube. The reaction vessel was then removed from the glovebox and transferred to a Bruker 400 MHz spectrometer preheated to 60 °C. The spectra were recorded at 0 and 60 minutes of heating, within a spectral width ranging from 120 to -120 ppm.

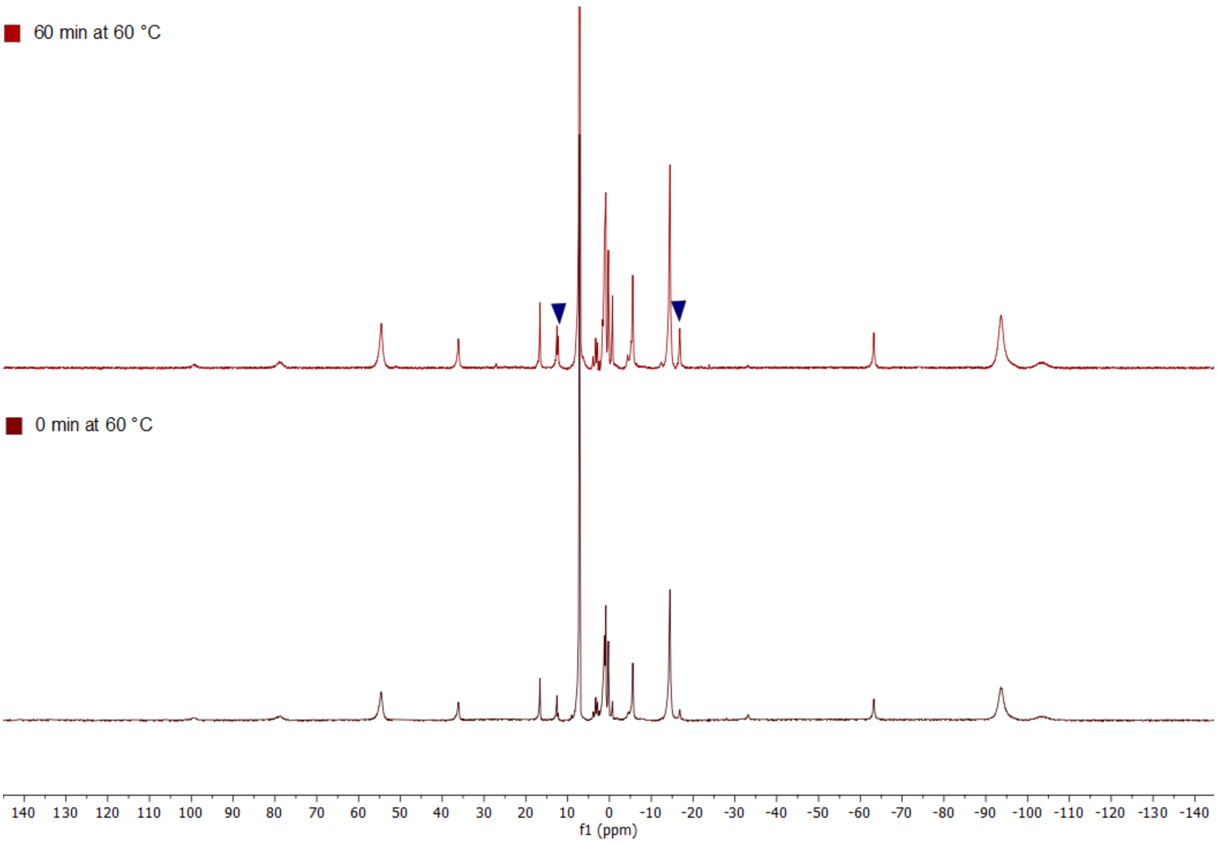

Figure S47. ^{1}H NMR trace of **2i** at 0 min (**bottom**) and 60 min (**top**) at 60 °C. The newly observed signals at 12.3 and -16.8 ppm are indicated by a blue dot.

Interpretation: While **2i** exhibits C_{2V} symmetry, the branched complex **2j**, relevant to catalysis, lacks symmetry due to the stereogenic carbon (*) of the alkyl ligand. As a result, signals from the two chemically and magnetically inequivalent halves of the diketiminate ligand in **2j** split into two distinct ones. In this study, while new minor signals appear at 12.3 and -16.8 ppm, chemical shifts of all signals

of interest remained unchanged and no significant signal splitting occurred after 1 h at 60 °C. This indicates that alkyl migration in **2i** is a demanding/slow process.

Identification of the resting state in [(^{Me}BDI^{iPr})Fe-H]$_2$ catalyzed isomerization of allylbenzene 3a:

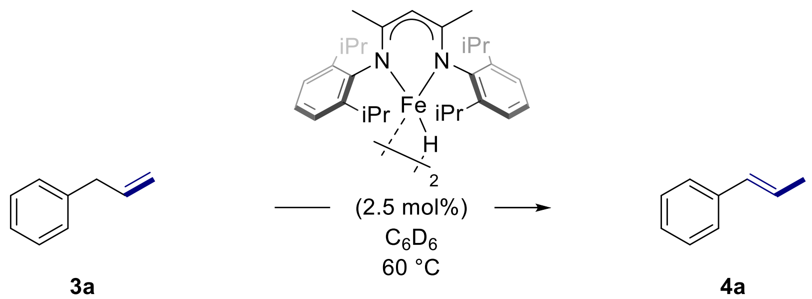

3a **4a**

In an argon-filled glovebox, **[(^{Me}BDI^{iPr})Fe-H]$_2$** (15.5 mg, 0.0125 mmol) was dissolved in deuterated benzene and added to a J-Young tap NMR tube. Next, allylbenzene **3a** (59.1 mg, 0.5 mmol) was added at room temperature. The reaction vessel was then removed from the glovebox and transferred to a Bruker 400 MHz spectrometer preheated to 60 °C. The spectra were recorded at 60 °C within a spectral width ranging from 120 to -120 ppm every 5 min for 30 min then every 10 min up to 1 h.

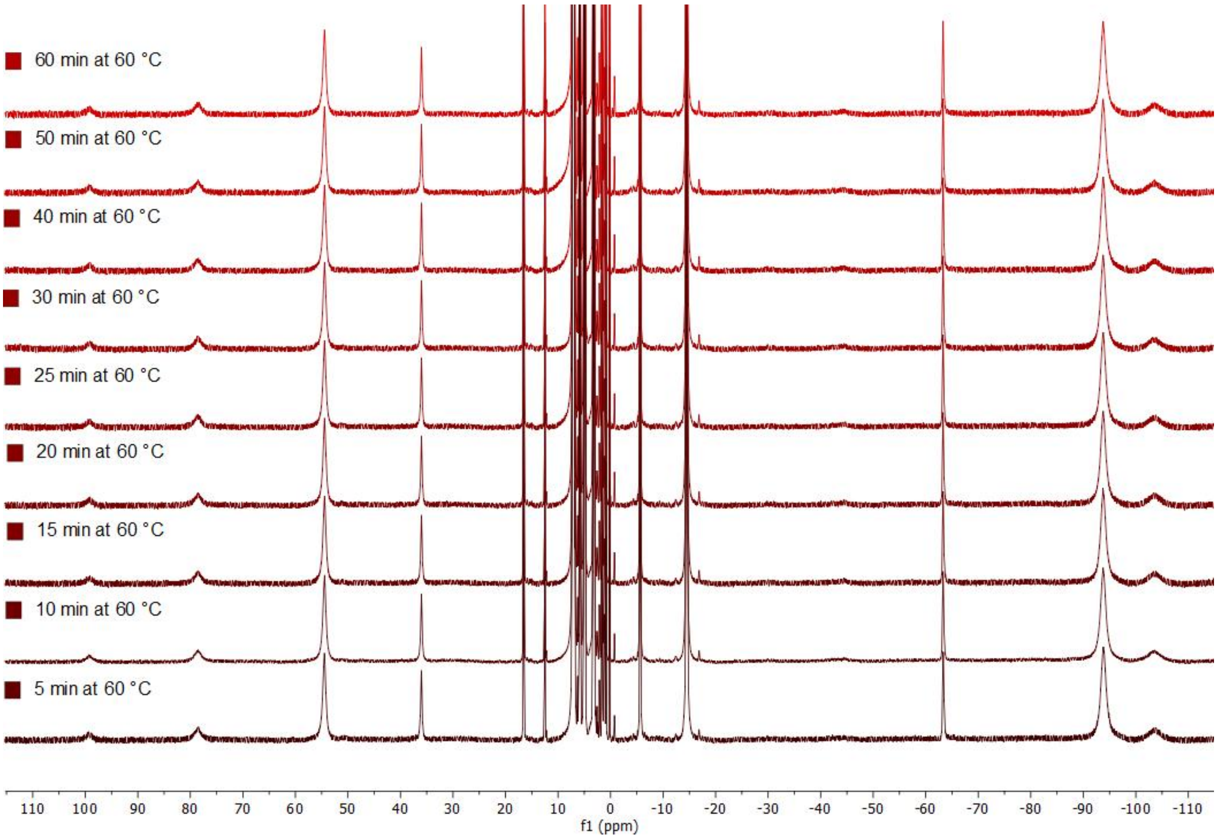

Figure S48. Wide-sweep ^{1}H NMR trace of **[(^{Me}BDI^{iPr})Fe-H]$_2$** catalyzed isomerization of allylbenzene **3a** (40 equiv.).

Interpretation: The paramagnetic species signals detected along the 1-h measurement perfectly match those of **2i** indicating that this latter likely represents the catalyst resting state along the **[(^{Me}BDI^{iPr})Fe-H]$_2$**-catalyzed isomerization of allylbenzene **3a**.

DFT Calculations for 2,1- and 1,2-insertion step of 3a into [(^{Me}BDI^{iPr})Fe-H]$_2$

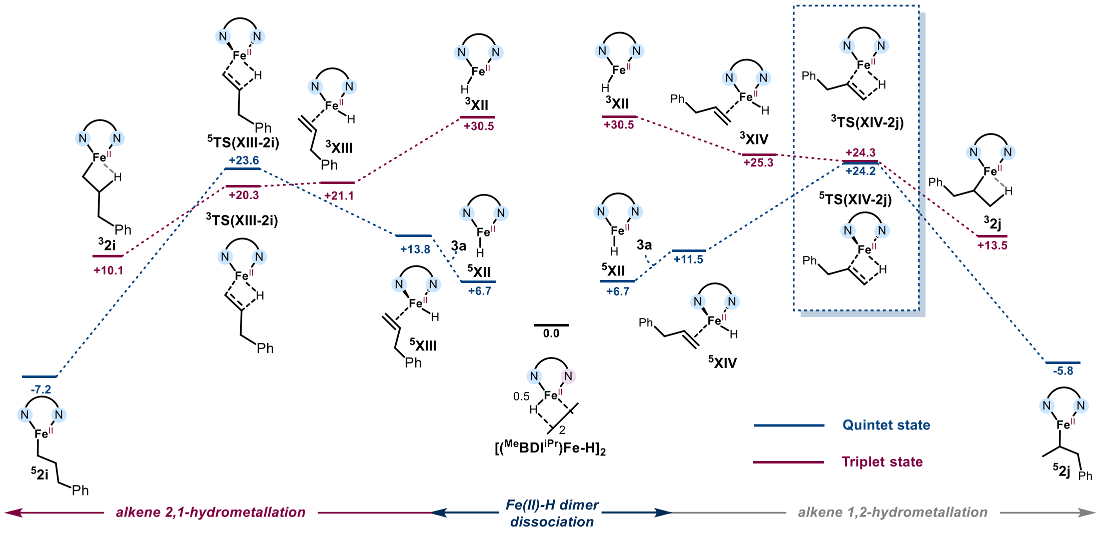

Figure S49. Calculated Gibbs energy profiles for the *2,1-* and *1,2-*insertion step of **3a** from **[(^{Me}BDI^{iPr})Fe-H]$_2$**. Energies are in kcal mol^{-1} at the B3PW91-D3(C_6H_6)/TZVP//BP86/SDD/6-31G** level of theory.

DFT Calculations for [(^{Me}BDI^{iPr})Fe-H]$_2$ catalyzed isomerization of allylbenzene 3a

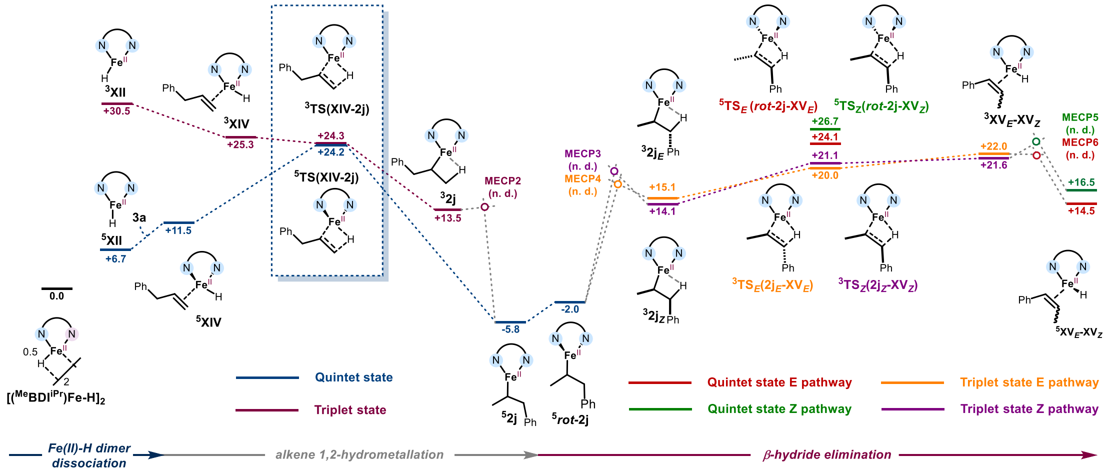

Figure S50. Calculated Gibbs energy profiles for the **[(^{Me}BDI^{iPr})Fe-H]$_2$** catalyzed isomerization of allylbenzene **3a**. Energies are in kcal mol^{-1} at the B3PW91-D3(C_6H_6)/TZVP//BP86/SDD/6-31G** level of theory. MECPs were identified using Harvey *et al.* method and are expressed by means of electronic energy relative to the former species.

Given that the hydrometallation step—and its reverse—is more likely to proceed on the **triplet manifold**, the **transition state** for the **2,1-hydrometallation** appears to be facilitated on the **triplet surface** than on the **quintet surface** by means of **3.3 kcal/mol**. In contrast, for the relevant **1,2-hydrometallation** pathway, the **triplet and quintet transition states** exhibit **almost identical energies of +24.3 kcal/mol**, suggesting comparable accessibility on both spin surfaces. In addition, these latter are higher in energy by means of 4 kcal/mol with respect to the triplet **2,1-hydrometallation** transitions states. This suggests the feasibility of **1,2-hydrometallation** is energetically and spin-hindered while the **2,1-hydrometallation** facilitated via a two-state reactivity.

Interestingly, with our catalytic system **2a**, the **1,2-hydrometallation** step operates at the triplet manifold with an energy barrier of 19.2 kcal/mol relative to **[2b]$_2$** and an energy gap of 4.2 kcal/mol relative to the higher energy transition state at the quintet manifold. In addition, this value is lower by 5 kcal/mol compared to the energy barrier of **1,2-hydrometallation** transition state calculated with **BDI** supported Fe(II) complex. This trend was also notable for β-H elimination step for which the calculated energy for the corresponding transition states at the triplet manifold were found to be lower with **AA** supported Fe(II) complex by means of **5.4 kcal/mol** for the *E*-selective TS and **5.1 kcal/mol** for the Z-selective TS with respect those calculated with BDI analogues. The triplet-quintet energy gap for β-H elimination from species **AA**-Fe(II) complex (**IX**) are of the order of -**6.0 kcal/mol** for the *E*-selective TS and -**7.0 kcal/mol** for the Z-selective TS while these values were found to be lower with **BDI** ligand at -**4.1 kcal/mol** and -**5.6 kcal/mol** respectively. This suggests that our **AA**-ligand effectively stabilizes the triplet spin state to higher extent enhancing its accessibility and underscore the ligand pivotal role in promoting **alkene isomerization** via two state reactivity.

7-Catalyst structure optimization

In an argon-filled glovebox, the appropriate Fe-catalyst **2** (0.025 mmol) was dissolved in deuterated benzene and added to a 10 mL screw-capped tube, equipped with a magnetic stir bar, containing $Cy_2NH.BH_3$ (4.9 mg, 0.025 mmol). Next, allylbenzene **3a** (59.1 mg, 0.5 mmol) was added, and the reaction was stirred at 60 °C for 24 h (entries 1-5, Table S7) and 48 h (entries 6-10, Table S8). Upon completion, the reaction mixture was exposed to air and 1,3,5-trimethoxybenzene (42 mg, 0.25 mmol, 0.5 equiv.) was added to the reaction vessel. Conversion, spectroscopic yield and stereoselectivity given by *E*:*Z* ratio were determined by ^{1}H NMR analysis using 1,3,5-trimethoxybenzene as internal standard.

Table S7. Catalyst structure optimization studies

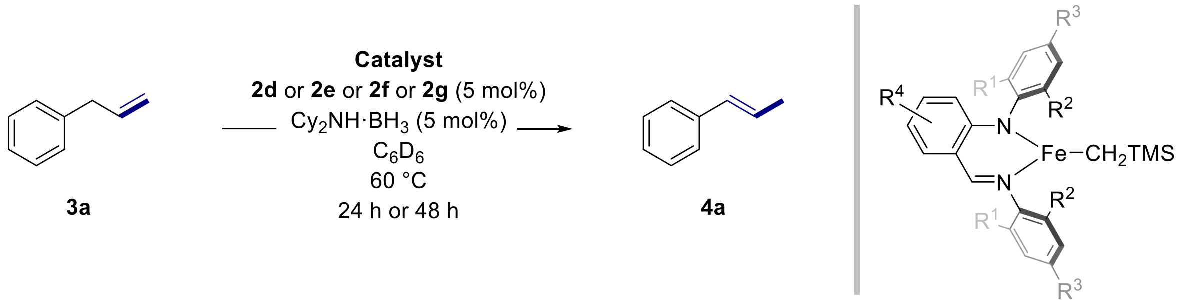

2d $R^1 = R^2 = i$Pr, R^3 = H, R^4 = 5-MeO
2e $R^1 = R^2 = i$Pr, R^3 = H, R^4 = 4-MeO
2f $R^1 = R^2 = i$Pr, R^3 = H, R^4 = 4-F
2g $R^1 = R^2 = R^3$ = Me, R^4 = H

Entry	Cat	*t* [h]	Conv [%][b]	Yield [%][b]	*E*:*Z* [b]
1	**2a**	24	>99	99	8:1
2	**2d**	24	56	55	8:1
3	**2e**	24	92	87	7:1
4	**2f**	24	>99	95	12:1
5	**2g**	24	>99	>99	26:1
6	**2a**	48	>99	98	28:1
7	**2d**	48	>99	99	9:1
8	**2e**	48	>99	97	24:1
9	**2f**	48	>99	97	24:1
10	**2g**	48	>99	97	25:1

[a] reactions run on a 0.5 mmol scale at 60 °C for 24 or 48 h with **3a** (1.0 equiv), $Cy_2N{\cdot}BH_3$ (5 mol%), **2** (5 mol%) in benzene (0.8 M). [b] determined by ^{1}H NMR using 1,3,5-trimethoxybenzene as internal standard.

8-Time course studies with complex 2g

2g-catalyzed isomerization of allylbenzene 3a:

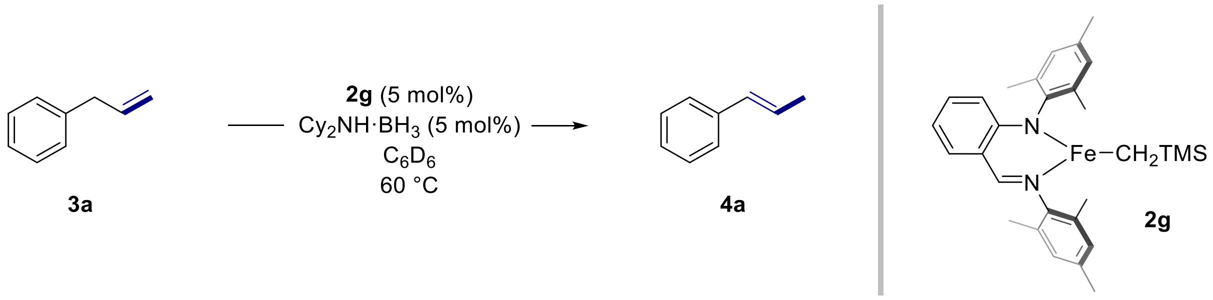

In an argon-filled glovebox, **2g** (12.5 mg, 0.025 mmol) was dissolved in deuterated benzene (0.6 mL) and added to a 10 mL screw-capped tube, equipped with a magnetic stir bar, containing $Cy_2NH.BH_3$ (4.9 mg, 0.025 mmol). Next, allylbenzene **4a** (66.1 mg, 0.5 mmol) was added and the reaction was stirred at 60 °C. Samples (0.2 mL) were taken over time, diluted with cold deuterated benzene (1 mL), exposed to air and 1,3,5-trimethoxybenzene (14 mg, 0.08 mmol) was added prior to filtration through a Millipore filter. The consumption of **3a** and the appearance of product **4a** over time were determined by ^{1}H NMR analysis using 1,3,5-trimethoxybenzene as internal standard. Data were processed using time normalization analysis to determine rate constant value **k_r(2g)** of **2g**-catalyzed isomerization of **3a** which was found to be of 0.12 min^{-1}. This value is a four-fold increase compared with that obtained with complex **2a**.

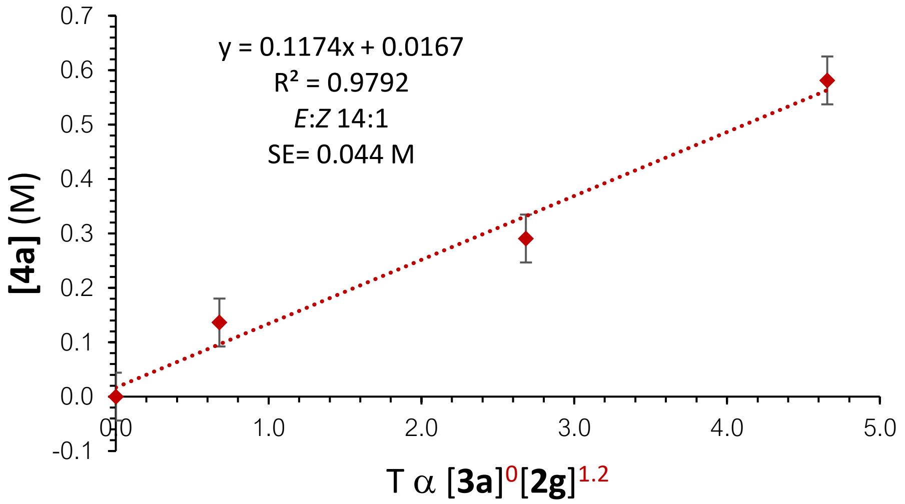

Figure S51: Time Normalized Analysis to determine **k_r (2g)** of **2g**-catalyzed isomerization of **3a**

2g-catalyzed isomerization of but-3-en-1-ylbenzene 3ab

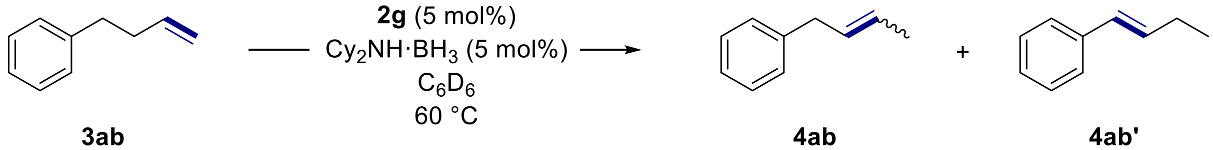

3ab 4ab 4ab'

In an argon-filled glovebox, **2g** (12.5 mg, 0.025 mmol) was dissolved in deuterated benzene (0.6 mL) and added to a 10 mL screw-capped tube, equipped with a magnetic stir bar, containing $Cy_2NH.BH_3$ (4.9 mg, 0.025 mmol). Next, but-3-en-1-ylbenzene **4ab** (66.1 mg, 0.5 mmol) was added and the reaction was stirred at 60 °C. Samples (0.2 mL) were taken over time, diluted with cold deuterated benzene (1 mL), exposed to air and 1,3,5-trimethoxybenzene (14 mg, 0.08 mmol) was added prior to filtration through a Millipore filter. The consumption of **3ab** and the appearance of products **4ab** and **4ab**' over time were determined by ^{1}H NMR analysis using 1,3,5-trimethoxybenzene as internal standard.

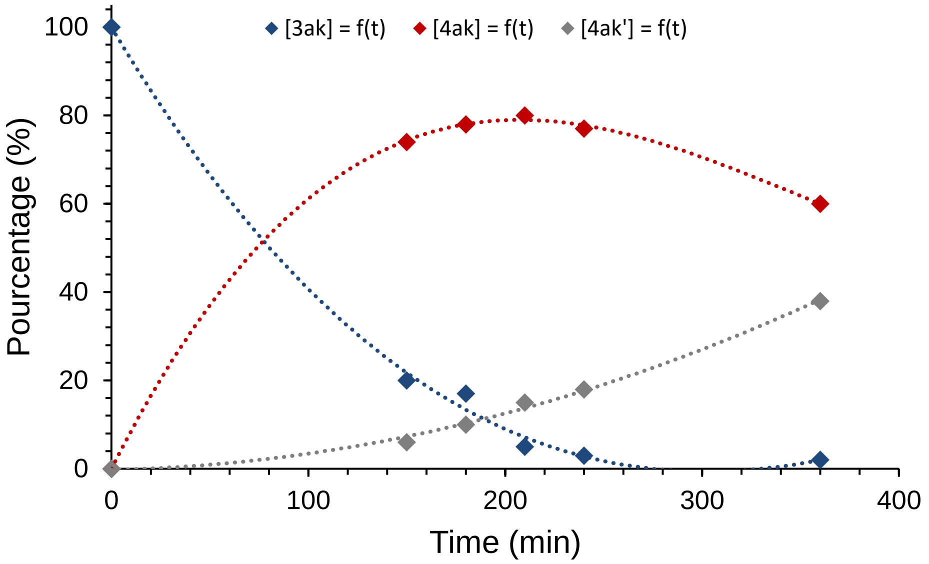

Figure S52. Reaction profile of **2g**-catalyzed isomerization of **4ab.**

9-Catalytic Procedures for Fe-catalyzed isomerization of alkene and characterization data

General catalytic procedure for Fe-catalyzed monoisomerization of alkene under optimized conditions: In an argon-filled glovebox, **2g** (12.5 mg, 0.025 mmol) was dissolved in deuterated benzene (0.6 mL) and added to a 10 mL screw-capped tube, equipped with a magnetic stir bar, containing $Cy_2NH.BH_3$ (4.9 mg, 0.025 mmol). Next, alkene **3** (0.5 mmol) was added and the reaction was stirred at 60 °C for 24 h (unless otherwise stated). Upon completion, the reaction mixture was exposed to air and 1,3,5-trimethoxybenzene (42 mg, 0.25 mmol, 0.5 equiv.) was added to the reaction vessel. Conversion, spectroscopic yield and stereoselectivity given by *E*:*Z* ratio were determined by ^{1}H NMR analysis using 1,3,5-trimethoxybenzene as internal standard.

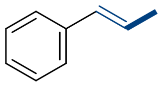

(*E*)-**4a**

(*E*)-prop-1-en-1-ylbenzene 4a: Prepared on a 0.5 mmol scale from allylbenzene **3a** according to the general catalytic procedure under optimized conditions. 97% NMR yield, *E*:*Z* 25:1. Purification by flash chromatography on silica gel using pentane as eluant yielded product as colorless oil (49.3 mg, 0.42 mmol, 84%). **^{1}H NMR** (400 MHz, $CDCl_3$, RT) *δ* 7.39-7.30 (m, 4H), 7.23 (tt, 1H, *J* = 1.6, 7.1 Hz), 6.45 (dq, 1H, *J* = 15.7, 1.6Hz), 6.28 (dq, 1H, *J* = 15.7, 6.5 Hz), 1.83 (dd, 3H, *J* = 1.6, 6.5 Hz). **^{13}C{^{1}H} NMR** (100 MHz, $CDCl_3$, RT) *δ* 138.0, 131.1, 128.6, 126.8, 125.9, 125.8, 18.6. These data are in agreement with literature data.[29]

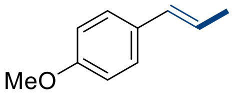

(*E*)-**4c**

(*E*)-1-methoxy-4-(prop-1-en-1-yl)benzene 4c: Prepared on a 0.5 mmol scale from 1-allyl-4-methoxybenzene **3c** according to the general catalytic procedure under optimized conditions. 99% NMR yield, *E*:*Z* 22:1.

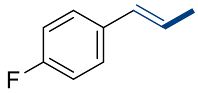

(*E*)-**4d**

(*E*)-1-fluoro-4-(prop-1-en-1-yl)benzene 4d: Prepared on a 0.5 mmol scale from 1-allyl-4-fluorobenzene **3d** according to the general catalytic procedure under optimized conditions. 99% NMR yield, *E*:*Z* 24:1.

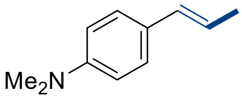

(*E*)-**4e**

(*E*)-*N*,*N*-dimethyl-4-(prop-1-en-1-yl)aniline 4e: Prepared on a 0.5 mmol scale from 4-allyl-*N*,*N*-dimethylaniline **3e** according to the general catalytic procedure under optimized conditions. >99% NMR yield, *E*:*Z* 24:1. Purification by flash chromatography on silica gel using pentane/Et_2O : 98/2 as eluant yielded product as off-white solid (78.1 mg, 0.48 mmol, 97%). **^{1}H NMR** (400 MHz, $CDCl_3$, RT) *δ* 7.24 (d, 2H, *J* = 8.9 Hz), 6.69 (d, 2H, *J* = 8.9 Hz), 6.34 (dq, 1H, *J* = 15.7, 1.7 Hz), 6.04 (dq, 1H, *J* = 15.7, 6.6 Hz), 2.95 (s, 6H), 1.86 (dd, 3H, *J* = 1.7, 6.6 Hz). **^{13}C{^{1}H } NMR** (100 MHz, $CDCl_3$, RT) *δ* 149.7, 130.9, 126.9, 126.8, 121.5, 112.8, 40.8, 18.6. These data are in agreement with literature data.[30]

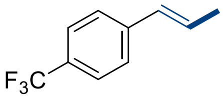

(*E*)-**4f**

(*E*)-1-(prop-1-en-1-yl)-4-(trifluoromethyl)benzene 4f: Prepared on a 0.5 mmol scale from 1-allyl-4-(trifluoromethyl)benzene **3f** according to the general catalytic procedure using **2g** (50 mg, 0.1) and $Cy_2NH \cdot BH_3$ (19.6 mg, 0.1 mmol) and stirring for 24 h at 80 °C. 84% NMR yield, *E*:*Z* 18:1. Purification by flash chromatography on silica gel using pentane as eluant yielded product as colorless viscous oil (70.4 mg, 0.38 mmol, 76%). **^{1}H NMR** (400 MHz, $CDCl_3$, RT) δ 7.54 (d, 2H, *J* = 8.1 Hz), 7.41 (d, 2H, *J* = 8.1 Hz), 6.44 (d, 1H, *J* = 15.7 Hz), 6.35 (dq, 1H, *J* = 15.7, 5.9 Hz), 1.92 (d, 3H, *J* = 5.9 Hz). **^{13}C{^{1}H } NMR** (100 MHz, $CDCl_3$, RT) δ 141.5, 130.1, 128.8, 128.7 (q, *J* = 31.8 Hz), 126.1, 125.8, 125.5 (q, *J* = 36 Hz), 123.1, 120.4, 18.6. **^{19}F{^{1}H } NMR** (376 MHz, $CDCl_3$, RT) δ -62.42. These data are in agreement with literature data.[1]

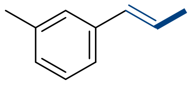

(*E*)-**4g**

(*E*)-1-methyl-3-(prop-1-en-1-yl)benzene 4g: Prepared on a 0.5 mmol scale from 1-allyl-3-methylbenzene **3g** according to the general catalytic procedure under optimized conditions. 98% NMR yield, *E*:*Z* 26:1. Purification by flash chromatography on silica gel using pentane as eluant yielded product as colorless oil (57.6 mg, 0.44 mmol, 87%). **^{1}H NMR** (300 MHz, $CDCl_3$, RT) δ 7.23-7.12 (m, 3H), 7.03-7.00 (d, 1H, *J* = 7.0 Hz), 6.38 (dq, 1H, *J* = 15.8, 1.4 Hz), 6.21 (dq, 1H, *J* = 15.8, 6.4 Hz), 2.34 (s, 3H), 1.88 (dd, 3H, *J* = 1.4, 6.4 Hz). **^{13}C{^{1}H } NMR** (75 MHz, $CDCl_3$, RT) δ 138.1, 138.0, 131.2, 128.5, 127.6, 126.7, 125.6, 123.1, 21.5, 18.6. These data are in agreement with literature data.[29]

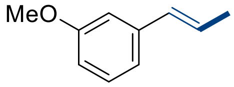

(*E*)-**4h**

(*E*)-1-methoxy-3-(prop-1-en-1-yl)benzene 4h: Prepared on a 0.5 mmol scale from 1-allyl-3-methoxybenzene **3h** according to the general catalytic procedure under optimized conditions. 99% NMR yield, *E*:*Z* 30:1. Purification by flash chromatography on silica gel using pentane/Et_2O : 99/1 as eluant yielded product as yellowish oil (66.7 mg, 0.45 mmol, 90%). **^{1}H NMR** (400 MHz, $CDCl_3$, RT) δ 7.21 (t, 1H, *J* = 7.9 Hz), 6.93 (d, 1H, *J* = 7.9 Hz), 6.87 (m, 1H), 6.75 (dd, 1H, *J* = 2.3, 7.9 Hz), 6.38 (d, 1H, *J* = 15.7 Hz), 6.24 (dq, 1H, *J* = 15.7, 6.4 Hz), 3.81 (s, 3H), 1.88 (dd, 3H, *J* = 1.1, 6.4 Hz). **^{13}C{^{1}H } NMR** (100 MHz, $CDCl_3$, RT) δ 159.9, 139.6, 131.1, 129.6, 126.1, 118.7, 112.5, 111.4, 55.3, 18.6. These data are in agreement with literature data.[30]

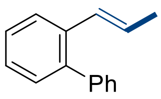

(*E*)-**4i**

(*E*)-2-(prop-1-en-1-yl)-1,1'-biphenyl 4i: Prepared on a 0.5 mmol scale from 2-allyl-1,1'-biphenyl **3i** according to the general catalytic procedure under optimized conditions. 94% NMR yield, *E*:*Z* 15:1. Purification by flash chromatography on silica gel using pentane as eluant yielded product as colorless oil (88.9 mg, 0.46 mmol, 92%). **^{1}H NMR** (400 MHz, $CDCl_3$, RT) δ 7.61 (d, 1H, *J* = 7.5 Hz), 7.47-7.29 (m, 8H), 6.43 (dq, 1H, *J* = 15.7, 1.7 Hz), 6.21 (dq, 1H, *J* = 15.7, 6.7 Hz), 1.83 (dd, 3H, *J* = 1.7, 6.7 Hz). **^{13}C{^{1}H } NMR** (100 MHz, $CDCl_3$, RT) δ 141.4, 140.2, 136.0, 130.2 129.9, 128.1, 127.5, 126.9, 126.8, 126.6, 125.9, 18.8.

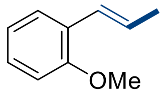

(*E*)-**4j**

(*E*)-1-methoxy-2-(prop-1-en-1-yl)benzene 4j: Prepared on a 0.5 mmol scale from 1-allyl-2-methoxybenzene **3j** according to the general catalytic procedure under optimized conditions. 97% NMR yield, *E*:*Z* 19:1. Purification by flash chromatography on silica gel using pentane/Et_2O : 99/1 as eluant yielded product as colorless oil (64.5 mg, 0.44 mmol, 87%). ^{1}**H NMR** (400 MHz, $CDCl_3$, RT) δ 7.45 (dd, 1H, *J* = 1.2, 7.5 Hz), 7.23 (dt, 1H, *J* = 1.4, 7.5 Hz), 6.96 (t, 1H, *J* = 7.5 Hz), 6.90 (d, 1H, *J* = 8.2 Hz), 6.79 (dq, 1H, *J* = 15.9, 1.6 Hz), 6.29 (dq, 1H, *J* = 15.9, 6.6 Hz), 3.88 (s, 3H), 1.96 (dd, 3H, *J* = 1.6, 6.6 Hz). ^{13}**C{^{1}H } NMR** (100 MHz, $CDCl_3$, RT) δ 156.2, 127.8, 127.1, 126.6, 126.5, 125.7, 120.7, 110.8, 55.5, 19.0. These data are in agreement with literature data.[30]

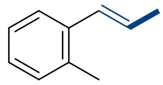

4k

(*E*)-1-methyl-2-(prop-1-en-1-yl)benzene 4k: Prepared on a 0.5 mmol scale from 1-allyl-2-methylbenzene **3k** according to the general catalytic procedure under optimized conditions. 99% NMR yield, *E*:*Z* 8.5:1.

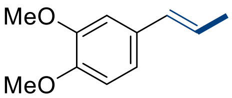

(*E*)-**4l**

(*E*)-1,2-dimethoxy-4-(prop-1-en-1-yl)benzene 4l: Prepared on a 0.5 mmol scale from 4-allyl-1,2-dimethoxybenzene **3l** according to the general catalytic procedure under optimized conditions. 98% NMR yield, *E*:*Z* 25:1 . Purification by flash chromatography on silica gel using pentane/EtOAc : 95/5 as eluant yielded product as colorless oil (78.9 mg, 0.44 mmol, 88%). ^{1}**H NMR** (400 MHz, $CDCl_3$, RT) δ 6.88 (d, 1H, *J* = 1.5 Hz), 6.85 (dd, 1H, *J* = 1.5, 8.3 Hz), 6.78 (d, 1H, *J* = 8.3 Hz), 6.34 (d, 1H, *J* = 15.7 Hz), 6.1 (dq, 1H, *J* = 15.7, 6.6 Hz), 3.88 (s, 3H), 3.86 (s, 3H) 1.86 (dd, 3H, *J* = 1.4, 6.6 Hz). ^{13}**C{^{1}H } NMR** (100 MHz, $CDCl_3$, RT) δ 149.0, 148.2, 131.2, 130.7, 123.8, 118.7, 111.2, 108.5, 55.9, 55.8, 18.4. These data are in agreement with literature data.[31]

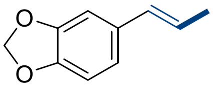

(*E*)-**4m**

(*E*)-5-(prop-1-en-1-yl)benzo[*d*][1,3]dioxole 4m: Prepared on a 0.5 mmol scale from 5-allylbenzo[*d*][1,3]dioxole **3m** according to the general catalytic procedure under optimized conditions. >99% NMR yield, *E*:*Z* 19:1. Purification by flash chromatography on silica gel using pentane as eluant yielded product as yellowish oil (78.8 mg, 0.49 mmol, 98%). ^{1}**H NMR** (400 MHz, $CDCl_3$, RT) δ 6.88 (s, 1H), 6.73 (s, 2H), 6.31 (d, 1H, *J* = 15.6 Hz), 6.06 (dq, 1H, *J* = 15.6, 6.6 Hz), 5.92 (s, 2H), 1.85 (d, 3H, *J* = 6.6 Hz). ^{13}**C{^{1}H } NMR** (100 MHz, $CDCl_3$, RT) δ 148.0, 146.6, 132.6, 130.7, 124.0, 120.2, 108.3, 105.5, 101.0, 18.4.[31]

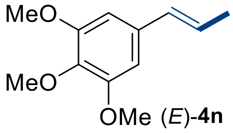

(*E*)-**4n**

(*E*)-1,2,3-trimethoxy-5-(prop-1-en-1-yl)benzene 4n: Prepared on a 0.5 mmol scale from 5-allyl-1,2,3-trimethoxybenzene **3n** according to the general catalytic procedure under optimized conditions. 95% NMR yield, *E*:*Z* 28:1. Purification by flash chromatography on silica gel using pentane/EtOAc : 95/5 as eluant yielded product as yellow oil (98.5 mg, 0.47 mmol, 94%). ^{1}**H NMR** (400 MHz, $CDCl_3$, RT) δ 6.54 (s, 2H), 6.31 (dq, 1H, *J* = 15.7, 1.6 Hz), 6.13 (dq, 1H, *J* = 15.7, 6.5 Hz), 3.84 (s, 6H), 3.82 (s, 3H), 1.85 (dd, 3H, *J* = 1.6, 6.5 Hz). ^{13}**C{^{1}H } NMR** (100

MHz, $CDCl_3$, RT) δ 153.2, 137.1, 133.8, 130.9, 125.2, 102.8, 60.9, 56.0, 18.3.[31]

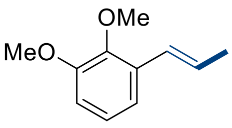

(*E*)-**4o**

(*E*)-1,2-dimethoxy-3-(prop-1-en-1-yl)benzene 4o: Prepared on a 0.5 mmol scale from 1-allyl-2,3-dimethoxybenzene **3o** according to the general catalytic procedure under optimized conditions. 98% NMR yield, *E*:*Z* 20:1. Purification by flash chromatography on silica gel using pentane/EtOAc : 95/5 as eluant yielded product as yellow oil (78.5 mg, 0.44 mmol, 88%). **^{1}H NMR** (400 MHz, $CDCl_3$, RT) δ 7.07 (dd, 1H, J = 1.35, 7.9 Hz), 7.00 (t, 1H, J = 7.9 Hz), 6.80-6.70 (m, 2H), 6.27 (dq, 1H, J = 15.6, 6.6 Hz), 3.85 (s, 3H), 3.82 (s, 3H), 1.93 (dd, 3H, J = 1.6, 6.6 Hz). **^{13}C{^{1}H } NMR** (100 MHz, $CDCl_3$, RT) δ 153.0, 146.0, 132.0, 127.1, 125.1, 123.9, 117.9, 110.5, 60.7, 55.7, 18.9.

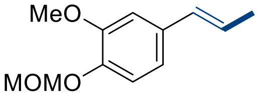

(*E*)-**4p**

(*E*)-2-methoxy-1-(methoxymethoxy)-4-(prop-1-en-1-yl)benzene 4p: Prepared on a 0.5 mmol scale from 4-allyl-2-methoxy-1-(methoxymethoxy)benzene **3p** according to the general catalytic procedure using **2g** (25.0 mg, 0.05 mmol) and $Cy_2NH{\cdot}BH_3$ (9.8 mg, 0.05 mmol). >99% NMR yield, *E*:*Z* 21:1. Purification by flash chromatography on silica gel using pentane/EtOAc : 95/5 as eluant yielded product as as colorless oil (88.8 mg, 0.43 mmol, 86%). **^{1}H NMR** (400 MHz, $CDCl_3$, RT) δ 7.07 (d, 1H, J = 8.3 Hz), 6.89 (d, 1H, J = 1.9 Hz), 6.84 (dd, 1H, J = 1.9, 8.3 Hz), 6.34 (m, 1H), 6.12 (dq, 1H, J = 15.7, 6.6 Hz), 5.21 (s, 2H), 3.89 (s, 3H), 3.51 (s, 3H), 1.86 (dd, 3H, J = 1.6, 6.6 Hz). **^{13}C{^{1}H } NMR** (100 MHz, $CDCl_3$, RT) δ 149.9, 154.7, 132.9, 130.7, 124.5, 118.8, 116.6, 109.2, 95.7, 56.3, 55.9, 18.5. **HRMS (ESI)** *m/z* Calcd for $C_{12}H_{16}NaO_3^+$ $[M+Na]^+$ 231.0992. Found: 231.0987.

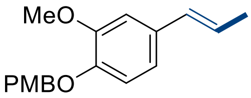

(*E*)-**4q**

(*E*)-2-methoxy-1-((4-methoxybenzyl)oxy)-4-(prop-1-en-1-yl)benzene 4q: Prepared on a 0.5 mmol scale from 4-allyl-2-methoxy-1-((4-methoxybenzyl)oxy)benzene **3q** according to the general catalytic procedure under optimized conditions. >99% NMR yield, *E*:*Z* 22:1. Purification by flash chromatography on silica gel using pentane/EtOAc : 95/5 as eluant yielded product as white solid (134.1 mg, 0.47 mmol, 94%). **^{1}H NMR** (400 MHz, $CDCl_3$, RT) δ 7.36 (d, 2H, J = 8.6 Hz), 6.90-6.87 (m, 3H), 6.84-6.78 (m, 2H), 6.33 (dq, 1H, J = 15.7, 1.5 Hz), 6.10 (dq,1H, J = 15.7, 6.6 Hz), 5.07 (s, 2H), 3.89 (s, 3H), 3.80 (s, 3H), 1.86 (dd, 3H, J = 1.5, 6.6 Hz). **^{13}C{^{1}H } NMR** (100 MHz, $CDCl_3$, RT) δ159.4, 149.9, 147.5, 131.8, 130.7, 129.4, 129.1, 124.0, 118.7, 114.4, 114.0, 109.2, 71.1, 56.0, 55.4, 18.5. **HRMS (ESI)** *m/z* Calcd for $C_{18}H_{20}NaO_3^+$ $[M+Na]^+$ 307.1305. Found: 307.1309.

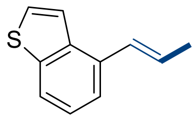

(*E*)-**4r**

(*E*)-4-(prop-1-en-1-yl)benzo[*b*]thiophene 4r: Prepared on a 0.5 mmol scale from 4-allylbenzo[*b*]thiophene **3r** according to the general catalytic procedure using **2g** (25 mg, 0.05 mmol) and $Cy_2NH{\cdot}BH_3$ (9.8 mg, 0.05 mmol). 89% NMR yield, *E*:*Z* 21:1. Purification by flash chromatography on silica gel using pentane as eluant yielded product as colorless oil (72.5 mg, 0.42 mmol, 84%). **^{1}H NMR** (400 MHz, $CDCl_3$, RT) δ 7.76 (d, 1H, *J* = 8.2 Hz), 7.56 (dd, 1H, *J* = 0.8, 5.6 Hz), 7.46-7.44 (m, 2H), 7.31 (t, 1H, *J* = 7.7 Hz), 6.91 (dq, 1H, *J* = 15.7, 1.6 Hz), 6.35 (dq, 1H, *J* = 15.7, 6.7 Hz), 2.00 (dd, 3H, *J* = 1.6, 6.7 Hz). **^{13}C{^{1}H } NMR** (100 MHz, $CDCl_3$, RT) δ 140.3, 137.5, 133.6, 128.6, 128.2, 126.0, 124.5, 121.9, 121.0, 120.6, 19.0.

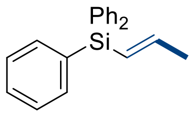

(*E*)-**4s**

(*E*)-triphenyl(prop-1-en-1-yl)silane 4s: Prepared on a 0.5 mmol scale from allyltriphenylsilane **3s** (97.1 mg, 0.5 mmol) according to the general catalytic procedure under optimized conditions. 97% NMR yield, *E*:*Z* 15:1. Purification by flash chromatography on silica gel using pentane as eluant yielded product as white solid (133.0 mg, 0.44 mmol, 89%). **^{1}H NMR** (400 MHz, $CDCl_3$, RT) δ 7.56 (dd, 6H, *J* = 1.4, 7.6 Hz), 7.44-7.36 (m, 9H), 6.23 (m, 2H), 1.96 (d, 3H, *J* = 4.3 Hz). **^{13}C{^{1}H} NMR** (100 MHz, $CDCl_3$, RT) δ 148.5, 136.1, 135.1, 129.5, 127.9, 125.3, 23.1. These data are in agreement with literature data.[31]

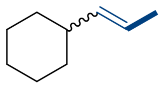

4y

prop-1-en-1-ylcyclohexane 4y: Prepared on a 0.5 mmol scale from allylcyclohexane **3y** according to the general catalytic procedure under optimized conditions. 81% NMR yield, *E*:*Z* 3:1.

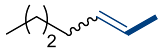

4z

2-octene 4z: Prepared on a 0.5 mmol scale from 1-octene **3z** according to the general catalytic procedure under optimized conditions using **2a** (14.6 mg, 0.025 mmol). 63 % NMR yield, *E*:*Z* 2.5:1.

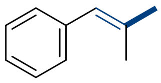

4aa

(2-methylprop-1-en-1-yl)benzene 4aa: Prepared on a 0.5 mmol scale from (2-methylallyl)benzene **3aa** according to the general catalytic procedure using **2g** (50 mg, 0.1) and $Cy_2NH{\cdot}BH_3$ (19.6 mg, 0.1 mmol) and stirring for 48 h at 80 °C. 92% NMR yield. Purification by flash chromatography on silica gel using pentane as eluant yielded product as colorless oil (50.7 mg, 0.38 mmol, 76%). **^{1}H NMR** (400 MHz, $CDCl_3$, RT) δ 7.35-7.31 (m, 2H), 7.25-7.23 (m, 2H), 7.19 (tt, 1H, *J* = 1.4, 7.2 Hz), 6.29 (s, 1H), 1.92 (d, 3H, *J* = 1.4 Hz), 1.88 (d, 3H, *J* = 1.4 Hz). **^{13}C{^{1}H} NMR** (100 MHz, $CDCl_3$, RT) δ 138.8, 135.6, 128.9, 128.1, 125.9, 125.2, 27.0, 19.5.[32]

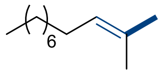

4ab

2-methylundec-2-ene 4ab: Prepared on a 0.5 mmol scale from 2-methylundec-1-ene **3ab** according to the general catalytic procedure using **2g** (50 mg, 0.1) and $Cy_2NH{\cdot}BH_3$ (19.6 mg, 0.1 mmol) and stirring for 48 h at 80 °C. 52% NMR yield (76% conversion).

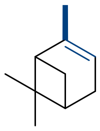
4ac

2,6,6-trimethylbicyclo[3.1.1]hept-2-ene 4ac: Prepared on a 0.5 mmol scale from 6,6-dimethyl-2-methylenebicyclo[3.1.1]heptane **3ac** according to the general catalytic procedure using **2g** (50 mg, 0.1) and $Cy_2NH{\cdot}BH_3$ (19.6 mg, 0.1 mmol) and stirring for 48 h at 80 °C. 56% NMR yield (full conversion).

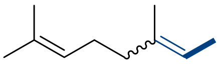
4ad

2,6-dimethylocta-2,6-diene 4ad: Prepared on a 0.5 mmol scale from 3,7-dimethylocta-1,6-diene **3ad** according to the general catalytic procedure using **2g** (25 mg, 0.05) and $Cy_2NH{\cdot}BH_3$ (9.8 mg, 0.05 mmol). 92% NMR yield. Purification by preparative thin layer chromatography using pentane as eluant yielded product as colorless oil. **^{1}H NMR** (600 MHz, C_6D_6, RT) δ 5.30-5.20 (m, 2H), 2.17-2.05 (m, 4H), 1,69-1.65 (m, 4H), 1.57-1.54 (m, 8H). **^{13}C{^{1}H} NMR** (150 MHz, C_6D_6, RT) δ 135.9, 135.8, 131.3, 131.1, 125.0, 124.9, 119.5, 118.7, 40.2, 31.9, 27.2, 26.8, 25.9, 23.6, 17.7, 17.7, 15.7, 13.6, 13.5. [33]

General catalytic procedure for Fe-catalyzed geometrical isomerization of alkene under optimized conditions: In an argon-filled glovebox, **2g** (12.5 mg, 0.025 mmol) was dissolved in deuterated benzene (0.6 mL) and added to a 10 mL screw-capped tube, equipped with a magnetic stir bar, containing $Cy_2NH.BH_3$ (4.9 mg, 0.025 mmol). Next, alkene **3** or stereoisomeric mixture of alkene **4** (0.5 mmol) was added and the reaction was stirred at 60 °C for 24 h. Upon completion, the reaction mixture was exposed to air and 1,3,5-trimethoxybenzene (42 mg, 0.25 mmol, 0.5 equiv.) was added to the reaction vessel. Conversion, spectroscopic yield and stereoselectivity given by *E*:*Z* ratio were determined by ^{1}H NMR analysis using 1,3,5-trimethoxybenzene as internal standard.

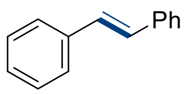
(*E*)-**3b**

(*E*)-1,2-diphenylethene (*E*)-3b: Prepared on a 0.5 mmol scale from (*Z*)-1,2-diphenylethene **(*Z*)-3b** according to the general catalytic procedure under optimized conditions. >99% NMR yield. Purification by flash chromatography on silica gel using pentane as eluant yielded product as white crystalline solid (83.0 mg, 0.46 mmol, 92%). **^{1}H NMR** (400 MHz, $CDCl_3$, RT) δ 7.57 (d, 4H, J = 7.8 Hz), 7.42 (t, 4H, J = 7.8 Hz), 7.32 (t, 2H, J = 7.8 Hz), 7.17 (s, 2H). **^{13}C{^{1}H} NMR** (100 MHz, $CDCl_3$, RT) δ 137.4, 128.7, 127.6, 126.6. These data are in agreement with literature data.[34]

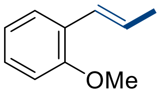
(*E*)-**4j**

(*E*)-1-methoxy-2-(prop-1-en-1-yl)benzene 4j: Prepared on a 0.5 mmol scale from stereoisomeric mixture of 1-methoxy-2-(prop-1-en-1-yl)benzene **4j** (*E*:*Z* 3.5:1) according to the general catalytic procedure under optimized conditions. >99% NMR yield, *E*:*Z* 5:1.

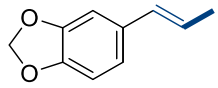

(*E*)-**4m**

(*E*)-5-(prop-1-en-1-yl)benzo[*d*][1,3]dioxole 4m: Prepared on a 0.5 mmol scale from stereoisomeric mixture of 5-(prop-1-en-1-yl)benzo[*d*][1,3]dioxole **4m** (*E*:*Z* 7:1) according to the general catalytic procedure under optimized conditions. >99% NMR yield, *E*:*Z* 18:1.

General catalytic procedure for Fe-catalyzed regiodivergent isomerization of alkene under optimized conditions: In an argon-filled glovebox, **2g** (12.5 mg, 0.025 mmol) was dissolved in deuterated benzene (0.6 mL) and added to a 10 mL screw-capped tube, equipped with a magnetic stir bar, containing $Cy_2NH.BH_3$ (4.9 mg, 0.025 mmol). Next, alkene **3** (0.5 mmol) was added and the reaction was stirred at 60 °C for 24 h (unless otherwise stated). Upon completion, the reaction mixture was exposed to air and 1,3,5-trimethoxybenzene (42 mg, 0.25 mmol, 0.5 equiv.) was added to the reaction vessel. Conversion, spectroscopic yield and stereoselectivity given by *E*:*Z* ratio were determined by ^{1}H NMR analysis using 1,3,5-trimethoxybenzene as internal standard.

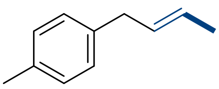

4ae

1-(but-2-en-1-yl)-4-methylbenzene 4ae: Prepared on a 0.5 mmol scale from **1-**(but-3-en-1-yl)-4-methylbenzene **3ae** according to the general catalytic procedure under optimized conditions and stirring for 3 h. 81% NMR yield, *E*:*Z* 1.8:1. Purification by flash chromatography on silica gel using pentane as eluant yielded product as colorless oil (54.1 mg, 0.37 mmol, 74%). **^{1}H NMR** (400 MHz, $CDCl_3$, RT) δ 7.17-7.12 (m, 4H), 5.68-5.52 (m, 2H), 3.42 (d, 0.4H_Z, J = 5.5 Hz), 3.28 (d, 1.5H_E, J = 6.3 Hz), 2.38 (s, 3H), 1.76 (d, 0.6H_Z, J = 5.0 Hz), 1.69 (dd, 2.2H_E, J = 1.2, 6.1 Hz). **^{13}C{^{1}H} NMR** (100 MHz, $CDCl_3$, RT) δ 138.3, 138.1, 135.5, 135.4, 130.5, 129.4, 129.2, 129.1, 128.5, 128.3, 126.2, 124.7, 38.8, 32.8, 21.1, 18.0, 13.0. These data are in agreement with literature data.[35]

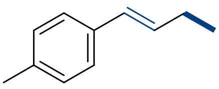

4ae'

(*E*)-1-(but-1-en-1-yl)-4-methylbenzene 4ae': Prepared on a 0.5 mmol scale from 1-(but-3-en-1-yl)-4-methylbenzene **3ae** according to the general catalytic procedure under optimized conditions. 84% NMR yield, *E*:*Z* 47:1. Purification by flash chromatography on silica gel using pentane as eluant yielded product as colorless oil (57.0 mg, 0.39 mmol, 78%). **^{1}H NMR** (400 MHz, $CDCl_3$, RT) δ 7.29 (d, 2H, J = 7.9 Hz), 7.14 (d, 2H, J = 7.9 Hz), 6.40 (d, 1H, J = 15.8 Hz), 6.26 (dt, 1H, J = 15.8, 6.4 Hz), 2.37 (s, 3H), 2.31-2.23 (m, 2H), 1.14 (t, 3H, J = 7.5 Hz). **^{13}C{^{1}H} NMR** (100 MHz, $CDCl_3$, RT) δ 136.5, 135.3, 131.7, 129.3, 128.7, 125.9, 26.2, 21.2, 13.8. These data are in agreement with literature data.[29]

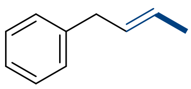

4af

but-2-en-1-ylbenzene 4af: Prepared on a 0.5 mmol scale from but-3-en-1-ylbenzene **3af** according to the general catalytic procedure under optimized conditions and stirring for 3 h. 84% NMR yield, *E*:*Z* 2:1. Purification by flash chromatography on silica

gel using pentane as eluant yielded product as colorless oil (52.9 mg, 0.4 mmol, 80%). **^{1}H NMR** (400 MHz, $CDCl_3$, RT) δ 7.38-7.34 (m, 2H), 7.28-7.25 (m, 3H), 5.71-5.55 (m, 2H), 3.48 (d, 0.6H_Z, J = 5.0 Hz), 3.39 (d, 1.4H_E, J = 6.4 Hz), 1.80 (d, 0.8H_Z, J = 4.8 Hz), 1.76 (d, 2.1H_E, J = 6.0 Hz). **^{13}C{^{1}H} NMR** (100 MHz, $CDCl_3$, RT) δ 141.2, 141.1, 130.1, 129.1, 128.5, 128.4, 126.4, 126.0, 125.9, 124.9, 39.1, 33.2, 18.0, 12.9. These data are in agreement with literature data.[5]

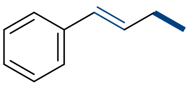

4af'

(*E*)-but-1-en-1-ylbenzene 4af': Prepared on a 0.5 mmol scale from but-3-en-1-ylbenzene **3af** according to the general catalytic procedure under optimized conditions. 83% NMR yield, *E*:*Z* 39:1. Purification by flash chromatography on silica gel using pentane as eluant yielded product as colorless oil (47.6 mg, 0.36 mmol, 72%). **^{1}H NMR** (400 MHz, $CDCl_3$, RT) δ 7.39 (d, 2H, J = 7.6 Hz), 7.33 (t, 2H, J = 7.6 Hz), 7.23 (t, 1H, J = 7.6 Hz), 6.43 (d, 1H, J = 15.8 Hz), 6.31 (dt, 1H, J =15.8, 6.3 Hz), 2.31-2.24 (m, 2H), 1.14 (t, 3H, J = 7.5 Hz). **^{13}C{^{1}H} NMR** (100 MHz, $CDCl_3$, RT) δ 138.1, 132.7, 128.9, 128.6, 126.9, 126.0, 26.2, 13.8. These data are in agreement with literature data.[29]

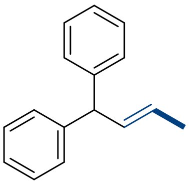

4ag

but-2-ene-1,1-diyldibenzene 4ag: Prepared on a 0.5 mmol scale from but-3-ene-1,1-diyldibenzene **3ag** according to the general catalytic procedure under optimized conditions. 91% NMR yield, *E*:*Z* 2.7:1. Purification by flash chromatography on silica gel using pentane as eluant yielded product as colorless oil (83.3 mg, 0.4 mmol, 80%). **^{1}H NMR** (400 MHz, $CDCl_3$, RT) δ 7.37-7.24 (m, 10H), 6.02-5.94 (m, 1H_{E+Z}), 5.79-5.71 (m, 0.3H_Z), 5.56-4.47 (m, 0.7H_E), 5.09 (d, 0.3H_Z, J = 9.6 Hz), 4.74 (d, 0.7H_E, J = 7.6 Hz), 1.80 (d, 3H, J = 6.6 Hz). **^{13}C{^{1}H} NMR** (100 MHz, $CDCl_3$, RT) δ 144.7, 144.4, 133.7, 133.1, 128.6, 128.5, 128.4, 127.1, 126.3, 124.8, 54.2, 48.2, 18.1, 13.2. These data are in agreement with literature data.[5]

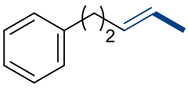

4ah

pent-3-en-1-ylbenzene 4ah: Prepared on a 0.5 mmol scale from pent-4-en-1-ylbenzene **3ah** according to the general catalytic procedure under optimized conditions. 88% NMR yield, *E*:*Z* 2.4:1. Purification by flash chromatography on silica gel using pentane as eluant yielded product as colorless oil (59.9 mg, 0.41 mmol, 82%). **^{1}H NMR** (400 MHz, $CDCl_3$, RT) δ 7.39-7.34(m, 2H), 7.29-7.24 (m, 3H), 5.57-5.53 (m, 2H), 2.77-2.73 (m, 2H), 2.48-2.43 (m, 0.7H_Z), 2.42-2.35 (m, 1.4H_E), 1.74-1.73 (m, 2.1H_E), 1.66-1.64 (m, 0.9H_Z). **^{13}C{^{1}H} NMR** (100 MHz, $CDCl_3$, RT) δ 142.3, 130.7, 129.7, 128.6, 128.4, 125.9, 125.8, 125.5, 124.6, 36.2, 36.9, 34.6, 28.9, 18.1, 12.8. These data are in agreement with literature data.[35]

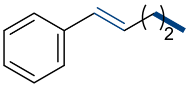

4ah'

(*E*)-pent-1-en-1-ylbenzene 4ah': Prepared on a 0.5 mmol scale from pent-3-en-1-ylbenzene **4ah** according to the general catalytic procedure using **2g** (50 mg, 0.1 mmol) and $Cy_2NH{\cdot}BH_3$ (19.6 mg, 0.1 mmol) and stirring for 48 h at 80 °C. 84% NMR

yield. *E*:*Z* >99:1.

Prepared on a 0.25 mmol scale from pent-4-en-1-ylbenzene **3ah** according to the general catalytic procedure using **2g** (25 mg, 0.05 mmol) and $Cy_2NH{\cdot}BH_3$ (9.8 mg, 0.05 mmol) and stirring for 48 h at 80 °C. 84% NMR yield. *E*:*Z* >99:1.

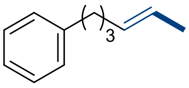

4ai

hex-4-en-1-ylbenzene 4ai: Prepared on a 0.5 mmol scale from hex-5-en-1-ylbenzene **3ai** according to the general catalytic procedure under optimized conditions. 90% NMR yield, *E*:*Z* 5.6:1. Purification by flash chromatography on silica gel using pentane as eluant yielded product as colorless oil (62.5 mg, 0.39 mmol, 78%). **^{1}H NMR** (400 MHz, $CDCl_3$, RT) δ 7.31-7.27 (m, 2H), 7.21 -7.18 (m, 3H), 5.54-5.39 (m, 2H), 2.66-2.60 (m, 2H), 2.13-2.01 (m, 2H), 1.74-1.60 (m, 5H). **^{13}C{^{1}H } NMR** (100 MHz, $CDCl_3$, RT) δ 142.8, 142.7, 131.2, 130.4, 128.6, 128.4, 125.8, 125.7, 125.3, 124.4, 35.6, 35.5, 32.3, 31.4, 26.6, 18.1, 13.0. These data are in agreement with literature data.[5]

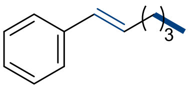

4ai'

(*E*)-hex-1-en-1-ylbenzene 4ai': Prepared on a 0.5 mmol scale from hex-5-en-1-ylbenzene **3ai** according to the general catalytic procedure using **2g** (50 mg, 0.1) and $Cy_2NH{\cdot}BH_3$ (19.6 mg, 0.1 mmol) and stirring for 48 h at 80 °C. 74% NMR yield. *E*:*Z* >99:1.

Catalytic procedures for Fe-catalyzed regio- and stereoconvergent isomerization of alkenes under optimized conditions

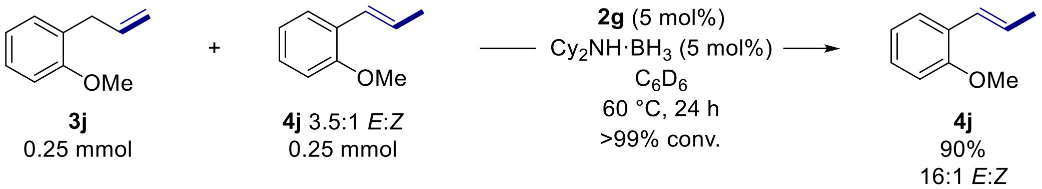

In an argon-filled glovebox, **2g** (12.5 mg, 0.025 mmol) was dissolved in benzene and added to an oven-dried 10 mL screw-capped tube, equipped with a magnetic stir bar, containing $Cy_2NH.BH_3$ (4.9 mg, 0.025 mmol). Next, a mixture of 1-allyl-2-methoxybenzene **3j** (37.1 mg, 0.25 mmol) and (*E*/*Z*)-1-methoxy-2-(prop-1-en-1-yl)benzene **4j** (*E*:*Z* 3.5:1) (37.1 mg, 0.25 mmol) was added and the reaction was stirred at 60 °C for 24 h. Upon completion, the reaction mixture was exposed to air and the crude was subjected to NMR analysis using 1,3,5-trimethoxybenzene (0.25 mmol) as internal standard. ^{1}H NMR analysis revealed 90% NMR yield and *E*:*Z* 16:1.

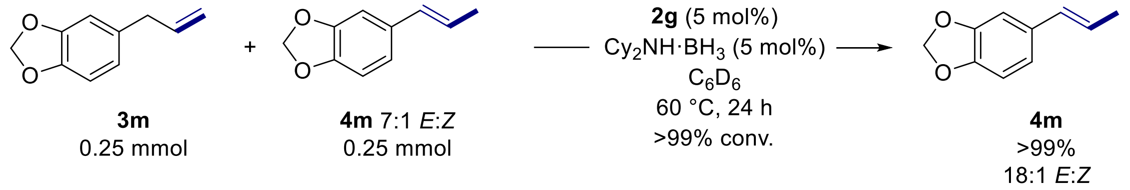

In an argon-filled glovebox, **2g** (12.5 mg, 0.025 mmol) was dissolved in benzene and added to an oven-dried 10 mL screw-capped tube, equipped with a magnetic stir bar, containing $Cy_2NH.BH_3$ (4.9 mg, 0.025 mmol). Next, a mixture of 5-allylbenzo[d][1,3]dioxole **3m** and (*E*/*Z*)-5-(prop-1-en-1-yl)benzo[d][1,3]dioxole **4m** (*E*:*Z* 7:1) (81.1 mg, 0.5 mmol) was added and the reaction was stirred at 60 °C for 24 h. Upon completion, the reaction mixture was exposed to air and the crude was subjected to NMR analysis using 1,3,5-trimethoxybenzene (0.25 mmol) as internal standard. ^{1}H NMR analysis revealed >99% NMR yield and *E*:*Z* 18:1.

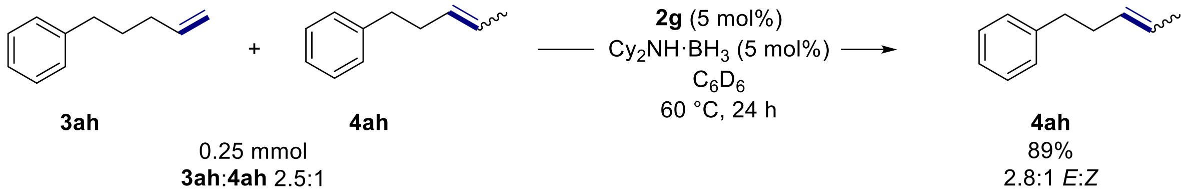

In an argon-filled glovebox, **2g** (6.2 mg, 0.0125 mmol) was dissolved in benzene and added to an oven-dried 10 mL screw-capped tube, equipped with a magnetic stir bar, containing $Cy_2NH.BH_3$ (2.4 mg, 0.0125 mmol). Next, a mixture of pent-4-en-1-ylbenzene **3ah** and (*E*/*Z*)-pent-3-en-1-ylbenzene **4ah** (*E*:*Z* n.d) (36.6 mg, 0.25 mmol) was added and the reaction was stirred at 60 °C for 24 h. Upon completion, the reaction mixture was exposed to air and the crude was subjected to NMR analysis using 1,3,5-trimethoxybenzene (0.25 mmol) as internal standard. ^{1}H NMR analysis revealed 89% NMR yield of **4ah** and *E*:*Z* 2.8:1.

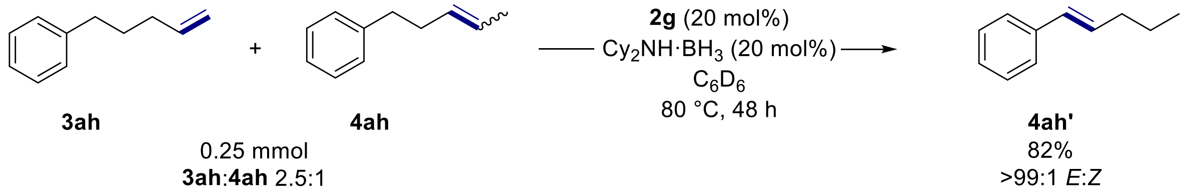

In an argon-filled glovebox, **2g** (25 mg, 0.05 mmol) was dissolved in benzene and added to an oven-dried 10 mL screw-capped tube, equipped with a magnetic stir bar, containing $Cy_2NH.BH_3$ (9.8 mg, 0.05 mmol). Next, a mixture of pent-4-en-1-ylbenzene **3ah** and (*E*/*Z*)-pent-3-en-1-ylbenzene **4ah** (*E*:*Z* n.d) (36.6 mg, 0.25 mmol) was added and the reaction was stirred at 80 °C for 48 h. Upon completion, the reaction mixture was exposed to air and the crude was subjected to NMR analysis using 1,3,5-trimethoxybenzene (0.25 mmol) as internal standard. ^{1}H NMR analysis revealed 82% NMR yield of **4ah'** and *E*:*Z* >99:1.

Limitations under optimized conditions:

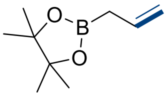

3aj

20% conv.

conditions:

5 mol% **2g**

5 mol% $Cy_2NH{\cdot}BH_3$

C_6D_6

60 °C, 24 h

Remark:

Decoloration of the solution upon addition of substrate

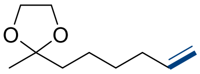

3ak

0% conv.

conditions:

5 mol% **2g**

5 mol% $Cy_2NH{\cdot}BH_3$

C_6D_6

60 °C, 24 h

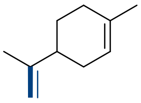

3al

51% conv.

unidentified products

conditions:

20 mol% **2g**

20 mol% $Cy_2NH{\cdot}BH_3$

C_6D_6

80 °C, 48 h

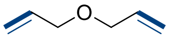

3am

18% conv.

(no isomerization product)

conditions:

5 mol% **2g**

5 mol% $Cy_2NH{\cdot}BH_3$

C_6D_6

60 °C, 24 h

Remark:

Control reaction using

3a (1 eq.) + 3am (1 eq.)

shows no conversion of **3a**

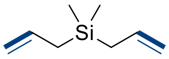

3an

61% (*E*,*E*)-*isomer*

+

31% monoisomerization

6:1 *E*:*Z*

conditions:

10 mol% **2g**

10 mol% $Cy_2NH{\cdot}BH_3$

C_6D_6

60 °C, 24 h

10-X-ray Single Crystal Data of Fe complexes

X-ray diffraction data for compounds **1f**, **2a**, **[2b]₂**, **2c**, **2f, 2g, 2h & 2i** were collected by using a VENTURE PHOTONIII CMOS Bruker diffractometer with Micro-focus IuS source Mo Kα radiation. Crystal was selected under a polarizing optical microscope and glued in paratone oil. Crystals were mounted on a CryoLoop (Hampton Research) with Paratone-N (Hampton Research) as cryoprotectant and then flashfrozen in a nitrogen-gas stream at 200 K or 100K. For compounds, the temperature of the crystal was maintained at the selected value by means of a 700+ series Cryostream cooling device to within an accuracy of ±1K. Data reduction was accomplished using SAINT V7.53a. The substantial redundancy in data allowed a semi-empirical absorption correction (SADABS V2.10) to be applied, on the basis of multiple measurements of equivalent reflections. The structures were solved by direct methods using SHELXS-97[36] and refined against F^2 by full-matrix least-squares techniques using SHELXL-2018[37] with anisotropic displacement parameters for all non-hydrogen atoms. Hydrogen atoms were introduced into the calculations as a riding model with isotropic thermal parameters. All calculations were performed by using the Crystal Structure crystallographic software package WINGX.[38]

The crystal data collection and refinement parameters are given in Table S8. Selected geometrical parameters are summarized in Tables S9-S14.

CCDC 2365925-2365930 & 2468334-2468335 contains the supplementary crystallographic data for this paper. These data can be obtained free of charge from the Cambridge Crystallographic Data Centre and Fachinformationszentrum Karlsruhe via http://www.ccdc.cam.ac.uk/structures/.

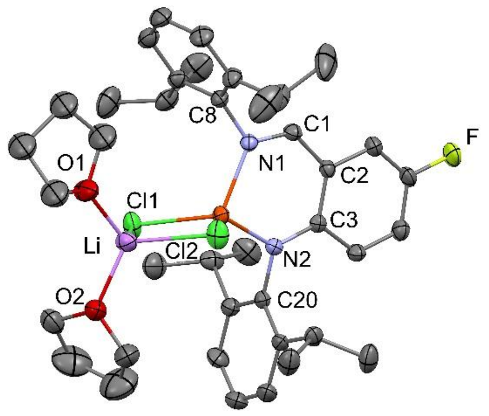

Figure S53. An ORTEP drawing of compound **1f**. Thermal ellipsoids are shown at the 30% level. (*hydrogen* atoms are omitted for clarity).

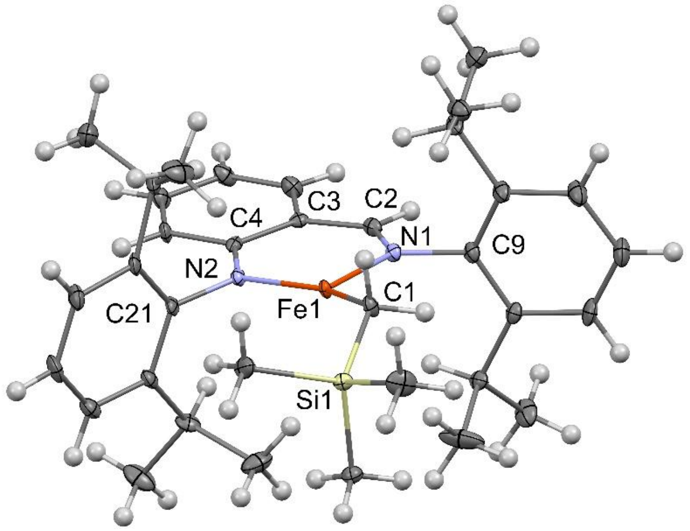

Figure S54. An ORTEP drawing of compound **2a**. Thermal ellipsoids are shown at the 30% level. Only one molecule of the asymmetric unit is depicted.

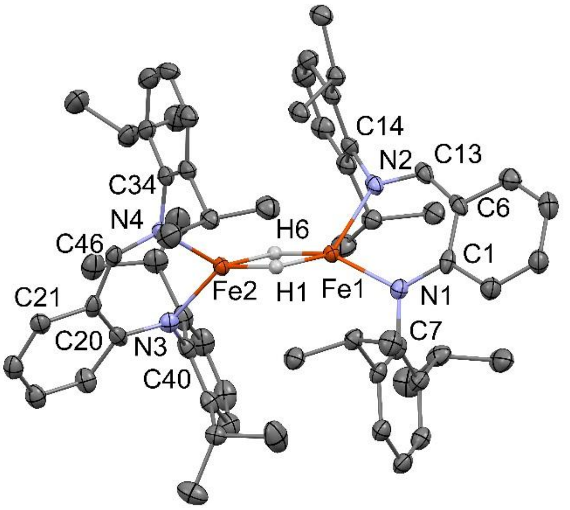

Figure S55. An ORTEP drawing of compound **[2b]$_2$**. Thermal ellipsoids are shown at the 30% level. *(*hydrogen atoms of ligands are omitted for clarity).

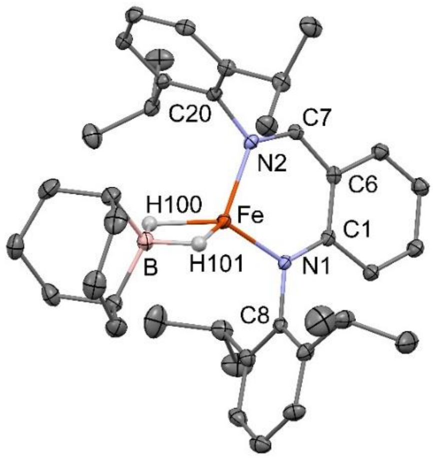

Figure S56. An ORTEP drawing of compound **2c**. Thermal ellipsoids are shown at the 30% level (all hydrogen atoms except those bonded to iron have been omitted for clarity)

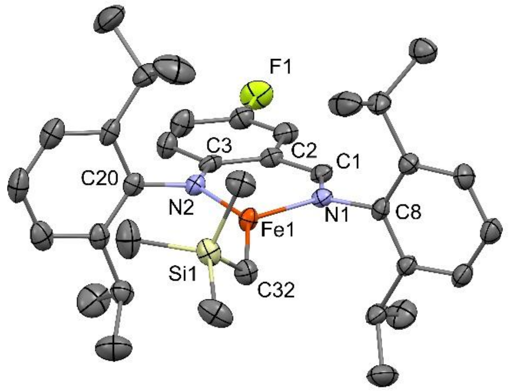

Figure S57. An ORTEP drawing of compound **2f**. Thermal ellipsoids are shown at the 30% level. (*hydrogen* atoms are omitted for clarity). Only *one molecule of the asymmetric unit* is depicted.

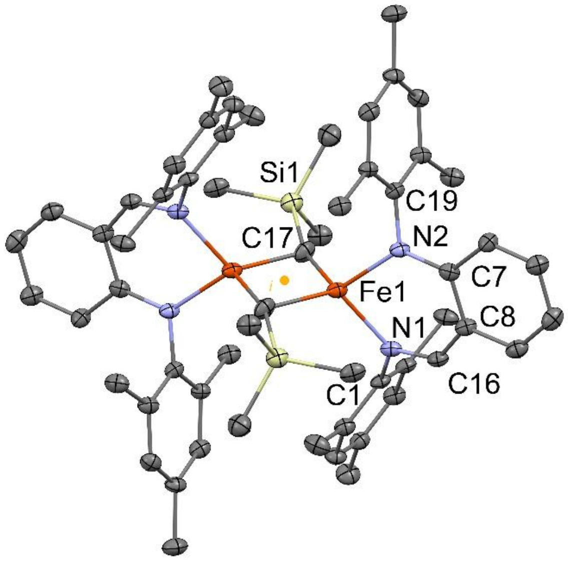

Figure S58. An ORTEP drawing of compound **2g**. Thermal ellipsoids are shown at the 30% level. (hydrogen atoms are omitted for clarity). Only one molecule of the asymmetric unit is depicted. (The inversion center is always located at the central point of the molecule).

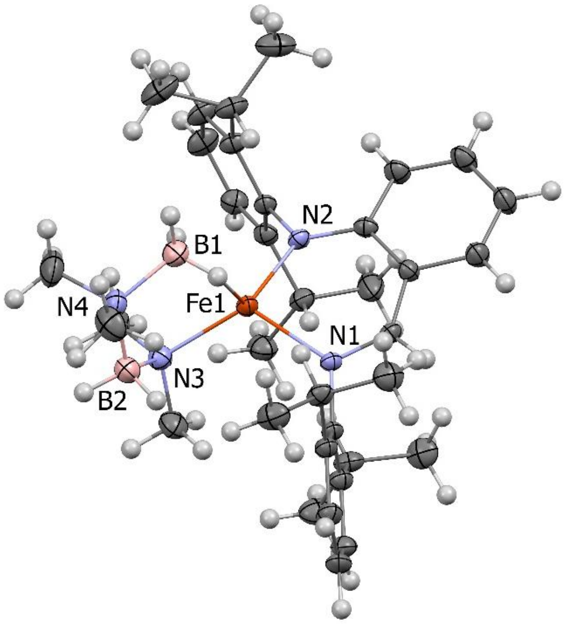

Figure S59. An ORTEP drawing of compound **2h**. Thermal ellipsoids are shown at the 30% level.

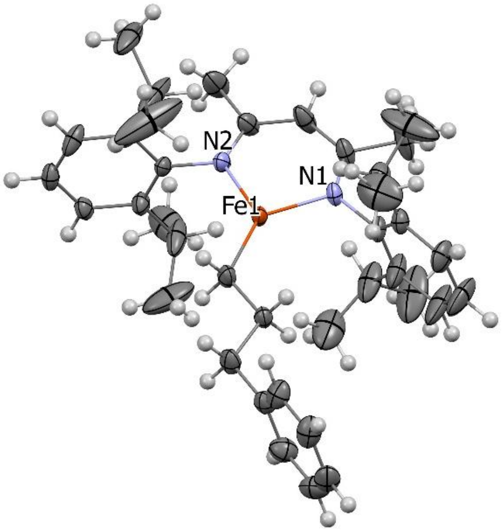

Figure S60. An ORTEP drawing of compound **2i**. Thermal ellipsoids are shown at the 30% level.

Table S8. Crystallographic data and structure refinement details.

Compound	1f	2a	$[2b]_2$
CCDC	2365929	2365927	2365925
Empirical Formula	C_{39} H_{54} Cl_2 F Fe Li N_2 O_2	C_{35} H_{50} Fe N_2 Si	C_{62} H_{80} Fe_2 N_4
M_r	735.53	582.71	993.00
Crystal size, mm^3	0.110 x 0.060 x 0.015	0.210 x 0.120 x 0.010	0.070 x 0.060 x 0.040
Crystal system	monoclinic	monoclinic	monoclinic
Space group	$P\ 2_1/n$	$P\ c$	$P\ 2_1/c$
a, Å	12.916(3)	9.8964(8)	11.4668(10)
b, Å	17.668(4)	19.8085(17)	24.770(2)
c, Å	18.468(4)	17.3153(15)	19.2894(16)
α, °	90	90	90
β, °	101.496(8)	102.238(3)	92.751(3)
γ, °	90	90	90
Cell volume, Å^3	4129.7(15)	3317.2(5)	5472.4(8)
Z ; Z'	4 ; 1	4 ; 2	4 ; 1
T, K	200(1)	100(1)	100(1)
Radiation type ; wavelength Å	MoKα ; 0.71073	MoKα ; 0.71073	MoKα ; 0.71073
F_{000}	1560	1256	2128
μ, mm^{-1}	0.531	0.515	0.568
range, °	2.112 - 30.557	2.106 - 31.106	2.185 - 26.464
Reflection collected	251 102	173 810	101 902
Reflections unique	12 628	21 222	11 267
R_{int}	0.1712	0.0492	0.0904
GOF	0.923	1.069	1.039
Refl. obs. (I>2(I))	4 705	17 692	7 147
Parameters ; Restraints	436 ; 3	726 ; 2	637 ; 0
wR_2 (all data) [b]	0.1502	0.1775	0.1724
R_1 value (I>2(I)) [a]	0.0603	0.0661	0.0674
Largest diff. peak and hole (e-.Å^{-3})	0.371 ; -0.336	3.485 ; -0.560	0.788 ; -0.300

**Compound**	**2c**	**2f**	**2g**
CCDC	2365928	2365926	2365930
Empirical Formula	C_{39} H_{55} B Fe N_2	C_{35} H_{49} F Fe N_2 Si	C_{29} H_{38} Fe N_2 Si
M_r	618.51	600.70	498.55
Crystal size, mm^3	0.120 x 0.090 x 0.025	0.090 x 0.070 x 0.020	0.060 x 0.040 x 0.030
Crystal system	monoclinic	monoclinic	triclinic
Space group	$P\ 2_1/n$	$P\ 2_1/c$	P -1
a, Å	10.8925(7)	11.5731(6)	10.9974(3)
b, Å	15.6042(9)	32.7794(16)	13.0651(4)
c, Å	20.7845(12)	18.7822(9)	19.7757(7)
α, °	90	90	89.750(2)
β, °	91.567(2)	103.924(2)	75.0667(19)
γ, °	90	90	77.775(2)
Cell volume, $Å^3$	3531.4(4)	6915.8(6)	2679.40(15)
Z ; Z'	4 ; 1	8 ; 2	4 ; 2
T, K	100(1)	200(1)	100(1)
Radiation type ; wavelength Å	MoKα ; 0.71073	MoKα ; 0.71073	MoKα ; 0.71073
F_{000}	1336	2576	1064
μ, mm^{-1}	0.455	0.500	0.626
range, °	2.088 - 30.601	1.813 - 31.030	2.135 - 30.671
Reflection collected	92 217	375 040	180 023
Reflections unique	10 795	22 059	16 490
R_{int}	0.0647	0.2233	0.2336
GOF	1.092	0.893	1.010
Refl. obs. (I>2(I))	7 718	7 396	6 247
Parameters ; Restraints	402 ; 0	743 ; 0	613 ; 0
wR_2 (all data) [b]	0.2465	0.2213	0.2116
R_1 value (I>2(I)) [a]	0.0927	0.0646	0.0796
Largest diff. peak and hole (e-.$Å^{-3}$)	3.527 ; -0.498	0.552 ; -0.323	0.794 ; -0.659

[a]R1 = Σ||F0| – |Fc||/Σ|F0|. [b]wR2 = $[\Sigma w(F_o^2 - F_c^2)^2/\Sigma(F_o^2)^2]^{1/2}$

**Compound**	**2h**	**2i**
CCDC	2468335	2468334
Empirical Formula	$C_{35} H_{56} B_2 Fe N_4$	$C_{38} H_{52} Fe N_2$
M_r	610.30	592.66
Crystal size, mm^3	0.110 x 0.020 x 0.015	0.14 x 0.08 x 0.04
Crystal system	orthorhombic	monoclinic
Space group	*P b c a*	*C 2/c*
a, Å	11.844(5)	36.2250(18)
b, Å	15.304(5)	9.3645(4)
c, Å	38.740(14)	22.7548(8)
α, °	90	90
β, °	90	113.7180(10)
γ, °	90	90
Cell volume, Å^3	7022(5)	7067.1(5)
Z ; Z'	8 ; 1	8 ; 1
T, K	200(1)	200(1)
Radiation type ; wavelength Å	MoKα ; 0.71073	MoKα ; 0.71073
F_{000}	2640	2560
μ, mm^{-1}	0.455	0.450
range, °	2.103-30.534	2.260 - 30.517
Reflection collected	185 228	206 242
Reflections unique	10 727	10 773
R_{int}	0.2663	0.0502
GOF	0.961	1.026
Refl. obs. (I>2(I))	3 888	8 124
Parameters ; Restraints	392 , 0	462 ; 0
wR_2 (all data) [b]	0.1670	0.1951
R_1 value (I>2(I)) [a]	0.0653	0.0670
Largest diff. peak and hole (e-.Å^{-3})	0.573 ; -0.305	1.185 ; -0.976

Table S9. Selected bond lengths [Å] and angles [°] for complex **1f** with esd's in parenthesis (all esds are estimated using the full covariance matrix

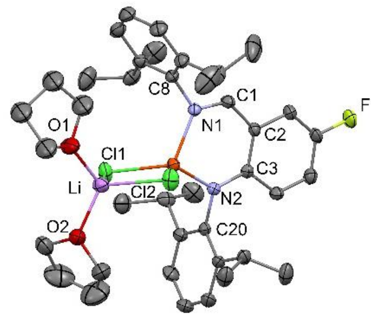

Fe-N(1)	2.015(2)	Fe-Cl(1)	2.3182(9)
Fe-N(2)	1.966(2)	Fe-Cl(2)	2.3117(10)
N(1)-Fe-N(2)	91.51(9)	N(1)-Fe-Cl(1)	114.47(6)
N(1)-Fe-Cl(2)	111.83(7)	N(2)-Fe-Cl(1)	121.20(6)
N(2)-Fe-Cl(2)	121.47(7)	Cl(1)-Fe-Cl(2)	97.34(3)
N(1)-C(8)	1.438(3)	N(1)-C(1)	1.308(3)
C(1)-C(2)	1.419(4)	C(2)- C(3)	1.422(3)
C(3)-N(2)	1.363(3)	N(2)-C(20)	1.439(3)
C(8)-N(1)-C(1)	117.1(2)	C(20)-N(2)-C(3)	117.9(2)

Table S10. Selected bond lengths [Å] and angles [°] for complex **2a** with esd's in parenthesis (all esds are estimated using the full covariance matrix

Fe(1) molecule			Fe(2) molecule	
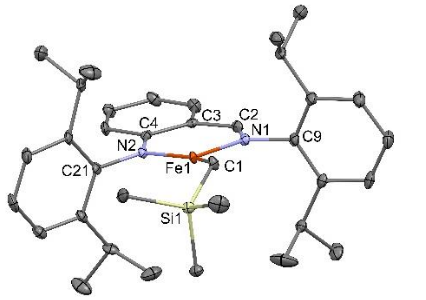			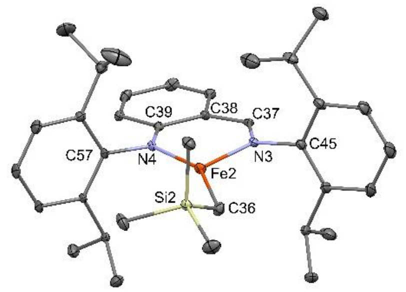	
Fe(1)-N(1)	2.027(4)		Fe(2)-N(3)	2.017(4)
Fe(1)-N(2)	1.952(4)		Fe(2)-N(4)	1.961(4)
Fe(1)-C(1)	2.029(5)		Fe(2)-C(36)	2.029(5)
N(1)-Fe(1)-N(2)	91.84(17)		N(3)-Fe(2)-N(4)	92.31(16)
N(2)-Fe(1)-C(1)	150.31(18)		N(4)-Fe(2)-C(36)	150.39(18)
N(1)-Fe(1)-C(1)	117.10(17)		N(3)-Fe(2)-C(36)	116.50(18)
N(1)-C(9)	1.450(6)		N(3)-C(45)	1.445(6)
C(2)-C(3)	1.419(7)		C(37)-C(38)	1.421(6)
C(4)-N(2)	1.376(6)		C(39)-N(4)	1.361(6)
N(1)-C(2)	1.292(6)		N(3)-C(37)	1.305(6)
C(3)-C(4)	1.433(7)		C(38)-C(39)	1.434(6)
N(2)-C(21)	1.433(6)		N(4)-C(39)	1.361(6)
C(9)-N(1)-C(2)	117.4(4)		C(45)-N(3)-C(37)	117.7(4)
C(21)-N(2)-C(4)	119.0(4)		C(57)-N(4)-C(39)	119.3(4)

Table S11. Selected bond lengths [Å] and angles [°] for complex **[2b]$_2$** with esd's in parenthesis (all esds are estimated using the full covariance matrix

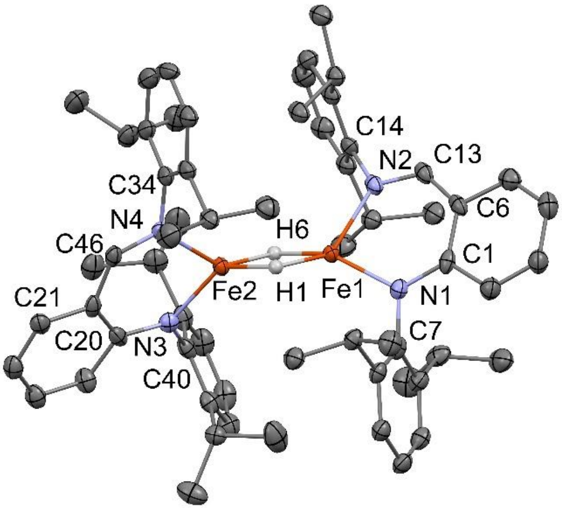

Fe(1)-Fe(2) = 2.6659(8) Å

Fe(1)-N(1)	1.969(3)	Fe(2)-N(3)	1.968(4)
Fe(1)-N(2)	2.035(3)	Fe(2)-N(4)	2.017(3)
Fe(1)-H(1)	1.67(4)	Fe(2)-H(1)	1.66(4)
Fe(1)-H(6)	1.72(4)	Fe(2)-H(6)	1.66(4)
N(1)-Fe(1)-N(2)	91.55(14)	N(3)-Fe(2)-N(4)	91.43(14)
N(1)-Fe(1)-H(1)	119.9(14)	N(3)-Fe(2)-H(1)	135.9(15)
N(1)-Fe(1)-H(6)	151.2(13)	N(3)-Fe(2)-H(6)	113.7(14)
N(2)-Fe(1)-H(1)	135.8(14)	N(4)-Fe(2)-H(1)	104.5(15)
N(2)-Fe(1)-H(6)	103.4(13)	N(4)-Fe(2)-H(6)	144.1(14)
H(1)-Fe(1)-H(6)	74(2)	H(1)-Fe(2)-H(6)	76(2)
N(1)-C(7)	1.426(5)	N(3)-C(40)	1.428(6)
N(1)-C(1)	1.378(5)	N(3)-C(20)	1.368(5)
C(1)-C(6)	1.439(6)	C(20)-C(21)	1.422(6)
C(6)-C(13)	1.389(6)	C(21)-C(46)	1.402(6)
C(13)-N(2)	1.297(5)	C(46)-N(4)	1.314(5)
N(2)-C(14)	1.447(5)	N(4)-C(34)	1.447(6)
C(7)-N(1)-C(1)	117.6(3)	C(40)-N(3)-C(20)	118.7(4)
C(14)-N(2)-C(13)	115.9(4)	C(34)-N(4)-C(46)	114.0(3)

The hydride ligands in complex **[2b]$_2$** (H6 & H1) were located in a difference Fourier synthesis and refined with a isotropic displacement parameter.

Table S12. Selected bond lengths [Å] and angles [°] for complex **2c** with esd's in parenthesis (all esds are estimated using the full covariance matrix

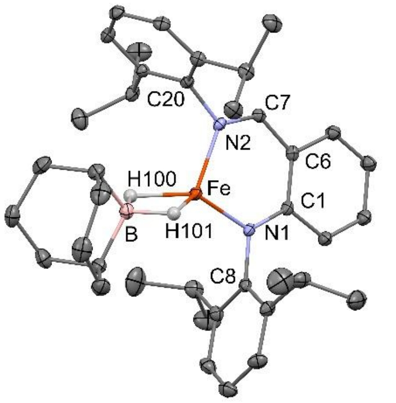

Fe-N(1)	1.937(3)	Fe-H(100)	1.68(4)
Fe-N(2)	1.983(3)	Fe-H(101)	1.72(4)
N(1)-Fe-N(2)	94.18(12)	N(1)-Fe- H(100)	133.5(15)
N(1)-Fe- H(101)	122.8(15)	N(2)-Fe- H(100)	110.1(15)
N(2)-Fe- H(101)	128.4(14)	H(100)-Fe- H(101)	71(2)
Fe-B	2.180(4)		
N(1)-C(8)	1.439(4)	N(1)-C(1)	1.362(4)
C(1)-C(6)	1.432(5)	C(6)- C(7)	1.427(5)
C(7)-N(2)	1.298(4)	N(2)-C(20)	1.445(4)
C(8)-N(1)-C(1)	120.8(3)	C(20)-N(2)-C(7)	120.9(3)

The hydrogen atoms in **2c** (H100 & H101) on iron were refined freely. They were located in a difference Fourier map and refined with an isotropic displacement parameter

Table S13. Selected bond lengths [Å] and angles [°] for complex **2f** with esd's in parenthesis (all esds are estimated using the full covariance matrix

Fe(1) molecule			Fe(2) molecule	

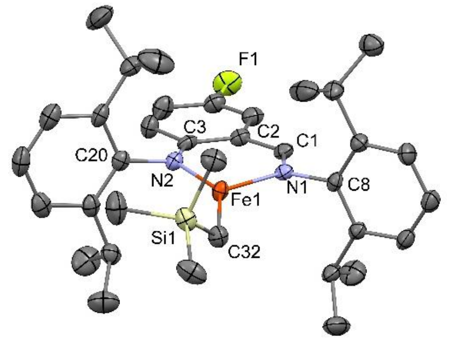

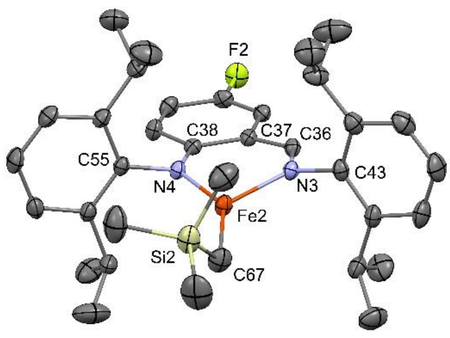

Fe(1)-N(1)	2.015(2)		Fe(2)-N(3)	2.022(2)
Fe(1)-N(2)	1.959(2)		Fe(2)-N(4)	1.953(2)
Fe(1)-C(32)	2.012(3)		Fe(2)-C(67)	2.008(3)
N(1)-Fe(1)-N(2)	92.36(9)		N(3)-Fe(2)-N(4)	92.04(9)
N(2)-Fe(1)-C(32)	140.85(12)		N(4)-Fe(2)-C(67)	140.65(12)
N(1)-Fe(1)-C(32)	125.71(11)		N(3)-Fe(2)-C(67)	127.08(12)
N(1)-C(8)	1.457(3)		N(3)-C(43)	1.448(3)
C(1)-C(2)	1.402(4)		C(36)-C(37)	1.429(3)
C(3)-N(2)	1.368(3)		C(38)-N(4)	1.367(3)
N(1)-C(1)	1.309(3)		N(3)-C(36)	1.302(3)
C(3)-C(2)	1.425(4)		C(38)-C(37)	1.428(4)
N(2)-C(20)	1.440(4)		N(4)-C(55)	1.440(3)
C(8)-N(1)-C(1)	116.2(2)		C(43)-N(3)-C(36)	116.6(2)
C(20)-N(2)-C(3)	119.3(2)		C(55)-N(4)-C(38)	117.7(2)

Table S14. Selected bond lengths [Å] and angles [°] for complex **2g** with esd's in parenthesis (all esds are estimated using the full covariance matrix

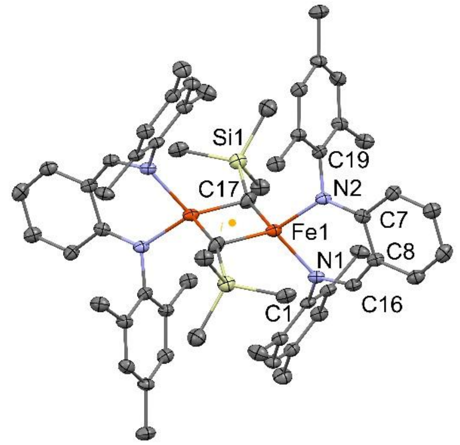

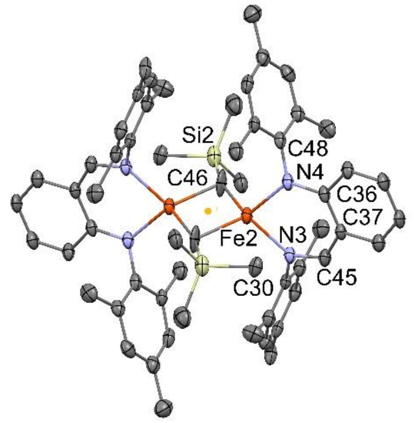

Fe(1) molecule			Fe(2) molecule	
Fe(1)-Fe(1)^{i}= 2.7071(12) Å			Fe(2)-Fe(2)^{j}=2.7962(12) Å	
Fe(1)-N(1)	2.053(3)		Fe(2)-N(3)	2.052(4)
Fe(1)-N(2)	2.001(3)		Fe(2)-N(4)	2.002(3)
Fe(1)-C(17)	2.103(5)		Fe(2)-C(46)	2.082(5)
Fe(1)-C(17)^{i}	2.258(4)		Fe(2)-C(46)^{j}	2.247(5)
N(1)-Fe(1)-N(2)	90.68(14		N(3)-Fe(2)-N(4)	89.94(15)
N(1)-Fe(1)-C(17)	124.22(16)		N(3)-Fe(2)-C(46)	117.4(2)
N(1)-Fe(1)-C(17)^{i}	101.73(15)		N(3)-Fe(2)-C(46)^{j}	111.62(18)
N(2)-Fe(1)-C(17)	120.42(16)		N(4)-Fe(2)-C(46)	128.66(19)
N(2)-Fe(1)-C(17)^{i}	115.66(16)		N(4)-Fe(2)-C(46)^{j}	109.7(2)
C(17)-Fe(1)-C(17)^{i}	103.35(15)		C(46)-Fe(2)-C(46)^{j}	99.6(2)
N(1)-C(1)	1.465(5)		N(3)-C(30)	1.459(5)
C(16)-C(8)	1.432(5)		C(45)-C(37)	1.435(6)
C(7)-N(2)	1.363(5)		C(36)-N(4)	1.378(5)
N(1)-C(16)	1.296(5)		N(3)-C(45)	1.308(5)
C(7)-C(8)	1.437(5)		C(36)-C(37)	1.421(6)
N(2)-C(19)	1.443(5)		N(4)-C(48)	1.435(6)
C(1)-N(1)-C(16)	115.2(3)		C(30)-N(3)-C(45)	113.0(4)
C(19)-N(2)-C(7)	115.0(3)		C(48)-N(4)-C(36)	116.2(3)

structure is centrosymmetric with two inversion centers between atoms Fe(1)/Fe(1)^{i} (i: -x,2-y,1-z) and Fe(2)/Fe(2)^{j} (j: 2-x,1-y,-z)

12-References

[1] K. E. Kawamura, A. S. Chang, D. J. Martin, H. M. Smith, P. T. Morris, A. K. Cook *Organometallics* **2022**, *41*, 486−496.

[2] P. M. Paduraru, R. T. W. Popoff, R. Nair, R. Gries, G. Gries, E. Plettner, *J. Comb. Chem*. **2008,** *10*, 123–134.

[3] C. E. Sear, P. Pieper, M. Amaral, M. M. Romanelli, T. A. Costa-Silva, M. M. Haugland, J. A. Tate, J. H. G. Lago, A. G. Tempone, E. A. Anderson, *ACS Infect. Dis*. **2020**, *6*, 2872−2878.

[4] Z. Wu, J. Meng, H. Liu, Y. Li, X. Zhang, W. Zhang, *Nat. Chem.* **2023**, *15*, 988–997.

[5] Q. -Y. Meng, T. E. Schirmer, K. Katou, B. König, *Angew.Chem. Int.Ed.* **2019**, *58*, 5723–5728.

[6] a) H. Jiang, H. Gao, B. Liu and W. Wu, *Chem. Commun*. **2014**, *50*, 15348–15351; b) D. Kim, G. Pillon, D. J. DiPrimio, P. L. Holland, *J. Am. Chem. Soc.* **2021**, *143*, 3070−3074.

[7] G. Liu, J. M. Wurst, D. S. Tan, *Org. Lett.* **2009**, *11*, 3670–3673.

[8] C. R. Woof, D. J. Durand, N. Fey, E. Richards, R. L. Webster, *Chem*. *Eur*. *J*. **2021**, *27*, 5972 –5977.

[9] D. Chen, C. Lepori, R. Guillot, R. Gil, S. Bezzenine, J. Hannedouche, *Angew. Chem. Int. Ed*. **2024**, *63*, e202408419.

[10] N. T. Coles, M. F. Mahon, R. L. Webster, *Organometallics* **2017**, *36*, 2262−2268.

[11] P.V. Ramachandran, H. J. Hamann, S. Mishra, *ACS Omega* **2022**, *7*, 14377−14389.

[12] K. K. Wang, H. C. Brown, *J. Am. Chem. Soc*. **1982**, *104*, 7148-7155.

[13] J. Burés, *Angew. Chem. Int. Ed*. **2016**, *55*, 2028 –2031.

[14] J. Burés, *Angew. Chem. Int. Ed*. **2016**, *55*, 16084–16087.

[15] J. M. Smith, R. J. Lachicotte, P. L. Holland, *J. Am. Chem. Soc.* **2003**, *125*, 15752–15753.

[16] M. J. Frisch, G. W. Trucks, H. B. Schlegel, G. E. Scuseria, M. A. Robb, J. R. Cheeseman, G. Scalmani, V. Barone, G. A. Petersson, H. Nakatsuji, X. Li, M. Caricato, A. V. Marenich, J. Bloino, B. G. Janesko, R. Gomperts, B. Mennucci, H. P. Hratchian, J. V. Ortiz, A. F. Izmaylov, J. L. Sonnenberg, D. Williams-Young, F. Ding, F. Lipparini, F. Egidi, J. Goings, B. Peng, A. Petrone, T. Henderson, D. Ranasinghe, V. G. Zakrzewski, J. Gao, N. Rega, G. Zheng, W. Liang, M. Hada, M. Ehara, K. Toyota, R. Fukuda, J. Hasegawa, M. Ishida, T. Nakajima, Y. Honda, O. Kitao, H. Nakai, T. Vreven, K. Throssell, J. A. Montgomery Jr, J. E. Peralta, F. Ogliaro, M. J. Bearpark, J. J. Heyd, E. N. Brothers, K. N. Kudin, V. N. Staroverov, T. A. Keith, R. Kobayashi, J. Normand, K. Raghavachari, A. P. Rendell, J. C. Burant, S. S. Iyengar, J. Tomasi, M. Cossi, J. M. Millam, M. Klene, C. Adamo, R. Cammi, J. W. Ochterski, R. L. Martin, K. Morokuma, O. Farkas, J. B. Foresman, and D. J. Fox, Gaussian 16, Revision B.01, Gaussian, Inc., Wallingford CT, 2016.

[17] M. Espinal-Viguri, S. E. Neale, N. T. Coles, S. A. Macgregor, R. L. Webster, *J. Am. Chem. Soc.* **2019**, *141*, 572−582.

[18] A. D. Becke, *Physical Review A* **1988**, *38*, 3098-3100.

[19] J. P. Perdew, *Physical Review B* **1986**, *33*, 8822-8824.

[20] M. Dolg, U. Wedig, H. Stoll, H. Preuss, *J. Chem. Phys.***1987**, *86*, 866-872.

[21] W. J. Hehre, R. Ditchfield, J. A. Pople, *J. Chem. Phys.*, **1972**, *56*, 2257-2261.

[22] P. C. Hariharan, J. A. Pople, *Theor. Chem. Acc.* **1973**, *28***,** 213-222. .

[23] J. Tomasi, B. Mennucci, R. Cammi, *Chem. Rev*. **2005**, *105*, 2999-3094.

[24] A. D. Becke, *J. Chem. Phys.* **1993,** *98*, 5648-5652.

[25] S. Grimme, J. Antony, S. Ehrlich, H. Krieg, *J. Chem. Phys.* **2010**, *132*, 154104-154119.

[26] A. Schaefer, C. Huber, R. Ahlrichs, *J. Chem. Phys.* **1994**, *100*, 5829-5835.

[27] Jaime Rodríguez-Guerra. (2020). jaimergp/easymecp: v0.3.2 (v0.3.2). Zenodo. https://doi.org/10.5281/zenodo.4293422

[28] O. Kahn, in *Molecular Magnetism*, VCH-Verlag, Weinheim, **1993**.

[29] N. Zhu, J. Zhao, H. Bao, *Chem. Sci*. **2017**, *8*, 2081-2085.

[30] K. Nakayama, N. Maeta, G. Horiguchi, H. Kamiya, Y. Okada, *Org. Lett*. **2019**, *21*, 2246–2250.

[31] A. Kapat, T. Sperger, S. Guven, F. Schoenebeck, *Science* **2019** *363*, 391–396.

[32] M. Petsi, M. Orfanidou, A. Zografos, *Green Chem*. **2021**, *23*, 9172-9178.

[33] R. Jennerjahn, R. Jackstell, I. Piras, R. Franke, H. Jiao, M. Bauer, M. Beller, *ChemSusChem* **2012**, *5*, 734 – 739.

[34] R. Bandari, T. Höche, A. Prager, K. Dimberger, M. R. Buchmeiser, *Chem. Eur. J*. **2010**, *16*, 4650–4658.

[35] X. Yu, H. Zhao, P. Li, M. Joo Koh , *J. Am. Chem. Soc*. **2020**, *142*, 18223−18230.

[36] G. M. Sheldrick, SHELXS-97, Program for Crystal Structure Solution, University of Göttingen, Göttingen, Germany, **1997**.

[37] G. M. Sheldrick, *Acta Crystallogr., Sect. A: Found. Crystallogr.,* **2008**, *64*, 112-122.

[38] L. J. Farrugia, *J. Appl. Cryst*. **1999**, *32*, 837.

13-NMR spectra

^{1}H NMR spectrum of (*E*)-*N*-(2-fluorobenzylidene)-2,6-diisopropylaniline (**I-2a**) (400 MHz, $CDCl_3$, RT)

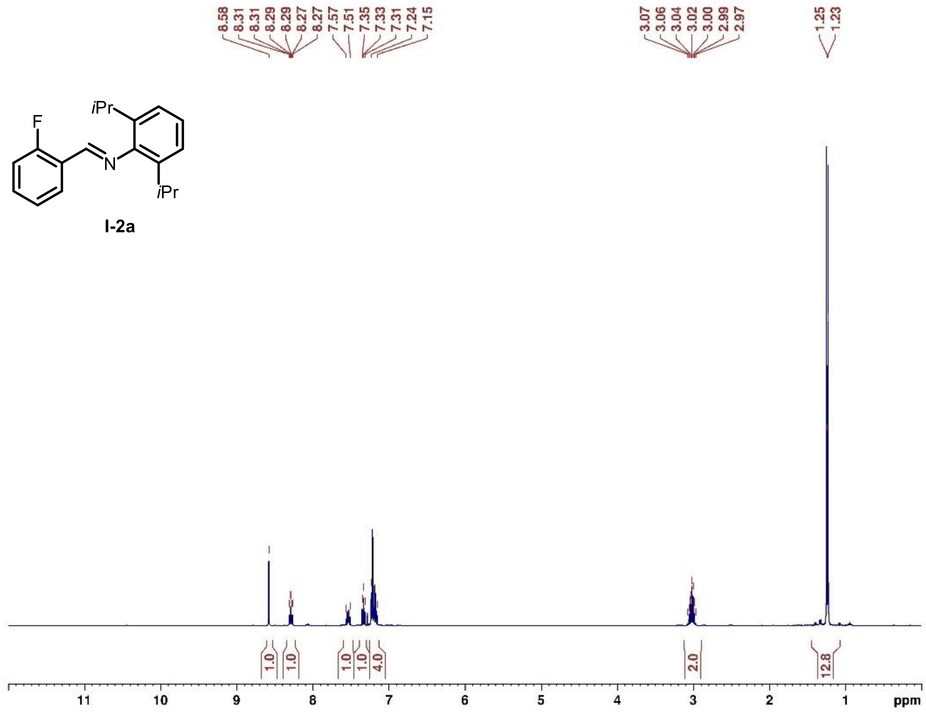

^{13}C NMR spectrum of (*E*)-*N*-(2-fluorobenzylidene)-2,6-diisopropylaniline (**I-2a**) (100 MHz, $CDCl_3$, RT)

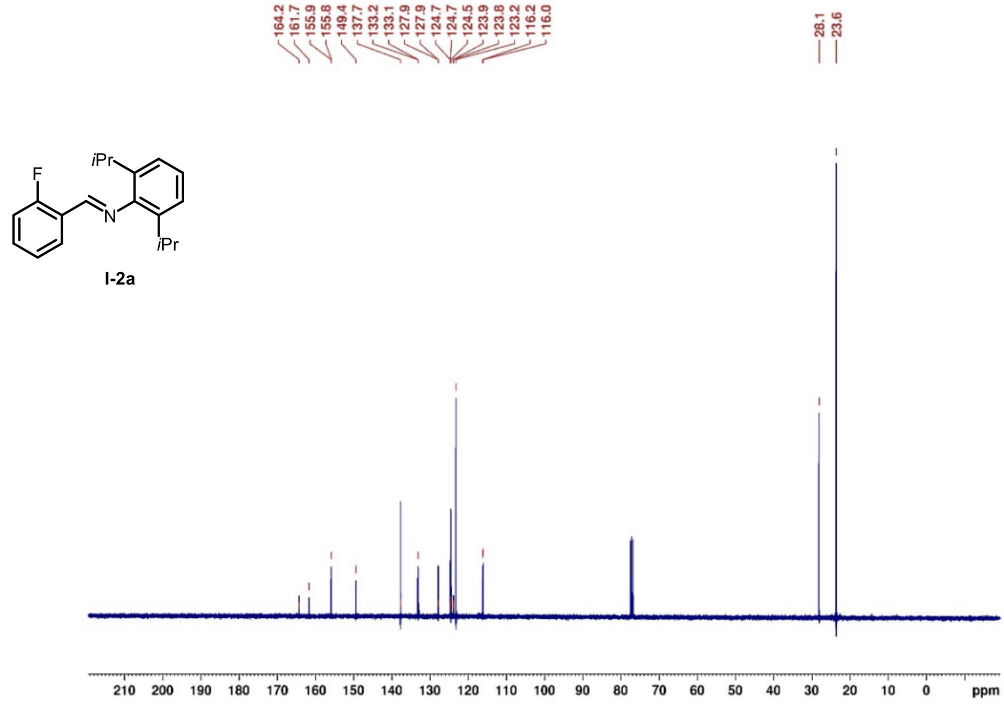

^{1}H NMR spectrum of (*E*)-*N*-(2-((2,6-diisopropylphenyl)amino)benzylidene)-2,6-diisopropylaniline (**AA-2a**) (400 MHz, $CDCl_3$, RT)

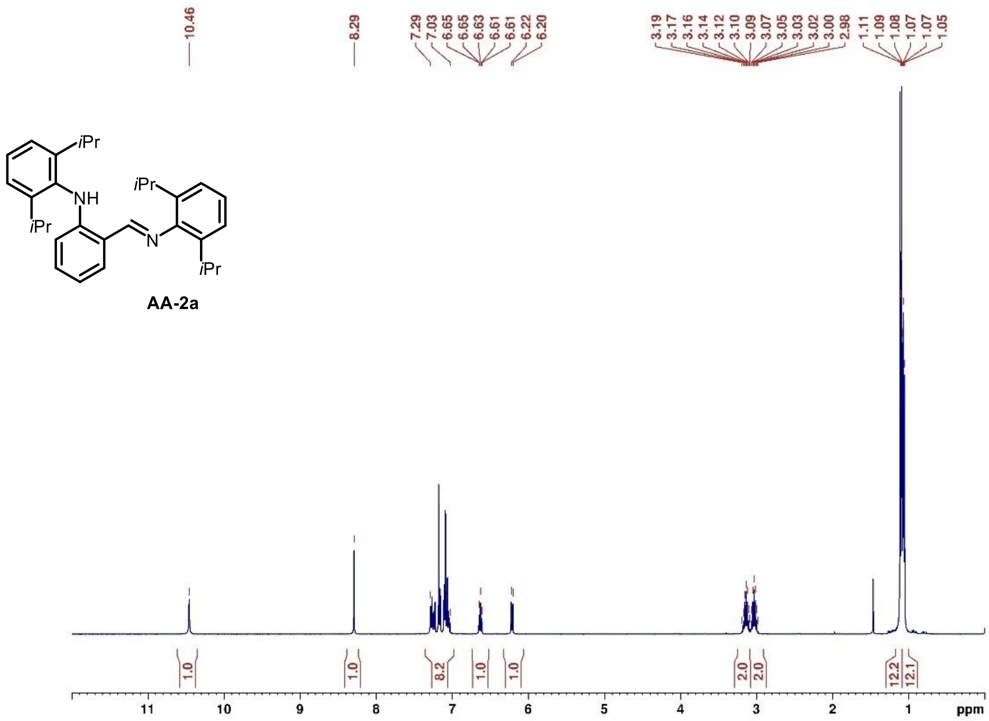

^{13}C NMR spectrum of (*E*)-*N*-(2-((2,6-diisopropylphenyl)amino)benzylidene)-2,6-diisopropylaniline (**AA-2a**) (100 MHz, $CDCl_3$, RT)

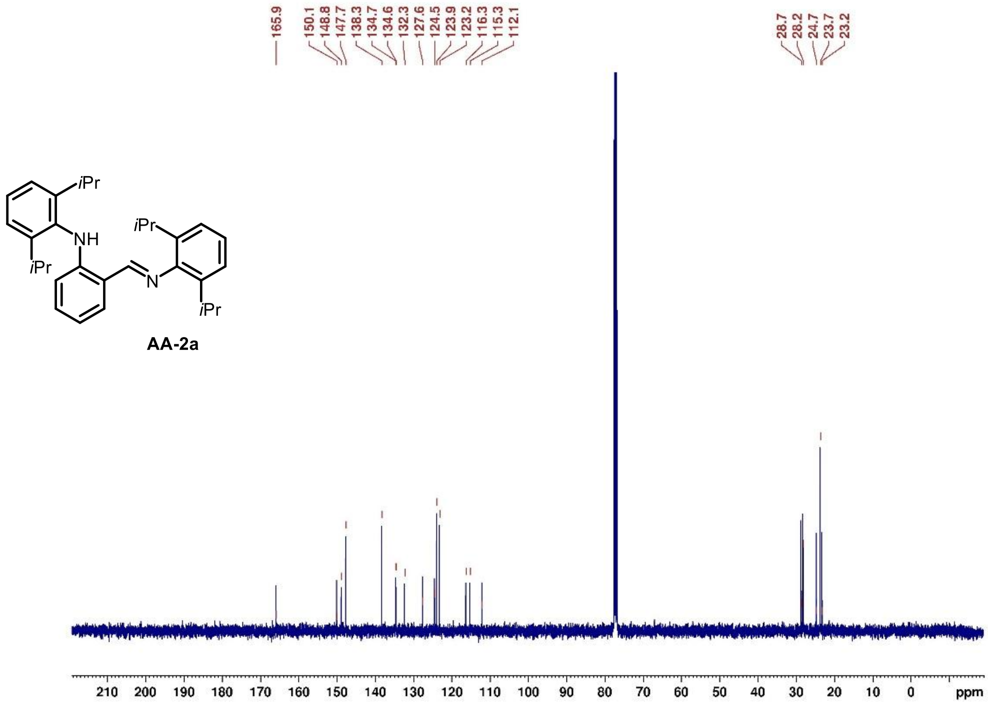

^{1}H NMR spectrum of (*E*)-*N*-(2-fluoro-4-methoxybenzylidene)-2,6-diisopropylaniline (**I-2d**) (400 MHz, $CDCl_3$, RT)

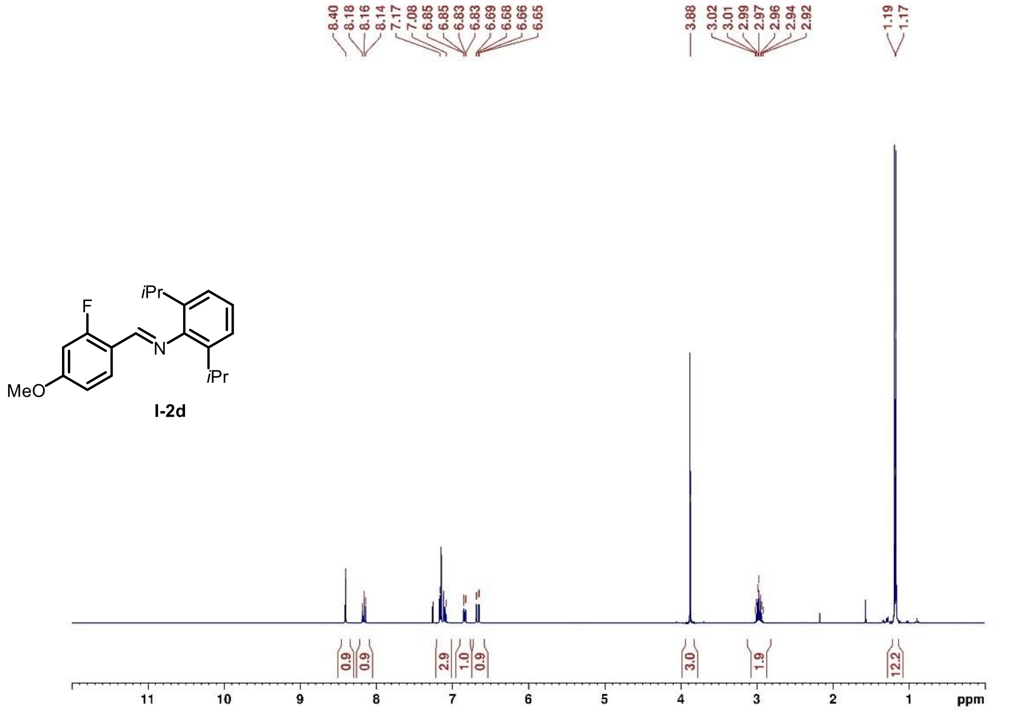

^{13}C NMR spectrum of (*E*)-*N*-(2-fluoro-4-methoxybenzylidene)-2,6-diisopropylaniline (**I-2d**) (100 MHz, $CDCl_3$, RT)

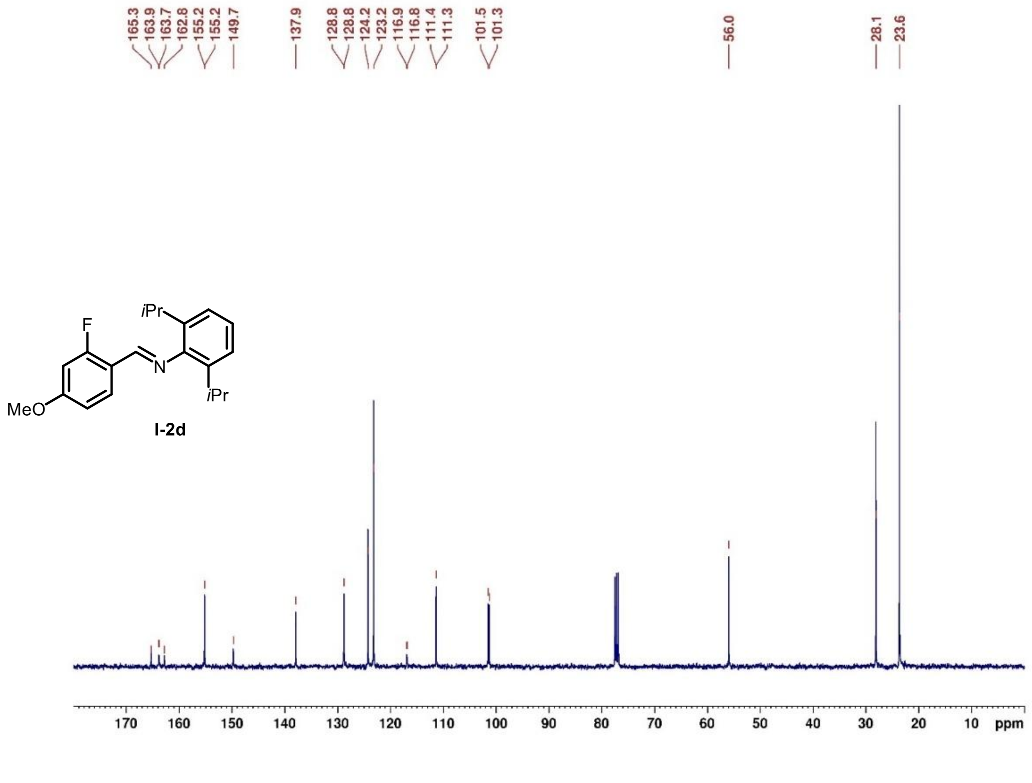

^{1}H NMR spectrum of (*E*)-*N*-(2,6-diisopropylphenyl)-2-(((2,6-diisopropylphenyl)imino)methyl)-5-methoxyaniline (**AA-2d**) (400 MHz, $CDCl_3$, RT)

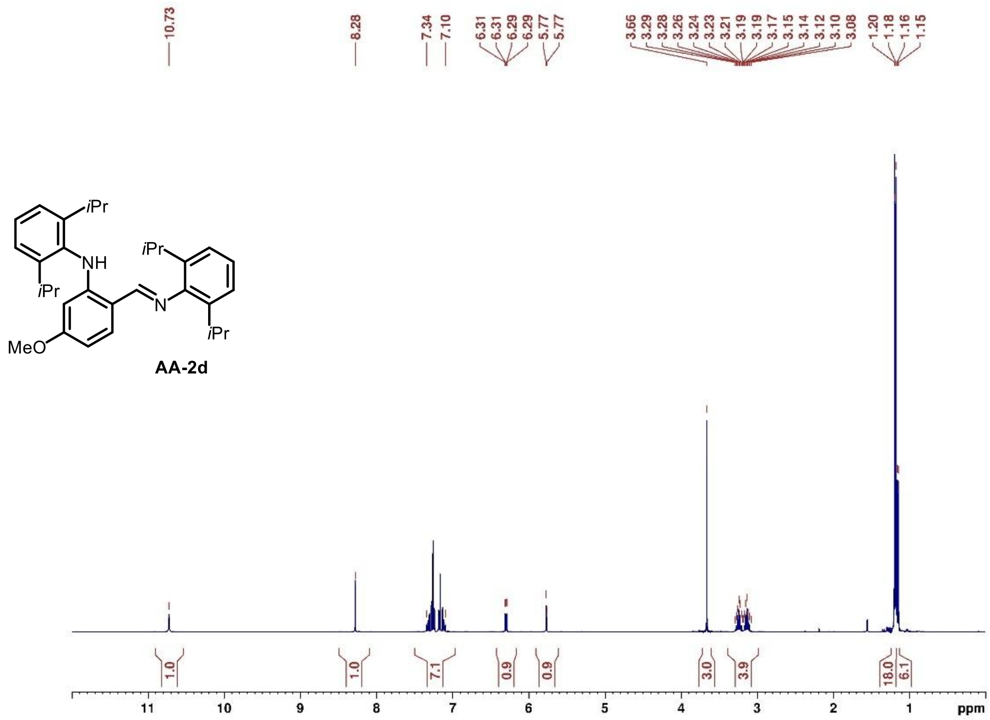

^{13}C NMR spectrum of (*E*)-*N*-(2,6-diisopropylphenyl)-2-(((2,6-diisopropylphenyl)imino)methyl)-5-methoxyaniline (**AA-2d**) (400 MHz, $CDCl_3$, RT)

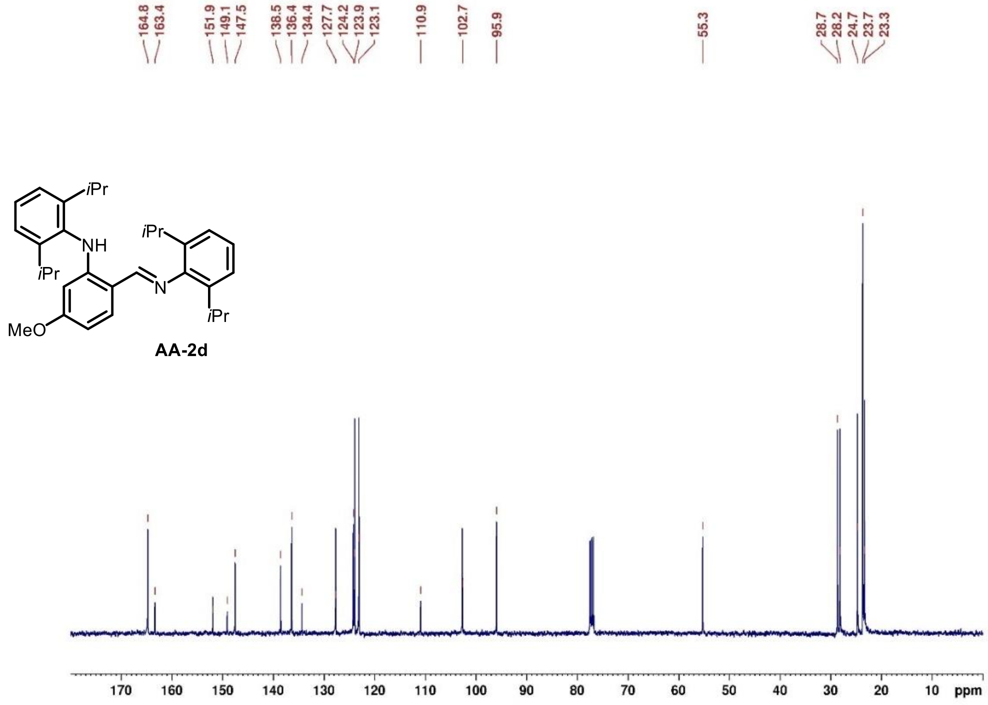

^{1}H NMR spectrum of (E)-N-(2,6-diisopropylphenyl)-1-(2-fluoro-5-methoxyphenyl)methanimine (**I-2e**) (400 MHz, $CDCl_3$, RT)

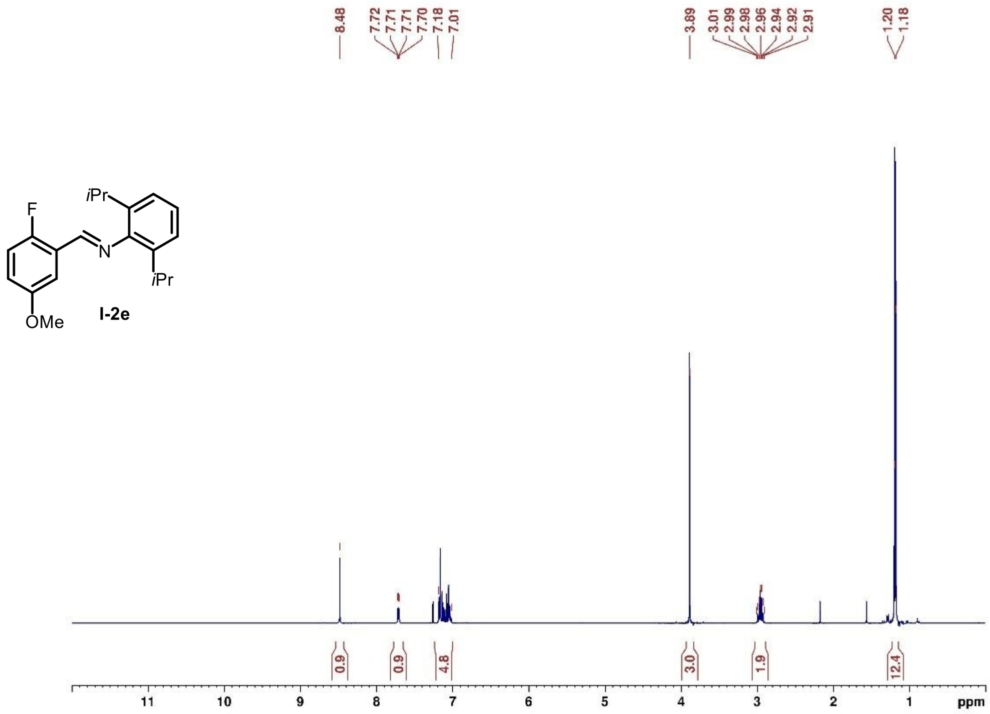

^{13}C NMR spectrum of ((E)-N-(2,6-diisopropylphenyl)-1-(2-fluoro-5-methoxyphenyl)methanimine (**I-2e**) (100 MHz, $CDCl_3$, RT)

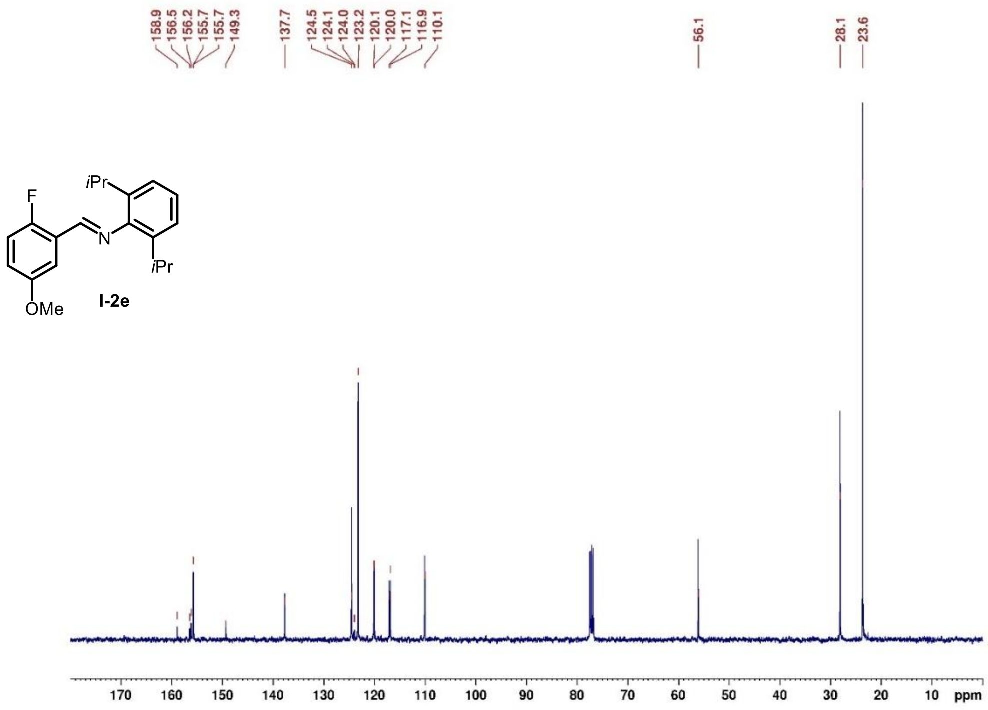

^1H NMR spectrum of (*E*)-*N*-(2,6-diisopropylphenyl)-2-(((2,6-diisopropylphenyl)imino)methyl)-4-methoxyaniline **(AA-2e)** (400 MHz, $CDCl_3$, RT)

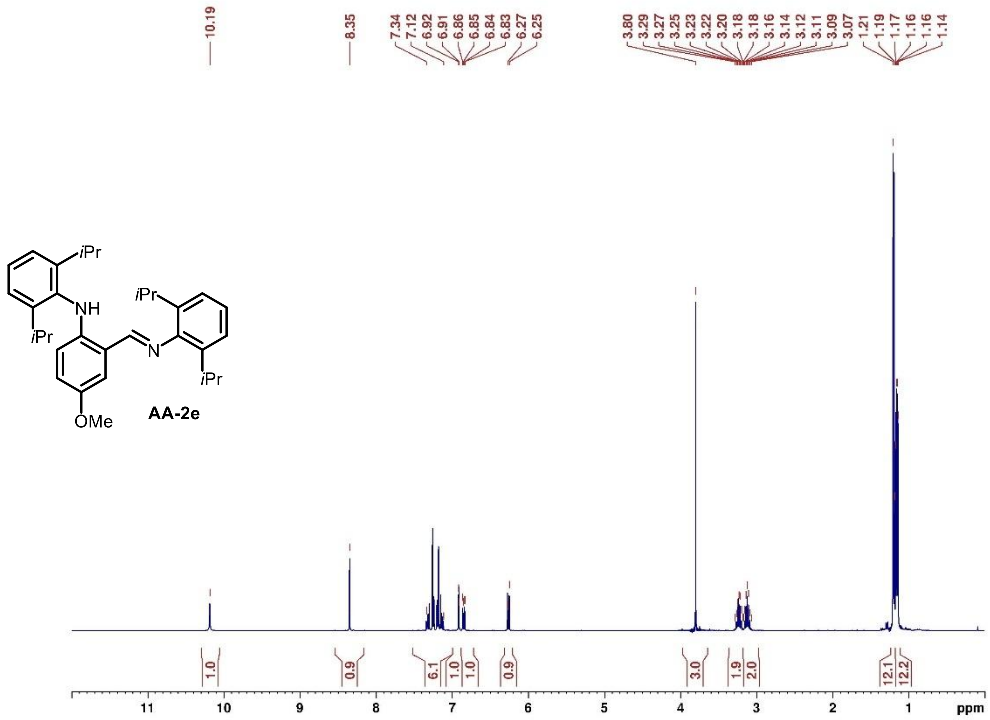

^{13}C NMR spectrum of (*E*)-*N*-(2,6-diisopropylphenyl)-2-(((2,6-diisopropylphenyl)imino)methyl)-4-methoxyaniline **(AA-2e)** (100 MHz, $CDCl_3$, RT)

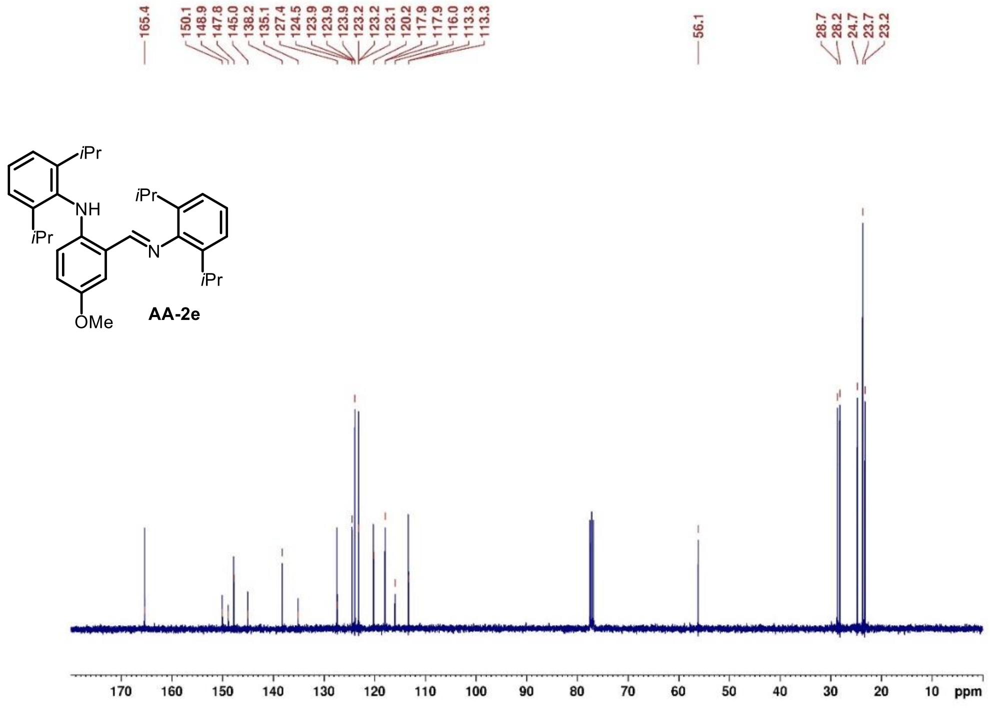

^{1}H NMR spectrum of (*E*)-1-(2,5-difluorophenyl)-*N*-(2,6-diisopropylphenyl)methanimine (**I-2f**) (400 MHz, $CDCl_3$, RT)

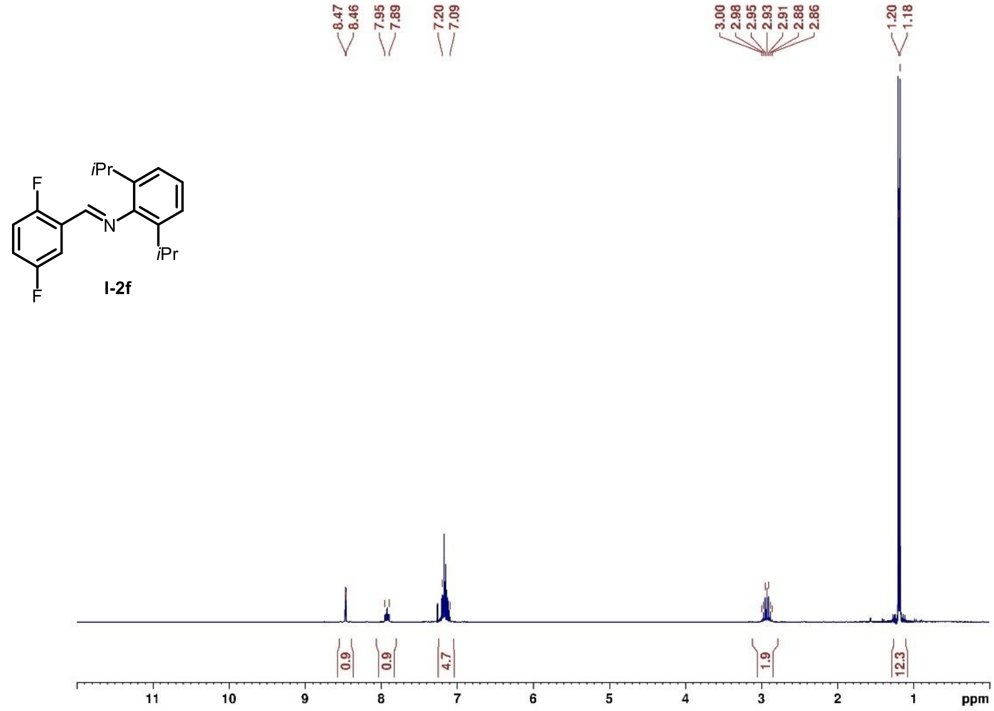

^{13}C NMR spectrum of (*E*)-1-(2,5-difluorophenyl)-*N*-(2,6-diisopropylphenyl)methanimine (**I-2f**) (100 MHz, $CDCl_3$, RT)

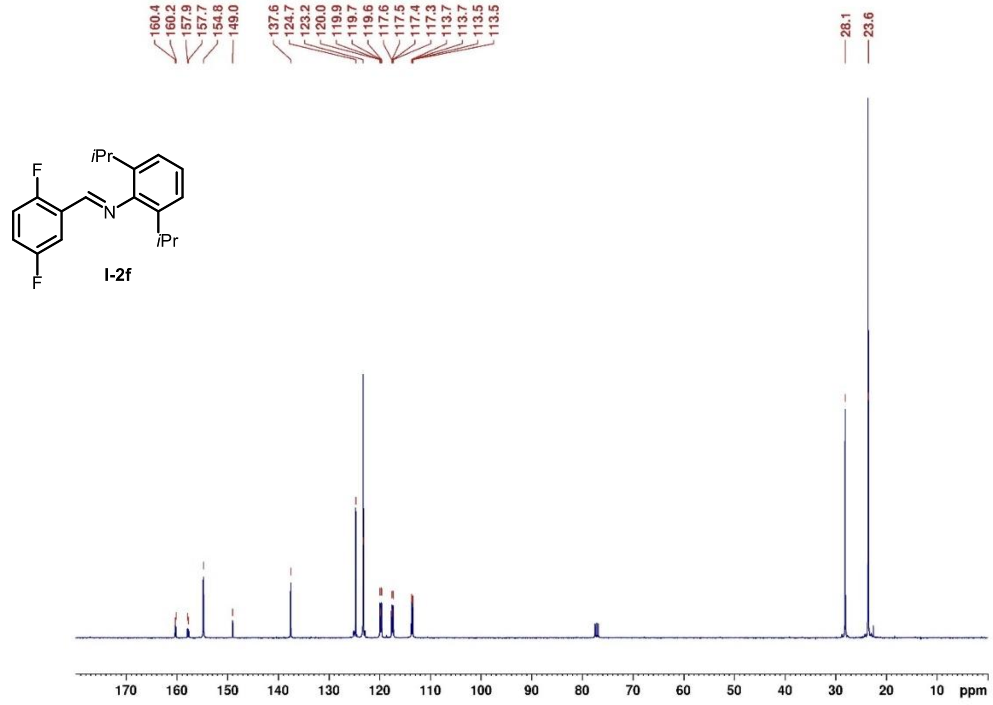

^{1}H NMR spectrum of (*E*)-*N*-(2,6-diisopropylphenyl)-2-(((2,6-diisopropylphenyl)imino)methyl)-4-fluoroaniline (**AA-2f**) (400 MHz, $CDCl_3$, RT)

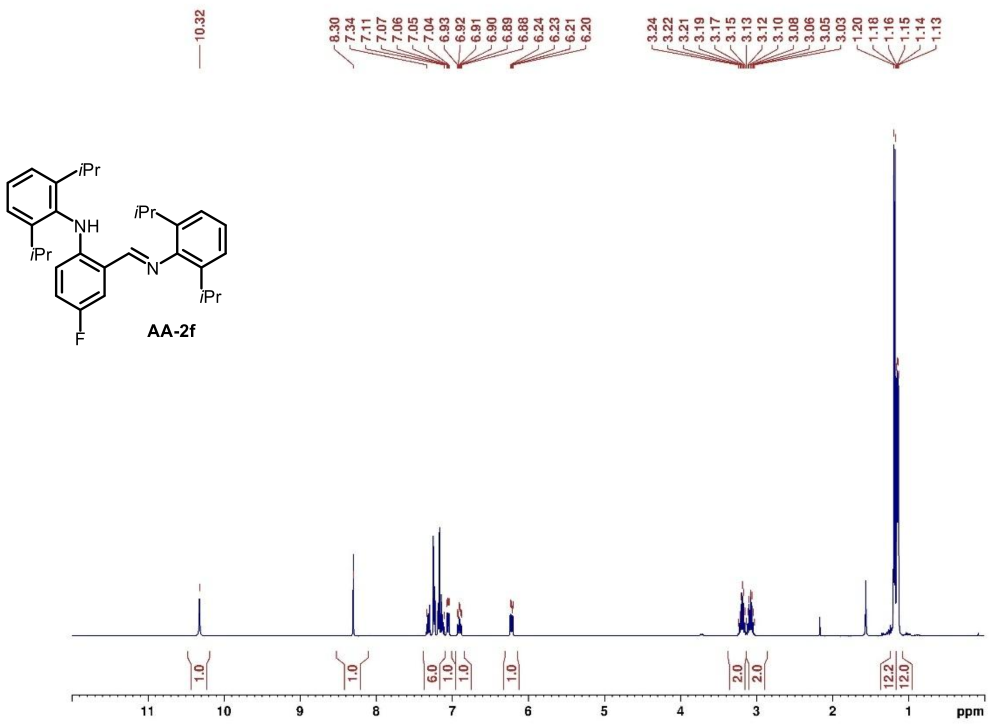

^{13}C NMR spectrum of (*E*)-*N*-(2,6-diisopropylphenyl)-2-(((2,6-diisopropylphenyl)imino)methyl)-4-fluoroaniline (**AA-2f**) (100 MHz, $CDCl_3$, RT)

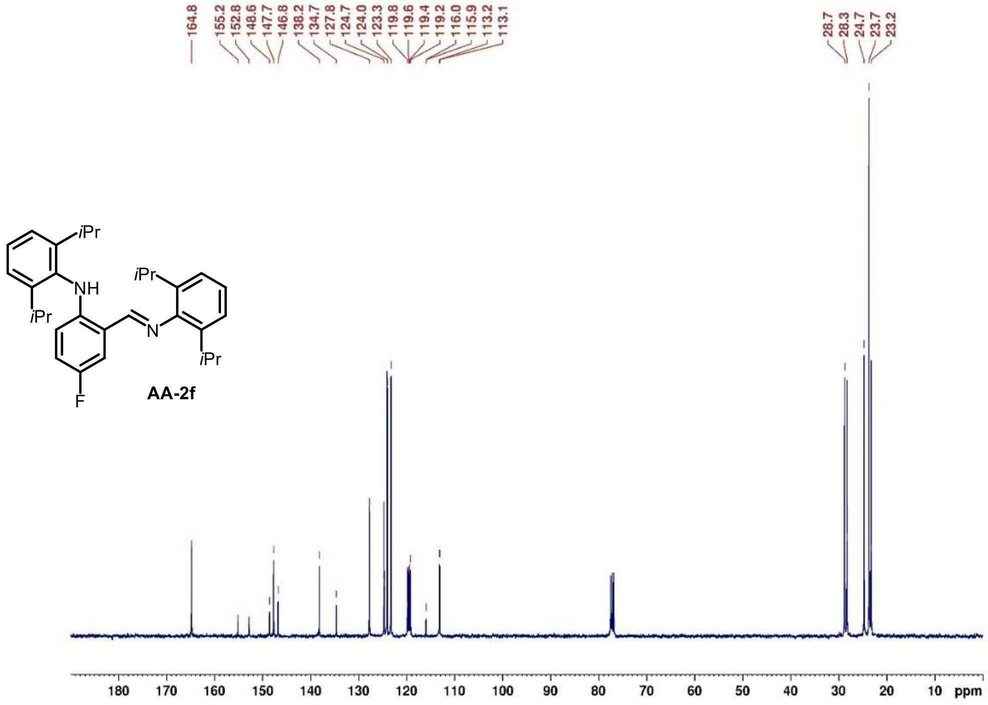

^{1}H NMR spectrum of (*E*)-*N*-(2-fluorobenzylidene)-2,4,6-trimethylaniline (**I-2g**) (250 MHz, $CDCl_3$, RT)

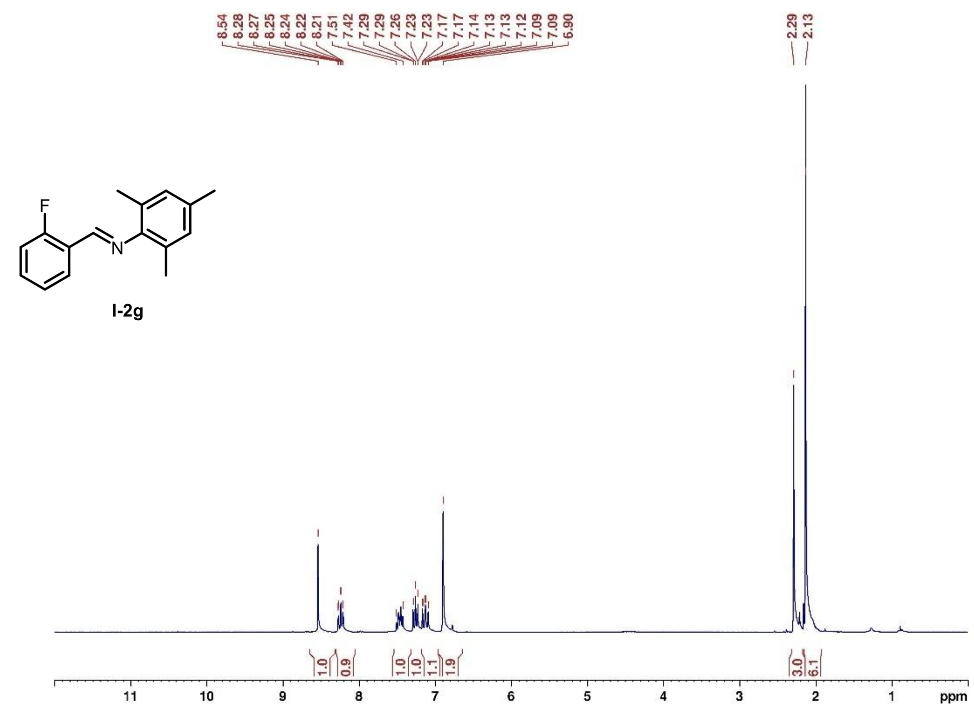

^{1}H NMR spectrum of (*E*)-*N*-(2-(mesitylamino)benzylidene)-2,4,6-trimethylaniline (**AA-2g**) (250 MHz, $CDCl_3$, RT)

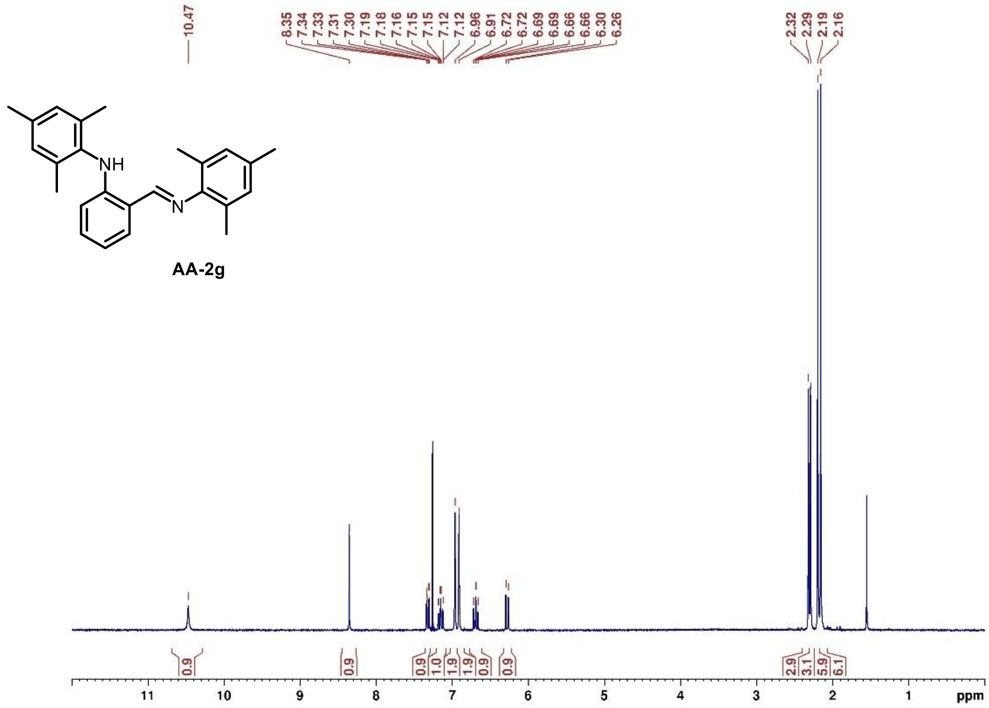

^{13}C NMR spectrum of (*E*)-*N*-(2-(mesitylamino)benzylidene)-2,4,6-trimethylaniline (**AA-2g**) (100 MHz, $CDCl_3$, RT)

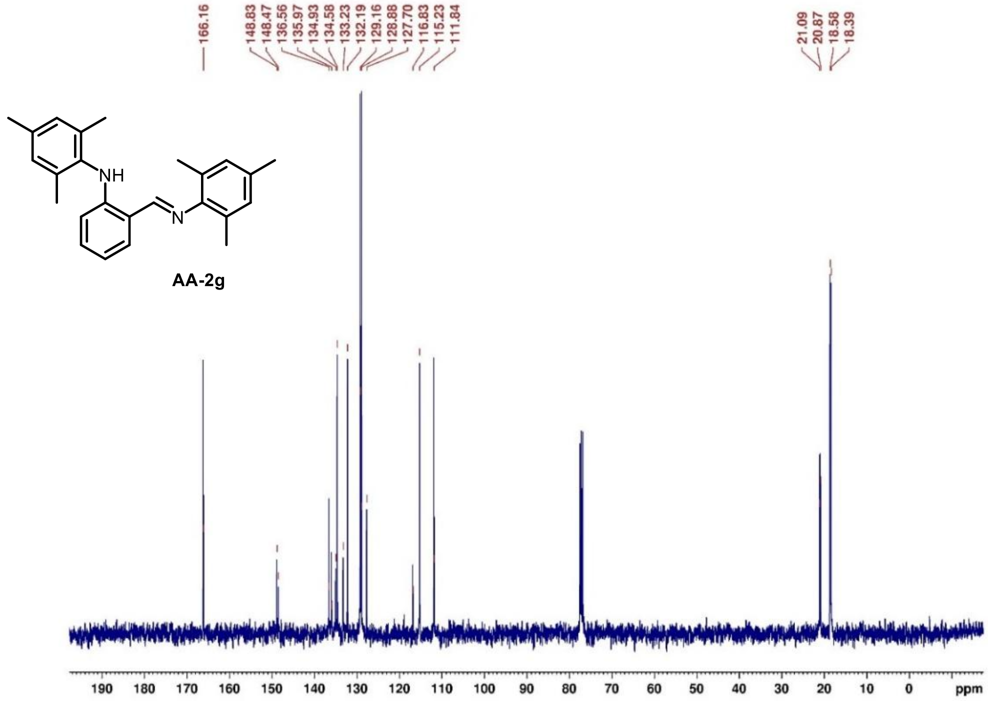

^{1}H NMR spectrum of complex **1f** (400 MHz, C_6D_6-50 µL THF-d_8, RT)

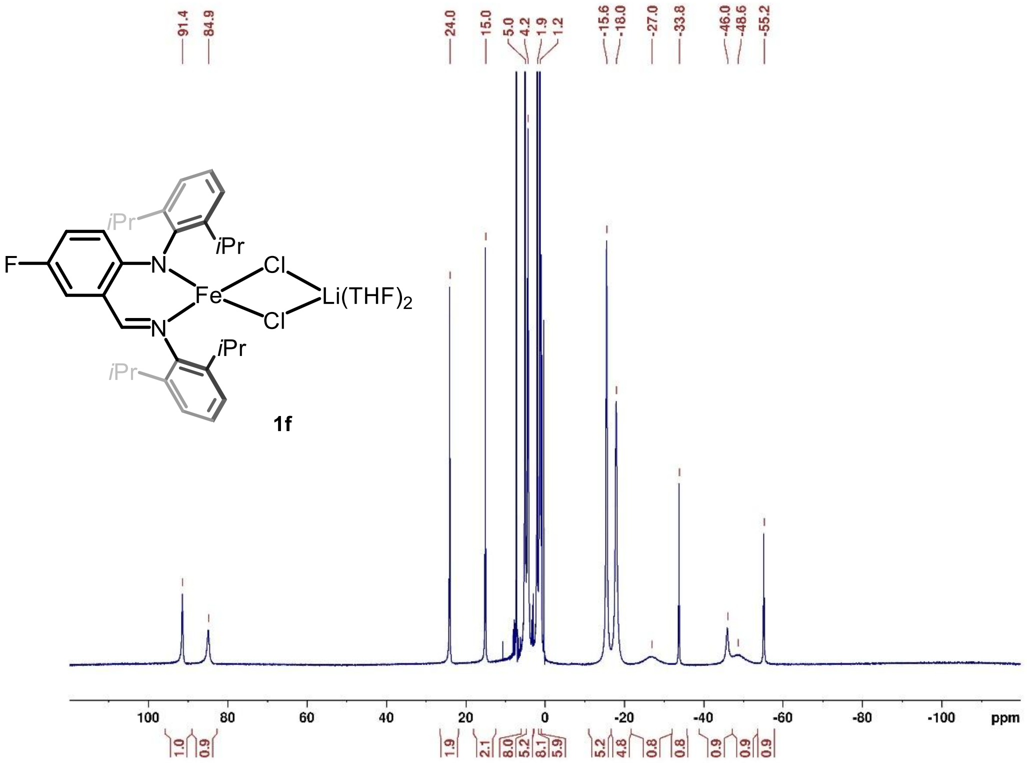

^{7}Li NMR spectrum of complex **1f** (117 MHz, C_6D_6-50 µL THF-d_8, RT)

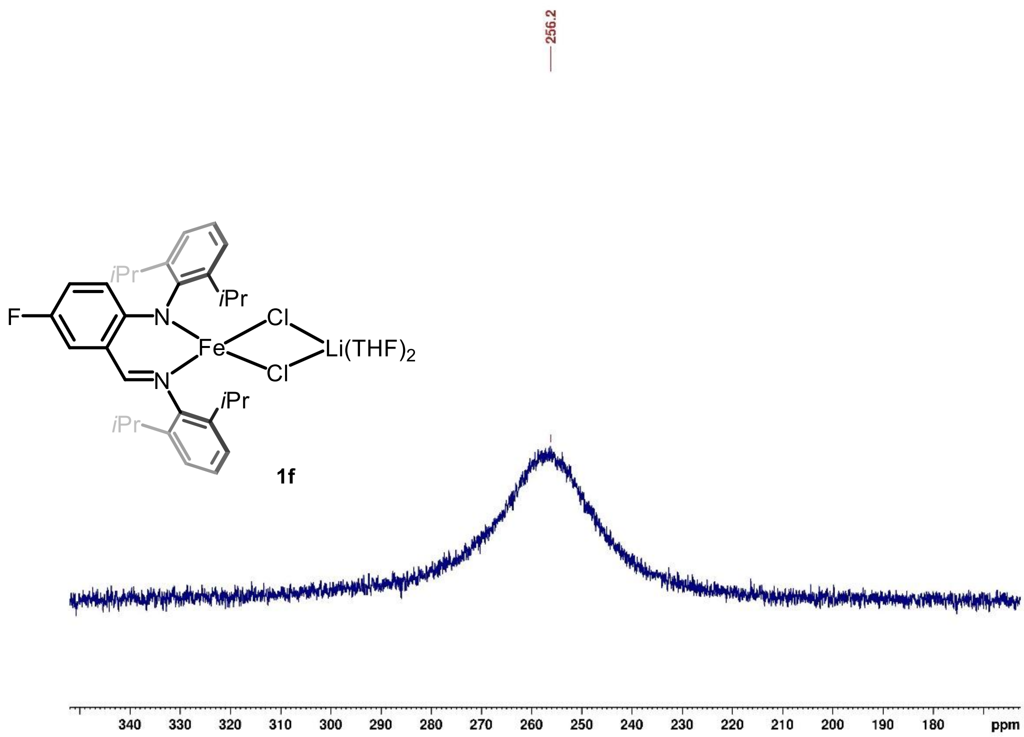

^{1}H NMR spectrum of complex **2a** (400 MHz, C_6D_6, RT)

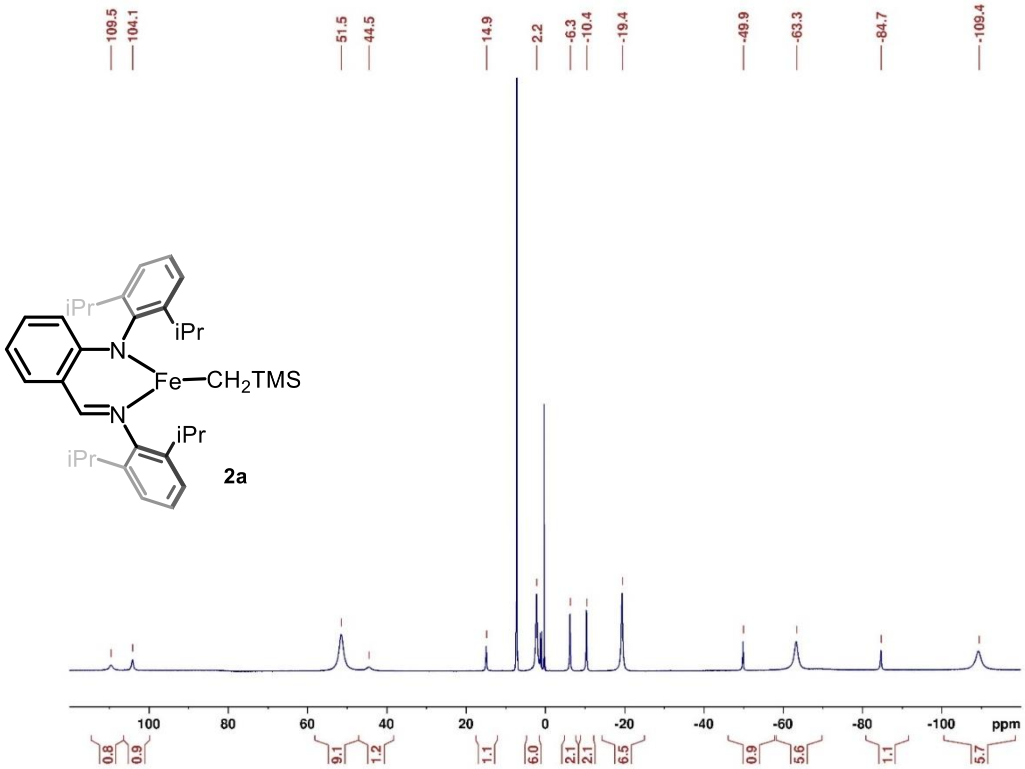

^{1}H NMR spectrum of complex **[2b]₂** (400 MHz, C_6D_6, 60 °C)

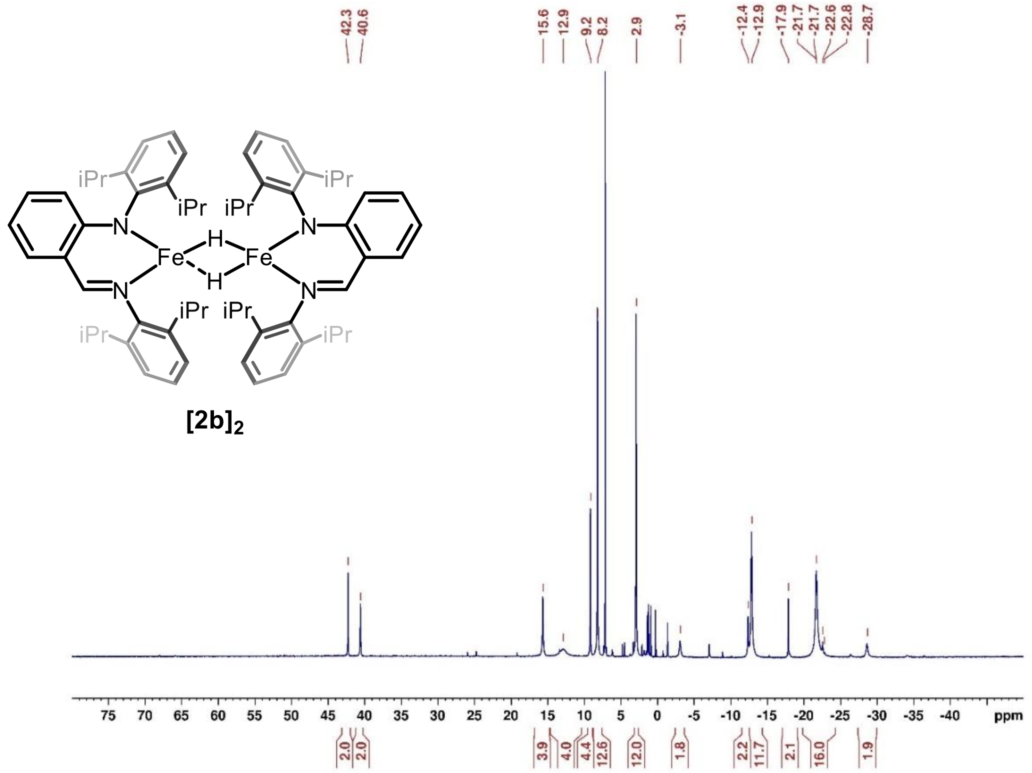

^{1}H NMR spectrum of complex **2c** (400 MHz, C_6D_6, RT)

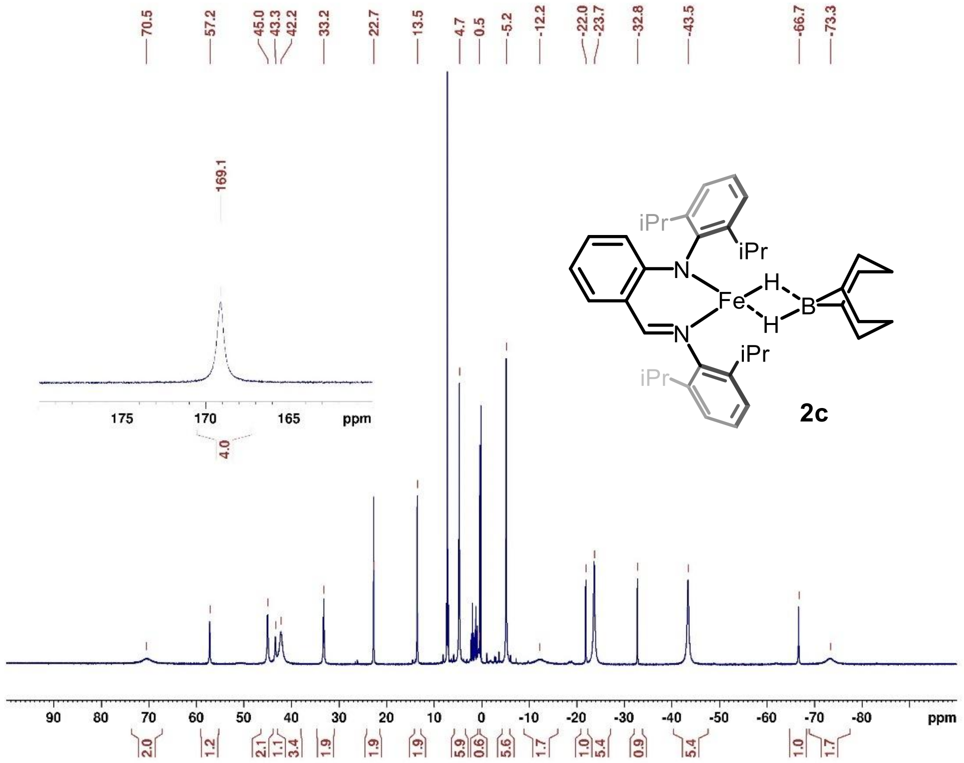

^1H NMR spectrum of complex **2d** (400 MHz, C_6D_6, RT)

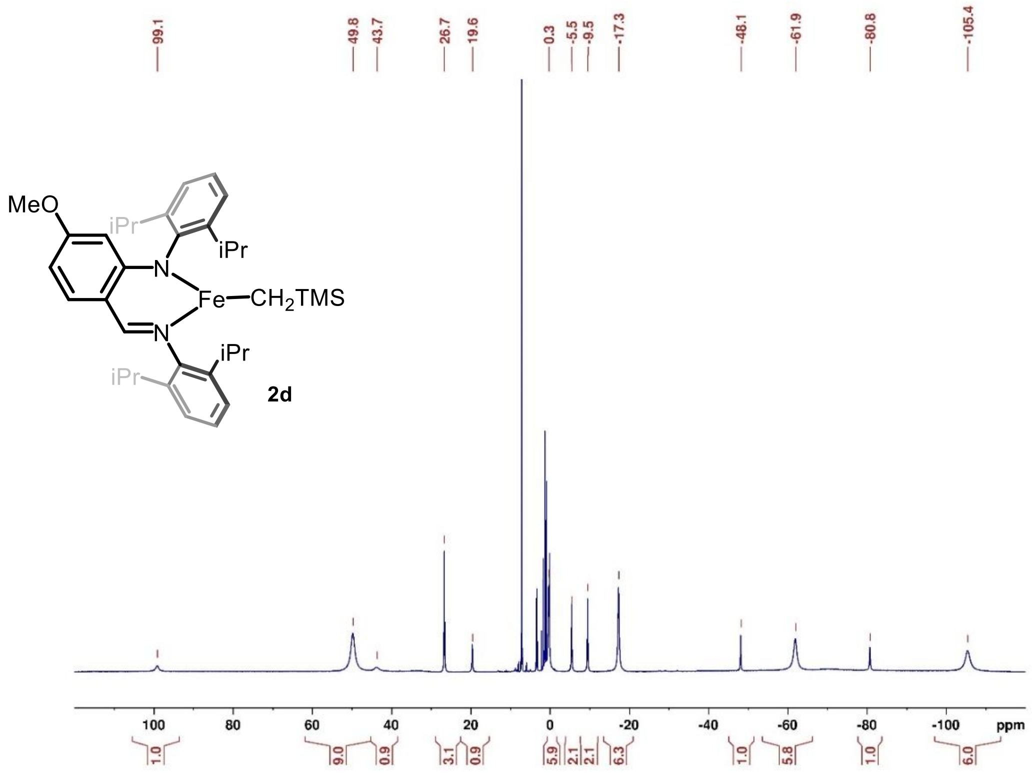

^1H NMR spectrum of complex **2e** (400 MHz, C_6D_6, RT)

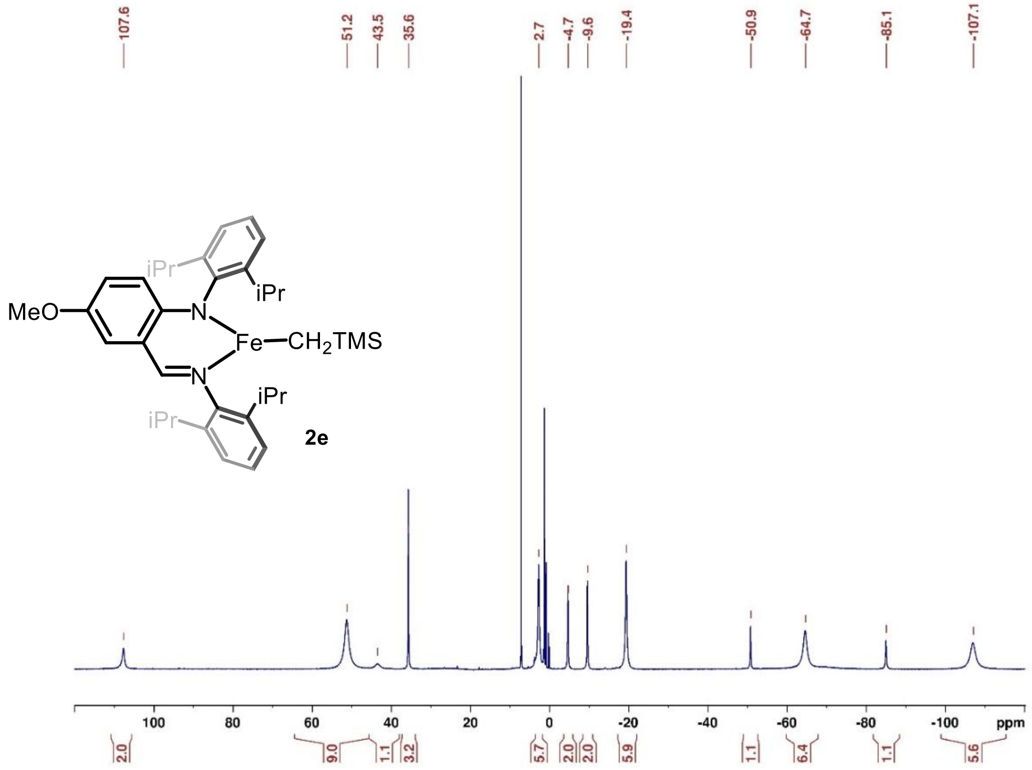

^{1}H NMR spectrum of complex **2f** (400 MHz, C_6D_6, RT)

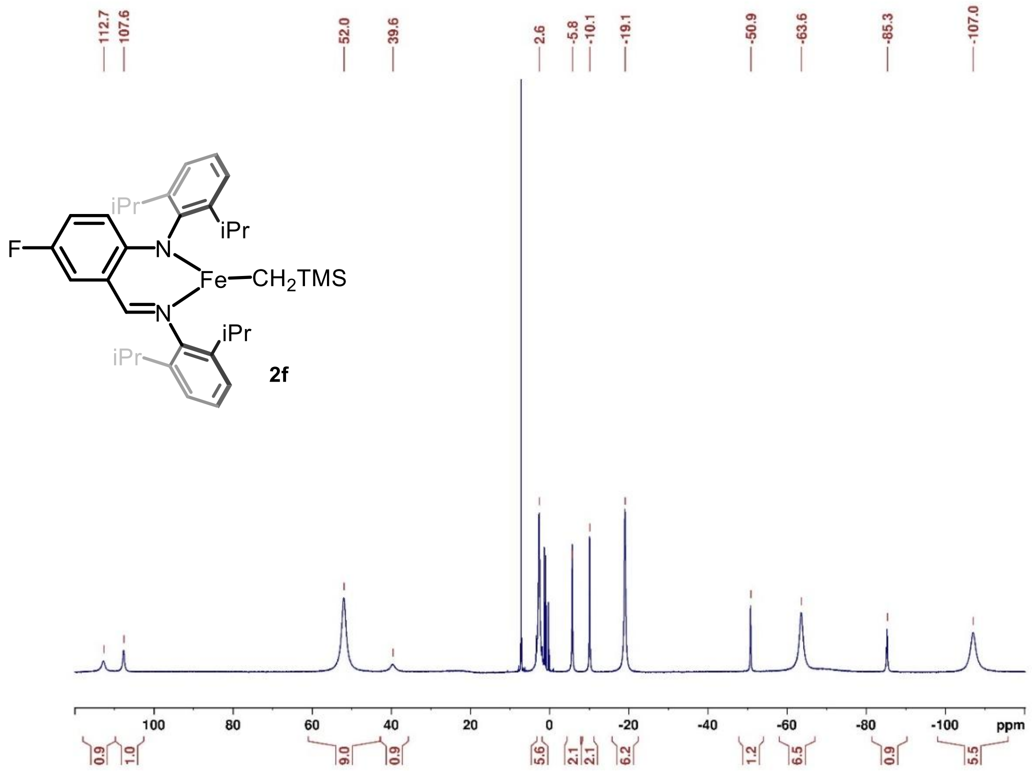

^{1}H NMR spectrum of complex **2g** (400 MHz, C_6D_6, RT)

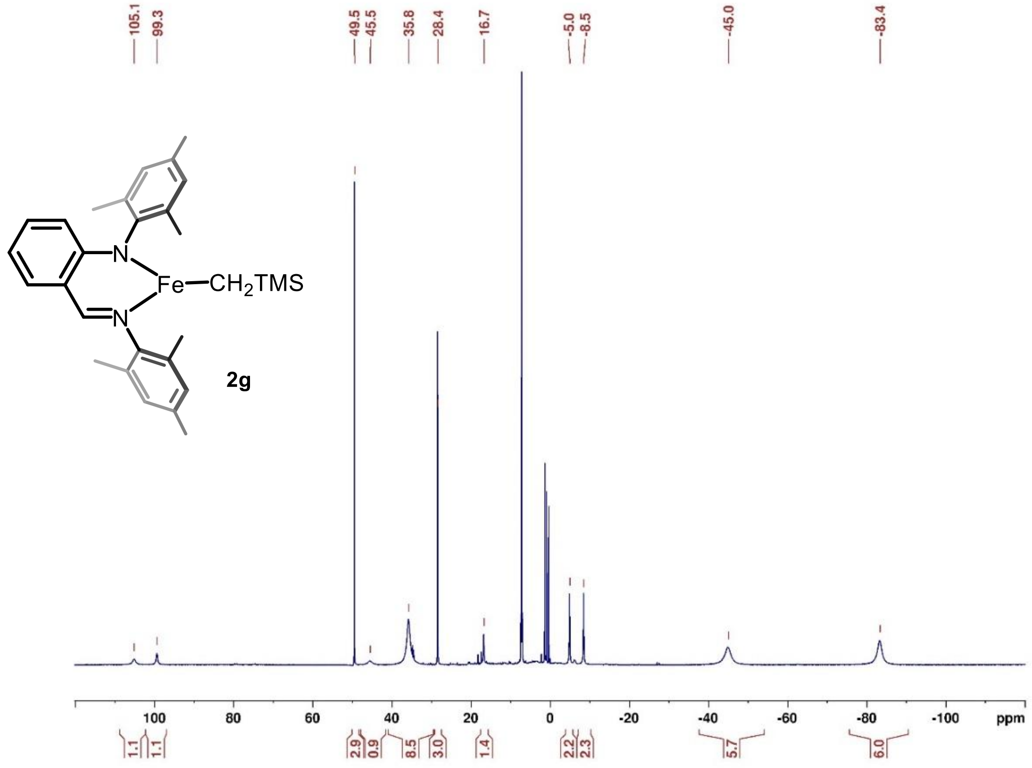

^{1}H NMR spectrum of complex **2h** (300 MHz, C_6D_6, RT)

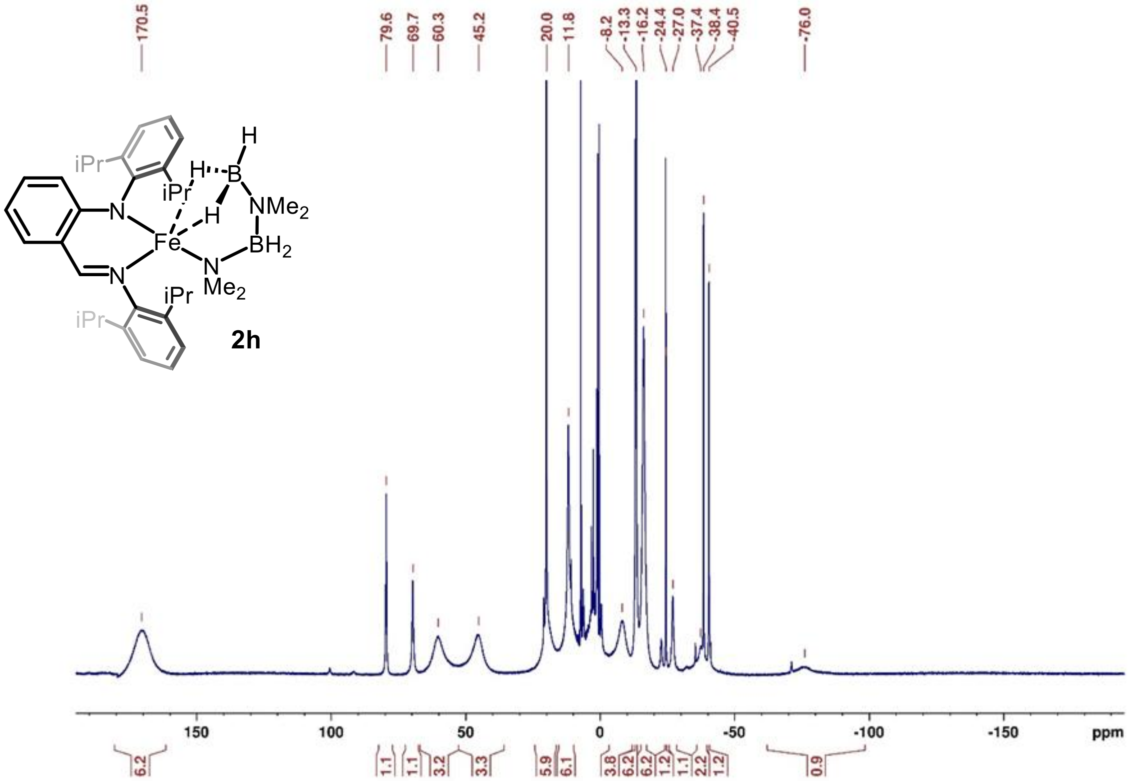

^1H NMR spectrum of **$Cy_2NH{\cdot}BD_3$** (400 MHz, $CDCl_3$, RT)

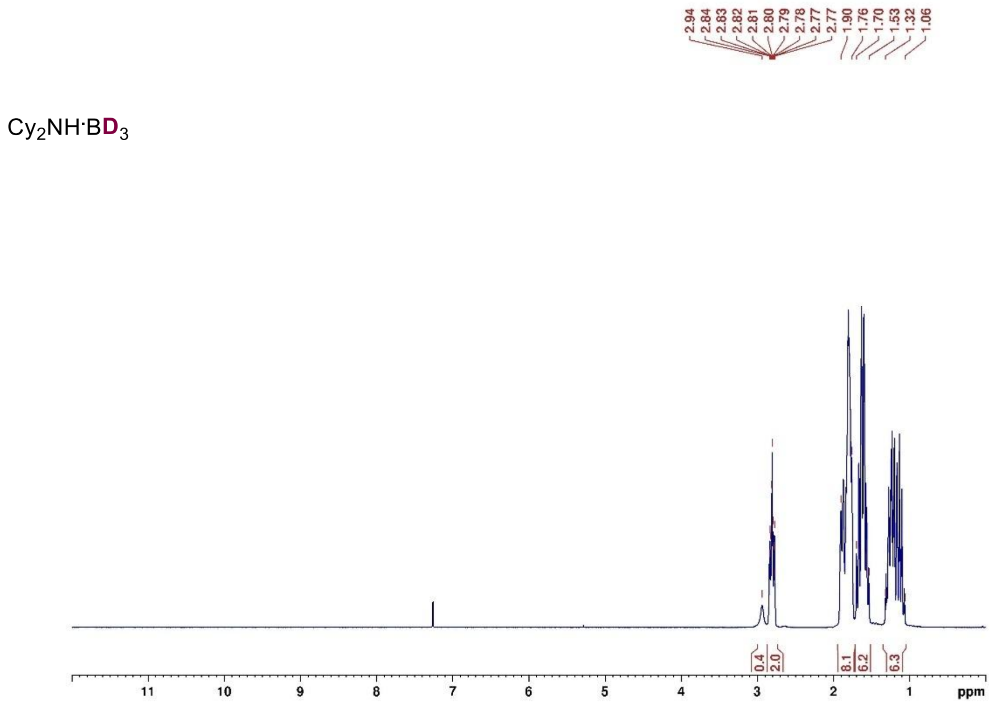

^{11}B NMR spectrum of **$Cy_2NH{\cdot}BD_3$** (96 MHz, $CDCl_3$, RT)

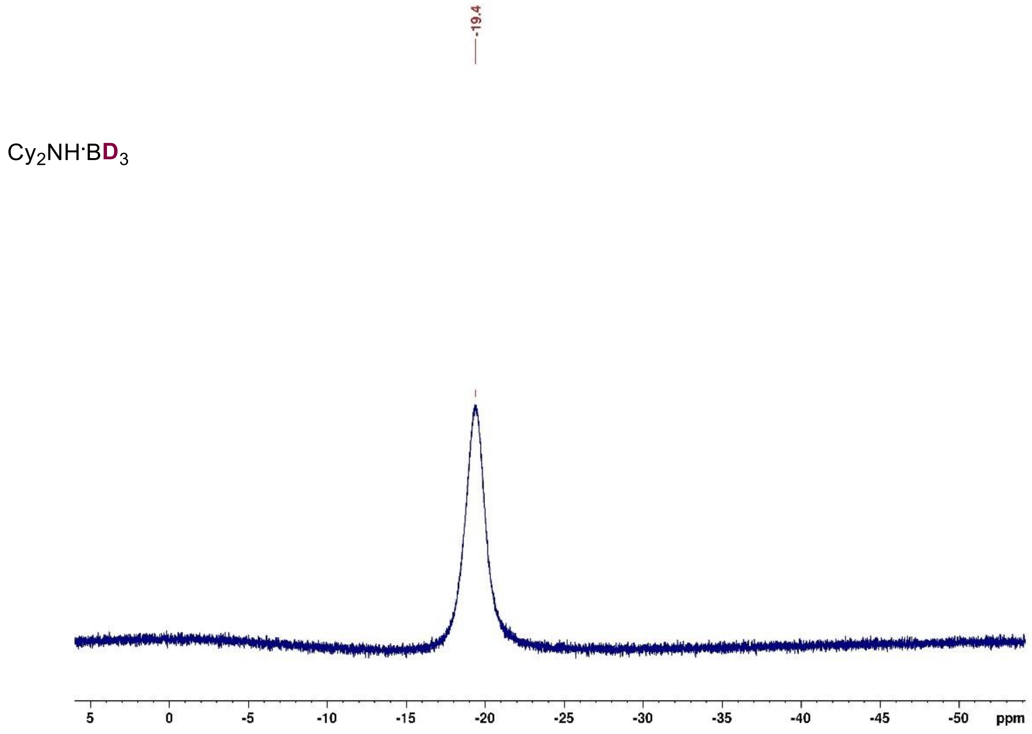

^{13}C NMR spectrum of **$Cy_2NH{\cdot}BD_3$** (100 MHz, $CDCl_3$, RT)

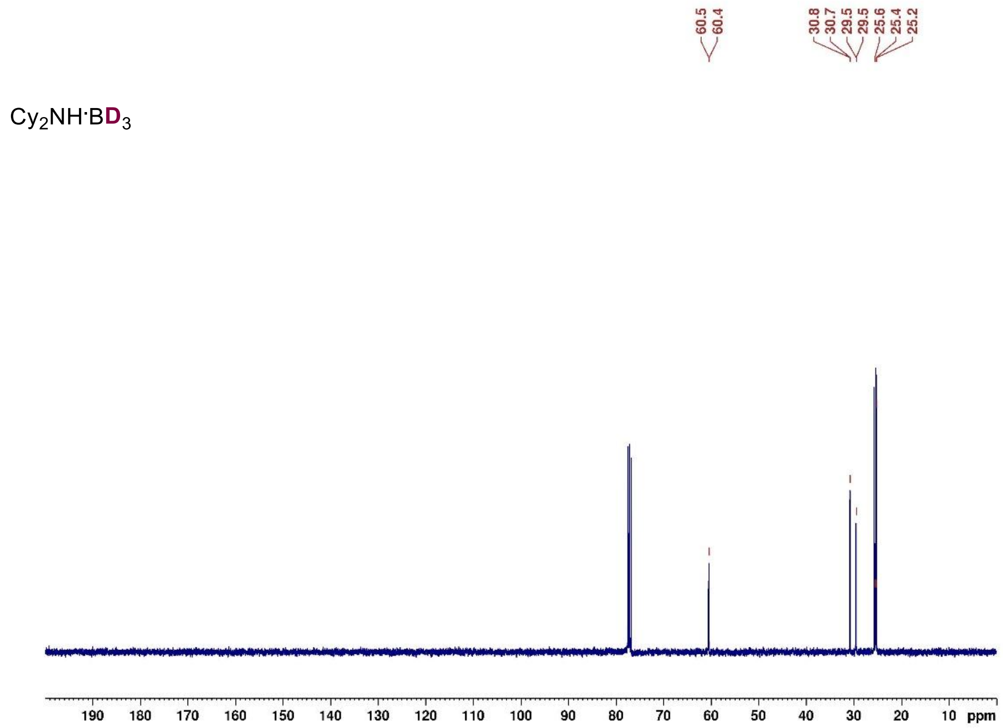

^{1}H NMR spectrum of ***p*-tolylmethan-*d*$_2$-ol** (400 MHz, $CDCl_3$, RT)

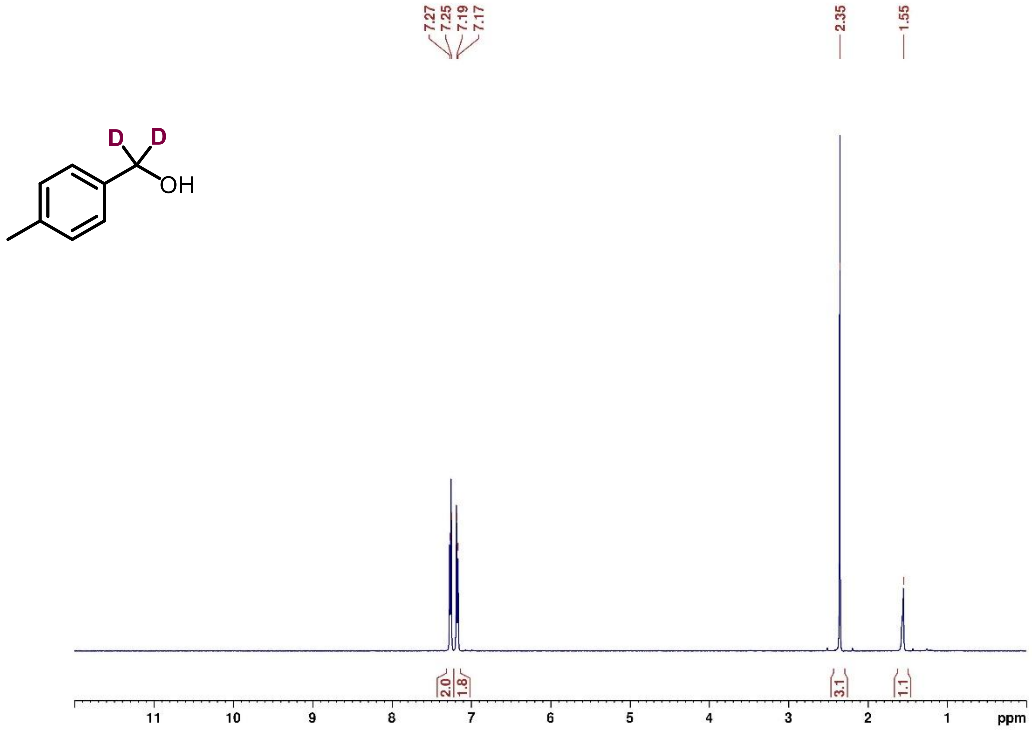

^{13}C NMR spectrum of ***p*-tolylmethan-*d*$_2$-ol** (100 MHz, $CDCl_3$, RT)

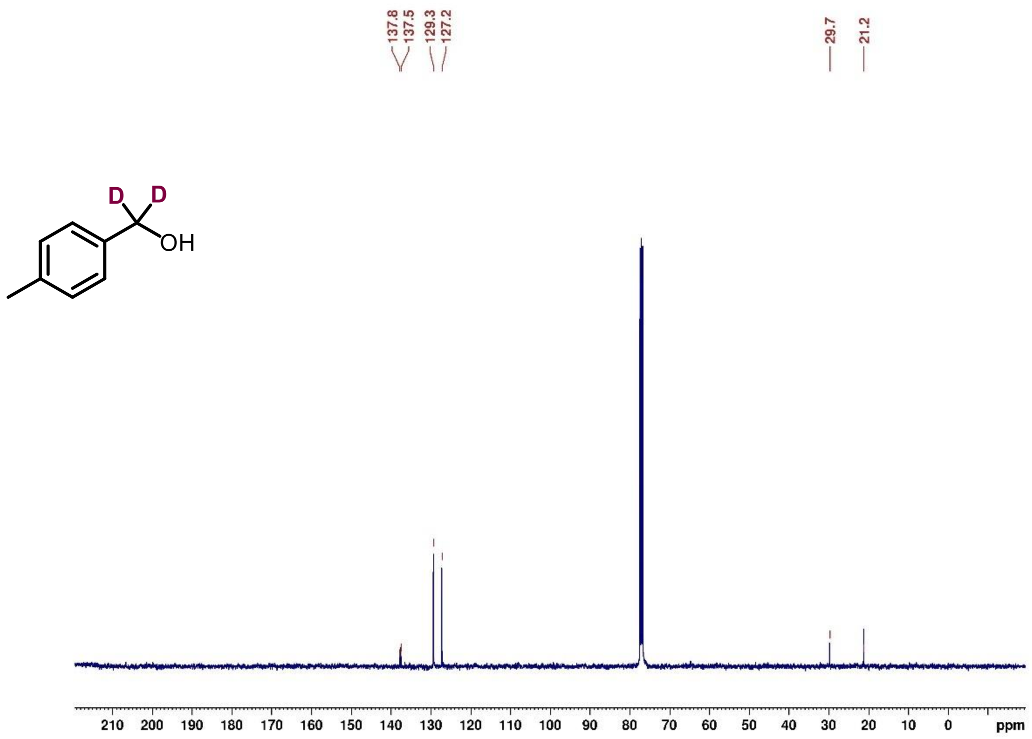

^{1}H NMR spectrum of **1-(bromomethyl-*d*$_2$)-4-methylbenzene** (400 MHz, $CDCl_3$, RT)

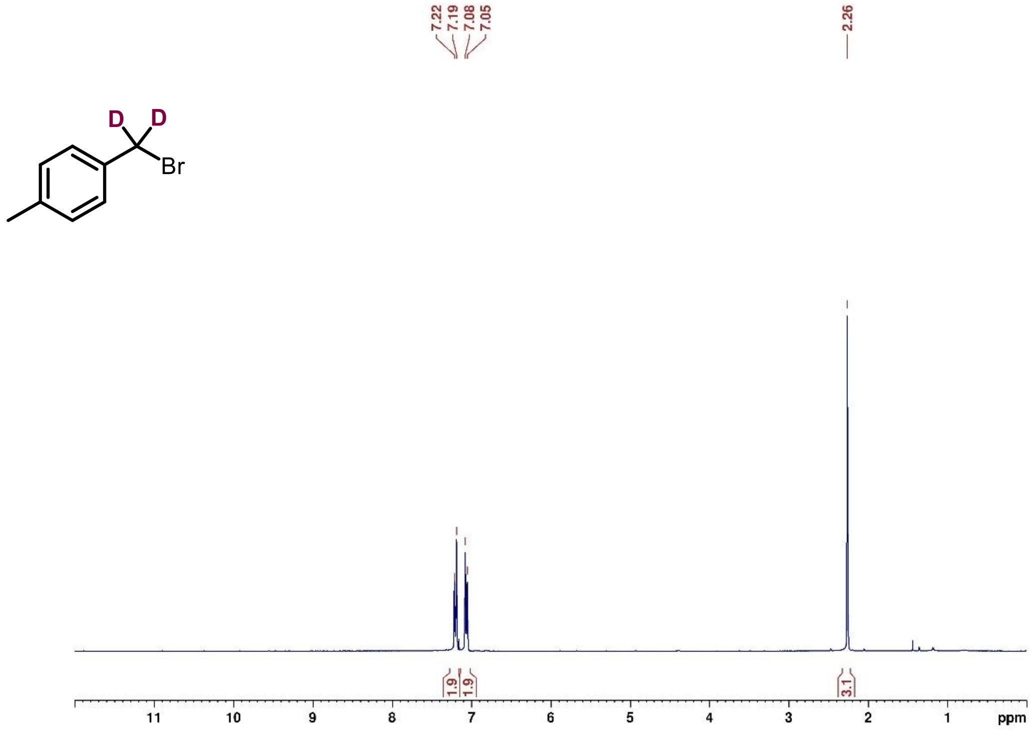

^{13}C NMR spectrum of **1-(bromomethyl-*d*$_2$)-4-methylbenzene** (100 MHz, $CDCl_3$, RT)

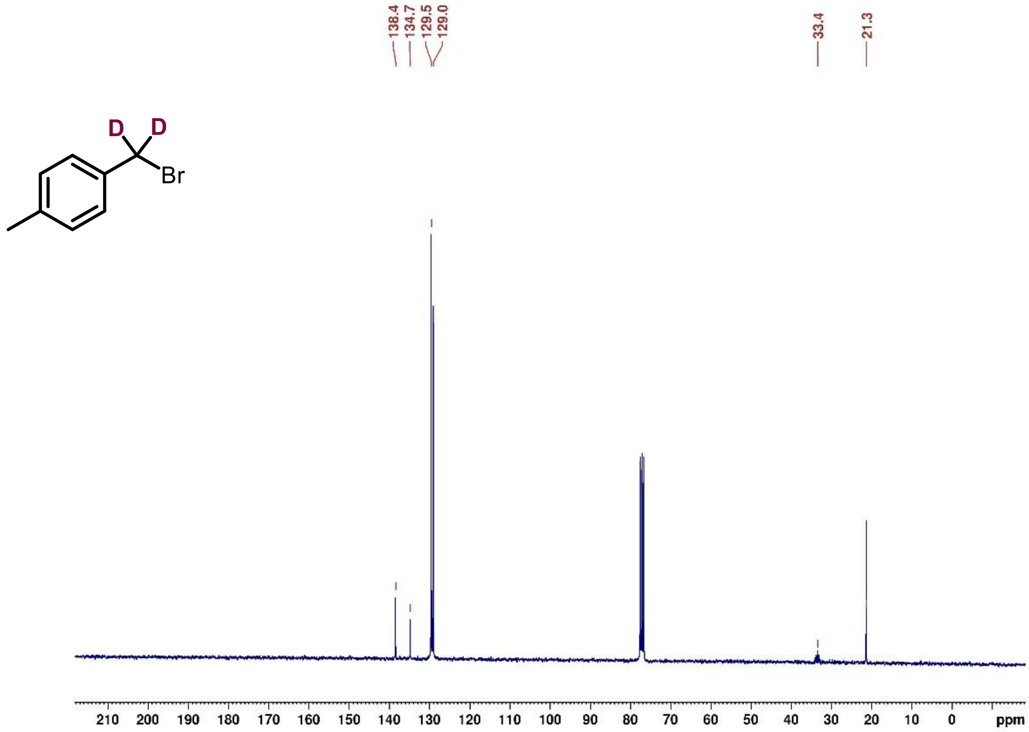

^{1}H NMR spectrum of **1-(but-3-en-1-yl-1,1-*d*$_2$)-4-methylbenzene** (400 MHz, $CDCl_3$, RT)

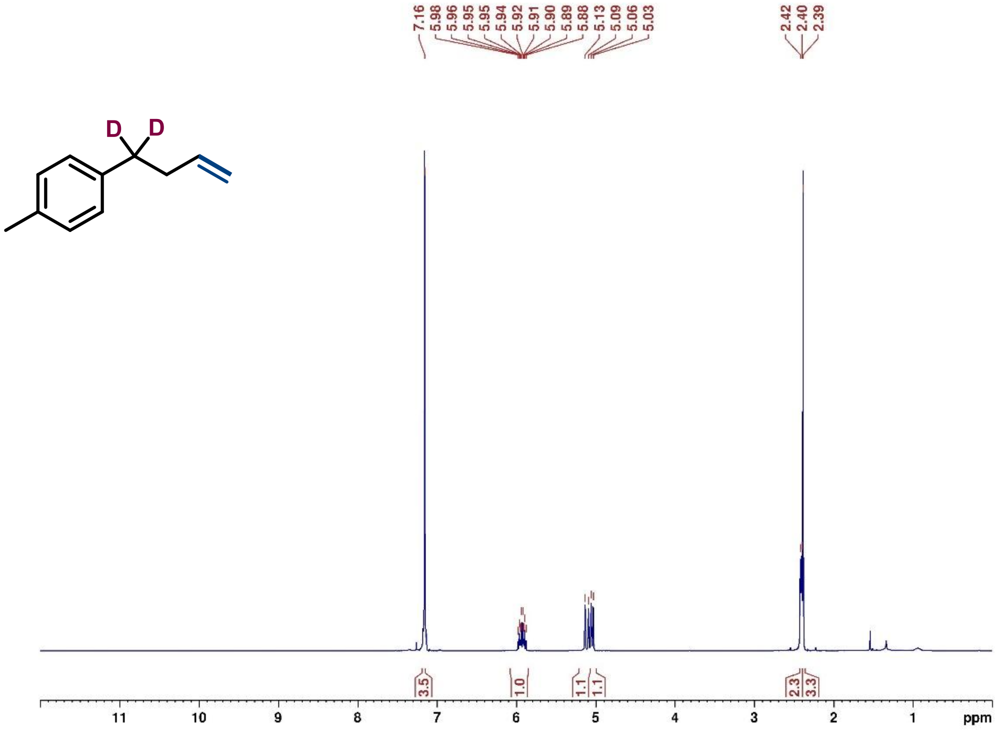

^{13}C NMR spectrum of **1-(but-3-en-1-yl-1,1-*d*$_2$)-4-methylbenzene** (100 MHz, $CDCl_3$, RT)

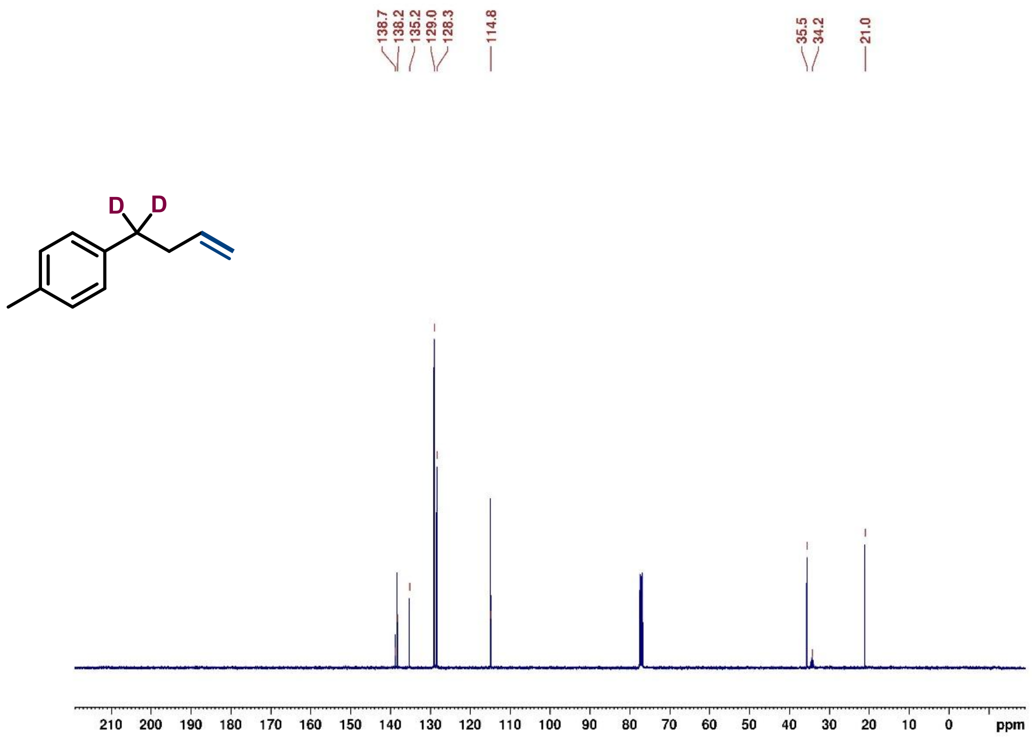

^{1}H NMR spectrum of (*E*)-prop-1-en-1-ylbenzene (*E*)-**4a** (400 MHz, $CDCl_3$, RT)

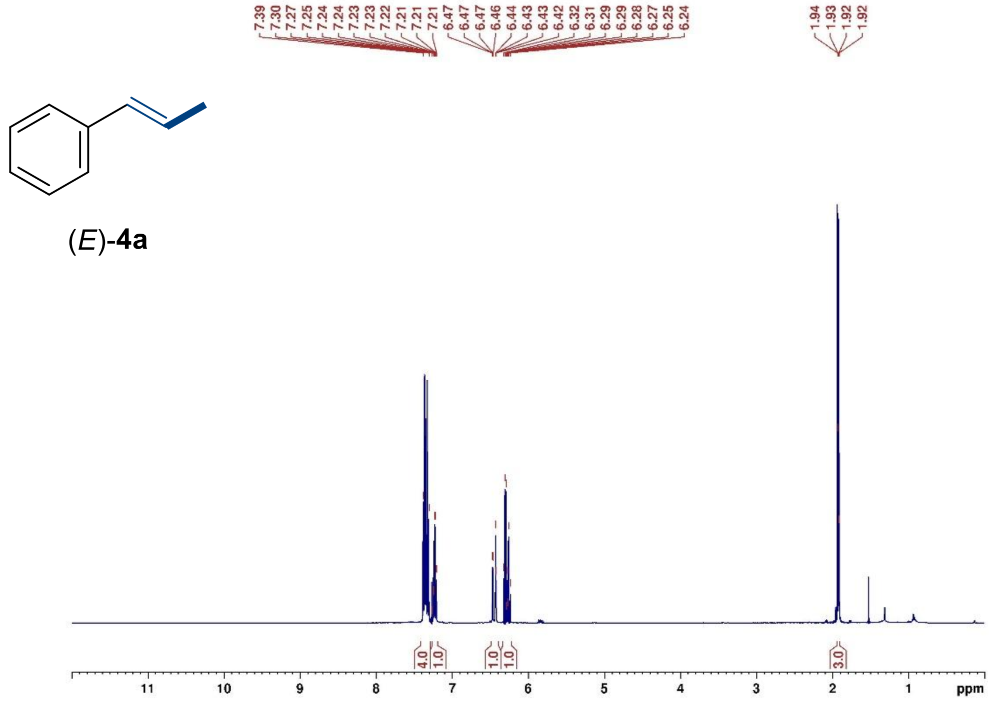

(*E*)-**4a**

^{13}C NMR spectrum of (*E*)-prop-1-en-1-ylbenzene (*E*)-**4a** (100 MHz, $CDCl_3$, RT)

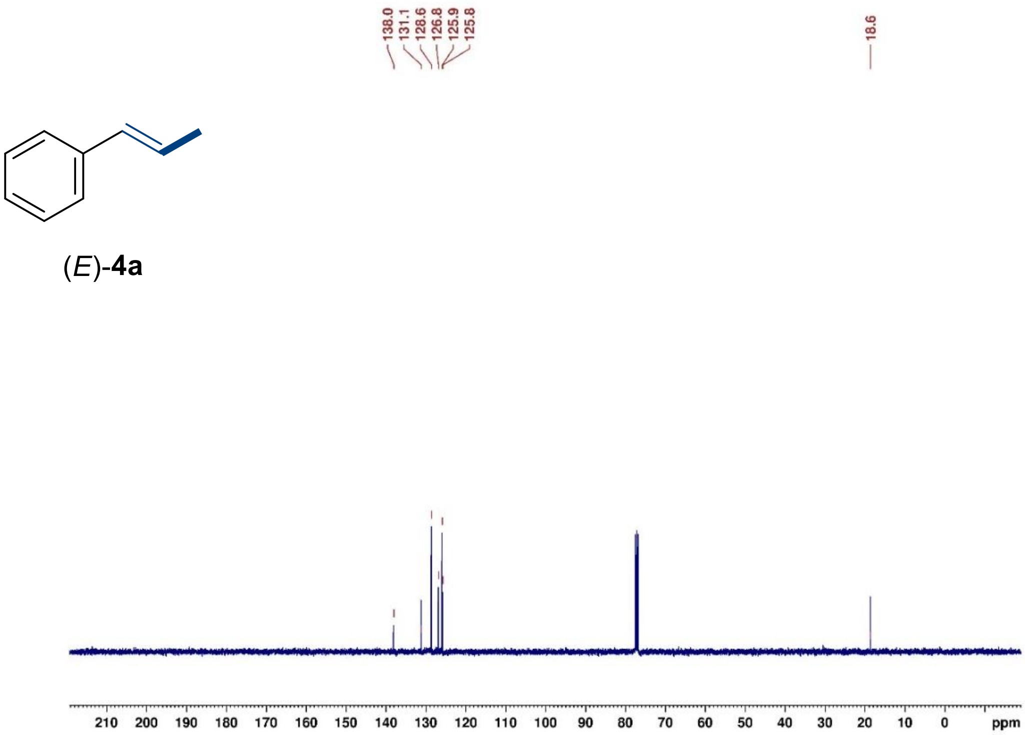

^{1}H NMR spectrum of (*E*)-1,2-diphenylethene (*E*)-**3b** (400 MHz, $CDCl_3$, RT)

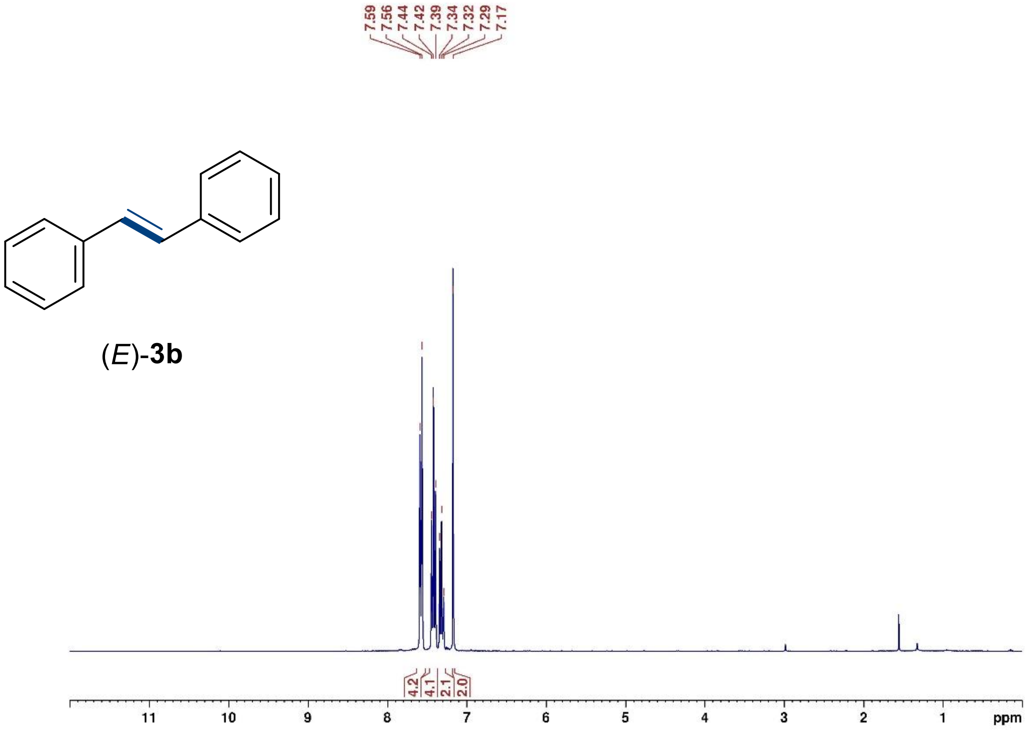

^{13}C NMR spectrum of (*E*)-1,2-diphenylethene (*E*)-**3b** (100 MHz, $CDCl_3$, RT)

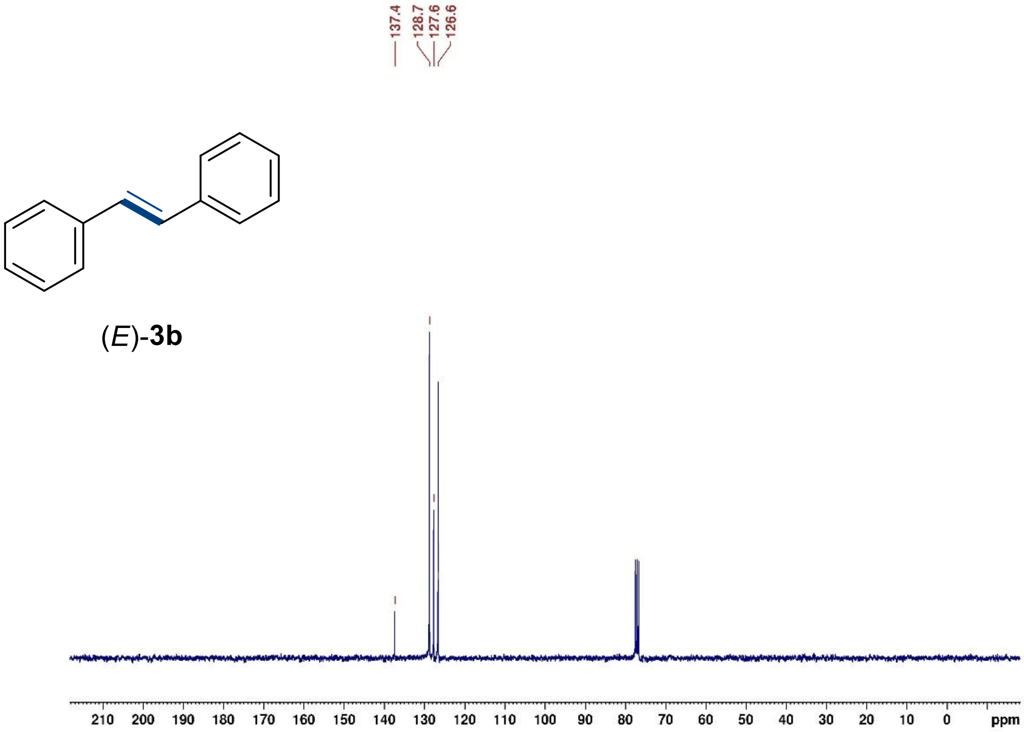

^{1}H NMR spectrum of (*E*)-*N*,*N*-dimethyl-4-(prop-1-en-1-yl)aniline (*E*)-**4e** (400 MHz, $CDCl_3$, RT)

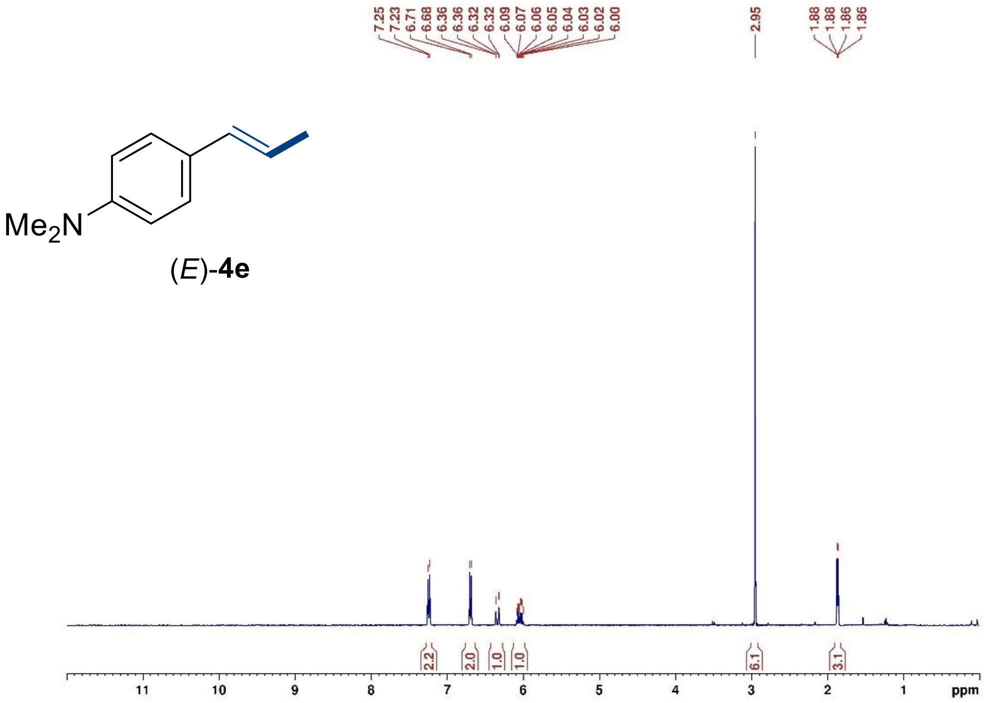

^{13}C NMR spectrum of (*E*)-*N*,*N*-dimethyl-4-(prop-1-en-1-yl)aniline (*E*)-**4e** (100 MHz, $CDCl_3$, RT)

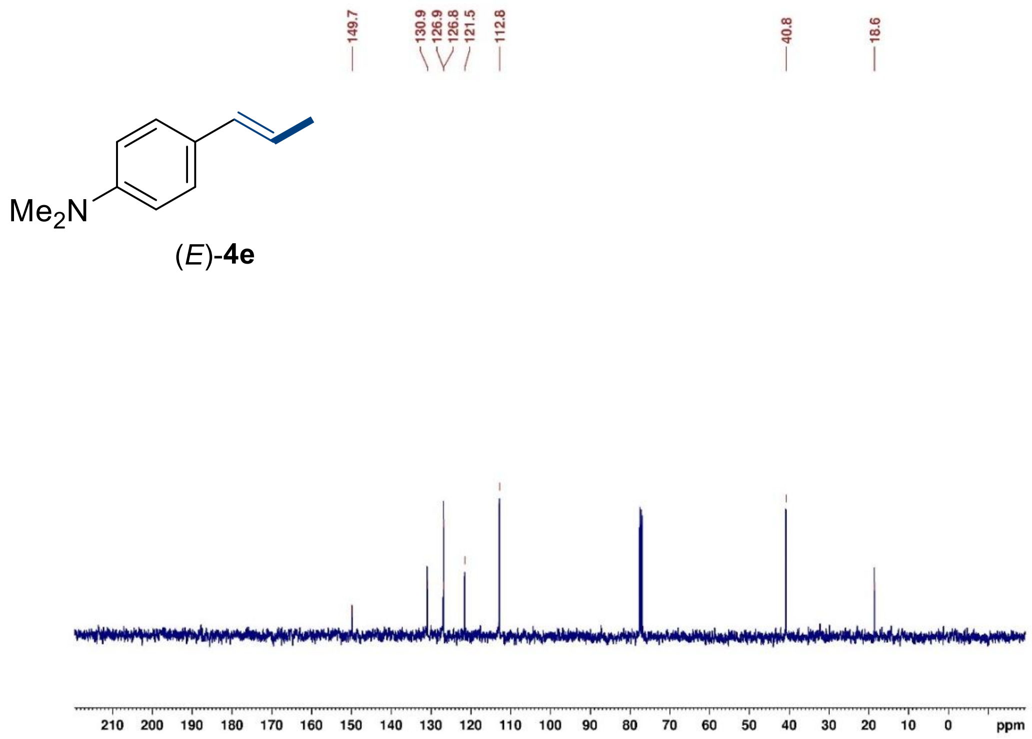

^{1}H NMR spectrum of (*E*)-1-(prop-1-en-1-yl)-4-(trifluoromethyl)benzene (*E*)-**4f** (400 MHz, $CDCl_3$, RT)

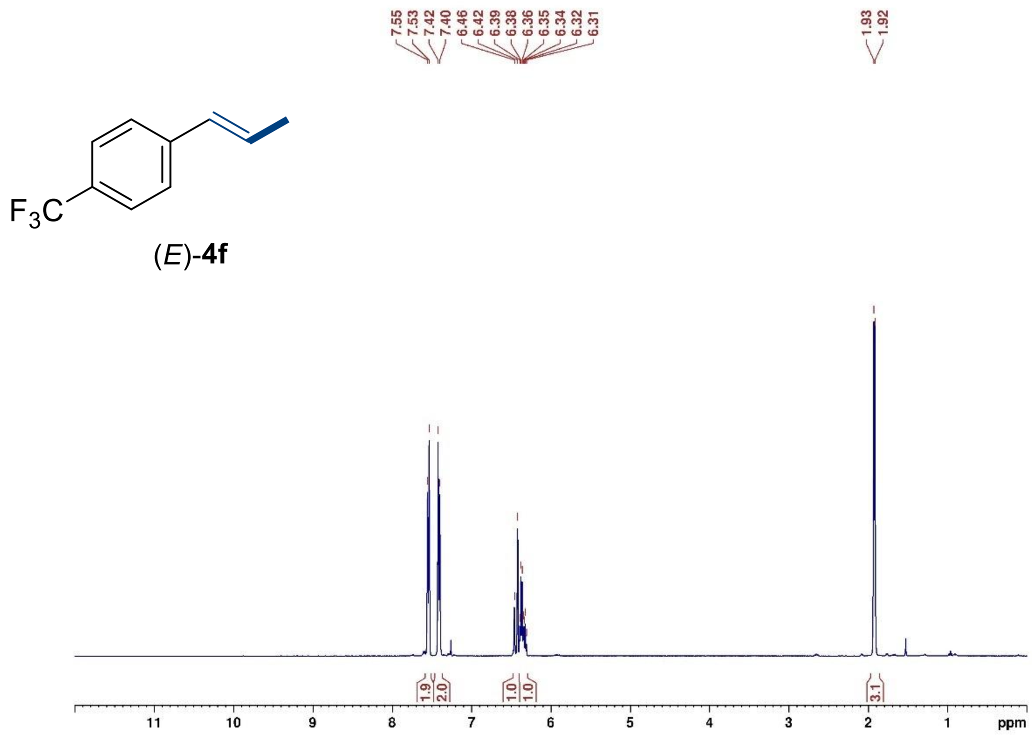

^{13}C NMR spectrum of (*E*)-1-(prop-1-en-1-yl)-4-(trifluoromethyl)benzene (*E*)-**4f** (100 MHz, $CDCl_3$, RT)

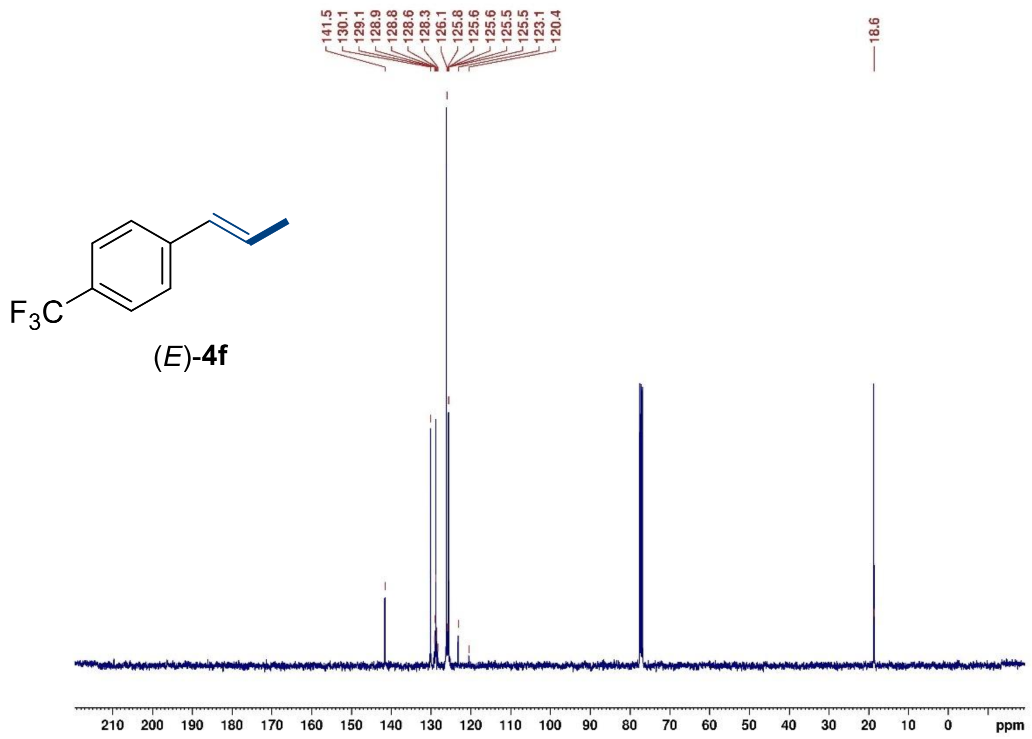

^{19}F NMR spectrum of (*E*)-1-(prop-1-en-1-yl)-4-(trifluoromethyl)benzene (*E*)-**4f** (376 MHz, $CDCl_3$, RT)

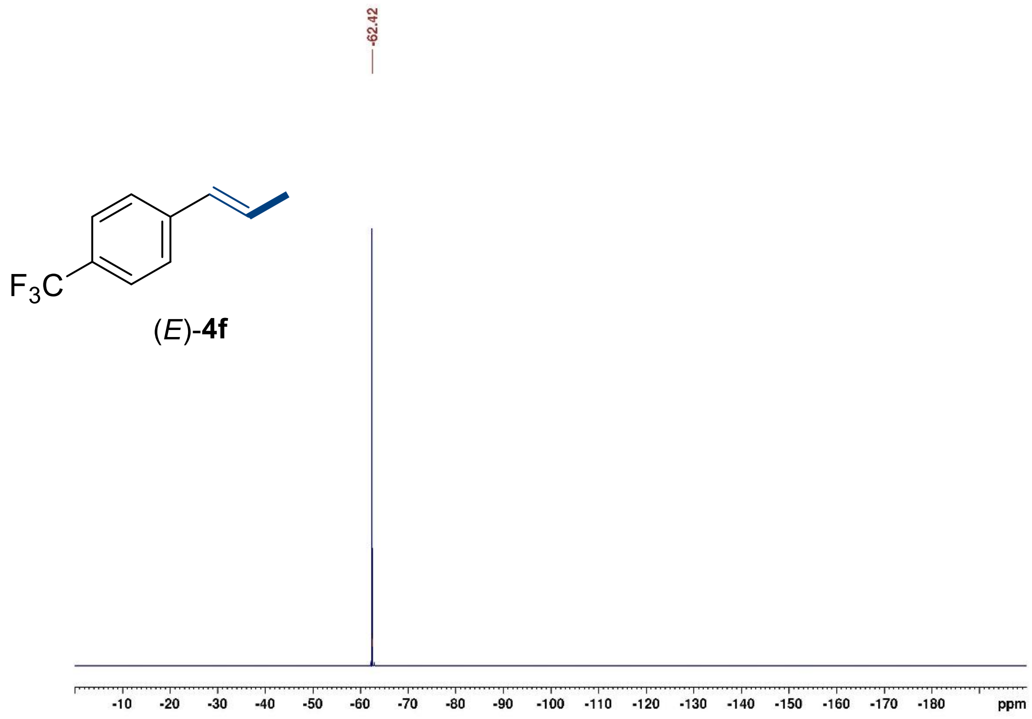

^{1}H NMR spectrum of (*E*)-1-methyl-3-(prop-1-en-1-yl)benzene (*E*)-**4g** (400 MHz, $CDCl_3$, RT)

(*E*)-**4g**

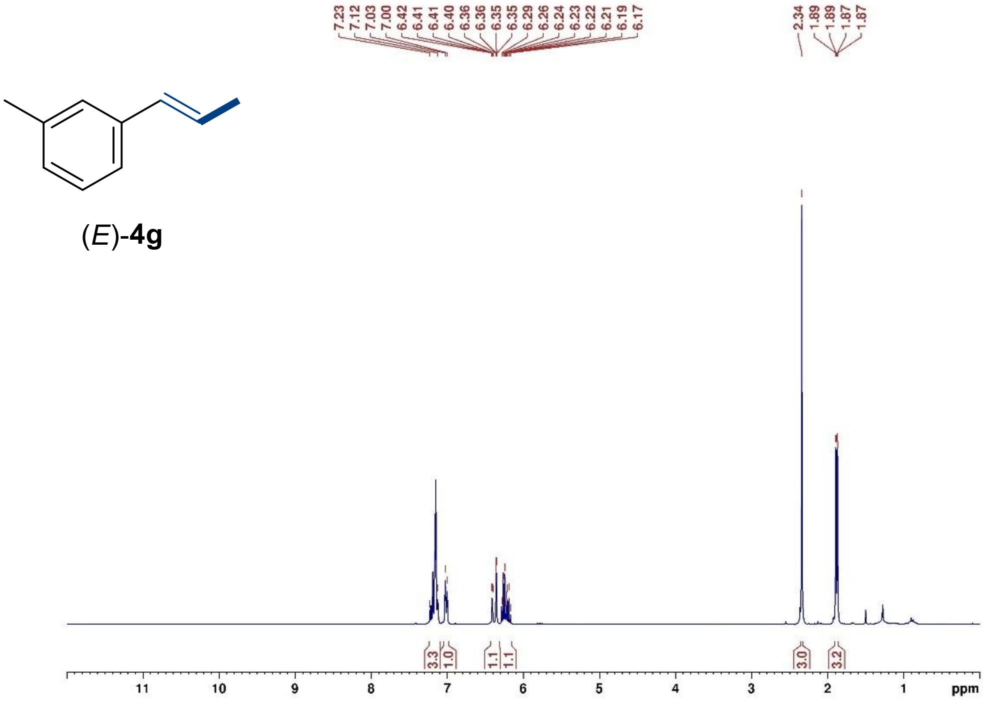

^{13}C NMR spectrum of (*E*)-1-methyl-3-(prop-1-en-1-yl)benzene (*E*)-**4g** (100 MHz, $CDCl_3$, RT)

(*E*)-**4g**

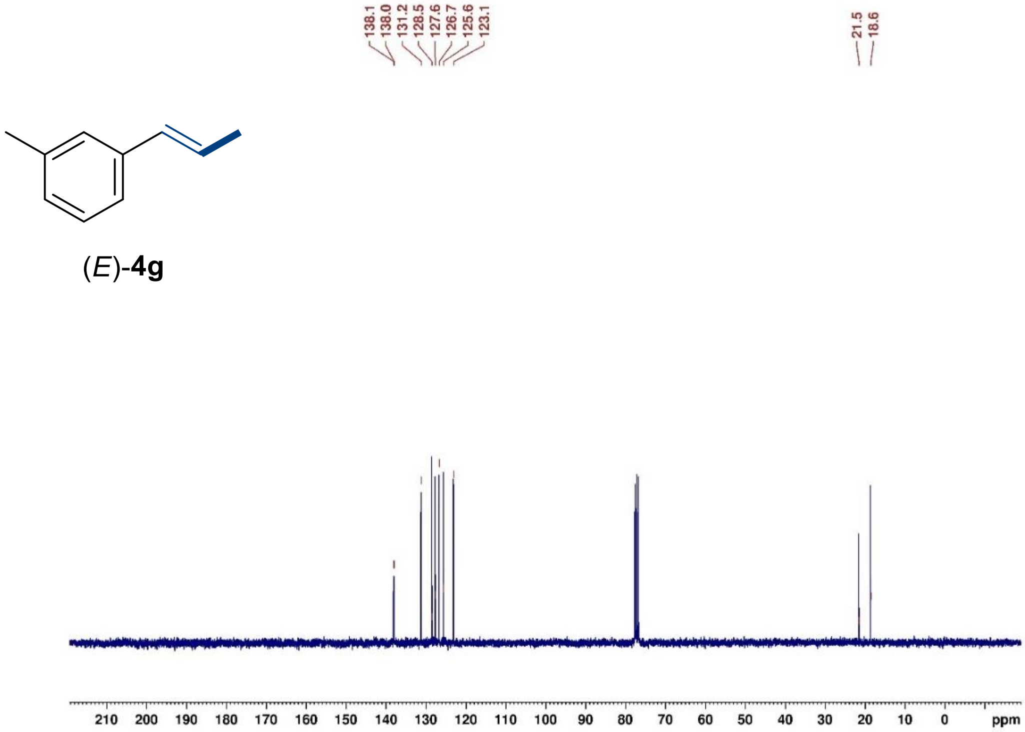

^{1}H NMR spectrum of (*E*)-1-methoxy-3-(prop-1-en-1-yl)benzene (*E*)-**4h** (400 MHz, $CDCl_3$, RT)

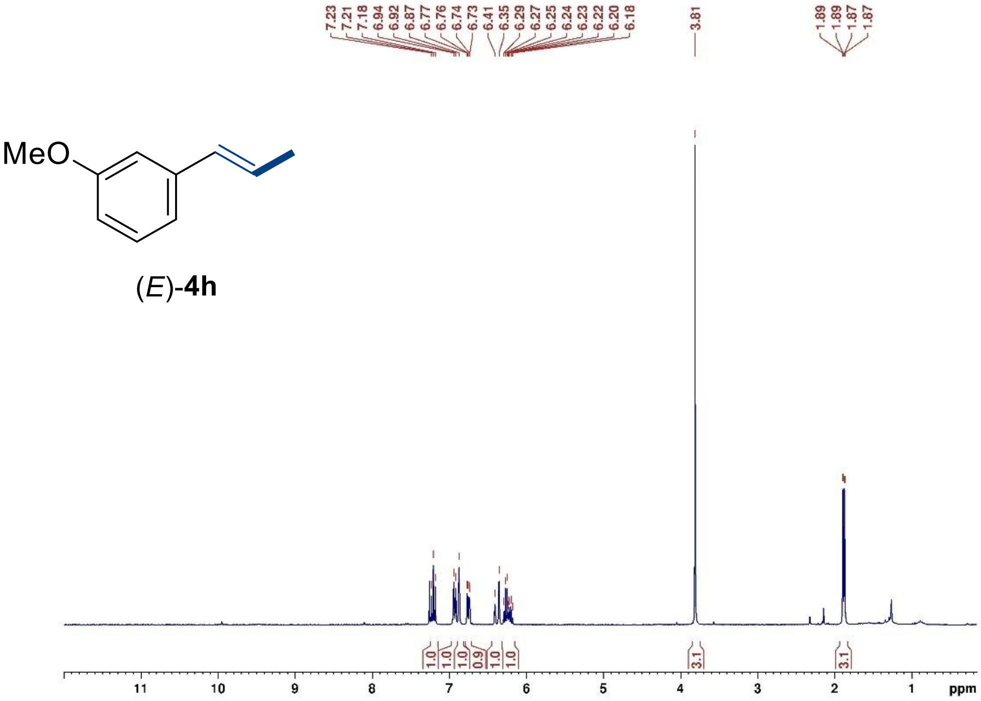

^{13}C NMR spectrum of (*E*)-1-methoxy-3-(prop-1-en-1-yl)benzene (*E*)-**4h** (100 MHz, $CDCl_3$, RT)

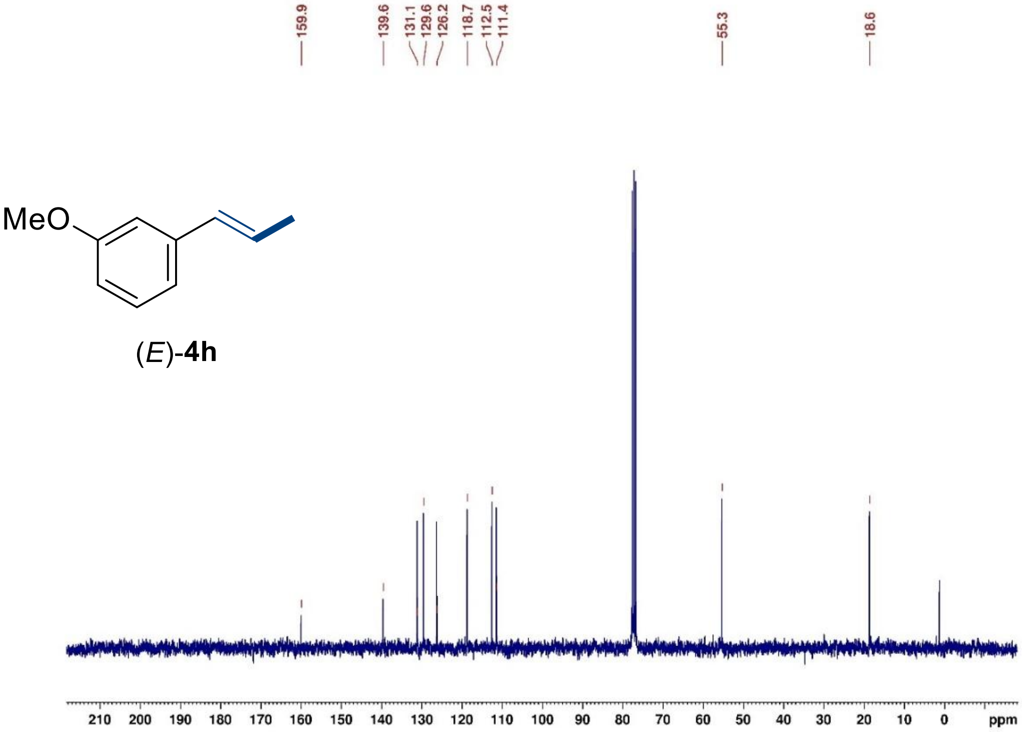

^{1}H NMR spectrum of (*E*)-2-(prop-1-en-1-yl)-1,1'-biphenyl (*E*)-**4i** (400 MHz, $CDCl_3$, RT)

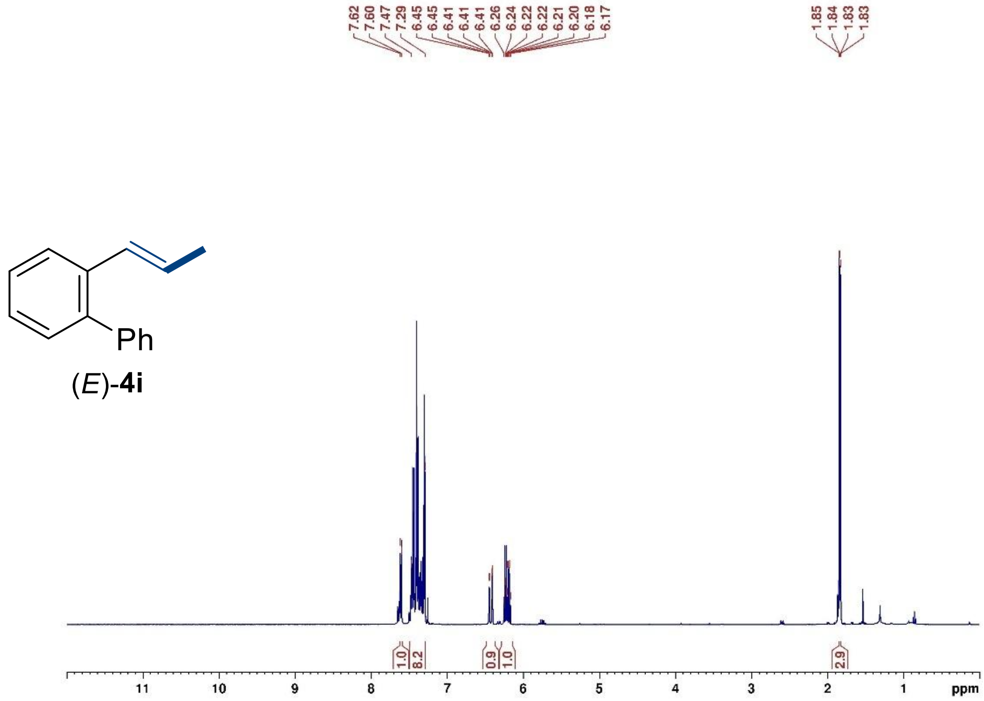

^{13}C NMR spectrum of (*E*)-2-(prop-1-en-1-yl)-1,1'-biphenyl (*E*)-**4i** (100 MHz, $CDCl_3$, RT)

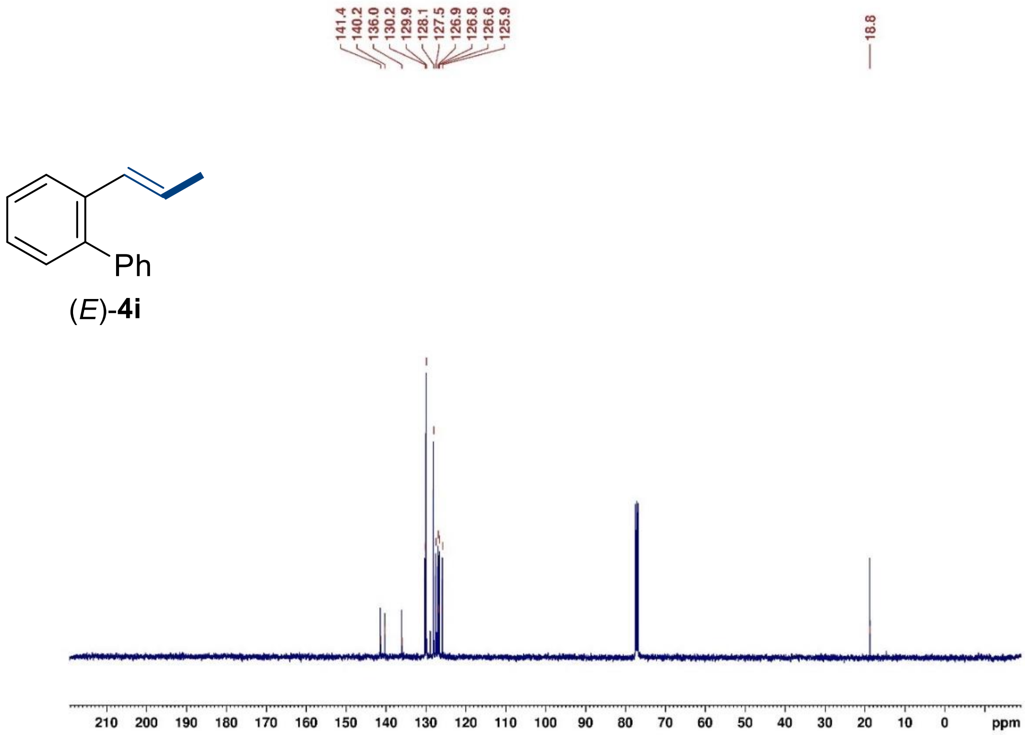

^{1}H NMR spectrum of (*E*)-1-methoxy-2-(prop-1-en-1-yl)benzene (*E*)-**4j** (400 MHz, $CDCl_3$, RT)

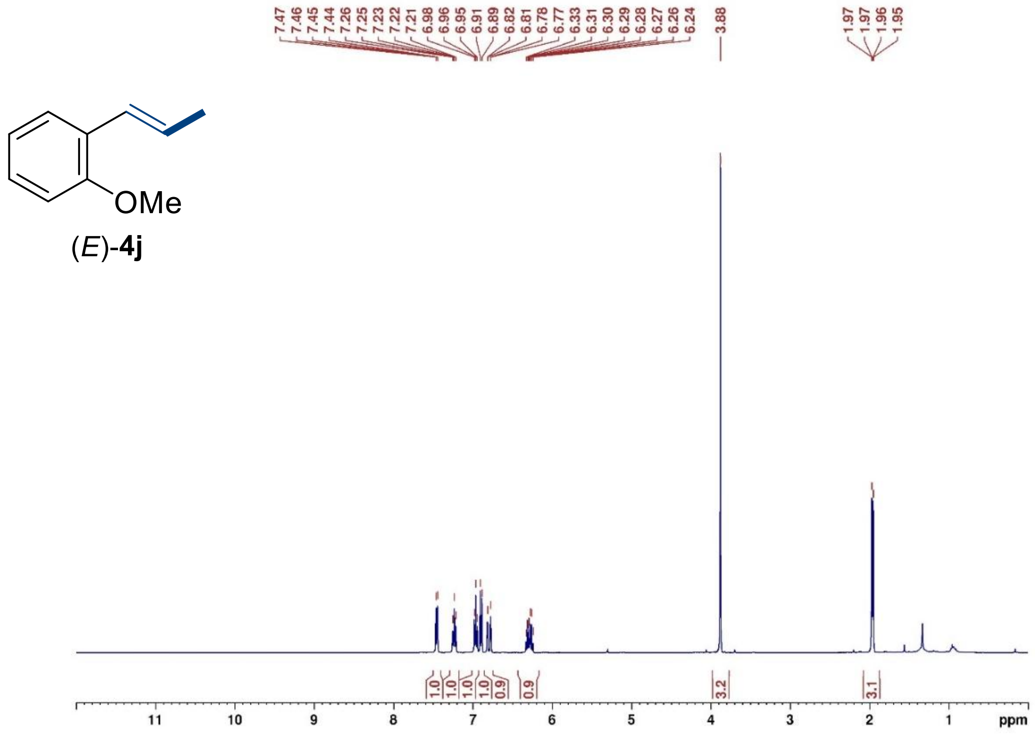

^{13}C NMR spectrum of (*E*)-1-methoxy-2-(prop-1-en-1-yl)benzene (*E*)-**4j** (100 MHz, $CDCl_3$, RT)

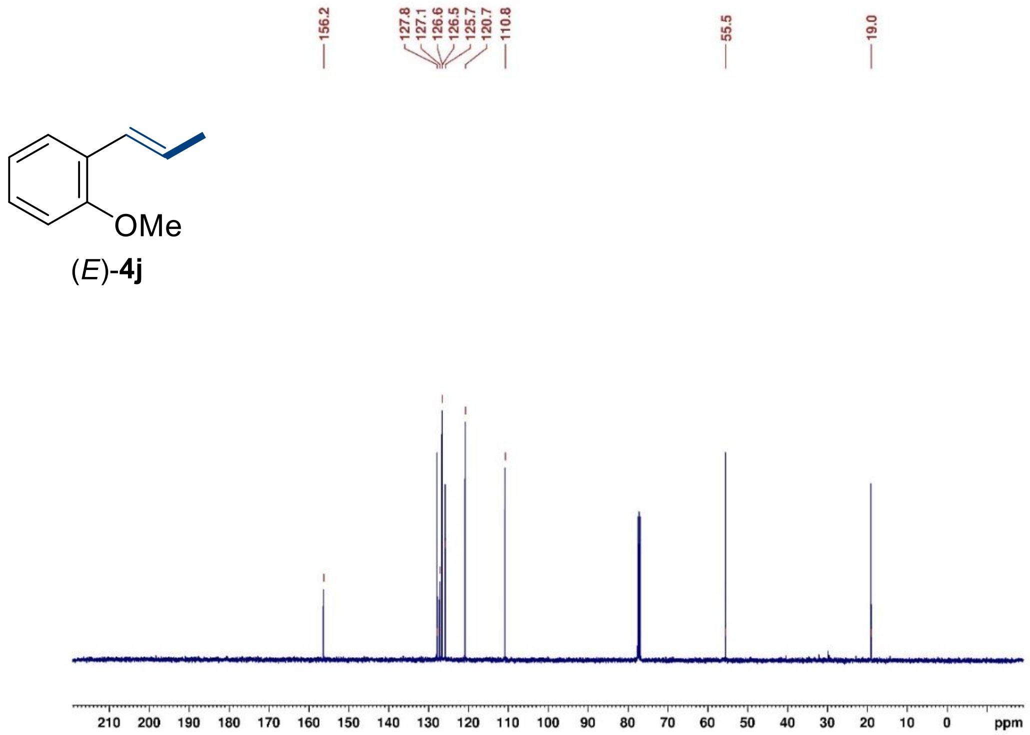

^{1}H NMR spectrum of (*E*)-1,2-dimethoxy-4-(prop-1-en-1-yl)benzene (*E*)-**4l** (400 MHz, $CDCl_3$, RT)

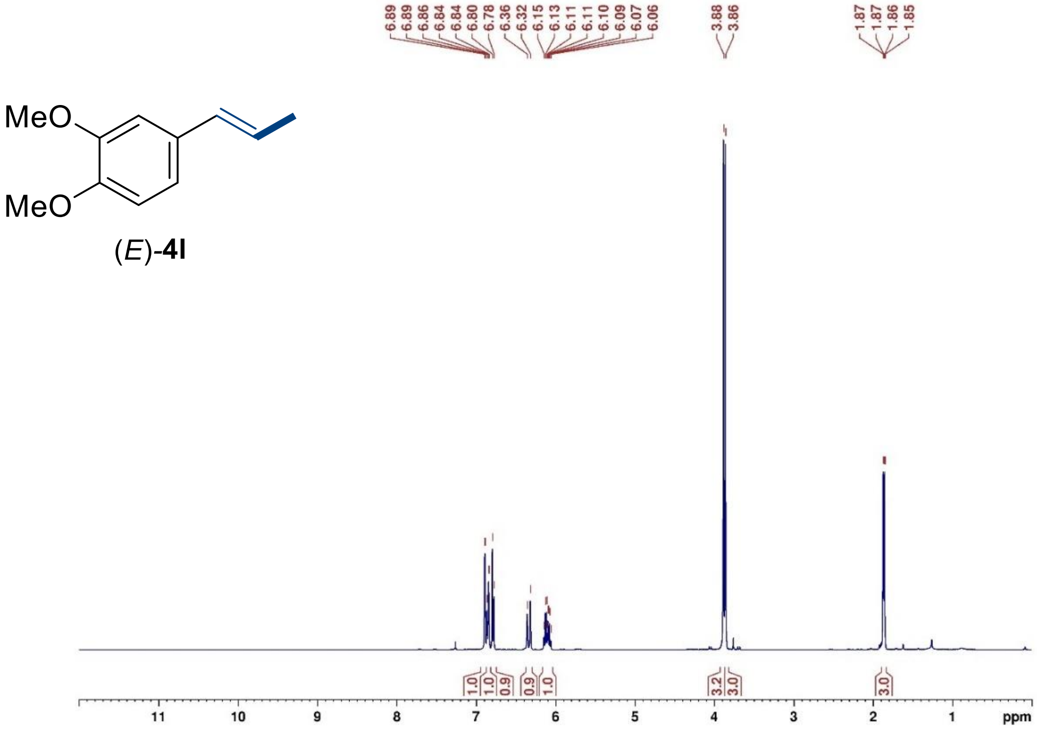

^{13}C NMR spectrum of (*E*)-1,2-dimethoxy-4-(prop-1-en-1-yl)benzene (*E*)-**4l** (100 MHz, $CDCl_3$, RT)

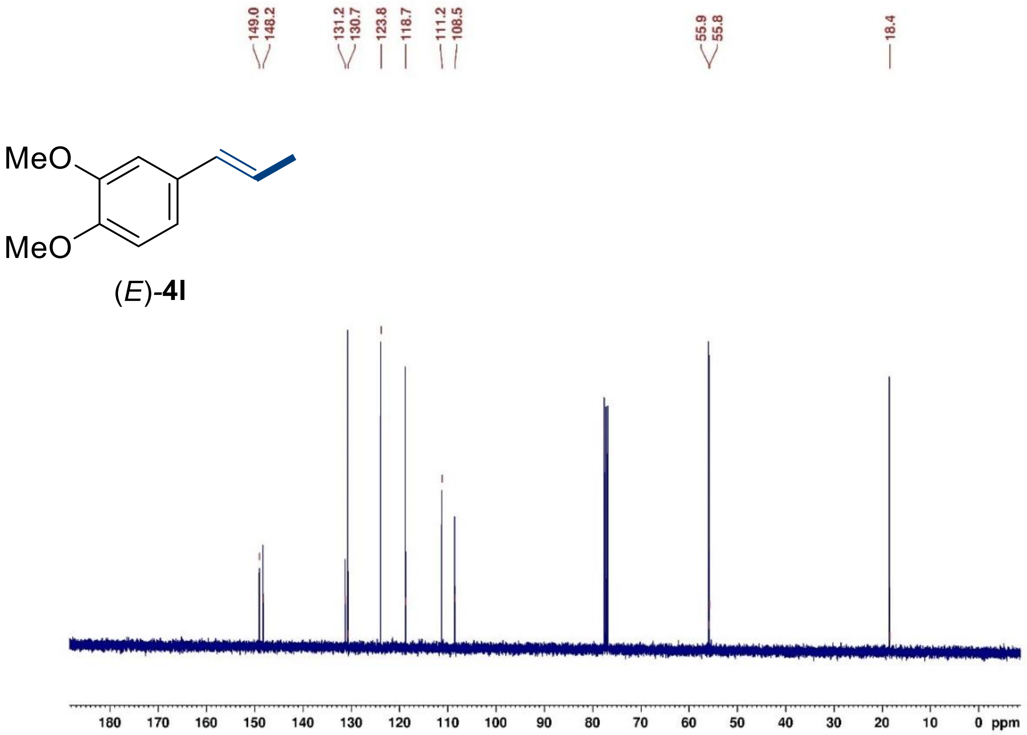

^{1}H NMR spectrum of (*E*)-5-(prop-1-en-1-yl)benzo[*d*][1,3]dioxole (*E*)-**4m** (400 MHz, $CDCl_3$, RT)

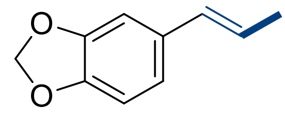

(*E*)-**4m**

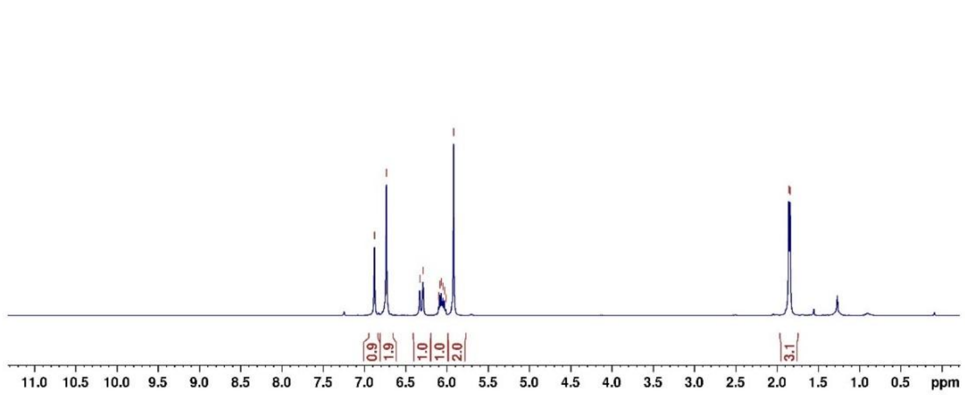

^{13}C NMR spectrum of (*E*)- 5-(prop-1-en-1-yl)benzo[*d*][1,3]dioxole (*E*)-**4m** (100 MHz, $CDCl_3$, RT)

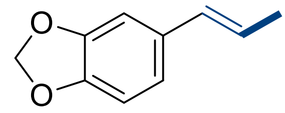

(*E*)-**4m**

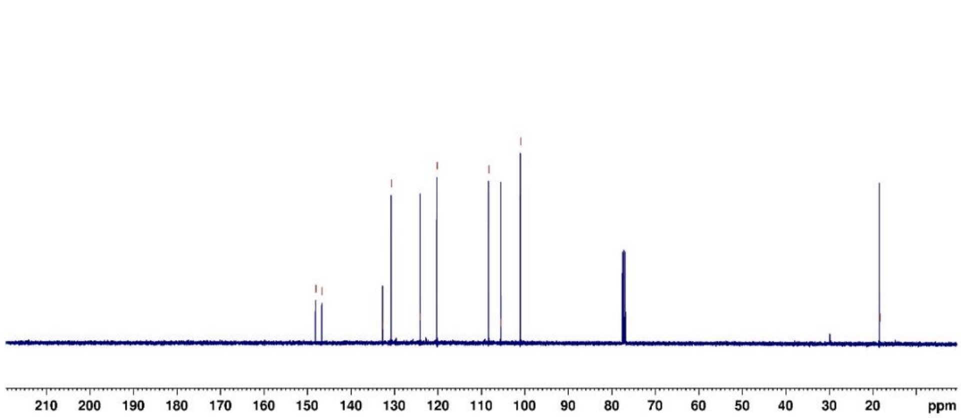

^{1}H NMR spectrum of (*E*)-1,2,3-trimethoxy-5-(prop-1-en-1-yl)benzene (*E*)-**4n** (400 MHz, $CDCl_3$, RT)

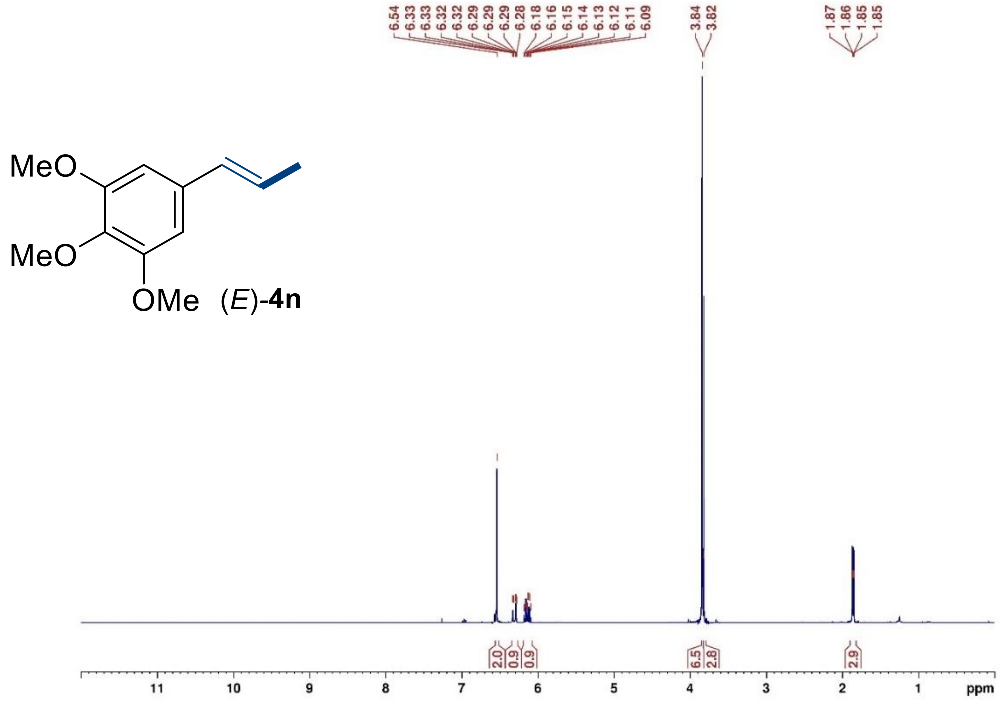

^{13}C NMR spectrum of (*E*)-1,2,3-trimethoxy-5-(prop-1-en-1-yl)benzene (*E*)-**4n** (100 MHz, $CDCl_3$, RT)

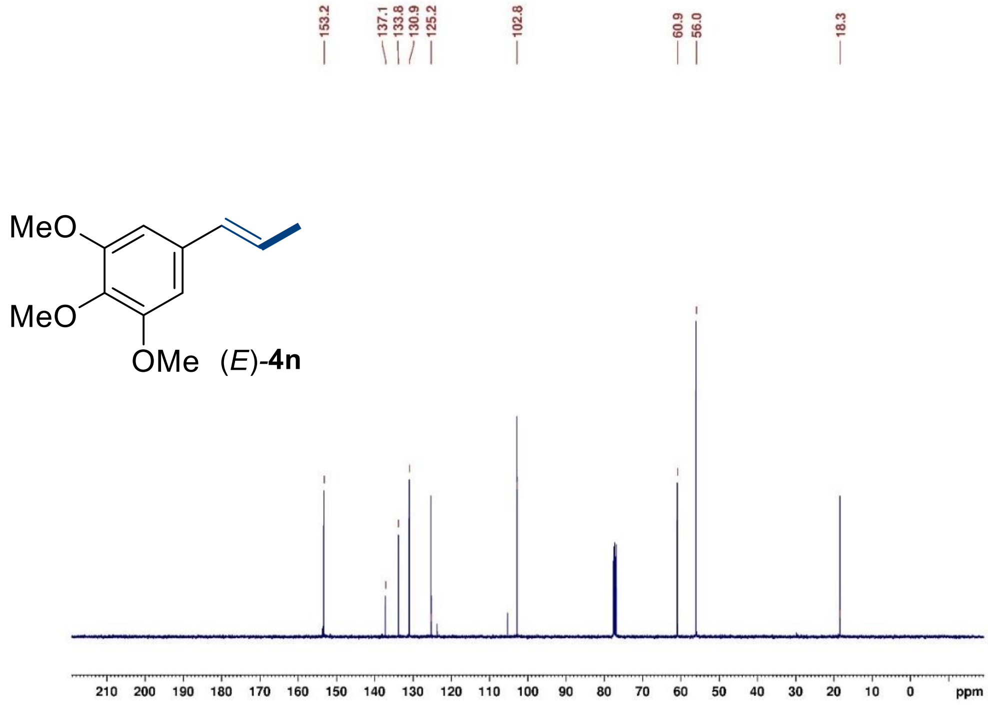

^{1}H NMR spectrum of (*E*)-1,2-dimethoxy-3-(prop-1-en-1-yl)benzene (*E*)-**4o** (400 MHz, $CDCl_3$, RT)

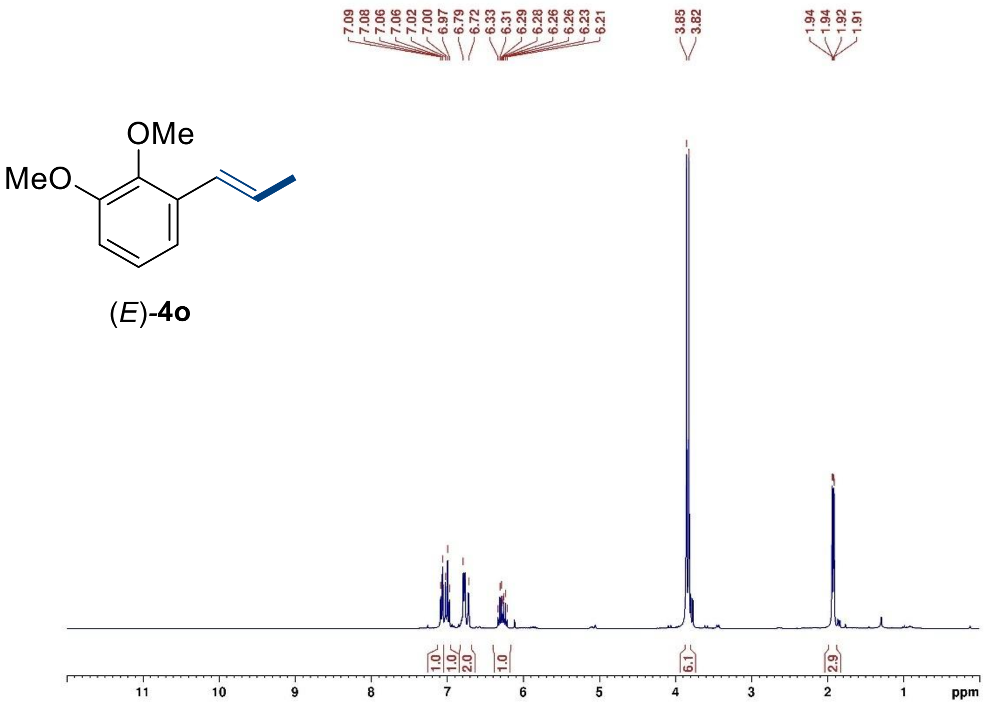

^{13}C NMR spectrum of (*E*)-1,2-dimethoxy-3-(prop-1-en-1-yl)benzene (*E*)-**4o** (100 MHz, $CDCl_3$, RT)

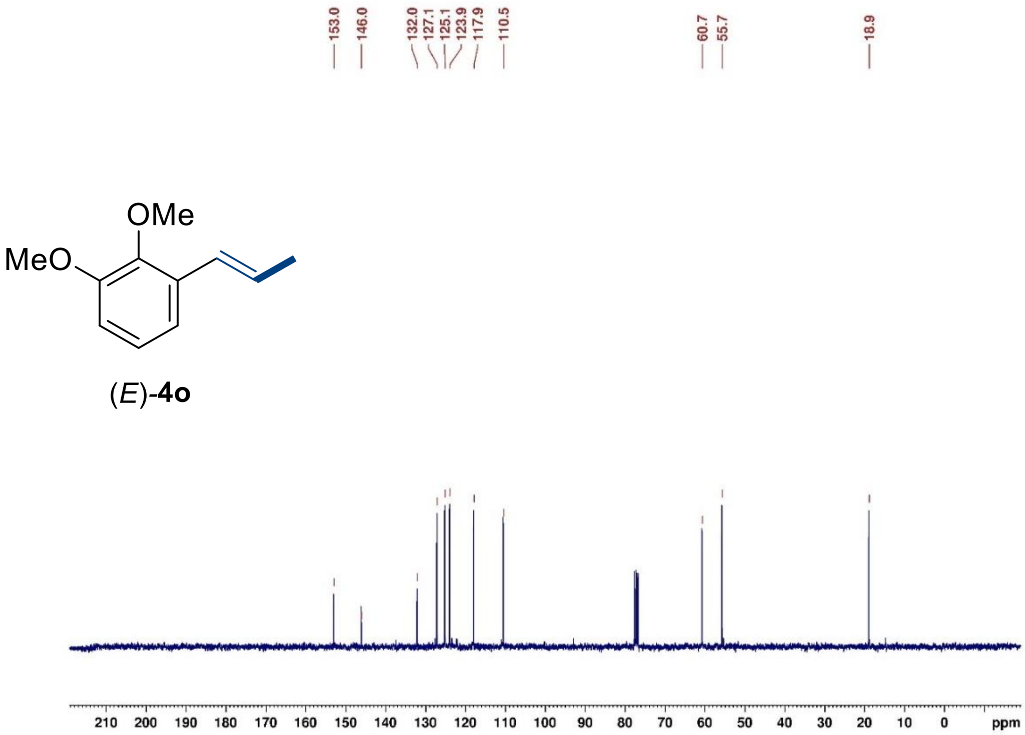

^{1}H NMR spectrum of (*E*)-2-methoxy-1-(methoxymethoxy)-4-(prop-1-en-1-yl)benzene (*E*)-**4p** (400 MHz, $CDCl_3$, RT)

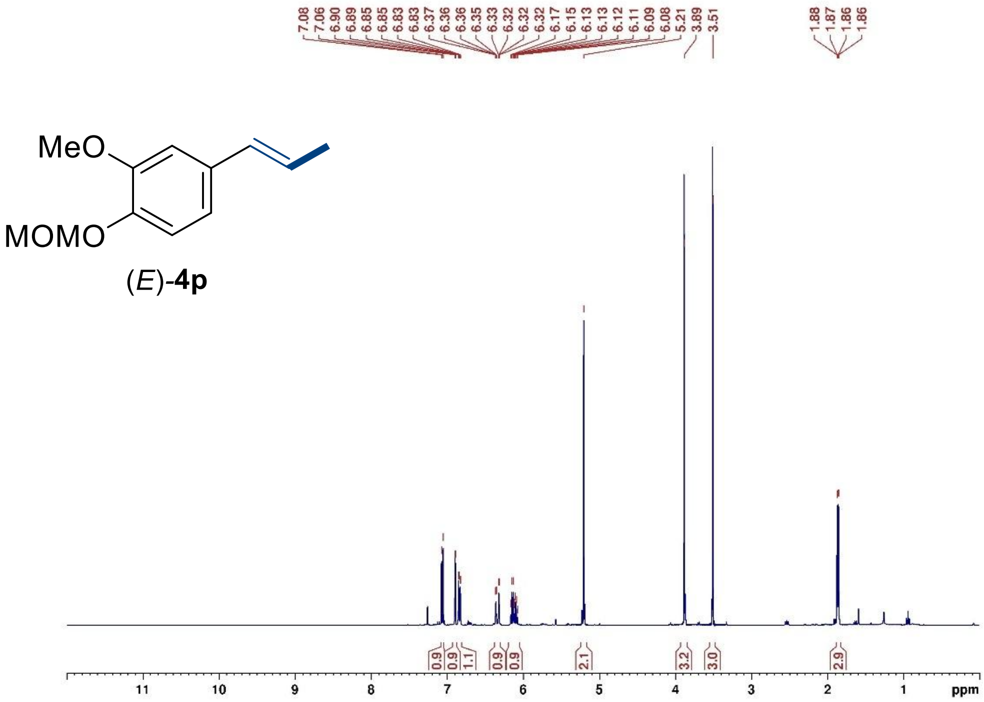

^{13}C NMR spectrum of (*E*)-2-methoxy-1-(methoxymethoxy)-4-(prop-1-en-1-yl)benzene (*E*)-**4p** (100 MHz, $CDCl_3$, RT)

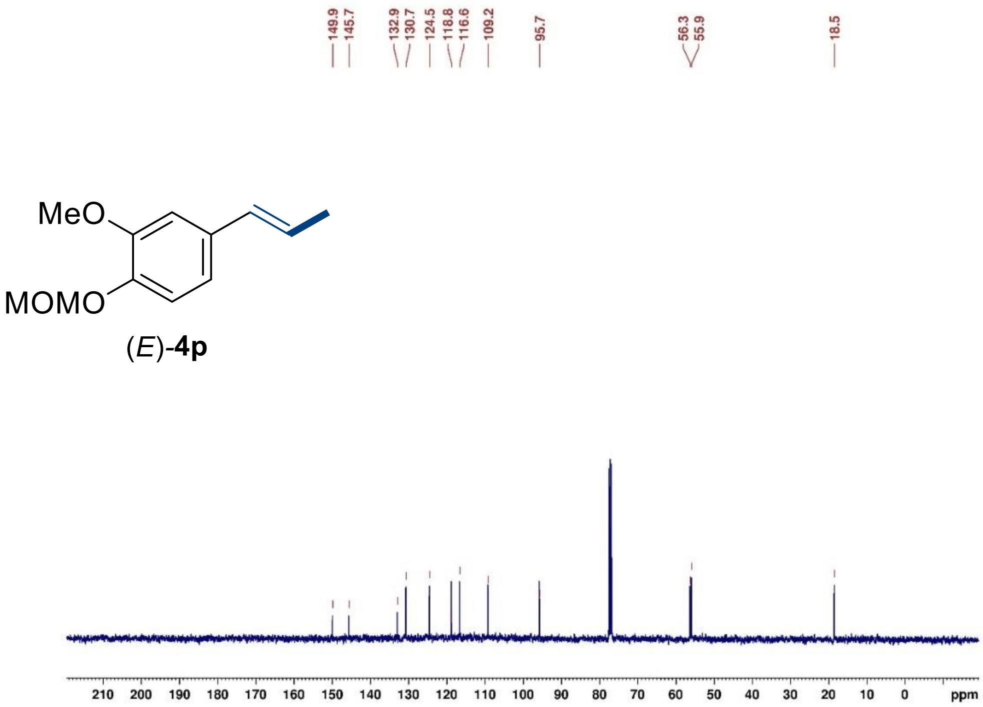

^{1}H NMR spectrum of (*E*)-2-methoxy-1-((4-methoxybenzyl)oxy)-4-(prop-1-en-1-yl)benzene (*E*)-**4q** (400 MHz, $CDCl_3$, RT)

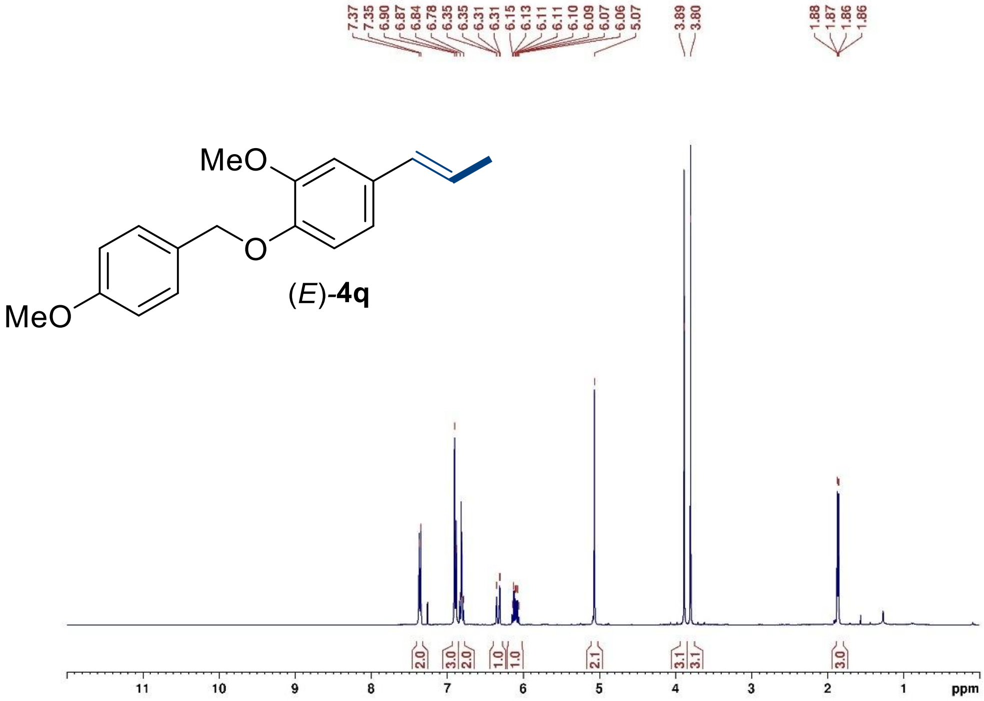

^{13}C NMR spectrum of (*E*)-2-methoxy-1-((4-methoxybenzyl)oxy)-4-(prop-1-en-1-yl)benzene (*E*)-**4q** (100 MHz, $CDCl_3$, RT)

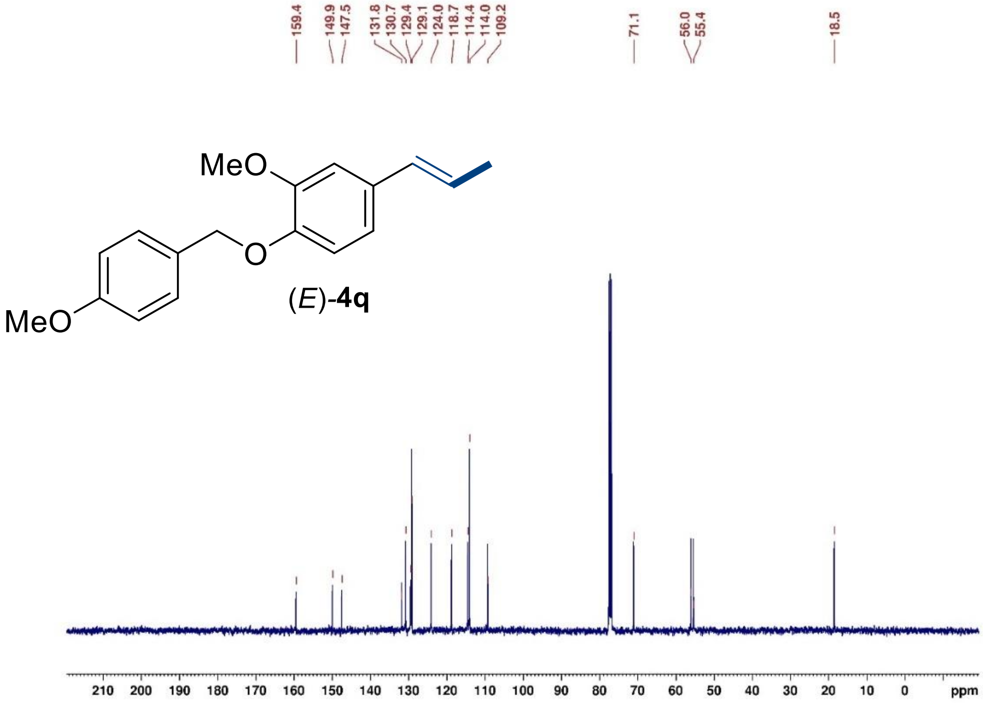

^{1}H NMR spectrum of (*E*)-4-(prop-1-en-1-yl)benzo[*b*]thiophene (*E*)-**4r** (400 MHz, $CDCl_3$, RT)

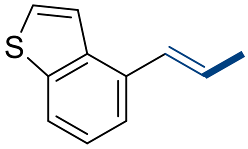

(*E*)-**4r**

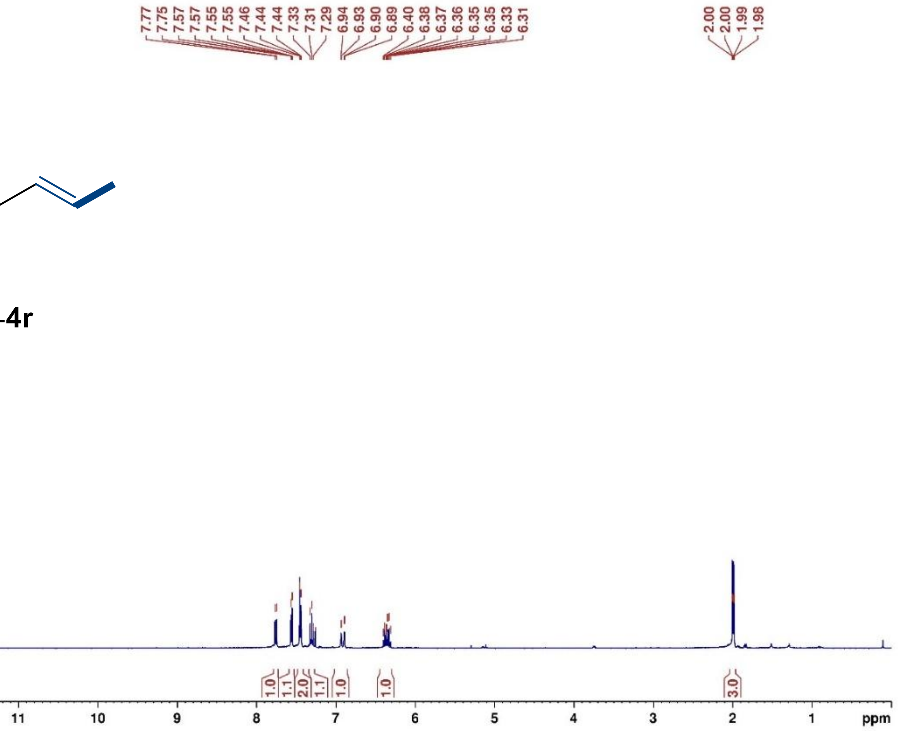

^{13}C NMR spectrum of (*E*)-4-(prop-1-en-1-yl)benzo[*b*]thiophene (*E*)-**4r** (100 MHz, $CDCl_3$, RT)

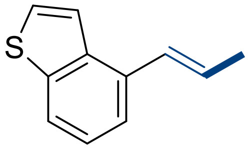

(*E*)-**4r**

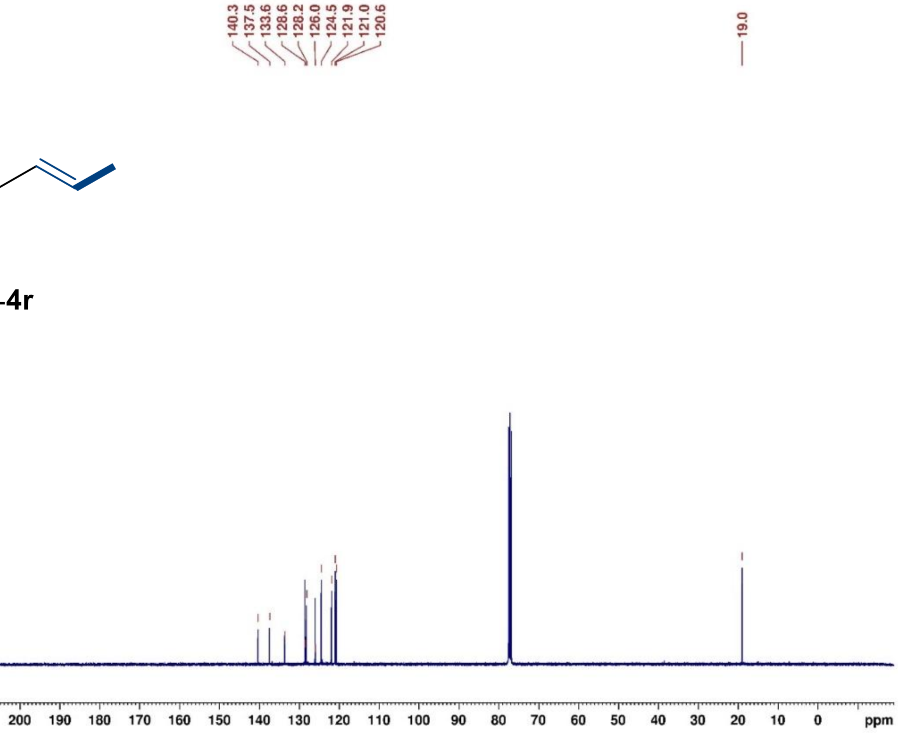

^{1}H NMR spectrum of (*E*)-triphenyl(prop-1-en-1-yl)silane (*E*)-**4s** (400 MHz, $CDCl_3$, RT)

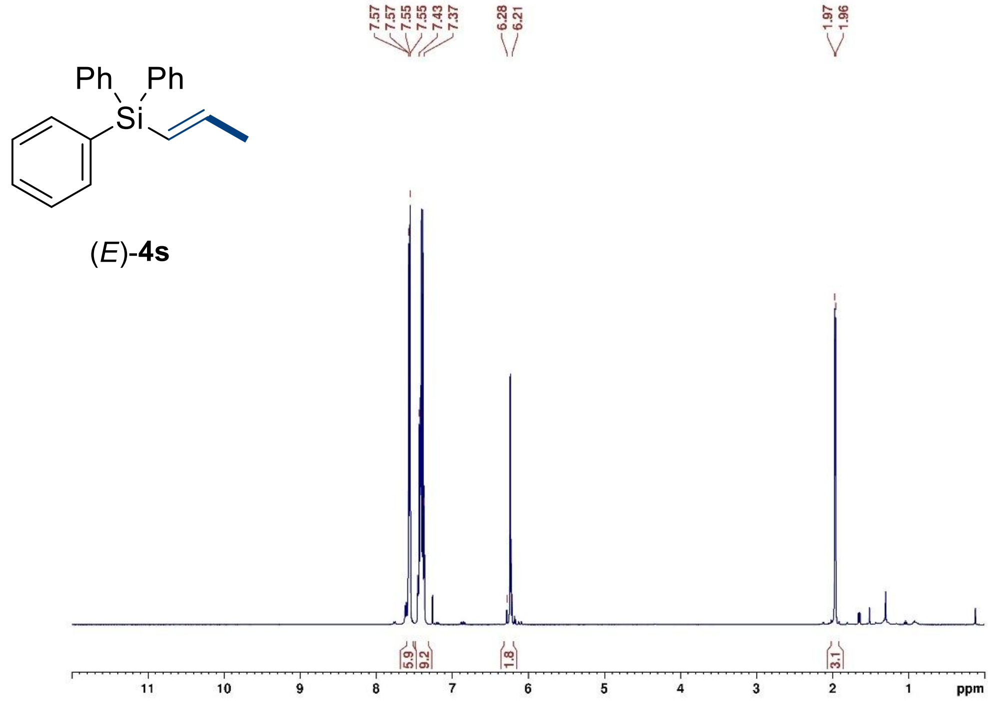

^{13}C NMR spectrum of (*E*)-triphenyl(prop-1-en-1-yl)silane (*E*)-**4s** (100 MHz, $CDCl_3$, RT)

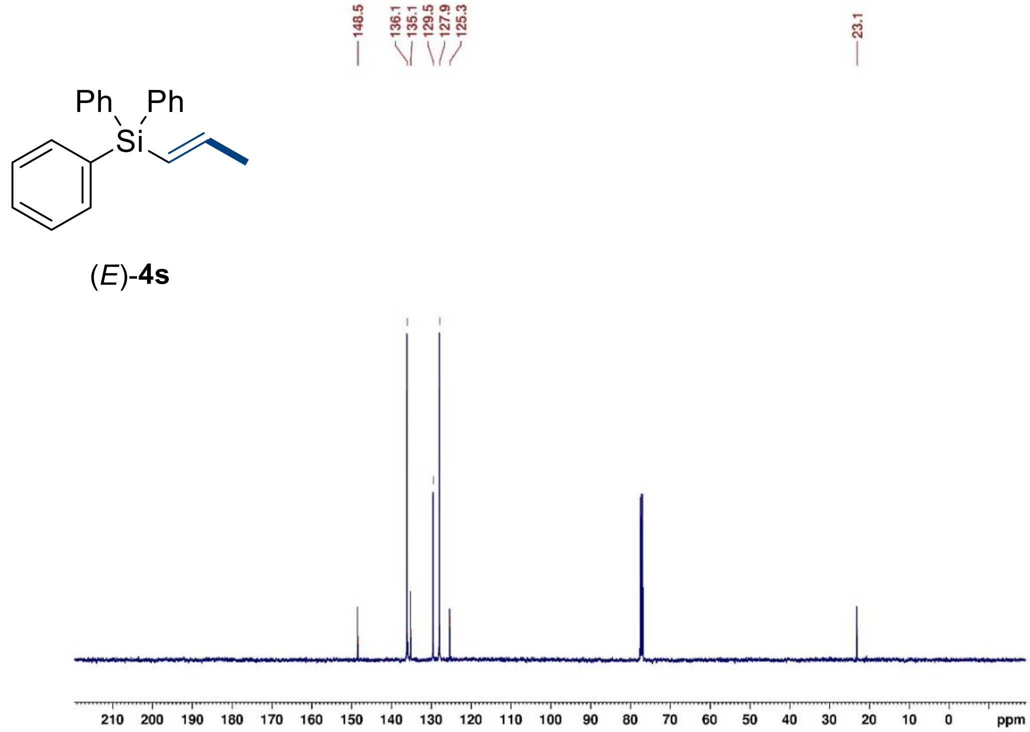

^{1}H NMR spectrum of (2-methylprop-1-en-1-yl)benzene **4aa** (400 MHz, $CDCl_3$, RT)

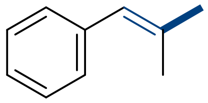

4aa

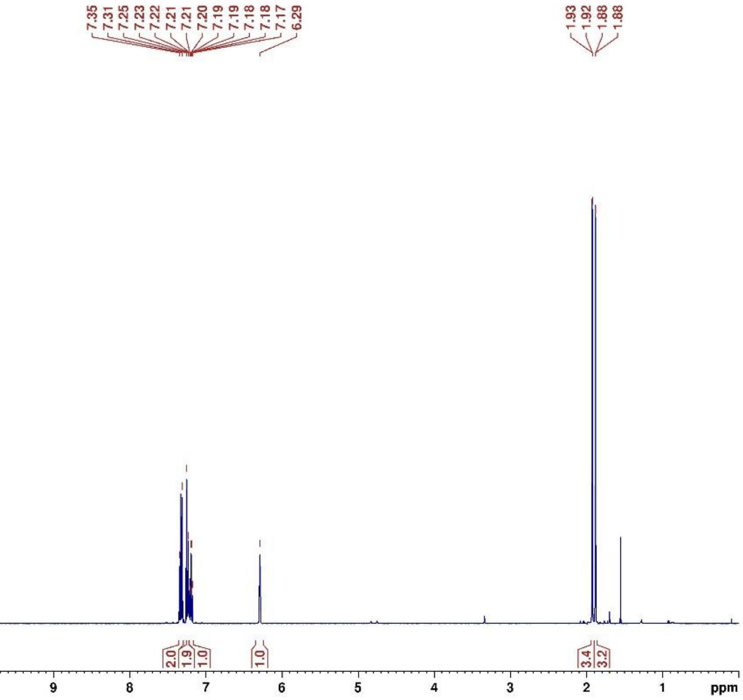

^{13}C NMR spectrum of (2-methylprop-1-en-1-yl)benzene **4aa** (100 MHz, $CDCl_3$, RT)

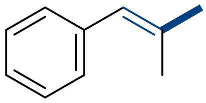

4aa

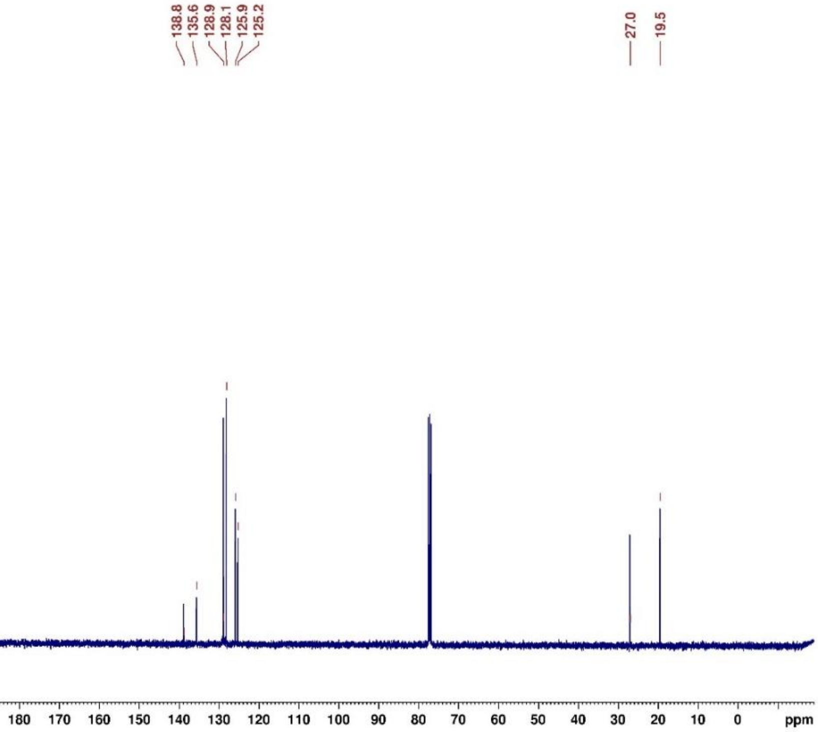

^{1}H NMR spectrum of 2,6-dimethylocta-2,6-diene **4ad** (600 MHz, C_6D_6, RT)

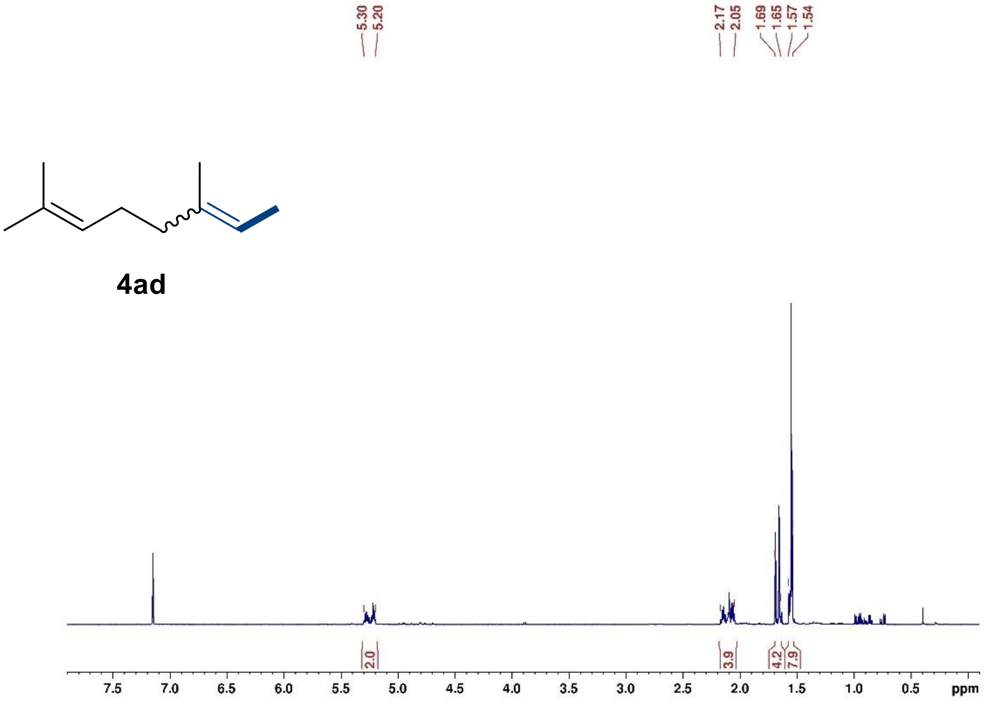

4ad

^{13}C NMR spectrum of 2,6-dimethylocta-2,6-diene **4ad** (150 MHz, C_6D_6, RT)

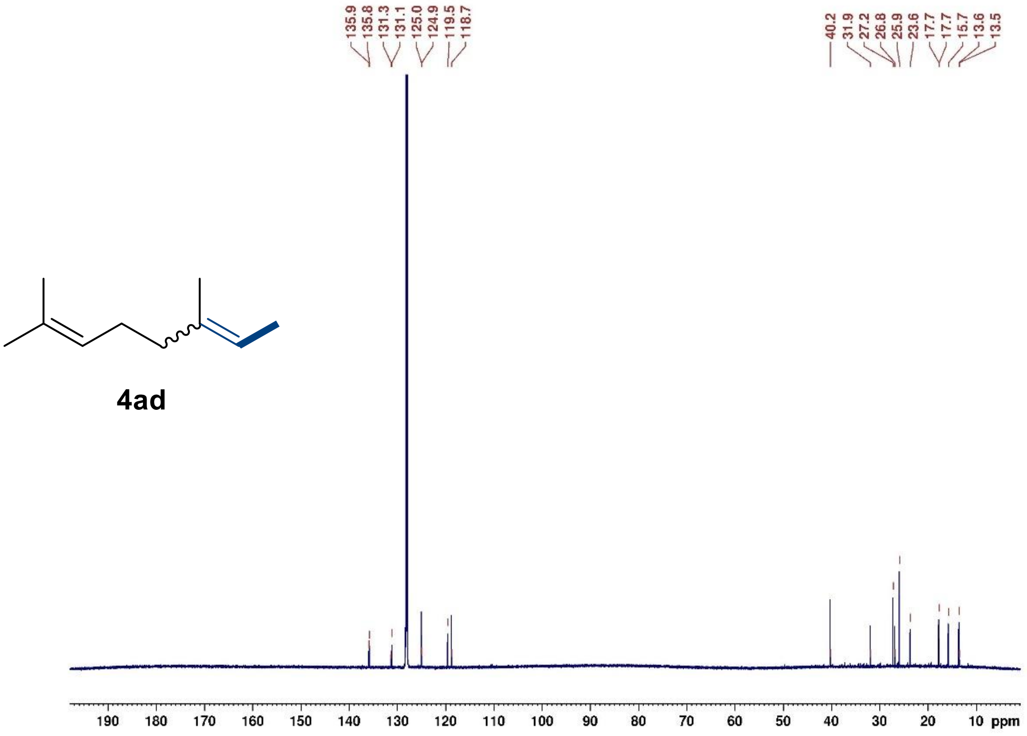

4ad

^{1}H NMR spectrum of 1-(but-2-en-1-yl)-4-methylbenzene **4ae** (400 MHz, $CDCl_3$, RT)

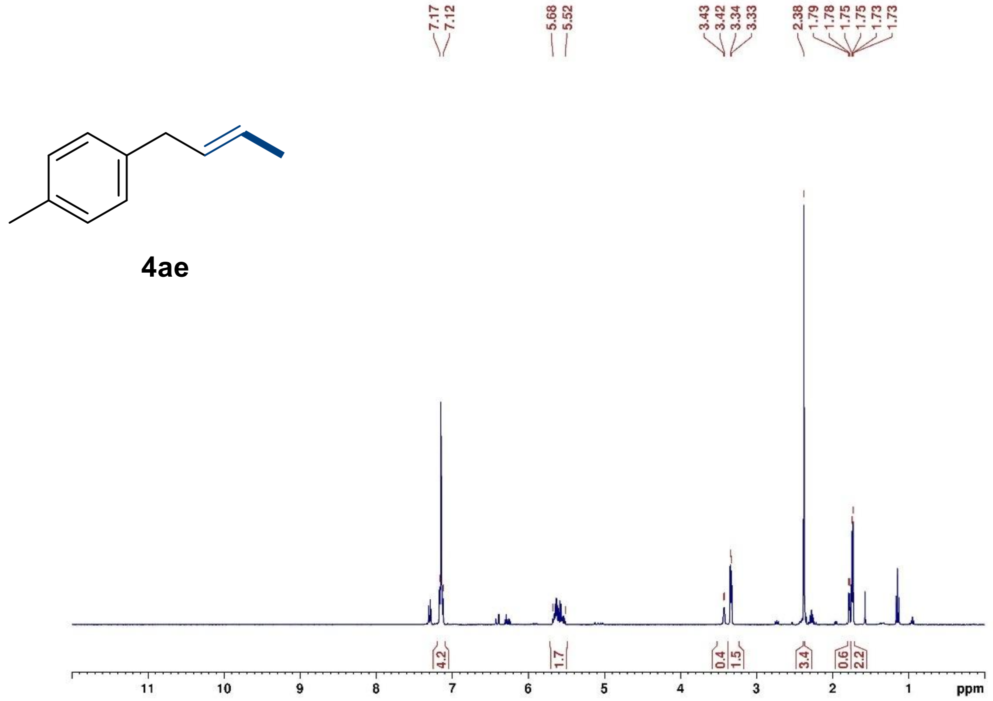

^{13}C NMR spectrum of 1-(but-2-en-1-yl)-4-methylbenzene **4ae** (100 MHz, $CDCl_3$, RT)

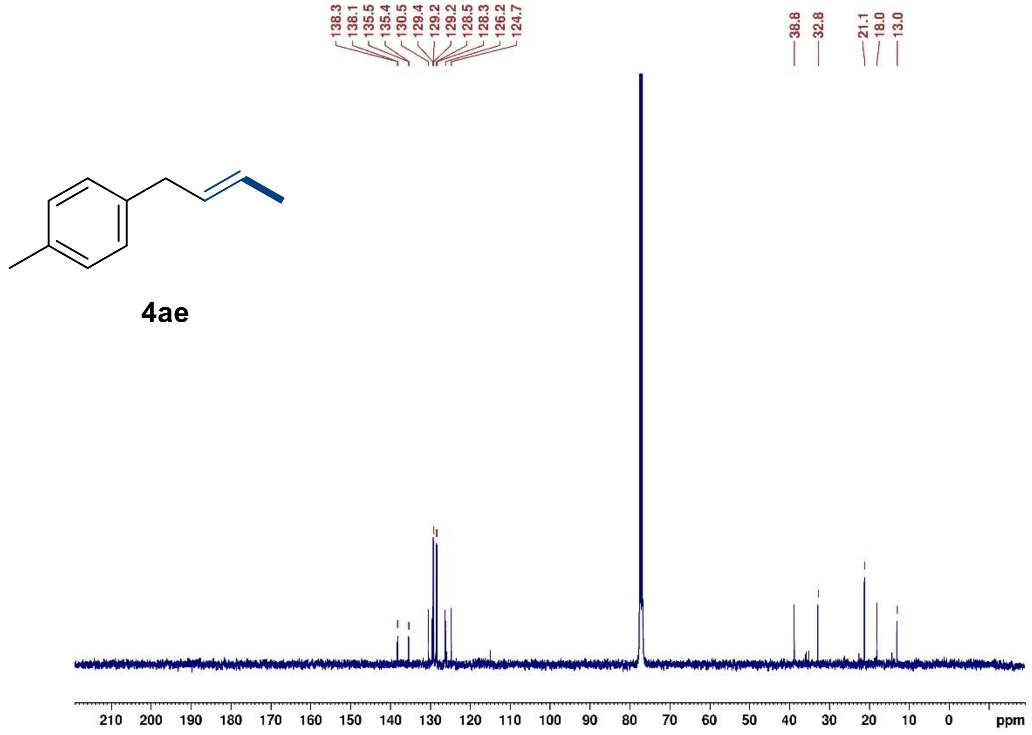

^{1}H NMR spectrum of (*E*)-1-(but-1-en-1-yl)-4-methylbenzene **4ae'** (400 MHz, $CDCl_3$, RT)

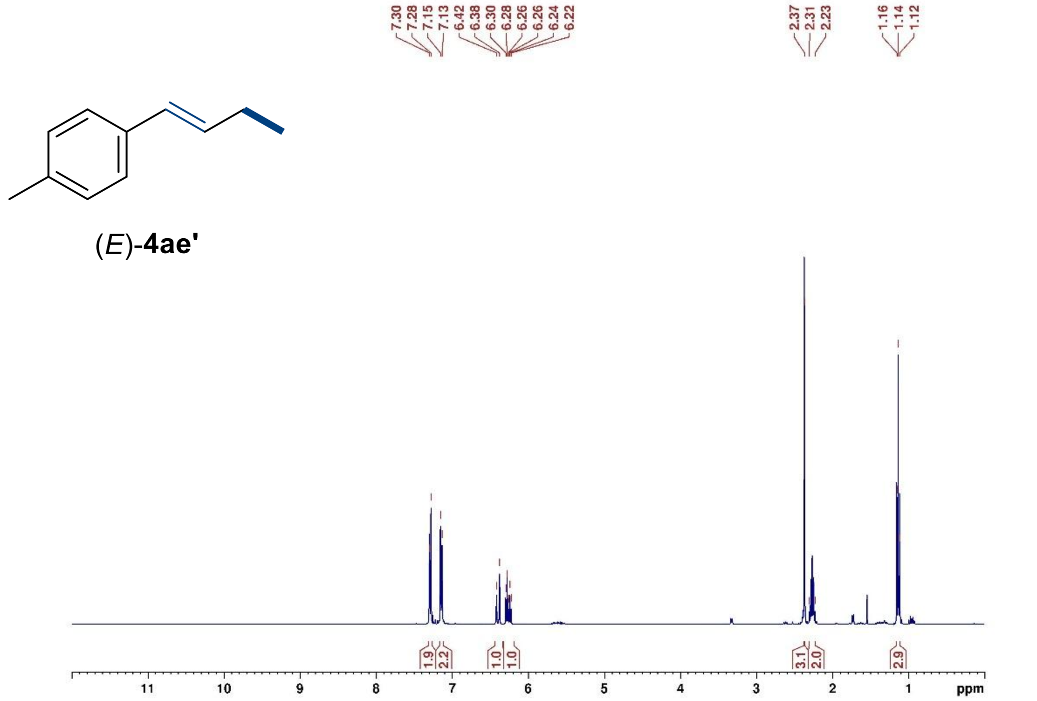

^{13}C NMR spectrum of (*E*)-1-(but-1-en-1-yl)-4-methylbenzene **4ae’** (100 MHz, $CDCl_3$, RT)

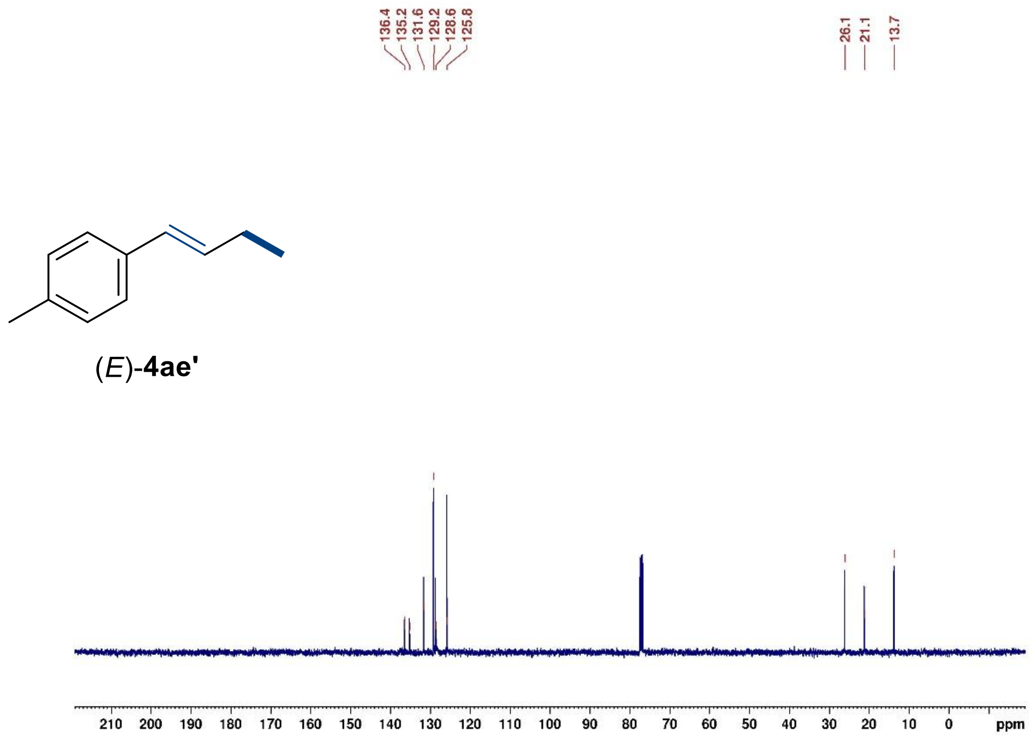

^{1}H NMR spectrum of but-2-en-1-ylbenzene **4af** (400 MHz, $CDCl_3$, RT)

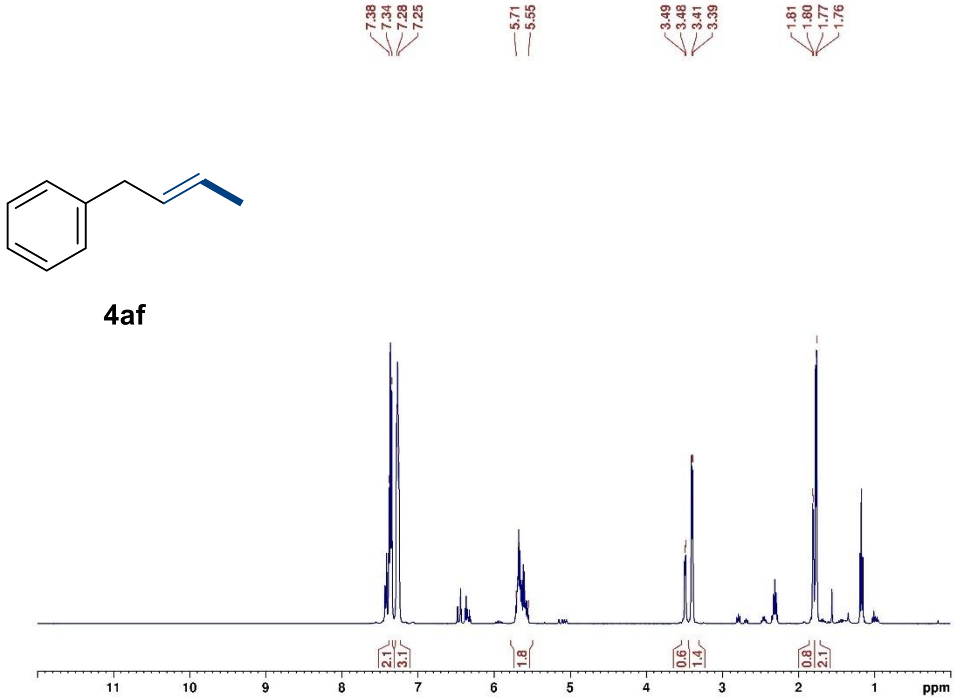

^{13}C NMR spectrum of but-2-en-1-ylbenzene **4af** (100 MHz, $CDCl_3$, RT)

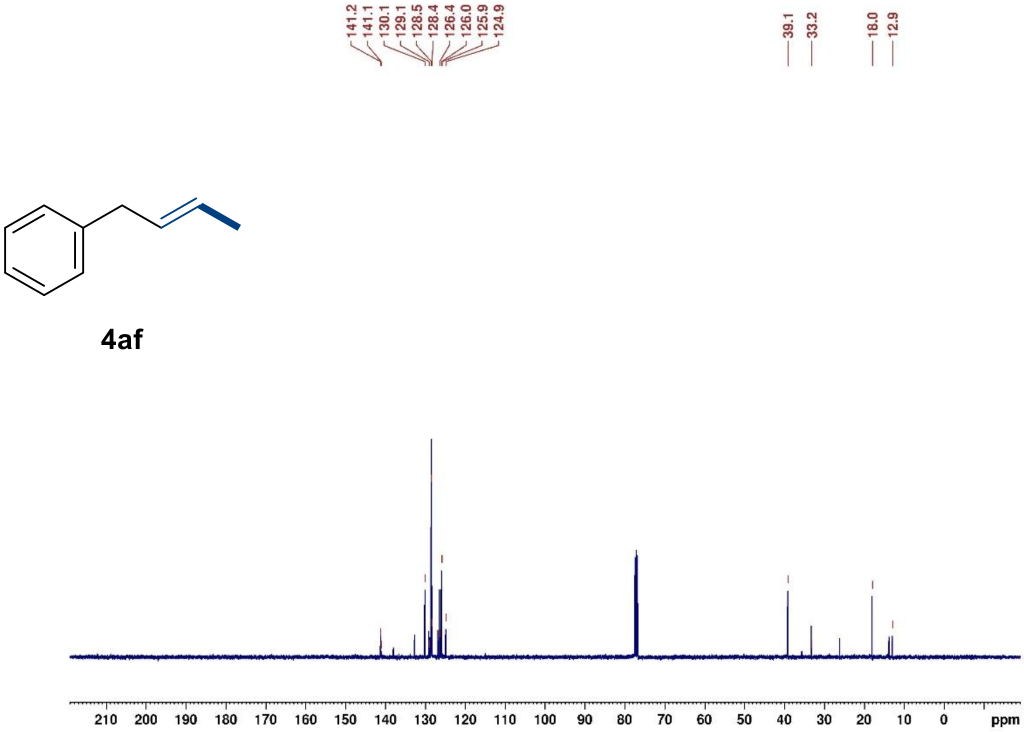

^{1}H NMR spectrum of (*E*)-but-1-en-1-ylbenzene **4af'** (400 MHz, $CDCl_3$, RT)

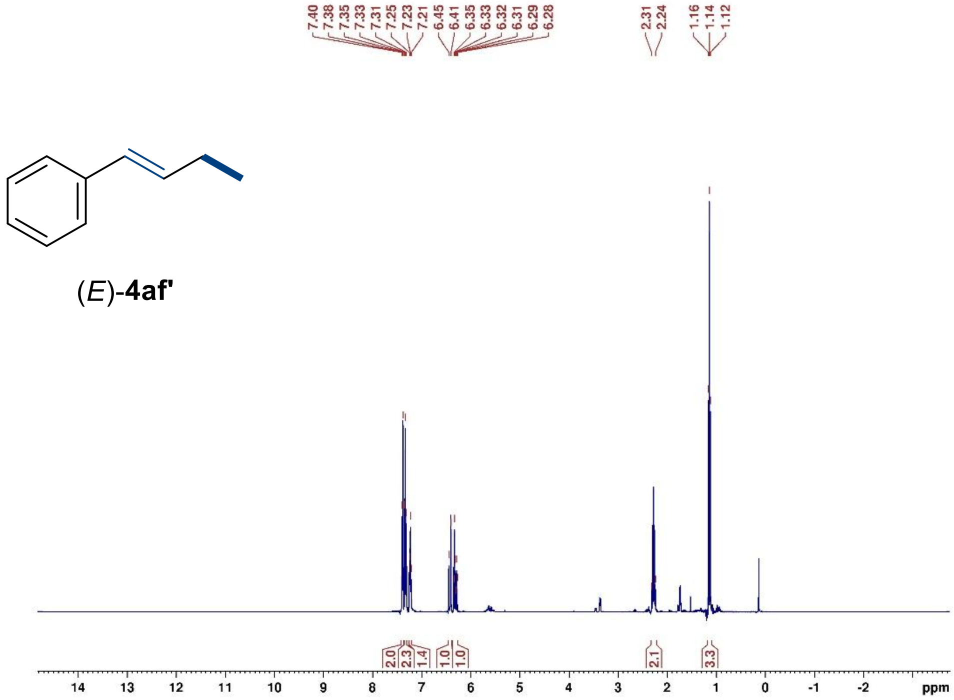

^{13}C NMR spectrum of (*E*)-but-1-en-1-ylbenzene **4af’** (100 MHz, $CDCl_3$, RT)

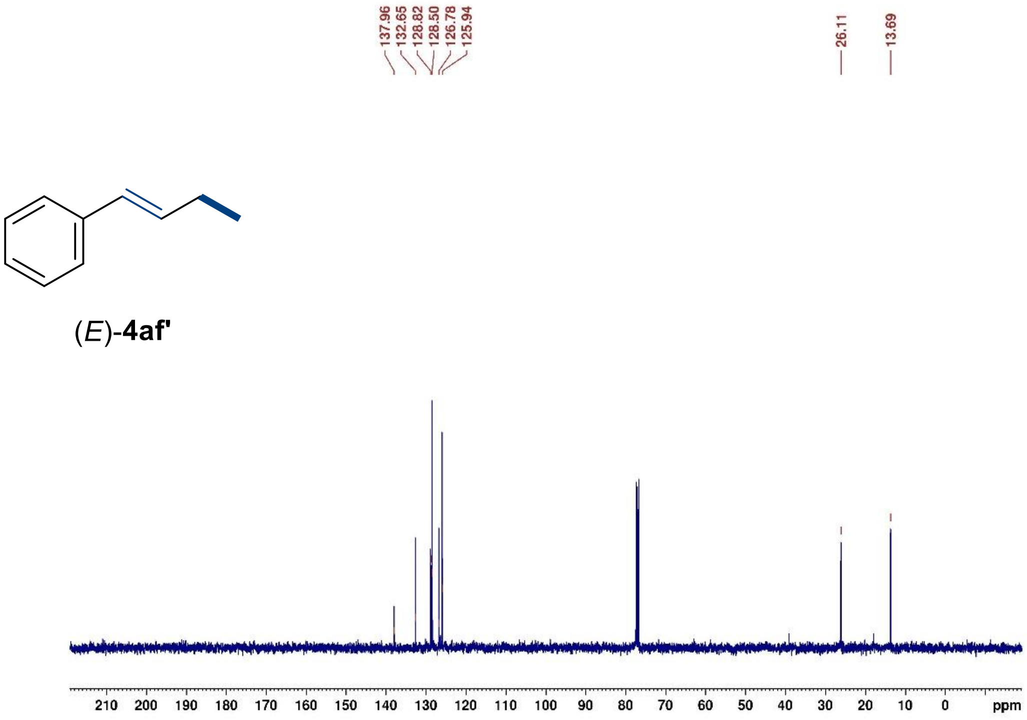

^{1}H NMR spectrum of but-2-ene-1,1-diyldibenzene **4ag** (400 MHz, $CDCl_3$, RT)

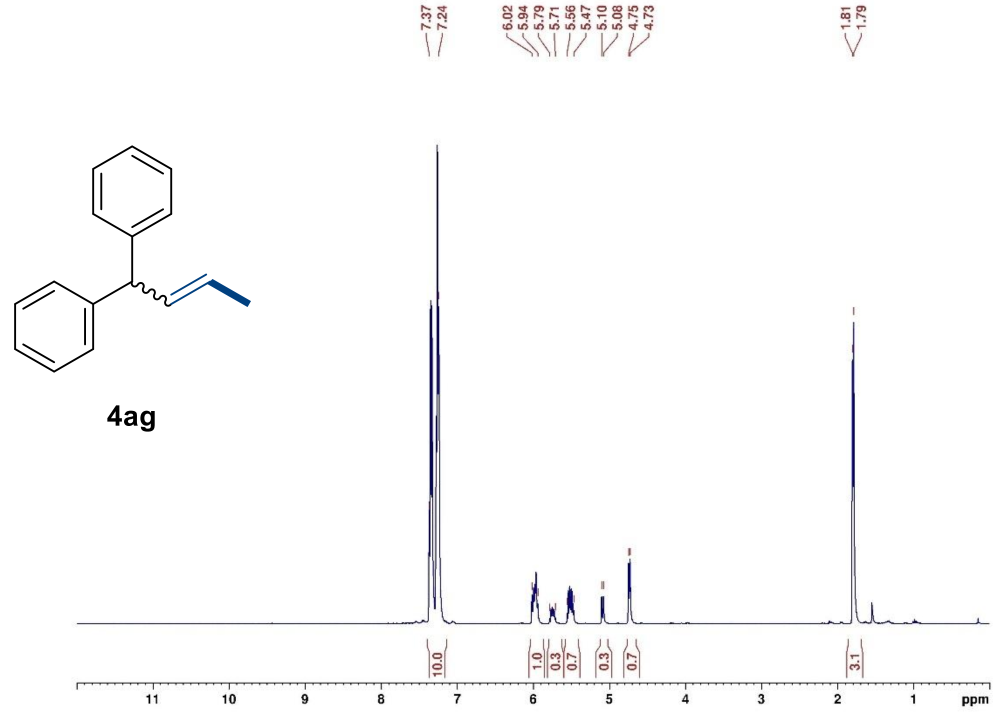

^{13}C NMR spectrum of but-2-ene-1,1-diyldibenzene **4ag** (100 MHz, $CDCl_3$, RT)

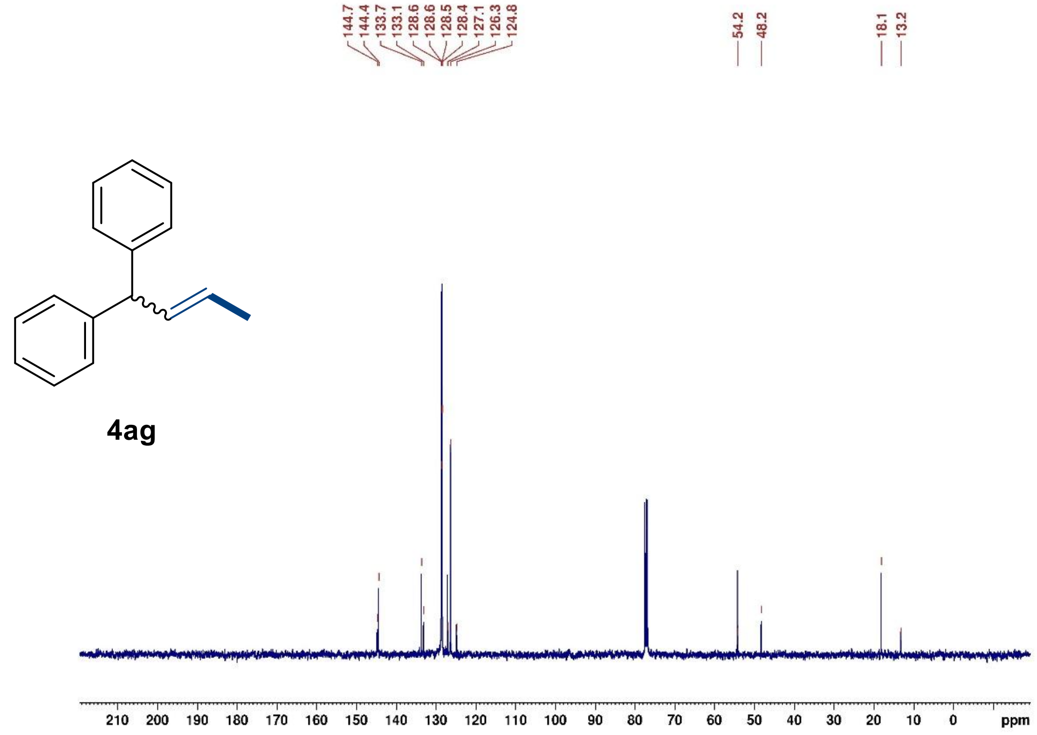

^{1}H NMR spectrum of pent-3-en-1-ylbenzene **4ah** (400 MHz, $CDCl_3$, RT)

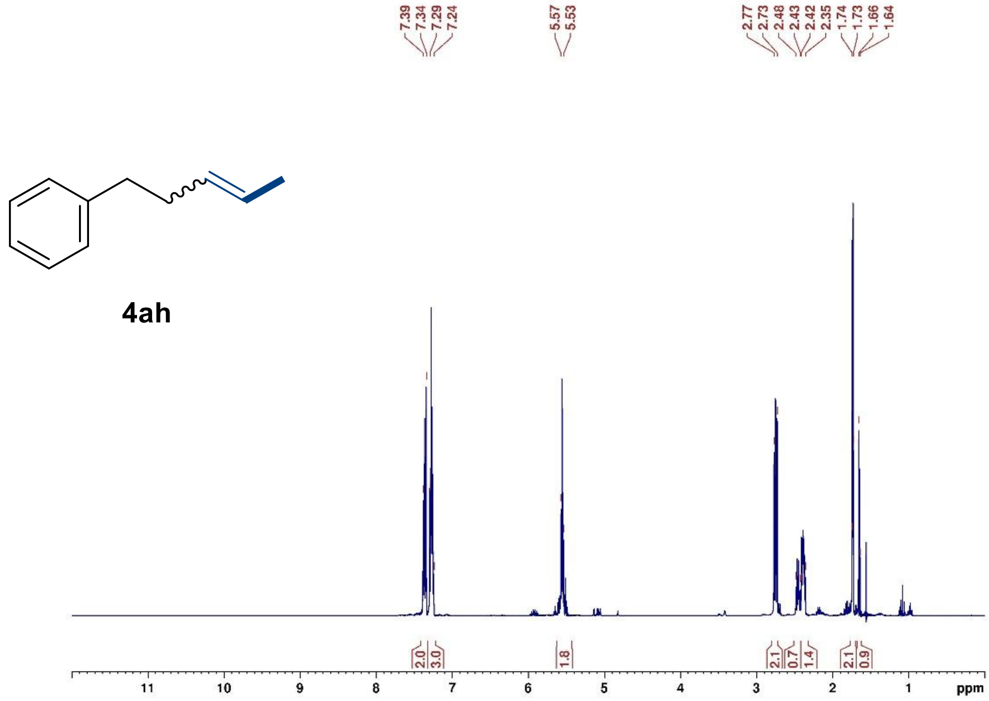

^{13}C NMR spectrum of pent-3-en-1-ylbenzene **4ah** (100 MHz, $CDCl_3$, RT)

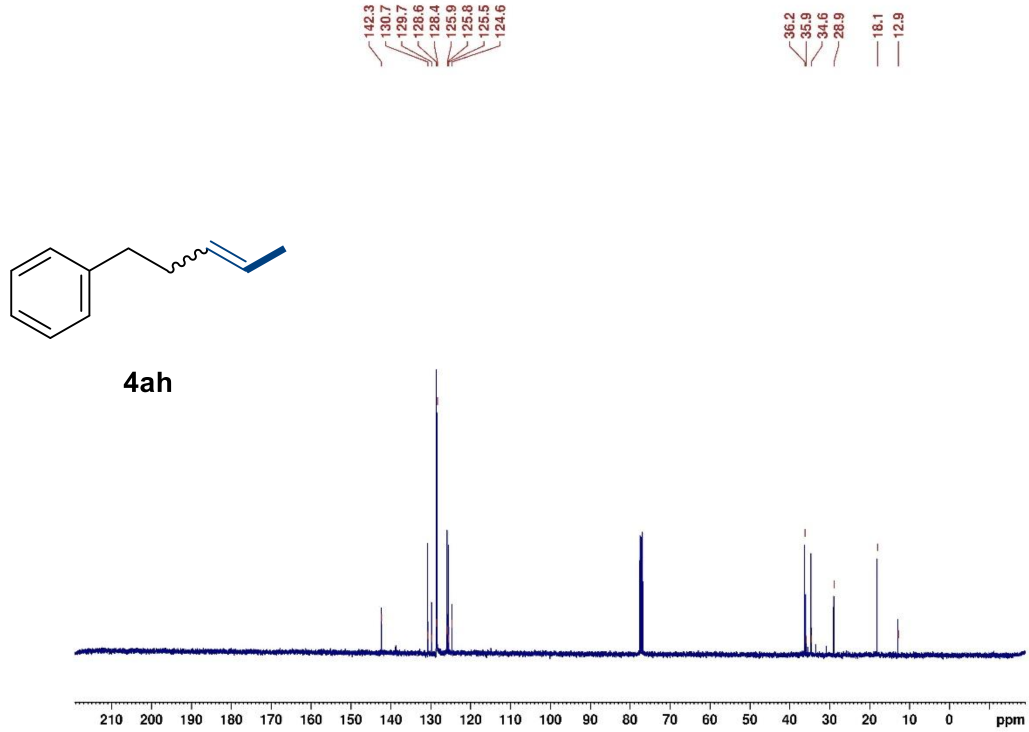

^{1}H NMR spectrum of hex-4-en-1-ylbenzene **4ai** (400 MHz, $CDCl_3$, RT)

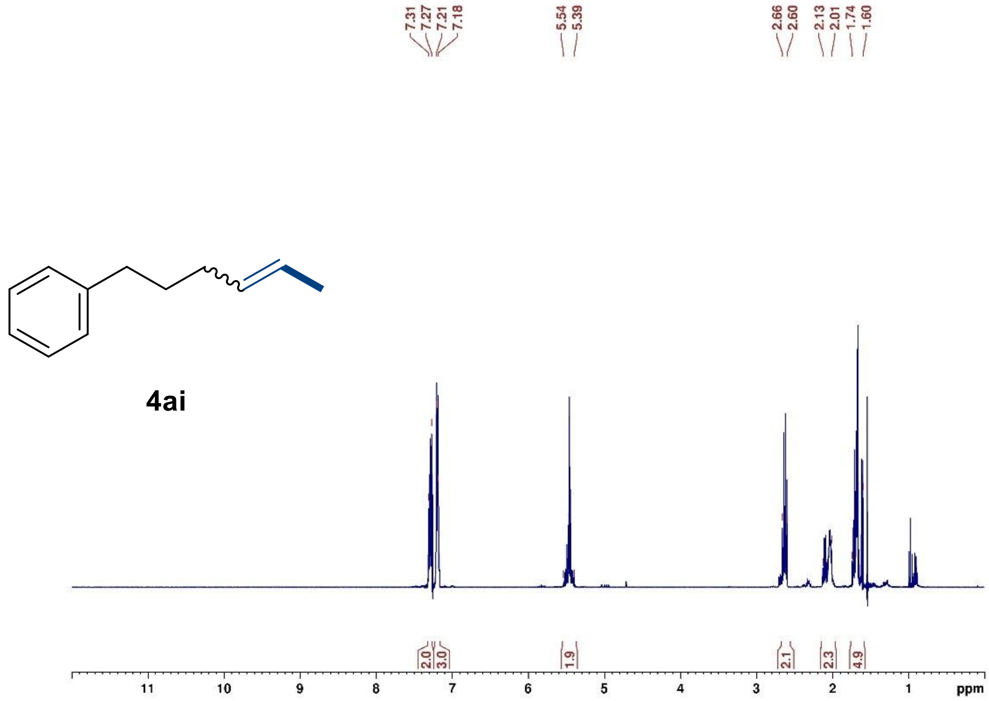

^{13}C NMR spectrum of hex-4-en-1-ylbenzene **4ai** (100 MHz, $CDCl_3$, RT)

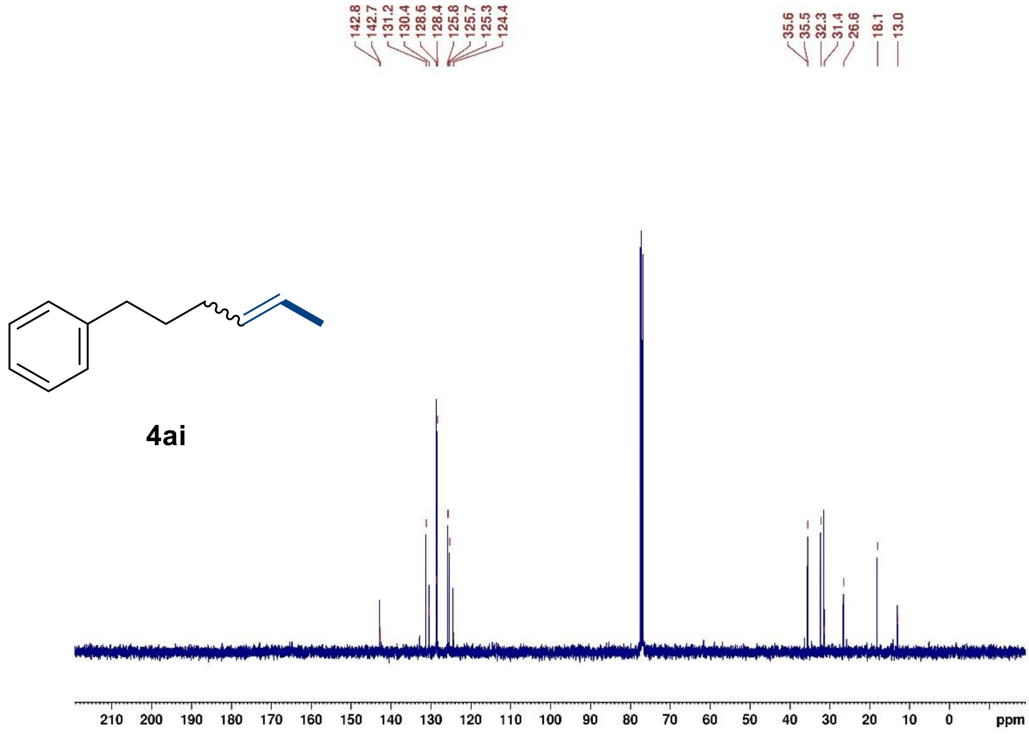

Supplement: Supplementary file 1 — Supporting Information [file ANIE-64-e202519729-s001.pdf]
