# Supplementary material for: Iron‐Catalyzed Tunable Double Bond Migration and Geometrical Isomerization in Olefins via a Spin‐Accelerated Alkyl Mechanism
Source: Angew Chem Int Ed Engl. 2025 Oct 16;64(50):e202519729. doi: 10.1002/anie.202519729 (PMC12684360; doi:10.1002/anie.202519729)
Supplement: Supplementary file 2 — Supporting Information [file ANIE-64-e202519729-s003.pdf]

Cartesian coordinates & computed energies

a. Activation of 2a

2a(V)
SCF (BP86/SDD/6-31G**) Energy 333 K = -1886.93587224
Thermal correction to Gibbs Free Energy= 0.641410
Lowest Frequency = 14.2546 cm^{-1}
Second Frequency = 17.4129 cm^{-1}
SCF (B3PW91-D3,C6H6/tzvp) Energy 333 K= -3026.66207953

6	-0.524155000	2.315451000	0.889342000
1	-1.617656000	2.333715000	1.087429000
1	-0.015992000	2.428058000	1.870707000
6	-1.294700000	-2.251559000	-0.101599000
1	-2.157748000	-2.934102000	-0.184592000
6	-0.004595000	-2.871786000	-0.153970000
6	1.266482000	-2.169571000	-0.080519000
6	2.454598000	-2.972414000	-0.130156000
1	3.420562000	-2.464476000	-0.068175000
6	2.404931000	-4.355273000	-0.251021000
1	3.342178000	-4.922082000	-0.282273000
6	1.165560000	-5.037476000	-0.332940000
1	1.132293000	-6.126297000	-0.430459000
6	-0.003482000	-4.294381000	-0.283369000
1	-0.975235000	-4.799720000	-0.340902000
6	-2.943006000	-0.556407000	0.064483000
6	-3.644620000	-0.389786000	-1.161610000
6	-4.990074000	0.023572000	-1.099956000
1	-5.552175000	0.148655000	-2.031857000
6	-5.619627000	0.277737000	0.123933000
1	-6.666536000	0.598183000	0.147365000
6	-4.905313000	0.123564000	1.319413000
1	-5.403049000	0.324608000	2.273585000
6	-3.559903000	-0.288776000	1.319213000
6	-2.994387000	-0.666395000	-2.519049000
1	-1.909772000	-0.790804000	-2.352450000
6	-3.175824000	0.504589000	-3.509683000
1	-2.636025000	0.294311000	-4.449409000
1	-2.785757000	1.448443000	-3.094785000
1	-4.237123000	0.661793000	-3.770061000
6	-3.526128000	-1.984096000	-3.130693000
1	-3.345833000	-2.843887000	-2.463628000
1	-3.034099000	-2.192080000	-4.096960000
1	-4.614234000	-1.924353000	-3.310155000
6	-2.809616000	-0.504516000	2.634582000
1	-1.727996000	-0.453976000	2.404497000
6	-3.096543000	-1.915275000	3.201977000
1	-4.170582000	-2.032393000	3.431542000
1	-2.527923000	-2.085062000	4.133196000
1	-2.818630000	-2.704883000	2.483919000
6	-3.103237000	0.580343000	3.690501000
1	-2.915765000	1.591436000	3.292873000
1	-2.456282000	0.433147000	4.572385000
1	-4.147836000	0.540803000	4.046250000
6	2.614307000	-0.161630000	0.051613000
6	3.226019000	0.153408000	1.301773000
6	4.467359000	0.817895000	1.292659000
1	4.949704000	1.060448000	2.246127000
6	5.098657000	1.168549000	0.093088000
1	6.064624000	1.684315000	0.109163000
6	4.487361000	0.855648000	-1.127745000
1	4.984299000	1.129396000	-2.065373000
6	3.246375000	0.192675000	-1.178600000
6	2.591526000	-0.248515000	2.634399000
1	1.566049000	-0.599595000	2.416601000
6	3.356666000	-1.427117000	3.281673000
1	3.387455000	-2.301610000	2.611215000
1	2.871022000	-1.735194000	4.224497000
1	4.397589000	-1.140645000	3.516013000
6	2.480338000	0.937343000	3.616939000
1	3.473568000	1.311152000	3.922067000
1	1.951349000	0.626183000	4.534753000
1	1.927341000	1.779829000	3.169186000
6	2.627590000	-0.147895000	-2.536097000
1	1.629002000	-0.578330000	-2.344322000
6	3.460884000	-1.214890000	-3.283967000

1	4.477723000	-0.843444000	-3.503855000
1	2.983376000	-1.474928000	-4.245158000
1	3.556083000	-2.138265000	-2.689770000
6	2.435972000	1.106642000	-3.417046000
1	1.826839000	1.871172000	-2.906643000
1	1.932073000	0.839466000	-4.362572000
1	3.402890000	1.571603000	-3.678554000
6	1.811241000	3.994862000	-0.305047000
1	2.247550000	4.188765000	0.690697000
1	2.086538000	4.841942000	-0.958088000
1	2.296923000	3.087268000	-0.702489000
6	-0.822615000	3.599155000	-1.927900000
1	-0.431007000	2.702789000	-2.441010000
1	-0.602855000	4.471204000	-2.568850000
1	-1.921150000	3.497732000	-1.870078000
6	-0.788367000	5.411629000	0.556079000
1	-1.888889000	5.365232000	0.633218000
1	-0.532816000	6.289026000	-0.064841000
1	-0.391719000	5.592926000	1.570430000
26	-0.156350000	0.456623000	0.257728000
7	-1.558450000	-0.960036000	0.044578000
7	1.326839000	-0.802878000	0.030439000
14	-0.076715000	3.800636000	-0.184333000

2a(I)
SCF (BP86/SDD/6-31G**) Energy 333 K = -1886.90241162
Thermal correction to Gibbs Free Energy= 0.646795
Lowest Frequency = 14.3921 cm^{-1}
Second Frequency = 19.4305 cm^{-1}
SCF (B3PW91-D3,C6H6/tzvp) Energy 333 K= -3026.60161453

6	0.529959000	1.746572000	-1.150076000
1	1.521450000	1.587791000	-1.614819000
1	-0.202390000	1.818567000	-1.974664000
6	1.072226000	-2.272268000	-0.376413000
1	1.929442000	-2.955983000	-0.442409000
6	-0.230859000	-2.818190000	-0.549575000
6	-1.435382000	-2.035374000	-0.375941000
6	-2.692104000	-2.711825000	-0.438981000
1	-3.604459000	-2.131751000	-0.273506000
6	-2.767791000	-4.074329000	-0.708752000
1	-3.749287000	-4.557785000	-0.759802000
6	-1.590904000	-4.836665000	-0.920968000
1	-1.656611000	-5.906849000	-1.139201000
6	-0.355477000	-4.212286000	-0.833286000
1	0.565033000	-4.791985000	-0.971432000
6	2.724101000	-0.608584000	0.107677000
6	3.219355000	-0.425185000	1.432117000
6	4.563175000	-0.033948000	1.584700000
1	4.966680000	0.112288000	2.591245000
6	5.394511000	0.165013000	0.475116000
1	6.437056000	0.468579000	0.618607000
6	4.892048000	-0.030312000	-0.816605000
1	5.551076000	0.117868000	-1.678615000
6	3.554901000	-0.415487000	-1.033898000
6	2.349100000	-0.712276000	2.657709000
1	1.301574000	-0.493900000	2.365090000
6	2.682940000	0.171701000	3.875133000
1	1.932078000	0.014830000	4.668212000
1	2.686382000	1.242824000	3.613090000
1	3.667002000	-0.076265000	4.311535000
6	2.426626000	-2.210285000	3.039077000
1	2.104465000	-2.856775000	2.206525000
1	1.777924000	-2.423716000	3.906862000
1	3.460604000	-2.490625000	3.308896000
6	3.059591000	-0.660595000	-2.461912000
1	1.956856000	-0.697237000	-2.430781000
6	3.567978000	-2.022834000	-2.993495000
1	4.671637000	-2.042110000	-3.032453000
1	3.188481000	-2.206191000	-4.013989000
1	3.241147000	-2.859929000	-2.355021000
6	3.462467000	0.465377000	-3.440204000
1	3.137863000	1.456355000	-3.082049000
1	2.997477000	0.291036000	-4.425825000
1	4.554715000	0.504088000	-3.597497000
6	-2.530264000	0.102821000	0.103987000
6	-3.194356000	0.695985000	-1.015868000
6	-4.329979000	1.490002000	-0.769986000
1	-4.853121000	1.949356000	-1.615461000
6	-4.806621000	1.703701000	0.530046000

1	-5.690350000	2.329605000	0.694512000
6	-4.154084000	1.110054000	1.616588000
1	-4.537348000	1.270943000	2.630420000
6	-3.018588000	0.297268000	1.432980000
6	-2.736689000	0.457705000	-2.456411000
1	-1.716048000	0.039456000	-2.410241000
6	-3.643371000	-0.575060000	-3.169748000
1	-3.641356000	-1.545919000	-2.649837000
1	-3.292879000	-0.742129000	-4.203566000
1	-4.685952000	-0.212760000	-3.220929000
6	-2.683173000	1.761179000	-3.285146000
1	-3.692930000	2.165591000	-3.476584000
1	-2.220818000	1.566019000	-4.268212000
1	-2.098139000	2.547928000	-2.780833000
6	-2.379542000	-0.379876000	2.645836000
1	-1.487405000	-0.921908000	2.287341000
6	-3.339965000	-1.415559000	3.275272000
1	-4.256105000	-0.934561000	3.662008000
1	-2.849911000	-1.931120000	4.119819000
1	-3.641363000	-2.178785000	2.538975000
6	-1.905521000	0.648119000	3.695980000
1	-1.175569000	1.352036000	3.260412000
1	-1.421853000	0.135104000	4.545656000
1	-2.746302000	1.239130000	4.100153000
6	-1.173209000	3.901399000	0.392550000
1	-1.862898000	4.002268000	-0.463158000
1	-1.149423000	4.871143000	0.920912000
1	-1.613857000	3.156202000	1.077630000
6	1.707830000	3.274487000	1.343029000
1	1.287829000	2.586280000	2.098597000
1	1.834756000	4.260545000	1.824865000
1	2.709230000	2.898012000	1.071597000
6	1.253473000	4.756679000	-1.329466000
1	2.279463000	4.521101000	-1.660974000
1	1.282538000	5.730687000	-0.808732000
1	0.628080000	4.874170000	-2.231078000
26	0.126936000	0.319127000	0.092666000
7	1.337404000	-0.990265000	-0.076050000
7	-1.342625000	-0.688263000	-0.097228000
14	0.568381000	3.394151000	-0.180292000

2a(III)
SCF (BP86/SDD/6-31G**) Energy 333 K = -1886.91820595
Thermal correction to Gibbs Free Energy= 0.642898
Lowest Frequency = 13.2160 cm^{-1}
Second Frequency = 19.9447 cm^{-1}
SCF (B3PW91-D3,C6H6/tzvp) Energy 333 K= -3026.63177109

6	1.041482000	2.018758000	-0.806564000
1	2.143005000	1.952005000	-0.744236000
1	0.796755000	2.053159000	-1.894518000
6	1.077603000	-2.283446000	0.139256000
1	1.946016000	-2.955340000	0.207122000
6	-0.204165000	-2.899578000	0.203980000
6	-1.441964000	-2.151078000	0.138438000
6	-2.671359000	-2.881945000	0.215929000
1	-3.610896000	-2.324188000	0.159455000
6	-2.678226000	-4.264270000	0.349705000
1	-3.636878000	-4.792328000	0.401134000
6	-1.465588000	-5.000519000	0.417829000
1	-1.484304000	-6.088878000	0.525194000
6	-0.260286000	-4.319827000	0.344710000
1	0.686958000	-4.870207000	0.394510000
6	2.777136000	-0.650947000	0.003022000
6	3.432878000	-0.423106000	1.242625000
6	4.811615000	-0.136575000	1.212757000
1	5.342224000	0.034776000	2.155216000
6	5.514793000	-0.068612000	0.003763000
1	6.587203000	0.153769000	0.004318000
6	4.843043000	-0.282933000	-1.206658000
1	5.398223000	-0.225278000	-2.148763000
6	3.466151000	-0.574461000	-1.237173000
6	2.691678000	-0.519502000	2.577268000
1	1.611099000	-0.446206000	2.357180000
6	3.044131000	0.630615000	3.543966000
1	2.416016000	0.567805000	4.449495000
1	2.875698000	1.615142000	3.078662000
1	4.096169000	0.584359000	3.876125000
6	2.944512000	-1.888816000	3.252363000
1	2.625717000	-2.726060000	2.609418000

1	2.390073000	-1.961782000	4.204523000
1	4.018373000	-2.024437000	3.472650000
6	2.755517000	-0.831356000	-2.566125000
1	1.671072000	-0.723670000	-2.379489000
6	3.009565000	-2.275744000	-3.058656000
1	4.085917000	-2.445568000	-3.239872000
1	2.471426000	-2.465305000	-4.004125000
1	2.671910000	-3.022080000	-2.320132000
6	3.135692000	0.192574000	-3.656255000
1	2.969729000	1.225747000	-3.309672000
1	2.521928000	0.028845000	-4.558987000
1	4.192743000	0.099634000	-3.962074000
6	-2.573360000	0.001837000	-0.048265000
6	-3.138568000	0.348936000	-1.316169000
6	-4.277339000	1.176254000	-1.336595000
1	-4.724112000	1.446683000	-2.299731000
6	-4.853091000	1.651927000	-0.152331000
1	-5.740340000	2.292785000	-0.191980000
6	-4.291450000	1.306533000	1.084893000
1	-4.748843000	1.681252000	2.007287000
6	-3.151487000	0.487230000	1.168350000
6	-2.570945000	-0.194509000	-2.627889000
1	-1.622454000	-0.707176000	-2.386889000
6	-3.523987000	-1.241320000	-3.251407000
1	-3.709367000	-2.077094000	-2.556762000
1	-3.088772000	-1.656796000	-4.177231000
1	-4.498399000	-0.789589000	-3.509835000
6	-2.249163000	0.929669000	-3.636453000
1	-3.157425000	1.475647000	-3.946949000
1	-1.791503000	0.506607000	-4.547649000
1	-1.545038000	1.662614000	-3.207566000
6	-2.580654000	0.113492000	2.536574000
1	-1.629411000	-0.418608000	2.360396000
6	-3.526181000	-0.849726000	3.292396000
1	-4.503030000	-0.373793000	3.491478000
1	-3.087347000	-1.136919000	4.264048000
1	-3.705929000	-1.770860000	2.714509000
6	-2.268233000	1.357872000	3.396595000

1	-1.581896000	2.046735000	2.876381000
1	-1.796257000	1.056104000	4.347813000
1	-3.183421000	1.922381000	3.647963000
6	-1.375295000	3.916401000	-0.121319000
1	-1.729270000	3.868530000	-1.166333000
1	-1.659084000	4.906504000	0.278140000
1	-1.934798000	3.154172000	0.448403000
6	1.076051000	3.773422000	1.786554000
1	0.634017000	2.970612000	2.401779000
1	0.794262000	4.740148000	2.240079000
1	2.174260000	3.680408000	1.858154000
6	1.325383000	5.110081000	-0.973413000
1	2.426406000	5.033630000	-0.946685000
1	1.044165000	6.084036000	-0.533715000
1	1.018109000	5.119647000	-2.033811000
26	0.096356000	0.368642000	-0.226928000
7	1.364704000	-0.982136000	0.008242000
7	-1.388282000	-0.798196000	-0.001661000
14	0.510266000	3.660863000	-0.028897000

I (V)
SCF (BP86/SDD/6-31G**) Energy 333 K = -2439.47830444
Thermal correction to Gibbs Free Energy= 0.983548
Lowest Frequency = 5.3485 cm^{-1}
Second Frequency = 15.3224 cm^{-1}
SCF (B3PW91-D3,C6H6/tzvp) Energy 333 K= -3579.28454454

26	-0.402009000	-0.015418000	0.406576000
6	0.574231000	0.132041000	2.227211000
6	0.963858000	-0.863811000	5.170898000
1	0.488011000	-1.003146000	6.158420000
7	-0.988464000	-1.966246000	-0.092228000
6	-2.267073000	-2.295429000	-0.046565000
6	-3.385440000	-1.422414000	0.181217000
6	-3.351510000	0.028636000	0.057141000
7	-2.189722000	0.718123000	-0.192356000
6	-2.322799000	2.024821000	-0.783768000
6	-2.131059000	3.211697000	-0.016739000
6	-2.264383000	4.464585000	-0.648458000
6	-2.580285000	4.572731000	-2.005791000
6	-2.761367000	3.405316000	-2.755450000
6	-2.634525000	2.129034000	-2.176686000

6	-1.843617000	3.183948000	1.483062000
6	-0.579471000	3.996953000	1.838222000
6	-2.841702000	0.903802000	-3.068308000
6	-1.908446000	0.936700000	-4.298849000
6	-0.063311000	-3.009268000	-0.474470000
6	0.509297000	-3.862420000	0.512086000
6	1.430055000	-4.845102000	0.095979000
6	1.784354000	-4.992845000	-1.249416000
6	1.207798000	-4.151565000	-2.208750000
6	0.282679000	-3.151795000	-1.850940000
6	0.097013000	-3.782908000	1.981217000
6	-1.052422000	-4.775014000	2.284704000
6	-0.386622000	-2.311488000	-2.938859000
6	0.566472000	-1.923942000	-4.087502000
6	-1.625266000	-3.051493000	-3.501142000
6	1.266861000	-4.030230000	2.955336000
6	-3.064631000	3.692098000	2.286128000
6	-4.317696000	0.764859000	-3.508151000
1	1.380935000	-0.633305000	2.191680000
1	1.076746000	1.126863000	2.246519000
1	1.471582000	-4.279861000	-3.263370000
1	2.496688000	-5.768274000	-1.551126000
1	1.869390000	-5.513950000	0.842870000
1	-3.007634000	3.482853000	-3.820621000
1	-2.686549000	5.556747000	-2.475154000
1	-2.131213000	5.375243000	-0.053317000
1	-2.581020000	0.011565000	-2.474121000
1	-0.736765000	-1.376502000	-2.466085000
1	0.052196000	-1.247524000	-4.789672000
1	1.461050000	-1.400992000	-3.711629000
1	0.898218000	-2.802503000	-4.668784000
1	-2.127930000	-2.440436000	-4.271317000
1	-1.329310000	-4.009441000	-3.965034000
1	-2.361994000	-3.273993000	-2.711618000
1	-2.033726000	0.018845000	-4.900588000
1	-2.134734000	1.791849000	-4.960055000
1	-0.850713000	1.015036000	-3.997221000
1	-4.456883000	-0.142968000	-4.121772000
1	-4.989639000	0.696686000	-2.637359000
1	-4.633367000	1.632175000	-4.115678000
1	-0.280926000	-2.760864000	2.159643000
1	-1.361466000	-4.702321000	3.342511000
1	-1.938595000	-4.580427000	1.658979000
1	-0.729301000	-5.814089000	2.094473000
1	0.932390000	-3.868765000	3.993841000
1	1.641462000	-5.067536000	2.898971000
1	2.113872000	-3.352111000	2.757476000
1	-1.658301000	2.132231000	1.767066000
1	-2.864020000	3.664924000	3.371985000
1	-3.308577000	4.735654000	2.017819000
1	-3.957308000	3.075282000	2.090303000
1	-0.360361000	3.924734000	2.916545000
1	0.296504000	3.622192000	1.281384000
1	-0.699930000	5.067222000	1.594026000
1	1.310199000	-1.851451000	4.823957000
1	1.859739000	-0.234579000	5.320488000
7	2.769657000	1.110720000	-0.219409000
5	1.403029000	0.955049000	-1.089939000
1	2.447383000	0.882705000	0.739494000
1	1.011095000	-0.221713000	-0.926541000
6	-0.637872000	1.663812000	4.705967000
1	-1.435300000	2.193855000	4.161101000
1	-0.983870000	1.528866000	5.746514000
1	0.250466000	2.319007000	4.735131000
6	-1.872044000	-1.016003000	3.925690000
1	-2.329103000	-1.017937000	4.931751000
1	-1.738795000	-2.067733000	3.621674000
14	-0.238323000	-0.034426000	3.935156000
1	-2.596654000	-0.563323000	3.226675000
1	0.630001000	1.797310000	-0.649080000
1	1.605591000	1.124688000	-2.273151000
6	3.220711000	2.567754000	-0.127251000
6	3.886762000	3.080732000	-1.413319000
6	4.198739000	4.587282000	-1.284019000
6	5.068006000	4.882745000	-0.047134000
6	4.414176000	4.336180000	1.236095000

6	4.099359000	2.829719000	1.111323000
1	2.271592000	3.118988000	0.004689000
1	3.226895000	2.892358000	-2.276732000
1	4.832954000	2.533366000	-1.589911000
1	4.698391000	4.941363000	-2.203099000
1	3.247478000	5.149345000	-1.208102000
1	5.244366000	5.969218000	0.047723000
1	6.063159000	4.412927000	-0.178905000
1	5.065966000	4.508682000	2.110830000
1	3.473396000	4.886770000	1.431577000
1	3.585236000	2.470423000	2.023134000
1	5.051509000	2.269386000	1.034425000
6	3.902426000	0.116497000	-0.506434000
6	4.001536000	-0.304756000	-1.982050000
6	5.202588000	-1.253476000	-2.183068000
6	5.096910000	-2.494645000	-1.278242000
6	4.946602000	-2.088598000	0.199171000
6	3.763491000	-1.119714000	0.402469000
1	4.841981000	0.632331000	-0.229985000
1	4.088570000	0.576102000	-2.639054000
1	3.070117000	-0.824095000	-2.267575000
1	5.259460000	-1.551408000	-3.245154000
1	6.144507000	-0.712276000	-1.960021000
1	5.982404000	-3.142963000	-1.408178000
1	4.214902000	-3.092223000	-1.579100000
1	4.803103000	-2.980326000	0.834241000
1	5.880570000	-1.601536000	0.544944000
1	3.712528000	-0.804996000	1.462300000
1	2.813801000	-1.638081000	0.170545000
1	-2.528694000	-3.354942000	-0.218685000
6	-4.614280000	0.705983000	0.171351000
1	-4.620407000	1.793470000	0.059943000
6	-5.798887000	0.032027000	0.437472000
6	-4.632996000	-2.073407000	0.423558000
1	-6.726928000	0.605860000	0.541633000
1	-4.628368000	-3.168088000	0.494899000
6	-5.823652000	-1.378679000	0.571350000
1	-6.760121000	-1.906713000	0.772392000

I (I)
SCF (BP86/SDD/6-31G**) Energy 333 K = -2439.45947410
Thermal correction to Gibbs Free Energy= 0.995615
Lowest Frequency = 13.8839 cm^{-1}
Second Frequency = 18.6362 cm^{-1}
SCF (B3PW91-D3,C6H6/tzvp) Energy 333 K= -3579.23242990

26	-0.333107000	-0.130536000	-0.002364000
6	0.323679000	-0.079749000	1.935660000
6	0.590526000	-1.575880000	4.632417000
1	0.083788000	-1.931085000	5.547951000
7	-0.945493000	-1.884768000	-0.260331000
6	-2.227817000	-2.275171000	-0.264916000
6	-3.355264000	-1.439500000	-0.021991000
6	-3.270191000	0.006293000	0.004971000
7	-2.050664000	0.647023000	-0.108821000
6	-2.110596000	2.028693000	-0.552887000
6	-1.954754000	3.119918000	0.351308000
6	-2.009514000	4.438663000	-0.145103000
6	-2.230211000	4.706378000	-1.498429000
6	-2.404705000	3.633863000	-2.379168000
6	-2.350398000	2.297955000	-1.939354000
6	-1.801759000	2.929147000	1.856978000
6	-0.574027000	3.684065000	2.412005000
6	-2.570580000	1.196767000	-2.977609000
6	-1.602449000	1.345870000	-4.172045000
6	-0.003031000	-2.904955000	-0.714002000
6	0.471286000	-3.917801000	0.169651000
6	1.379185000	-4.875470000	-0.330564000
6	1.811920000	-4.857172000	-1.658591000
6	1.318713000	-3.874352000	-2.522914000
6	0.408155000	-2.895149000	-2.082184000
6	-0.005689000	-4.050059000	1.616525000
6	-1.210822000	-5.017728000	1.722485000
6	-0.172185000	-1.916419000	-3.102990000
6	0.845047000	-1.470490000	-4.172835000
6	-1.423398000	-2.534248000	-3.773564000
6	1.105244000	-4.532633000	2.573904000
6	-3.087504000	3.377719000	2.593091000
6	-4.036614000	1.169224000	-3.470156000
1	1.228482000	-0.724984000	1.954415000

1	0.679707000	0.962396000	2.110523000
1	1.635059000	-3.873465000	-3.570497000
1	2.515903000	-5.613499000	-2.022500000
1	1.748109000	-5.657988000	0.339155000
1	-2.591850000	3.835187000	-3.439900000
1	-2.277812000	5.738727000	-1.861848000
1	-1.898440000	5.273322000	0.556386000
1	-2.360945000	0.229138000	-2.491609000
1	-0.491304000	-1.012569000	-2.550717000
1	0.403715000	-0.682846000	-4.804920000
1	1.762058000	-1.063818000	-3.715132000
1	1.132996000	-2.298089000	-4.845410000
1	-1.861878000	-1.833451000	-4.505439000
1	-1.157723000	-3.463698000	-4.308767000
1	-2.199744000	-2.779684000	-3.030695000
1	-1.734906000	0.509963000	-4.881316000
1	-1.786670000	2.278694000	-4.733618000
1	-0.551817000	1.355871000	-3.837061000
1	-4.186485000	0.347072000	-4.192334000
1	-4.736881000	1.021460000	-2.632486000
1	-4.302063000	2.114603000	-3.977040000
1	-0.337476000	-3.050722000	1.946559000
1	-1.542677000	-5.108029000	2.771822000
1	-2.070678000	-4.675695000	1.125768000
1	-0.930432000	-6.024918000	1.365573000
1	0.743334000	-4.509245000	3.615136000
1	1.401657000	-5.576119000	2.367540000
1	2.010410000	-3.905637000	2.507622000
1	-1.652714000	1.851957000	2.036924000
1	-2.985122000	3.248257000	3.685238000
1	-3.300752000	4.444904000	2.403115000
1	-3.961762000	2.793312000	2.262777000
1	-0.460354000	3.501293000	3.493142000
1	0.353160000	3.357195000	1.909563000
1	-0.665664000	4.775363000	2.268839000
1	1.004617000	-2.453262000	4.114132000
1	1.443344000	-0.949727000	4.952764000
7	2.714061000	1.104474000	0.123051000

5	1.406688000	0.648675000	-0.753600000
1	2.388060000	0.911453000	1.088904000
1	1.264678000	-0.589784000	-0.474601000
6	-0.954636000	0.961455000	4.684424000
1	-1.772779000	1.603962000	4.324865000
1	-1.249074000	0.584613000	5.681022000
1	-0.057909000	1.589912000	4.825741000
6	-2.265909000	-1.441560000	3.373322000
1	-2.664802000	-1.692097000	4.372910000
1	-2.203624000	-2.373314000	2.789664000
14	-0.601867000	-0.538592000	3.551619000
1	-3.003647000	-0.795916000	2.867879000
1	0.469834000	1.388137000	-0.296355000
1	1.605795000	0.851783000	-1.932396000
6	2.933506000	2.614207000	0.080576000
6	3.599373000	3.099357000	-1.217058000
6	3.685298000	4.640998000	-1.224329000
6	4.428035000	5.173549000	0.015478000
6	3.780176000	4.659669000	1.314948000
6	3.684941000	3.118907000	1.327622000
1	1.906744000	3.026838000	0.109836000
1	3.029507000	2.738861000	-2.089768000
1	4.622972000	2.683879000	-1.292141000
1	4.184255000	4.977691000	-2.150226000
1	2.661032000	5.061140000	-1.249260000
1	4.443607000	6.278106000	0.008402000
1	5.485572000	4.843868000	-0.021772000
1	4.347368000	5.004137000	2.197736000
1	2.761743000	5.083744000	1.410248000
1	3.166910000	2.777397000	2.244052000
1	4.707342000	2.693216000	1.354951000
6	3.980003000	0.260077000	-0.056721000
6	4.169608000	-0.271801000	-1.489345000
6	5.485300000	-1.072872000	-1.589438000
6	5.514350000	-2.234496000	-0.579366000
6	5.285384000	-1.720988000	0.854070000
6	3.983745000	-0.899898000	0.958088000
1	4.832835000	0.923453000	0.184686000

1	4.163142000	0.554501000	-2.219080000
1	3.320450000	-0.930808000	-1.744937000
1	5.604890000	-1.451513000	-2.619931000
1	6.345868000	-0.399094000	-1.402010000
1	6.475086000	-2.776580000	-0.642334000
1	4.719691000	-2.960865000	-0.837878000
1	5.248210000	-2.561582000	1.569221000
1	6.142138000	-1.087082000	1.158920000
1	3.866108000	-0.505093000	1.985136000
1	3.113993000	-1.554487000	0.758979000
1	-2.436068000	-3.325468000	-0.515777000
6	-4.502080000	0.728497000	0.120379000
1	-4.464206000	1.821016000	0.115849000
6	-5.726206000	0.082086000	0.256580000
6	-4.634032000	-2.064535000	0.094560000
1	-6.637720000	0.678856000	0.372932000
1	-4.671769000	-3.160332000	0.063460000
6	-5.802574000	-1.333583000	0.245410000
1	-6.767449000	-1.840077000	0.345559000

I (III)
SCF (BP86/SDD/6-31G**) Energy 333 K = -2439.48053645
Thermal correction to Gibbs Free Energy= 0.992971
Lowest Frequency = 10.4842 cm^{-1}
Second Frequency = 18.0596 cm^{-1}
SCF (B3PW91-D3,C6H6/tzvp) Energy 333 K= -3579.27498610

26	-0.495077000	0.017989000	0.297637000
6	-0.174111000	-1.283284000	1.841299000
6	-0.188025000	-1.760629000	4.971071000
1	0.014927000	-1.277553000	5.944396000
7	-2.333465000	-0.526365000	0.036528000
6	-3.355190000	0.323038000	-0.023772000
6	-3.308068000	1.746179000	-0.076906000
6	-2.091544000	2.506235000	-0.299308000
7	-0.849222000	1.916846000	-0.285086000
6	0.208489000	2.749218000	-0.814321000
6	1.058092000	3.499219000	0.054590000
6	2.068812000	4.300026000	-0.513834000
6	2.256073000	4.377655000	-1.899069000
6	1.419986000	3.639475000	-2.742059000
6	0.393622000	2.823853000	-2.227717000
6	0.878428000	3.484810000	1.574934000
6	2.213478000	3.608053000	2.339940000
6	-0.503695000	2.070644000	-3.212007000
6	0.314048000	1.180644000	-4.173605000
6	-2.710738000	-1.919066000	-0.152423000
6	-3.359416000	-2.655261000	0.880405000
6	-3.687317000	-4.004883000	0.633101000
6	-3.404579000	-4.616502000	-0.590993000
6	-2.811163000	-3.865971000	-1.613024000
6	-2.467131000	-2.514090000	-1.425482000
6	-3.769090000	-2.035102000	2.218820000
6	-5.282226000	-1.705391000	2.242157000
6	-1.969647000	-1.686326000	-2.611082000
6	-1.075551000	-2.474975000	-3.586961000
6	-3.171546000	-1.071594000	-3.369732000
6	-3.428517000	-2.941242000	3.422309000
6	-0.084222000	4.596554000	2.059734000
6	-1.404167000	3.042742000	-4.010022000
1	-1.005327000	-2.012793000	1.831060000
1	0.739002000	-1.920280000	1.757255000
1	-2.629088000	-4.333331000	-2.585950000
1	-3.664832000	-5.667483000	-0.756754000
1	-4.181327000	-4.583480000	1.420658000
1	1.558183000	3.700585000	-3.827851000
1	3.045198000	5.013082000	-2.316014000
1	2.719404000	4.884851000	0.144447000
1	-1.161235000	1.409877000	-2.621803000
1	-1.368913000	-0.852814000	-2.205029000
1	-0.640072000	-1.791762000	-4.335332000
1	-0.247498000	-2.978126000	-3.059817000
1	-1.640700000	-3.245115000	-4.141693000
1	-2.822056000	-0.448427000	-4.211841000
1	-3.822052000	-1.865750000	-3.778182000
1	-3.784587000	-0.435736000	-2.710471000
1	-0.360766000	0.607409000	-4.834451000
1	0.974089000	1.780965000	-4.824960000
1	0.945768000	0.469916000	-3.615941000

1	-2.074458000	2.484607000	-4.688055000
1	-2.028268000	3.652835000	-3.337285000
1	-0.796803000	3.728416000	-4.628212000
1	-3.211130000	-1.090050000	2.335268000
1	-5.563561000	-1.247094000	3.206653000
1	-5.570191000	-1.006979000	1.439692000
1	-5.882240000	-2.623923000	2.114776000
1	-3.605373000	-2.399523000	4.367606000
1	-4.062323000	-3.845189000	3.445801000
1	-2.376148000	-3.266148000	3.401254000
1	0.419612000	2.512018000	1.831481000
1	-0.197398000	4.555471000	3.157871000
1	0.313699000	5.593945000	1.797627000
1	-1.084117000	4.500930000	1.609791000
1	2.053506000	3.430791000	3.416820000
1	2.966425000	2.885042000	1.980361000
1	2.649031000	4.618589000	2.242608000
1	-1.184662000	-2.226344000	5.033182000
1	0.551349000	-2.571570000	4.843984000
7	2.645601000	-1.196807000	0.028719000
5	1.567325000	-0.121113000	-0.532985000
1	2.115504000	-1.666035000	0.785495000
1	0.626260000	-0.738299000	-1.067030000
6	1.655769000	0.317616000	3.804123000
1	1.905562000	1.031306000	3.003761000
1	1.698973000	0.863442000	4.763410000
1	2.444575000	-0.455894000	3.837592000
6	-1.401813000	0.841267000	3.837988000
1	-1.265465000	1.338547000	4.814899000
1	-2.412296000	0.396630000	3.833146000
14	-0.059251000	-0.479538000	3.553025000
1	-1.380799000	1.618028000	3.054511000
1	1.183193000	0.519954000	0.492398000
1	2.104842000	0.646022000	-1.294468000
6	3.812145000	-0.514738000	0.744380000
6	4.836820000	0.116193000	-0.212003000
6	5.906678000	0.884218000	0.593785000
6	6.597140000	-0.020221000	1.631678000

6	5.566730000	-0.687415000	2.562220000
6	4.494599000	-1.453749000	1.758137000
1	3.325726000	0.300261000	1.310838000
1	4.326580000	0.791876000	-0.917948000
1	5.335960000	-0.675668000	-0.803637000
1	6.649332000	1.317226000	-0.099476000
1	5.426415000	1.738102000	1.110251000
1	7.325534000	0.561624000	2.224463000
1	7.176003000	-0.805146000	1.104995000
1	6.065262000	-1.376049000	3.267276000
1	5.068165000	0.086805000	3.177115000
1	3.740515000	-1.884907000	2.443619000
1	4.976531000	-2.301354000	1.232723000
6	3.064272000	-2.318201000	-0.924156000
6	3.137003000	-1.888711000	-2.399629000
6	3.639263000	-3.065798000	-3.263129000
6	2.743595000	-4.308576000	-3.105155000
6	2.602515000	-4.711980000	-1.625169000
6	2.130773000	-3.531193000	-0.750961000
1	4.078178000	-2.628649000	-0.606935000
1	3.793667000	-1.012188000	-2.522463000
1	2.131200000	-1.580878000	-2.736602000
1	3.683473000	-2.753060000	-4.321333000
1	4.677681000	-3.321010000	-2.969738000
1	3.148376000	-5.152335000	-3.692628000
1	1.741700000	-4.087661000	-3.521036000
1	1.899029000	-5.556211000	-1.516623000
1	3.581993000	-5.070106000	-1.249728000
1	2.103061000	-3.839280000	0.311885000
1	1.099423000	-3.238686000	-1.024418000
1	-4.360451000	-0.120092000	-0.101111000
6	-2.274386000	3.911397000	-0.561255000
1	-1.391411000	4.514826000	-0.781357000
6	-3.519746000	4.524752000	-0.512135000
6	-4.571277000	2.414359000	-0.048565000
1	-3.586542000	5.604034000	-0.691118000
1	-5.464967000	1.801443000	0.121817000
6	-4.694781000	3.780389000	-0.240517000

1	-5.673682000	4.267349000	-0.206680000

TS(I-II)(V)
SCF (BP86/SDD/6-31G**) Energy 333 K = -2439.44587170
Thermal correction to Gibbs Free Energy= 0.984724
Lowest Frequency = -1109.8107 cm^{-1}
Second Frequency = 15.8654 cm^{-1}
SCF (B3PW91-D3,C6H6/tzvp) Energy 333 K= -3579.24767284

1	-1.841577000	0.168502000	0.915645000
6	-1.059691000	-0.213879000	1.971430000
26	0.352082000	0.094050000	0.175251000
7	1.944480000	1.299483000	-0.387668000
6	3.191119000	0.848787000	-0.342030000
6	3.668836000	-0.480515000	-0.098798000
6	2.837320000	-1.668994000	-0.016164000
7	1.473032000	-1.590982000	0.079321000
6	0.726159000	-2.815884000	-0.098919000
6	0.377541000	-3.640323000	1.016989000
6	-0.392230000	-4.798472000	0.784053000
6	-0.808934000	-5.159963000	-0.501390000
6	-0.437493000	-4.363738000	-1.590736000
6	0.332114000	-3.197010000	-1.421140000
6	0.865271000	-3.362886000	2.443685000
6	-0.245641000	-3.543291000	3.503035000
6	0.788223000	-2.429228000	-2.664308000
6	-0.364491000	-2.127495000	-3.644938000
6	1.823031000	2.657050000	-0.903002000
6	2.078343000	3.784684000	-0.070865000
6	1.963276000	5.069151000	-0.642319000
6	1.610367000	5.247936000	-1.982482000
6	1.381079000	4.128229000	-2.789246000
6	1.488077000	2.821121000	-2.278972000
6	2.518600000	3.663758000	1.391153000
6	4.062292000	3.686038000	1.526787000
6	1.337267000	1.624986000	-3.220606000
6	0.326858000	1.859948000	-4.361191000
6	2.712382000	1.224236000	-3.810258000
6	1.931660000	4.774032000	2.291797000
6	2.066297000	-4.266289000	2.820735000
6	1.921097000	-3.195850000	-3.390400000
1	-2.050619000	-0.317753000	2.465502000
1	-0.735597000	-1.272077000	1.900548000
1	1.120721000	4.268635000	-3.842522000
1	1.521655000	6.256692000	-2.399655000
1	2.155538000	5.946984000	-0.017884000
1	-0.737282000	-4.660853000	-2.601655000
1	-1.404300000	-6.066308000	-0.655112000
1	-0.659057000	-5.437699000	1.632206000
1	1.201557000	-1.463224000	-2.325712000
1	0.965400000	0.775596000	-2.620133000
1	0.154126000	0.916388000	-4.906165000
1	-0.643259000	2.210900000	-3.975355000
1	0.699120000	2.593156000	-5.099003000
1	2.606977000	0.352675000	-4.480304000
1	3.138094000	2.056287000	-4.399059000
1	3.439503000	0.959489000	-3.025300000
1	0.010598000	-1.535433000	-4.497923000
1	-0.803840000	-3.051006000	-4.061570000
1	-1.168207000	-1.551095000	-3.159558000
1	2.280170000	-2.620035000	-4.261709000
1	2.779451000	-3.377750000	-2.723680000
1	1.560387000	-4.173552000	-3.757528000
1	2.157583000	2.691568000	1.769787000
1	4.356653000	3.633531000	2.589847000
1	4.544889000	2.844963000	1.004617000
1	4.472005000	4.621527000	1.106463000
1	2.119316000	4.536248000	3.352661000
1	2.406969000	5.750895000	2.093880000
1	0.845333000	4.892069000	2.154766000
1	1.211373000	-2.314832000	2.475782000
1	2.394608000	-4.054021000	3.853767000
1	1.782130000	-5.332750000	2.770206000
1	2.927043000	-4.107551000	2.153765000
1	0.126317000	-3.234514000	4.495352000
1	-1.144494000	-2.948902000	3.272623000
1	-0.554031000	-4.599644000	3.593006000
7	-2.736416000	0.400241000	-0.182612000
5	-1.518960000	0.798954000	-1.080198000
1	-0.860029000	1.672413000	-0.475028000

14	-0.135942000	0.678121000	3.392346000
6	1.753752000	0.417743000	3.360487000
1	2.249209000	1.152772000	4.019833000
1	2.204778000	0.514998000	2.358356000
6	-0.547738000	2.531161000	3.392835000
1	0.047502000	3.065326000	4.153629000
1	-0.348603000	2.995402000	2.413815000
1	-1.612553000	2.695650000	3.629405000
6	-0.755875000	-0.005078000	5.063187000
1	-0.571407000	-1.087663000	5.163231000
1	-0.247175000	0.500471000	5.903727000
1	-1.840930000	0.161612000	5.185078000
1	2.012611000	-0.587334000	3.736074000
1	-0.826359000	-0.264696000	-1.183944000
1	-1.720983000	1.147635000	-2.223777000
6	-3.320375000	-0.942243000	-0.516407000
6	-4.080210000	-0.997807000	-1.861802000
6	-4.505475000	-2.441379000	-2.201795000
6	-5.354609000	-3.058855000	-1.074875000
6	-4.618485000	-2.982080000	0.275895000
6	-4.199257000	-1.534463000	0.610299000
1	-2.438580000	-1.607940000	-0.612699000
1	-3.448742000	-0.578625000	-2.664811000
1	-4.984747000	-0.360605000	-1.797709000
1	-5.058889000	-2.458603000	-3.158711000
1	-3.597289000	-3.058200000	-2.351227000
1	-5.611037000	-4.107285000	-1.314260000
1	-6.314820000	-2.509794000	-0.999042000
1	-5.252706000	-3.386187000	1.086187000
1	-3.713459000	-3.619530000	0.234850000
1	-3.652541000	-1.510434000	1.569701000
1	-5.113549000	-0.924020000	0.746583000
6	-3.762896000	1.480776000	0.038679000
6	-3.983230000	2.419535000	-1.172109000
6	-5.091478000	3.453066000	-0.884927000
6	-4.770216000	4.285442000	0.369616000
6	-4.524752000	3.370138000	1.582577000
6	-3.436940000	2.315836000	1.294222000
1	-4.728253000	0.974000000	0.233741000
1	-4.228160000	1.831296000	-2.072894000
1	-3.038441000	2.949437000	-1.393531000
1	-5.225923000	4.111597000	-1.762579000
1	-6.057967000	2.929411000	-0.737306000
1	-5.587360000	4.998719000	0.583662000
1	-3.862349000	4.891860000	0.179777000
1	-4.243970000	3.966930000	2.469924000
1	-5.470836000	2.852998000	1.841964000
1	-3.326038000	1.646765000	2.165567000
1	-2.460961000	2.815868000	1.149195000
1	3.983702000	1.575395000	-0.586273000
6	3.524698000	-2.930109000	-0.082464000
1	2.923173000	-3.841627000	-0.104786000
6	4.910204000	-3.020255000	-0.096429000
6	5.091906000	-0.622304000	-0.106717000
1	5.378721000	-4.010553000	-0.122412000
1	5.692443000	0.294019000	-0.157374000
6	5.719427000	-1.857107000	-0.082389000
1	6.810386000	-1.932496000	-0.086916000

TS(I-II)(III)
SCF (BP86/SDD/6-31G**) Energy 333 K = -2439.44752712
Thermal correction to Gibbs Free Energy= 0.989214
Lowest Frequency = -1206.5707 cm^{-1}
Second Frequency = 16.5364 cm^{-1}
SCF (B3PW91-D3,C6H6/tzvp) Energy 333 K= -3579.24379270

6	-1.098807000	0.907189000	1.770036000
1	-1.807314000	0.470776000	0.677354000
26	0.318641000	-0.018083000	0.200401000
7	1.626690000	1.421031000	0.318576000
6	2.914443000	1.218111000	0.565449000
6	3.588139000	-0.035142000	0.644303000
6	2.993330000	-1.283770000	0.213777000
7	1.661759000	-1.386989000	-0.127125000
6	1.317907000	-2.592824000	-0.863138000
6	0.769112000	-3.730805000	-0.198367000
6	0.508388000	-4.890859000	-0.954025000
6	0.768104000	-4.951033000	-2.327380000
6	1.298580000	-3.829665000	-2.969288000

6	1.583959000	-2.644766000	-2.263320000
6	0.493771000	-3.755867000	1.306616000
6	-0.801581000	-4.519171000	1.658388000
6	2.200242000	-1.478038000	-3.035185000
6	1.317729000	-1.063170000	-4.232783000
6	1.269943000	2.814550000	0.069493000
6	1.177755000	3.754539000	1.138044000
6	0.810210000	5.081036000	0.828866000
6	0.554033000	5.486620000	-0.482569000
6	0.704789000	4.567141000	-1.525958000
6	1.077205000	3.231496000	-1.282104000
6	1.538581000	3.426711000	2.590113000
6	2.959311000	3.941141000	2.933419000
6	1.400350000	2.317739000	-2.464816000
6	0.531085000	2.582259000	-3.709200000
6	2.900681000	2.445607000	-2.829037000
6	0.530316000	4.012412000	3.602471000
6	1.678160000	-4.358800000	2.100676000
6	3.635993000	-1.810133000	-3.505716000
1	-0.545137000	1.857610000	1.684207000
1	-2.123251000	1.267857000	2.018424000
1	0.549721000	4.896278000	-2.557498000
1	0.262804000	6.520835000	-0.694498000
1	0.733006000	5.812029000	1.639772000
1	1.506352000	-3.870845000	-4.044347000
1	0.558635000	-5.866852000	-2.890513000
1	0.097782000	-5.771924000	-0.451111000
1	2.263081000	-0.617489000	-2.347983000
1	1.218544000	1.276900000	-2.144676000
1	0.727609000	1.814891000	-4.475107000
1	-0.543966000	2.551771000	-3.467333000
1	0.750884000	3.561368000	-4.170630000
1	3.154214000	1.772334000	-3.666594000
1	3.139020000	3.479252000	-3.137328000
1	3.552444000	2.186701000	-1.978541000
1	1.765796000	-0.202259000	-4.759812000

1	1.221884000	-1.879798000	-4.969794000
1	0.303075000	-0.786077000	-3.902666000
1	4.083778000	-0.944500000	-4.025648000
1	4.286285000	-2.073138000	-2.655622000
1	3.636284000	-2.660803000	-4.210655000
1	1.536812000	2.328788000	2.703112000
1	3.222927000	3.682832000	3.974022000
1	3.731383000	3.513987000	2.272852000
1	3.008923000	5.039702000	2.832897000
1	0.785580000	3.682237000	4.623864000
1	0.552553000	5.115910000	3.610473000
1	-0.501584000	3.694616000	3.383622000
1	0.371103000	-2.707010000	1.630060000
1	1.453388000	-4.364477000	3.181998000
1	1.863089000	-5.402622000	1.788315000
1	2.608624000	-3.789363000	1.949825000
1	-1.030121000	-4.407621000	2.731457000
1	-1.665243000	-4.150109000	1.079091000
1	-0.705927000	-5.601954000	1.464435000
7	-2.665276000	-0.043822000	-0.367333000
5	-1.387604000	-0.690638000	-0.987918000
1	-0.606904000	0.223236000	-1.388578000
14	-0.682981000	-0.033416000	3.382757000
6	-1.138125000	1.018490000	4.911457000
1	-1.045068000	0.405446000	5.826306000
1	-0.492663000	1.901488000	5.037311000
1	-2.183030000	1.372615000	4.858144000
6	1.146711000	-0.525396000	3.558861000
1	1.284115000	-1.129546000	4.473738000
1	1.800000000	0.359476000	3.649237000
6	-1.781169000	-1.576590000	3.539257000
1	-1.721636000	-2.234767000	2.659967000
1	-1.487716000	-2.168084000	4.424825000
1	-2.837960000	-1.287549000	3.674686000
1	1.514259000	-1.120328000	2.706440000
1	-0.799843000	-1.293231000	0.006911000

1	-1.513232000	-1.535594000	-1.849392000
6	-3.593413000	-1.059121000	0.234718000
6	-4.362494000	-1.918190000	-0.797629000
6	-5.196903000	-3.016281000	-0.107023000
6	-6.171573000	-2.418067000	0.923801000
6	-5.421386000	-1.547615000	1.948721000
6	-4.581134000	-0.451744000	1.259064000
1	-2.926781000	-1.744073000	0.796548000
1	-3.650678000	-2.363782000	-1.513203000
1	-5.044383000	-1.266034000	-1.378603000
1	-5.746016000	-3.605318000	-0.864274000
1	-4.515097000	-3.724926000	0.404441000
1	-6.732817000	-3.219576000	1.438137000
1	-6.922684000	-1.796145000	0.396529000
1	-6.131326000	-1.084506000	2.658305000
1	-4.752457000	-2.193560000	2.550932000
1	-4.029298000	0.136442000	2.013788000
1	-5.269835000	0.251748000	0.751842000
6	-3.364878000	0.999865000	-1.193831000
6	-3.313136000	0.756730000	-2.721046000
6	-4.143380000	1.814827000	-3.477030000
6	-3.678489000	3.245240000	-3.146516000
6	-3.675184000	3.492725000	-1.626683000
6	-2.862824000	2.423412000	-0.867111000
1	-4.434057000	0.972444000	-0.905379000
1	-3.674714000	-0.257540000	-2.960561000
1	-2.260775000	0.800383000	-3.060027000
1	-4.086714000	1.631595000	-4.565709000
1	-5.211489000	1.706622000	-3.198867000
1	-4.320416000	3.987552000	-3.655902000
1	-2.654398000	3.394815000	-3.541764000
1	-3.275607000	4.497500000	-1.398948000
1	-4.720832000	3.481051000	-1.257347000
1	-2.934007000	2.603837000	0.220169000
1	-1.790963000	2.508467000	-1.130194000
1	3.549743000	2.111437000	0.664605000
6	3.886124000	-2.409128000	0.133374000
1	3.491968000	-3.358387000	-0.233846000
6	5.214866000	-2.331292000	0.531415000
6	4.963212000	0.006726000	1.028982000
1	5.837406000	-3.231230000	0.474304000
1	5.373611000	0.973457000	1.344772000
6	5.773241000	-1.116087000	1.000013000
1	6.821362000	-1.061723000	1.307596000

II (V)
SCF (BP86/SDD/6-31G**) Energy 333 K = -1990.31424993
Thermal correction to Gibbs Free Energy= 0.847176
Lowest Frequency = -6.3256 cm^{-1}
Second Frequency = 10.6990 cm^{-1}
SCF (B3PW91-D3,C6H6/tzvp) Energy 333 K= -3130.10053057

26	-0.445056000	0.315104000	-0.000585000
7	-1.367134000	2.107295000	-0.003168000
6	-2.685065000	2.194541000	-0.003362000
6	-3.654170000	1.136102000	-0.002751000
6	-3.357217000	-0.288628000	-0.001894000
7	-2.073282000	-0.758110000	-0.001242000
6	-1.831463000	-2.175167000	-0.000549000
6	-1.688661000	-2.865392000	-1.241803000
6	-1.409960000	-4.245251000	-1.210923000
6	-1.273005000	-4.935332000	0.000794000
6	-1.411127000	-4.244306000	1.211840000
6	-1.689858000	-2.864421000	1.241388000
6	-1.854486000	-2.156185000	-2.586521000
6	-0.590349000	-2.285485000	-3.464654000
6	-1.857044000	-2.154192000	2.585402000
6	-0.593928000	-2.283047000	3.465066000
6	-0.611594000	3.339858000	-0.004004000
6	-0.231059000	3.913541000	-1.246722000
6	0.493901000	5.120150000	-1.217209000
6	0.847629000	5.727502000	-0.005949000
6	0.490797000	5.124021000	1.206305000
6	-0.234260000	3.917494000	1.237834000
6	-0.605344000	3.269962000	-2.582766000
6	-1.756702000	4.041785000	-3.268424000
6	-0.611278000	3.278202000	2.575172000

6	0.602153000	3.138823000	3.519553000
6	-1.760556000	4.055103000	3.258551000
6	0.608717000	3.131857000	-3.526468000
6	-3.106260000	-2.664417000	-3.338899000
6	-3.109732000	-2.661665000	3.336777000
1	0.780872000	5.596599000	2.150899000
1	1.409033000	6.667780000	-0.006733000
1	0.786377000	5.589755000	-2.162531000
1	-1.307139000	-4.787471000	2.157898000
1	-1.061451000	-6.009840000	0.001316000
1	-1.305052000	-4.789150000	-2.156459000
1	-2.003147000	-1.080661000	2.374532000
1	-0.974951000	2.256493000	2.364079000
1	0.307661000	2.603970000	4.439150000
1	1.416587000	2.573110000	3.038406000
1	1.002392000	4.121430000	3.825287000
1	-2.046462000	3.572561000	4.209577000
1	-1.456773000	5.093100000	3.482677000
1	-2.657174000	4.102626000	2.617064000
1	-0.726034000	-1.727443000	4.410126000
1	-0.383282000	-3.335401000	3.725223000
1	0.291877000	-1.876083000	2.949312000
1	-3.241192000	-2.110404000	4.284459000
1	-4.022264000	-2.524704000	2.733133000
1	-3.022857000	-3.735030000	3.582894000
1	-0.966085000	2.247680000	-2.369270000
1	-2.040674000	3.556264000	-4.218517000
1	-2.653786000	4.087966000	-2.627493000
1	-1.455939000	5.080208000	-3.494650000
1	0.316152000	2.593246000	-4.444479000
1	1.005703000	4.114835000	-3.835238000
1	1.424986000	2.570331000	-3.043532000
1	-2.001004000	-1.082525000	-2.376597000
1	-3.236786000	-2.113866000	-4.287123000
1	-3.018944000	-3.737945000	-3.584146000
1	-4.019471000	-2.527178000	-2.736343000
1	-0.721487000	-1.730675000	-4.410315000
1	0.294784000	-1.877930000	-2.948208000
1	-0.379189000	-3.338007000	-3.723712000
7	2.846393000	-0.697437000	0.004357000
5	1.708590000	0.224614000	0.002383000
1	1.970680000	1.416110000	0.002048000
1	0.897869000	0.039013000	-1.059341000
1	0.895143000	0.040089000	1.062050000
6	2.625989000	-2.150482000	0.004580000
6	3.163346000	-2.852801000	1.277845000
6	2.859210000	-4.364602000	1.273822000
6	3.405894000	-5.046213000	0.005300000
6	2.859885000	-4.365081000	-1.263770000
6	3.163976000	-2.853275000	-1.268168000
1	1.525316000	-2.288785000	0.004343000
1	2.725323000	-2.367529000	2.169203000
1	4.259858000	-2.704687000	1.342923000
1	3.277952000	-4.839688000	2.180539000
1	1.761796000	-4.512031000	1.317643000
1	3.150741000	-6.122227000	0.005436000
1	4.513101000	-4.985818000	0.005588000
1	3.279148000	-4.840487000	-2.170080000
1	1.762502000	-4.512560000	-1.308151000
1	2.726366000	-2.368337000	-2.159912000
1	4.260514000	-2.705126000	-1.332738000
6	4.235942000	-0.210109000	0.004748000
6	4.585524000	0.603842000	1.275413000
6	6.051915000	1.080358000	1.274283000
6	6.382355000	1.887610000	0.004564000
6	6.053494000	1.078512000	-1.264389000
6	4.587089000	0.602033000	-1.266653000
1	4.895897000	-1.101488000	0.005782000
1	4.373436000	-0.010188000	2.169609000
1	3.910650000	1.480164000	1.324155000
1	6.258372000	1.680943000	2.179654000
1	6.723518000	0.199160000	1.326415000
1	7.445956000	2.190367000	0.005006000
1	5.787573000	2.822901000	0.003510000
1	6.261096000	1.677774000	-2.170376000
1	6.725142000	0.197225000	-1.314398000

1 4.376101000 -0.013252000 -2.160246000

1 3.912311000 1.478310000 -1.317472000

1 -3.108389000 3.214402000 -0.004173000

6 -4.480053000 -1.180509000 -0.001825000

1 -4.280840000 -2.255160000 -0.001280000

6 -5.789627000 -0.715603000 -0.002388000

6 -5.016990000 1.562993000 -0.003287000

1 -6.610448000 -1.441705000 -0.002274000

1 -5.211560000 2.642284000 -0.003907000

6 -6.078586000 0.670828000 -0.003096000

1 -7.111799000 1.028242000 -0.003521000

II (III)
SCF (BP86/SDD/6-31G**) Energy 333 K = -1990.33473648
Thermal correction to Gibbs Free Energy= 0.848547
Lowest Frequency = 9.6967 cm^{-1}
Second Frequency = 12.6834 cm^{-1}
SCF (B3PW91-D3,C6H6/tzvp) Energy 333 K= -3130.10107962

26 0.473776000 -0.181983000 -0.155853000

7 1.456469000 -1.810468000 -0.190693000

6 2.768504000 -1.908420000 -0.382449000

6 3.696731000 -0.830634000 -0.468481000

6 3.323426000 0.562248000 -0.318481000

7 2.015920000 0.937604000 -0.134185000

6 1.755354000 2.343668000 0.073600000

6 1.485793000 3.183032000 -1.045630000

6 1.208721000 4.543324000 -0.811867000

6 1.197668000 5.073533000 0.484665000

6 1.468570000 4.237311000 1.574439000

6 1.752774000 2.869162000 1.396882000

6 1.501336000 2.650062000 -2.479338000

6 0.157406000 2.897793000 -3.199618000

6 2.067818000 1.998430000 2.614219000

6 0.940062000 2.045106000 3.668172000

6 0.738372000 -3.065301000 -0.078182000

6 0.246503000 -3.691810000 -1.254508000

6 -0.431422000 -4.917962000 -1.114142000

6 -0.625674000 -5.502692000 0.143603000

6 -0.144398000 -4.859932000 1.290872000

6 0.541376000 -3.632438000 1.208864000

6 0.455006000 -3.083792000 -2.642208000

6 1.529438000 -3.862789000 -3.436034000

6 1.082046000 -2.962738000 2.472771000

6 0.032896000 -2.893035000 3.602423000

6 2.370749000 -3.664976000 2.961487000

6 -0.860906000 -2.988982000 -3.444122000

6 2.676295000 3.242403000 -3.291360000

6 3.426091000 2.385888000 3.244175000

1 -0.300006000 -5.320405000 2.272462000

1 -1.152821000 -6.458843000 0.229646000

1 -0.810360000 -5.424110000 -2.008846000

1 1.464506000 4.653886000 2.588201000

1 0.981920000 6.135466000 0.644434000

1 1.002086000 5.199822000 -1.664968000

1 2.148178000 0.955820000 2.259908000

1 1.347138000 -1.923944000 2.205847000

1 0.434024000 -2.324791000 4.459445000

1 -0.889741000 -2.394815000 3.261065000

1 -0.241248000 -3.895664000 3.975117000

1 2.774047000 -3.161619000 3.857724000

1 2.169473000 -4.718725000 3.224707000

1 3.154142000 -3.657347000 2.185103000

1 1.169413000 1.365596000 4.507904000

1 0.814799000 3.058612000 4.088550000

1 -0.025582000 1.739240000 3.231763000

1 3.660457000 1.726576000 4.098718000

1 4.245226000 2.300152000 2.511349000

1 3.409818000 3.425935000 3.616583000

1 0.825309000 -2.052810000 -2.499600000

1 1.696063000 -3.400588000 -4.424905000

1 2.493825000 -3.879823000 -2.900858000

1 1.219038000 -4.910243000 -3.598904000

1 -0.685493000 -2.476238000 -4.405971000

1 -1.276556000 -3.985674000 -3.674194000

1 -1.626572000 -2.423617000 -2.887101000

1 1.649846000 1.557472000 -2.421197000

1 2.693840000 2.820951000 -4.312109000

1 2.585812000 4.339685000 -3.382821000

1 3.644605000 3.019633000 -2.813850000

1	0.173447000	2.451354000	-4.209679000
1	-0.682317000	2.452966000	-2.639370000
1	-0.049127000	3.976309000	-3.317291000
7	-2.830981000	0.459629000	0.042602000
5	-1.422794000	0.184149000	0.191044000
1	-0.998295000	-0.886705000	-0.529117000
1	-0.612978000	1.059578000	-0.436711000
1	-0.945294000	0.035224000	1.329877000
6	-3.395421000	0.620167000	-1.312220000
6	-3.967444000	2.035789000	-1.568756000
6	-4.489488000	2.184607000	-3.012599000
6	-5.529141000	1.100490000	-3.353299000
6	-4.957374000	-0.308939000	-3.110675000
6	-4.437804000	-0.468345000	-1.667099000
1	-2.541390000	0.486819000	-2.006225000
1	-3.184731000	2.786126000	-1.355673000
1	-4.798687000	2.231466000	-0.862757000
1	-4.918136000	3.193089000	-3.157338000
1	-3.637472000	2.104098000	-3.716957000
1	-5.864732000	1.204574000	-4.401342000
1	-6.427387000	1.243988000	-2.719669000
1	-5.719683000	-1.079980000	-3.325622000
1	-4.123780000	-0.488430000	-3.818922000
1	-3.983592000	-1.465256000	-1.521001000
1	-5.293745000	-0.402306000	-0.966565000
6	-3.745760000	0.595934000	1.194070000
6	-3.384568000	1.800267000	2.095685000
6	-4.363328000	1.945860000	3.278044000
6	-4.448256000	0.648919000	4.104727000
6	-4.818148000	-0.551807000	3.213519000
6	-3.839656000	-0.701689000	2.031357000
1	-4.756962000	0.789602000	0.784327000
1	-3.364159000	2.721341000	1.486003000
1	-2.356561000	1.653934000	2.480278000
1	-4.059712000	2.794672000	3.917624000
1	-5.371842000	2.194329000	2.889439000
1	-5.181966000	0.761165000	4.923964000
1	-3.467371000	0.458038000	4.583812000
1	-4.839198000	-1.483997000	3.807222000
1	-5.845874000	-0.409416000	2.822000000
1	-4.138745000	-1.539486000	1.376064000
1	-2.829570000	-0.944576000	2.414840000
1	3.183331000	-2.925361000	-0.466341000
6	4.389608000	1.523782000	-0.360828000
1	4.139792000	2.580023000	-0.236803000
6	5.710417000	1.145762000	-0.563993000
6	5.067401000	-1.173583000	-0.675370000
1	6.485615000	1.919518000	-0.597173000
1	5.315349000	-2.236116000	-0.787082000
6	6.067756000	-0.216630000	-0.729650000
1	7.110569000	-0.504966000	-0.890107000

2b (V)
SCF (BP86/SDD/6-31G**) Energy 333 K = -1438.92820774
Thermal correction to Gibbs Free Energy= 0.525770
Lowest Frequency = 6.9360 cm^{-1}
Second Frequency = 16.4803 cm^{-1}
SCF (B3PW91-D3,C6H6/tzvp) Energy 333 K= -2578.66450851

26	0.147901000	0.003923000	-1.077133000
7	1.546122000	0.000365000	0.330573000
6	1.299609000	-0.003446000	1.633925000
6	0.012610000	-0.005848000	2.264805000
6	-1.260490000	-0.004621000	1.562896000
7	-1.317820000	-0.000488000	0.191739000
6	-2.593676000	0.000344000	-0.472190000
6	-3.203930000	1.240871000	-0.822085000
6	-4.422217000	1.212581000	-1.526313000
6	-5.031197000	0.002376000	-1.881242000
6	-4.419928000	-1.208827000	-1.533764000
6	-3.201524000	-1.239153000	-0.829803000
6	-2.567020000	2.583877000	-0.464586000
6	-2.163939000	3.366219000	-1.735683000
6	-2.562129000	-2.583172000	-0.480613000
6	-2.159833000	-3.358126000	-1.756463000
6	2.922518000	0.001534000	-0.103708000
6	3.574561000	1.243415000	-0.338267000
6	4.910968000	1.214339000	-0.780218000
6	5.580895000	0.004059000	-0.997215000

6	4.913197000	-1.207479000	-0.780421000
6	3.576828000	-1.239126000	-0.338481000
6	2.874420000	2.585653000	-0.121738000
6	3.511914000	3.372673000	1.046071000
6	2.879084000	-2.582748000	-0.122706000
6	2.857488000	-3.427113000	-1.416810000
6	3.517491000	-3.369001000	1.045121000
6	2.850543000	3.430359000	-1.415565000
6	-3.484657000	3.431374000	0.444885000
6	-3.477280000	-3.436768000	0.425672000
1	5.437945000	-2.152754000	-0.958011000
1	6.621023000	0.005042000	-1.339639000
1	5.433994000	2.160613000	-0.957560000
1	-4.896073000	-2.154518000	-1.816629000
1	-5.978957000	0.003168000	-2.430047000
1	-4.900128000	2.159088000	-1.803402000
1	-1.638318000	-2.372762000	0.086629000
1	1.829808000	-2.375969000	0.153004000
1	2.299869000	-4.365895000	-1.254117000
1	2.376375000	-2.878195000	-2.243601000
1	3.877313000	-3.698582000	-1.741353000
1	2.981302000	-4.319367000	1.212872000
1	4.573664000	-3.611835000	0.832832000
1	3.490503000	-2.790047000	1.983732000
1	-1.654773000	-4.304323000	-1.493379000
1	-3.043940000	-3.609526000	-2.368657000
1	-1.476639000	-2.762694000	-2.386275000
1	-2.975954000	-4.381290000	0.700838000
1	-3.730483000	-2.900885000	1.355537000
1	-4.421982000	-3.697464000	-0.083541000
1	1.825677000	2.376741000	0.154507000
1	2.973932000	4.321903000	1.214510000
1	3.486586000	2.793256000	1.984442000
1	4.567482000	3.617701000	0.833292000
1	2.291460000	4.368160000	-1.252239000
1	3.869713000	3.703616000	-1.740683000
1	2.369798000	2.880847000	-2.242180000
1	-1.643776000	2.371695000	0.102894000
1	-2.984856000	4.374933000	0.726057000
1	-4.428847000	3.693848000	-0.064360000
1	-3.738735000	2.890095000	1.371380000
1	-1.660682000	4.311761000	-1.466841000
1	-1.478936000	2.775151000	-2.367648000
1	-3.047532000	3.619494000	-2.347854000
1	0.234446000	0.008583000	-2.685841000
1	2.170411000	-0.005035000	2.311140000
6	-2.449679000	-0.007802000	2.361977000
1	-3.414070000	-0.007007000	1.847044000
6	-2.398943000	-0.011739000	3.750169000
6	0.012391000	-0.009906000	3.692471000
1	-3.335314000	-0.014033000	4.319118000
1	0.983689000	-0.010732000	4.201741000
6	-1.158582000	-0.012806000	4.435374000
1	-1.125499000	-0.015881000	5.528603000

2b (I)
SCF (BP86/SDD/6-31G**) Energy 333 K = -1438.90197017
Thermal correction to Gibbs Free Energy= 0.533565
Lowest Frequency = 14.5682 cm^{-1}
Second Frequency = 24.1651 cm^{-1}
SCF (B3PW91-D3,C6H6/tzvp) Energy 333 K= -2578.60718001

26	0.174518000	0.028970000	-0.821219000
7	-1.242629000	-0.012489000	0.250473000
7	1.447948000	-0.064996000	0.413080000
6	-1.270857000	-0.217940000	1.611000000
6	-0.027151000	-0.342615000	2.345772000
6	-0.091590000	-0.530824000	3.759493000
1	0.853134000	-0.625131000	4.308165000
6	-1.299532000	-0.592970000	4.439284000
1	-1.316984000	-0.740827000	5.523288000
6	-2.512015000	-0.459016000	3.715298000
1	-3.471002000	-0.506071000	4.242373000
6	-2.498481000	-0.273105000	2.337571000
1	-3.435246000	-0.170975000	1.781429000
6	2.787096000	0.041930000	-0.122350000
6	3.358148000	1.333312000	-0.308452000
6	4.648226000	1.408286000	-0.869228000
1	5.108995000	2.390970000	-1.018477000
6	5.351762000	0.253625000	-1.231851000

1	6.354449000	0.335717000	-1.664920000
6	4.772916000	-1.007279000	-1.036976000
1	5.331557000	-1.906721000	-1.317897000
6	3.485748000	-1.145470000	-0.484956000
6	-2.436357000	0.071113000	-0.548404000
6	-3.022163000	-1.122799000	-1.073210000
6	-4.140720000	-0.996496000	-1.917749000
1	-4.601593000	-1.900644000	-2.330554000
6	-4.674934000	0.258035000	-2.239956000
1	-5.544011000	0.330212000	-2.902832000
6	-4.099378000	1.418971000	-1.709182000
1	-4.526659000	2.396615000	-1.958842000
6	-2.982424000	1.355511000	-0.854461000
6	1.254791000	-0.242789000	1.727710000
1	2.147917000	-0.285905000	2.366296000
6	2.639176000	2.616846000	0.108188000
1	1.625954000	2.336902000	0.446016000
6	2.474531000	3.594261000	-1.076026000
1	1.916712000	4.492208000	-0.758319000
1	1.920797000	3.123243000	-1.906247000
1	3.450243000	3.930467000	-1.468686000
6	2.898328000	-2.538049000	-0.258088000
1	1.849754000	-2.409042000	0.057531000
6	2.882010000	-3.375429000	-1.555033000
1	2.411649000	-4.356452000	-1.368184000
1	3.900447000	-3.564431000	-1.938214000
1	2.306721000	-2.864392000	-2.344583000
6	-2.493504000	-2.512879000	-0.720386000
1	-1.571000000	-2.376433000	-0.131818000
6	-3.509600000	-3.287279000	0.151624000
1	-3.101686000	-4.274998000	0.429368000
1	-4.458463000	-3.455794000	-0.388435000
1	-3.738873000	-2.741067000	1.081482000
6	-2.407333000	2.644175000	-0.267355000
1	-1.553242000	2.361436000	0.371918000
6	-1.871359000	3.580227000	-1.372997000
1	-1.436819000	4.493619000	-0.930336000
1	-2.673075000	3.892234000	-2.065453000
1	-1.086810000	3.080858000	-1.968197000
6	3.359793000	3.296813000	1.295146000
1	2.810119000	4.198794000	1.616405000
1	4.383386000	3.606684000	1.019436000
1	3.437162000	2.617177000	2.160391000
6	3.653150000	-3.276314000	0.872046000
1	3.198514000	-4.265080000	1.058042000
1	3.625611000	-2.705738000	1.815889000
1	4.713842000	-3.437023000	0.608863000
6	-2.116053000	-3.320977000	-1.980850000
1	-1.705601000	-4.304765000	-1.693435000
1	-1.352132000	-2.789828000	-2.572557000
1	-2.990798000	-3.503639000	-2.629969000
6	-3.441835000	3.368682000	0.623917000
1	-2.993187000	4.268743000	1.079548000
1	-3.794518000	2.714354000	1.438278000
1	-4.323371000	3.692549000	0.042677000
1	0.331075000	-1.286017000	-1.539850000

2b (III)
SCF (BP86/SDD/6-31G**) Energy 333 K = -1438.91329763
Thermal correction to Gibbs Free Energy= 0.528340
Lowest Frequency = 11.4792 cm^{-1}
Second Frequency = 19.6296 cm^{-1}
SCF (B3PW91-D3,C6H6/tzvp) Energy 333 K= -2578.63488073

26	0.089582000	-0.174017000	-0.934795000
7	-1.308201000	-0.061793000	0.299994000
7	1.437203000	-0.069092000	0.310591000
6	-1.283403000	-0.047743000	1.661632000
6	0.000026000	-0.101350000	2.332907000
6	0.025978000	-0.113316000	3.759969000
1	1.001965000	-0.157572000	4.257907000
6	-1.138041000	-0.064276000	4.513382000
1	-1.091962000	-0.074068000	5.606242000
6	-2.391055000	0.003216000	3.849624000
1	-3.314875000	0.042710000	4.437271000
6	-2.465636000	0.016211000	2.462239000
1	-3.433773000	0.062678000	1.953863000
6	2.807003000	-0.008467000	-0.157757000
6	3.406006000	1.263804000	-0.357569000
6	4.748226000	1.300827000	-0.781934000

1	5.233512000	2.271239000	-0.934036000
6	5.470898000	0.123597000	-1.009565000
1	6.515206000	0.174360000	-1.335636000
6	4.852387000	-1.119536000	-0.823951000
1	5.419793000	-2.037939000	-1.010234000
6	3.513214000	-1.216633000	-0.402550000
6	-2.499622000	0.028571000	-0.483072000
6	-3.142941000	-1.171658000	-0.924260000
6	-4.247511000	-1.057625000	-1.788987000
1	-4.748442000	-1.967751000	-2.137048000
6	-4.715142000	0.192523000	-2.213281000
1	-5.574447000	0.256220000	-2.889360000
6	-4.081839000	1.362336000	-1.771866000
1	-4.454516000	2.336805000	-2.107026000
6	-2.975348000	1.311410000	-0.905476000
6	1.245199000	-0.111767000	1.635054000
1	2.156421000	-0.132679000	2.250808000
6	2.646997000	2.569370000	-0.118711000
1	1.593321000	2.309794000	0.086911000
6	2.661732000	3.476588000	-1.369051000
1	2.049587000	4.378944000	-1.196331000
1	2.259224000	2.948866000	-2.249815000
1	3.683546000	3.813251000	-1.617161000
6	2.869255000	-2.589353000	-0.213828000
1	1.794872000	-2.427627000	-0.019661000
6	2.971043000	-3.447572000	-1.493246000
1	2.448959000	-4.409708000	-1.349131000
1	4.019894000	-3.675900000	-1.752716000
1	2.508016000	-2.926608000	-2.346954000
6	-2.676224000	-2.552457000	-0.465127000
1	-1.812717000	-2.402124000	0.205610000
6	-3.776652000	-3.278161000	0.342889000
1	-3.404146000	-4.250428000	0.709696000
1	-4.670926000	-3.474978000	-0.274683000
1	-4.091918000	-2.682799000	1.215956000
6	-2.312371000	2.606936000	-0.439066000
1	-1.493029000	2.330948000	0.247228000
6	-1.688973000	3.378769000	-1.624370000
1	-1.184786000	4.294343000	-1.269187000
1	-2.456790000	3.680918000	-2.358238000
1	-0.943280000	2.763401000	-2.158235000
6	3.193216000	3.318625000	1.118209000
1	2.618131000	4.243646000	1.299451000
1	4.251397000	3.601539000	0.976857000
1	3.132559000	2.695063000	2.026103000
6	3.467795000	-3.325969000	1.006453000
1	2.972423000	-4.302008000	1.151892000
1	3.346102000	-2.740229000	1.933670000
1	4.547724000	-3.513397000	0.869662000
6	-2.195623000	-3.413487000	-1.654900000
1	-1.841705000	-4.396913000	-1.298945000
1	-1.364994000	-2.920362000	-2.188421000
1	-3.008999000	-3.593197000	-2.380286000
6	-3.298481000	3.499163000	0.348305000
1	-2.786695000	4.404790000	0.717851000
1	-3.713922000	2.963202000	1.217606000
1	-4.142611000	3.827138000	-0.283646000
1	0.809055000	-0.868400000	-2.169830000

III (V)
SCF (BP86/SDD/6-31G**) Energy 333 K = -2438.29208794
Thermal correction to Gibbs Free Energy= 0.962487
Lowest Frequency = 11.2144 cm^{-1}
Second Frequency = 11.4789 cm^{-1}
SCF (B3PW91-D3,C6H6/tzvp) Energy 333 K= -3578.07718528

26	-0.494553000	-0.104474000	0.584996000
6	-0.594485000	0.601142000	2.493885000
6	-0.907272000	-0.276846000	5.468833000
1	-1.606564000	-0.595274000	6.262694000
7	-0.565440000	-2.138768000	0.145602000
6	-1.778047000	-2.643200000	-0.030002000
6	-2.990987000	-1.958881000	-0.379113000
6	-3.076434000	-0.573544000	-0.832378000
7	-2.025765000	0.289944000	-0.705358000
6	-2.098984000	1.534382000	-1.429298000
6	-2.602646000	2.718084000	-0.812126000
6	-2.649712000	3.909581000	-1.564071000
6	-2.210121000	3.959152000	-2.890715000
6	-1.699280000	2.798606000	-3.482623000

6	-1.628054000	1.582494000	-2.777420000
6	-3.122686000	2.738714000	0.624703000
6	-2.454111000	3.854533000	1.458573000
6	-1.067997000	0.349560000	-3.486447000
6	0.311114000	0.627804000	-4.123851000
6	0.533981000	-3.072699000	0.111639000
6	1.309098000	-3.319436000	1.280711000
6	2.391558000	-4.215883000	1.196666000
6	2.715925000	-4.864265000	0.000534000
6	1.948045000	-4.614250000	-1.140939000
6	0.858329000	-3.721308000	-1.120479000
6	0.966383000	-2.714772000	2.640296000
6	0.480907000	-3.811157000	3.617555000
6	0.084125000	-3.493974000	-2.422442000
6	1.021561000	-3.115791000	-3.591230000
6	-0.766625000	-4.730416000	-2.796839000
6	2.151980000	-1.928310000	3.239296000
6	-4.662095000	2.887570000	0.667874000
6	-2.062033000	-0.188353000	-4.542295000
1	0.432919000	0.449600000	2.885799000
1	-0.717823000	1.698777000	2.390416000
1	2.197399000	-5.123130000	-2.078414000
1	3.557318000	-5.564089000	-0.040331000
1	2.979611000	-4.420913000	2.098100000
1	-1.349733000	2.832966000	-4.520713000
1	-2.263469000	4.894412000	-3.458358000
1	-3.050309000	4.815890000	-1.096156000
1	-0.930633000	-0.432945000	-2.719857000
1	-0.604766000	-2.646235000	-2.270980000
1	0.429014000	-2.871630000	-4.490066000
1	1.642882000	-2.240747000	-3.339993000
1	1.698274000	-3.945143000	-3.861807000
1	-1.328355000	-4.547883000	-3.729473000
1	-0.127083000	-5.617044000	-2.954214000
1	-1.493217000	-4.981181000	-2.006132000
1	0.719189000	-0.292216000	-4.575866000
1	0.248676000	1.386041000	-4.924295000
1	1.031865000	0.991426000	-3.371569000
1	-1.666404000	-1.100466000	-5.024107000
1	-3.035256000	-0.435181000	-4.086896000
1	-2.238996000	0.560315000	-5.335394000
1	0.135928000	-2.002335000	2.493902000
1	0.178425000	-3.368372000	4.582299000
1	-0.383206000	-4.361629000	3.208430000
1	1.279141000	-4.547411000	3.819295000
1	1.868870000	-1.476795000	4.205606000
1	3.025223000	-2.579690000	3.420080000
1	2.468412000	-1.115306000	2.564162000
1	-2.861238000	1.770141000	1.084801000
1	-5.023973000	2.888034000	1.711507000
1	-4.980953000	3.837599000	0.202447000
1	-5.162068000	2.062439000	0.135108000
1	-2.776715000	3.793976000	2.511565000
1	-1.354000000	3.774081000	1.430712000
1	-2.725386000	4.858757000	1.087360000
1	-0.130096000	-1.056245000	5.380881000
1	-0.410015000	0.648238000	5.810080000
7	2.700461000	1.346362000	-0.169263000
5	1.400709000	0.825722000	-0.288841000
1	0.431635000	1.559834000	-0.181624000
6	-3.163463000	1.319385000	4.158184000
1	-3.795833000	1.505681000	3.273752000
1	-3.828183000	0.981647000	4.973415000
1	-2.719164000	2.281388000	4.467475000
6	-2.747636000	-1.603677000	3.385740000
1	-3.442044000	-1.875527000	4.201159000
1	-2.062679000	-2.455315000	3.236664000
14	-1.822721000	0.007051000	3.814377000
1	-3.340966000	-1.489296000	2.462428000
1	1.263100000	-0.312638000	-0.697627000
6	3.886656000	0.504621000	-0.474943000
6	4.858036000	0.365104000	0.719457000
6	6.033009000	-0.572948000	0.372676000
6	6.779508000	-0.101496000	-0.889242000
6	5.813996000	0.043196000	-2.080454000
6	4.634606000	0.978403000	-1.742936000

1	3.480598000	-0.502118000	-0.686854000
1	4.305835000	-0.012700000	1.598369000
1	5.261514000	1.358899000	0.996072000
1	6.725171000	-0.639205000	1.231524000
1	5.642550000	-1.596099000	0.204959000
1	7.594836000	-0.804597000	-1.138765000
1	7.259033000	0.877032000	-0.686197000
1	6.348648000	0.419368000	-2.971395000
1	5.419002000	-0.956101000	-2.351219000
1	3.922514000	1.029001000	-2.586253000
1	5.020696000	2.005005000	-1.586492000
6	2.960130000	2.766157000	0.197398000
6	2.489657000	3.088481000	1.632533000
6	2.797563000	4.551174000	2.010459000
6	2.193095000	5.538285000	0.993871000
6	2.661585000	5.216134000	-0.437923000
6	2.347660000	3.755817000	-0.818985000
1	4.057510000	2.909580000	0.171176000
1	2.969086000	2.390972000	2.342951000
1	1.400449000	2.904426000	1.697690000
1	2.421553000	4.762407000	3.027800000
1	3.896352000	4.695931000	2.048153000
1	2.459519000	6.577011000	1.261650000
1	1.087866000	5.474533000	1.037263000
1	2.189664000	5.902117000	-1.164286000
1	3.754698000	5.388373000	-0.510533000
1	2.725407000	3.524618000	-1.831362000
1	1.251944000	3.607088000	-0.847646000
1	-1.875660000	-3.741669000	0.041115000
6	-4.319666000	-0.183157000	-1.443014000
1	-4.392549000	0.827883000	-1.851098000
6	-5.421202000	-1.026676000	-1.486539000
6	-4.153557000	-2.788336000	-0.447282000
1	-6.353370000	-0.661690000	-1.932888000
1	-4.061743000	-3.820717000	-0.088492000
6	-5.359619000	-2.343698000	-0.965027000
1	-6.232530000	-3.001557000	-0.998942000

III (I)

SCF (BP86/SDD/6-31G**) Energy 333 K = -2438.28554581
Thermal correction to Gibbs Free Energy= 0.972820
Lowest Frequency = 8.2908 cm^{-1}
Second Frequency = 13.6381 cm^{-1}
SCF (B3PW91-D3,C6H6/tzvp) Energy 333 K= -3578.03826001

26	-0.374744000	0.140874000	-0.276039000
6	-0.881154000	0.545605000	-2.230012000
6	-2.234681000	1.706569000	-4.682149000
1	-3.157089000	1.819447000	-5.279932000
7	-1.236949000	1.552497000	0.616906000
6	-2.481686000	1.495557000	1.098292000
6	-3.335381000	0.353214000	1.061169000
6	-2.867615000	-0.971701000	0.708928000
7	-1.566357000	-1.182539000	0.295792000
6	-1.046786000	-2.535810000	0.447497000
6	-0.840522000	-3.394262000	-0.672778000
6	-0.249721000	-4.658015000	-0.468435000
6	0.103330000	-5.108429000	0.806319000
6	-0.148510000	-4.282977000	1.906719000
6	-0.713561000	-3.001421000	1.759942000
6	-1.299556000	-3.045562000	-2.084857000
6	-0.142493000	-3.073836000	-3.107667000
6	-0.955635000	-2.185833000	3.031900000
6	0.329172000	-2.053894000	3.878162000
6	-0.487696000	2.753692000	0.990304000
6	-0.432486000	3.883402000	0.128611000
6	0.293074000	5.018466000	0.545996000
6	0.943501000	5.060268000	1.780577000
6	0.872794000	3.947122000	2.624686000
6	0.170861000	2.782011000	2.258381000
6	-1.171024000	3.959708000	-1.203719000
6	-2.344247000	4.964670000	-1.117136000
6	0.113497000	1.618259000	3.249999000
6	1.443113000	1.412217000	4.007333000
6	-1.041424000	1.807318000	4.263554000
6	-0.227597000	4.330348000	-2.369047000
6	-2.430668000	-4.004352000	-2.528642000
6	-2.094270000	-2.796999000	3.882660000
1	-0.325565000	1.472545000	-2.468339000
1	-0.383282000	-0.238913000	-2.834497000

1	1.374514000	3.980785000	3.596317000
1	1.495313000	5.954799000	2.088463000
1	0.329519000	5.892613000	-0.113085000
1	0.102616000	-4.638157000	2.912182000
1	0.551041000	-6.098542000	0.943991000
1	-0.088990000	-5.310249000	-1.334209000
1	-1.266730000	-1.170952000	2.733451000
1	-0.087141000	0.699980000	2.668566000
1	1.393463000	0.494350000	4.614600000
1	2.295367000	1.320243000	3.314680000
1	1.656541000	2.243385000	4.702096000
1	-1.078828000	0.960707000	4.971618000
1	-0.896039000	2.733148000	4.848498000
1	-2.020824000	1.872831000	3.763308000
1	0.139790000	-1.422128000	4.763551000
1	0.678000000	-3.033658000	4.248416000
1	1.150385000	-1.601332000	3.297794000
1	-2.279247000	-2.180634000	4.780160000
1	-3.033769000	-2.858573000	3.310897000
1	-1.831351000	-3.814965000	4.221956000
1	-1.588975000	2.962733000	-1.409033000
1	-2.898701000	5.001276000	-2.071263000
1	-3.054459000	4.689381000	-0.318975000
1	-1.979920000	5.984736000	-0.901186000
1	-0.778529000	4.332405000	-3.325159000
1	0.207488000	5.337015000	-2.239878000
1	0.605885000	3.613191000	-2.457540000
1	-1.700396000	-2.021092000	-2.056864000
1	-2.793057000	-3.743237000	-3.537200000
1	-2.078458000	-5.050759000	-2.556919000
1	-3.290295000	-3.959267000	-1.838831000
1	-0.502957000	-2.772345000	-4.106962000
1	0.669771000	-2.386377000	-2.816515000
1	0.290235000	-4.085522000	-3.206029000
1	-1.829245000	2.716212000	-4.498019000
1	-1.498669000	1.163643000	-5.300933000
7	2.856444000	-0.336779000	-0.561395000
5	1.471507000	-0.077556000	-0.538919000

1	1.015832000	1.109588000	-0.603258000
6	-3.448724000	-0.850558000	-3.589655000
1	-3.816484000	-1.443443000	-2.736259000
1	-4.324737000	-0.592748000	-4.212435000
1	-2.790255000	-1.492421000	-4.198736000
6	-3.974621000	1.724064000	-2.144362000
1	-4.823772000	1.837775000	-2.843204000
1	-3.688674000	2.732720000	-1.810154000
14	-2.598365000	0.767544000	-3.051376000
1	-4.340622000	1.168315000	-1.266447000
1	0.683608000	-1.037192000	-0.859058000
6	3.333571000	-1.743102000	-0.595978000
6	4.125929000	-2.135968000	0.671846000
6	4.539928000	-3.621447000	0.632589000
6	5.338461000	-3.951648000	-0.642458000
6	4.549758000	-3.559613000	-1.906060000
6	4.133768000	-2.074136000	-1.876910000
1	2.418032000	-2.365469000	-0.611167000
1	3.508394000	-1.927156000	1.563625000
1	5.035154000	-1.507557000	0.753549000
1	5.127336000	-3.872266000	1.534524000
1	3.627956000	-4.249484000	0.666312000
1	5.594613000	-5.026493000	-0.668208000
1	6.299747000	-3.399700000	-0.625505000
1	5.143482000	-3.765932000	-2.815043000
1	3.640244000	-4.188339000	-1.975969000
1	3.523779000	-1.822274000	-2.763233000
1	5.043545000	-1.443446000	-1.923691000
6	3.877185000	0.741616000	-0.544941000
6	3.832418000	1.575882000	0.754311000
6	4.912689000	2.675664000	0.760699000
6	4.799344000	3.583517000	-0.478478000
6	4.852544000	2.755330000	-1.776352000
6	3.772784000	1.655292000	-1.787765000
1	4.866576000	0.246016000	-0.583694000
1	3.955240000	0.902116000	1.621847000
1	2.831656000	2.039669000	0.848094000
1	4.832821000	3.272725000	1.686920000

1 5.916543000 2.204694000 0.777307000

1 5.602377000 4.343050000 -0.473951000

1 3.839555000 4.134895000 -0.435580000

1 4.732861000 3.408295000 -2.659854000

1 5.853361000 2.286800000 -1.868732000

1 3.845109000 1.040176000 -2.702826000

1 2.769230000 2.123488000 -1.797823000

1 -2.878627000 2.399366000 1.584298000

6 -3.804225000 -2.049034000 0.818246000

1 -3.463294000 -3.056662000 0.565160000

6 -5.122916000 -1.838214000 1.204610000

6 -4.688747000 0.521469000 1.485022000

1 -5.813832000 -2.687556000 1.240402000

1 -5.017260000 1.531330000 1.758182000

6 -5.578701000 -0.540216000 1.548207000

1 -6.614392000 -0.379813000 1.862247000

III (III)
SCF (BP86/SDD/6-31G**) Energy 333 K = -2438.29999583
Thermal correction to Gibbs Free Energy= 0.968629
Lowest Frequency = 2.4348 cm^{-1}
Second Frequency = 17.1918 cm^{-1}
SCF (B3PW91-D3,C6H6/tzvp) Energy 333 K= -3578.07653592

26 -0.524406000 0.016241000 0.303001000

6 -0.390334000 -1.291474000 1.881689000

6 -0.633813000 -1.709153000 4.994286000

1 -0.437293000 -1.236179000 5.973758000

7 -2.406152000 -0.352427000 -0.024680000

6 -3.318555000 0.604950000 -0.147448000

6 -3.111033000 2.013331000 -0.233033000

6 -1.811489000 2.626768000 -0.431045000

7 -0.648328000 1.900841000 -0.353528000

6 0.529371000 2.568770000 -0.866791000

6 1.429282000 3.243845000 0.014746000

6 2.568007000 3.867049000 -0.534559000

6 2.828891000 3.846143000 -1.909962000

6 1.934870000 3.194648000 -2.764978000

6 0.780320000 2.555551000 -2.272456000

6 1.175193000 3.341274000 1.522351000

6 2.475481000 3.344196000 2.354269000

6 -0.183981000 1.912135000 -3.271773000

6 0.528463000 0.919617000 -4.215422000

6 -2.931182000 -1.700764000 -0.191612000

6 -3.715163000 -2.314973000 0.827062000

6 -4.193539000 -3.622574000 0.601577000

6 -3.924118000 -4.308593000 -0.585336000

6 -3.187101000 -3.675298000 -1.592348000

6 -2.690044000 -2.368988000 -1.427020000

6 -4.104654000 -1.608367000 2.127725000

6 -5.565728000 -1.097244000 2.074481000

6 -2.029071000 -1.660233000 -2.609198000

6 -1.130970000 -2.582556000 -3.456354000

6 -3.109998000 -0.995203000 -3.496176000

6 -3.929378000 -2.514102000 3.366529000

6 0.341641000 4.592304000 1.895337000

6 -0.934224000 2.985595000 -4.096196000

1 -1.286957000 -1.932303000 1.801786000

1 0.481615000 -1.974794000 1.833405000

1 -3.008896000 -4.198026000 -2.537292000

1 -4.301835000 -5.325842000 -0.733505000

1 -4.793446000 -4.108254000 1.378028000

1 2.127683000 3.188027000 -3.843787000

1 3.719338000 4.342154000 -2.311114000

1 3.262779000 4.388873000 0.130936000

1 -0.933863000 1.346596000 -2.692415000

1 -1.392331000 -0.854434000 -2.202645000

1 -0.584367000 -1.990082000 -4.209212000

1 -0.391648000 -3.110654000 -2.831079000

1 -1.714588000 -3.342531000 -4.005474000

1 -2.644124000 -0.453282000 -4.338073000

1 -3.793107000 -1.755538000 -3.915140000

1 -3.716769000 -0.274690000 -2.923111000

1 -0.204790000 0.427966000 -4.879179000

1 1.263962000 1.426926000 -4.864599000

1 1.062859000 0.138982000 -3.650052000

1 -1.651855000 2.510810000 -4.788777000

1 -1.493441000 3.675704000 -3.444438000

1 -0.227975000 3.582744000 -4.700845000

1 -3.439677000 -0.735923000 2.248323000

1	-5.829164000	-0.581217000	3.014462000
1	-5.734345000	-0.392328000	1.244038000
1	-6.267273000	-1.939856000	1.941879000
1	-4.068238000	-1.925802000	4.289957000
1	-4.676168000	-3.327193000	3.387166000
1	-2.928713000	-2.973351000	3.394862000
1	0.583196000	2.451572000	1.806669000
1	0.183458000	4.633970000	2.987657000
1	0.872761000	5.513855000	1.595095000
1	-0.645147000	4.593813000	1.408466000
1	2.239929000	3.247772000	3.427359000
1	3.150692000	2.517161000	2.074999000
1	3.035751000	4.288446000	2.233101000
1	-1.671297000	-2.078763000	5.005972000
1	0.033051000	-2.585377000	4.909740000
7	2.630520000	-1.177178000	-0.070532000
5	1.332753000	-0.641137000	-0.105478000
1	0.516508000	-0.966723000	-0.973229000
6	1.460838000	0.174103000	3.927440000
1	1.804216000	0.898176000	3.173073000
1	1.510949000	0.669691000	4.913396000
1	2.177802000	-0.665737000	3.944870000
6	-1.520240000	0.993103000	3.780324000
1	-1.375576000	1.495129000	4.753674000
1	-2.570199000	0.655036000	3.738392000
14	-0.301553000	-0.459868000	3.583426000
1	-1.385520000	1.749225000	2.987454000
1	1.115803000	0.365731000	0.629637000
6	3.657621000	-0.654997000	0.870386000
6	4.815108000	0.059259000	0.133658000
6	5.848050000	0.633700000	1.125218000
6	6.376318000	-0.446357000	2.087121000
6	5.217114000	-1.136755000	2.829359000
6	4.190795000	-1.731936000	1.843242000
1	3.138118000	0.105245000	1.481962000
1	4.397749000	0.860914000	-0.501304000
1	5.324616000	-0.656909000	-0.541144000
1	6.680394000	1.101956000	0.569140000

1	5.374316000	1.443247000	1.715402000
1	7.086187000	-0.004149000	2.809602000
1	6.944562000	-1.204073000	1.510909000
1	5.599665000	-1.930752000	3.496072000
1	4.709174000	-0.398216000	3.480493000
1	3.341692000	-2.183790000	2.387200000
1	4.677009000	-2.548329000	1.274105000
6	3.064661000	-2.280256000	-0.971586000
6	2.981234000	-1.910514000	-2.466885000
6	3.516187000	-3.054590000	-3.351953000
6	2.778482000	-4.378359000	-3.073025000
6	2.830462000	-4.744020000	-1.577162000
6	2.306367000	-3.596037000	-0.690459000
1	4.132827000	-2.465584000	-0.749080000
1	3.540036000	-0.975215000	-2.649022000
1	1.924639000	-1.707749000	-2.725234000
1	3.427137000	-2.779083000	-4.418380000
1	4.598781000	-3.191993000	-3.155415000
1	3.208681000	-5.193888000	-3.682582000
1	1.720791000	-4.276591000	-3.386307000
1	2.251329000	-5.664853000	-1.382874000
1	3.878660000	-4.970418000	-1.295171000
1	2.399555000	-3.855221000	0.379446000
1	1.227999000	-3.432169000	-0.881871000
1	-4.363193000	0.275225000	-0.258297000
6	-1.821977000	4.031064000	-0.748392000
1	-0.868529000	4.520524000	-0.958110000
6	-2.991352000	4.780319000	-0.765402000
6	-4.291091000	2.818389000	-0.273244000
1	-2.931084000	5.852572000	-0.983691000
1	-5.253935000	2.316675000	-0.118053000
6	-4.251066000	4.182071000	-0.511953000
1	-5.168605000	4.777032000	-0.529595000

TS(III-IV) (V)
SCF (BP86/SDD/6-31G**) Energy 333 K = -2438.26038403
Thermal correction to Gibbs Free Energy= 0.969991
Lowest Frequency = -193.1683 cm^{-1}
Second Frequency = 19.5668 cm^{-1}
SCF (B3PW91-D3,C6H6/tzvp) Energy 333 K= -3578.06471491

26	0.051573000	-0.290588000	0.481634000

6	1.129246000	-1.074946000	2.056621000
6	1.641888000	-3.259711000	4.158110000
1	1.793355000	-3.509159000	5.223743000
7	1.154665000	-1.169923000	-1.153651000
6	0.766484000	-2.398534000	-1.444938000
6	-0.455199000	-3.069411000	-1.090333000
6	-1.657380000	-2.423678000	-0.586856000
7	-1.677681000	-1.102726000	-0.204813000
6	-2.971021000	-0.465246000	-0.189649000
6	-3.664733000	-0.222506000	1.036725000
6	-4.922511000	0.413460000	0.985605000
6	-5.505543000	0.798565000	-0.226380000
6	-4.825449000	0.546441000	-1.422650000
6	-3.563838000	-0.079199000	-1.435815000
6	-3.129218000	-0.688917000	2.393202000
6	-3.414930000	0.319627000	3.526348000
6	-2.906764000	-0.361719000	-2.791266000
6	-2.820810000	0.898189000	-3.680459000
6	2.429728000	-0.790339000	-1.723569000
6	3.647366000	-1.375858000	-1.264733000
6	4.855458000	-0.935768000	-1.845681000
6	4.880119000	0.037911000	-2.847626000
6	3.673746000	0.581164000	-3.307593000
6	2.438276000	0.178815000	-2.769150000
6	3.717140000	-2.471368000	-0.196389000
6	4.120715000	-3.830582000	-0.816638000
6	1.133690000	0.667697000	-3.394575000
6	1.188946000	2.122104000	-3.901981000
6	0.733033000	-0.286834000	-4.546965000
6	4.685359000	-2.107099000	0.952201000
6	-3.700214000	-2.076678000	2.776707000
6	-3.655816000	-1.485533000	-3.547899000
1	0.809465000	-2.067667000	1.645804000
1	2.163004000	-0.915586000	1.690686000
1	3.689654000	1.319475000	-4.115414000
1	5.832645000	0.363266000	-3.278843000
1	5.797629000	-1.375910000	-1.501300000
1	-5.286204000	0.834590000	-2.374135000

1	-6.486738000	1.285021000	-0.238263000
1	-5.462905000	0.595850000	1.919720000
1	-1.876505000	-0.707261000	-2.599398000
1	0.349248000	0.602590000	-2.617386000
1	0.184942000	2.448373000	-4.222729000
1	1.541652000	2.816496000	-3.120811000
1	1.855053000	2.229833000	-4.776241000
1	-0.213562000	0.035571000	-5.013524000
1	1.513466000	-0.295723000	-5.328449000
1	0.602482000	-1.321952000	-4.189919000
1	-2.340178000	0.652219000	-4.643307000
1	-3.819640000	1.306365000	-3.914120000
1	-2.236376000	1.698817000	-3.199156000
1	-3.148936000	-1.712743000	-4.502343000
1	-3.700797000	-2.412432000	-2.954649000
1	-4.691205000	-1.178812000	-3.781645000
1	2.714541000	-2.588013000	0.247751000
1	4.140479000	-4.618721000	-0.043784000
1	3.419867000	-4.146163000	-1.608208000
1	5.126176000	-3.776527000	-1.270120000
1	4.636391000	-2.869779000	1.748512000
1	5.732555000	-2.060168000	0.605466000
1	4.436264000	-1.130292000	1.398718000
1	-2.032975000	-0.795565000	2.302797000
1	-3.315011000	-2.390551000	3.763280000
1	-4.802817000	-2.040753000	2.841877000
1	-3.425381000	-2.851998000	2.044363000
1	-2.881159000	0.018761000	4.442394000
1	-3.086536000	1.337906000	3.260426000
1	-4.489810000	0.362554000	3.777140000
1	0.791671000	-3.863037000	3.792971000
1	2.541499000	-3.587367000	3.607927000
7	0.364556000	1.990697000	0.910610000
5	0.338730000	1.252786000	2.198157000
1	1.245623000	1.379139000	2.975543000
6	2.836246000	-0.464752000	4.611033000
1	2.709240000	0.629235000	4.559675000
1	3.003402000	-0.735170000	5.668950000

1	3.752716000	-0.726040000	4.053422000
6	-0.196786000	-0.953427000	4.977442000
1	-0.008773000	-1.202359000	6.037081000
1	-1.082848000	-1.525407000	4.652684000
14	1.320512000	-1.390886000	3.921638000
1	-0.440119000	0.119006000	4.913512000
1	-0.765532000	1.023335000	2.627586000
6	-0.953604000	2.629131000	0.522917000
6	-0.962648000	3.266891000	-0.878936000
6	-2.371945000	3.780831000	-1.247605000
6	-2.915369000	4.768341000	-0.199513000
6	-2.909459000	4.126043000	1.198843000
6	-1.498432000	3.633017000	1.575753000
1	-1.685038000	1.794832000	0.501447000
1	-0.614554000	2.532438000	-1.628338000
1	-0.257384000	4.119553000	-0.913658000
1	-2.342712000	4.252221000	-2.247241000
1	-3.065729000	2.921721000	-1.321369000
1	-3.935706000	5.092445000	-0.474274000
1	-2.285848000	5.680986000	-0.187818000
1	-3.270627000	4.841757000	1.959864000
1	-3.612975000	3.270146000	1.207843000
1	-1.508338000	3.146691000	2.566442000
1	-0.823806000	4.504181000	1.655943000
6	1.617704000	2.697343000	0.481973000
6	2.867888000	1.815520000	0.671513000
6	4.141997000	2.514182000	0.158165000
6	4.365810000	3.862253000	0.864255000
6	3.126766000	4.759333000	0.709095000
6	1.839209000	4.058361000	1.191740000
1	1.525372000	2.892023000	-0.604411000
1	2.728369000	0.859459000	0.141426000
1	2.989419000	1.579394000	1.744013000
1	5.010368000	1.846918000	0.302144000
1	4.056636000	2.676721000	-0.934155000
1	5.262625000	4.369192000	0.463085000
1	4.557260000	3.684679000	1.941283000
1	3.258571000	5.709878000	1.257802000
1	3.010138000	5.031450000	-0.359567000
1	0.982637000	4.729829000	1.017322000
1	1.889315000	3.878179000	2.283296000
1	1.448379000	-3.004771000	-2.067749000
6	-2.841247000	-3.237977000	-0.544277000
1	-3.774208000	-2.768161000	-0.224560000
6	-2.826838000	-4.590517000	-0.859786000
6	-0.493138000	-4.459338000	-1.421366000
1	-3.754993000	-5.166608000	-0.772416000
1	0.429206000	-4.915439000	-1.800870000
6	-1.638391000	-5.228917000	-1.291404000
1	-1.631068000	-6.293704000	-1.540289000

TS(III-IV) (III)
SCF (BP86/SDD/6-31G**) Energy 333 K = -2438.25703914
Thermal correction to Gibbs Free Energy= 0.974607
Lowest Frequency = -157.3976 cm^{-1}
Second Frequency = 20.2567 cm^{-1}
SCF (B3PW91-D3,C6H6/tzvp) Energy 333 K= -3578.04399483

26	0.055145000	-0.402975000	0.418850000
6	1.629687000	-0.473656000	1.752172000
6	2.946541000	-1.481540000	4.341049000
1	3.173842000	-1.267075000	5.401051000
7	0.855575000	-1.772051000	-0.717156000
6	0.339444000	-2.991759000	-0.798422000
6	-0.924992000	-3.435408000	-0.305900000
6	-1.968010000	-2.531865000	0.133653000
7	-1.747653000	-1.179077000	0.273944000
6	-2.945475000	-0.369146000	0.300807000
6	-3.480376000	0.111865000	1.534610000
6	-4.665649000	0.873814000	1.502311000
6	-5.324451000	1.162551000	0.301578000
6	-4.798407000	0.678025000	-0.900593000
6	-3.620281000	-0.093540000	-0.930237000
6	-2.860063000	-0.229544000	2.892394000
6	-2.823336000	0.984118000	3.845344000
6	-3.154870000	-0.657489000	-2.277064000
6	-2.969601000	0.443722000	-3.342763000
6	2.124085000	-1.602499000	-1.408954000
6	3.313458000	-2.231420000	-0.934995000
6	4.511548000	-2.000124000	-1.644098000

6	4.548462000	-1.199617000	-2.787810000
6	3.361294000	-0.638062000	-3.272405000
6	2.135473000	-0.832969000	-2.609952000
6	3.361905000	-3.199459000	0.252290000
6	3.493054000	-4.663473000	-0.236535000
6	0.837267000	-0.346083000	-3.252294000
6	1.004741000	0.915815000	-4.119915000
6	0.228935000	-1.491511000	-4.100198000
6	4.515062000	-2.883237000	1.230018000
6	-3.603774000	-1.405456000	3.572414000
6	-4.136360000	-1.735832000	-2.796131000
1	1.297490000	-1.533744000	1.891490000
1	2.549313000	-0.532542000	1.141572000
1	3.383797000	-0.046561000	-4.192111000
1	5.493895000	-1.031716000	-3.314295000
1	5.434552000	-2.469439000	-1.288424000
1	-5.322069000	0.886813000	-1.840318000
1	-6.247203000	1.752526000	0.305292000
1	-5.089847000	1.236274000	2.444298000
1	-2.174835000	-1.139398000	-2.121448000
1	0.129074000	-0.106785000	-2.436882000
1	0.015480000	1.282987000	-4.441387000
1	1.511881000	1.726728000	-3.571088000
1	1.582265000	0.712240000	-5.039297000
1	-0.713501000	-1.168579000	-4.574590000
1	0.928966000	-1.789092000	-4.901148000
1	0.013832000	-2.383649000	-3.489746000
1	-2.645501000	0.000691000	-4.300800000
1	-3.910417000	0.986851000	-3.540438000
1	-2.212363000	1.182453000	-3.033808000
1	-3.766601000	-2.170707000	-3.741532000
1	-4.258596000	-2.552957000	-2.067647000
1	-5.132769000	-1.301242000	-2.993238000
1	2.416163000	-3.108731000	0.812861000
1	3.501603000	-5.357641000	0.621898000
1	2.666098000	-4.958750000	-0.903759000
1	4.433953000	-4.805607000	-0.796798000
1	4.438932000	-3.524160000	2.124672000

1	5.502381000	-3.075023000	0.774816000
1	4.491683000	-1.832026000	1.557804000
1	-1.820230000	-0.553543000	2.708418000
1	-3.141524000	-1.638407000	4.548155000
1	-4.662866000	-1.147613000	3.753961000
1	-3.575185000	-2.318849000	2.957351000
1	-2.281129000	0.726287000	4.769738000
1	-2.318188000	1.849564000	3.384425000
1	-3.837617000	1.304388000	4.142226000
1	2.284974000	-2.365779000	4.317862000
1	3.893214000	-1.759235000	3.849103000
7	0.506658000	2.147813000	-0.061699000
5	0.499250000	1.429314000	1.238478000
1	0.914414000	1.965471000	2.236127000
6	3.400615000	1.444626000	3.599813000
1	2.995192000	2.409360000	3.254098000
1	3.747035000	1.578797000	4.640309000
1	4.288713000	1.209865000	2.986847000
6	0.641330000	0.450341000	4.652833000
1	0.994973000	0.610653000	5.687024000
1	-0.076729000	-0.387905000	4.675867000
14	2.116030000	0.038496000	3.528025000
1	0.100665000	1.353838000	4.333287000
1	-0.613000000	0.807097000	1.428556000
6	-0.818382000	2.767719000	-0.429408000
6	-0.809011000	3.506011000	-1.784569000
6	-2.206561000	4.057164000	-2.142084000
6	-2.751887000	4.983802000	-1.041167000
6	-2.785515000	4.248009000	0.309601000
6	-1.395440000	3.695177000	0.678879000
1	-1.530039000	1.924023000	-0.526086000
1	-0.448553000	2.828463000	-2.579548000
1	-0.103496000	4.358235000	-1.749114000
1	-2.156068000	4.587830000	-3.110759000
1	-2.911654000	3.215443000	-2.279059000
1	-3.760389000	5.347553000	-1.311058000
1	-2.104292000	5.880104000	-0.959181000
1	-3.145261000	4.919672000	1.110847000

1	-3.508212000	3.411291000	0.251628000
1	-1.452643000	3.131410000	1.627804000
1	-0.704017000	4.538154000	0.860126000
6	1.738585000	2.893182000	-0.445982000
6	2.986675000	1.999456000	-0.297168000
6	4.262882000	2.705195000	-0.793372000
6	4.494328000	4.034790000	-0.053635000
6	3.257491000	4.941434000	-0.173600000
6	1.971901000	4.233587000	0.304640000
1	1.650378000	3.133792000	-1.524400000
1	2.829516000	1.057237000	-0.848054000
1	3.115845000	1.734301000	0.768870000
1	5.131642000	2.033266000	-0.671334000
1	4.172955000	2.902405000	-1.880804000
1	5.390631000	4.549381000	-0.446479000
1	4.692326000	3.826014000	1.016458000
1	3.402915000	5.877851000	0.395888000
1	3.131267000	5.240112000	-1.234239000
1	1.113336000	4.910633000	0.158988000
1	2.033940000	4.028113000	1.390675000
1	0.930207000	-3.744294000	-1.342594000
6	-3.250194000	-3.126173000	0.392188000
1	-4.075457000	-2.472287000	0.681779000
6	-3.458899000	-4.498555000	0.317848000
6	-1.186698000	-4.835860000	-0.395435000
1	-4.449269000	-4.898258000	0.563127000
1	-0.372624000	-5.485722000	-0.738415000
6	-2.420015000	-5.379212000	-0.071632000
1	-2.592800000	-6.457290000	-0.134649000

IV (V)
SCF (BP86/SDD/6-31G**) Energy 333 K = -2438.29423939
Thermal correction to Gibbs Free Energy= 0.973447
Lowest Frequency = 24.9617 cm^{-1}
Second Frequency = 28.1818 cm^{-1}
SCF (B3PW91-D3,C6H6/tzvp) Energy 333 K= -3578.10363021

26	0.033353000	-0.036959000	0.092739000
6	1.021075000	0.510724000	2.201395000
6	1.841689000	-0.885450000	4.857610000
1	2.145385000	-0.760991000	5.912361000
7	1.170419000	-1.628652000	-0.517217000
6	0.816804000	-2.857002000	-0.160042000
6	-0.410994000	-3.296695000	0.429928000
6	-1.619975000	-2.497025000	0.541605000
7	-1.637138000	-1.149177000	0.264767000
6	-2.944700000	-0.570686000	0.044988000
6	-3.628478000	0.126161000	1.089145000
6	-4.907886000	0.653051000	0.816333000
6	-5.515177000	0.507297000	-0.435767000
6	-4.838655000	-0.179510000	-1.449411000
6	-3.558635000	-0.727906000	-1.237729000
6	-3.052085000	0.275854000	2.500431000
6	-3.362691000	1.652116000	3.126892000
6	-2.903765000	-1.515895000	-2.376286000
6	-2.869676000	-0.720982000	-3.700270000
6	2.435301000	-1.537156000	-1.214190000
6	3.662665000	-1.790372000	-0.534937000
6	4.859719000	-1.696428000	-1.274569000
6	4.861177000	-1.358988000	-2.631191000
6	3.645672000	-1.113627000	-3.282318000
6	2.419138000	-1.199983000	-2.598702000
6	3.747071000	-2.155549000	0.950196000
6	4.189056000	-3.625298000	1.145886000
6	1.104716000	-1.050881000	-3.363717000
6	1.141356000	0.034004000	-4.458239000
6	0.694854000	-2.418340000	-3.962822000
6	4.694529000	-1.209355000	1.722674000
6	-3.552415000	-0.844961000	3.444984000
6	-3.614900000	-2.873478000	-2.593772000
1	0.335753000	-0.373389000	2.256544000
1	1.977849000	0.155644000	1.770823000
1	3.646752000	-0.865663000	-4.348407000
1	5.805217000	-1.290782000	-3.181768000
1	5.811247000	-1.892241000	-0.768407000
1	-5.316515000	-0.304250000	-2.427473000
1	-6.511926000	0.922947000	-0.618128000
1	-5.443043000	1.181950000	1.611059000
1	-1.860684000	-1.725283000	-2.081341000
1	0.324444000	-0.763246000	-2.632741000

1	0.133319000	0.172928000	-4.884290000
1	1.481823000	1.004320000	-4.059112000
1	1.808831000	-0.242088000	-5.293456000
1	-0.266359000	-2.339947000	-4.499638000
1	1.458293000	-2.770463000	-4.679113000
1	0.587475000	-3.186722000	-3.179261000
1	-2.352140000	-1.303711000	-4.482390000
1	-3.885348000	-0.507162000	-4.076710000
1	-2.347163000	0.242766000	-3.585616000
1	-3.110915000	-3.451497000	-3.388444000
1	-3.614947000	-3.481052000	-1.674862000
1	-4.664768000	-2.721147000	-2.902352000
1	2.741104000	-2.044676000	1.390348000
1	4.219333000	-3.881293000	2.219222000
1	3.504462000	-4.331528000	0.646332000
1	5.198018000	-3.793842000	0.729918000
1	4.674098000	-1.444474000	2.800878000
1	5.740196000	-1.311877000	1.383845000
1	4.402555000	-0.153703000	1.596807000
1	-1.955182000	0.186839000	2.419175000
1	-3.134073000	-0.704460000	4.457557000
1	-4.654023000	-0.820657000	3.530546000
1	-3.259208000	-1.846467000	3.093483000
1	-2.795631000	1.774424000	4.064287000
1	-3.081888000	2.479362000	2.455543000
1	-4.433104000	1.757769000	3.379254000
1	0.950663000	-1.537756000	4.843516000
1	2.655403000	-1.422878000	4.339648000
7	0.282964000	1.997397000	-0.034215000
5	0.388190000	1.950345000	1.596143000
1	1.072723000	2.894036000	1.980427000
6	3.045995000	1.874243000	4.142403000
1	2.884652000	2.851198000	3.657596000
1	3.324290000	2.058195000	5.195216000
1	3.906330000	1.387301000	3.651350000
6	0.080590000	1.638463000	5.009330000
1	0.388664000	1.828990000	6.052691000
1	-0.820534000	1.001949000	5.037949000

14	1.480039000	0.800109000	4.039927000
1	-0.198198000	2.599600000	4.548196000
1	-0.747841000	2.058821000	2.022978000
6	-1.036299000	2.574671000	-0.518169000
6	-1.168193000	2.600178000	-2.055546000
6	-2.602463000	2.973761000	-2.487297000
6	-3.054130000	4.309268000	-1.867598000
6	-2.912825000	4.274877000	-0.334901000
6	-1.466876000	3.939647000	0.086248000
1	-1.795182000	1.855256000	-0.146704000
1	-0.889462000	1.611630000	-2.471685000
1	-0.462727000	3.334309000	-2.489671000
1	-2.659395000	3.019855000	-3.590736000
1	-3.297721000	2.174325000	-2.166651000
1	-4.097771000	4.529752000	-2.157876000
1	-2.432886000	5.133320000	-2.273186000
1	-3.214095000	5.243451000	0.104616000
1	-3.604232000	3.512055000	0.075132000
1	-1.377539000	3.893939000	1.183338000
1	-0.804509000	4.752927000	-0.255042000
6	1.507595000	2.514223000	-0.761306000
6	2.795905000	1.840118000	-0.247797000
6	4.039604000	2.271380000	-1.048723000
6	4.231360000	3.795592000	-1.001809000
6	2.957184000	4.501296000	-1.490967000
6	1.700470000	4.052362000	-0.713813000
1	1.391954000	2.230793000	-1.828034000
1	2.697430000	0.743445000	-0.299289000
1	2.933874000	2.107184000	0.815915000
1	4.929716000	1.749715000	-0.653628000
1	3.931670000	1.942897000	-2.101295000
1	5.102474000	4.100269000	-1.610681000
1	4.447189000	4.106071000	0.039751000
1	3.062018000	5.599091000	-1.413933000
1	2.814552000	4.278577000	-2.568174000
1	0.829268000	4.565506000	-1.148513000
1	1.768940000	4.368042000	0.343815000
1	1.538111000	-3.661968000	-0.379932000

6	-2.812563000	-3.205775000	0.919734000
1	-3.751672000	-2.649607000	0.955452000
6	-2.801220000	-4.550260000	1.267517000
6	-0.449242000	-4.681120000	0.787969000
1	-3.737625000	-5.026086000	1.579610000
1	0.481305000	-5.254021000	0.695510000
6	-1.604939000	-5.308151000	1.222460000
1	-1.599911000	-6.366311000	1.498553000

TS(IV-V) (V)
SCF (BP86/SDD/6-31G**) Energy 333 K = -2438.29276622
Thermal correction to Gibbs Free Energy= 0.971244
Lowest Frequency = -56.4182 cm^{-1}
Second Frequency = 9.2148 cm^{-1}
SCF (B3PW91-D3,C6H6/tzvp) Energy 333 K= -3578.09897428

6	1.731601000	1.475885000	1.689573000
5	0.465532000	2.136823000	0.855678000
7	0.437686000	1.858758000	-0.734417000
6	-0.829639000	2.388892000	-1.364982000
26	-0.001404000	-0.028158000	0.075033000
7	0.971438000	-1.844855000	0.075276000
6	0.568410000	-2.775491000	0.933546000
6	-0.657542000	-2.849271000	1.668986000
6	-1.813130000	-1.993004000	1.455716000
7	-1.749835000	-0.872379000	0.665415000
6	-3.016266000	-0.289868000	0.275015000
6	-3.630841000	0.749556000	1.040475000
6	-4.854523000	1.284959000	0.586060000
6	-5.483468000	0.817051000	-0.571080000
6	-4.890589000	-0.222580000	-1.296354000
6	-3.667689000	-0.794246000	-0.896432000
6	-3.079790000	1.260643000	2.376002000
6	-2.966383000	2.801043000	2.406308000
6	-3.125578000	-1.990568000	-1.683942000
6	-3.159776000	-1.776239000	-3.212736000
6	2.184274000	-2.174658000	-0.647428000
6	3.445972000	-2.236482000	0.010552000
6	4.578137000	-2.579038000	-0.758813000
6	4.485451000	-2.849682000	-2.126494000
6	3.235720000	-2.791457000	-2.755971000
6	2.071643000	-2.460316000	-2.039752000
6	3.640892000	-1.973258000	1.506252000
6	3.963864000	-3.280572000	2.269381000
6	0.710207000	-2.510462000	-2.732265000
6	0.737437000	-2.022168000	-4.194437000
6	0.132498000	-3.943923000	-2.645563000
6	4.744114000	-0.924693000	1.770601000
6	-3.950881000	0.780179000	3.563139000
6	-3.900796000	-3.281230000	-1.320551000
1	1.651792000	0.378139000	1.871254000
1	2.706920000	1.605668000	1.183457000
1	3.159683000	-3.018683000	-3.823807000
1	5.380694000	-3.113151000	-2.699463000
1	5.554614000	-2.634192000	-0.265861000
1	-5.393363000	-0.610395000	-2.188779000
1	-6.436118000	1.247897000	-0.897261000
1	-5.332834000	2.077325000	1.171870000
1	-2.073170000	-2.136477000	-1.383367000
1	0.028647000	-1.843519000	-2.170469000
1	-0.290575000	-1.960863000	-4.589213000
1	1.197978000	-1.023869000	-4.282950000
1	1.294636000	-2.712423000	-4.851955000
1	-0.864392000	-3.995720000	-3.116887000
1	0.793921000	-4.658375000	-3.167049000
1	0.033066000	-4.277498000	-1.599325000
1	-2.690797000	-2.633344000	-3.726906000
1	-4.193073000	-1.702513000	-3.594763000
1	-2.625878000	-0.860176000	-3.513736000
1	-3.482264000	-4.150053000	-1.859015000
1	-3.854208000	-3.492047000	-0.240352000
1	-4.964661000	-3.187754000	-1.603892000
1	2.697454000	-1.569024000	1.912113000
1	4.074847000	-3.081105000	3.349411000
1	3.174905000	-4.041180000	2.142496000
1	4.908879000	-3.723712000	1.908872000
1	4.808561000	-0.703679000	2.848890000
1	5.735934000	-1.284853000	1.446354000
1	4.536658000	0.019763000	1.242022000
1	-2.067429000	0.842437000	2.507268000

1	-3.527788000	1.144777000	4.515569000
1	-4.982358000	1.167724000	3.482722000
1	-4.002209000	-0.318830000	3.616608000
1	-2.563934000	3.132230000	3.379108000
1	-2.293223000	3.173318000	1.618221000
1	-3.950922000	3.285693000	2.283213000
1	0.275904000	3.321054000	1.106006000
14	1.923931000	2.268669000	3.414447000
6	3.298796000	1.394254000	4.409131000
1	3.402048000	1.842533000	5.413529000
1	3.078690000	0.320965000	4.548248000
1	4.278890000	1.475362000	3.907940000
6	0.314712000	2.128677000	4.414603000
1	0.436440000	2.551613000	5.427365000
1	0.000402000	1.076091000	4.526901000
6	2.407845000	4.100791000	3.239651000
1	1.649481000	4.658545000	2.665869000
1	2.507065000	4.578725000	4.230244000
1	3.374996000	4.213512000	2.718730000
1	-0.502771000	2.672489000	3.914413000
1	-0.580825000	1.552873000	1.227089000
6	-1.181364000	1.652561000	-2.676386000
6	-2.533136000	2.120005000	-3.255277000
6	-2.572875000	3.647370000	-3.446890000
6	-2.232606000	4.371126000	-2.131925000
6	-0.862006000	3.920782000	-1.585569000
1	-1.629840000	2.155905000	-0.635653000
1	-1.205263000	0.561303000	-2.484405000
1	-0.387482000	1.821510000	-3.430648000
1	-2.727469000	1.605588000	-4.214656000
1	-3.344581000	1.816769000	-2.566718000
1	-3.566815000	3.959784000	-3.816298000
1	-1.840223000	3.942457000	-4.225082000
1	-2.235622000	5.466763000	-2.278776000
1	-3.016891000	4.151108000	-1.381039000
1	-0.633287000	4.434384000	-0.636321000
1	-0.081243000	4.211816000	-2.315265000
6	1.659175000	2.223550000	-1.542741000
6	2.625055000	1.042377000	-1.738144000
6	3.795885000	1.410654000	-2.672409000
6	4.546428000	2.660069000	-2.176418000
6	3.576685000	3.840097000	-1.981919000
6	2.419876000	3.471818000	-1.029375000
1	1.295223000	2.480199000	-2.557616000
1	2.070149000	0.182874000	-2.153188000
1	3.024781000	0.713887000	-0.760951000
1	4.481051000	0.549380000	-2.764880000
1	3.399879000	1.608911000	-3.689284000
1	5.351934000	2.932070000	-2.883149000
1	5.038948000	2.430922000	-1.210264000
1	4.113922000	4.724412000	-1.593091000
1	3.163263000	4.135560000	-2.967838000
1	1.728228000	4.322625000	-0.919567000
1	2.827562000	3.274819000	-0.022787000
1	1.235600000	-3.643763000	1.067057000
6	-3.033292000	-2.407602000	2.092413000
1	-3.936905000	-1.825918000	1.898408000
6	-3.088037000	-3.491683000	2.958653000
6	-0.762476000	-3.959586000	2.563976000
1	-4.040564000	-3.739681000	3.440270000
1	0.129480000	-4.581219000	2.707875000
6	-1.938566000	-4.276637000	3.223621000
1	-1.985927000	-5.127557000	3.908895000

V (V)
SCF (BP86/SDD/6-31G**) Energy 333 K = -2438.30333854
Thermal correction to Gibbs Free Energy= 0.968477
Lowest Frequency = 10.3523 cm^{-1}
Second Frequency = 17.6640 cm^{-1}
SCF (B3PW91-D3,C6H6/tzvp) Energy 333 K= -3578.10446746

26	0.048008000	-0.238013000	0.048888000
6	1.402057000	3.278849000	0.948433000
6	2.954188000	4.620057000	3.296792000
1	2.925765000	4.954275000	4.349118000
7	1.175316000	-1.896772000	0.466183000
6	0.758200000	-2.798378000	1.346154000
6	-0.539182000	-2.950032000	1.927134000
6	-1.716308000	-2.196846000	1.537731000
7	-1.641602000	-1.113236000	0.694828000

6	-2.901452000	-0.622676000	0.186188000
6	-3.609914000	0.416272000	0.864717000
6	-4.840234000	0.847131000	0.327106000
6	-5.374984000	0.280065000	-0.835498000
6	-4.678092000	-0.746569000	-1.484302000
6	-3.445081000	-1.219127000	-0.994717000
6	-3.116819000	1.031358000	2.178057000
6	-3.236646000	2.570247000	2.191775000
6	-2.769509000	-2.402193000	-1.694531000
6	-2.661690000	-2.221520000	-3.224002000
6	2.520572000	-2.135061000	-0.014436000
6	3.639489000	-1.832026000	0.809556000
6	4.924593000	-2.118391000	0.304833000
6	5.105220000	-2.674226000	-0.966407000
6	3.989622000	-2.950184000	-1.768438000
6	2.683426000	-2.688266000	-1.316191000
6	3.499522000	-1.218690000	2.204976000
6	3.873452000	-2.238317000	3.306616000
6	1.473336000	-3.045708000	-2.179708000
6	1.678173000	-2.741950000	-3.677693000
6	1.075093000	-4.526332000	-1.971021000
6	4.338257000	0.069852000	2.356685000
6	-3.871947000	0.450156000	3.399311000
6	-3.506137000	-3.725631000	-1.372434000
1	2.469180000	3.128958000	0.693300000
1	1.089619000	4.181751000	0.384158000
1	4.135181000	-3.385265000	-2.762413000
1	6.113703000	-2.890713000	-1.334287000
1	5.799768000	-1.899993000	0.926558000
1	-5.105778000	-1.203276000	-2.383696000
1	-6.335254000	0.630209000	-1.228985000
1	-5.396841000	1.636694000	0.842881000
1	-1.744456000	-2.483140000	-1.290574000
1	0.627075000	-2.425105000	-1.827352000
1	0.735545000	-2.903459000	-4.228130000
1	1.995772000	-1.698246000	-3.840756000
1	2.436358000	-3.401783000	-4.134817000
1	0.180024000	-4.778131000	-2.566610000

1	1.894080000	-5.197332000	-2.285618000
1	0.851456000	-4.739882000	-0.912659000
1	-2.100399000	-3.063171000	-3.666592000
1	-3.654422000	-2.205857000	-3.707257000
1	-2.145246000	-1.285553000	-3.492308000
1	-2.992319000	-4.580405000	-1.846880000
1	-3.550155000	-3.911119000	-0.287511000
1	-4.541727000	-3.698737000	-1.756711000
1	2.441996000	-0.937134000	2.347492000
1	3.741975000	-1.792584000	4.307863000
1	3.249052000	-3.146224000	3.253822000
1	4.927532000	-2.554599000	3.213202000
1	4.171404000	0.517475000	3.351659000
1	5.420184000	-0.129911000	2.262893000
1	4.063008000	0.817875000	1.595549000
1	-2.048278000	0.773395000	2.281185000
1	-3.502349000	0.915082000	4.330268000
1	-4.954765000	0.658544000	3.326005000
1	-3.737618000	-0.638783000	3.488902000
1	-2.767763000	2.973721000	3.105385000
1	-2.735834000	3.025116000	1.322337000
1	-4.290607000	2.900957000	2.196086000
1	3.815308000	3.937187000	3.187976000
1	3.154976000	5.507705000	2.671130000
7	0.295736000	1.577872000	-1.030195000
5	0.466796000	2.015579000	0.513752000
1	-0.674659000	2.101170000	0.947301000
6	-0.095819000	5.009204000	3.067649000
1	-1.062520000	4.595177000	2.734571000
1	-0.194473000	5.276298000	4.134637000
1	0.077470000	5.943864000	2.505407000
6	1.055428000	2.263226000	3.921084000
1	1.019700000	2.561185000	4.983915000
1	1.868201000	1.524958000	3.806810000
14	1.323311000	3.769289000	2.786857000
1	0.108374000	1.751142000	3.678928000
1	0.990574000	0.972319000	1.084682000
6	-1.023677000	2.020950000	-1.631643000

6	-1.227639000	1.560362000	-3.089580000
6	-2.661511000	1.859212000	-3.574652000
6	-3.007924000	3.351550000	-3.419741000
6	-2.793369000	3.815307000	-1.967310000
6	-1.353216000	3.530675000	-1.490310000
1	-1.792598000	1.502031000	-1.025516000
1	-1.013463000	0.477448000	-3.172048000
1	-0.515425000	2.076786000	-3.761124000
1	-2.770173000	1.543829000	-4.628864000
1	-3.378347000	1.255222000	-2.986265000
1	-4.051077000	3.536919000	-3.735252000
1	-2.363643000	3.950978000	-4.094364000
1	-3.014379000	4.893695000	-1.866459000
1	-3.507541000	3.282455000	-1.308856000
1	-1.230595000	3.833000000	-0.436687000
1	-0.650684000	4.143790000	-2.082066000
6	1.473395000	1.668757000	-1.967824000
6	2.724096000	0.998771000	-1.373951000
6	3.892593000	0.960890000	-2.378028000
6	4.250886000	2.371311000	-2.876104000
6	3.009322000	3.061144000	-3.465360000
6	1.835254000	3.094822000	-2.462983000
1	1.205729000	1.079267000	-2.868332000
1	2.484026000	-0.031077000	-1.062954000
1	3.041487000	1.538663000	-0.464497000
1	4.763886000	0.471108000	-1.908914000
1	3.611409000	0.329454000	-3.244935000
1	5.061940000	2.324838000	-3.625969000
1	4.636874000	2.972275000	-2.028807000
1	3.249416000	4.091012000	-3.787129000
1	2.693168000	2.516051000	-4.378178000
1	0.968198000	3.567173000	-2.951451000
1	2.098348000	3.725732000	-1.596146000
1	1.490238000	-3.568100000	1.643518000
6	-2.970008000	-2.673032000	2.055449000
1	-3.885514000	-2.172145000	1.734224000
6	-3.048771000	-3.724182000	2.960560000
6	-0.666175000	-4.023571000	2.862134000
1	-4.031929000	-4.025692000	3.339027000
1	0.246685000	-4.563483000	3.141342000
6	-1.886046000	-4.405921000	3.395770000
1	-1.952664000	-5.228539000	4.113212000

IV (III)
SCF (BP86/SDD/6-31G**) Energy 333 K = -2438.28413794
Thermal correction to Gibbs Free Energy= 0.974602
Lowest Frequency = 10.8126 cm^{-1}
Second Frequency = 19.1697 cm^{-1}
SCF (B3PW91-D3,C6H6/tzvp) Energy 333 K= -3578.07200982

26	-0.236957000	-0.091450000	0.298931000
6	1.414244000	1.303331000	2.051239000
6	3.708388000	1.631907000	4.146096000
1	4.323350000	2.337011000	4.733053000
7	0.172469000	-2.014515000	0.195073000
6	-0.610907000	-2.937168000	0.758826000
6	-1.900585000	-2.776621000	1.336825000
6	-2.662996000	-1.552950000	1.238552000
7	-2.117892000	-0.418516000	0.695965000
6	-3.056278000	0.611579000	0.306561000
6	-3.285135000	1.757257000	1.125221000
6	-4.151578000	2.760391000	0.647954000
6	-4.801074000	2.644155000	-0.587094000
6	-4.617258000	1.487087000	-1.352802000
6	-3.766404000	0.449407000	-0.924805000
6	-2.679483000	1.893401000	2.524798000
6	-2.147554000	3.312246000	2.815613000
6	-3.707499000	-0.841616000	-1.751336000
6	-3.279849000	-0.609499000	-3.214926000
6	1.400707000	-2.603328000	-0.317687000
6	2.420733000	-3.015648000	0.588754000
6	3.562159000	-3.650276000	0.057990000
6	3.702791000	-3.873496000	-1.315742000
6	2.686560000	-3.468683000	-2.190311000
6	1.518438000	-2.840527000	-1.716015000
6	2.315276000	-2.823640000	2.106208000
6	2.023836000	-4.163638000	2.824257000
6	0.363921000	-2.536305000	-2.670733000
6	0.820668000	-2.155124000	-4.093173000
6	-0.612237000	-3.737191000	-2.734952000

6	3.582261000	-2.178404000	2.711860000
6	-3.702768000	1.487509000	3.614885000
6	-5.069771000	-1.575966000	-1.709582000
1	0.609254000	1.150444000	2.801902000
1	1.941295000	0.335650000	1.990152000
1	2.791376000	-3.663437000	-3.262097000
1	4.598640000	-4.369050000	-1.704585000
1	4.353377000	-3.978942000	0.740347000
1	-5.158414000	1.375131000	-2.299517000
1	-5.466239000	3.440376000	-0.938209000
1	-4.329885000	3.647131000	1.265302000
1	-2.955163000	-1.504387000	-1.292832000
1	-0.194394000	-1.680244000	-2.245638000
1	-0.042473000	-1.806561000	-4.685496000
1	1.576262000	-1.351780000	-4.083589000
1	1.250619000	-3.018393000	-4.631404000
1	-1.458438000	-3.512387000	-3.407603000
1	-0.097118000	-4.634427000	-3.121876000
1	-1.023877000	-3.983032000	-1.742679000
1	-3.283377000	-1.564642000	-3.769685000
1	-3.965434000	0.077636000	-3.741852000
1	-2.264901000	-0.186193000	-3.275193000
1	-5.008321000	-2.533249000	-2.256874000
1	-5.377082000	-1.794622000	-0.674639000
1	-5.862856000	-0.968971000	-2.181705000
1	1.468221000	-2.142940000	2.302975000
1	1.939316000	-4.007526000	3.913867000
1	1.085838000	-4.625373000	2.475151000
1	2.838708000	-4.887894000	2.647732000
1	3.424436000	-1.966986000	3.783199000
1	4.458131000	-2.846305000	2.635664000
1	3.837489000	-1.229961000	2.212308000
1	-1.830757000	1.188876000	2.586277000
1	-3.253566000	1.589636000	4.618815000
1	-4.594654000	2.138588000	3.576137000
1	-4.035220000	0.444028000	3.497629000
1	-1.629330000	3.329735000	3.789295000
1	-1.436342000	3.644290000	2.042505000
1	-2.965738000	4.052089000	2.873490000
1	3.135803000	1.014821000	4.860582000
1	4.397377000	0.960508000	3.604783000
7	1.067900000	1.379481000	-0.744183000
5	0.690847000	1.851130000	0.688182000
1	0.363051000	3.023622000	0.780628000
6	3.609060000	3.640731000	1.797082000
1	2.982161000	4.148790000	1.044509000
1	4.124859000	4.421867000	2.383739000
1	4.384119000	3.065424000	1.263396000
6	1.466324000	3.743599000	3.980201000
1	2.090740000	4.473316000	4.525379000
1	0.865901000	3.194255000	4.726278000
14	2.548226000	2.562962000	2.953727000
1	0.769124000	4.309426000	3.339884000
1	-0.588919000	1.430506000	0.737448000
6	0.167127000	2.065607000	-1.776383000
6	0.233779000	1.443738000	-3.187423000
6	-0.809602000	2.078077000	-4.134530000
6	-0.675162000	3.608290000	-4.205215000
6	-0.774192000	4.211567000	-2.794065000
6	0.295624000	3.615569000	-1.857341000
1	-0.859417000	1.877088000	-1.404793000
1	0.067354000	0.353725000	-3.118291000
1	1.236380000	1.583293000	-3.632749000
1	-0.715275000	1.627339000	-5.139632000
1	-1.826574000	1.834267000	-3.774219000
1	-1.453525000	4.029621000	-4.867537000
1	0.302191000	3.878006000	-4.653860000
1	-0.662531000	5.310770000	-2.828789000
1	-1.782313000	4.009831000	-2.380732000
1	0.195517000	4.043832000	-0.848424000
1	1.290631000	3.911370000	-2.230359000
6	2.437960000	1.030010000	-1.263343000
6	3.323705000	0.313529000	-0.231305000
6	4.639733000	-0.188229000	-0.857071000
6	5.450592000	0.972532000	-1.454994000
6	4.589373000	1.754857000	-2.458068000

6	3.249051000	2.220554000	-1.847433000
1	2.274268000	0.310984000	-2.091846000
1	2.775202000	-0.534751000	0.203421000
1	3.563086000	1.011638000	0.589389000
1	5.229109000	-0.724624000	-0.091661000
1	4.410669000	-0.930404000	-1.645759000
1	6.371142000	0.600059000	-1.940906000
1	5.775661000	1.651806000	-0.641668000
1	5.137822000	2.632261000	-2.847155000
1	4.378268000	1.107664000	-3.333389000
1	2.681068000	2.737961000	-2.633855000
1	3.432786000	2.959417000	-1.045078000
1	-0.220441000	-3.966720000	0.767800000
6	-4.019626000	-1.603359000	1.710611000
1	-4.638229000	-0.708970000	1.608629000
6	-4.546498000	-2.741516000	2.307000000
6	-2.483634000	-3.930290000	1.945485000
1	-5.577701000	-2.718090000	2.677017000
1	-1.874289000	-4.840568000	2.002714000
6	-3.776168000	-3.925210000	2.443201000
1	-4.199970000	-4.816708000	2.914155000

TS(V-2b)(V)
SCF (BP86/SDD/6-31G**) Energy 333 K = -2438.27957876
Thermal correction to Gibbs Free Energy= 0.962269
Lowest Frequency = -72.9747 cm^{-1}
Second Frequency = 9.3342 cm^{-1}
SCF (B3PW91-D3,C6H6/tzvp) Energy 333 K= -3578.06297945

1	-1.045516000	-0.317863000	0.966492000
5	-1.766305000	-1.592616000	0.887729000
7	-1.910389000	-2.045713000	-0.525631000
26	0.325418000	0.395513000	0.380475000
7	0.371065000	2.363348000	-0.033716000
6	1.439702000	3.097696000	0.246055000
6	2.708086000	2.651131000	0.738466000
6	3.125510000	1.262866000	0.840289000
7	2.276291000	0.220275000	0.547227000
6	2.855978000	-1.085926000	0.356937000
6	2.730791000	-2.083924000	1.370816000
6	3.297278000	-3.351231000	1.133241000
6	3.974520000	-3.640477000	-0.058274000
6	4.089208000	-2.653940000	-1.044101000
6	3.538102000	-1.370463000	-0.866672000
6	2.047274000	-1.785752000	2.707397000
6	1.380535000	-3.019444000	3.345809000
6	3.719935000	-0.328456000	-1.975495000
6	3.135383000	-0.801295000	-3.325498000
6	-0.741141000	3.071951000	-0.630878000
6	-1.779159000	3.575437000	0.199873000
6	-2.830105000	4.282985000	-0.415462000
6	-2.860986000	4.484296000	-1.800966000
6	-1.835814000	3.966800000	-2.602268000
6	-0.762183000	3.246895000	-2.042672000
6	-1.735722000	3.415524000	1.719956000
6	-1.074825000	4.646415000	2.386228000
6	0.359067000	2.719923000	-2.941465000
6	-0.167937000	2.021174000	-4.213239000
6	1.343205000	3.853373000	-3.317694000
6	-3.123130000	3.152801000	2.339942000
6	3.027383000	-1.138072000	3.715413000
6	5.208190000	0.058122000	-2.145825000
1	-1.865218000	4.125799000	-3.685319000
1	-3.685364000	5.043154000	-2.256261000
1	-3.636122000	4.687903000	0.204441000
1	4.619671000	-2.881019000	-1.975775000
1	4.411172000	-4.632381000	-0.216224000
1	3.210164000	-4.126240000	1.900544000
1	3.169625000	0.580465000	-1.677601000
1	0.925084000	1.967968000	-2.361545000
1	0.669941000	1.566893000	-4.769387000
1	-0.890117000	1.224695000	-3.969522000
1	-0.665996000	2.730331000	-4.897248000
1	2.158587000	3.466659000	-3.953679000
1	0.823914000	4.651327000	-3.877454000
1	1.797464000	4.312755000	-2.423996000
1	3.259157000	-0.015415000	-4.091511000
1	3.648770000	-1.705705000	-3.696367000
1	2.061484000	-1.035623000	-3.244407000
1	5.318065000	0.838872000	-2.919000000

1	5.633215000	0.445896000	-1.206299000
1	5.810996000	-0.812210000	-2.460873000
1	-1.102039000	2.534956000	1.938582000
1	-1.025281000	4.515208000	3.481280000
1	-0.048629000	4.810253000	2.017729000
1	-1.657152000	5.561465000	2.177607000
1	-3.019452000	2.925849000	3.414532000
1	-3.785753000	4.032472000	2.259350000
1	-3.627313000	2.298974000	1.858123000
1	1.247055000	-1.047210000	2.501992000
1	2.507052000	-0.909686000	4.662238000
1	3.858656000	-1.829338000	3.943879000
1	3.460004000	-0.201475000	3.330291000
1	0.781317000	-2.709664000	4.218315000
1	0.708843000	-3.531248000	2.637676000
1	2.127057000	-3.747857000	3.710371000
6	-3.059903000	-1.123433000	1.767096000
1	-3.518336000	-0.177537000	1.418518000
1	-3.841723000	-1.897462000	1.615790000
1	-0.969464000	-2.303949000	1.480136000
14	-2.843258000	-1.051083000	3.651500000
6	-1.356321000	0.013457000	4.187754000
1	-1.255712000	0.036540000	5.287552000
1	-1.458204000	1.056260000	3.840518000
6	-4.412456000	-0.316971000	4.453769000
1	-4.332692000	-0.299080000	5.555315000
1	-4.593406000	0.718589000	4.115928000
1	-5.307495000	-0.910024000	4.195368000
6	-2.606300000	-2.813782000	4.332473000
1	-1.733217000	-3.304269000	3.870355000
1	-2.453933000	-2.808221000	5.426192000
1	-3.490234000	-3.441826000	4.123118000
1	-0.415409000	-0.386195000	3.772289000
6	-1.061793000	-3.150322000	-1.019060000
6	-0.134761000	-2.760993000	-2.196970000
6	0.768553000	-3.933558000	-2.630684000
6	-0.055835000	-5.187058000	-2.977283000
6	-0.962936000	-5.588508000	-1.800102000

6	-1.876886000	-4.422640000	-1.372998000
1	-0.404281000	-3.413680000	-0.170544000
1	0.483362000	-1.897043000	-1.885023000
1	-0.738501000	-2.430635000	-3.065708000
1	1.392639000	-3.630365000	-3.491320000
1	1.469997000	-4.171814000	-1.807875000
1	0.613530000	-6.022890000	-3.252525000
1	-0.683822000	-4.980558000	-3.867520000
1	-1.574177000	-6.471558000	-2.063193000
1	-0.330522000	-5.889831000	-0.941250000
1	-2.493047000	-4.707677000	-0.501222000
1	-2.577382000	-4.195854000	-2.201271000
6	-2.893302000	-1.519638000	-1.493431000
6	-2.816791000	0.015139000	-1.652875000
6	-3.763615000	0.524370000	-2.757754000
6	-5.215903000	0.071065000	-2.515383000
6	-5.297617000	-1.458075000	-2.351223000
6	-4.363102000	-1.950006000	-1.227786000
1	-2.631237000	-1.942344000	-2.484741000
1	-1.771457000	0.302939000	-1.880134000
1	-3.072548000	0.500596000	-0.692608000
1	-3.703358000	1.625286000	-2.823414000
1	-3.424098000	0.129899000	-3.737528000
1	-5.866346000	0.408240000	-3.343530000
1	-5.599673000	0.555743000	-1.595544000
1	-6.337550000	-1.770066000	-2.142170000
1	-5.013014000	-1.942163000	-3.307792000
1	-4.414621000	-3.048499000	-1.129290000
1	-4.698312000	-1.527010000	-0.265263000
1	1.355780000	4.183158000	0.069181000
6	4.482706000	1.029204000	1.245861000
1	4.827168000	-0.005479000	1.314532000
6	5.346633000	2.066841000	1.571687000
6	3.638012000	3.685348000	1.067588000
1	6.365187000	1.828582000	1.897672000
1	3.296485000	4.723340000	0.974717000
6	4.930449000	3.418716000	1.489525000
1	5.615918000	4.232057000	1.743459000

TS(IV-V) (III)
SCF (BP86/SDD/6-31G**) Energy 333 K = -2438.28003685
Thermal correction to Gibbs Free Energy= 0.973923
Lowest Frequency = -16.1297 cm^{-1}
Second Frequency = 18.0852 cm^{-1}
SCF (B3PW91-D3,C6H6/tzvp) Energy 333 K= -3578.06023054

1	0.610744000	-0.896411000	0.853142000
5	-0.650842000	-1.403712000	1.091530000
7	-1.047835000	-2.143817000	-0.160882000
26	0.186664000	0.545172000	0.355458000
7	-0.281394000	2.309758000	-0.301383000
6	0.555247000	3.343381000	-0.304533000
6	1.932211000	3.329291000	0.060856000
6	2.681117000	2.113606000	0.301749000
7	2.080930000	0.873330000	0.327260000
6	2.978391000	-0.260643000	0.259366000
6	3.332019000	-0.980395000	1.440112000
6	4.192522000	-2.088428000	1.315126000
6	4.706101000	-2.484713000	0.073356000
6	4.366416000	-1.759067000	-1.073773000
6	3.512225000	-0.640365000	-1.008703000
6	2.836135000	-0.555791000	2.824648000
6	2.523674000	-1.752756000	3.746113000
6	3.224986000	0.143333000	-2.292922000
6	2.602162000	-0.744359000	-3.391637000
6	-1.589272000	2.592251000	-0.860812000
6	-2.564306000	3.285598000	-0.090911000
6	-3.814211000	3.546838000	-0.689032000
6	-4.094161000	3.148886000	-2.000714000
6	-3.114155000	2.484279000	-2.749750000
6	-1.849283000	2.194993000	-2.204644000
6	-2.282091000	3.796615000	1.324256000
6	-1.965942000	5.312386000	1.318697000
6	-0.760171000	1.553266000	-3.066121000
6	-1.290848000	0.499668000	-4.057816000
6	0.040951000	2.643600000	-3.818787000
6	-3.443869000	3.515444000	2.299917000
6	3.843754000	0.390683000	3.522661000
6	4.501868000	0.837812000	-2.822833000
1	-3.332192000	2.191252000	-3.781482000
1	-5.071786000	3.365573000	-2.444207000
1	-4.578683000	4.080403000	-0.114551000
1	4.781200000	-2.056753000	-2.043724000
1	5.375626000	-3.348708000	0.003761000
1	4.472650000	-2.649287000	2.212420000
1	2.492146000	0.931024000	-2.049312000
1	-0.059093000	1.040723000	-2.379266000
1	-0.445444000	-0.004355000	-4.556145000
1	-1.895563000	-0.269825000	-3.549878000
1	-1.910738000	0.951545000	-4.852451000
1	0.848833000	2.187650000	-4.417548000
1	-0.617748000	3.205068000	-4.505166000
1	0.499692000	3.365452000	-3.123360000
1	2.376932000	-0.142807000	-4.290390000
1	3.286295000	-1.553902000	-3.701357000
1	1.665399000	-1.211203000	-3.045709000
1	4.272256000	1.432890000	-3.724446000
1	4.932594000	1.514554000	-2.067514000
1	5.273140000	0.096332000	-3.098083000
1	-1.389311000	3.265278000	1.700039000
1	-1.756417000	5.667652000	2.342888000
1	-1.091746000	5.552483000	0.692059000
1	-2.824581000	5.887053000	0.928523000
1	-3.147378000	3.779800000	3.329810000
1	-4.337803000	4.116606000	2.057640000
1	-3.736842000	2.454566000	2.288200000
1	1.897840000	0.008402000	2.669890000
1	3.461728000	0.694474000	4.513673000
1	4.812860000	-0.117780000	3.676349000
1	4.027522000	1.303512000	2.934552000
1	2.054977000	-1.396068000	4.679009000
1	1.834654000	-2.465098000	3.263090000
1	3.438350000	-2.299572000	4.037295000
6	-1.516801000	-0.194589000	1.805258000
1	-0.867511000	0.554825000	2.324134000
1	-2.270358000	0.371603000	1.234303000
1	-0.155935000	-2.148033000	1.939409000
14	-2.385183000	-0.962221000	3.355137000
6	-3.160293000	-2.656437000	2.999272000
1	-2.432310000	-3.352434000	2.552524000
1	-3.526302000	-3.101349000	3.941844000
1	-4.022894000	-2.575254000	2.316424000
6	-3.781933000	0.179293000	3.966752000
1	-4.277882000	-0.277870000	4.842051000
1	-3.405504000	1.167737000	4.278683000
1	-4.556646000	0.337782000	3.196479000
6	-1.118340000	-1.130412000	4.756755000
1	-1.603856000	-1.520447000	5.668737000
1	-0.665100000	-0.156126000	5.010917000
1	-0.305822000	-1.821123000	4.481451000
6	-0.020929000	-3.175571000	-0.517604000
6	0.059503000	-3.529119000	-2.019455000
6	1.291691000	-4.413865000	-2.303816000
6	1.282227000	-5.694982000	-1.448281000
6	1.161869000	-5.357813000	0.049852000
6	-0.075148000	-4.479451000	0.327600000
1	0.952687000	-2.705896000	-0.273626000
1	0.110095000	-2.603556000	-2.622443000
1	-0.849527000	-4.071479000	-2.344312000
1	1.329589000	-4.667857000	-3.379347000
1	2.209493000	-3.835279000	-2.081258000
1	2.193992000	-6.290259000	-1.640340000
1	0.423713000	-6.328885000	-1.749339000
1	1.113968000	-6.283661000	0.652484000
1	2.071881000	-4.815335000	0.374493000
1	-0.128169000	-4.214127000	1.397462000
1	-0.988248000	-5.056419000	0.091372000
6	-2.299981000	-2.176911000	-0.947781000
6	-3.222055000	-0.959512000	-0.752954000
6	-4.431182000	-1.000703000	-1.709297000
6	-5.259371000	-2.283213000	-1.521047000
6	-4.365645000	-3.524997000	-1.674568000
6	-3.142141000	-3.471249000	-0.736722000
1	-2.017400000	-2.170872000	-2.022880000
1	-2.656848000	-0.024458000	-0.910534000
1	-3.600355000	-0.945784000	0.285796000
1	-5.054065000	-0.102130000	-1.551701000
1	-4.073910000	-0.948970000	-2.757293000
1	-6.098310000	-2.314789000	-2.240509000
1	-5.711851000	-2.280182000	-0.509031000
1	-4.942493000	-4.448543000	-1.483270000
1	-4.014941000	-3.589934000	-2.724590000
1	-2.519951000	-4.363886000	-0.908051000
1	-3.469803000	-3.513280000	0.318355000
1	0.157898000	4.307342000	-0.659152000
6	4.097912000	2.268007000	0.489847000
1	4.701778000	1.370841000	0.640971000
6	4.709733000	3.515191000	0.510216000
6	2.602838000	4.590647000	0.073362000
1	5.789852000	3.572825000	0.685383000
1	2.001242000	5.487738000	-0.117265000
6	3.963137000	4.702943000	0.308015000
1	4.451939000	5.681273000	0.321821000

V (III)
SCF (BP86/SDD/6-31G**) Energy 333 K = -2438.30596766
Thermal correction to Gibbs Free Energy= 0.970631
Lowest Frequency = 8.6614 cm^{-1}
Second Frequency = 14.1999 cm^{-1}
SCF (B3PW91-D3,C6H6/tzvp) Energy 333 K= -3578.08026103

1	0.641713000	1.041590000	-0.155663000
5	1.472533000	-0.067157000	-0.295012000
7	2.766018000	0.339323000	0.276604000
26	-0.570322000	0.049179000	0.204848000
7	-1.931290000	-1.193592000	0.800788000
6	-3.061221000	-0.802085000	1.381475000
6	-3.601824000	0.517963000	1.404017000
6	-3.030433000	1.620336000	0.658696000
7	-1.788358000	1.526693000	0.074443000
6	-1.358228000	2.665529000	-0.708206000
6	-0.775683000	3.795074000	-0.063457000
6	-0.362795000	4.883810000	-0.855761000
6	-0.515818000	4.875543000	-2.246913000
6	-1.093602000	3.762009000	-2.868356000
6	-1.526727000	2.647547000	-2.123960000
6	-0.578690000	3.861558000	1.451840000
6	0.913088000	4.041427000	1.804796000
6	-2.211021000	1.479947000	-2.836035000
6	-1.509289000	1.069967000	-4.147408000
6	-1.822759000	-2.627129000	0.602431000
6	-1.090939000	-3.434090000	1.518080000
6	-1.017789000	-4.818402000	1.264592000
6	-1.655038000	-5.395211000	0.158982000
6	-2.397364000	-4.589868000	-0.711575000
6	-2.502939000	-3.198801000	-0.512198000
6	-0.473569000	-2.846339000	2.788197000
6	-1.520092000	-2.768707000	3.927906000
6	-3.397931000	-2.369322000	-1.439196000
6	-3.164563000	-2.656525000	-2.937526000
6	-4.889481000	-2.585991000	-1.084782000
6	0.764890000	-3.620982000	3.282088000
6	-1.424355000	4.979838000	2.103099000
6	-3.703573000	1.796243000	-3.096826000
1	-2.915750000	-5.047691000	-1.560770000
1	-1.582672000	-6.473945000	-0.015311000
1	-0.459108000	-5.458850000	1.953334000
1	-1.219547000	3.758920000	-3.956347000
1	-0.189694000	5.733847000	-2.844165000
1	0.084904000	5.757117000	-0.367592000
1	-2.170175000	0.613582000	-2.150131000
1	-3.164561000	-1.304017000	-1.269169000
1	-3.794500000	-1.987529000	-3.549287000
1	-2.113417000	-2.496238000	-3.224521000
1	-3.434033000	-3.692426000	-3.208984000
1	-5.535514000	-1.969627000	-1.733974000
1	-5.175497000	-3.643684000	-1.224525000
1	-5.105010000	-2.316104000	-0.037885000
1	-1.975413000	0.157038000	-4.556957000
1	-1.592815000	1.851213000	-4.923544000
1	-0.437970000	0.865413000	-3.986050000
1	-4.207778000	0.937283000	-3.574790000
1	-4.236393000	2.030982000	-2.160912000
1	-3.804332000	2.665521000	-3.771514000
1	-0.159914000	-1.811789000	2.550359000
1	-1.066353000	-2.339170000	4.838473000
1	-2.383520000	-2.141752000	3.654746000
1	-1.896734000	-3.776548000	4.177651000
1	1.248111000	-3.070041000	4.105933000
1	0.493864000	-4.615952000	3.677990000
1	1.509518000	-3.765428000	2.481731000
1	-0.907762000	2.895481000	1.872190000
1	-1.270432000	4.990475000	3.196698000
1	-1.138118000	5.974233000	1.716102000
1	-2.499920000	4.835316000	1.913687000
1	1.063453000	3.995840000	2.897922000
1	1.523109000	3.253990000	1.334653000
1	1.296618000	5.017583000	1.458044000
6	1.187760000	-0.740334000	-1.749099000
1	0.086646000	-0.922151000	-1.838660000
1	1.403477000	-0.031546000	-2.569219000
1	0.812964000	-0.801480000	0.648594000
14	1.938513000	-2.454578000	-2.202359000
6	3.398954000	-2.296921000	-3.419630000
1	4.291779000	-1.818287000	-2.987010000
1	3.698195000	-3.303776000	-3.762343000
1	3.108805000	-1.718331000	-4.314592000

6	0.627767000	-3.486882000	-3.116076000
1	1.057893000	-4.444281000	-3.460621000
1	-0.227570000	-3.723897000	-2.460959000
1	0.244074000	-2.957963000	-4.006495000
6	2.478167000	-3.424484000	-0.660903000
1	2.944708000	-4.385063000	-0.942112000
1	1.601110000	-3.650913000	-0.029469000
1	3.200142000	-2.857415000	-0.050354000
6	2.788090000	0.531922000	1.758384000
6	3.725375000	1.640645000	2.290623000
6	3.540322000	1.832382000	3.811055000
6	3.801853000	0.526540000	4.582559000
6	2.917860000	-0.613186000	4.044724000
6	3.080237000	-0.785939000	2.521493000
1	1.755848000	0.837365000	2.034107000
1	3.539079000	2.595549000	1.767582000
1	4.782815000	1.370127000	2.102332000
1	4.212159000	2.634031000	4.168474000
1	2.506639000	2.176579000	4.012266000
1	3.628663000	0.675449000	5.664124000
1	4.868039000	0.245184000	4.469912000
1	3.153808000	-1.561296000	4.562086000
1	1.856585000	-0.386035000	4.272379000
1	2.407597000	-1.575911000	2.146658000
1	4.113124000	-1.116125000	2.297355000
6	3.993249000	0.725227000	-0.455554000
6	3.761154000	1.205865000	-1.903659000
6	5.061466000	1.794232000	-2.490272000
6	6.206964000	0.764235000	-2.460889000
6	6.428124000	0.217548000	-1.037998000
6	5.125691000	-0.334360000	-0.419903000
1	4.399203000	1.614460000	0.064865000
1	2.952074000	1.958336000	-1.918910000
1	3.439376000	0.366645000	-2.541933000
1	4.882658000	2.141855000	-3.524155000
1	5.357995000	2.688932000	-1.906534000
1	7.140180000	1.212618000	-2.848665000
1	5.956950000	-0.074009000	-3.141038000
1	7.208581000	-0.565328000	-1.040261000
1	6.810979000	1.035396000	-0.394384000
1	5.313437000	-0.649037000	0.621843000
1	4.794556000	-1.237892000	-0.964469000
1	-3.694017000	-1.589323000	1.822047000
6	-3.838460000	2.802933000	0.552750000
1	-3.468920000	3.632689000	-0.053450000
6	-5.053978000	2.925941000	1.215941000
6	-4.849878000	0.687594000	2.075462000
1	-5.619718000	3.859188000	1.117057000
1	-5.245728000	-0.166632000	2.638148000
6	-5.568602000	1.871005000	2.009411000
1	-6.522831000	1.978095000	2.533080000

VI (V)
SCF (BP86/SDD/6-31G**) Energy 333 K = -1991.49582980
Thermal correction to Gibbs Free Energy= 0.866908
Lowest Frequency = 8.1009 cm^{-1}
Second Frequency = 12.5789 cm^{-1}
SCF (B3PW91-D3,C6H6/tzvp) Energy 333 K= -3131.28982093

26	0.412018000	-0.178043000	-0.146757000
1	-0.488397000	0.357051000	-1.457573000
7	0.698798000	-2.168162000	-0.103219000
6	1.875283000	-2.769252000	0.000510000
6	3.158421000	-2.141562000	0.098627000
6	3.381501000	-0.704513000	0.124930000
7	2.340573000	0.183762000	0.093151000
6	2.622873000	1.592396000	0.123868000
6	2.767417000	2.310080000	-1.101485000
6	2.993975000	3.698730000	-1.043468000
6	3.080579000	4.373581000	0.180507000
6	2.940657000	3.657676000	1.376457000
6	2.709022000	2.269169000	1.378866000
6	2.725939000	1.609502000	-2.460605000
6	1.657879000	2.215822000	-3.395836000
6	2.598177000	1.522307000	2.709918000
6	1.577400000	2.170630000	3.670503000
6	-0.472097000	-2.998781000	-0.209351000
6	-0.983567000	-3.310103000	-1.500581000
6	-2.145301000	-4.102545000	-1.577643000
6	-2.789488000	-4.566803000	-0.423224000

6	-2.273713000	-4.243471000	0.839051000
6	-1.114087000	-3.455656000	0.977023000
6	-0.267473000	-2.862279000	-2.775458000
6	0.522284000	-4.038500000	-3.397013000
6	-0.542054000	-3.160233000	2.365098000
6	-1.614359000	-2.702596000	3.375642000
6	0.229016000	-4.385867000	2.911370000
6	-1.223400000	-2.231520000	-3.808364000
6	4.119209000	1.615489000	-3.132394000
6	3.980497000	1.397572000	3.394398000
1	-2.774645000	-4.618300000	1.738190000
1	-3.688267000	-5.187277000	-0.506705000
1	-2.545873000	-4.367033000	-2.562350000
1	3.015808000	4.186830000	2.333118000
1	3.262012000	5.453799000	0.203017000
1	3.112319000	4.258695000	-1.978305000
1	2.242371000	0.501844000	2.482225000
1	0.180147000	-2.331968000	2.255132000
1	-1.139433000	-2.431309000	4.334276000
1	-2.159964000	-1.818968000	3.006128000
1	-2.350037000	-3.497956000	3.589422000
1	0.667022000	-4.161473000	3.899805000
1	-0.443889000	-5.254349000	3.025886000
1	1.047934000	-4.684425000	2.235798000
1	1.481265000	1.562741000	4.587481000
1	1.894458000	3.181964000	3.982237000
1	0.582133000	2.249622000	3.204969000
1	3.895165000	0.829618000	4.337895000
1	4.705902000	0.877611000	2.747858000
1	4.391251000	2.394275000	3.637200000
1	0.455962000	-2.079849000	-2.487109000
1	1.071616000	-3.706573000	-4.295533000
1	1.254319000	-4.454903000	-2.684552000
1	-0.155896000	-4.857543000	-3.696721000
1	-0.653671000	-1.882909000	-4.687250000
1	-1.979700000	-2.949750000	-4.171808000
1	-1.746367000	-1.362767000	-3.376254000
1	2.443389000	0.557815000	-2.279334000
1	4.088067000	1.070972000	-4.092813000
1	4.458385000	2.646074000	-3.341478000
1	4.875041000	1.132683000	-2.491134000
1	1.640352000	1.674194000	-4.358236000
1	0.656690000	2.136996000	-2.940899000
1	1.861461000	3.278726000	-3.617727000
7	-2.355594000	1.466125000	0.089780000
5	-1.296046000	1.031249000	1.229913000
1	-1.850134000	1.081943000	-0.756210000
1	-1.166513000	-0.208734000	1.161243000
1	-0.248585000	1.605505000	0.929725000
1	-1.640802000	1.334065000	2.350612000
6	-2.378589000	2.972616000	-0.131565000
6	-3.139801000	3.740426000	0.961262000
6	-3.014946000	5.261793000	0.730433000
6	-3.498356000	5.663546000	-0.676012000
6	-2.756231000	4.868939000	-1.767275000
6	-2.880051000	3.347009000	-1.540453000
1	-1.311808000	3.256157000	-0.069211000
1	-2.749267000	3.464423000	1.955493000
1	-4.211361000	3.461425000	0.936855000
1	-3.585322000	5.803180000	1.506226000
1	-1.955645000	5.561083000	0.853540000
1	-3.362197000	6.748746000	-0.833356000
1	-4.586357000	5.468021000	-0.757799000
1	-3.141668000	5.129087000	-2.769235000
1	-1.684843000	5.149348000	-1.760091000
1	-2.298058000	2.796232000	-2.302358000
1	-3.940616000	3.052247000	-1.663837000
6	-3.730426000	0.801944000	0.121625000
6	-4.250559000	0.527004000	1.544125000
6	-5.656504000	-0.106773000	1.489673000
6	-5.659531000	-1.399863000	0.653484000
6	-5.108726000	-1.142603000	-0.760968000
6	-3.711807000	-0.492057000	-0.714605000
1	-4.429202000	1.507632000	-0.367494000
1	-4.266181000	1.453901000	2.141523000
1	-3.554383000	-0.163132000	2.053094000

1	-6.009998000	-0.306843000	2.517064000
1	-6.372054000	0.616897000	1.049208000
1	-6.679689000	-1.821555000	0.598721000
1	-5.026695000	-2.159199000	1.152704000
1	-5.051947000	-2.085305000	-1.332752000
1	-5.806004000	-0.478626000	-1.310839000
1	-3.359931000	-0.271547000	-1.739090000
1	-2.985633000	-1.202358000	-0.277515000
1	1.880057000	-3.872790000	-0.003578000
6	4.747643000	-0.264523000	0.181034000
1	4.940031000	0.811546000	0.187099000
6	5.809206000	-1.158796000	0.229074000
6	4.282160000	-3.020458000	0.156828000
1	6.832656000	-0.769243000	0.272187000
1	4.083690000	-4.099279000	0.141378000
6	5.587795000	-2.558797000	0.222981000
1	6.427667000	-3.258257000	0.264014000

VI (III)
SCF (BP86/SDD/6-31G**) Energy 333 K = -1991.50510443
Thermal correction to Gibbs Free Energy= 0.872811
Lowest Frequency = 9.9077 cm^{-1}
Second Frequency = 14.5530 cm^{-1}
SCF (B3PW91-D3,C6H6/tzvp) Energy 333 K= -3131.28914560

1	-0.729514000	-0.616722000	-1.353609000
1	-2.306251000	-0.104667000	-0.746140000
26	0.391180000	-0.156265000	-0.331861000
7	1.317102000	-1.786308000	-0.550515000
6	2.633928000	-1.918582000	-0.673848000
6	3.613190000	-0.897928000	-0.495820000
6	3.299057000	0.459348000	-0.086970000
7	2.002668000	0.883123000	0.073285000
6	1.829058000	2.242085000	0.523590000
6	1.732413000	3.293093000	-0.434659000
6	1.530486000	4.608636000	0.024899000
6	1.430524000	4.896800000	1.391940000
6	1.531653000	3.856338000	2.323564000
6	1.729943000	2.522330000	1.917212000
6	1.862561000	3.031577000	-1.936881000
6	0.610314000	3.493541000	-2.714037000
6	1.869520000	1.420690000	2.969693000
6	0.741895000	1.458708000	4.023603000
6	0.546338000	-3.009760000	-0.648056000
6	0.078820000	-3.453234000	-1.914459000
6	-0.661872000	-4.649670000	-1.963477000
6	-0.935454000	-5.386919000	-0.804063000
6	-0.462169000	-4.934499000	0.433857000
6	0.285156000	-3.744706000	0.541188000
6	0.395472000	-2.692625000	-3.202020000
6	1.479734000	-3.426775000	-4.024985000
6	0.845260000	-3.303416000	1.895396000
6	-0.180295000	-3.415791000	3.042520000
6	2.128381000	-4.096675000	2.240699000
6	-0.863253000	-2.432926000	-4.055807000
6	3.136686000	3.686011000	-2.519153000
6	3.254675000	1.477903000	3.657046000
1	-0.667227000	-5.520393000	1.336385000
1	-1.509603000	-6.317760000	-0.866639000
1	-1.022909000	-5.013956000	-2.931777000
1	1.459015000	4.082768000	3.393423000
1	1.279425000	5.927957000	1.729811000
1	1.459328000	5.423732000	-0.705148000
1	1.796837000	0.455653000	2.437533000
1	1.122942000	-2.237995000	1.804378000
1	0.247186000	-3.007125000	3.974398000
1	-1.101816000	-2.853277000	2.816056000
1	-0.464102000	-4.463339000	3.246703000
1	2.548550000	-3.759214000	3.204519000
1	1.912409000	-5.177081000	2.321597000
1	2.903771000	-3.965562000	1.467987000
1	0.830601000	0.596845000	4.708643000
1	0.789144000	2.371982000	4.643425000
1	-0.250361000	1.423814000	3.545227000
1	3.359808000	0.658631000	4.390826000
1	4.071951000	1.386956000	2.923171000
1	3.386090000	2.433086000	4.197193000
1	0.796502000	-1.707208000	-2.909618000
1	1.729863000	-2.855611000	-4.936467000
1	2.406948000	-3.560694000	-3.442508000

1	1.133584000	-4.428552000	-4.336657000
1	-0.604571000	-1.827137000	-4.941886000
1	-1.320479000	-3.370102000	-4.419787000
1	-1.621413000	-1.882851000	-3.473809000
1	1.950242000	1.939646000	-2.072327000
1	3.234947000	3.455200000	-3.594931000
1	3.107346000	4.785524000	-2.413305000
1	4.041686000	3.318916000	-2.008226000
1	0.710383000	3.253350000	-3.787540000
1	-0.296139000	2.991603000	-2.334862000
1	0.455704000	4.584056000	-2.628972000
7	-2.657711000	0.599761000	-0.057326000
5	-1.231768000	0.982766000	0.615301000
1	-0.761836000	-0.031660000	1.178926000
1	-0.498759000	1.341779000	-0.359451000
1	-1.322314000	1.920090000	1.372938000
6	-3.219999000	1.734429000	-0.903458000
6	-3.872643000	2.850802000	-0.072029000
6	-4.319974000	4.008380000	-0.990325000
6	-5.262920000	3.517018000	-2.104665000
6	-4.616953000	2.378930000	-2.917321000
6	-4.166464000	1.218183000	-2.004827000
1	-2.325192000	2.155785000	-1.399121000
1	-3.163779000	3.212334000	0.691622000
1	-4.759426000	2.452041000	0.458210000
1	-4.809064000	4.792287000	-0.385251000
1	-3.425336000	4.476525000	-1.445218000
1	-5.538413000	4.353664000	-2.771556000
1	-6.206276000	3.152518000	-1.651083000
1	-5.316442000	2.000138000	-3.683578000
1	-3.736711000	2.772181000	-3.462456000
1	-3.657399000	0.437804000	-2.600923000
1	-5.061639000	0.748847000	-1.551715000
6	-3.677451000	-0.108182000	0.829835000
6	-3.634860000	0.351560000	2.298681000
6	-4.708881000	-0.389301000	3.122590000
6	-4.538631000	-1.916704000	3.027963000
6	-4.543468000	-2.383893000	1.560450000
6	-3.490432000	-1.633918000	0.720071000
1	-4.676097000	0.138768000	0.421004000
1	-3.776931000	1.442783000	2.370280000
1	-2.634300000	0.135515000	2.714589000
1	-4.659346000	-0.057317000	4.174843000
1	-5.716519000	-0.109255000	2.754152000
1	-5.335575000	-2.429807000	3.595941000
1	-3.578888000	-2.204868000	3.500161000
1	-4.359588000	-3.470690000	1.495355000
1	-5.548283000	-2.208980000	1.126071000
1	-3.557374000	-1.946039000	-0.338823000
1	-2.472559000	-1.901033000	1.060995000
1	3.011496000	-2.926369000	-0.910574000
6	4.421221000	1.332145000	0.131633000
1	4.223174000	2.356914000	0.453999000
6	5.731874000	0.918082000	-0.069822000
6	4.975065000	-1.281766000	-0.686903000
1	6.546471000	1.631360000	0.100250000
1	5.172730000	-2.315469000	-0.996519000
6	6.028352000	-0.403293000	-0.488427000
1	7.062879000	-0.723449000	-0.642850000

TS(VI-VII) (V)
SCF (BP86/SDD/6-31G**) Energy 333 K = -1991.47936231
Thermal correction to Gibbs Free Energy= 0.864318
Lowest Frequency = -261.0585 cm^{-1}
Second Frequency = 12.9236 cm^{-1}
SCF (B3PW91-D3,C6H6/tzvp) Energy 333 K= -3131.27842356

1	0.488395000	0.459662000	1.367985000
1	1.083097000	0.889500000	0.933129000
26	-0.343028000	-0.204498000	-0.191449000
7	-0.256362000	-2.193467000	0.188756000
6	-1.314790000	-2.973194000	0.339800000
6	-2.693678000	-2.584055000	0.323081000
6	-3.169044000	-1.226764000	0.128536000
7	-2.298800000	-0.184868000	-0.060641000
6	-2.829748000	1.140498000	-0.242087000
6	-3.016174000	1.983987000	0.893917000
6	-3.500444000	3.289191000	0.685539000
6	-3.792143000	3.762446000	-0.600155000
6	-3.601688000	2.925167000	-1.706426000

6	-3.121120000	1.610408000	-1.557680000
6	-2.738989000	1.497031000	2.317135000
6	-1.780271000	2.437125000	3.079629000
6	-2.954326000	0.722931000	-2.792306000
6	-2.043633000	1.371568000	-3.858516000
6	1.035870000	-2.831548000	0.267776000
6	1.641734000	-3.017512000	1.541412000
6	2.907156000	-3.634425000	1.584368000
6	3.559040000	-4.042939000	0.413960000
6	2.948404000	-3.840496000	-0.830762000
6	1.682298000	-3.232811000	-0.934630000
6	0.938655000	-2.611268000	2.838434000
6	0.347611000	-3.848951000	3.554883000
6	1.002671000	-3.079870000	-2.296548000
6	1.959691000	-2.588011000	-3.401292000
6	0.317266000	-4.402887000	-2.714932000
6	1.856529000	-1.820223000	3.794624000
6	-4.054489000	1.300335000	3.107073000
6	-4.327032000	0.345313000	-3.397323000
1	3.460089000	-4.168355000	-1.741563000
1	4.541732000	-4.522785000	0.471470000
1	3.387625000	-3.799463000	2.554719000
1	-3.832163000	3.297505000	-2.710870000
1	-4.168320000	4.781672000	-0.739392000
1	-3.654958000	3.945268000	1.549597000
1	-2.467445000	-0.212143000	-2.462106000
1	0.210737000	-2.316356000	-2.185026000
1	1.397549000	-2.402349000	-4.332534000
1	2.457796000	-1.648550000	-3.110831000
1	2.740139000	-3.333173000	-3.635864000
1	-0.200040000	-4.284178000	-3.683140000
1	1.061288000	-5.212088000	-2.822513000
1	-0.426689000	-4.729017000	-1.968788000
1	-1.901803000	0.681179000	-4.708702000
1	-2.486830000	2.300013000	-4.260498000
1	-1.052076000	1.618176000	-3.444854000
1	-4.195773000	-0.324280000	-4.265609000
1	-4.963473000	-0.171846000	-2.660439000

1	-4.868375000	1.243501000	-3.744726000
1	0.099065000	-1.947408000	2.566619000
1	-0.182781000	-3.549876000	4.475949000
1	-0.366382000	-4.389593000	2.911138000
1	1.145683000	-4.558383000	3.837177000
1	1.284617000	-1.483199000	4.676546000
1	2.696074000	-2.434204000	4.165067000
1	2.279947000	-0.930761000	3.299929000
1	-2.245387000	0.512491000	2.237109000
1	-3.844446000	0.915272000	4.120641000
1	-4.598841000	2.255503000	3.215876000
1	-4.722408000	0.583803000	2.601459000
1	-1.549074000	2.022868000	4.076747000
1	-0.830971000	2.568737000	2.534122000
1	-2.223731000	3.436715000	3.233813000
5	1.099102000	1.211532000	-1.344231000
7	2.013737000	1.757667000	-0.222647000
1	-0.083372000	1.508760000	-1.014751000
1	1.240204000	1.592131000	-2.491415000
6	1.720921000	3.167262000	0.159962000
6	2.237210000	4.221428000	-0.851415000
6	1.792735000	5.645124000	-0.458489000
6	2.234928000	5.999165000	0.973751000
6	1.733779000	4.949074000	1.983237000
6	2.183885000	3.525966000	1.591626000
1	0.613335000	3.233935000	0.157675000
1	1.877357000	3.966244000	-1.863472000
1	3.344549000	4.183301000	-0.881340000
1	2.193194000	6.380663000	-1.180335000
1	0.688429000	5.711253000	-0.525031000
1	1.871900000	7.005332000	1.253251000
1	3.342035000	6.042132000	1.013111000
1	2.088806000	5.190342000	3.001975000
1	0.626689000	4.981917000	2.021564000
1	1.784705000	2.783389000	2.306873000
1	3.288095000	3.472508000	1.661339000
6	3.460064000	1.391007000	-0.258495000
6	4.059438000	1.305910000	-1.683995000

6	5.562756000	0.964838000	-1.644201000
6	5.822744000	-0.345249000	-0.877903000
6	5.226122000	-0.279691000	0.539975000
6	3.727060000	0.077055000	0.508199000
1	4.015140000	2.187984000	0.275351000
1	3.889337000	2.254154000	-2.223025000
1	3.521446000	0.524565000	-2.253636000
1	5.960924000	0.897932000	-2.673546000
1	6.114623000	1.790412000	-1.150324000
1	6.906686000	-0.558974000	-0.832111000
1	5.356075000	-1.187108000	-1.426750000
1	5.368559000	-1.241226000	1.065338000
1	5.772513000	0.485922000	1.127726000
1	3.336800000	0.173516000	1.537643000
1	3.163516000	-0.746493000	0.029503000
1	-1.125834000	-4.048428000	0.501249000
6	-4.589713000	-1.028139000	0.146148000
1	-4.969715000	-0.013484000	0.002444000
6	-5.476123000	-2.080784000	0.338433000
6	-3.642016000	-3.632833000	0.515278000
1	-6.552632000	-1.876376000	0.343619000
1	-3.254924000	-4.648955000	0.658968000
6	-5.010090000	-3.405312000	0.525879000
1	-5.713202000	-4.229381000	0.676154000
1	1.177732000	-0.061084000	-1.382103000

TS(VI-VII) (III)
SCF (BP86/SDD/6-31G**) Energy 333 K = -1991.48104907
Thermal correction to Gibbs Free Energy= 0.868787
Lowest Frequency = -325.8108 cm^{-1}
Second Frequency = 13.5616 cm^{-1}
SCF (B3PW91-D3,C6H6/tzvp) Energy 333 K= -3131.26596836

1	-0.709477000	0.160144000	-1.345050000
1	-1.292388000	0.639280000	-0.907766000
26	0.327401000	-0.314340000	0.101971000
7	0.406126000	-2.141266000	-0.298488000
6	1.490746000	-2.901354000	-0.394685000
6	2.835904000	-2.451604000	-0.281688000
6	3.182758000	-1.068037000	-0.032303000
7	2.220481000	-0.104886000	0.148933000
6	2.678878000	1.249123000	0.354502000
6	2.859808000	2.103729000	-0.771279000
6	3.283801000	3.427583000	-0.544644000
6	3.530322000	3.901746000	0.749634000
6	3.357740000	3.045944000	1.845322000
6	2.933996000	1.714193000	1.677331000
6	2.642674000	1.614856000	-2.204538000
6	1.639599000	2.497155000	-2.978718000
6	2.783072000	0.807786000	2.900383000
6	1.861185000	1.424340000	3.975587000
6	-0.873894000	-2.801003000	-0.456867000
6	-1.444959000	-2.905972000	-1.754675000
6	-2.684626000	-3.563034000	-1.875848000
6	-3.338109000	-4.096334000	-0.757841000
6	-2.757285000	-3.980896000	0.511840000
6	-1.520739000	-3.332147000	0.693907000
6	-0.726370000	-2.384651000	-2.999774000
6	-0.079670000	-3.550415000	-3.785349000
6	-0.882905000	-3.265378000	2.082607000
6	-1.874330000	-2.813758000	3.175155000
6	-0.239658000	-4.622747000	2.454453000
6	-1.649109000	-1.553377000	-3.915814000
6	3.983941000	1.512852000	-2.968488000
6	4.161648000	0.450443000	3.505004000
1	-3.269592000	-4.408236000	1.380201000
1	-4.299034000	-4.608217000	-0.876594000
1	-3.140823000	-3.664450000	-2.866300000
1	3.559162000	3.417347000	2.856363000
1	3.861205000	4.934423000	0.904372000
1	3.429148000	4.095819000	-1.401087000
1	2.312541000	-0.130207000	2.556463000
1	-0.076356000	-2.511753000	2.040928000
1	-1.348576000	-2.697122000	4.138385000
1	-2.337150000	-1.846202000	2.920035000
1	-2.682654000	-3.549708000	3.330981000
1	0.246762000	-4.563405000	3.443762000
1	-1.001506000	-5.421351000	2.496866000
1	0.523293000	-4.926112000	1.717987000
1	1.726762000	0.715790000	4.812006000

1	2.289624000	2.351098000	4.397221000
1	0.867724000	1.662190000	3.562056000
1	4.040687000	-0.229679000	4.366742000
1	4.810478000	-0.047394000	2.765934000
1	4.683813000	1.356062000	3.862789000
1	0.085105000	-1.718097000	-2.658801000
1	0.465206000	-3.169570000	-4.666839000
1	0.634581000	-4.112944000	-3.160841000
1	-0.846792000	-4.261256000	-4.140570000
1	-1.070107000	-1.134254000	-4.756840000
1	-2.460402000	-2.163638000	-4.350002000
1	-2.109343000	-0.716231000	-3.365545000
1	2.211977000	0.599999000	-2.144420000
1	3.820693000	1.122115000	-3.988534000
1	4.465512000	2.502870000	-3.060663000
1	4.687792000	0.839000000	-2.453078000
1	1.473621000	2.091352000	-3.992237000
1	0.665495000	2.539321000	-2.463541000
1	2.008579000	3.531704000	-3.094279000
7	-2.070715000	1.577070000	0.177761000
5	-1.008583000	1.070735000	1.179749000
1	-1.041954000	-0.220739000	1.271984000
1	0.149219000	1.318495000	0.656718000
1	-1.003297000	1.518980000	2.308876000
6	-1.820473000	2.957864000	-0.326880000
6	-2.190945000	4.084094000	0.669181000
6	-1.815322000	5.474311000	0.115766000
6	-2.459550000	5.726546000	-1.260176000
6	-2.094560000	4.608111000	-2.254328000
6	-2.471290000	3.216241000	-1.705424000
1	-0.721949000	3.009475000	-0.477530000
1	-1.688805000	3.901314000	1.635399000
1	-3.282262000	4.055002000	0.860962000
1	-2.113226000	6.260977000	0.833193000
1	-0.713298000	5.540349000	0.019333000
1	-2.149751000	6.710556000	-1.657629000
1	-3.561489000	5.766599000	-1.145071000
1	-2.590505000	4.776640000	-3.227816000
1	-1.004113000	4.638817000	-2.449481000
1	-2.161589000	2.423656000	-2.411127000
1	-3.574092000	3.154239000	-1.621810000
6	-3.498687000	1.249335000	0.466733000
6	-3.864624000	1.238684000	1.971018000
6	-5.358370000	0.920514000	2.184517000
6	-5.748883000	-0.418330000	1.531546000
6	-5.383480000	-0.427913000	0.035650000
6	-3.895944000	-0.093264000	-0.188265000
1	-4.121171000	2.034123000	-0.007466000
1	-3.603089000	2.207436000	2.431230000
1	-3.251807000	0.475183000	2.487999000
1	-5.589883000	0.906926000	3.265518000
1	-5.973052000	1.732140000	1.744809000
1	-6.828372000	-0.615174000	1.667634000
1	-5.210507000	-1.241654000	2.041871000
1	-5.617289000	-1.409613000	-0.414964000
1	-6.010246000	0.318143000	-0.494053000
1	-3.671219000	-0.050343000	-1.269227000
1	-3.268380000	-0.902629000	0.232293000
1	1.332125000	-3.972845000	-0.588201000
6	4.584481000	-0.756017000	0.008142000
1	4.880568000	0.280923000	0.181414000
6	5.558102000	-1.732484000	-0.159854000
6	3.867714000	-3.424362000	-0.444238000
1	6.613612000	-1.441741000	-0.115217000
1	3.565880000	-4.462905000	-0.626222000
6	5.210509000	-3.088720000	-0.384648000
1	5.985038000	-3.850226000	-0.512677000

VII (V)
SCF (BP86/SDD/6-31G**) Energy 333 K = -1991.47977206
Thermal correction to Gibbs Free Energy= 0.863456
Lowest Frequency = 11.3873 cm^{-1}
Second Frequency = 14.5884 cm^{-1}
SCF (B3PW91-D3,C6H6/tzvp) Energy 333 K= -3131.27854161

1	0.216832000	0.457168000	1.606678000
1	0.803008000	0.800583000	1.197165000
26	-0.325046000	-0.174981000	-0.166820000
7	-0.167503000	-2.139227000	0.311114000
6	-1.200431000	-2.945788000	0.498746000

6	-2.592195000	-2.609928000	0.455778000
6	-3.120179000	-1.286917000	0.175073000
7	-2.293730000	-0.220383000	-0.066482000
6	-2.889673000	1.060610000	-0.343883000
6	-3.145970000	1.964710000	0.729661000
6	-3.690845000	3.227050000	0.427323000
6	-3.977786000	3.600210000	-0.891771000
6	-3.721745000	2.703061000	-1.936101000
6	-3.177857000	1.427807000	-1.692107000
6	-2.880984000	1.585460000	2.187723000
6	-1.983040000	2.616393000	2.906086000
6	-2.940532000	0.471304000	-2.861873000
6	-2.054833000	1.099316000	-3.960703000
6	1.141144000	-2.740671000	0.411930000
6	1.765708000	-2.838703000	1.686099000
6	3.045677000	-3.422907000	1.750063000
6	3.692214000	-3.888831000	0.598438000
6	3.060793000	-3.778535000	-0.647458000
6	1.780986000	-3.204229000	-0.771762000
6	1.067960000	-2.379460000	2.968245000
6	0.552447000	-3.592519000	3.778725000
6	1.083877000	-3.152284000	-2.132531000
6	2.011374000	-2.678000000	-3.269925000
6	0.454864000	-4.524268000	-2.474618000
6	1.964914000	-1.481198000	3.846742000
6	-4.203859000	1.382256000	2.963559000
6	-4.279755000	-0.028225000	-3.453691000
1	3.567298000	-4.153161000	-1.543070000
1	4.685714000	-4.343572000	0.672062000
1	3.540889000	-3.518718000	2.722458000
1	-3.949419000	2.996352000	-2.967019000
1	-4.401784000	4.587321000	-1.105106000
1	-3.898168000	3.928464000	1.243491000
1	-2.403264000	-0.407878000	-2.463608000
1	0.261014000	-2.418772000	-2.053616000
1	1.436934000	-2.569902000	-4.205913000
1	2.466342000	-1.701790000	-3.034656000
1	2.825448000	-3.397118000	-3.468830000

1	-0.074633000	-4.479252000	-3.442433000
1	1.232644000	-5.305166000	-2.547362000
1	-0.268334000	-4.842741000	-1.705088000
1	-1.857988000	0.361039000	-4.758126000
1	-2.547521000	1.967819000	-4.433058000
1	-1.086959000	1.434994000	-3.553651000
1	-4.096604000	-0.745723000	-4.273006000
1	-4.895213000	-0.531726000	-2.689999000
1	-4.868055000	0.810555000	-3.867193000
1	0.188298000	-1.780751000	2.672420000
1	0.023719000	-3.257775000	4.688399000
1	-0.144507000	-4.208843000	3.186396000
1	1.389349000	-4.241736000	4.091438000
1	1.397190000	-1.112166000	4.718604000
1	2.841481000	-2.028370000	4.235357000
1	2.336192000	-0.608843000	3.283652000
1	-2.345836000	0.619224000	2.183440000
1	-4.000441000	1.075967000	4.005004000
1	-4.792014000	2.316787000	2.996020000
1	-4.827258000	0.603705000	2.494354000
1	-1.755645000	2.280050000	3.933055000
1	-1.028800000	2.760934000	2.371991000
1	-2.475611000	3.601597000	2.984574000
5	0.952605000	1.286618000	-1.368159000
7	1.950448000	1.794223000	-0.323163000
1	-0.199747000	1.578381000	-0.949232000
1	1.015027000	1.668467000	-2.525543000
6	1.662159000	3.141400000	0.226078000
6	2.079703000	4.297707000	-0.722183000
6	1.667308000	5.673507000	-0.160439000
6	2.232113000	5.897606000	1.254449000
6	1.819239000	4.754439000	2.200463000
6	2.237579000	3.377693000	1.643019000
1	0.557195000	3.187126000	0.315203000
1	1.629681000	4.130640000	-1.716550000
1	3.179690000	4.274784000	-0.856814000
1	2.000539000	6.477262000	-0.842729000
1	0.561203000	5.733593000	-0.123975000

1	1.894483000	6.870507000	1.656599000
1	3.338483000	5.945405000	1.202445000
1	2.257631000	4.903909000	3.204283000
1	0.719059000	4.774787000	2.333867000
1	1.901966000	2.569494000	2.319005000
1	3.343732000	3.328006000	1.618007000
6	3.388939000	1.458335000	-0.476695000
6	3.869498000	1.406745000	-1.948199000
6	5.377733000	1.099552000	-2.039253000
6	5.730676000	-0.213298000	-1.315961000
6	5.254422000	-0.180307000	0.147921000
6	3.750071000	0.141243000	0.247006000
1	3.972692000	2.260443000	0.018114000
1	3.634040000	2.360590000	-2.452483000
1	3.301845000	0.624159000	-2.486596000
1	5.689761000	1.054575000	-3.099103000
1	5.950870000	1.931376000	-1.581411000
1	6.819235000	-0.401582000	-1.364461000
1	5.239496000	-1.059214000	-1.836652000
1	5.462626000	-1.144515000	0.645658000
1	5.830869000	0.591075000	0.698355000
1	3.442003000	0.215526000	1.305803000
1	3.165662000	-0.687167000	-0.196812000
1	-0.974392000	-4.004376000	0.713817000
6	-4.549093000	-1.150179000	0.165429000
1	-4.970165000	-0.164149000	-0.046216000
6	-5.393246000	-2.225015000	0.414995000
6	-3.499027000	-3.683842000	0.704405000
1	-6.477286000	-2.066089000	0.396263000
1	-3.071526000	-4.672330000	0.912267000
6	-4.875225000	-3.514572000	0.689926000
1	-5.545032000	-4.356826000	0.884861000
1	1.008972000	0.001341000	-1.465321000

VII (III)
SCF (BP86/SDD/6-31G**) Energy 333 K = -1991.49899859
Thermal correction to Gibbs Free Energy= 0.865137
Lowest Frequency = 6.8342 cm^{-1}
Second Frequency = 10.1523 cm^{-1}
SCF (B3PW91-D3,C6H6/tzvp) Energy 333 K= -3131.27114087

1	-0.456279000	-0.956505000	1.733008000
1	0.008280000	-0.325000000	1.828716000
26	-0.445938000	-0.204452000	0.003332000
7	-1.499371000	-1.672229000	-0.634235000
6	-2.762272000	-1.605959000	-1.024820000
6	-3.625014000	-0.474965000	-0.904676000
6	-3.215557000	0.803310000	-0.351720000
7	-1.918900000	1.056535000	0.029524000
6	-1.642411000	2.385536000	0.526201000
6	-1.738687000	2.658106000	1.921476000
6	-1.438924000	3.956334000	2.379106000
6	-1.063793000	4.972417000	1.493142000
6	-0.986143000	4.695907000	0.122295000
6	-1.269787000	3.415422000	-0.389666000
6	-2.192879000	1.598744000	2.927592000
6	-1.172187000	1.406412000	4.071119000
6	-1.205559000	3.176172000	-1.900662000
6	0.079438000	3.742484000	-2.542105000
6	-0.842379000	-2.957346000	-0.756412000
6	-0.937535000	-3.887753000	0.314825000
6	-0.276527000	-5.124544000	0.177920000
6	0.452572000	-5.435828000	-0.975551000
6	0.529355000	-4.506887000	-2.021410000
6	-0.108359000	-3.254813000	-1.938409000
6	-1.764494000	-3.603305000	1.570237000
6	-3.069706000	-4.433775000	1.571481000
6	-0.050899000	-2.283123000	-3.117703000
6	1.381732000	-2.080780000	-3.654143000
6	-1.003139000	-2.740805000	-4.248191000
6	-0.964993000	-3.841409000	2.870039000
6	-3.591244000	1.928959000	3.501096000
6	-2.449064000	3.759688000	-2.615078000
1	1.090890000	-4.760240000	-2.927009000
1	0.955864000	-6.404588000	-1.062979000
1	-0.343281000	-5.858215000	0.988776000
1	-0.701292000	5.494719000	-0.571236000
1	-0.838213000	5.976807000	1.867444000
1	-1.512011000	4.174418000	3.450866000
1	-1.204613000	2.082818000	-2.056885000

1	-0.407099000	-1.302081000	-2.757229000
1	1.382305000	-1.318578000	-4.452288000
1	2.063141000	-1.740128000	-2.857439000
1	1.796261000	-3.008323000	-4.087054000
1	-0.987585000	-2.020074000	-5.084426000
1	-0.702167000	-3.727318000	-4.643492000
1	-2.043589000	-2.826807000	-3.891792000
1	0.127321000	3.457391000	-3.607810000
1	0.108822000	4.845907000	-2.499585000
1	0.982338000	3.354869000	-2.044066000
1	-2.402579000	3.555468000	-3.699686000
1	-3.384108000	3.326619000	-2.225780000
1	-2.497737000	4.855530000	-2.480444000
1	-2.054193000	-2.537558000	1.546463000
1	-3.678963000	-4.201359000	2.462408000
1	-3.679967000	-4.225763000	0.676791000
1	-2.849928000	-5.516006000	1.582903000
1	-1.567583000	-3.553517000	3.748935000
1	-0.690898000	-4.903648000	2.993744000
1	-0.032118000	-3.252603000	2.885011000
1	-2.276993000	0.642666000	2.382384000
1	-3.919655000	1.142206000	4.203541000
1	-3.579615000	2.887084000	4.050769000
1	-4.343434000	2.005885000	2.699123000
1	-1.485212000	0.581968000	4.736033000
1	-0.165963000	1.173850000	3.682473000
1	-1.083486000	2.314750000	4.692838000
7	2.844658000	0.354984000	0.077920000
5	1.529588000	0.527805000	-0.517237000
1	0.958123000	-0.666606000	-0.754315000
1	0.599385000	1.002767000	0.363368000
1	1.500068000	1.212475000	-1.515133000
6	3.052247000	-0.546870000	1.224234000
6	3.408207000	0.201283000	2.532883000
6	3.548290000	-0.768482000	3.723963000
6	4.569945000	-1.882550000	3.429222000
6	4.207723000	-2.636326000	2.135771000
6	4.075094000	-1.674482000	0.937439000

1	2.076882000	-1.044888000	1.389180000
1	2.634695000	0.964316000	2.736247000
1	4.363016000	0.746592000	2.396140000
1	3.832749000	-0.210895000	4.635006000
1	2.562446000	-1.229356000	3.936279000
1	4.630199000	-2.584884000	4.280690000
1	5.577548000	-1.433500000	3.319936000
1	4.962702000	-3.412772000	1.914459000
1	3.246940000	-3.169281000	2.282489000
1	3.762444000	-2.221673000	0.030001000
1	5.067508000	-1.232841000	0.720439000
6	4.020962000	1.113666000	-0.399852000
6	3.841996000	2.643373000	-0.249206000
6	5.093394000	3.413213000	-0.715765000
6	5.474511000	3.047455000	-2.162796000
6	5.662068000	1.526245000	-2.317018000
6	4.412447000	0.751650000	-1.852597000
1	4.876782000	0.828179000	0.243708000
1	3.603523000	2.882551000	0.802911000
1	2.967189000	2.955682000	-0.850042000
1	4.923527000	4.501500000	-0.622650000
1	5.943581000	3.172589000	-0.045414000
1	6.393481000	3.581725000	-2.466571000
1	4.671402000	3.386235000	-2.847033000
1	5.897755000	1.269032000	-3.366064000
1	6.536685000	1.206579000	-1.714625000
1	4.579619000	-0.338552000	-1.924316000
1	3.558233000	0.988054000	-2.514431000
1	-3.203266000	-2.520012000	-1.454681000
6	-4.249617000	1.793777000	-0.218600000
1	-3.984737000	2.761730000	0.212292000
6	-5.556477000	1.561526000	-0.629402000
6	-4.975801000	-0.666326000	-1.324199000
1	-6.299305000	2.358139000	-0.508615000
1	-5.242657000	-1.642808000	-1.746623000
6	-5.938748000	0.322699000	-1.200466000
1	-6.968182000	0.148668000	-1.526136000

$Cy_2NH{\cdot}BH_3$

SCF (BP86/SDD/6-31G**) Energy 333 K = -552.555873460
Thermal correction to Gibbs Free Energy= 0.314770
Lowest Frequency = 30.0053 cm^{-1}
Second Frequency = 59.0602 cm^{-1}
SCF (B3PW91-D3,C6H6/tzvp) Energy 333 K= -552.588664159

1	4.187130000	-0.689298000	-1.388530000
1	3.815537000	0.381310000	1.481363000
1	4.660661000	1.211361000	0.165741000
1	0.177155000	2.196475000	1.714857000
1	-1.359250000	2.698147000	0.489615000
1	0.514032000	3.201701000	-0.027683000
5	-0.182271000	2.393509000	0.563497000
1	0.157528000	1.201232000	-1.229517000
6	-1.734745000	-0.219110000	1.182860000
6	-2.978807000	-1.130714000	1.146458000
6	-4.085378000	-0.545950000	0.249314000
6	-3.557004000	-0.269482000	-1.170800000
6	-2.321067000	0.651754000	-1.138111000
6	-1.222722000	0.051588000	-0.240522000
7	0.014485000	0.939919000	-0.241416000
6	2.500588000	1.163231000	-0.060795000
6	1.297439000	0.232137000	0.189495000
1	4.989913000	-1.308042000	0.067575000
1	2.995851000	-2.735084000	-0.574952000
1	2.802339000	-2.010576000	1.029360000
1	1.532037000	-0.994579000	-1.594490000
1	0.709463000	-1.840361000	-0.264570000
1	1.186996000	0.088735000	1.279664000
1	2.550460000	1.393838000	-1.145934000
1	2.334972000	2.117811000	0.459276000
1	-0.933111000	-0.912964000	-0.697760000
1	-2.598200000	1.647608000	-0.752545000
1	-1.928128000	0.798048000	-2.163846000
1	-4.343626000	0.189068000	-1.796141000
1	-3.292558000	-1.231056000	-1.655330000
1	-4.451205000	0.400848000	0.692093000
1	-4.950514000	-1.232326000	0.209678000
1	-3.353984000	-1.284733000	2.173867000
1	-2.693813000	-2.132280000	0.765215000
1	-1.978215000	0.747331000	1.657195000
1	-0.942201000	-0.685442000	1.795913000
6	1.525360000	-1.134063000	-0.493627000
6	2.860602000	-1.771874000	-0.051040000
6	4.058035000	-0.840361000	-0.298287000
6	3.827815000	0.518885000	0.382107000

Cy_2N-BH_2
SCF (BP86/SDD/6-31G**) Energy 333 K = -551.380445328
Thermal correction to Gibbs Free Energy= 0.293996
Lowest Frequency = 43.6796 cm^{-1}
Second Frequency = 63.3692 cm^{-1}
SCF (B3PW91-D3,C6H6/tzvp) Energy 333 K= -551.402175272

6	3.260575000	1.270079000	0.438963000
1	-1.451654000	-0.003675000	-2.972930000
1	0.604186000	-0.004201000	-3.403228000
6	1.774396000	-1.274419000	0.031033000
6	3.260920000	-1.268780000	0.441872000
6	3.624500000	0.001608000	1.232716000
6	1.774045000	1.274394000	0.028110000
6	-1.932688000	-1.271760000	-0.243989000
6	-1.932569000	1.271030000	-0.247229000
1	2.016300000	-0.001942000	-1.687229000
1	-0.545022000	0.000895000	0.793583000
6	-1.057792000	-0.000325000	-0.187990000
6	1.406710000	-0.000975000	-0.764505000
7	-0.000257000	-0.001658000	-1.237391000
5	-0.302536000	-0.003287000	-2.607089000
6	-3.005899000	-1.268995000	0.862360000
6	-3.875309000	0.001069000	0.802732000
6	-3.005783000	1.271200000	0.859114000
1	1.149538000	-1.340854000	0.943546000
1	1.541547000	-2.163954000	-0.581607000
1	3.491980000	-2.173676000	1.032553000
1	3.889823000	-1.321469000	-0.469065000
1	3.074747000	0.002639000	2.195239000
1	4.700195000	0.002058000	1.486104000
1	3.491448000	2.176378000	1.027560000
1	3.889437000	1.320843000	-0.472120000
1	1.149163000	1.342787000	0.940463000
1	1.540981000	2.162469000	-0.586565000
1	-2.418518000	-1.318432000	-1.236825000

1	-1.288448000	-2.166013000	-0.163465000
1	-3.634971000	-2.173779000	0.778879000
1	-2.511517000	-1.324432000	1.853349000
1	-4.456311000	-0.000105000	-0.140724000
1	-4.612253000	0.002158000	1.626450000
1	-3.634763000	2.175830000	0.773305000
1	-2.511398000	1.329127000	1.849956000
1	-2.418388000	1.315194000	-1.240185000
1	-1.288262000	2.165436000	-0.168985000

Cy$_2$N-BHCH$_2$TMS
SCF (BP86/SDD/6-31G**) Energy 333 K = -999.390209708
Thermal correction to Gibbs Free Energy= 0.404997
Lowest Frequency = 13.9218 cm^{-1}
Second Frequency = 34.6582 cm^{-1}
SCF (B3PW91-D3,C6H6/tzvp) Energy 333 K= -999.391797551

1	-5.870418000	-0.640556000	0.281938000
1	-4.992887000	0.885556000	0.010666000
1	-5.350268000	-0.191443000	-1.361856000
1	1.477039000	-1.492794000	1.859068000
1	2.579139000	-0.129112000	1.573320000
1	-4.386916000	-3.395924000	0.029913000
1	-2.658530000	-3.455406000	-0.407815000
1	-3.867903000	-2.972407000	-1.620867000
1	-2.357015000	-0.445346000	-2.123399000
1	-2.129612000	0.860135000	-0.940782000
1	1.784718000	1.338220000	0.247832000
1	3.257362000	0.402640000	-0.975439000
1	2.566286000	-0.637948000	-2.239099000
6	-2.969290000	-1.015208000	1.829056000
6	0.531982000	2.122072000	-1.295303000
6	0.782918000	3.599376000	-0.932886000
6	-0.088875000	4.047601000	0.255263000
6	0.124903000	3.132491000	1.475788000
6	-0.133808000	1.653111000	1.126688000
6	3.527716000	-2.067495000	1.341069000
6	4.662153000	-1.638515000	0.392206000
6	4.166616000	-1.566043000	-1.064000000
6	2.943599000	-0.635963000	-1.200471000
1	1.501497000	-2.084769000	-0.541452000
1	4.977027000	-1.224890000	-1.733525000
1	3.888824000	-2.583831000	-1.403357000
1	5.038337000	-0.642231000	0.699915000
6	0.732536000	1.200401000	-0.070003000
6	1.802999000	-1.062984000	-0.247689000
6	-3.596079000	-2.893364000	-0.553892000
6	2.301894000	-1.139985000	1.213864000
6	-5.055771000	-0.172497000	-0.298096000
14	-3.402811000	-1.078136000	-0.022598000
1	5.516312000	-2.335135000	0.473922000
1	3.882106000	-2.083677000	2.387769000
1	3.224908000	-3.105475000	1.097841000
1	-1.201994000	1.513738000	0.882554000
1	0.076895000	1.003325000	1.994986000
1	-0.527334000	3.442670000	2.312377000
1	1.167608000	3.246886000	1.835243000
1	-1.155944000	4.014853000	-0.042439000
1	0.131096000	5.098196000	0.519022000
1	0.598061000	4.240976000	-1.813627000
1	1.851147000	3.734860000	-0.668034000
1	-0.497007000	2.003199000	-1.679948000
1	1.208889000	1.799223000	-2.106650000
6	-2.048408000	-0.232035000	-1.078478000
1	-2.881034000	0.023000000	2.192956000
1	-3.746051000	-1.514626000	2.434256000
1	-2.009402000	-1.520505000	2.032176000
5	-0.615338000	-0.862329000	-0.865616000
7	0.572101000	-0.241194000	-0.399930000
1	-0.494117000	-2.043276000	-1.122187000

CH$_3$TMS
SCF (BP86/SDD/6-31G**) Energy 333 K = -449.199853572
Thermal correction to Gibbs Free Energy= 0.106136
Lowest Frequency = 135.4644 cm^{-1}
Second Frequency = 156.6485 cm^{-1}
SCF (B3PW91-D3,C6H6/tzvp) Energy 333 K= -449.175151150

1	-1.739384000	-0.182521000	1.817427000
1	-0.861066000	-1.720671000	1.630742000
1	-0.102422000	-0.360989000	2.495250000
1	-0.783853000	2.386426000	0.229693000
1	0.613872000	2.287737000	-0.869509000
1	0.858556000	2.197483000	0.892392000

1	-2.150621000	-0.015092000	-1.318070000
1	-0.745403000	-0.127340000	-2.406542000
1	1.631299000	-1.899256000	-0.312303000
1	2.167207000	-0.457009000	-1.208820000
1	2.399667000	-0.546840000	0.554333000
1	-1.289523000	-1.562725000	-1.503602000
6	1.705709000	-0.798919000	-0.266222000
6	-0.743716000	-0.623133000	1.635905000
6	0.189720000	1.891566000	0.069356000
14	0.000207000	0.000018000	-0.000086000
6	-1.151918000	-0.469422000	-1.439005000

H_2
SCF (BP86/SDD/6-31G**) Energy 333 K = -1.17646513411
Thermal correction to Gibbs Free Energy= -0.003337
Lowest Frequency = 4355.8266 cm^{-1}
SCF (B3PW91-D3,C6H6/tzvp) Energy 333 K= -1.17868640424

1	0.000000000	0.000000000	0.375271000
1	0.000000000	0.000000000	-0.375271000

b. Positional isomerization:

[2b]$_2$ (VII) [Iron1-essai11-septet]
SCF (BP86/SDD/6-31G**) Energy 333 K = -2877.89891372
Thermal correction to Gibbs Free Energy= 1.096964
Lowest Frequency = 11.3889 cm^{-1}
Second Frequency = 18.4905 cm^{-1}
SCF (B3PW91-D3,C6H6/tzvp) Energy 333 K= -5157.40170198

26	-1.290158000	0.259517000	0.115492000
26	1.196989000	-0.262716000	-0.288491000
7	-3.013811000	-0.202611000	-0.646230000
7	-2.004916000	1.772229000	1.153095000
7	2.428282000	-1.765861000	0.048984000
7	2.540344000	0.709092000	-1.452451000
6	-4.114322000	0.615406000	-0.726267000
6	-5.259445000	0.275314000	-1.528088000
1	-5.237272000	-0.656644000	-2.096624000
6	-6.373893000	1.098178000	-1.608015000
1	-7.208611000	0.799924000	-2.252327000
6	-6.451228000	2.308090000	-0.870190000
1	-7.335212000	2.948638000	-0.937184000
6	-5.387776000	2.647790000	-0.052386000
1	-5.426867000	3.563256000	0.550406000
6	-4.211014000	1.841210000	0.042953000
6	-3.227177000	-1.557809000	-1.104856000
6	-3.856657000	-2.480852000	-0.214129000
6	-4.164738000	-3.771768000	-0.683347000
1	-4.657345000	-4.480379000	-0.007942000
6	-3.863894000	-4.162747000	-1.993089000
1	-4.123164000	-5.167321000	-2.344174000
6	-3.222356000	-3.259416000	-2.847973000
1	-2.985203000	-3.565309000	-3.873174000
6	-2.891041000	-1.954714000	-2.431025000
6	-3.230386000	2.260037000	0.985178000
1	-3.534517000	3.077331000	1.659411000
6	-1.244223000	2.393127000	2.217596000
6	-0.770615000	3.729041000	2.069308000
6	0.012863000	4.260091000	3.114091000
1	0.398004000	5.279942000	3.027209000
6	0.299689000	3.518926000	4.267651000
1	0.913269000	3.957320000	5.061813000
6	-0.222288000	2.228038000	4.414016000
1	-0.026037000	1.667153000	5.332818000
6	-1.008464000	1.644261000	3.402984000
6	3.569617000	-2.087899000	-0.635010000
6	4.068539000	-1.240228000	-1.701602000
6	5.217289000	-1.656674000	-2.443052000
1	5.567499000	-1.001220000	-3.249683000
6	5.898240000	-2.829373000	-2.158385000
1	6.773684000	-3.124463000	-2.743808000
6	5.454681000	-3.621869000	-1.069339000
1	5.996592000	-4.537089000	-0.805820000
6	4.340407000	-3.258486000	-0.324724000
1	4.015805000	-3.882591000	0.511470000
6	-4.228707000	-2.109184000	1.222691000
1	-3.843429000	-1.092755000	1.413621000
6	-5.762042000	-2.073666000	1.418989000
1	-6.013924000	-1.755257000	2.445951000
1	-6.207659000	-3.071951000	1.259048000
1	-6.238709000	-1.372683000	0.714391000
6	-2.232403000	-1.006659000	-3.431894000
1	-1.978427000	-0.078703000	-2.890745000
6	-0.922941000	-1.605955000	-3.990368000
1	-0.436836000	-0.898304000	-4.684174000
1	-1.108243000	-2.538799000	-4.551634000
1	-0.212798000	-1.835888000	-3.178145000
6	-1.095101000	4.570342000	0.827396000
1	-2.164590000	4.418009000	0.592783000
6	-0.911149000	6.085853000	1.038283000
1	-1.286828000	6.629930000	0.155134000
1	0.152428000	6.358833000	1.156529000
1	-1.459712000	6.452146000	1.923062000
6	-1.686061000	0.290352000	3.616393000
1	-1.779356000	-0.193507000	2.621134000
6	-3.120133000	0.501270000	4.161684000
1	-3.635090000	-0.467058000	4.288822000
1	-3.728802000	1.125109000	3.486563000

1	-3.086354000	1.001880000	5.145730000
6	2.524305000	2.126700000	-1.766135000
6	3.289282000	3.005814000	-0.948006000
6	3.372595000	4.360322000	-1.325258000
1	3.971930000	5.046514000	-0.717185000
6	2.716496000	4.841227000	-2.464735000
1	2.801821000	5.896196000	-2.746084000
6	1.945009000	3.966573000	-3.238098000
1	1.429400000	4.345906000	-4.126589000
6	1.827233000	2.601469000	-2.908407000
6	2.080044000	-2.555951000	1.200996000
6	1.282114000	-3.728980000	1.050491000
6	0.919319000	-4.445150000	2.208206000
1	0.305983000	-5.347211000	2.104347000
6	1.339298000	-4.039904000	3.480831000
1	1.046375000	-4.613521000	4.366741000
6	2.154122000	-2.907979000	3.611884000
1	2.507620000	-2.609968000	4.605425000
6	2.544438000	-2.151883000	2.490228000
6	3.579943000	0.072502000	-1.988214000
1	4.190706000	0.648027000	-2.704453000
6	-3.565616000	-3.059214000	2.245279000
1	-3.809062000	-2.744870000	3.275747000
1	-2.467937000	-3.068582000	2.137893000
1	-3.922525000	-4.097690000	2.126792000
6	-3.199876000	-0.639520000	-4.581650000
1	-2.717363000	0.057535000	-5.289682000
1	-4.113380000	-0.154716000	-4.199999000
1	-3.502382000	-1.536280000	-5.151452000
6	-0.297493000	4.099523000	-0.405941000
1	-0.605586000	4.663985000	-1.302832000
1	-0.454511000	3.025876000	-0.601474000
1	0.783398000	4.259239000	-0.260484000
6	-0.908132000	-0.678722000	4.524251000
1	-1.418501000	-1.656246000	4.547988000
1	-0.856969000	-0.315461000	5.566298000
1	0.118872000	-0.846619000	4.163476000
6	4.051612000	2.516847000	0.284854000
1	3.686141000	1.501425000	0.520141000
6	3.792585000	3.398204000	1.525610000
1	4.306011000	2.972739000	2.405283000
1	2.717233000	3.465594000	1.758559000
1	4.178507000	4.423991000	1.389506000
6	1.019439000	1.668347000	-3.808332000
1	0.843420000	0.734868000	-3.244149000
6	-0.358560000	2.257202000	-4.176315000
1	-0.942697000	1.524801000	-4.758800000
1	-0.267529000	3.165334000	-4.797784000
1	-0.937355000	2.518734000	-3.275193000
6	0.864188000	-4.258879000	-0.321204000
1	1.184157000	-3.518299000	-1.074916000
6	1.566263000	-5.599622000	-0.644929000
1	1.271921000	-5.950874000	-1.649553000
1	1.281439000	-6.382938000	0.080197000
1	2.663579000	-5.501109000	-0.628625000
6	3.511870000	-0.980675000	2.671680000
1	3.590814000	-0.464115000	1.698734000
6	3.020012000	0.050856000	3.708077000
1	3.740906000	0.883056000	3.789973000
1	2.921391000	-0.395116000	4.713814000
1	2.042856000	0.477939000	3.428382000
6	5.567388000	2.413144000	-0.006390000
1	6.109535000	2.039805000	0.879997000
1	5.986097000	3.401046000	-0.269045000
1	5.773903000	1.727337000	-0.845022000
6	1.817335000	1.308179000	-5.083932000
1	1.241486000	0.617013000	-5.724565000
1	2.777285000	0.823243000	-4.839245000
1	2.039849000	2.213352000	-5.676658000
6	-0.663350000	-4.416686000	-0.435381000
1	-0.943192000	-4.759077000	-1.445258000
1	-1.184139000	-3.463978000	-0.250027000
1	-1.050020000	-5.160218000	0.283960000
6	4.924492000	-1.495460000	3.039309000
1	5.634553000	-0.653666000	3.121788000
1	5.307957000	-2.193666000	2.277183000

1 4.913455000 -2.023266000 4.009793000

1 -0.392191000 -0.514001000 -1.012735000

1 0.169784000 0.561735000 0.838073000

$[2b]_2$ (IX) [Iron1-essai11-nonet]
SCF (BP86/SDD/6-31G**) Energy 333 K = -2877.86991360
Thermal correction to Gibbs Free Energy= 1.091866
Lowest Frequency = 11.4042 cm^{-1}
Second Frequency = 15.8835 cm^{-1}
SCF (B3PW91-D3,C6H6/tzvp) Energy 333 K= -5157.38999846

26 -1.336727000 0.325793000 -0.035233000

26 1.300628000 -0.254419000 -0.138391000

7 -3.148502000 -0.226895000 -0.627358000

7 -2.100626000 1.939665000 0.997408000

7 2.428075000 -1.859406000 0.099637000

7 2.742820000 0.670436000 -1.288774000

6 -4.254891000 0.589515000 -0.725590000

6 -5.415802000 0.194158000 -1.473543000

1 -5.401717000 -0.774342000 -1.977739000

6 -6.538084000 1.006488000 -1.586399000

1 -7.385999000 0.658661000 -2.187162000

6 -6.602820000 2.262451000 -0.937053000

1 -7.491051000 2.894595000 -1.023309000

6 -5.514931000 2.662706000 -0.175774000

1 -5.543068000 3.620054000 0.358791000

6 -4.333597000 1.872356000 -0.052368000

6 -3.347333000 -1.593273000 -1.040088000

6 -3.952390000 -2.505753000 -0.122094000

6 -4.245920000 -3.809507000 -0.561848000

1 -4.719752000 -4.512629000 0.132616000

6 -3.955870000 -4.219887000 -1.869222000

1 -4.205893000 -5.234425000 -2.197719000

6 -3.332912000 -3.327254000 -2.749121000

1 -3.099196000 -3.652181000 -3.769259000

6 -3.008902000 -2.011835000 -2.360573000

6 -3.327887000 2.393792000 0.825511000

1 -3.634919000 3.263084000 1.432961000

6 -1.303516000 2.621049000 1.988300000

6 -0.791235000 3.923312000 1.720537000

6 0.017817000 4.519580000 2.708252000

1 0.425376000 5.519407000 2.534026000

6 0.299296000 3.867593000 3.916650000

1 0.931650000 4.354928000 4.666345000

6 -0.248699000 2.605122000 4.173622000

1 -0.052689000 2.116224000 5.132986000

6 -1.062911000 1.959310000 3.224250000

6 3.556216000 -2.226452000 -0.588087000

6 4.124514000 -1.372042000 -1.610500000

6 5.251019000 -1.828928000 -2.356336000

1 5.652793000 -1.164983000 -3.131417000

6 5.856230000 -3.053954000 -2.111706000

1 6.717838000 -3.380588000 -2.700727000

6 5.353911000 -3.852998000 -1.056930000

1 5.837765000 -4.806621000 -0.817551000

6 4.251249000 -3.449744000 -0.311954000

1 3.882634000 -4.079824000 0.500971000

6 -4.308000000 -2.101295000 1.309672000

1 -3.947507000 -1.068983000 1.460637000

6 -5.837382000 -2.101605000 1.536226000

1 -6.077556000 -1.761145000 2.558903000

1 -6.259041000 -3.115404000 1.413553000

1 -6.347042000 -1.433656000 0.822539000

6 -2.357358000 -1.075715000 -3.378655000

1 -2.103470000 -0.138445000 -2.852366000

6 -1.041117000 -1.669792000 -3.925374000

1 -0.570739000 -0.970321000 -4.638213000

1 -1.211125000 -2.620841000 -4.460688000

1 -0.325155000 -1.857189000 -3.108141000

6 -1.111978000 4.655484000 0.411844000

1 -2.187206000 4.506245000 0.201947000

6 -0.887819000 6.178474000 0.481121000

1 -1.258423000 6.649942000 -0.444819000

1 0.183535000 6.432309000 0.566199000

1 -1.418225000 6.637217000 1.333154000

6 -1.776030000 0.649138000 3.560886000

1 -1.974374000 0.127885000 2.604007000

6 -3.145314000 0.951173000 4.218318000

1 -3.692229000 0.014434000 4.424919000

1 -3.779637000 1.580923000 3.572088000

1	-3.007258000	1.484156000	5.175941000
6	2.748712000	2.078498000	-1.633209000
6	3.385404000	2.988132000	-0.742899000
6	3.487403000	4.337036000	-1.133855000
1	3.989993000	5.048724000	-0.470827000
6	2.975485000	4.779393000	-2.360881000
1	3.075186000	5.830561000	-2.651638000
6	2.329406000	3.873196000	-3.208809000
1	1.923869000	4.224302000	-4.163851000
6	2.192618000	2.512275000	-2.867148000
6	2.042592000	-2.640510000	1.245424000
6	1.212893000	-3.789637000	1.091967000
6	0.812646000	-4.486146000	2.249379000
1	0.173924000	-5.370184000	2.143475000
6	1.223418000	-4.081559000	3.525126000
1	0.898078000	-4.637577000	4.411007000
6	2.070077000	-2.973728000	3.660186000
1	2.415665000	-2.677110000	4.656890000
6	2.500570000	-2.240904000	2.538486000
6	3.728756000	-0.013052000	-1.847190000
1	4.373802000	0.538169000	-2.552849000
6	-3.596805000	-2.998804000	2.347363000
1	-3.827292000	-2.657068000	3.372107000
1	-2.502051000	-2.980610000	2.216212000
1	-3.927383000	-4.049838000	2.269024000
6	-3.326424000	-0.726831000	-4.532265000
1	-2.848968000	-0.025116000	-5.238872000
1	-4.248001000	-0.253562000	-4.154890000
1	-3.615370000	-1.629309000	-5.100427000
6	-0.332886000	4.053533000	-0.776326000
1	-0.623970000	4.546188000	-1.720145000
1	-0.528026000	2.972569000	-0.882863000
1	0.752517000	4.188617000	-0.641926000
6	-0.955334000	-0.313633000	4.437141000
1	-1.512483000	-1.255546000	4.577101000
1	-0.762224000	0.100347000	5.442897000
1	0.010290000	-0.561830000	3.969247000
6	4.021583000	2.512815000	0.564236000

1	3.541459000	1.553449000	0.833777000
6	3.797268000	3.486850000	1.738485000
1	4.189641000	3.046692000	2.671340000
1	2.727159000	3.703919000	1.889132000
1	4.326390000	4.444543000	1.587324000
6	1.500518000	1.553803000	-3.837841000
1	1.329823000	0.602847000	-3.303751000
6	0.118426000	2.080488000	-4.280570000
1	-0.374438000	1.346827000	-4.941660000
1	0.196535000	3.026723000	-4.844438000
1	-0.537168000	2.252182000	-3.411596000
6	0.790453000	-4.311528000	-0.281067000
1	1.162155000	-3.597926000	-1.037097000
6	1.417810000	-5.693977000	-0.581585000
1	1.118743000	-6.036695000	-1.587750000
1	1.076089000	-6.452857000	0.144847000
1	2.518631000	-5.661656000	-0.549263000
6	3.505202000	-1.101975000	2.722515000
1	3.607664000	-0.592302000	1.747656000
6	3.044312000	-0.048940000	3.751444000
1	3.797333000	0.754179000	3.838081000
1	2.917156000	-0.486599000	4.757479000
1	2.087328000	0.412556000	3.456865000
6	5.531707000	2.237050000	0.366898000
1	5.985672000	1.867897000	1.303264000
1	6.060659000	3.161157000	0.072815000
1	5.709964000	1.482910000	-0.417628000
6	2.390934000	1.265096000	-5.069448000
1	1.892240000	0.553844000	-5.751046000
1	3.362581000	0.831172000	-4.778829000
1	2.595623000	2.190463000	-5.636960000
6	-0.741433000	-4.386700000	-0.415848000
1	-1.026095000	-4.724761000	-1.425646000
1	-1.212715000	-3.405848000	-0.245692000
1	-1.176913000	-5.099878000	0.306096000
6	4.896827000	-1.665718000	3.098438000
1	5.637418000	-0.850478000	3.180034000
1	5.256891000	-2.381477000	2.341049000

1	4.862212000	-2.188803000	4.070941000
1	-0.082971000	0.091238000	-1.290829000
1	0.103109000	0.017008000	1.105466000

[2b]$_2$ (V) [Iron1-essai11-quintet]
SCF (BP86/SDD/6-31G**) Energy 333 K = -2877.88563061
Thermal correction to Gibbs Free Energy= 1.101708
Lowest Frequency = 12.7935 cm^{-1}
Second Frequency = 18.9485 cm^{-1}
SCF (B3PW91-D3,C6H6/tzvp) Energy 333 K= -5157.37103150

26	-1.336727000	0.325793000	-0.035233000
26	1.300628000	-0.254419000	-0.138391000
7	-3.148502000	-0.226895000	-0.627358000
7	-2.100626000	1.939665000	0.997408000
7	2.428075000	-1.859406000	0.099637000
7	2.742820000	0.670436000	-1.288774000
6	-4.254891000	0.589515000	-0.725590000
6	-5.415802000	0.194158000	-1.473543000
1	-5.401717000	-0.774342000	-1.977739000
6	-6.538084000	1.006488000	-1.586399000
1	-7.385999000	0.658661000	-2.187162000
6	-6.602820000	2.262451000	-0.937053000
1	-7.491051000	2.894595000	-1.023309000
6	-5.514931000	2.662706000	-0.175774000
1	-5.543068000	3.620054000	0.358791000
6	-4.333597000	1.872356000	-0.052368000
6	-3.347333000	-1.593273000	-1.040088000
6	-3.952390000	-2.505753000	-0.122094000
6	-4.245920000	-3.809507000	-0.561848000
1	-4.719752000	-4.512629000	0.132616000
6	-3.955870000	-4.219887000	-1.869222000
1	-4.205893000	-5.234425000	-2.197719000
6	-3.332912000	-3.327254000	-2.749121000
1	-3.099196000	-3.652181000	-3.769259000
6	-3.008902000	-2.011835000	-2.360573000
6	-3.327887000	2.393792000	0.825511000
1	-3.634919000	3.263084000	1.432961000
6	-1.303516000	2.621049000	1.988300000
6	-0.791235000	3.923312000	1.720537000
6	0.017817000	4.519580000	2.708252000
1	0.425376000	5.519407000	2.534026000
6	0.299296000	3.867593000	3.916650000
1	0.931650000	4.354928000	4.666345000
6	-0.248699000	2.605122000	4.173622000
1	-0.052689000	2.116224000	5.132986000
6	-1.062911000	1.959310000	3.224250000
6	3.556216000	-2.226452000	-0.588087000
6	4.124514000	-1.372042000	-1.610500000
6	5.251019000	-1.828928000	-2.356336000
1	5.652793000	-1.164983000	-3.131417000
6	5.856230000	-3.053954000	-2.111706000
1	6.717838000	-3.380588000	-2.700727000
6	5.353911000	-3.852998000	-1.056930000
1	5.837765000	-4.806621000	-0.817551000
6	4.251249000	-3.449744000	-0.311954000
1	3.882634000	-4.079824000	0.500971000
6	-4.308000000	-2.101295000	1.309672000
1	-3.947507000	-1.068983000	1.460637000
6	-5.837382000	-2.101605000	1.536226000
1	-6.077556000	-1.761145000	2.558903000
1	-6.259041000	-3.115404000	1.413553000
1	-6.347042000	-1.433656000	0.822539000
6	-2.357358000	-1.075715000	-3.378655000
1	-2.103470000	-0.138445000	-2.852366000
6	-1.041117000	-1.669792000	-3.925374000
1	-0.570739000	-0.970321000	-4.638213000
1	-1.211125000	-2.620841000	-4.460688000
1	-0.325155000	-1.857189000	-3.108141000
6	-1.111978000	4.655484000	0.411844000
1	-2.187206000	4.506245000	0.201947000
6	-0.887819000	6.178474000	0.481121000
1	-1.258423000	6.649942000	-0.444819000
1	0.183535000	6.432309000	0.566199000
1	-1.418225000	6.637217000	1.333154000
6	-1.776030000	0.649138000	3.560886000
1	-1.974374000	0.127885000	2.604007000
6	-3.145314000	0.951173000	4.218318000
1	-3.692229000	0.014434000	4.424919000
1	-3.779637000	1.580923000	3.572088000

1	-3.007258000	1.484156000	5.175941000
6	2.748712000	2.078498000	-1.633209000
6	3.385404000	2.988132000	-0.742899000
6	3.487403000	4.337036000	-1.133855000
1	3.989993000	5.048724000	-0.470827000
6	2.975485000	4.779393000	-2.360881000
1	3.075186000	5.830561000	-2.651638000
6	2.329406000	3.873196000	-3.208809000
1	1.923869000	4.224302000	-4.163851000
6	2.192618000	2.512275000	-2.867148000
6	2.042592000	-2.640510000	1.245424000
6	1.212893000	-3.789637000	1.091967000
6	0.812646000	-4.486146000	2.249379000
1	0.173924000	-5.370184000	2.143475000
6	1.223418000	-4.081559000	3.525126000
1	0.898078000	-4.637577000	4.411007000
6	2.070077000	-2.973728000	3.660186000
1	2.415665000	-2.677110000	4.656890000
6	2.500570000	-2.240904000	2.538486000
6	3.728756000	-0.013052000	-1.847190000
1	4.373802000	0.538169000	-2.552849000
6	-3.596805000	-2.998804000	2.347363000
1	-3.827292000	-2.657068000	3.372107000
1	-2.502051000	-2.980610000	2.216212000
1	-3.927383000	-4.049838000	2.269024000
6	-3.326424000	-0.726831000	-4.532265000
1	-2.848968000	-0.025116000	-5.238872000
1	-4.248001000	-0.253562000	-4.154890000
1	-3.615370000	-1.629309000	-5.100427000
6	-0.332886000	4.053533000	-0.776326000
1	-0.623970000	4.546188000	-1.720145000
1	-0.528026000	2.972569000	-0.882863000
1	0.752517000	4.188617000	-0.641926000
6	-0.955334000	-0.313633000	4.437141000
1	-1.512483000	-1.255546000	4.577101000
1	-0.762224000	0.100347000	5.442897000
1	0.010290000	-0.561830000	3.969247000
6	4.021583000	2.512815000	0.564236000

1	3.541459000	1.553449000	0.833777000
6	3.797268000	3.486850000	1.738485000
1	4.189641000	3.046692000	2.671340000
1	2.727159000	3.703919000	1.889132000
1	4.326390000	4.444543000	1.587324000
6	1.500518000	1.553803000	-3.837841000
1	1.329823000	0.602847000	-3.303751000
6	0.118426000	2.080488000	-4.280570000
1	-0.374438000	1.346827000	-4.941660000
1	0.196535000	3.026723000	-4.844438000
1	-0.537168000	2.252182000	-3.411596000
6	0.790453000	-4.311528000	-0.281067000
1	1.162155000	-3.597926000	-1.037097000
6	1.417810000	-5.693977000	-0.581585000
1	1.118743000	-6.036695000	-1.587750000
1	1.076089000	-6.452857000	0.144847000
1	2.518631000	-5.661656000	-0.549263000
6	3.505202000	-1.101975000	2.722515000
1	3.607664000	-0.592302000	1.747656000
6	3.044312000	-0.048940000	3.751444000
1	3.797333000	0.754179000	3.838081000
1	2.917156000	-0.486599000	4.757479000
1	2.087328000	0.412556000	3.456865000
6	5.531707000	2.237050000	0.366898000
1	5.985672000	1.867897000	1.303264000
1	6.060659000	3.161157000	0.072815000
1	5.709964000	1.482910000	-0.417628000
6	2.390934000	1.265096000	-5.069448000
1	1.892240000	0.553844000	-5.751046000
1	3.362581000	0.831172000	-4.778829000
1	2.595623000	2.190463000	-5.636960000
6	-0.741433000	-4.386700000	-0.415848000
1	-1.026095000	-4.724761000	-1.425646000
1	-1.212715000	-3.405848000	-0.245692000
1	-1.176913000	-5.099878000	0.306096000
6	4.896827000	-1.665718000	3.098438000
1	5.637418000	-0.850478000	3.180034000
1	5.256891000	-2.381477000	2.341049000

1	4.862212000	-2.188803000	4.070941000
1	-0.082971000	0.091238000	-1.290829000
1	0.103109000	0.017008000	1.105466000

$[2b]_2$ (III) [Iron1-essai11-triplet]
SCF (BP86/SDD/6-31G**) Energy 333 K = -2877.89325081
Thermal correction to Gibbs Free Energy= 1.099928
Lowest Frequency = 12.6123 cm^{-1}
Second Frequency = 20.1343 cm^{-1}
SCF (B3PW91-D3,C6H6/tzvp) Energy 333 K= -5157.33650897

26	-1.297841000	0.273665000	0.108817000
26	1.180367000	-0.249933000	-0.295421000
7	-3.009790000	-0.151316000	-0.651573000
7	-1.997032000	1.733961000	1.183553000
7	2.401129000	-1.763911000	0.043075000
7	2.551067000	0.713042000	-1.448898000
6	-4.112716000	0.667124000	-0.711140000
6	-5.258408000	0.349209000	-1.519666000
1	-5.240408000	-0.568945000	-2.110381000
6	-6.370165000	1.178403000	-1.577208000
1	-7.207352000	0.899337000	-2.226817000
6	-6.441118000	2.369982000	-0.809829000
1	-7.322856000	3.015245000	-0.859892000
6	-5.375453000	2.686181000	0.015231000
1	-5.409914000	3.586389000	0.640807000
6	-4.204033000	1.870951000	0.088497000
6	-3.224613000	-1.495450000	-1.141708000
6	-3.856728000	-2.435579000	-0.270175000
6	-4.174213000	-3.713083000	-0.768110000
1	-4.669069000	-4.434128000	-0.107715000
6	-3.880091000	-4.075187000	-2.087748000
1	-4.146084000	-5.069975000	-2.461061000
6	-3.237650000	-3.155513000	-2.924167000
1	-3.007229000	-3.438573000	-3.957392000
6	-2.898255000	-1.862041000	-2.479217000
6	-3.213153000	2.252509000	1.034244000
1	-3.494411000	3.062504000	1.726042000
6	-1.219716000	2.328715000	2.252995000
6	-0.721628000	3.656927000	2.114953000
6	0.068066000	4.167152000	3.165504000
1	0.471891000	5.180390000	3.086593000
6	0.335160000	3.414192000	4.316109000
1	0.952999000	3.836474000	5.115728000
6	-0.212703000	2.132941000	4.453068000
1	-0.032033000	1.563712000	5.369930000
6	-1.004683000	1.568971000	3.435385000
6	3.521962000	-2.109868000	-0.665513000
6	4.036295000	-1.261599000	-1.723956000
6	5.168547000	-1.695384000	-2.479595000
1	5.530456000	-1.036113000	-3.277870000
6	5.820070000	-2.890546000	-2.219328000
1	6.683096000	-3.199533000	-2.815829000
6	5.362915000	-3.686745000	-1.139383000
1	5.882833000	-4.619067000	-0.892036000
6	4.263531000	-3.305696000	-0.381141000
1	3.931449000	-3.932766000	0.449527000
6	-4.224882000	-2.094745000	1.175326000
1	-3.834251000	-1.085063000	1.388944000
6	-5.757939000	-2.056754000	1.374024000
1	-6.006895000	-1.759431000	2.408000000
1	-6.208266000	-3.049396000	1.193404000
1	-6.232638000	-1.338650000	0.685508000
6	-2.243165000	-0.894564000	-3.463734000
1	-1.976832000	0.017950000	-2.903113000
6	-0.943419000	-1.489897000	-4.048481000
1	-0.459286000	-0.769954000	-4.730939000
1	-1.140315000	-2.408371000	-4.629162000
1	-0.227698000	-1.742068000	-3.248021000
6	-1.032483000	4.514428000	0.880680000
1	-2.106265000	4.387158000	0.650593000
6	-0.813809000	6.023912000	1.101027000
1	-1.180529000	6.582156000	0.222963000
1	0.256070000	6.272171000	1.217009000
1	-1.350857000	6.396476000	1.990225000
6	-1.704490000	0.224900000	3.636478000
1	-1.799267000	-0.248151000	2.637086000
6	-3.136339000	0.453408000	4.180504000
1	-3.665924000	-0.508312000	4.297961000
1	-3.734247000	1.091430000	3.508969000

1	-3.098623000	0.944927000	5.169059000
6	2.559663000	2.137053000	-1.735935000
6	3.334880000	2.989421000	-0.899808000
6	3.435655000	4.350037000	-1.250193000
1	4.042045000	5.016734000	-0.627683000
6	2.787876000	4.860921000	-2.381246000
1	2.886518000	5.920125000	-2.641484000
6	2.007962000	4.011207000	-3.173722000
1	1.499369000	4.414267000	-4.055705000
6	1.870953000	2.641832000	-2.870292000
6	2.048142000	-2.569578000	1.184233000
6	1.253032000	-3.741786000	1.017894000
6	0.891747000	-4.473949000	2.166188000
1	0.278765000	-5.374828000	2.050378000
6	1.312817000	-4.086199000	3.443650000
1	1.021853000	-4.672488000	4.321865000
6	2.126266000	-2.955101000	3.589267000
1	2.481307000	-2.670960000	4.586183000
6	2.512580000	-2.182288000	2.477714000
6	3.580523000	0.068073000	-1.988247000
1	4.207578000	0.643709000	-2.690032000
6	-3.565385000	-3.070227000	2.175928000
1	-3.809333000	-2.778838000	3.213002000
1	-2.467743000	-3.079119000	2.069671000
1	-3.924993000	-4.104755000	2.033120000
6	-3.218549000	-0.494381000	-4.595656000
1	-2.737248000	0.215995000	-5.291241000
1	-4.125184000	-0.012265000	-4.194736000
1	-3.532415000	-1.375257000	-5.183854000
6	-0.252110000	4.035293000	-0.360260000
1	-0.554784000	4.610372000	-1.252230000
1	-0.432125000	2.965484000	-0.559619000
1	0.832435000	4.174007000	-0.220443000
6	-0.942950000	-0.761805000	4.539319000
1	-1.467139000	-1.732239000	4.553625000
1	-0.889178000	-0.408256000	5.584672000
1	0.082255000	-0.941521000	4.178948000
6	4.089854000	2.466734000	0.323700000

1	3.714151000	1.450442000	0.538192000
6	3.839540000	3.325013000	1.582303000
1	4.348075000	2.875933000	2.452958000
1	2.764919000	3.399082000	1.816634000
1	4.236448000	4.349146000	1.467339000
6	1.053691000	1.735971000	-3.789490000
1	0.858982000	0.796779000	-3.241264000
6	-0.312503000	2.351985000	-4.156870000
1	-0.903724000	1.637526000	-4.754247000
1	-0.203632000	3.267940000	-4.763855000
1	-0.893133000	2.607683000	-3.255413000
6	0.828740000	-4.253496000	-0.358996000
1	1.175515000	-3.521251000	-1.108836000
6	1.487499000	-5.615080000	-0.685866000
1	1.189762000	-5.948842000	-1.695402000
1	1.169856000	-6.393911000	0.030271000
1	2.587415000	-5.555225000	-0.659982000
6	3.480453000	-1.013827000	2.671206000
1	3.527963000	-0.464669000	1.713992000
6	3.019478000	-0.019611000	3.756637000
1	3.737646000	0.814966000	3.838287000
1	2.961286000	-0.496402000	4.751321000
1	2.030207000	0.408179000	3.525447000
6	5.604605000	2.351937000	0.031257000
1	6.141702000	1.954755000	0.910245000
1	6.034384000	3.340176000	-0.211312000
1	5.804146000	1.680973000	-0.820984000
6	1.854241000	1.384282000	-5.065893000
1	1.271989000	0.712146000	-5.720710000
1	2.805376000	0.881034000	-4.823720000
1	2.093858000	2.295376000	-5.642683000
6	-0.702594000	-4.360704000	-0.480463000
1	-0.989882000	-4.693686000	-1.491431000
1	-1.191093000	-3.390939000	-0.296968000
1	-1.115863000	-5.090288000	0.238322000
6	4.904888000	-1.537043000	2.976780000
1	5.615882000	-0.696932000	3.068700000
1	5.266597000	-2.206811000	2.179237000

1	4.922805000	-2.099713000	3.927417000
1	-0.420432000	-0.402173000	-1.096230000
1	0.111836000	0.439573000	0.938307000

VIII (V)
SCF (BP86/SDD/6-31G**) Energy 333 K = -1787.88850899
Thermal correction to Gibbs Free Energy= 0.672851
Lowest Frequency = 11.8096 cm^{-1}
Second Frequency = 18.7475 cm^{-1}
SCF (B3PW91-D3,C6H6/tzvp) Energy 333 K= -2927.63944644

26	-0.248249000	-0.108911000	-0.830837000
1	-0.156922000	0.162104000	-2.447265000
7	0.382978000	1.582431000	0.149572000
6	-0.476076000	2.497365000	0.565386000
6	-1.900186000	2.388219000	0.678780000
6	-2.666670000	1.177903000	0.429898000
7	-2.068520000	0.027003000	0.002241000
6	-2.855933000	-1.173315000	-0.069758000
6	-3.475529000	-1.549689000	-1.298475000
6	-4.192396000	-2.762315000	-1.342516000
6	-4.304164000	-3.588523000	-0.218412000
6	-3.694348000	-3.205898000	0.983578000
6	-2.964092000	-2.006976000	1.086096000
6	-3.419204000	-0.666384000	-2.546631000
6	-2.810095000	-1.410196000	-3.754886000
6	-2.341032000	-1.610988000	2.427690000
6	-1.498179000	-2.743810000	3.055026000
6	1.778017000	1.955153000	0.171423000
6	2.356551000	2.576784000	-0.970504000
6	3.726460000	2.905497000	-0.917213000
6	4.501558000	2.630462000	0.215316000
6	3.911049000	2.018425000	1.329653000
6	2.547525000	1.666764000	1.334115000
6	1.534090000	2.940039000	-2.208473000
6	1.262013000	4.462177000	-2.254712000
6	1.911699000	1.047192000	2.580611000
6	2.814168000	-0.000132000	3.267781000
6	1.502451000	2.144961000	3.592499000
6	2.190903000	2.457855000	-3.518949000
6	-4.816428000	-0.101486000	-2.894213000
6	-3.425655000	-1.137541000	3.424895000
1	4.518461000	1.815302000	2.217705000
1	5.563416000	2.897933000	0.233619000
1	4.189490000	3.391852000	-1.782467000
1	-3.789180000	-3.850850000	1.864399000
1	-4.867362000	-4.526119000	-0.276367000
1	-4.676795000	-3.058773000	-2.279823000
1	-1.667091000	-0.757304000	2.235080000
1	0.986010000	0.536850000	2.256224000
1	2.256403000	-0.512348000	4.070344000
1	3.176664000	-0.763480000	2.558651000
1	3.698630000	0.465304000	3.737282000
1	1.028878000	1.696617000	4.483501000
1	2.386933000	2.715704000	3.926509000
1	0.788095000	2.860204000	3.152323000
1	-1.015876000	-2.390359000	3.983065000
1	-2.118671000	-3.617006000	3.322683000
1	-0.704836000	-3.096184000	2.373982000
1	-2.966219000	-0.819254000	4.377512000
1	-3.998795000	-0.287617000	3.020624000
1	-4.136335000	-1.953313000	3.648540000
1	0.564422000	2.417980000	-2.133060000
1	0.637770000	4.717182000	-3.128839000
1	0.736816000	4.809440000	-1.348558000
1	2.204367000	5.033531000	-2.332731000
1	1.535949000	2.692033000	-4.375735000
1	3.163919000	2.946720000	-3.703612000
1	2.348619000	1.367155000	-3.503901000
1	-2.752967000	0.185088000	-2.323578000
1	-4.753881000	0.566582000	-3.771149000
1	-5.528677000	-0.909987000	-3.138754000
1	-5.236324000	0.477874000	-2.055204000
1	-2.762020000	-0.739374000	-4.630306000
1	-1.783267000	-1.745288000	-3.533701000
1	-3.410941000	-2.291959000	-4.040938000
6	0.277097000	-2.176777000	-0.563699000
1	0.165415000	-2.588408000	-1.574995000
1	-0.429714000	-2.547291000	0.185594000

1	-0.055591000	3.471039000	0.871759000
6	-4.082048000	1.253157000	0.668207000
1	-4.679926000	0.354860000	0.494824000
6	-4.696221000	2.424959000	1.089434000
6	-2.576682000	3.566587000	1.117136000
1	-5.781115000	2.430324000	1.244533000
1	-1.970936000	4.462821000	1.298484000
6	-3.947662000	3.606776000	1.318302000
1	-4.439258000	4.525313000	1.650461000
6	1.423610000	-1.474517000	-0.208890000
1	1.604838000	-1.260014000	0.851765000
6	2.611339000	-1.280307000	-1.137579000
1	2.293816000	-1.482834000	-2.176723000
1	2.960382000	-0.233236000	-1.102074000
6	3.765116000	-2.201519000	-0.746525000
6	3.702051000	-3.585497000	-1.010021000
6	4.905587000	-1.689015000	-0.096725000
6	4.754972000	-4.434871000	-0.640017000
6	5.960466000	-2.538204000	0.277261000
6	5.888670000	-3.913419000	0.006503000
1	2.818872000	-3.998168000	-1.511570000
1	4.965415000	-0.613769000	0.109295000
1	4.692184000	-5.506398000	-0.858686000
1	6.841948000	-2.122124000	0.777227000
1	6.711493000	-4.576070000	0.294766000

VIII (III)
SCF (BP86/SDD/6-31G**) Energy 333 K = -1787.89760116
Thermal correction to Gibbs Free Energy= 0.676742
Lowest Frequency = 8.3870 cm^{-1}
Second Frequency = 15.8876 cm^{-1}
SCF (B3PW91-D3,C6H6/tzvp) Energy 333 K= -2927.62639902

1	-0.957076000	-0.085105000	-1.975175000
6	0.837497000	0.934233000	-2.168448000
1	0.953810000	0.290957000	-3.047627000
6	1.676801000	0.773890000	-1.041747000
26	-0.215435000	-0.029704000	-0.668849000
1	2.408661000	-0.044690000	-1.053800000
7	0.393822000	-1.577280000	0.390513000
7	-1.844563000	0.219079000	0.287164000
6	-0.342968000	-2.122934000	1.354318000
6	1.623493000	-2.280383000	0.087709000
1	0.052776000	-3.037207000	1.825735000
6	-1.612160000	-1.682252000	1.836588000
6	-2.352199000	-0.556082000	1.300844000
6	-2.181775000	-2.447577000	2.900593000
6	-3.636660000	-0.287232000	1.883689000
6	-3.419680000	-2.152141000	3.447453000
1	-1.601579000	-3.295850000	3.283362000
1	-4.215370000	0.553112000	1.493159000
6	-4.146570000	-1.053376000	2.923547000
6	-2.688065000	1.265610000	-0.246588000
1	-5.128489000	-0.801135000	3.339090000
1	-3.829484000	-2.755196000	4.262731000
6	2.750184000	-2.147904000	0.947740000
6	1.673539000	-3.096553000	-1.078098000
6	3.922033000	-2.857460000	0.617297000
6	2.720850000	-1.290141000	2.215738000
6	2.871400000	-3.782222000	-1.356522000
6	0.449282000	-3.298635000	-1.972873000
1	4.798043000	-2.770888000	1.268872000
6	3.989463000	-3.667712000	-0.521503000
1	1.825267000	-0.645642000	2.162316000
6	3.954504000	-0.368570000	2.337772000
6	2.592787000	-2.170008000	3.482656000
1	4.911235000	-4.209821000	-0.757576000
1	2.924321000	-4.423553000	-2.242538000
1	-0.245496000	-2.461635000	-1.771108000
6	-0.277275000	-4.614796000	-1.607568000
6	0.785040000	-3.258066000	-3.477832000
1	3.842206000	0.299655000	3.209564000
1	4.093289000	0.257436000	1.441447000
1	4.883282000	-0.945400000	2.491487000
1	1.681182000	-2.789885000	3.463325000
1	2.557803000	-1.542338000	4.390421000
1	3.457277000	-2.851239000	3.574034000
1	0.379594000	-5.487200000	-1.775371000
1	-1.182277000	-4.745900000	-2.226566000
1	-0.584823000	-4.625161000	-0.548418000

1	1.322016000	-2.332963000	-3.747605000
1	-0.143355000	-3.300085000	-4.072987000
1	1.409930000	-4.113523000	-3.790043000
6	-3.651064000	0.963391000	-1.255179000
6	-2.539054000	2.598405000	0.241630000
6	-4.443941000	2.010936000	-1.760175000
6	-3.858953000	-0.453031000	-1.794608000
6	-3.364719000	3.605377000	-0.296572000
6	-1.546872000	2.947757000	1.353144000
1	-5.186213000	1.789534000	-2.535521000
6	-4.310176000	3.322488000	-1.289003000
1	-3.105537000	-1.104303000	-1.320936000
6	-5.257284000	-1.000288000	-1.424960000
6	-3.631198000	-0.518645000	-3.321532000
1	-4.941157000	4.121411000	-1.693043000
1	-3.263745000	4.631547000	0.072007000
1	-0.793782000	2.139133000	1.379372000
6	-2.241190000	2.984428000	2.736384000
6	-0.807448000	4.279559000	1.100461000
1	-5.409337000	-1.007175000	-0.333193000
1	-5.375357000	-2.035017000	-1.792386000
1	-6.060050000	-0.390911000	-1.877263000
1	-4.362947000	0.100627000	-3.869817000
1	-3.738317000	-1.556885000	-3.682088000
1	-2.620674000	-0.164676000	-3.587029000
1	-3.020710000	3.767233000	2.759184000
1	-1.509425000	3.212735000	3.531742000
1	-2.718881000	2.021510000	2.978015000
1	-0.364639000	4.321119000	0.090815000
1	0.002973000	4.411692000	1.837929000
1	-1.480432000	5.149174000	1.203402000
1	0.350576000	1.897941000	-2.367317000
6	1.992162000	1.917693000	-0.092108000
1	1.188393000	2.675638000	-0.164404000
1	1.986447000	1.569401000	0.958204000
6	3.336044000	2.602383000	-0.358915000
6	4.073510000	3.148240000	0.712494000
6	3.850751000	2.740913000	-1.663708000
6	5.285733000	3.819339000	0.488835000
6	5.064155000	3.409296000	-1.890828000
6	5.786596000	3.952005000	-0.816121000
1	3.689157000	3.046123000	1.734468000
1	3.294891000	2.316966000	-2.506691000
1	5.841770000	4.234437000	1.336543000
1	5.447003000	3.504888000	-2.912838000
1	6.734298000	4.471212000	-0.993585000

VIII’ (III)
SCF (BP86/SDD/6-31G**) Energy 333 K = -1787.89451770
Thermal correction to Gibbs Free Energy= 0.677374
Lowest Frequency = 11.1572 cm^{-1}
Second Frequency = 13.7851 cm^{-1}
SCF (B3PW91-D3,C6H6/tzvp) Energy 333 K= -2927.62465537

26	0.424294000	-0.113292000	-0.692639000
1	1.214140000	-0.405262000	-1.943255000
7	2.089317000	-0.054063000	0.257090000
6	2.532969000	0.996081000	0.936543000
6	1.833828000	2.216436000	1.170940000
6	0.459842000	2.465844000	0.770824000
7	-0.302751000	1.514329000	0.131550000
6	-1.687621000	1.853055000	-0.093401000
6	-2.082248000	2.379086000	-1.359763000
6	-3.440683000	2.689394000	-1.568585000
6	-4.397491000	2.488935000	-0.567407000
6	-3.999932000	1.965507000	0.669585000
6	-2.655638000	1.638256000	0.936774000
6	-1.070755000	2.647493000	-2.476780000
6	-1.504175000	2.031969000	-3.825106000
6	-2.275739000	1.097315000	2.319892000
6	-3.271488000	0.043183000	2.855679000
6	2.998245000	-1.173143000	0.117998000
6	3.976520000	-1.162241000	-0.913591000
6	4.838766000	-2.271472000	-1.015532000
6	4.742851000	-3.354192000	-0.133899000
6	3.771876000	-3.344665000	0.875601000
6	2.881336000	-2.264205000	1.024415000
6	4.132734000	0.007967000	-1.886231000
6	5.448029000	0.777651000	-1.623742000
6	1.861988000	-2.246420000	2.164933000

6	1.258170000	-3.633484000	2.466254000
6	2.483199000	-1.644389000	3.448727000
6	4.045487000	-0.450103000	-3.358754000
6	-0.798973000	4.162199000	-2.634195000
6	-2.145933000	2.235495000	3.362706000
1	3.704027000	-4.192507000	1.564611000
1	5.425681000	-4.204930000	-0.230993000
1	5.603044000	-2.280510000	-1.800550000
1	-4.750375000	1.810894000	1.452325000
1	-5.448335000	2.738644000	-0.748787000
1	-3.750175000	3.100927000	-2.535784000
1	-1.285592000	0.616401000	2.219273000
1	1.035803000	-1.581225000	1.850638000
1	0.431184000	-3.537059000	3.190182000
1	0.862428000	-4.114675000	1.556024000
1	1.999807000	-4.318826000	2.913387000
1	1.738995000	-1.612661000	4.263880000
1	3.338179000	-2.254557000	3.790387000
1	2.847326000	-0.617304000	3.282000000
1	-2.872699000	-0.417781000	3.776053000
1	-4.242001000	0.498649000	3.121226000
1	-3.471594000	-0.761548000	2.129559000
1	-1.856582000	1.824277000	4.346173000
1	-1.391885000	2.980872000	3.067978000
1	-3.112715000	2.756198000	3.486513000
1	3.294942000	0.704153000	-1.713027000
1	5.535829000	1.639970000	-2.307606000
1	5.495209000	1.156916000	-0.588788000
1	6.328792000	0.130218000	-1.781598000
1	4.103766000	0.421103000	-4.034282000
1	4.870658000	-1.133074000	-3.626804000
1	3.094613000	-0.973186000	-3.555165000
1	-0.122324000	2.162396000	-2.181148000
1	-0.043828000	4.341541000	-3.419994000
1	-1.719554000	4.701603000	-2.921767000
1	-0.425660000	4.603483000	-1.695647000
1	-0.707130000	2.161900000	-4.577935000
1	-1.713057000	0.952946000	-3.729962000
1	-2.414143000	2.513411000	-4.225071000
6	-0.420876000	-1.475637000	-2.013350000
1	0.142987000	-2.417729000	-1.993629000
1	-0.547848000	-1.048430000	-3.014360000
1	3.551139000	0.925904000	1.352529000
6	-0.055744000	3.776872000	1.059492000
1	-1.078407000	4.007244000	0.752631000
6	0.704210000	4.750862000	1.693627000
6	2.569069000	3.238712000	1.846910000
1	0.261423000	5.737059000	1.872543000
1	3.598283000	3.008303000	2.147601000
6	2.033172000	4.487898000	2.110720000
1	2.622334000	5.254870000	2.621400000
6	-1.341232000	-1.177504000	-0.980790000
1	-2.132907000	-0.448639000	-1.196556000
6	-1.656859000	-2.162874000	0.138649000
1	-0.791953000	-2.830064000	0.302862000
1	-1.821178000	-1.617240000	1.085840000
6	-2.889557000	-3.008577000	-0.175381000
6	-4.166657000	-2.413982000	-0.263265000
6	-2.778661000	-4.394826000	-0.399041000
6	-5.299696000	-3.186502000	-0.559414000
6	-3.910771000	-5.171091000	-0.696199000
6	-5.176029000	-4.569070000	-0.776798000
1	-4.272207000	-1.334384000	-0.102393000
1	-1.793281000	-4.871717000	-0.334867000
1	-6.282917000	-2.707277000	-0.620180000
1	-3.802667000	-6.248266000	-0.863959000
1	-6.060381000	-5.172301000	-1.007622000

TS(VIII-IX) (V)
SCF (BP86/SDD/6-31G**) Energy 333 K = -1787.86752926
Thermal correction to Gibbs Free Energy= 0.669661
Lowest Frequency = -818.3964 cm^{-1}
Second Frequency = 8.4847 cm^{-1}
SCF (B3PW91-D3,C6H6/tzvp) Energy 333 K= -2927.61342604

1	0.574789000	-0.595291000	-2.084443000
6	-0.624358000	-1.730928000	-1.756001000
6	-1.179491000	-1.732242000	-0.422713000
1	-0.787896000	-2.498942000	0.263408000
26	0.257642000	-0.199306000	-0.514741000

6	-2.610268000	-1.289161000	-0.164694000
7	2.148817000	-0.283039000	0.194360000
6	2.853775000	0.803643000	0.431567000
1	3.915905000	0.666085000	0.700314000
6	2.399841000	2.167682000	0.378674000
6	1.024494000	2.589038000	0.160143000
6	3.401793000	3.154638000	0.612112000
6	0.769420000	3.999373000	0.215563000
7	0.014637000	1.686820000	-0.052069000
1	-0.258866000	4.339813000	0.068752000
6	1.781766000	4.925846000	0.440893000
1	1.530635000	5.992335000	0.463325000
6	3.120773000	4.514141000	0.639298000
1	3.912881000	5.246910000	0.815928000
1	4.428952000	2.807147000	0.776904000
6	2.800198000	-1.564239000	0.264611000
6	3.641555000	-1.987951000	-0.801766000
6	2.528633000	-2.402944000	1.383379000
6	4.234728000	-3.262585000	-0.700239000
6	3.919464000	-1.118334000	-2.029888000
1	4.894052000	-3.606824000	-1.504714000
6	3.996198000	-4.095088000	0.398690000
1	4.468976000	-5.081355000	0.453822000
6	3.145968000	-3.665738000	1.427480000
1	2.960641000	-4.323383000	2.282502000
6	1.646577000	-1.912218000	2.531999000
1	3.260853000	-0.234713000	-1.975616000
6	3.572395000	-1.851264000	-3.344816000
6	5.385237000	-0.625757000	-2.047197000
1	3.733784000	-1.181805000	-4.207557000
1	2.516561000	-2.168490000	-3.353462000
1	4.200468000	-2.746616000	-3.497097000
1	5.633621000	-0.054689000	-1.136319000
1	5.565421000	0.026913000	-2.919181000
1	6.090652000	-1.473395000	-2.108982000
1	0.936447000	-1.176478000	2.105483000
6	2.496647000	-1.172044000	3.592785000
6	0.812360000	-3.029281000	3.189584000

1	3.242313000	-1.854255000	4.038172000
1	1.856902000	-0.786780000	4.405984000
1	3.040713000	-0.318703000	3.154704000
1	0.230217000	-3.596929000	2.444435000
1	0.105179000	-2.593352000	3.915416000
1	1.443633000	-3.745607000	3.744585000
6	-1.347747000	2.139637000	-0.058884000
6	-2.041405000	2.297989000	1.179272000
6	-2.012245000	2.373461000	-1.301902000
6	-3.390544000	2.697467000	1.146021000
6	-1.357383000	2.064283000	2.527938000
1	-3.931965000	2.825885000	2.090378000
6	-4.052541000	2.933202000	-0.065482000
1	-5.103004000	3.243136000	-0.068476000
6	-3.363611000	2.770364000	-1.273892000
1	-3.884550000	2.958180000	-2.218760000
6	-1.280237000	2.248356000	-2.640633000
1	-0.343595000	1.680560000	2.321604000
6	-1.211277000	3.384431000	3.320379000
6	-2.091074000	1.001234000	3.375682000
1	-0.641891000	4.134357000	2.747456000
1	-0.681183000	3.207564000	4.272875000
1	-2.198914000	3.816699000	3.561410000
1	-3.118067000	1.318095000	3.629369000
1	-1.554437000	0.830811000	4.325581000
1	-2.157906000	0.037214000	2.843991000
1	-0.375759000	1.633978000	-2.467665000
6	-0.809317000	3.632157000	-3.149474000
6	-2.119962000	1.538476000	-3.723819000
1	-1.672854000	4.298643000	-3.326201000
1	-0.260421000	3.528060000	-4.102173000
1	-0.141444000	4.124517000	-2.424293000
1	-2.508049000	0.568303000	-3.370219000
1	-1.503202000	1.354871000	-4.620460000
1	-2.984128000	2.147417000	-4.043669000
1	-1.253782000	-1.391342000	-2.588205000
1	-2.699636000	-0.790080000	0.818161000
1	-2.884397000	-0.505638000	-0.900768000

6	-3.667625000	-2.397622000	-0.222243000
6	-3.463089000	-3.599720000	-0.926555000
6	-4.903343000	-2.212146000	0.433887000
6	-4.463917000	-4.584108000	-0.981213000
6	-5.906225000	-3.191178000	0.381214000
6	-5.690154000	-4.384385000	-0.328633000
1	-2.504878000	-3.765999000	-1.430299000
1	-5.077064000	-1.283901000	0.992190000
1	-4.281999000	-5.512337000	-1.534505000
1	-6.856919000	-3.025153000	0.900179000
1	-6.469574000	-5.152826000	-0.368033000
1	0.073845000	-2.526034000	-2.045375000

TS(VIII-IX$_0$) (III)
SCF (BP86/SDD/6-31G**) Energy 333 K = -1787.89604044
Thermal correction to Gibbs Free Energy= 0.675952
Lowest Frequency = -469.2209 cm^{-1}
Second Frequency = 10.7801 cm^{-1}
SCF (B3PW91-D3,C6H6/tzvp) Energy 333 K= -2927.62635695

1	-0.716889000	0.431087000	-1.960153000
6	0.805855000	1.059243000	-2.101751000
26	-0.174124000	0.011999000	-0.596268000
1	0.952866000	0.505788000	-3.038182000
6	1.673669000	0.799158000	-0.987834000
1	0.432973000	2.081195000	-2.258456000
1	2.399394000	-0.018708000	-1.091115000
6	2.075083000	1.901247000	-0.019412000
7	0.348460000	-1.585646000	0.379457000
7	-1.886000000	0.191190000	0.257738000
6	-0.441143000	-2.198266000	1.260377000
6	1.597618000	-2.266660000	0.102220000
6	-2.448363000	-0.636689000	1.193875000
6	-2.683847000	1.294491000	-0.225054000
1	-0.061405000	-3.138420000	1.691715000
6	-1.737897000	-1.797974000	1.695590000
6	2.689758000	-2.144637000	1.005757000
6	1.697918000	-3.050882000	-1.081173000
6	-2.364882000	-2.633353000	2.671848000
6	-3.764644000	-0.404719000	1.722045000
6	-3.632478000	-2.373821000	3.164829000
1	-1.804661000	-3.505690000	3.029795000
1	-4.322898000	0.460638000	1.356853000
6	-4.330862000	-1.239994000	2.675148000
1	-4.087480000	-3.029593000	3.912658000
1	-5.336029000	-1.016208000	3.049655000
6	-3.577776000	1.098385000	-1.319268000
6	-2.553669000	2.577118000	0.386353000
6	3.878343000	-2.838621000	0.703750000
6	2.599488000	-1.311943000	2.287102000
6	2.910393000	-3.721830000	-1.331547000
6	0.512631000	-3.225321000	-2.032465000
1	4.729349000	-2.761110000	1.388567000
6	3.993935000	-3.621898000	-0.450405000
1	1.723395000	-0.645880000	2.186709000
6	3.843248000	-0.423232000	2.509104000
6	2.367890000	-2.211361000	3.525295000
1	3.002840000	-4.339080000	-2.231423000
1	-0.197118000	-2.402126000	-1.823591000
6	-0.219698000	-4.559085000	-1.753445000
6	0.910940000	-3.119961000	-3.519222000
1	4.927652000	-4.153011000	-0.663458000
1	3.682171000	0.241432000	3.375914000
1	4.065567000	0.204312000	1.630655000
1	4.742962000	-1.024608000	2.728282000
1	1.441459000	-2.802245000	3.437354000
1	2.294458000	-1.599963000	4.441856000
1	3.205592000	-2.919365000	3.655130000
1	0.450215000	-5.419859000	-1.928939000
1	-1.096609000	-4.669363000	-2.415436000
1	-0.571674000	-4.614860000	-0.709785000
1	1.450555000	-2.180665000	-3.728197000
1	0.009690000	-3.146465000	-4.155689000
1	1.557753000	-3.956544000	-3.837401000
6	-4.331346000	2.195446000	-1.776660000
6	-3.743625000	-0.261393000	-1.999941000
6	-3.335786000	3.638929000	-0.110436000
6	-1.619020000	2.813711000	1.574847000
1	-5.023226000	2.053943000	-2.614945000
6	-4.220492000	3.457518000	-1.179907000

1	-3.016320000	-0.951893000	-1.540101000
6	-5.157789000	-0.842667000	-1.770160000
6	-3.419625000	-0.185884000	-3.508841000
1	-3.248927000	4.627578000	0.352736000
1	-0.888593000	1.983714000	1.579910000
6	-2.389539000	2.768628000	2.916522000
6	-0.831397000	4.137005000	1.462376000
1	-4.820175000	4.296954000	-1.547863000
1	-5.375703000	-0.950043000	-0.694916000
1	-5.247315000	-1.839117000	-2.237937000
1	-5.934996000	-0.193902000	-2.212357000
1	-4.120374000	0.479104000	-4.043880000
1	-3.493930000	-1.186512000	-3.970028000
1	-2.398051000	0.195225000	-3.678389000
1	-3.147097000	3.572098000	2.956934000
1	-1.698622000	2.912396000	3.766336000
1	-2.906858000	1.805908000	3.055590000
1	-0.321513000	4.231034000	0.488519000
1	-0.068413000	4.195464000	2.257830000
1	-1.487271000	5.017759000	1.580085000
1	1.293811000	2.686913000	-0.024317000
1	2.101196000	1.515697000	1.017422000
6	3.427736000	2.555980000	-0.314811000
6	4.185697000	3.105987000	0.740504000
6	3.935898000	2.659378000	-1.625017000
6	5.409978000	3.746531000	0.495551000
1	3.807620000	3.030552000	1.767213000
6	5.161502000	3.297565000	-1.874140000
1	3.366549000	2.230495000	-2.456553000
6	5.903525000	3.844689000	-0.815463000
1	5.981619000	4.164713000	1.331311000
1	5.538422000	3.365478000	-2.900591000
1	6.860682000	4.340038000	-1.009571000

TS(VIII'-IX$_0$') (III)
SCF (BP86/SDD/6-31G**) Energy 333 K = -1787.89433536
Thermal correction to Gibbs Free Energy= 0.676586
Lowest Frequency = -260.3981 cm^{-1}
Second Frequency = 10.6662 cm^{-1}
SCF (B3PW91-D3,C6H6/tzvp) Energy 333 K= -2927.62496511

26	-0.388533000	0.135157000	-0.643790000
1	-1.094607000	0.671325000	-1.876433000
7	-2.094714000	0.059080000	0.244047000
6	-2.560721000	-0.996753000	0.898641000
6	-1.876287000	-2.227129000	1.124994000
6	-0.497720000	-2.479482000	0.744935000
7	0.283362000	-1.521694000	0.138227000
6	1.665790000	-1.872317000	-0.086885000
6	2.056363000	-2.397292000	-1.354626000
6	3.412057000	-2.718596000	-1.564668000
6	4.369838000	-2.531248000	-0.561961000
6	3.976166000	-2.009570000	0.677110000
6	2.634727000	-1.671152000	0.944716000
6	1.044110000	-2.650277000	-2.474461000
6	1.470722000	-2.000086000	-3.808832000
6	2.256231000	-1.131399000	2.328374000
6	3.277408000	-0.113482000	2.885589000
6	-2.991395000	1.189051000	0.115410000
6	-3.933414000	1.218952000	-0.948911000
6	-4.788883000	2.333889000	-1.041873000
6	-4.718346000	3.386076000	-0.121266000
6	-3.778744000	3.339718000	0.916404000
6	-2.897176000	2.250603000	1.058132000
6	-4.053654000	0.082496000	-1.965671000
6	-5.364117000	-0.712159000	-1.759967000
6	-1.907429000	2.196005000	2.223362000
6	-1.290416000	3.568434000	2.563295000
6	-2.568243000	1.580188000	3.480307000
6	-3.939894000	0.591570000	-3.419478000
6	0.784460000	-4.163019000	-2.665975000
6	2.078022000	-2.273958000	3.359231000
1	-3.728447000	4.165252000	1.633587000
1	-5.395419000	4.242180000	-0.211243000
1	-5.526950000	2.372719000	-1.850720000
1	4.727690000	-1.864937000	1.460606000
1	5.418513000	-2.789798000	-0.743574000
1	3.718312000	-3.128815000	-2.533577000
1	1.282293000	-0.619654000	2.220039000
1	-1.082901000	1.525086000	1.916707000

1	-0.486302000	3.447688000	3.309094000
1	-0.860675000	4.055769000	1.671918000
1	-2.033351000	4.259668000	2.999033000
1	-1.844664000	1.521861000	4.312401000
1	-3.422232000	2.196733000	3.812979000
1	-2.943492000	0.562095000	3.285314000
1	2.879064000	0.356410000	3.801593000
1	4.227816000	-0.601990000	3.164925000
1	3.515907000	0.687481000	2.166684000
1	1.790713000	-1.862272000	4.343116000
1	1.304251000	-2.992541000	3.049430000
1	3.026298000	-2.826355000	3.488865000
1	-3.210605000	-0.608841000	-1.796348000
1	-5.427487000	-1.549675000	-2.476647000
1	-5.427737000	-1.129862000	-0.740900000
1	-6.248849000	-0.068779000	-1.912349000
1	-3.966072000	-0.257087000	-4.125038000
1	-4.770976000	1.268210000	-3.685232000
1	-2.995172000	1.138829000	-3.576538000
1	0.092411000	-2.180150000	-2.166043000
1	0.028587000	-4.330510000	-3.453690000
1	1.708232000	-4.688612000	-2.968287000
1	0.417206000	-4.628174000	-1.736655000
1	0.677644000	-2.125247000	-4.566747000
1	1.664006000	-0.920474000	-3.689767000
1	2.388954000	-2.459478000	-4.215674000
6	0.434713000	1.522868000	-1.959372000
1	-0.065786000	2.500389000	-1.930324000
1	0.576474000	1.134321000	-2.975172000
1	-3.585824000	-0.924972000	1.297414000
6	0.004154000	-3.798584000	1.020457000
1	1.031124000	-4.030805000	0.729788000
6	-0.773366000	-4.777222000	1.625750000
6	-2.630638000	-3.255139000	1.770008000
1	-0.339716000	-5.768715000	1.797772000
1	-3.663544000	-3.022254000	2.055937000
6	-2.108049000	-4.512388000	2.022655000
1	-2.711427000	-5.283644000	2.509747000
6	1.360585000	1.179246000	-0.930923000
1	2.147488000	0.455299000	-1.179195000
6	1.710515000	2.152808000	0.190202000
1	0.850545000	2.817572000	0.388682000
1	1.899714000	1.593704000	1.124452000
6	2.934534000	3.002417000	-0.144438000
6	4.211826000	2.410884000	-0.248537000
6	2.816703000	4.387408000	-0.372467000
6	5.338210000	3.184605000	-0.565775000
6	3.942113000	5.165067000	-0.691042000
6	5.207493000	4.565810000	-0.788300000
1	4.322017000	1.332374000	-0.083023000
1	1.831450000	4.862519000	-0.293678000
1	6.321829000	2.707829000	-0.638462000
1	3.828863000	6.241276000	-0.861677000
1	6.086667000	5.170196000	-1.035328000

IX (V)
SCF (BP86/SDD/6-31G**) Energy 333 K = -1787.91494969
Thermal correction to Gibbs Free Energy= 0.672231
Lowest Frequency = 7.7858 cm^{-1}
Second Frequency = 11.1165 cm^{-1}
SCF (B3PW91-D3,C6H6/tzvp) Energy 333 K= -2927.66136845

6	-1.515092000	-1.308775000	0.702181000
1	-2.338872000	-0.600537000	0.948717000
6	0.806223000	2.731652000	-0.300193000
1	0.570160000	3.802997000	-0.418501000
6	2.182572000	2.374671000	-0.470308000
6	2.721614000	1.026709000	-0.371667000
6	4.131644000	0.874869000	-0.592090000
1	4.555424000	-0.130670000	-0.525743000
6	4.954371000	1.956077000	-0.879721000
1	6.025101000	1.784993000	-1.037609000
6	4.428950000	3.269404000	-0.970230000
1	5.081029000	4.117781000	-1.196630000
6	3.070688000	3.454250000	-0.767661000
1	2.640405000	4.460872000	-0.835434000
6	-1.524552000	2.490548000	0.074097000
6	-2.059633000	2.766054000	1.363929000
6	-3.354640000	3.311335000	1.440673000
1	-3.780675000	3.542449000	2.422472000

6	-4.106779000	3.565547000	0.286194000
1	-5.111785000	3.992588000	0.368618000
6	-3.571441000	3.266061000	-0.972017000
1	-4.166475000	3.459489000	-1.871406000
6	-2.280708000	2.718323000	-1.108863000
6	-1.237374000	2.530504000	2.631732000
1	-0.438928000	1.808314000	2.373121000
6	-2.059650000	1.913750000	3.782017000
1	-1.397860000	1.665177000	4.629508000
1	-2.571984000	0.990919000	3.462957000
1	-2.825006000	2.612028000	4.163708000
6	-0.544779000	3.836628000	3.087690000
1	0.106515000	4.245758000	2.297316000
1	0.075361000	3.658605000	3.983767000
1	-1.294213000	4.608120000	3.338807000
6	-1.732333000	2.398736000	-2.500969000
1	-0.788146000	1.841551000	-2.369247000
6	-1.405047000	3.691545000	-3.283900000
1	-2.312928000	4.300925000	-3.439149000
1	-0.984980000	3.451071000	-4.276201000
1	-0.672725000	4.316043000	-2.745012000
6	-2.689416000	1.495898000	-3.310545000
1	-2.920147000	0.564092000	-2.767122000
1	-2.232835000	1.224340000	-4.278158000
1	-3.645838000	2.002465000	-3.528432000
6	2.507171000	-1.361797000	-0.013064000
6	2.573442000	-2.168159000	-1.189446000
6	3.102296000	-3.468612000	-1.082405000
1	3.161829000	-4.095574000	-1.979201000
6	3.557691000	-3.972927000	0.142015000
1	3.966870000	-4.987061000	0.202380000
6	3.490289000	-3.171256000	1.288443000
1	3.852984000	-3.566695000	2.243681000
6	2.970358000	-1.863693000	1.241015000
6	2.125365000	-1.644810000	-2.555901000
1	1.621432000	-0.676133000	-2.387531000
6	3.339439000	-1.384383000	-3.479081000
1	4.040504000	-0.664026000	-3.027193000

1	3.007683000	-0.975534000	-4.449887000
1	3.891941000	-2.319996000	-3.678579000
6	1.113936000	-2.585948000	-3.246265000
1	1.563594000	-3.566262000	-3.483078000
1	0.769104000	-2.145321000	-4.198157000
1	0.229878000	-2.766797000	-2.612767000
6	2.953979000	-1.007663000	2.509479000
1	2.331764000	-0.119325000	2.296350000
6	4.375283000	-0.506826000	2.861704000
1	5.049257000	-1.356237000	3.073577000
1	4.348815000	0.135318000	3.759899000
1	4.812713000	0.078218000	2.036585000
6	2.327954000	-1.741503000	3.715390000
1	1.315977000	-2.111966000	3.484304000
1	2.257401000	-1.059945000	4.581235000
1	2.939210000	-2.605288000	4.031197000
26	-0.006919000	-0.043977000	0.254128000
7	-0.205594000	1.917218000	-0.023759000
7	1.920509000	-0.049641000	-0.082748000
6	-1.230711000	-2.189263000	1.929358000
1	-0.376428000	-2.868430000	1.749577000
1	-0.990220000	-1.582196000	2.820826000
1	-2.099259000	-2.825635000	2.198358000
6	-1.957655000	-2.136461000	-0.536104000
1	-2.033118000	-1.475771000	-1.422882000
1	-1.179514000	-2.889115000	-0.767223000
6	-3.294165000	-2.845466000	-0.351734000
6	-4.505393000	-2.131905000	-0.477411000
6	-3.359229000	-4.213264000	-0.015808000
6	-5.741286000	-2.763383000	-0.274982000
6	-4.594462000	-4.850014000	0.190643000
6	-5.790404000	-4.127046000	0.061668000
1	-4.474662000	-1.067367000	-0.740719000
1	-2.428767000	-4.785985000	0.077481000
1	-6.669509000	-2.191637000	-0.384678000
1	-4.621061000	-5.914498000	0.448757000
1	-6.754619000	-4.622182000	0.218374000

IX_0 (III)

SCF (BP86/SDD/6-31G**) Energy 333 K = -1787.90841782
Thermal correction to Gibbs Free Energy= 0.677388
Lowest Frequency = 7.0016 cm^{-1}
Second Frequency = 13.3773 cm^{-1}
SCF (B3PW91-D3,C6H6/tzvp) Energy 333 K= -2927.64267928

1	-0.488557000	1.705142000	-1.066803000
6	0.604468000	2.119679000	-1.048697000
26	-0.081239000	0.081275000	-0.419733000
1	0.741337000	2.407266000	-2.104715000
6	1.597070000	1.109542000	-0.530149000
1	0.491825000	3.023297000	-0.427270000
1	2.215662000	0.671447000	-1.334118000
6	2.449066000	1.468318000	0.690166000
7	0.365340000	-1.680946000	0.032085000
7	-1.955862000	-0.010122000	0.094886000
6	-0.508520000	-2.562844000	0.527112000
6	1.727714000	-2.145409000	-0.122818000
6	-2.602636000	-1.116057000	0.583471000
6	-2.739573000	1.185396000	-0.094024000
1	-0.114269000	-3.568347000	0.738005000
6	-1.892502000	-2.364631000	0.798173000
6	2.530416000	-2.404701000	1.025502000
6	2.241595000	-2.321895000	-1.440326000
6	-2.606526000	-3.493191000	1.310406000
6	-4.004411000	-1.105327000	0.897552000
6	-3.958021000	-3.443388000	1.607782000
1	-2.045202000	-4.422767000	1.465087000
1	-4.562269000	-0.179152000	0.737696000
6	-4.655776000	-2.226728000	1.392957000
1	-4.477475000	-4.323286000	1.998239000
1	-5.726262000	-2.165650000	1.619236000
6	-3.341134000	1.445416000	-1.361156000
6	-2.865061000	2.122819000	0.973421000
6	3.855794000	-2.840396000	0.817673000
6	2.010573000	-2.265363000	2.461237000
6	3.572754000	-2.752103000	-1.584735000
6	1.349970000	-2.119891000	-2.666426000
1	4.488063000	-3.049312000	1.687493000
6	4.379136000	-3.010571000	-0.467834000
1	1.039787000	-1.742018000	2.422627000
6	2.947043000	-1.431044000	3.364196000
6	1.775947000	-3.655575000	3.101177000
1	3.983402000	-2.896549000	-2.588920000
1	0.592775000	-1.354821000	-2.391558000
6	0.578948000	-3.419657000	-2.999143000
6	2.102542000	-1.599535000	-3.906374000
1	5.412097000	-3.348819000	-0.601634000
1	2.496666000	-1.311881000	4.364950000
1	3.125979000	-0.425717000	2.950821000
1	3.927515000	-1.918901000	3.503557000
1	1.068834000	-4.266933000	2.516311000
1	1.369846000	-3.549441000	4.122304000
1	2.722813000	-4.219858000	3.170032000
1	1.281262000	-4.231038000	-3.260717000
1	-0.098048000	-3.263449000	-3.857466000
1	-0.027640000	-3.758585000	-2.143493000
1	2.689440000	-0.693369000	-3.679002000
1	1.384383000	-1.351454000	-4.706548000
1	2.793815000	-2.355581000	-4.318928000
6	-4.051192000	2.648768000	-1.536080000
6	-3.247924000	0.445376000	-2.514946000
6	-3.587571000	3.310296000	0.744593000
6	-2.251645000	1.865415000	2.351185000
1	-4.519335000	2.856119000	-2.505298000
6	-4.178378000	3.578852000	-0.496324000
1	-2.560317000	-0.357789000	-2.195189000
6	-4.620827000	-0.203560000	-2.806922000
6	-2.660036000	1.083132000	-3.793465000
1	-3.690693000	4.036668000	1.559071000
1	-1.669308000	0.930420000	2.280441000
6	-3.344236000	1.659958000	3.426007000
6	-1.278667000	2.989800000	2.770263000
1	-4.737881000	4.507460000	-0.651964000
1	-5.018662000	-0.711069000	-1.912933000
1	-4.533902000	-0.951313000	-3.615057000
1	-5.359363000	0.553494000	-3.126341000
1	-3.311220000	1.882844000	-4.188319000
1	-2.551051000	0.324359000	-4.588237000

1	-1.666869000	1.526382000	-3.605402000
1	-3.965919000	2.565701000	3.543008000
1	-2.887119000	1.437581000	4.406453000
1	-4.009540000	0.821748000	3.162068000
1	-0.473363000	3.121679000	2.027297000
1	-0.811300000	2.753925000	3.742507000
1	-1.796257000	3.959464000	2.877971000
1	1.798315000	1.825437000	1.510454000
1	2.939740000	0.543537000	1.044213000
6	3.511649000	2.520396000	0.394787000
6	3.323687000	3.870265000	0.754558000
6	4.697128000	2.169160000	-0.286038000
6	4.285720000	4.843997000	0.439064000
1	2.416401000	4.159144000	1.299246000
6	5.661194000	3.137629000	-0.602142000
1	4.864079000	1.121224000	-0.563648000
6	5.457892000	4.480952000	-0.242223000
1	4.121046000	5.886772000	0.732033000
1	6.577467000	2.843058000	-1.125710000
1	6.211128000	5.237466000	-0.486338000

IX$_0$' (III)
SCF (BP86/SDD/6-31G**) Energy 333 K = -1787.90624798
Thermal correction to Gibbs Free Energy= 0.677636
Lowest Frequency = 11.1210 cm^{-1}
Second Frequency = 15.0052 cm^{-1}
SCF (B3PW91-D3,C6H6/tzvp) Energy 333 K= -2927.64062702

26	-0.276374000	0.210742000	-0.432934000
1	-0.793477000	1.841019000	-0.990631000
7	-2.149980000	0.002921000	0.036453000
6	-2.705990000	-1.179000000	0.290380000
6	-2.039989000	-2.440355000	0.320282000
6	-0.609789000	-2.619664000	0.147229000
7	0.242629000	-1.561640000	-0.079069000
6	1.660568000	-1.827987000	-0.092326000
6	2.352230000	-1.890597000	-1.339519000
6	3.744851000	-2.096475000	-1.327569000
6	4.449677000	-2.250494000	-0.126091000
6	3.758043000	-2.201514000	1.089783000
6	2.365086000	-1.992394000	1.139376000
6	1.600993000	-1.779874000	-2.668163000
6	2.371419000	-0.994954000	-3.749779000
6	1.664134000	-1.987267000	2.502967000
6	2.233057000	-0.916415000	3.461318000
6	-3.038362000	1.143839000	0.071134000
6	-3.682743000	1.559009000	-1.127822000
6	-4.536070000	2.677520000	-1.068045000
6	-4.744447000	3.370675000	0.131427000
6	-4.089326000	2.954826000	1.296939000
6	-3.223198000	1.843538000	1.294811000
6	-3.491856000	0.801083000	-2.443077000
6	-4.707892000	-0.107583000	-2.739824000
6	-2.533471000	1.401690000	2.586435000
6	-1.831019000	2.570441000	3.310549000
6	-3.530172000	0.690530000	3.531330000
6	-3.206396000	1.738100000	-3.636507000
6	1.201763000	-3.177589000	-3.199535000
6	1.734346000	-3.380213000	3.173989000
1	-4.253308000	3.501974000	2.231792000
1	-5.416031000	4.235375000	0.156814000
1	-5.050616000	3.006854000	-1.977287000
1	4.309239000	-2.330815000	2.028266000
1	5.532496000	-2.414554000	-0.139115000
1	4.286742000	-2.146315000	-2.277879000
1	0.601047000	-1.748144000	2.331754000
1	-1.753607000	0.670779000	2.307890000
1	-1.278770000	2.197033000	4.190191000
1	-1.112553000	3.081114000	2.647318000
1	-2.550584000	3.325937000	3.672132000
1	-3.021166000	0.347581000	4.449172000
1	-4.346777000	1.371791000	3.830094000
1	-3.987790000	-0.188107000	3.046596000
1	1.691621000	-0.942520000	4.423458000
1	3.301222000	-1.091728000	3.680310000
1	2.137248000	0.098023000	3.043666000
1	1.188813000	-3.371538000	4.134108000
1	1.287176000	-4.159409000	2.536887000
1	2.779955000	-3.667121000	3.385825000
1	-2.609965000	0.145333000	-2.318669000

1	-4.552536000	-0.675666000	-3.673807000
1	-4.878594000	-0.830977000	-1.925237000
1	-5.628154000	0.492378000	-2.854685000
1	-2.983213000	1.145781000	-4.540764000
1	-4.072068000	2.381096000	-3.873438000
1	-2.344610000	2.397639000	-3.437743000
1	0.662522000	-1.227872000	-2.458043000
1	0.621986000	-3.087059000	-4.135490000
1	2.100990000	-3.782691000	-3.414571000
1	0.588257000	-3.728589000	-2.468646000
1	1.728868000	-0.839396000	-4.633785000
1	2.699214000	-0.005811000	-3.386389000
1	3.268036000	-1.538692000	-4.096541000
6	0.266851000	2.328878000	-0.938645000
1	0.103231000	3.167999000	-0.243339000
1	0.368860000	2.707513000	-1.969839000
1	-3.789445000	-1.193261000	0.493559000
6	-0.120492000	-3.968661000	0.204711000
1	0.949004000	-4.135859000	0.056743000
6	-0.962348000	-5.052991000	0.415440000
6	-2.864391000	-3.584501000	0.553573000
1	-0.535747000	-6.062138000	0.430814000
1	-3.939260000	-3.413925000	0.690130000
6	-2.355702000	-4.871650000	0.602825000
1	-3.013404000	-5.728110000	0.776700000
6	1.331847000	1.350764000	-0.508639000
1	1.933572000	0.984482000	-1.359739000
6	2.223981000	1.705496000	0.681832000
1	1.598052000	2.041635000	1.529915000
1	2.746865000	0.787169000	1.003947000
6	3.252752000	2.781224000	0.354108000
6	4.426792000	2.452316000	-0.357164000
6	3.044267000	4.128365000	0.712761000
6	5.359532000	3.440732000	-0.704300000
6	3.975366000	5.121553000	0.366852000
6	5.136169000	4.781051000	-0.345096000
1	4.608553000	1.406110000	-0.631883000
1	2.145774000	4.399928000	1.280632000
1	6.267709000	3.163964000	-1.251209000
1	3.795961000	6.161919000	0.659918000
1	5.865419000	5.552898000	-0.613020000

IX (III)
SCF (BP86/SDD/6-31G**) Energy 333 K = -1787.89646572
Thermal correction to Gibbs Free Energy= 0.676823
Lowest Frequency = 10.9956 cm^{-1}
Second Frequency = 14.4349 cm^{-1}
SCF (B3PW91-D3,C6H6/tzvp) Energy 333 K= -2927.62566337

6	-1.548204000	2.437773000	-0.765083000
6	-0.955177000	1.638666000	0.406553000
26	0.008829000	-0.018456000	0.332937000
7	1.832064000	-0.095101000	-0.040339000
1	-0.440713000	1.862987000	2.533395000
7	-0.355877000	-1.866111000	-0.044385000
6	-0.702703000	2.480326000	1.656882000
6	0.548064000	-2.763027000	-0.426584000
6	-1.729147000	-2.288503000	0.072040000
6	1.936802000	-2.503484000	-0.616220000
1	0.202178000	-3.793129000	-0.609843000
6	2.552712000	-1.202078000	-0.428935000
6	2.749354000	-3.610332000	-1.012818000
6	3.971263000	-1.124085000	-0.640144000
6	2.512580000	1.168433000	0.095735000
6	2.775190000	1.964586000	-1.059698000
6	2.904478000	1.613927000	1.395575000
6	3.430955000	3.198317000	-0.885176000
6	2.393215000	1.515822000	-2.472737000
6	3.818552000	3.648197000	0.382625000
1	3.643795000	3.816676000	-1.764676000
6	3.552074000	2.859099000	1.508905000
1	4.328276000	4.611279000	0.493898000
1	3.857565000	3.215468000	2.497822000
6	2.669563000	0.749343000	2.636228000
6	1.497649000	2.550383000	-3.190163000
6	3.645510000	1.216748000	-3.331241000
1	1.814530000	0.580779000	-2.379572000
1	1.200991000	2.173442000	-4.184836000
1	0.581435000	2.758946000	-2.616160000
1	2.026078000	3.507617000	-3.345567000

6	2.373280000	1.564838000	3.911670000
6	3.861525000	-0.206451000	2.886980000
1	1.781884000	0.120096000	2.422608000
1	2.066273000	0.888343000	4.727969000
1	3.265411000	2.110110000	4.267822000
1	1.568027000	2.300902000	3.753000000
6	-2.531306000	-2.404374000	-1.098637000
6	-2.263802000	-2.541372000	1.368337000
6	-3.873573000	-2.802340000	-0.941583000
6	-1.979115000	-2.123925000	-2.497768000
6	-4.413679000	-3.065485000	0.322876000
1	-4.506115000	-2.905637000	-1.830142000
6	-3.612100000	-2.931439000	1.464558000
1	-5.460007000	-3.373854000	0.420181000
1	-4.040972000	-3.138727000	2.450293000
6	-1.384586000	-2.451243000	2.616349000
6	-1.802140000	-3.434089000	-3.300636000
6	-2.853111000	-1.120658000	-3.282403000
1	-0.982010000	-1.665964000	-2.377705000
1	-1.375660000	-3.225307000	-4.297389000
1	-1.130109000	-4.139069000	-2.783036000
1	-2.771272000	-3.943332000	-3.446868000
6	-2.111024000	-1.843599000	3.833437000
6	-0.788150000	-3.835453000	2.965721000
1	-0.536994000	-1.781527000	2.367577000
1	-1.398976000	-1.695487000	4.663474000
1	-2.564149000	-0.867663000	3.590640000
1	-2.912005000	-2.503758000	4.210179000
1	-0.122590000	-3.763823000	3.843888000
1	-1.590136000	-4.557188000	3.202454000
1	-0.202200000	-4.245108000	2.126323000
1	-2.386821000	-0.888386000	-4.255585000
1	-3.860093000	-1.523883000	-3.488475000
1	-2.976769000	-0.174227000	-2.729643000
1	3.347707000	0.870383000	-4.336669000
1	4.264637000	2.122802000	-3.458923000
1	4.272529000	0.434432000	-2.875111000
1	3.661354000	-0.854503000	3.758828000
1	4.058992000	-0.852510000	2.017224000
1	4.779824000	0.370911000	3.097625000
1	0.144841000	3.166053000	1.473571000
1	-1.578445000	3.101153000	1.936741000
1	4.464552000	-0.162206000	-0.481914000
6	4.722445000	-2.229834000	-1.015993000
1	5.803303000	-2.115489000	-1.154179000
6	4.114118000	-3.495118000	-1.216103000
1	2.252788000	-4.578347000	-1.151687000
1	4.710721000	-4.360669000	-1.518095000
1	-1.699119000	0.775215000	0.655897000
1	-0.838097000	3.251330000	-1.006841000
1	-1.611100000	1.789528000	-1.657573000
6	-2.919910000	3.039806000	-0.479971000
6	-4.074839000	2.228814000	-0.518476000
6	-3.070485000	4.398892000	-0.136607000
6	-5.339697000	2.759185000	-0.223797000
1	-3.979543000	1.169135000	-0.785800000
6	-4.335018000	4.934130000	0.160231000
1	-2.185628000	5.046046000	-0.111255000
6	-5.474339000	4.115540000	0.118096000
1	-6.223592000	2.113276000	-0.265625000
1	-4.429346000	5.994149000	0.420430000
1	-6.461450000	4.531483000	0.345868000

IX_E(III)
SCF (BP86/SDD/6-31G**) Energy 333 K = -1787.90652191
Thermal correction to Gibbs Free Energy= 0.679216
Lowest Frequency = 9.0756 cm^{-1}
Second Frequency = 16.2686 cm^{-1}
SCF (B3PW91-D3,C6H6/tzvp) Energy 333 K= -2927.64241020

6	-0.754346000	1.580516000	1.582676000
1	-1.364004000	2.313827000	1.024207000
6	-1.798095000	-1.869379000	-0.828632000
1	-2.777846000	-2.228754000	-1.177072000
6	-0.714289000	-2.784944000	-0.963760000
6	0.667391000	-2.481800000	-0.634992000
6	1.633996000	-3.513146000	-0.890227000
1	2.682863000	-3.304168000	-0.664486000
6	1.272426000	-4.752970000	-1.400726000
1	2.048341000	-5.508701000	-1.567144000

6	-0.079811000	-5.050357000	-1.708525000
1	-0.358810000	-6.027925000	-2.112321000
6	-1.037892000	-4.073648000	-1.492674000
1	-2.089250000	-4.275816000	-1.731209000
6	-3.014709000	0.102762000	-0.337184000
6	-3.142812000	1.253153000	-1.170736000
6	-4.350023000	1.972972000	-1.135179000
1	-4.465827000	2.856299000	-1.770641000
6	-5.411107000	1.574363000	-0.311111000
1	-6.344636000	2.146828000	-0.300305000
6	-5.272979000	0.438650000	0.491960000
1	-6.107460000	0.127329000	1.129718000
6	-4.083973000	-0.320269000	0.505563000
6	-2.030254000	1.652377000	-2.142203000
1	-1.066623000	1.343701000	-1.680296000
6	-1.941588000	3.166706000	-2.409636000
1	-1.048123000	3.387860000	-3.017618000
1	-1.865750000	3.744098000	-1.473196000
1	-2.815089000	3.539573000	-2.973380000
6	-2.163612000	0.866457000	-3.468535000
1	-2.146366000	-0.222103000	-3.296119000
1	-1.336823000	1.117809000	-4.155780000
1	-3.114688000	1.116314000	-3.971601000
6	-4.018223000	-1.568011000	1.395128000
1	-2.971688000	-1.917285000	1.419058000
6	-4.888910000	-2.709135000	0.813797000
1	-5.952489000	-2.413077000	0.784686000
1	-4.807106000	-3.616485000	1.437449000
1	-4.594046000	-2.975836000	-0.214743000
6	-4.444833000	-1.292821000	2.855085000
1	-3.844878000	-0.491200000	3.314350000
1	-4.314793000	-2.204933000	3.463106000
1	-5.507470000	-1.002063000	2.925483000
6	2.427538000	-1.008337000	0.079072000
6	3.021608000	-1.276484000	1.348799000
6	4.378316000	-0.948065000	1.539499000
1	4.844860000	-1.150434000	2.510354000
6	5.143117000	-0.376398000	0.515065000
1	6.196546000	-0.129408000	0.685110000
6	4.553299000	-0.132730000	-0.732714000
1	5.157057000	0.299199000	-1.539209000
6	3.201805000	-0.443510000	-0.979472000
6	2.234638000	-1.938069000	2.482687000
1	1.173552000	-1.950655000	2.176381000
6	2.682718000	-3.404558000	2.690858000
1	2.556480000	-3.996785000	1.770254000
1	2.086859000	-3.882779000	3.488432000
1	3.745852000	-3.455162000	2.987568000
6	2.332137000	-1.156636000	3.811247000
1	3.364676000	-1.138277000	4.202570000
1	1.698398000	-1.630138000	4.581481000
1	2.001786000	-0.110387000	3.695120000
6	2.610969000	-0.205597000	-2.371293000
1	1.523497000	-0.383238000	-2.299141000
6	3.183235000	-1.213896000	-3.395895000
1	4.275188000	-1.087835000	-3.508101000
1	2.722814000	-1.061616000	-4.388176000
1	2.989510000	-2.253376000	-3.085297000
6	2.815737000	1.243031000	-2.866126000
1	2.391731000	1.979365000	-2.162902000
1	2.327663000	1.384132000	-3.846504000
1	3.886036000	1.482877000	-2.997376000
26	-0.202525000	0.198041000	0.288826000
7	-1.761098000	-0.620282000	-0.356565000
7	1.022511000	-1.260030000	-0.121732000
6	0.722474000	1.868155000	1.498757000
1	1.253670000	1.106162000	0.773594000
1	1.238130000	1.612862000	2.445364000
6	-1.355624000	1.236670000	2.936026000
1	-2.401676000	0.909693000	2.818519000
1	-0.800918000	0.422699000	3.436839000
1	-1.361757000	2.102321000	3.631191000
6	1.196728000	3.229408000	0.996553000
6	0.422498000	4.388392000	1.202896000
6	2.457348000	3.361567000	0.375536000
6	0.892313000	5.647186000	0.793211000

6	2.926962000	4.618338000	-0.035011000
6	2.144348000	5.766624000	0.170670000
1	-0.551283000	4.304677000	1.696685000
1	3.076253000	2.470180000	0.215751000
1	0.276234000	6.536722000	0.963584000
1	3.906691000	4.699868000	-0.517761000
1	2.509343000	6.747776000	-0.150615000

TS(IX$_E$-IX$_Z$) (III)
SCF (BP86/SDD/6-31G**) Energy 333 K = -1787.89842523
Thermal correction to Gibbs Free Energy= 0.676677
Lowest Frequency = -109.8014 cm^{-1}
Second Frequency = 10.2340 cm^{-1}
SCF (B3PW91-D3,C6H6/tzvp) Energy 333 K= -2927.63357657

1	-1.532483000	1.289196000	-0.945298000
6	-1.168091000	2.012098000	-0.165071000
6	0.342281000	2.184263000	-0.319042000
26	0.164469000	0.214693000	-0.013420000
1	0.799452000	2.700612000	0.542315000
6	0.826361000	2.752440000	-1.644038000
7	1.915123000	-0.383354000	0.196667000
6	2.227001000	-1.667461000	0.424387000
1	3.298184000	-1.875865000	0.565330000
6	1.358273000	-2.792537000	0.497382000
6	-0.082445000	-2.722222000	0.334390000
6	1.966850000	-4.065118000	0.734953000
6	-0.814241000	-3.954827000	0.405022000
7	-0.688823000	-1.515982000	0.125969000
1	-1.899717000	-3.919503000	0.276976000
6	-0.178803000	-5.167614000	0.633607000
1	-0.777047000	-6.084400000	0.682148000
6	1.229180000	-5.235088000	0.805328000
1	1.721689000	-6.194856000	0.986612000
1	3.056234000	-4.095145000	0.859091000
6	3.038937000	0.528029000	0.122994000
6	3.366866000	1.314425000	1.262182000
6	3.791807000	0.608456000	-1.080866000
6	4.471047000	2.181997000	1.170989000
6	2.576544000	1.184043000	2.564343000
1	4.744367000	2.793868000	2.036677000
6	5.231951000	2.271331000	-0.002524000
1	6.090881000	2.949178000	-0.049879000
6	4.891159000	1.490412000	-1.112684000
1	5.489737000	1.563284000	-2.027111000
6	3.463284000	-0.247010000	-2.307640000
1	1.560948000	0.838297000	2.287137000
6	2.422567000	2.515295000	3.327279000
6	3.199948000	0.105134000	3.482210000
1	1.734791000	2.384039000	4.180313000
1	2.018170000	3.312468000	2.680692000
1	3.383384000	2.870458000	3.740290000
1	3.241909000	-0.876733000	2.982842000
1	2.607683000	-0.009231000	4.407330000
1	4.229711000	0.384349000	3.768278000
1	2.449851000	-0.660795000	-2.161069000
6	4.445694000	-1.436232000	-2.433906000
6	3.444238000	0.566645000	-3.620282000
1	5.481913000	-1.076617000	-2.564255000
1	4.190310000	-2.060653000	-3.308057000
1	4.425310000	-2.080619000	-1.539609000
1	2.750896000	1.420645000	-3.557450000
1	3.122250000	-0.076232000	-4.457946000
1	4.443955000	0.958604000	-3.877498000
6	-2.119584000	-1.471032000	-0.020152000
6	-2.698171000	-1.554774000	-1.322172000
6	-2.942248000	-1.274088000	1.129636000
6	-4.096057000	-1.434007000	-1.445870000
6	-1.844938000	-1.792830000	-2.569670000
1	-4.551861000	-1.501278000	-2.440353000
6	-4.913308000	-1.238614000	-0.324780000
1	-5.998741000	-1.150892000	-0.442447000
6	-4.334175000	-1.162415000	0.948548000
1	-4.976288000	-1.014368000	1.824120000
6	-2.352222000	-1.205761000	2.539704000
1	-0.787341000	-1.732041000	-2.257667000
6	-2.082944000	-3.207046000	-3.148555000
6	-2.076066000	-0.713939000	-3.650877000
1	-1.860075000	-3.986120000	-2.401360000
1	-1.436064000	-3.380652000	-4.026606000

1	-3.131628000	-3.333962000	-3.472206000
1	-3.111228000	-0.733300000	-4.035117000
1	-1.402603000	-0.879953000	-4.509786000
1	-1.884426000	0.300721000	-3.260822000
1	-1.254529000	-1.148403000	2.430917000
6	-2.677276000	-2.486715000	3.343778000
6	-2.814265000	0.050046000	3.311695000
1	-3.767824000	-2.606069000	3.474806000
1	-2.219721000	-2.440819000	4.347842000
1	-2.293673000	-3.385810000	2.834808000
1	-2.590334000	0.978046000	2.757856000
1	-2.306646000	0.105972000	4.290475000
1	-3.901161000	0.035576000	3.506092000
1	-1.376917000	1.519565000	0.826593000
6	-2.109361000	3.217200000	-0.243262000
1	1.925679000	2.690070000	-1.711671000
1	0.402837000	2.204817000	-2.506130000
1	0.548057000	3.818971000	-1.774447000
6	-1.676552000	4.492208000	0.169606000
6	-3.430838000	3.062280000	-0.708250000
6	-2.550332000	5.590005000	0.122685000
1	-0.648738000	4.622937000	0.523978000
6	-4.303143000	4.160331000	-0.755735000
1	-3.777182000	2.073072000	-1.030612000
6	-3.865930000	5.428726000	-0.340115000
1	-2.198468000	6.575954000	0.445145000
1	-5.326621000	4.024121000	-1.121525000
1	-4.545169000	6.286632000	-0.380646000

IX$_{Z}$ (III)
SCF (BP86/SDD/6-31G**) Energy 333 K = -1787.90442746
Thermal correction to Gibbs Free Energy= 0.677632
Lowest Frequency = 8.9758 cm^{-1}
Second Frequency = 14.1201 cm^{-1}
SCF (B3PW91-D3,C6H6/tzvp) Energy 333 K= -2927.64267371

6	0.446505000	2.249421000	-0.322733000
1	1.351739000	2.696923000	0.118713000
6	2.209747000	-1.702143000	-0.270385000
1	3.263409000	-2.010189000	-0.349269000
6	1.242204000	-2.739896000	-0.396979000
6	-0.191309000	-2.552123000	-0.257109000
6	-1.005263000	-3.733647000	-0.341047000
1	-2.085811000	-3.626822000	-0.218770000
6	-0.462272000	-4.990406000	-0.573454000
1	-1.130319000	-5.857098000	-0.631934000
6	0.936186000	-5.165411000	-0.732330000
1	1.358244000	-6.157613000	-0.916461000
6	1.755311000	-4.052957000	-0.638617000
1	2.841666000	-4.162000000	-0.744256000
6	3.171612000	0.414067000	0.144427000
6	3.520957000	0.834151000	1.459646000
6	4.678792000	1.616671000	1.624509000
1	4.967318000	1.941106000	2.629556000
6	5.474280000	1.977216000	0.529551000
1	6.374971000	2.582015000	0.678955000
6	5.111337000	1.561925000	-0.756029000
1	5.734403000	1.850388000	-1.609235000
6	3.958508000	0.782773000	-0.982881000
6	2.707265000	0.394909000	2.678179000
1	1.688082000	0.147012000	2.315413000
6	2.560756000	1.495732000	3.747838000
1	1.866726000	1.164768000	4.539407000
1	2.169551000	2.433016000	3.316753000
1	3.522211000	1.727443000	4.238943000
6	3.298056000	-0.896035000	3.292651000
1	3.333745000	-1.712991000	2.553250000
1	2.689690000	-1.236218000	4.149078000
1	4.327280000	-0.719654000	3.652903000
6	3.608336000	0.357164000	-2.411879000
1	2.558326000	0.014610000	-2.409409000
6	4.493685000	-0.820732000	-2.886630000
1	5.559939000	-0.532554000	-2.882869000
1	4.225799000	-1.116704000	-3.916087000
1	4.385224000	-1.707580000	-2.241523000
6	3.720136000	1.522082000	-3.421855000
1	3.164647000	2.412919000	-3.086133000
1	3.317050000	1.213525000	-4.401862000
1	4.769679000	1.824488000	-3.583794000
6	-2.163792000	-1.236779000	0.089707000

6	-2.972503000	-1.096080000	-1.078771000
6	-4.371194000	-1.020953000	-0.923115000
1	-5.000666000	-0.919594000	-1.814437000
6	-4.971151000	-1.070156000	0.341772000
1	-6.060515000	-1.011938000	0.439097000
6	-4.166162000	-1.193579000	1.481795000
1	-4.635910000	-1.232030000	2.471166000
6	-2.764549000	-1.283805000	1.383679000
6	-2.365745000	-1.028797000	-2.482143000
1	-1.273083000	-0.927453000	-2.357589000
6	-2.633737000	-2.325211000	-3.282340000
1	-2.225302000	-3.209135000	-2.766806000
1	-2.167101000	-2.265885000	-4.281641000
1	-3.717975000	-2.482446000	-3.426759000
6	-2.866627000	0.201140000	-3.272405000
1	-3.942251000	0.121709000	-3.511328000
1	-2.325515000	0.286319000	-4.231039000
1	-2.721066000	1.137115000	-2.707411000
6	-1.925774000	-1.458720000	2.650818000
1	-0.866969000	-1.338192000	2.357384000
6	-2.087699000	-2.881184000	3.236219000
1	-3.133543000	-3.066770000	3.540428000
1	-1.448917000	-3.010729000	4.127918000
1	-1.806361000	-3.650330000	2.498538000
6	-2.241895000	-0.388200000	3.718397000
1	-2.123031000	0.633195000	3.318019000
1	-1.566751000	-0.498745000	4.585090000
1	-3.275684000	-0.477907000	4.095996000
26	0.284048000	0.342106000	0.179788000
7	1.989512000	-0.408290000	-0.024418000
7	-0.730241000	-1.307903000	-0.036228000
6	-0.748597000	2.275481000	0.607516000
1	-1.191602000	1.186574000	0.733374000
1	-0.423013000	2.533656000	1.633358000
6	0.249375000	2.609808000	-1.786375000
1	1.145032000	2.347994000	-2.372822000
1	-0.610034000	2.078154000	-2.230394000
1	0.062105000	3.693138000	-1.933602000
6	-1.973147000	3.102770000	0.234972000
6	-3.222622000	2.523762000	-0.055508000
6	-1.846706000	4.508199000	0.189640000
6	-4.322659000	3.332623000	-0.388838000
6	-2.945001000	5.314470000	-0.140477000
6	-4.188627000	4.728234000	-0.433192000
1	-3.339849000	1.435240000	-0.020352000
1	-0.878133000	4.968504000	0.417615000
1	-5.286958000	2.863593000	-0.611201000
1	-2.830200000	6.403480000	-0.169076000
1	-5.046529000	5.357594000	-0.692192000

IX_E' (III)
SCF (BP86/SDD/6-31G**) Energy 333 K = -1787.90527209
Thermal correction to Gibbs Free Energy= 0.679757
Lowest Frequency = 15.3056 cm^{-1}
Second Frequency = 17.0350 cm^{-1}
SCF (B3PW91-D3,C6H6/tzvp) Energy 333 K= -2927.64272338

6	1.088200000	1.810998000	-1.467173000
1	1.440985000	1.202483000	-0.498625000
1	2.024858000	1.719324000	-2.048965000
6	-0.077089000	1.110417000	-2.118281000
6	0.884687000	3.240288000	-0.997712000
26	0.085540000	0.122611000	-0.426487000
1	-0.985083000	1.733302000	-2.172192000
6	0.207688000	0.342356000	-3.402353000
7	1.384567000	-1.174383000	0.238072000
7	-1.456987000	-0.827425000	0.045827000
6	-0.290514000	3.635506000	-0.322809000
6	1.880636000	4.207460000	-1.238447000
6	1.014986000	-2.394370000	0.632215000
6	2.802203000	-0.897631000	0.296014000
6	-1.517962000	-2.117204000	0.513623000
6	-2.664684000	-0.034086000	0.061980000
6	-0.315043000	-2.899072000	0.742667000
1	1.817154000	-3.086772000	0.936670000
6	3.304843000	-0.107705000	1.369657000
6	3.666725000	-1.390521000	-0.722739000
6	-2.769882000	-2.740944000	0.838718000
6	-2.981223000	0.706528000	1.243210000
6	-3.520340000	0.023612000	-1.078610000

1	1.100085000	-0.299545000	-3.299801000
1	-0.636685000	-0.306689000	-3.684472000
1	0.392431000	1.025528000	-4.257520000
6	-0.443872000	-4.251923000	1.189840000
6	4.684851000	0.165197000	1.408391000
6	2.393186000	0.384544000	2.494551000
6	5.040441000	-1.088521000	-0.630631000
6	3.156274000	-2.237331000	-1.891096000
6	-1.672184000	-4.837265000	1.445742000
1	0.478789000	-4.824990000	1.342969000
1	-3.684143000	-2.153299000	0.724114000
6	-2.844463000	-4.054914000	1.280073000
1	-1.737285000	-5.874815000	1.786040000
1	-3.825927000	-4.488484000	1.502130000
6	-4.142071000	1.503657000	1.248968000
6	-2.130463000	0.616210000	2.513004000
6	-4.670202000	0.834912000	-1.014063000
6	-3.247673000	-0.792233000	-2.343739000
6	-4.984210000	1.573650000	0.132987000
1	-4.394124000	2.071799000	2.151522000
6	-1.722568000	2.002048000	3.058968000
6	-2.857273000	-0.204817000	3.604860000
1	-1.203387000	0.075311000	2.251699000
1	-5.336975000	0.883028000	-1.881864000
6	-3.397034000	0.048809000	-3.631283000
6	-4.164113000	-2.037343000	-2.425741000
1	-2.203480000	-1.145816000	-2.280299000
1	-5.884785000	2.196505000	0.158550000
1	-1.075751000	1.885854000	3.946253000
1	-1.168160000	2.590637000	2.308895000
1	-2.600337000	2.594926000	3.371520000
1	-2.222072000	-0.304506000	4.502744000
1	-3.797388000	0.289311000	3.909451000
1	-3.104651000	-1.217107000	3.246247000
1	-3.077488000	-0.537919000	-4.510423000
1	-4.446995000	0.343160000	-3.805993000
1	-2.792949000	0.970124000	-3.594540000
1	-3.947930000	-2.614728000	-3.342195000
1	-4.020506000	-2.705960000	-1.562439000
1	-5.227838000	-1.740052000	-2.457445000
6	5.552144000	-0.320680000	0.421438000
1	5.087947000	0.761597000	2.233879000
6	2.519374000	-0.529899000	3.735912000
6	2.638753000	1.861359000	2.868135000
1	1.351659000	0.304326000	2.129942000
1	5.720970000	-1.465040000	-1.402203000
6	3.670805000	-1.727771000	-3.256186000
6	3.523402000	-3.729165000	-1.708635000
1	2.054389000	-2.160755000	-1.900272000
1	6.623867000	-0.101321000	0.473030000
1	1.834842000	-0.199153000	4.536760000
1	2.277142000	-1.577348000	3.489850000
1	3.548461000	-0.507657000	4.136852000
1	1.899689000	2.190563000	3.618716000
1	3.639708000	2.016242000	3.308012000
1	2.549122000	2.522245000	1.989690000
1	3.196805000	-2.291982000	-4.078105000
1	3.448112000	-0.657388000	-3.401729000
1	4.762394000	-1.858332000	-3.358265000
1	3.134132000	-4.332424000	-2.547613000
1	4.619336000	-3.862413000	-1.674324000
1	3.108336000	-4.139772000	-0.773338000
6	-0.465939000	4.965039000	0.089441000
1	-1.080101000	2.899534000	-0.126272000
6	1.705918000	5.538676000	-0.826534000
1	2.799827000	3.914308000	-1.759370000
6	0.531706000	5.922289000	-0.160676000
1	-1.386660000	5.252717000	0.607885000
1	2.490452000	6.275882000	-1.028123000
1	0.393983000	6.959405000	0.162233000

TS$_E$(IX$_E$'-IX$_Z$') (III)
SCF (BP86/SDD/6-31G**) Energy 333 K = -1787.89518672
Thermal correction to Gibbs Free Energy= 0.676475
Lowest Frequency = -62.4512 cm^{-1}
Second Frequency = 5.8628 cm^{-1}
SCF (B3PW91-D3,C6H6/tzvp) Energy 333 K= -2927.62713545

1	2.339062000	0.813301000	-0.226578000
6	1.712322000	1.706635000	0.001840000

6	0.510541000	1.763699000	-0.973362000
26	0.046082000	0.074522000	-0.079555000
1	1.342577000	1.584320000	1.052436000
6	2.620722000	2.931123000	-0.015883000
7	0.641456000	-1.750515000	0.109309000
7	-1.791648000	-0.225911000	-0.034915000
6	-0.202918000	-2.778973000	0.173190000
6	2.052895000	-2.045528000	0.160153000
6	-2.390581000	-1.462892000	0.079508000
6	-2.632798000	0.946019000	0.012386000
1	-0.180617000	2.581778000	-0.702488000
6	0.889983000	1.790963000	-2.446227000
6	-1.627298000	-2.697857000	0.158370000
1	0.230734000	-3.788403000	0.262640000
6	2.744831000	-1.889885000	1.395890000
6	2.732635000	-2.445725000	-1.025378000
6	-2.327742000	-3.940794000	0.264699000
6	-3.819606000	-1.582453000	0.168263000
6	-3.708471000	-4.017646000	0.323761000
1	-1.725724000	-4.856511000	0.310736000
1	-4.415891000	-0.667442000	0.144836000
6	-4.453735000	-2.811816000	0.286781000
6	-2.919696000	1.533528000	1.280225000
6	-3.159797000	1.511398000	-1.188082000
6	-3.740212000	2.676980000	1.324189000
6	-2.381595000	0.949452000	2.587535000
6	-3.977001000	2.653658000	-1.083164000
6	-2.870692000	0.905391000	-2.563395000
6	-4.272296000	3.235202000	0.156537000
1	-3.969719000	3.132121000	2.294559000
6	-1.536721000	1.979661000	3.369801000
6	-3.521561000	0.388507000	3.468485000
1	-1.717733000	0.107481000	2.324121000
1	-4.913092000	4.121873000	0.210120000
1	-4.391199000	3.097864000	-1.994113000
6	-2.668813000	1.977030000	-3.657810000
6	-3.981268000	-0.077338000	-3.008957000
1	-1.931366000	0.330506000	-2.465987000
1	-1.117105000	1.522923000	4.283419000
1	-0.698393000	2.356845000	2.758591000
1	-2.139899000	2.850564000	3.681349000
1	-3.113193000	-0.066146000	4.388387000
1	-4.223555000	1.185919000	3.771327000
1	-4.095546000	-0.384063000	2.931111000
1	-2.282398000	1.508539000	-4.579738000
1	-3.619477000	2.471616000	-3.926028000
1	-1.960317000	2.761808000	-3.345254000
1	-3.740847000	-0.504276000	-3.998986000
1	-4.104551000	-0.909873000	-2.299280000
1	-4.950571000	0.446674000	-3.093883000
6	4.125131000	-2.162284000	1.422760000
6	2.001703000	-1.493064000	2.672200000
6	4.113701000	-2.710718000	-0.937520000
6	2.004173000	-2.610684000	-2.360761000
6	4.808039000	-2.573544000	0.270282000
1	4.673862000	-2.055822000	2.364211000
6	1.577450000	-2.748770000	3.470698000
6	2.798887000	-0.521395000	3.566351000
1	1.074361000	-0.974539000	2.358885000
1	5.881718000	-2.784876000	0.314262000
1	4.653713000	-3.028949000	-1.835849000
6	2.749667000	-1.931308000	-3.530299000
6	1.753177000	-4.103805000	-2.678616000
1	1.021178000	-2.116267000	-2.263336000
1	1.011272000	-2.463984000	4.374963000
1	0.940448000	-3.414754000	2.865472000
1	2.463578000	-3.325908000	3.789776000
1	2.170397000	-0.177671000	4.405791000
1	3.690392000	-1.001218000	4.007181000
1	3.137722000	0.365764000	3.005113000
1	2.140859000	-1.981631000	-4.449477000
1	2.957988000	-0.869532000	-3.316363000
1	3.712732000	-2.425234000	-3.749089000
1	1.207689000	-4.212923000	-3.632405000
1	2.707595000	-4.652678000	-2.767939000
1	1.158881000	-4.592652000	-1.888936000

6	2.264715000	4.090883000	0.703324000
6	3.797501000	2.948768000	-0.791054000
1	-5.547005000	-2.842083000	0.352135000
1	-4.212544000	-4.984712000	0.408377000
1	1.551142000	0.945569000	-2.711149000
1	-0.001547000	1.725720000	-3.089424000
1	1.428299000	2.721734000	-2.722472000
6	3.066866000	5.240000000	0.649529000
1	1.351815000	4.089523000	1.310620000
6	4.599274000	4.100339000	-0.847507000
1	4.089717000	2.051526000	-1.348293000
6	4.237318000	5.249648000	-0.127811000
1	2.778260000	6.129880000	1.219258000
1	5.512684000	4.095069000	-1.452043000
1	4.864443000	6.146235000	-0.168476000

IX$_{Z}$′ (III)
SCF (BP86/SDD/6-31G**) Energy 333 K = -1787.90334103
Thermal correction to Gibbs Free Energy= 0.679151
Lowest Frequency = 10.7909 cm^{-1}
Second Frequency = 15.7641 cm^{-1}
SCF (B3PW91-D3,C6H6/tzvp) Energy 333 K= -2927.64187611

1	1.416599000	0.867322000	0.891095000
6	1.196199000	2.025157000	0.746777000
26	-0.092529000	0.322846000	0.139300000
7	0.726356000	-1.407953000	-0.223883000
7	-1.870881000	-0.192506000	-0.166913000
6	0.166827000	2.222239000	-0.344621000
6	0.018373000	-2.447819000	-0.669722000
6	2.141000000	-1.652099000	-0.060592000
6	-1.392602000	-2.511576000	-0.860809000
1	0.574847000	-3.373902000	-0.889022000
6	-2.315265000	-1.428930000	-0.568256000
6	-1.913258000	-3.764974000	-1.313586000
6	-3.716894000	-1.726189000	-0.676984000
6	-2.859677000	0.782495000	0.232853000
6	-3.244289000	0.846323000	1.605560000
6	-3.435361000	1.673153000	-0.722413000
6	-4.204129000	1.799696000	1.996236000
6	-2.666814000	-0.104381000	2.655800000
6	-4.782788000	2.672048000	1.067437000
1	-4.507718000	1.851132000	3.048143000
6	-4.394799000	2.603236000	-0.276355000
1	-5.532740000	3.403043000	1.388157000
1	-4.849045000	3.287771000	-1.000744000
6	-3.057770000	1.633616000	-2.205044000
6	-2.006729000	0.659258000	3.825296000
6	-3.741058000	-1.088124000	3.174319000
1	-1.879657000	-0.701973000	2.161901000
1	-1.549906000	-0.047500000	4.540121000
1	-1.216819000	1.341676000	3.465849000
1	-2.740922000	1.266125000	4.383873000
6	-2.829891000	3.044863000	-2.792215000
6	-4.119379000	0.887394000	-3.049271000
1	-2.110012000	1.070912000	-2.280960000
1	-2.413956000	2.970368000	-3.812306000
1	-3.775065000	3.610651000	-2.871985000
1	-2.133924000	3.640255000	-2.178864000
6	2.647498000	-1.922771000	1.243264000
6	3.002620000	-1.604397000	-1.192915000
6	4.027642000	-2.157598000	1.386134000
6	1.714241000	-2.012111000	2.451331000
6	4.889040000	-2.131850000	0.280783000
1	4.433961000	-2.373014000	2.379804000
6	4.375219000	-1.856066000	-0.991859000
1	5.958885000	-2.325166000	0.412792000
1	5.052606000	-1.829909000	-1.852291000
6	2.484234000	-1.289876000	-2.597932000
6	1.294789000	-3.478679000	2.710531000
6	2.306454000	-1.378354000	3.726928000
1	0.796868000	-1.448132000	2.193280000
1	0.594247000	-3.540464000	3.561895000
1	0.799356000	-3.917601000	1.828729000
1	2.176404000	-4.100461000	2.947783000
6	3.338650000	-0.218492000	-3.311788000
6	2.392271000	-2.567506000	-3.465144000
1	1.463466000	-0.882237000	-2.488012000
1	2.862503000	0.076296000	-4.262894000
1	3.459328000	0.684707000	-2.690601000

1	4.347978000	-0.594243000	-3.556246000
1	2.004221000	-2.328009000	-4.470783000
1	3.387029000	-3.032011000	-3.587076000
1	1.724567000	-3.319940000	-3.013846000
1	1.548564000	-1.362088000	4.528933000
1	3.169685000	-1.949908000	4.111010000
1	2.641456000	-0.342477000	3.549163000
1	-3.302919000	-1.795993000	3.899991000
1	-4.562052000	-0.550958000	3.682186000
1	-4.177369000	-1.671726000	2.347165000
1	-3.821045000	0.868016000	-4.112598000
1	-4.250442000	-0.153077000	-2.713584000
1	-5.098101000	1.396192000	-2.982782000
1	0.780042000	2.337553000	1.722977000
6	2.593202000	2.608236000	0.567840000
1	-4.433357000	-0.944492000	-0.413585000
6	-4.175803000	-2.964845000	-1.103348000
1	-5.255567000	-3.137774000	-1.172788000
6	-3.270410000	-4.002053000	-1.447033000
1	-1.192541000	-4.559490000	-1.542697000
1	-3.638009000	-4.972617000	-1.792540000
1	-0.692894000	2.836801000	-0.033268000
6	0.656727000	2.516028000	-1.754042000
1	1.475087000	1.838100000	-2.051284000
1	-0.156935000	2.403883000	-2.488135000
1	1.049775000	3.548929000	-1.851207000
6	2.755040000	4.006414000	0.675684000
6	3.721538000	1.814221000	0.289008000
6	4.015955000	4.595574000	0.506692000
1	1.882656000	4.633434000	0.894601000
6	4.985034000	2.405487000	0.117446000
1	3.614980000	0.727003000	0.208561000
6	5.137030000	3.795917000	0.224677000
1	4.124076000	5.681918000	0.596376000
1	5.851193000	1.770600000	-0.096989000
1	6.122039000	4.255693000	0.092471000

TS$_E$(IX-X$_E$) (V)
SCF (BP86/SDD/6-31G**) Energy 333 K = -1787.87006968
Thermal correction to Gibbs Free Energy= 0.672983
Lowest Frequency = -775.9161 cm^{-1}
Second Frequency = 13.4881 cm^{-1}
SCF (B3PW91-D3,C6H6/tzvp) Energy 333 K= -2927.61980322

1	0.760991000	1.437736000	0.719414000
6	0.889808000	2.320973000	-0.750218000
6	0.400504000	1.585740000	-1.900215000
1	1.173594000	1.099526000	-2.515092000
26	-0.026241000	0.366635000	-0.256212000
6	-0.795149000	2.114916000	-2.678478000
7	0.864219000	-1.333491000	0.361605000
6	0.279441000	-2.167042000	1.197805000
1	0.874600000	-3.030743000	1.542002000
6	-1.056932000	-2.090179000	1.722798000
6	-2.065756000	-1.122903000	1.321239000
6	-1.395266000	-3.096374000	2.674727000
6	-3.364241000	-1.270430000	1.913419000
7	-1.807112000	-0.141204000	0.398346000
1	-4.145917000	-0.566516000	1.616720000
6	-3.645873000	-2.264431000	2.844492000
1	-4.653529000	-2.322083000	3.271450000
6	-2.657743000	-3.194454000	3.244053000
1	-2.883814000	-3.974597000	3.976108000
1	-0.614170000	-3.813609000	2.954712000
6	2.231823000	-1.585998000	-0.007905000
6	3.274335000	-1.350016000	0.931280000
6	2.504471000	-2.019140000	-1.337872000
6	4.597899000	-1.594444000	0.513401000
6	3.010853000	-0.842108000	2.350692000
1	5.416488000	-1.426333000	1.221634000
6	4.887615000	-2.033462000	-0.783036000
1	5.925095000	-2.215977000	-1.082748000
6	3.846066000	-2.237600000	-1.699771000
1	4.079613000	-2.580955000	-2.712408000
6	1.363827000	-2.310023000	-2.314013000
1	1.943325000	-0.572031000	2.420896000
6	3.817835000	0.437084000	2.666477000
6	3.297790000	-1.941146000	3.400482000
1	3.549023000	0.813584000	3.668929000
1	3.612095000	1.231592000	1.931707000

1	4.905588000	0.246240000	2.666880000
1	2.689548000	-2.845324000	3.226086000
1	3.077344000	-1.572599000	4.417531000
1	4.358934000	-2.246806000	3.375723000
1	0.523077000	-1.638766000	-2.043813000
6	0.863106000	-3.764930000	-2.147193000
6	1.720176000	-2.025966000	-3.786224000
1	1.668469000	-4.482056000	-2.385895000
1	0.014510000	-3.965471000	-2.824431000
1	0.529382000	-3.963612000	-1.115316000
1	2.118155000	-1.006109000	-3.921311000
1	0.821168000	-2.128095000	-4.417332000
1	2.469960000	-2.737273000	-4.175584000
6	-2.899939000	0.641014000	-0.108255000
6	-3.694803000	0.126652000	-1.177292000
6	-3.152782000	1.941631000	0.427353000
6	-4.734156000	0.927193000	-1.686899000
6	-3.462457000	-1.267086000	-1.764988000
1	-5.351772000	0.541479000	-2.506119000
6	-4.993139000	2.202539000	-1.170475000
1	-5.806566000	2.809519000	-1.582251000
6	-4.205784000	2.698274000	-0.123404000
1	-4.414473000	3.695171000	0.278886000
6	-2.341478000	2.499637000	1.599738000
1	-2.557052000	-1.680098000	-1.288301000
6	-4.640962000	-2.215760000	-1.443096000
6	-3.205174000	-1.222101000	-3.287614000
1	-4.801280000	-2.300612000	-0.356034000
1	-4.442940000	-3.227373000	-1.839379000
1	-5.579752000	-1.854368000	-1.899723000
1	-4.076896000	-0.826689000	-3.838273000
1	-3.004684000	-2.237481000	-3.672618000
1	-2.337686000	-0.586112000	-3.530187000
1	-1.377917000	1.955716000	1.621913000
6	-3.052628000	2.237256000	2.949511000
6	-2.025623000	4.003825000	1.455948000
1	-4.034948000	2.742805000	2.977840000
1	-2.446599000	2.625824000	3.787139000

1	-3.217437000	1.161432000	3.120059000
1	-1.586756000	4.241409000	0.471851000
1	-1.310421000	4.315540000	2.236528000
1	-2.927996000	4.629422000	1.576965000
1	0.225720000	3.122908000	-0.396756000
6	2.334276000	2.643085000	-0.526824000
1	-1.253816000	1.335265000	-3.309068000
1	-1.585884000	2.494498000	-2.007962000
1	-0.505885000	2.950841000	-3.349338000
6	3.384596000	1.860558000	-1.056850000
6	2.672077000	3.803021000	0.206760000
6	4.723153000	2.232889000	-0.865091000
1	3.160242000	0.944047000	-1.612173000
6	4.009719000	4.178417000	0.394355000
1	1.869148000	4.419885000	0.627543000
6	5.044078000	3.394176000	-0.142897000
1	5.519104000	1.606214000	-1.280745000
1	4.244154000	5.087447000	0.958887000
1	6.090014000	3.684227000	0.001761000

TS_Z(IX-X_Z) (V)
SCF (BP86/SDD/6-31G**) Energy 333 K = -1787.86552625
Thermal correction to Gibbs Free Energy= 0.671718
Lowest Frequency = -777.7044 cm^{-1}
Second Frequency = 11.2324 cm^{-1}
SCF (B3PW91-D3,C6H6/tzvp) Energy 333 K= -2927.61464424

1	-0.138481000	1.472633000	0.834533000
6	0.507209000	2.417360000	-0.479295000
6	0.572648000	1.679844000	-1.724516000
1	1.591107000	1.376864000	-2.010943000
26	0.144213000	0.246258000	-0.227620000
6	-0.376843000	1.905169000	-2.888550000
7	1.782124000	-0.807509000	0.288783000
6	1.720329000	-1.996761000	0.867599000
1	2.676806000	-2.437862000	1.198415000
6	0.561857000	-2.802825000	1.100892000
6	-0.802236000	-2.441065000	0.735222000
6	0.805440000	-4.066305000	1.723472000
6	-1.821589000	-3.417481000	1.013869000
7	-1.094423000	-1.245515000	0.154903000
1	-2.850908000	-3.176041000	0.736101000

6	-1.532445000	-4.632124000	1.618412000
1	-2.349083000	-5.337247000	1.810901000
6	-0.205981000	-4.974695000	1.988597000
1	0.011605000	-5.933810000	2.466416000
1	1.841332000	-4.308401000	1.991007000
6	3.075702000	-0.192203000	0.132818000
6	3.585058000	0.632888000	1.174879000
6	3.786688000	-0.384989000	-1.085008000
6	4.844189000	1.233836000	0.980890000
6	2.822004000	0.830741000	2.486621000
1	5.262791000	1.863795000	1.772721000
6	5.568817000	1.042128000	-0.202123000
1	6.545975000	1.519740000	-0.330387000
6	5.039786000	0.241868000	-1.222957000
1	5.610196000	0.100516000	-2.146761000
6	3.232703000	-1.283151000	-2.192681000
1	1.764348000	0.569815000	2.297081000
6	2.844984000	2.290493000	2.986751000
6	3.356809000	-0.122366000	3.582273000
1	2.197388000	2.393620000	3.874088000
1	2.477032000	2.990112000	2.217999000
1	3.858544000	2.613042000	3.283680000
1	3.278139000	-1.179526000	3.277974000
1	2.787611000	0.004427000	4.519770000
1	4.420176000	0.085211000	3.797683000
1	2.138855000	-1.344357000	-2.042664000
6	3.804614000	-2.716464000	-2.076198000
6	3.474827000	-0.721269000	-3.609205000
1	4.903192000	-2.709281000	-2.190704000
1	3.383701000	-3.369359000	-2.860890000
1	3.572555000	-3.169264000	-1.097874000
1	3.101950000	0.312043000	-3.707556000
1	2.954517000	-1.345121000	-4.356113000
1	4.545905000	-0.721321000	-3.877887000
6	-2.457219000	-0.893317000	-0.129806000
6	-2.954348000	-1.024900000	-1.464845000
6	-3.279143000	-0.351080000	0.906229000
6	-4.274077000	-0.613528000	-1.730216000

6	-2.089286000	-1.632358000	-2.572433000
1	-4.670401000	-0.706855000	-2.746081000
6	-5.092580000	-0.088962000	-0.720663000
1	-6.117632000	0.221150000	-0.950610000
6	-4.593344000	0.037661000	0.580805000
1	-5.235368000	0.449181000	1.367949000
6	-2.787900000	-0.200284000	2.348210000
1	-1.038982000	-1.362052000	-2.343621000
6	-2.178537000	-3.178279000	-2.572851000
6	-2.419606000	-1.095080000	-3.979863000
1	-1.879302000	-3.609989000	-1.605119000
1	-1.523852000	-3.602378000	-3.354953000
1	-3.213264000	-3.502923000	-2.784727000
1	-3.398980000	-1.459477000	-4.338297000
1	-1.664281000	-1.448116000	-4.703028000
1	-2.434544000	0.006498000	-4.009818000
1	-1.722977000	-0.487147000	2.367338000
6	-3.555224000	-1.141729000	3.306249000
6	-2.877791000	1.261368000	2.840138000
1	-4.631783000	-0.894908000	3.332809000
1	-3.164683000	-1.043835000	4.334500000
1	-3.454245000	-2.196806000	3.004137000
1	-2.297083000	1.938227000	2.192321000
1	-2.478026000	1.341745000	3.866437000
1	-3.922160000	1.620874000	2.863496000
1	1.477527000	2.597811000	0.003725000
6	-0.467978000	3.535049000	-0.259277000
1	-0.269792000	1.102352000	-3.636295000
1	-1.435081000	1.932890000	-2.582001000
1	-0.171183000	2.865837000	-3.404010000
6	-1.868309000	3.354846000	-0.269210000
6	0.038250000	4.840878000	-0.079800000
6	-2.733709000	4.449130000	-0.123456000
1	-2.281116000	2.343941000	-0.363029000
6	-0.826894000	5.935505000	0.072472000
1	1.123226000	4.998180000	-0.066920000
6	-2.217339000	5.744584000	0.046517000
1	-3.816501000	4.285157000	-0.129768000

1	-0.411389000	6.939142000	0.213875000
1	-2.894029000	6.596931000	0.167650000

TS_E(IX_E-X_E) (III)
SCF (BP86/SDD/6-31G**) Energy 333 K = -1787.89782946
Thermal correction to Gibbs Free Energy= 0.677342
Lowest Frequency = -340.9273 cm^{-1}
Second Frequency = 9.9872 cm^{-1}
SCF (B3PW91-D3,C6H6/tzvp) Energy 333 K= -2927.63379438

1	-0.731983000	1.415236000	-0.803974000
6	0.459583000	1.689503000	-1.912228000
6	1.161841000	0.467685000	-2.199189000
1	2.222110000	0.409023000	-1.914490000
26	0.088445000	0.153129000	-0.510644000
6	0.761008000	-0.390483000	-3.386032000
7	1.163292000	-1.222134000	0.334141000
6	0.676292000	-2.298671000	0.941882000
1	1.405775000	-2.989118000	1.395670000
6	-0.698071000	-2.636712000	1.116001000
6	-1.803173000	-1.765981000	0.759053000
6	-0.962627000	-3.883733000	1.762019000
6	-3.116416000	-2.220687000	1.129390000
7	-1.608288000	-0.563856000	0.120062000
1	-3.969363000	-1.577743000	0.901641000
6	-3.328374000	-3.444823000	1.749645000
1	-4.353059000	-3.744874000	1.996426000
6	-2.246008000	-4.302249000	2.070914000
1	-2.419746000	-5.263758000	2.562504000
1	-0.102732000	-4.514551000	2.018515000
6	2.599345000	-1.046522000	0.423335000
6	3.102281000	-0.101605000	1.364705000
6	3.474276000	-1.799843000	-0.407159000
6	4.495852000	0.076209000	1.447332000
6	2.166507000	0.641772000	2.320699000
1	4.900714000	0.795156000	2.166320000
6	5.373163000	-0.654039000	0.634942000
1	6.454587000	-0.501530000	0.717111000
6	4.861544000	-1.583096000	-0.276884000
1	5.551300000	-2.157779000	-0.904395000
6	2.966863000	-2.846683000	-1.401730000
1	1.186639000	0.737840000	1.809850000
6	2.640501000	2.063689000	2.678207000
6	1.936239000	-0.190318000	3.605808000
1	1.860735000	2.579830000	3.263635000
1	2.843395000	2.664728000	1.777017000
1	3.553282000	2.050416000	3.300406000
1	1.518011000	-1.184873000	3.379984000
1	1.235166000	0.327825000	4.283227000
1	2.888291000	-0.337030000	4.146577000
1	1.876994000	-2.703902000	-1.508173000
6	3.203621000	-4.281305000	-0.871822000
6	3.602154000	-2.690006000	-2.801431000
1	4.281664000	-4.473303000	-0.728020000
1	2.817997000	-5.029087000	-1.586854000
1	2.703987000	-4.448236000	0.096799000
1	3.478848000	-1.667433000	-3.193848000
1	3.132127000	-3.389909000	-3.514065000
1	4.682528000	-2.916853000	-2.787979000
6	-2.784105000	0.259800000	-0.057439000
6	-3.554100000	0.157274000	-1.252665000
6	-3.155595000	1.181159000	0.964779000
6	-4.683658000	0.987006000	-1.398421000
6	-3.197118000	-0.831777000	-2.363811000
1	-5.285312000	0.912060000	-2.311349000
6	-5.056720000	1.898267000	-0.403141000
1	-5.940539000	2.531319000	-0.536490000
6	-4.289881000	1.990477000	0.765079000
1	-4.580362000	2.702100000	1.546791000
6	-2.359662000	1.311192000	2.264205000
1	-2.186407000	-1.217887000	-2.139994000
6	-4.167715000	-2.036334000	-2.388522000
6	-3.150345000	-0.157316000	-3.752991000
1	-4.166046000	-2.573848000	-1.426667000
1	-3.880566000	-2.751448000	-3.180013000
1	-5.201668000	-1.704469000	-2.592973000
1	-4.149570000	0.184350000	-4.076190000
1	-2.788686000	-0.869551000	-4.515626000
1	-2.483560000	0.721961000	-3.757431000
1	-1.486906000	0.640119000	2.183812000

6	-3.190704000	0.853434000	3.484778000
6	-1.824251000	2.746421000	2.465882000
1	-4.083209000	1.489771000	3.623155000
1	-2.589863000	0.914051000	4.409557000
1	-3.532069000	-0.188144000	3.365989000
1	-1.192914000	3.062487000	1.618198000
1	-1.217778000	2.806599000	3.387088000
1	-2.647004000	3.476810000	2.564524000
1	-0.363028000	1.941782000	-2.599694000
6	1.111177000	2.916605000	-1.332922000
1	0.963394000	-1.461040000	-3.216003000
1	-0.312550000	-0.283281000	-3.613000000
1	1.320220000	-0.095473000	-4.296718000
6	2.507184000	3.036160000	-1.164634000
6	0.301129000	4.027050000	-0.999345000
6	3.072628000	4.227194000	-0.680060000
1	3.162700000	2.200669000	-1.428100000
6	0.864307000	5.216096000	-0.517508000
1	-0.785464000	3.949351000	-1.125388000
6	2.256072000	5.321584000	-0.354161000
1	4.159681000	4.300202000	-0.567380000
1	0.215511000	6.063300000	-0.270957000
1	2.699858000	6.250354000	0.018985000

TS$_E$(IX$_E$'-X$_E$') (III)
SCF (BP86/SDD/6-31G**) Energy 333 K = -1787.89729376
Thermal correction to Gibbs Free Energy= 0.677449
Lowest Frequency = -332.2834 cm^{-1}
Second Frequency = 15.4028 cm^{-1}
SCF (B3PW91-D3,C6H6/tzvp) Energy 333 K= -2927.63234948

6	0.274737000	1.941166000	-1.705726000
1	1.142846000	1.423510000	-0.370307000
1	1.251376000	2.125710000	-2.178057000
6	-0.485250000	0.845703000	-2.248433000
6	-0.342375000	3.200550000	-1.174560000
26	0.191979000	0.218197000	-0.453324000
1	-1.579946000	0.922201000	-2.189181000
6	0.047210000	0.054347000	-3.429843000
7	1.702649000	-0.806439000	0.219916000
7	-1.141223000	-1.045350000	0.159170000
6	-1.697327000	3.293811000	-0.784362000
6	0.458551000	4.361669000	-1.088685000
6	1.600339000	-2.042176000	0.705032000
6	3.049456000	-0.269509000	0.196471000
6	-0.930977000	-2.264723000	0.749057000
6	-2.496725000	-0.546678000	0.135354000
6	0.409071000	-2.784779000	0.949406000
1	2.542193000	-2.541138000	0.986438000
6	3.466916000	0.585890000	1.253047000
6	3.925261000	-0.606210000	-0.872193000
6	-2.013776000	-3.079424000	1.229148000
6	-2.949404000	0.263604000	1.222445000
6	-3.367707000	-0.841499000	-0.957839000
1	1.149709000	0.034783000	-3.433862000
1	-0.309485000	-0.989399000	-3.430216000
1	-0.278048000	0.507351000	-4.388012000
6	0.578803000	-4.089177000	1.509826000
6	4.781026000	1.088562000	1.221805000
6	2.545983000	0.932249000	2.423457000
6	5.231303000	-0.076345000	-0.850541000
6	3.497728000	-1.526689000	-2.017359000
6	-0.493132000	-4.868185000	1.911252000
1	1.603849000	-4.459186000	1.633931000
1	-3.031024000	-2.689714000	1.144558000
6	-1.801719000	-4.335558000	1.780834000
1	-0.334735000	-5.862604000	2.338261000
1	-2.664093000	-4.920756000	2.119423000
6	-4.260376000	0.777199000	1.181201000
6	-2.073966000	0.541228000	2.447497000
6	-4.667706000	-0.297691000	-0.946464000
6	-2.951799000	-1.760896000	-2.109943000
6	-5.117042000	0.507159000	0.107400000
1	-4.615896000	1.395992000	2.012467000
6	-2.039724000	2.034488000	2.837519000
6	-2.523240000	-0.321584000	3.651408000
1	-1.042618000	0.241139000	2.187349000
1	-5.344211000	-0.518510000	-1.779297000
6	-3.310507000	-1.183347000	-3.497890000
6	-3.578544000	-3.169604000	-1.964804000

1	-1.854641000	-1.873653000	-2.053612000
1	-6.133089000	0.916051000	0.094584000
1	-1.330144000	2.191552000	3.668586000
1	-1.727859000	2.669911000	1.992700000
1	-3.026348000	2.391167000	3.183459000
1	-1.865303000	-0.145390000	4.520774000
1	-3.555494000	-0.066642000	3.952303000
1	-2.493565000	-1.396831000	3.411833000
1	-2.886906000	-1.818986000	-4.294865000
1	-4.403102000	-1.154298000	-3.655469000
1	-2.926282000	-0.159743000	-3.637530000
1	-3.261392000	-3.819203000	-2.799902000
1	-3.277122000	-3.655256000	-1.023841000
1	-4.681676000	-3.110204000	-1.984025000
6	5.662730000	0.761938000	0.183670000
1	5.121264000	1.740772000	2.033777000
6	2.978927000	0.182761000	3.704927000
6	2.457508000	2.453315000	2.671201000
1	1.531517000	0.584154000	2.159416000
1	5.922260000	-0.329115000	-1.662060000
6	3.879221000	-0.960465000	-3.403178000
6	4.081004000	-2.949377000	-1.845731000
1	2.396482000	-1.607123000	-1.981921000
1	6.683376000	1.159039000	0.181521000
1	2.291288000	0.406838000	4.539323000
1	2.983984000	-0.909446000	3.550074000
1	3.996225000	0.481587000	4.014790000
1	1.741380000	2.666884000	3.483651000
1	3.431024000	2.879619000	2.971193000
1	2.116627000	2.985978000	1.767741000
1	3.448882000	-1.586787000	-4.203835000
1	3.511798000	0.071032000	-3.536867000
1	4.972842000	-0.945805000	-3.554452000
1	3.753492000	-3.608148000	-2.669308000
1	5.185211000	-2.923253000	-1.849957000
1	3.761749000	-3.409446000	-0.895961000
6	-2.231114000	4.513176000	-0.340978000
1	-2.343228000	2.410292000	-0.818613000
6	-0.074804000	5.579757000	-0.643451000
1	1.512479000	4.305311000	-1.386630000
6	-1.425416000	5.661839000	-0.268861000
1	-3.285116000	4.562019000	-0.047867000
1	0.565898000	6.466701000	-0.593288000
1	-1.846746000	6.611438000	0.076902000

TS$_Z$(IX$_Z$-X$_Z$) (III)

SCF (BP86/SDD/6-31G**) Energy 333 K = -1787.89567177
Thermal correction to Gibbs Free Energy= 0.676395
Lowest Frequency = -467.8521 cm^{-1}
Second Frequency = 11.3925 cm^{-1}
SCF (B3PW91-D3,C6H6/tzvp) Energy 333 K= -2927.63088596

1	-0.743577000	1.160925000	0.986126000
6	-0.125597000	2.469695000	0.222743000
6	0.713735000	2.109965000	-0.901983000
1	1.793618000	2.243238000	-0.754523000
26	0.177781000	0.384262000	0.036734000
6	0.247634000	2.221485000	-2.340970000
7	1.912615000	-0.501396000	0.074599000
6	2.067884000	-1.821427000	0.167016000
1	3.102058000	-2.191592000	0.256729000
6	1.050564000	-2.818139000	0.191738000
6	-0.371431000	-2.536745000	0.140637000
6	1.494178000	-4.173411000	0.294657000
6	-1.255367000	-3.668781000	0.201865000
7	-0.841855000	-1.252086000	0.044756000
1	-2.332180000	-3.486975000	0.170324000
6	-0.779412000	-4.969114000	0.298664000
1	-1.496543000	-5.796516000	0.341671000
6	0.612510000	-5.239575000	0.346553000
1	0.979552000	-6.266850000	0.425972000
1	2.575596000	-4.352571000	0.333317000
6	3.147198000	0.255684000	0.128335000
6	3.561661000	0.800237000	1.375739000
6	3.915442000	0.440807000	-1.054492000
6	4.774641000	1.514992000	1.416483000
6	2.755306000	0.575836000	2.656350000
1	5.116895000	1.931791000	2.369612000
6	5.552482000	1.696247000	0.266390000
1	6.493852000	2.253229000	0.320959000

6	5.120098000	1.164619000	-0.954490000
1	5.729751000	1.312877000	-1.852028000
6	3.476731000	-0.138964000	-2.401178000
1	1.733133000	0.282896000	2.350509000
6	2.629954000	1.847592000	3.521094000
6	3.350784000	-0.588956000	3.482282000
1	1.950495000	1.662585000	4.370788000
1	2.228285000	2.695700000	2.940765000
1	3.600959000	2.160811000	3.943711000
1	3.378554000	-1.524486000	2.899149000
1	2.748959000	-0.771358000	4.389927000
1	4.383838000	-0.358456000	3.798733000
1	2.399791000	-0.373334000	-2.320655000
6	4.223075000	-1.458155000	-2.713606000
6	3.654264000	0.858188000	-3.567072000
1	5.312596000	-1.286080000	-2.771293000
1	3.892037000	-1.872548000	-3.682100000
1	4.044588000	-2.222421000	-1.939610000
1	3.171100000	1.825779000	-3.353854000
1	3.208159000	0.446618000	-4.489061000
1	4.719326000	1.053535000	-3.783225000
6	-2.274341000	-1.078791000	0.012600000
6	-2.948026000	-1.037672000	-1.245832000
6	-3.001156000	-0.934040000	1.231334000
6	-4.346274000	-0.858595000	-1.251846000
6	-2.199064000	-1.204432000	-2.570375000
1	-4.875937000	-0.830613000	-2.210214000
6	-5.070395000	-0.718203000	-0.061575000
1	-6.157005000	-0.583218000	-0.089810000
6	-4.395058000	-0.751920000	1.164944000
1	-4.962175000	-0.639701000	2.095962000
6	-2.313089000	-0.978478000	2.596322000
1	-1.129418000	-1.021466000	-2.360381000
6	-2.332117000	-2.646313000	-3.116844000
6	-2.650606000	-0.188698000	-3.642523000
1	-1.953847000	-3.389838000	-2.397298000
1	-1.763379000	-2.758485000	-4.057090000
1	-3.389532000	-2.885336000	-3.331120000
1	-3.677960000	-0.391494000	-3.993760000
1	-1.990430000	-0.248409000	-4.525107000
1	-2.625395000	0.846733000	-3.263785000
1	-1.226385000	-1.036884000	2.414184000
6	-2.726761000	-2.234207000	3.398141000
6	-2.578580000	0.307035000	3.411242000
1	-3.810741000	-2.234151000	3.611761000
1	-2.194819000	-2.267636000	4.365452000
1	-2.488879000	-3.158280000	2.846021000
1	-2.270007000	1.206002000	2.851246000
1	-2.017838000	0.282184000	4.362298000
1	-3.648645000	0.418186000	3.660847000
1	0.440115000	2.800850000	1.107384000
6	-1.422262000	3.223013000	0.071607000
1	0.741338000	1.475180000	-2.986469000
1	-0.841258000	2.090235000	-2.438485000
1	0.490254000	3.221133000	-2.754816000
6	-2.617968000	2.636930000	-0.391385000
6	-1.424925000	4.601445000	0.382280000
6	-3.777796000	3.411216000	-0.557914000
1	-2.647971000	1.562348000	-0.601031000
6	-2.583953000	5.373884000	0.219784000
1	-0.504755000	5.070568000	0.750466000
6	-3.765860000	4.781424000	-0.255702000
1	-4.695755000	2.931475000	-0.913151000
1	-2.564100000	6.440517000	0.468455000
1	-4.672548000	5.382804000	-0.380179000

TS$_Z$(IX$_Z$'-X$_Z$') (III)
SCF (BP86/SDD/6-31G**) Energy 333 K = -1787.89509468
Thermal correction to Gibbs Free Energy= 0.676342
Lowest Frequency = -397.2559 cm^{-1}
Second Frequency = 11.5485 cm^{-1}
SCF (B3PW91-D3,C6H6/tzvp) Energy 333 K= -2927.62924426

1	1.123877000	0.889618000	0.962949000
6	0.737354000	2.313153000	0.225328000
26	0.026220000	0.342427000	0.035213000
7	0.820542000	-1.419987000	-0.026621000
7	-1.803230000	-0.293034000	-0.015253000
6	-0.063625000	2.109391000	-0.964673000
6	0.128431000	-2.554318000	-0.097377000

6	2.259807000	-1.578199000	0.008933000
6	-1.288517000	-2.703205000	-0.096096000
1	0.711948000	-3.488384000	-0.139777000
6	-2.227236000	-1.599411000	-0.018241000
6	-1.782244000	-4.044440000	-0.131342000
6	-3.618326000	-1.947782000	0.085499000
6	-2.820544000	0.721187000	0.125197000
6	-3.145950000	1.199837000	1.429817000
6	-3.478485000	1.251312000	-1.026373000
6	-4.135633000	2.194433000	1.555822000
6	-2.486414000	0.634576000	2.689605000
6	-4.794481000	2.714422000	0.435901000
1	-4.398152000	2.561435000	2.554452000
6	-4.460750000	2.243657000	-0.840050000
1	-5.564304000	3.484237000	0.555736000
1	-4.976179000	2.653762000	-1.715313000
6	-3.160782000	0.760707000	-2.441728000
6	-1.904035000	1.740067000	3.596743000
6	-3.471181000	-0.257782000	3.481624000
1	-1.644614000	-0.000802000	2.360460000
1	-1.378580000	1.291327000	4.457714000
1	-1.183631000	2.372927000	3.051034000
1	-2.691855000	2.400330000	4.000363000
6	-2.987194000	1.921703000	-3.446744000
6	-4.240718000	-0.218648000	-2.962311000
1	-2.204573000	0.209947000	-2.385419000
1	-2.629306000	1.534212000	-4.416674000
1	-3.942794000	2.440431000	-3.640169000
1	-2.264396000	2.673531000	-3.090299000
6	2.920062000	-1.653139000	1.265874000
6	2.980339000	-1.653581000	-1.216266000
6	4.317868000	-1.819411000	1.270338000
6	2.151966000	-1.588877000	2.586217000
6	5.045829000	-1.906900000	0.077110000
1	4.843440000	-1.886895000	2.229088000
6	4.378293000	-1.821469000	-1.150930000
1	6.132409000	-2.041465000	0.103955000
1	4.951983000	-1.887668000	-2.081250000
6	2.274558000	-1.581573000	-2.572274000
6	2.060420000	-2.986947000	3.240745000
6	2.753816000	-0.556643000	3.563842000
1	1.124707000	-1.258116000	2.353192000
1	1.479064000	-2.940576000	4.178390000
1	1.570742000	-3.714669000	2.571721000
1	3.064619000	-3.378031000	3.483343000
6	3.037371000	-0.718456000	-3.600265000
6	2.021335000	-2.995650000	-3.147653000
1	1.290934000	-1.106263000	-2.401876000
1	2.424578000	-0.579590000	-4.507426000
1	3.282326000	0.277649000	-3.195515000
1	3.981920000	-1.194168000	-3.918169000
1	1.499835000	-2.933050000	-4.119024000
1	2.975506000	-3.528825000	-3.307031000
1	1.403361000	-3.607711000	-2.470419000
1	2.127624000	-0.479569000	4.469690000
1	3.769486000	-0.840723000	3.891086000
1	2.815155000	0.442289000	3.100725000
1	-2.974612000	-0.693759000	4.366574000
1	-4.338713000	0.327553000	3.836241000
1	-3.850743000	-1.085306000	2.860246000
1	-3.984463000	-0.571248000	-3.977203000
1	-4.340110000	-1.100155000	-2.309874000
1	-5.224988000	0.280615000	-3.017338000
1	0.170239000	2.734110000	1.070864000
6	2.153791000	2.828204000	0.186513000
1	-4.347847000	-1.140700000	0.184478000
6	-4.052247000	-3.266439000	0.064471000
1	-5.125006000	-3.474596000	0.145545000
6	-3.133051000	-4.339401000	-0.060112000
1	-1.046226000	-4.854362000	-0.204150000
1	-3.482916000	-5.375370000	-0.085646000
1	-1.107585000	2.444090000	-0.897706000
6	0.527135000	2.112609000	-2.360613000
1	1.575614000	1.776325000	-2.368327000
1	-0.046835000	1.464791000	-3.044669000
1	0.511589000	3.134215000	-2.791526000

6	2.373434000	4.180811000	0.530428000
6	3.260527000	2.047668000	-0.206233000
6	3.658254000	4.739765000	0.470726000
1	1.524040000	4.799451000	0.843584000
6	4.546653000	2.608232000	-0.270329000
1	3.116660000	0.987873000	-0.442471000
6	4.751029000	3.955327000	0.065677000
1	3.805897000	5.790040000	0.744594000
1	5.391547000	1.980806000	-0.573132000
1	5.755263000	4.389740000	0.021165000

X_E(V)
SCF (BP86/SDD/6-31G**) Energy 333 K = -1787.88753286
Thermal correction to Gibbs Free Energy= 0.675513
Lowest Frequency = 14.4958 cm^{-1}
Second Frequency = 19.0257 cm^{-1}
SCF (B3PW91-D3,C6H6/tzvp) Energy 333 K= -2927.63698358

1	0.795623000	1.298531000	1.325830000
6	0.634675000	2.048898000	-1.428063000
6	0.656861000	0.789678000	-2.057374000
1	1.625542000	0.290775000	-2.194839000
26	0.121083000	0.557048000	0.039165000
6	-0.453930000	0.345535000	-2.982589000
7	1.006087000	-1.271566000	0.393601000
6	0.418939000	-2.085485000	1.249964000
1	1.016717000	-2.937616000	1.618810000
6	-0.910924000	-2.004584000	1.787543000
6	-1.947344000	-1.071784000	1.367631000
6	-1.210330000	-2.971596000	2.794624000
6	-3.220851000	-1.197437000	2.021154000
7	-1.728158000	-0.125178000	0.401533000
1	-4.014582000	-0.504959000	1.729505000
6	-3.457059000	-2.141196000	3.012649000
1	-4.442560000	-2.174288000	3.490895000
6	-2.448800000	-3.051483000	3.412932000
1	-2.641522000	-3.796808000	4.189416000
1	-0.412561000	-3.666933000	3.082395000
6	2.382136000	-1.550523000	0.058277000
6	3.422706000	-1.142726000	0.940752000
6	2.665805000	-2.207859000	-1.172878000
6	4.752795000	-1.388454000	0.542616000
6	3.149024000	-0.506741000	2.305748000
1	5.568088000	-1.085323000	1.208334000
6	5.052964000	-2.010709000	-0.674143000
1	6.095445000	-2.186285000	-0.960442000
6	4.013161000	-2.422758000	-1.518113000
1	4.251023000	-2.931177000	-2.458098000
6	1.539230000	-2.758168000	-2.047949000
1	2.085835000	-0.212997000	2.335491000
6	3.965560000	0.782754000	2.534862000
6	3.407140000	-1.525129000	3.441864000
1	3.681435000	1.239454000	3.498724000
1	3.774616000	1.520143000	1.738975000
1	5.052082000	0.585848000	2.572748000
1	2.787540000	-2.431538000	3.329672000
1	3.176986000	-1.075049000	4.423413000
1	4.464108000	-1.846553000	3.456156000
1	0.632493000	-2.171595000	-1.814603000
6	1.242141000	-4.231889000	-1.677288000
6	1.812388000	-2.632101000	-3.560529000
1	2.128084000	-4.865760000	-1.858684000
1	0.408327000	-4.627349000	-2.283809000
1	0.968261000	-4.335670000	-0.614077000
1	2.066380000	-1.597945000	-3.847808000
1	0.920109000	-2.936686000	-4.134066000
1	2.641475000	-3.284932000	-3.885583000
6	-2.854482000	0.580016000	-0.143401000
6	-3.700737000	-0.056481000	-1.108153000
6	-3.098370000	1.934890000	0.248154000
6	-4.784825000	0.672226000	-1.634604000
6	-3.481922000	-1.498883000	-1.578143000
1	-5.442240000	0.191213000	-2.367108000
6	-5.036863000	1.993837000	-1.250244000
1	-5.885525000	2.540233000	-1.675467000
6	-4.192830000	2.614118000	-0.321548000
1	-4.391681000	3.648543000	-0.024025000
6	-2.239799000	2.631434000	1.306860000
1	-2.475667000	-1.805236000	-1.240916000
6	-4.513896000	-2.467896000	-0.951052000

6	-3.532176000	-1.636247000	-3.117748000
1	-4.470875000	-2.456825000	0.148699000
1	-4.326257000	-3.502055000	-1.290403000
1	-5.539423000	-2.194235000	-1.258070000
1	-4.550771000	-1.463717000	-3.507743000
1	-3.242851000	-2.659352000	-3.415372000
1	-2.857072000	-0.927324000	-3.622164000
1	-1.223704000	2.183688000	1.274165000
6	-2.787010000	2.375485000	2.731642000
6	-2.076378000	4.145872000	1.066937000
1	-3.807019000	2.788133000	2.836222000
1	-2.142057000	2.862448000	3.484006000
1	-2.827909000	1.299861000	2.965424000
1	-1.770187000	4.367589000	0.029967000
1	-1.307546000	4.550978000	1.746332000
1	-3.010164000	4.702142000	1.265639000
1	-0.294270000	2.634460000	-1.518985000
6	1.817542000	2.837405000	-1.009825000
1	-0.599700000	-0.746615000	-2.968067000
1	-1.414197000	0.821527000	-2.721963000
1	-0.215638000	0.626488000	-4.028121000
6	3.140796000	2.336808000	-1.073079000
6	1.633602000	4.175582000	-0.586730000
6	4.229941000	3.145249000	-0.722238000
1	3.323092000	1.305489000	-1.391286000
6	2.723186000	4.983010000	-0.237370000
1	0.616875000	4.580920000	-0.535725000
6	4.029927000	4.471957000	-0.302578000
1	5.244001000	2.735615000	-0.780259000
1	2.551768000	6.016104000	0.083645000
1	4.884436000	5.100868000	-0.032406000

X_Z(V)
SCF (BP86/SDD/6-31G**) Energy 333 K = -1787.88250536
Thermal correction to Gibbs Free Energy= 0.674830
Lowest Frequency = 9.1239 cm^{-1}
Second Frequency = 17.2037 cm^{-1}
SCF (B3PW91-D3,C6H6/tzvp) Energy 333 K= -2927.63398853

1	-0.303790000	1.400801000	-1.643150000
6	0.063521000	2.470034000	0.841313000
6	-0.405958000	1.506664000	1.759493000
1	-1.494985000	1.487926000	1.894105000
26	-0.161155000	0.596277000	-0.226960000
6	0.391422000	0.839094000	2.859174000
7	-1.990012000	-0.368137000	-0.303264000
6	-2.065935000	-1.505135000	-0.965885000
1	-3.077880000	-1.882313000	-1.198263000
6	-0.990791000	-2.336614000	-1.430037000
6	0.423098000	-2.154196000	-1.128690000
6	-1.400427000	-3.464690000	-2.204271000
6	1.314121000	-3.161202000	-1.637066000
7	0.886399000	-1.095645000	-0.393179000
1	2.379585000	-3.056871000	-1.418477000
6	0.868491000	-4.234439000	-2.397710000
1	1.599814000	-4.960824000	-2.769956000
6	-0.505603000	-4.402017000	-2.696557000
1	-0.851587000	-5.250129000	-3.293624000
1	-2.472276000	-3.576002000	-2.408255000
6	-3.230170000	0.267976000	0.067885000
6	-3.883719000	1.138007000	-0.848758000
6	-3.754361000	0.030240000	1.371391000
6	-5.074782000	1.762968000	-0.426600000
6	-3.369956000	1.371839000	-2.270693000
1	-5.598117000	2.429441000	-1.120926000
6	-5.603238000	1.547015000	0.850672000
1	-6.529537000	2.045786000	1.154927000
6	-4.945964000	0.683934000	1.737135000
1	-5.369013000	0.509643000	2.731681000
6	-3.085399000	-0.963269000	2.323462000
1	-2.356529000	0.940701000	-2.340313000
6	-3.230452000	2.872422000	-2.604495000
6	-4.273529000	0.659898000	-3.304692000
1	-2.804490000	2.996766000	-3.615022000
1	-2.557072000	3.375106000	-1.890765000
1	-4.202538000	3.396430000	-2.586888000
1	-4.336132000	-0.424681000	-3.111033000
1	-3.878544000	0.801003000	-4.325959000
1	-5.302673000	1.061008000	-3.281155000
1	-2.022563000	-1.032217000	2.026542000

6	-3.704235000	-2.373448000	2.164660000
6	-3.135264000	-0.524071000	3.801600000
1	-4.779533000	-2.357137000	2.416026000
1	-3.207078000	-3.096128000	2.835430000
1	-3.607262000	-2.747622000	1.132010000
1	-2.742145000	0.497196000	3.940161000
1	-2.533606000	-1.211120000	4.421410000
1	-4.163009000	-0.546240000	4.204692000
6	2.277577000	-1.073942000	-0.022196000
6	2.697237000	-1.713813000	1.188438000
6	3.222618000	-0.391109000	-0.848975000
6	4.058115000	-1.644963000	1.544534000
6	1.725400000	-2.506925000	2.069598000
1	4.393485000	-2.130658000	2.466922000
6	4.991710000	-0.976149000	0.743005000
1	6.045511000	-0.939099000	1.039826000
6	4.570572000	-0.360601000	-0.440701000
1	5.303582000	0.154692000	-1.070742000
6	2.830361000	0.251443000	-2.182524000
1	0.708565000	-2.136540000	1.844305000
6	1.762495000	-4.018818000	1.733414000
6	1.982447000	-2.325310000	3.582456000
1	1.510357000	-4.213600000	0.679881000
1	1.044980000	-4.571622000	2.365696000
1	2.769497000	-4.431003000	1.925631000
1	2.917264000	-2.820887000	3.898956000
1	1.164619000	-2.788745000	4.161122000
1	2.050262000	-1.265074000	3.874726000
1	1.727362000	0.318949000	-2.212799000
6	3.285831000	-0.622664000	-3.376312000
6	3.376037000	1.686765000	-2.336381000
1	4.386056000	-0.727447000	-3.391070000
1	2.978680000	-0.157497000	-4.329601000
1	2.847229000	-1.632686000	-3.335788000
1	3.074060000	2.329320000	-1.494965000
1	2.986295000	2.135790000	-3.266409000
1	4.478813000	1.703972000	-2.402563000
1	-0.731008000	3.006311000	0.304008000
6	1.371113000	3.153992000	0.719574000
1	-0.205816000	0.052020000	3.345699000
1	1.318707000	0.366463000	2.495717000
1	0.680830000	1.568376000	3.642504000
6	2.550561000	2.804636000	1.424890000
6	1.430134000	4.284936000	-0.136342000
6	3.723357000	3.560363000	1.283608000
1	2.562073000	1.936950000	2.085678000
6	2.599830000	5.040552000	-0.272403000
1	0.532738000	4.565812000	-0.699082000
6	3.756835000	4.682644000	0.440798000
1	4.619313000	3.265857000	1.840190000
1	2.609413000	5.910447000	-0.937737000
1	4.674838000	5.270377000	0.337895000

X_E (III)
SCF (BP86/SDD/6-31G**) Energy 333 K = -1787.90260678
Thermal correction to Gibbs Free Energy= 0.678882
Lowest Frequency = 12.6691 cm^{-1}
Second Frequency = 15.5608 cm^{-1}
SCF (B3PW91-D3,C6H6/tzvp) Energy 333 K= -2927.63343264

1	-0.227112000	1.469213000	0.296903000
6	0.577388000	1.742409000	-1.771767000
6	1.123164000	0.495624000	-2.165241000
1	2.183016000	0.303420000	-1.948425000
26	0.003548000	0.194255000	-0.451188000
6	0.539736000	-0.308084000	-3.311448000
7	1.105976000	-1.248649000	0.316251000
6	0.578668000	-2.313466000	0.912612000
1	1.282047000	-3.052152000	1.331046000
6	-0.806873000	-2.593422000	1.123045000
6	-1.892270000	-1.693505000	0.782458000
6	-1.105825000	-3.823854000	1.785089000
6	-3.213014000	-2.090315000	1.179749000
7	-1.668650000	-0.516164000	0.107285000
1	-4.043834000	-1.417499000	0.954744000
6	-3.457204000	-3.299796000	1.817486000
1	-4.486302000	-3.561934000	2.086962000
6	-2.398114000	-4.189949000	2.125060000
1	-2.595539000	-5.139652000	2.630468000
1	-0.266331000	-4.485351000	2.030733000

6	2.551154000	-1.158513000	0.367063000
6	3.140707000	-0.322884000	1.360247000
6	3.350572000	-1.890111000	-0.554996000
6	4.544694000	-0.224061000	1.391978000
6	2.290195000	0.382109000	2.419127000
1	5.016085000	0.410585000	2.148935000
6	5.348204000	-0.923777000	0.482490000
1	6.438518000	-0.829732000	0.525581000
6	4.751141000	-1.751492000	-0.474309000
1	5.382816000	-2.309237000	-1.174040000
6	2.746588000	-2.847806000	-1.584773000
1	1.287913000	0.538971000	1.978240000
6	2.832306000	1.765694000	2.828778000
6	2.125982000	-0.523208000	3.664535000
1	2.112760000	2.262386000	3.501964000
1	2.983993000	2.417058000	1.952870000
1	3.788041000	1.692714000	3.378470000
1	1.664087000	-1.491514000	3.409471000
1	1.487636000	-0.031515000	4.419537000
1	3.106770000	-0.729192000	4.129725000
1	1.663693000	-2.638972000	-1.638385000
6	2.916883000	-4.319755000	-1.137596000
6	3.334830000	-2.653665000	-2.999710000
1	3.986201000	-4.580182000	-1.046449000
1	2.461158000	-5.006000000	-1.872939000
1	2.444421000	-4.507147000	-0.159048000
1	3.255663000	-1.606786000	-3.335509000
1	2.798106000	-3.288188000	-3.726198000
1	4.400030000	-2.940432000	-3.042799000
6	-2.792436000	0.376024000	-0.078278000
6	-3.541631000	0.318462000	-1.291319000
6	-3.138595000	1.306610000	0.946761000
6	-4.624497000	1.205575000	-1.453636000
6	-3.234740000	-0.701038000	-2.390339000
1	-5.211013000	1.167142000	-2.377842000
6	-4.968249000	2.129013000	-0.459987000
1	-5.813689000	2.809483000	-0.607908000
6	-4.225590000	2.172976000	0.726159000
1	-4.497928000	2.892569000	1.506549000
6	-2.386047000	1.382622000	2.276432000
1	-2.218809000	-1.089882000	-2.197798000
6	-4.212352000	-1.899924000	-2.336123000
6	-3.248065000	-0.073837000	-3.801866000
1	-4.170624000	-2.412664000	-1.361875000
1	-3.965728000	-2.637063000	-3.120909000
1	-5.251946000	-1.564421000	-2.501947000
1	-4.266174000	0.224886000	-4.107947000
1	-2.888385000	-0.803293000	-4.548420000
1	-2.609230000	0.823973000	-3.860156000
1	-1.539753000	0.677655000	2.221688000
6	-3.286223000	0.949866000	3.457213000
6	-1.797836000	2.790397000	2.519637000
1	-4.153629000	1.624618000	3.569916000
1	-2.718083000	0.974554000	4.403861000
1	-3.668636000	-0.074430000	3.315680000
1	-1.119596000	3.085758000	1.701503000
1	-1.225186000	2.808859000	3.463590000
1	-2.590730000	3.555223000	2.598551000
1	-0.393488000	2.016744000	-2.214075000
6	1.334985000	2.904954000	-1.238018000
1	0.558989000	-1.396101000	-3.125297000
1	-0.501736000	-0.016029000	-3.519411000
1	1.121446000	-0.132391000	-4.238686000
6	2.718169000	2.859775000	-0.946052000
6	0.661012000	4.137367000	-1.063271000
6	3.395080000	4.002816000	-0.495412000
1	3.273830000	1.924901000	-1.070330000
6	1.337236000	5.278593000	-0.612706000
1	-0.410476000	4.192113000	-1.289412000
6	2.711060000	5.218262000	-0.325160000
1	4.467864000	3.943057000	-0.281608000
1	0.790298000	6.219755000	-0.490093000
1	3.243885000	6.109064000	0.023193000

X_Z(III)
SCF (BP86/SDD/6-31G**) Energy 333 K = -1787.89805303
Thermal correction to Gibbs Free Energy= 0.677761
Lowest Frequency = 11.5053 cm^{-1}
Second Frequency = 21.1534 cm^{-1}

SCF (B3PW91-D3,C6H6/tzvp) Energy 333 K= -2927.63142460

1	-0.582259000	0.999001000	1.196413000
6	0.079527000	2.510786000	-0.133518000
6	0.766314000	1.934440000	-1.239384000
1	1.862198000	1.934739000	-1.171314000
26	0.156876000	0.400408000	0.031325000
6	0.219428000	1.842511000	-2.648257000
7	1.902808000	-0.523348000	0.133216000
6	2.021465000	-1.835666000	0.313141000
1	3.042015000	-2.231934000	0.442220000
6	0.970785000	-2.797504000	0.392545000
6	-0.441808000	-2.483571000	0.299132000
6	1.373170000	-4.152384000	0.604073000
6	-1.357826000	-3.580893000	0.439503000
7	-0.883354000	-1.200590000	0.076997000
1	-2.428244000	-3.371813000	0.378061000
6	-0.921294000	-4.883578000	0.641353000
1	-1.662709000	-5.684432000	0.738229000
6	0.460849000	-5.187247000	0.726555000
1	0.798327000	-6.214844000	0.889201000
1	2.448348000	-4.358050000	0.671000000
6	3.150795000	0.209747000	0.152179000
6	3.545835000	0.857759000	1.356468000
6	3.948809000	0.277242000	-1.024380000
6	4.768989000	1.555807000	1.360544000
6	2.712229000	0.751222000	2.634916000
1	5.097257000	2.050869000	2.280422000
6	5.574458000	1.624211000	0.216968000
1	6.522367000	2.172017000	0.243894000
6	5.162077000	0.990779000	-0.961191000
1	5.794102000	1.048757000	-1.853763000
6	3.535066000	-0.415919000	-2.325069000
1	1.685325000	0.472387000	2.332569000
6	2.615468000	2.082481000	3.408290000
6	3.258031000	-0.371448000	3.549289000
1	1.914415000	1.977058000	4.253896000
1	2.251137000	2.900755000	2.764395000
1	3.588326000	2.392383000	3.829437000

1	3.264497000	-1.346149000	3.033412000
1	2.636895000	-0.471187000	4.456752000
1	4.293139000	-0.151683000	3.866966000
1	2.463062000	-0.667997000	-2.237475000
6	4.313800000	-1.738005000	-2.525128000
6	3.700964000	0.492215000	-3.563287000
1	5.399490000	-1.546502000	-2.591791000
1	3.998770000	-2.238676000	-3.457507000
1	4.148735000	-2.439576000	-1.690783000
1	3.175971000	1.453307000	-3.437047000
1	3.290832000	-0.007547000	-4.457940000
1	4.762267000	0.712849000	-3.772812000
6	-2.312328000	-0.993687000	0.012784000
6	-2.957857000	-1.015976000	-1.261790000
6	-3.061665000	-0.766405000	1.204944000
6	-4.349795000	-0.801649000	-1.310788000
6	-2.190074000	-1.302453000	-2.555262000
1	-4.858369000	-0.820271000	-2.280403000
6	-5.094622000	-0.568098000	-0.148290000
1	-6.175873000	-0.404292000	-0.210076000
6	-4.448263000	-0.550211000	1.093199000
1	-5.032610000	-0.371010000	2.002714000
6	-2.418432000	-0.766079000	2.592693000
1	-1.124981000	-1.088911000	-2.351581000
6	-2.301642000	-2.793410000	-2.957737000
6	-2.637598000	-0.409532000	-3.732876000
1	-1.926842000	-3.460105000	-2.165075000
1	-1.719682000	-2.990835000	-3.875619000
1	-3.354050000	-3.062403000	-3.160543000
1	-3.652506000	-0.669391000	-4.082535000
1	-1.957711000	-0.545878000	-4.591602000
1	-2.639493000	0.659700000	-3.463914000
1	-1.331026000	-0.887851000	2.455200000
6	-2.923347000	-1.950371000	3.449649000
6	-2.644562000	0.576386000	3.323622000
1	-4.011646000	-1.883361000	3.627290000
1	-2.423546000	-1.952550000	4.434431000
1	-2.717102000	-2.917077000	2.961392000

1	-2.264546000	1.422203000	2.726782000
1	-2.122096000	0.575984000	4.296555000
1	-3.715974000	0.756099000	3.522468000
1	0.732592000	2.859299000	0.680295000
6	-1.225440000	3.236130000	-0.174637000
1	0.477726000	0.884379000	-3.132947000
1	-0.873637000	1.960149000	-2.680856000
1	0.656763000	2.645099000	-3.274970000
6	-2.426764000	2.681434000	-0.670341000
6	-1.255958000	4.569698000	0.296933000
6	-3.605033000	3.441674000	-0.719266000
1	-2.451498000	1.631680000	-0.980515000
6	-2.433803000	5.329168000	0.251252000
1	-0.336956000	5.015587000	0.695551000
6	-3.614512000	4.769683000	-0.263813000
1	-4.523423000	2.981682000	-1.098791000
1	-2.428637000	6.360259000	0.621297000
1	-4.536708000	5.359332000	-0.297015000

X_{E}' (III)
SCF (BP86/SDD/6-31G**) Energy 333 K = -1787.90029402
Thermal correction to Gibbs Free Energy= 0.678891
Lowest Frequency = 13.2242 cm^{-1}
Second Frequency = 18.6236 cm^{-1}
SCF (B3PW91-D3,C6H6/tzvp) Energy 333 K= -2927.63232105

6	0.033297000	1.932097000	-1.675211000
1	0.676852000	1.465591000	0.346176000
1	1.072990000	2.166289000	-1.952035000
6	-0.528898000	0.766547000	-2.251381000
6	-0.722878000	3.106211000	-1.164604000
26	0.251580000	0.239406000	-0.408737000
1	-1.619909000	0.652464000	-2.207615000
6	0.159101000	0.016352000	-3.374255000
7	1.799705000	-0.700893000	0.214525000
7	-1.048452000	-1.076671000	0.208692000
6	-2.131504000	3.124551000	-1.037462000
6	-0.011132000	4.285052000	-0.837445000
6	1.759492000	-1.879050000	0.833658000
6	3.102325000	-0.069763000	0.122553000
6	-0.763350000	-2.245159000	0.872783000
6	-2.436388000	-0.690874000	0.119659000
6	0.604757000	-2.658213000	1.135884000
1	2.723734000	-2.290994000	1.171970000
6	3.528747000	0.806026000	1.159259000
6	3.928674000	-0.347711000	-1.002732000
6	-1.800564000	-3.102269000	1.377757000
6	-2.989781000	0.121740000	1.155866000
6	-3.237995000	-1.096476000	-0.991628000
1	1.242045000	0.219840000	-3.388653000
1	0.015745000	-1.076281000	-3.308046000
1	-0.249072000	0.333708000	-4.355100000
6	0.844059000	-3.899804000	1.804488000
6	4.794020000	1.411299000	1.033170000
6	2.690564000	1.069041000	2.411348000
6	5.186113000	0.283482000	-1.072172000
6	3.509586000	-1.343157000	-2.086100000
6	-0.183834000	-4.718522000	2.239600000
1	1.887340000	-4.189398000	1.979071000
1	-2.838523000	-2.791842000	1.237213000
6	-1.521358000	-4.294790000	2.030757000
1	0.029953000	-5.663231000	2.747719000
1	-2.351559000	-4.912995000	2.390044000
6	-4.332136000	0.532768000	1.042966000
6	-2.191444000	0.513790000	2.401686000
6	-4.573091000	-0.650094000	-1.052815000
6	-2.709761000	-2.034265000	-2.082148000
6	-5.121831000	0.160471000	-0.051050000
1	-4.764675000	1.155246000	1.834129000
6	-2.197496000	2.036733000	2.653829000
6	-2.704399000	-0.248638000	3.646735000
1	-1.143383000	0.214577000	2.228685000
1	-5.197695000	-0.953616000	-1.899947000
6	-3.082468000	-1.573074000	-3.509333000
6	-3.206939000	-3.485458000	-1.868471000
1	-1.608480000	-2.041879000	-1.998253000
1	-6.163316000	0.492592000	-0.119865000
1	-1.564202000	2.278141000	3.525570000
1	-1.813609000	2.590488000	1.782299000
1	-3.212825000	2.412336000	2.873834000

1	-2.097680000	0.008072000	4.533186000
1	-3.753677000	0.015978000	3.870309000
1	-2.652251000	-1.340268000	3.502059000
1	-2.575164000	-2.206965000	-4.257199000
1	-4.167372000	-1.661902000	-3.694977000
1	-2.796718000	-0.525616000	-3.699794000
1	-2.811164000	-4.148078000	-2.658622000
1	-2.885854000	-3.888074000	-0.895309000
1	-4.310034000	-3.529706000	-1.911883000
6	5.618329000	1.160036000	-0.069818000
1	5.141151000	2.085797000	1.823627000
6	3.333632000	0.403651000	3.650791000
6	2.455680000	2.576237000	2.649841000
1	1.700827000	0.607521000	2.257039000
1	5.838861000	0.080434000	-1.927363000
6	3.887059000	-0.881278000	-3.510010000
6	4.105230000	-2.745325000	-1.810821000
1	2.409235000	-1.432503000	-2.040637000
1	6.599585000	1.640569000	-0.145112000
1	2.705810000	0.562507000	4.545019000
1	3.455614000	-0.683573000	3.508122000
1	4.332261000	0.826939000	3.859560000
1	1.818647000	2.726748000	3.538633000
1	3.401655000	3.118230000	2.825533000
1	1.949875000	3.039829000	1.786584000
1	3.435886000	-1.553806000	-4.259675000
1	3.539608000	0.145324000	-3.715146000
1	4.978896000	-0.902405000	-3.673450000
1	3.787615000	-3.462882000	-2.587837000
1	5.209051000	-2.707354000	-1.812070000
1	3.784087000	-3.140704000	-0.833230000
6	-2.796642000	4.279442000	-0.601288000
1	-2.716443000	2.230004000	-1.273656000
6	-0.676471000	5.438466000	-0.401714000
1	1.081090000	4.289602000	-0.935525000
6	-2.076252000	5.442379000	-0.281484000
1	-3.888082000	4.267912000	-0.511057000
1	-0.100637000	6.338385000	-0.159749000
1	-2.600257000	6.342625000	0.055959000

X_Z' (III)
SCF (BP86/SDD/6-31G**) Energy 333 K = -1787.89709435
Thermal correction to Gibbs Free Energy= 0.678032
Lowest Frequency = 11.6279 cm^{-1}
Second Frequency = 21.9643 cm^{-1}
SCF (B3PW91-D3,C6H6/tzvp) Energy 333 K= -2927.62948773

1	0.922360000	0.857235000	1.128756000
6	0.527308000	2.385717000	-0.258578000
26	0.056635000	0.343149000	0.007268000
7	0.876867000	-1.385380000	0.115250000
7	-1.780975000	-0.317886000	0.089090000
6	-0.123828000	1.860355000	-1.410815000
6	0.210016000	-2.520057000	0.319025000
6	2.321404000	-1.501742000	0.090065000
6	-1.200976000	-2.690105000	0.424493000
1	0.816689000	-3.432709000	0.432363000
6	-2.169851000	-1.612736000	0.332150000
6	-1.657416000	-4.023313000	0.666747000
6	-3.546807000	-1.966342000	0.542650000
6	-2.819308000	0.681095000	0.046568000
6	-3.135650000	1.405270000	1.235438000
6	-3.502075000	0.959446000	-1.178019000
6	-4.138831000	2.392551000	1.173617000
6	-2.462225000	1.102233000	2.576223000
6	-4.817923000	2.671402000	-0.017951000
1	-4.395501000	2.947695000	2.082780000
6	-4.495640000	1.957681000	-1.178962000
1	-5.596154000	3.441662000	-0.042715000
1	-5.029139000	2.177299000	-2.110158000
6	-3.202304000	0.191850000	-2.469709000
6	-1.893604000	2.367918000	3.253182000
6	-3.433540000	0.365126000	3.528776000
1	-1.611085000	0.429348000	2.370919000
1	-1.368661000	2.097469000	4.185899000
1	-1.173432000	2.886819000	2.598600000
1	-2.688065000	3.087329000	3.519817000
6	-3.046912000	1.121524000	-3.694474000
6	-4.287445000	-0.872810000	-2.762662000
1	-2.243446000	-0.335844000	-2.318590000

1	-2.715663000	0.539574000	-4.572182000
1	-4.003632000	1.600782000	-3.967382000
1	-2.310360000	1.922258000	-3.517608000
6	3.057772000	-1.391329000	1.301168000
6	2.967929000	-1.731348000	-1.158100000
6	4.459825000	-1.505303000	1.233011000
6	2.381982000	-1.187092000	2.657450000
6	5.117080000	-1.725273000	0.016974000
1	5.044631000	-1.425017000	2.155900000
6	4.372609000	-1.839185000	-1.163935000
1	6.208381000	-1.813681000	-0.010911000
1	4.890542000	-2.019083000	-2.111460000
6	2.174168000	-1.910242000	-2.454207000
6	2.491466000	-2.461331000	3.526857000
6	2.941880000	0.044552000	3.401910000
1	1.311643000	-0.995539000	2.472721000
1	1.972696000	-2.317883000	4.490913000
1	2.041550000	-3.334384000	3.024112000
1	3.545901000	-2.708120000	3.744419000
6	2.855232000	-1.263128000	-3.678531000
6	1.899399000	-3.408158000	-2.732792000
1	1.198042000	-1.411903000	-2.309188000
1	2.177597000	-1.295300000	-4.548884000
1	3.121835000	-0.209933000	-3.491202000
1	3.776654000	-1.798336000	-3.968453000
1	1.313449000	-3.529895000	-3.660871000
1	2.847374000	-3.962160000	-2.852799000
1	1.335125000	-3.880281000	-1.912082000
1	2.390202000	0.203381000	4.344896000
1	4.008197000	-0.080696000	3.660369000
1	2.845507000	0.954908000	2.787550000
1	-2.928451000	0.112849000	4.477966000
1	-4.308238000	0.995821000	3.769791000
1	-3.803054000	-0.571253000	3.079553000
1	-4.044938000	-1.426049000	-3.687341000
1	-4.375094000	-1.602000000	-1.942172000
1	-5.273640000	-0.395093000	-2.904387000
1	-0.142765000	2.877251000	0.462983000
6	1.929329000	2.897442000	-0.189740000
1	-4.294278000	-1.170077000	0.513247000
6	-3.943443000	-3.273713000	0.789440000
1	-5.006559000	-3.487384000	0.946461000
6	-2.996467000	-4.328220000	0.842467000
1	-0.902287000	-4.816794000	0.720720000
1	-3.316406000	-5.357436000	1.028737000
1	-1.206570000	2.034479000	-1.461034000
6	0.542731000	1.613832000	-2.746739000
1	1.631703000	1.487304000	-2.657004000
1	0.130134000	0.724090000	-3.253012000
1	0.366689000	2.475768000	-3.421368000
6	2.127080000	4.234129000	0.228217000
6	3.068747000	2.135225000	-0.533626000
6	3.410690000	4.796796000	0.278562000
1	1.256910000	4.839414000	0.508584000
6	4.352959000	2.698460000	-0.486841000
1	2.953442000	1.080273000	-0.803497000
6	4.530947000	4.032094000	-0.084944000
1	3.534894000	5.835126000	0.604882000
1	5.218933000	2.082217000	-0.750631000
1	5.534724000	4.467875000	-0.043546000

XI_Z (V)
SCF (BP86/SDD/6-31G**) Energy 333 K = -1787.89155974
Thermal correction to Gibbs Free Energy= 0.674392
Lowest Frequency = 14.1073 cm^{-1}
Second Frequency = 20.6287 cm^{-1}
SCF (B3PW91-D3,C6H6/tzvp) Energy 333 K= -2927.64718241

1	0.128742000	1.535265000	-1.572815000
6	0.610650000	1.787792000	1.554539000
1	1.027416000	1.022181000	2.219581000
6	-0.781801000	1.746113000	1.401371000
6	1.532453000	2.944946000	1.225603000
26	0.149568000	0.573650000	-0.249501000
1	-1.292941000	0.932514000	1.932916000
6	-1.712475000	2.770152000	0.882821000
7	1.925648000	-0.478527000	-0.281349000
7	-0.902781000	-1.141327000	-0.301127000
6	-1.329593000	3.900824000	0.115874000
6	-3.087400000	2.618874000	1.197974000

6	2.001404000	-1.706056000	-0.764182000
6	3.179792000	0.190681000	-0.028585000
1	3.013058000	-2.105285000	-0.954560000
6	0.926980000	-2.602298000	-1.070419000
6	-0.479535000	-2.333019000	-0.820571000
6	1.313229000	-3.861158000	-1.621923000
6	-1.398988000	-3.391958000	-1.138215000
6	0.394887000	-4.851862000	-1.932698000
1	2.382483000	-4.032265000	-1.796682000
1	-2.462484000	-3.221626000	-0.954135000
6	-0.976241000	-4.599476000	-1.678064000
6	-2.294863000	-1.006291000	0.034107000
1	-1.722809000	-5.367391000	-1.910607000
1	0.719743000	-5.805339000	-2.358243000
6	3.777546000	0.066504000	1.258189000
6	3.777463000	0.974317000	-1.054458000
6	4.981599000	0.755440000	1.498981000
6	3.177241000	-0.839345000	2.336365000
6	4.982836000	1.640725000	-0.755371000
6	3.192603000	1.054688000	-2.466244000
1	5.461964000	0.667889000	2.479151000
6	5.580148000	1.542932000	0.506382000
1	2.115219000	-1.001843000	2.075996000
6	3.227321000	-0.222273000	3.750621000
6	3.868894000	-2.224222000	2.334309000
1	6.516512000	2.071361000	0.714769000
1	5.463136000	2.243575000	-1.533611000
1	2.164053000	0.656249000	-2.427337000
6	4.014208000	0.180747000	-3.443586000
6	3.082479000	2.502996000	-2.986203000
1	2.682589000	-0.864202000	4.464054000
1	2.772713000	0.782607000	3.778373000
1	4.262391000	-0.130910000	4.123637000
1	3.775838000	-2.723676000	1.355780000
1	3.422248000	-2.885365000	3.097639000
1	4.945574000	-2.122231000	2.557799000
1	5.055838000	0.540816000	-3.520875000
1	3.570159000	0.207373000	-4.454077000
1	4.050215000	-0.872093000	-3.115349000
1	2.458833000	3.117180000	-2.316029000
1	2.610010000	2.509611000	-3.983561000
1	4.069148000	2.989438000	-3.084848000
6	-3.214280000	-0.486637000	-0.925312000
6	-2.736572000	-1.370932000	1.344004000
6	-4.566585000	-0.353609000	-0.552131000
6	-2.787628000	-0.112409000	-2.346346000
6	-4.099479000	-1.214418000	1.661589000
6	-1.779170000	-1.970062000	2.378346000
1	-5.282076000	0.037724000	-1.283923000
6	-5.013430000	-0.712503000	0.724700000
1	-1.688581000	-0.202650000	-2.397867000
6	-3.396039000	-1.082747000	-3.386176000
6	-3.138054000	1.351234000	-2.691386000
1	-6.070622000	-0.604226000	0.990315000
1	-4.452701000	-1.500858000	2.658520000
1	-0.752150000	-1.782051000	2.016168000
6	-1.964817000	-3.503244000	2.484066000
6	-1.915962000	-1.324920000	3.775484000
1	-3.103974000	-2.126784000	-3.185240000
1	-3.049962000	-0.822300000	-4.401926000
1	-4.499865000	-1.031371000	-3.385168000
1	-4.228782000	1.526002000	-2.675963000
1	-2.776422000	1.596099000	-3.705486000
1	-2.666901000	2.051584000	-1.983428000
1	-2.983134000	-3.749730000	2.834788000
1	-1.245900000	-3.933542000	3.203779000
1	-1.811299000	-3.995318000	1.510370000
1	-1.792752000	-0.228791000	3.742779000
1	-1.150113000	-1.731883000	4.458477000
1	-2.900052000	-1.534985000	4.230085000
6	-2.280516000	4.848046000	-0.287127000
1	-0.290106000	4.028219000	-0.188468000
6	-4.033696000	3.569034000	0.796391000
1	-3.408756000	1.736184000	1.761877000
6	-3.634097000	4.693683000	0.055607000
1	-1.960356000	5.711708000	-0.879704000

1	-5.087478000	3.427302000	1.058243000
1	-4.371867000	5.438475000	-0.260716000
1	1.157730000	3.886532000	1.669499000
1	2.543778000	2.750400000	1.612831000
1	1.630794000	3.112780000	0.137585000

XI$_z$ (III)
SCF (BP86/SDD/6-31G**) Energy 333 K = -1787.89758545
Thermal correction to Gibbs Free Energy= 0.678567
Lowest Frequency = 10.8930 cm^{-1}
Second Frequency = 20.5189 cm^{-1}
SCF (B3PW91-D3,C6H6/tzvp) Energy 333 K= -2927.63195939

1	-0.927756000	-0.675200000	-1.872436000
6	0.238080000	0.830685000	-2.597638000
1	0.314299000	0.056493000	-3.373389000
6	1.409641000	0.967523000	-1.789552000
6	-0.731601000	1.941438000	-2.964369000
26	-0.114510000	-0.085617000	-0.770631000
1	2.202617000	0.237526000	-2.002371000
6	1.926297000	2.217532000	-1.179018000
7	1.104745000	-1.112533000	0.428287000
7	-1.647188000	-0.255356000	0.372850000
6	1.140382000	3.117522000	-0.420300000
6	3.286054000	2.556971000	-1.395255000
6	0.658272000	-1.763227000	1.498089000
6	2.522883000	-1.275207000	0.184266000
1	1.393438000	-2.373426000	2.047626000
6	-0.668745000	-1.796309000	2.022663000
6	-1.794079000	-1.074948000	1.464080000
6	-0.867767000	-2.619578000	3.173217000
6	-3.063044000	-1.252384000	2.112878000
6	-2.105625000	-2.762459000	3.778740000
1	0.003595000	-3.150938000	3.574507000
1	-3.929335000	-0.723262000	1.708977000
6	-3.210282000	-2.063529000	3.230536000
6	-2.836580000	0.387584000	-0.143461000
1	-4.199985000	-2.161195000	3.690507000
1	-2.228670000	-3.401047000	4.658081000
6	3.468871000	-0.638035000	1.036346000
6	2.939429000	-2.081712000	-0.913650000
6	4.838294000	-0.824718000	0.756241000
6	3.064318000	0.202085000	2.252641000
6	4.319667000	-2.231118000	-1.143536000
6	1.923993000	-2.834716000	-1.775472000
1	5.581106000	-0.343579000	1.401727000
6	5.266908000	-1.607505000	-0.320560000
1	1.977513000	0.383035000	2.194155000
6	3.755433000	1.582315000	2.282282000
6	3.349018000	-0.559558000	3.570004000
1	6.336668000	-1.737021000	-0.516443000
1	4.657993000	-2.854650000	-1.977566000
1	0.958850000	-2.298741000	-1.684717000
6	1.710091000	-4.267275000	-1.231095000
6	2.292869000	-2.869610000	-3.272265000
1	3.376539000	2.173503000	3.134179000
1	3.567401000	2.149219000	1.357935000
1	4.848505000	1.489736000	2.411233000
1	2.819922000	-1.526345000	3.614423000
1	3.032859000	0.041214000	4.440781000
1	4.428419000	-0.767841000	3.677007000
1	2.648445000	-4.848578000	-1.276888000
1	0.947848000	-4.801517000	-1.825374000
1	1.374131000	-4.252843000	-0.180814000
1	2.476968000	-1.857092000	-3.670700000
1	1.470331000	-3.321184000	-3.853332000
1	3.195350000	-3.476116000	-3.466063000
6	-3.685009000	-0.297899000	-1.065539000
6	-3.149777000	1.715219000	0.280286000
6	-4.808805000	0.380445000	-1.575222000
6	-3.443556000	-1.747604000	-1.494293000
6	-4.292479000	2.340061000	-0.257118000
6	-2.317671000	2.447000000	1.336585000
1	-5.463250000	-0.136064000	-2.286593000
6	-5.115173000	1.688650000	-1.183014000
1	-2.493824000	-2.077731000	-1.041410000
6	-4.562769000	-2.681195000	-0.975814000
6	-3.297147000	-1.878033000	-3.027109000
1	-5.996746000	2.195402000	-1.590058000
1	-4.542783000	3.357797000	0.059637000

1	-1.321136000	1.968872000	1.354180000
6	-2.934112000	2.284187000	2.748185000
6	-2.129638000	3.948366000	1.027387000
1	-4.641237000	-2.641923000	0.122787000
1	-4.355161000	-3.726565000	-1.265042000
1	-5.545179000	-2.405098000	-1.398754000
1	-4.223832000	-1.585437000	-3.551661000
1	-3.074672000	-2.923403000	-3.304534000
1	-2.479105000	-1.241978000	-3.405917000
1	-3.945123000	2.728765000	2.782851000
1	-2.311965000	2.797933000	3.502477000
1	-3.017136000	1.225106000	3.038441000
1	-1.763928000	4.121259000	0.001221000
1	-1.404313000	4.390418000	1.732421000
1	-3.072234000	4.511778000	1.143816000
6	1.676532000	4.318563000	0.066714000
1	0.102106000	2.858318000	-0.192925000
6	3.821541000	3.759893000	-0.914373000
1	3.919952000	1.867780000	-1.964235000
6	3.017162000	4.652459000	-0.185368000
1	1.041585000	4.994760000	0.649169000
1	4.872833000	3.999786000	-1.107368000
1	3.433926000	5.592049000	0.191769000
1	-0.792572000	2.731266000	-2.202094000
1	-0.401935000	2.418973000	-3.907531000
1	-1.748229000	1.547769000	-3.125979000

TS(XI$_Z$-XII)(V)
SCF (BP86/SDD/6-31G**) Energy 333 K = -1787.87326639
Thermal correction to Gibbs Free Energy= 0.672123
Lowest Frequency = -667.6284 cm^{-1}
Second Frequency = 11.3074 cm^{-1}
SCF (B3PW91-D3,C6H6/tzvp) Energy 333 K= -2927.62465202

1	0.330053000	-0.100181000	-2.193523000
6	0.792255000	1.613146000	-2.114159000
1	1.879082000	1.449005000	-2.149287000
6	0.370179000	2.289933000	-0.910191000
6	0.210116000	1.884473000	-3.494594000
26	0.227669000	0.153302000	-0.588136000
1	1.182313000	2.571498000	-0.224673000
6	-0.882727000	3.039078000	-0.706889000
7	1.872128000	-0.592011000	0.324766000
7	-0.975652000	-1.184979000	0.228684000
6	-2.131959000	2.677237000	-1.281501000
6	-0.862792000	4.194569000	0.121498000
6	1.886939000	-1.803311000	0.860064000
6	3.124713000	0.112382000	0.214345000
1	2.869426000	-2.190705000	1.182035000
6	0.778180000	-2.671450000	1.108755000
6	-0.616868000	-2.346725000	0.841555000
6	1.099229000	-3.922064000	1.722187000
6	-1.593751000	-3.313382000	1.265022000
6	0.130399000	-4.840918000	2.089912000
1	2.158243000	-4.140536000	1.906306000
1	-2.648474000	-3.085309000	1.091695000
6	-1.230924000	-4.514563000	1.856796000
6	-2.372257000	-0.881713000	0.061111000
1	-2.015721000	-5.222952000	2.145538000
1	0.405872000	-5.791000000	2.555821000
6	3.443707000	1.092557000	1.196143000
6	3.986658000	-0.151054000	-0.887629000
6	4.661529000	1.785945000	1.065472000
6	2.520219000	1.339678000	2.390249000
6	5.193924000	0.571719000	-0.964085000
6	3.650858000	-1.203991000	-1.947032000
1	4.932486000	2.538177000	1.813120000
6	5.533075000	1.529677000	-0.001322000
1	1.500597000	1.045776000	2.077587000
6	2.465215000	2.817596000	2.827198000
6	2.919538000	0.439597000	3.584597000
1	6.476433000	2.079844000	-0.083125000
1	5.878692000	0.379337000	-1.796809000
1	2.571576000	-1.426844000	-1.865467000
6	4.429740000	-2.516213000	-1.689865000
6	3.903485000	-0.707304000	-3.387097000
1	1.688939000	2.953554000	3.599063000
1	2.226126000	3.484646000	1.981874000
1	3.420612000	3.156270000	3.265604000

1	2.886564000	-0.629790000	3.317684000
1	2.236099000	0.597818000	4.437157000
1	3.945727000	0.671289000	3.921035000
1	5.519268000	-2.343162000	-1.744945000
1	4.167302000	-3.278549000	-2.444108000
1	4.209486000	-2.934863000	-0.693604000
1	3.370559000	0.236146000	-3.591385000
1	3.551032000	-1.460626000	-4.112368000
1	4.976526000	-0.539467000	-3.586096000
6	-3.041187000	-1.279535000	-1.136149000
6	-3.060861000	-0.150810000	1.075470000
6	-4.396214000	-0.928181000	-1.292028000
6	-2.348537000	-2.110072000	-2.219063000
6	-4.415408000	0.168524000	0.866446000
6	-2.377740000	0.254424000	2.382730000
1	-4.923838000	-1.233673000	-2.202812000
6	-5.083340000	-0.211422000	-0.304495000
1	-1.281228000	-2.182324000	-1.948072000
6	-2.926443000	-3.543766000	-2.277808000
6	-2.422688000	-1.435422000	-3.605870000
1	-6.138587000	0.046730000	-0.444881000
1	-4.956148000	0.726729000	1.638413000
1	-1.293836000	0.087618000	2.249336000
6	-2.849331000	-0.640289000	3.554074000
6	-2.583089000	1.746369000	2.721915000
1	-2.826179000	-4.057372000	-1.307496000
1	-2.394053000	-4.143757000	-3.036900000
1	-3.997581000	-3.533305000	-2.548334000
1	-3.464668000	-1.322576000	-3.954487000
1	-1.886157000	-2.042698000	-4.355973000
1	-1.957824000	-0.435494000	-3.586404000
1	-3.934275000	-0.521961000	3.726522000
1	-2.328000000	-0.363068000	4.487506000
1	-2.650623000	-1.706175000	3.355096000
1	-2.252740000	2.400849000	1.899537000
1	-2.009161000	2.009850000	3.627958000
1	-3.642838000	1.975633000	2.931977000
6	-3.282165000	3.446934000	-1.063166000
1	-2.215061000	1.760245000	-1.874053000
6	-2.012841000	4.963692000	0.335260000
1	0.084580000	4.494695000	0.584823000
6	-3.232721000	4.600355000	-0.262492000
1	-4.229087000	3.130979000	-1.513427000
1	-1.955952000	5.854977000	0.970140000
1	-4.133033000	5.200793000	-0.096937000
1	-0.888686000	1.888613000	-3.509381000
1	0.544078000	2.885212000	-3.829695000
1	0.560660000	1.138996000	-4.226368000

TS(XI$_Z$-XII$_Z$)(III)
SCF (BP86/SDD/6-31G**) Energy 333 K = -1787.89725326
Thermal correction to Gibbs Free Energy= 0.678475
Lowest Frequency = -239.2587 cm^{-1}
Second Frequency = 10.8668 cm^{-1}
SCF (B3PW91-D3,C6H6/tzvp) Energy 333 K= -2927.63303238

1	-0.938205000	-0.272417000	-1.960978000
6	0.274825000	0.852377000	-2.589485000
1	0.393201000	0.094046000	-3.377067000
6	1.432114000	0.983589000	-1.741357000
6	-0.621517000	2.004615000	-3.024043000
26	-0.085896000	-0.046992000	-0.740654000
1	2.232763000	0.260562000	-1.947586000
6	1.936888000	2.244881000	-1.143872000
7	1.085431000	-1.132503000	0.420270000
7	-1.654084000	-0.276840000	0.360872000
6	1.132394000	3.157283000	-0.419242000
6	3.302909000	2.579078000	-1.326266000
6	0.626353000	-1.823042000	1.461101000
6	2.506978000	-1.289752000	0.187217000
1	1.358064000	-2.451389000	1.994061000
6	-0.703913000	-1.874537000	1.969345000
6	-1.819071000	-1.127881000	1.422553000
6	-0.918619000	-2.739218000	3.086969000
6	-3.096772000	-1.323664000	2.051369000
6	-2.163713000	-2.899360000	3.671514000
1	-0.053514000	-3.287880000	3.478552000
1	-3.956859000	-0.776663000	1.658252000
6	-3.259332000	-2.175315000	3.135658000
6	-2.834924000	0.397372000	-0.133870000

1	-4.254736000	-2.285953000	3.580280000
1	-2.300386000	-3.569330000	4.525102000
6	3.441883000	-0.659533000	1.055455000
6	2.935416000	-2.085520000	-0.913258000
6	4.814565000	-0.844899000	0.791067000
6	3.018240000	0.170036000	2.272180000
6	4.318328000	-2.233741000	-1.128308000
6	1.928353000	-2.822846000	-1.798155000
1	5.549844000	-0.370035000	1.449655000
6	5.255518000	-1.619077000	-0.287261000
1	1.933891000	0.357948000	2.192215000
6	3.716915000	1.545501000	2.331443000
6	3.270145000	-0.608346000	3.586404000
1	6.327516000	-1.748161000	-0.470828000
1	4.666676000	-2.848665000	-1.964636000
1	0.963590000	-2.285692000	-1.707698000
6	1.705568000	-4.264302000	-1.281848000
6	2.313893000	-2.832070000	-3.291281000
1	3.323016000	2.129824000	3.181233000
1	3.552253000	2.123424000	1.409303000
1	4.806392000	1.445146000	2.483275000
1	2.731371000	-1.570436000	3.609382000
1	2.941694000	-0.014345000	4.457251000
1	4.345420000	-0.827235000	3.712390000
1	2.643526000	-4.846188000	-1.327203000
1	0.949512000	-4.786673000	-1.894311000
1	1.357479000	-4.267977000	-0.235499000
1	2.504968000	-1.813090000	-3.669521000
1	1.497703000	-3.272112000	-3.889911000
1	3.217636000	-3.436670000	-3.484819000
6	-3.672585000	-0.242447000	-1.096871000
6	-3.148798000	1.704889000	0.345490000
6	-4.798722000	0.452994000	-1.576128000
6	-3.405075000	-1.661728000	-1.603962000
6	-4.291740000	2.351150000	-0.166702000
6	-2.309702000	2.394318000	1.424507000
1	-5.447613000	-0.030573000	-2.315320000
6	-5.112454000	1.739273000	-1.120913000
1	-2.445846000	-1.994098000	-1.172264000
6	-4.501718000	-2.645255000	-1.132175000
6	-3.262182000	-1.706800000	-3.141881000
1	-5.995904000	2.260890000	-1.504453000
1	-4.543620000	3.354167000	0.193083000
1	-1.311960000	1.917957000	1.412673000
6	-2.912564000	2.175156000	2.834069000
6	-2.124905000	3.906861000	1.172715000
1	-4.575692000	-2.665359000	-0.032716000
1	-4.275074000	-3.669506000	-1.477447000
1	-5.491350000	-2.366241000	-1.536158000
1	-4.196629000	-1.406618000	-3.648024000
1	-3.018179000	-2.730612000	-3.475991000
1	-2.459942000	-1.033635000	-3.489723000
1	-3.923063000	2.618083000	2.895898000
1	-2.283339000	2.658305000	3.602610000
1	-2.993878000	1.105282000	3.082176000
1	-1.773080000	4.119710000	0.149016000
1	-1.389611000	4.320606000	1.884485000
1	-3.065610000	4.465536000	1.323080000
6	1.658478000	4.361373000	0.070306000
1	0.086690000	2.904628000	-0.218732000
6	3.828903000	3.785132000	-0.842191000
1	3.950741000	1.881458000	-1.868747000
6	3.007502000	4.687674000	-0.145475000
1	1.009270000	5.045762000	0.626967000
1	4.886158000	4.019533000	-1.007590000
1	3.416717000	5.629683000	0.233927000
1	-0.823001000	2.724736000	-2.218619000
1	-0.130474000	2.557757000	-3.846728000
1	-1.591828000	1.634405000	-3.392216000

XII (V)
SCF (BP86/SDD/6-31G**) Energy 333 K = -1787.92420489
Thermal correction to Gibbs Free Energy= 0.678245
Lowest Frequency = 11.1535 cm^{-1}
Second Frequency = 13.8929 cm^{-1}
SCF (B3PW91-D3,C6H6/tzvp) Energy 333 K= -2927.67435209

1	1.439355000	2.502827000	2.070905000
6	0.410896000	2.381625000	2.461001000
1	0.468535000	1.545175000	3.182993000

6	-0.552173000	1.989671000	1.327662000
6	-0.004191000	3.662115000	3.218261000
26	-0.098699000	0.231422000	0.412823000
1	-1.554190000	1.752298000	1.745065000
6	-0.709800000	2.941228000	0.190589000
7	-1.418644000	-1.190132000	-0.042315000
7	1.466584000	-0.808677000	-0.137700000
6	0.348718000	3.776069000	-0.265032000
6	-1.956417000	3.051774000	-0.489050000
6	-1.071410000	-2.384889000	-0.507251000
6	-2.822763000	-0.918588000	0.129469000
1	-1.887011000	-3.101964000	-0.701579000
6	0.249520000	-2.859544000	-0.787378000
6	1.476607000	-2.097952000	-0.609768000
6	0.330597000	-4.198818000	-1.280928000
6	2.703257000	-2.763482000	-0.948044000
6	1.534158000	-4.806666000	-1.599070000
1	-0.609327000	-4.750040000	-1.406243000
1	3.638180000	-2.212925000	-0.815803000
6	2.730831000	-4.067684000	-1.423960000
6	2.712896000	-0.097664000	-0.022437000
1	3.696126000	-4.527438000	-1.663762000
1	1.560440000	-5.833211000	-1.975462000
6	-3.641881000	-0.664309000	-1.006884000
6	-3.348831000	-0.863588000	1.453069000
6	-5.000337000	-0.361047000	-0.784446000
6	-3.109924000	-0.727769000	-2.441272000
6	-4.711403000	-0.552798000	1.613119000
6	-2.478247000	-1.213139000	2.661696000
1	-5.648981000	-0.166762000	-1.645728000
6	-5.534860000	-0.301287000	0.507161000
1	-2.013064000	-0.837880000	-2.392765000
6	-3.402529000	0.564181000	-3.235743000
6	-3.671596000	-1.962568000	-3.185079000
1	-6.593167000	-0.061254000	0.654074000
1	-5.137285000	-0.511741000	2.620509000
1	-1.432139000	-0.960207000	2.394233000
6	-2.522172000	-2.734918000	2.940702000

6	-2.832017000	-0.417430000	3.933331000
1	-2.964903000	0.494421000	-4.246871000
1	-2.970964000	1.447154000	-2.737133000
1	-4.486847000	0.733904000	-3.357823000
1	-3.424904000	-2.901882000	-2.661666000
1	-3.257901000	-2.021606000	-4.206935000
1	-4.771525000	-1.908388000	-3.268447000
1	-3.549350000	-3.052825000	3.192925000
1	-1.865182000	-2.995974000	3.788964000
1	-2.194138000	-3.316742000	2.063637000
1	-2.841041000	0.669401000	3.745943000
1	-2.091347000	-0.623024000	4.724909000
1	-3.820210000	-0.700896000	4.336493000
6	3.367197000	-0.021097000	1.246550000
6	3.256786000	0.564802000	-1.163975000
6	4.555967000	0.727202000	1.341582000
6	2.824830000	-0.765677000	2.469857000
6	4.450227000	1.296282000	-1.009119000
6	2.600290000	0.486055000	-2.544594000
1	5.068702000	0.796880000	2.306203000
6	5.098559000	1.382278000	0.228125000
1	1.728660000	-0.853729000	2.333834000
6	3.394239000	-2.203413000	2.551200000
6	3.076843000	-0.030346000	3.802451000
1	6.025703000	1.957096000	0.326431000
1	4.877728000	1.806788000	-1.879630000
1	1.654431000	-0.071671000	-2.431670000
6	3.485983000	-0.290729000	-3.547184000
6	2.253298000	1.884165000	-3.102125000
1	3.169632000	-2.786533000	1.644479000
1	2.969760000	-2.739361000	3.418804000
1	4.491870000	-2.174764000	2.674634000
1	4.146267000	-0.038237000	4.078539000
1	2.533041000	-0.535198000	4.619586000
1	2.743919000	1.019515000	3.764302000
1	4.451854000	0.221571000	-3.705498000
1	2.982436000	-0.365358000	-4.527143000
1	3.695482000	-1.313347000	-3.193947000

1	1.596158000	2.444153000	-2.417140000
1	1.736707000	1.790160000	-4.073628000
1	3.162210000	2.489351000	-3.269740000
6	0.164181000	4.673692000	-1.325999000
1	1.324394000	3.724878000	0.229465000
6	-2.135332000	3.946281000	-1.552081000
1	-2.797088000	2.433188000	-0.151720000
6	-1.076688000	4.767319000	-1.979294000
1	0.998021000	5.310396000	-1.642292000
1	-3.114264000	4.016219000	-2.039585000
1	-1.218771000	5.474004000	-2.803182000
1	-0.048504000	4.527586000	2.535831000
1	-1.004616000	3.543720000	3.670764000
1	0.707863000	3.900940000	4.027887000

XII$_Z$ (III)
SCF (BP86/SDD/6-31G**) Energy 333 K = -1787.91048005
Thermal correction to Gibbs Free Energy= 0.680789
Lowest Frequency = 13.0788 cm^{-1}
Second Frequency = 19.0134 cm^{-1}
SCF (B3PW91-D3,C6H6/tzvp) Energy 333 K= -2927.65085588

1	-0.974903000	0.961579000	-1.704088000
6	-0.075227000	1.337964000	-2.363994000
1	-0.126724000	0.656084000	-3.233186000
6	1.251486000	1.148199000	-1.653233000
6	-0.537405000	2.745216000	-2.788665000
26	-0.009013000	0.009516000	-0.578005000
1	1.923699000	0.475607000	-2.210801000
6	1.995607000	2.286727000	-1.057850000
7	1.049119000	-1.132859000	0.475346000
7	-1.670816000	-0.240834000	0.401755000
6	1.404482000	3.200141000	-0.147486000
6	3.354942000	2.499230000	-1.400027000
6	0.607944000	-1.738509000	1.579483000
6	2.433771000	-1.374731000	0.126262000
1	1.341523000	-2.353087000	2.122925000
6	-0.713267000	-1.727456000	2.112902000
6	-1.836743000	-1.033924000	1.510207000
6	-0.925116000	-2.510474000	3.290468000
6	-3.121883000	-1.238933000	2.118709000
6	-2.174850000	-2.657646000	3.869024000
1	-0.055077000	-3.013662000	3.729514000
1	-3.991341000	-0.755938000	1.666028000
6	-3.282478000	-2.015987000	3.258650000
6	-2.860462000	0.353085000	-0.160307000
1	-4.283926000	-2.131031000	3.688433000
1	-2.307019000	-3.264869000	4.769152000
6	3.479716000	-0.862352000	0.944449000
6	2.714926000	-2.133610000	-1.046818000
6	4.809014000	-1.131344000	0.557777000
6	3.229175000	-0.059167000	2.224789000
6	4.060306000	-2.363438000	-1.385844000
6	1.589213000	-2.753102000	-1.876858000
1	5.627526000	-0.747261000	1.176205000
6	5.104441000	-1.868933000	-0.592470000
1	2.152895000	0.178080000	2.273833000
6	3.992422000	1.283069000	2.238193000
6	3.591027000	-0.888481000	3.480636000
1	6.145829000	-2.062315000	-0.870994000
1	4.295531000	-2.949015000	-2.280186000
1	0.698237000	-2.101154000	-1.753401000
6	1.208102000	-4.146565000	-1.322563000
6	1.896969000	-2.830668000	-3.384945000
1	3.727603000	1.856758000	3.143520000
1	3.747963000	1.895898000	1.357439000
1	5.086206000	1.130397000	2.256556000
1	3.026380000	-1.834930000	3.532348000
1	3.376555000	-0.313838000	4.398627000
1	4.665334000	-1.144863000	3.486670000
1	2.064576000	-4.840033000	-1.396752000
1	0.367553000	-4.578930000	-1.893527000
1	0.908394000	-4.090105000	-0.263311000
1	2.209269000	-1.852883000	-3.789857000
1	1.000469000	-3.161060000	-3.936994000
1	2.697062000	-3.558527000	-3.607419000
6	-3.568373000	-0.331957000	-1.193324000
6	-3.303125000	1.631984000	0.294127000
6	-4.707463000	0.278582000	-1.751972000
6	-3.135717000	-1.714530000	-1.685072000

6	-4.451814000	2.194017000	-0.297177000
6	-2.580601000	2.390199000	1.410094000
1	-5.259755000	-0.242905000	-2.542174000
6	-5.154146000	1.530272000	-1.310458000
1	-2.155568000	-1.933099000	-1.223723000
6	-4.124236000	-2.809006000	-1.219212000
6	-2.951899000	-1.761296000	-3.218126000
1	-6.046378000	1.985953000	-1.753046000
1	-4.803741000	3.172734000	0.047524000
1	-1.606515000	1.891137000	1.562308000
6	-3.361149000	2.318456000	2.744579000
6	-2.308451000	3.865124000	1.039622000
1	-4.215681000	-2.821390000	-0.120912000
1	-3.783205000	-3.807198000	-1.546455000
1	-5.130351000	-2.640285000	-1.643419000
1	-3.903511000	-1.588408000	-3.750930000
1	-2.576347000	-2.751415000	-3.530774000
1	-2.234258000	-0.997632000	-3.564471000
1	-4.354810000	2.791162000	2.643138000
1	-2.813722000	2.851900000	3.541848000
1	-3.508997000	1.276933000	3.071050000
1	-1.784613000	3.956768000	0.073251000
1	-1.687391000	4.345746000	1.815665000
1	-3.243465000	4.448156000	0.965768000
6	2.124427000	4.287803000	0.365654000
1	0.369219000	3.038136000	0.173415000
6	4.078772000	3.584010000	-0.883849000
1	3.841001000	1.801458000	-2.091750000
6	3.465095000	4.491695000	-0.004370000
1	1.637601000	4.977088000	1.064361000
1	5.126490000	3.722559000	-1.172834000
1	4.026881000	5.341939000	0.395996000
1	-0.520469000	3.453203000	-1.947375000
1	0.144896000	3.132744000	-3.563563000
1	-1.559311000	2.720251000	-3.201244000

TS(XII_Z–XII_E)(III)
SCF (BP86/SDD/6-31G**) Energy 333 K = -1787.90776298
Thermal correction to Gibbs Free Energy= 0.680299
Lowest Frequency = -111.6602 cm^{-1}
Second Frequency = 10.5424 cm^{-1}

SCF (B3PW91-D3,C6H6/tzvp) Energy 333 K= -2927.64737826

1	0.652504000	0.991976000	2.388350000
6	0.054762000	1.833394000	1.951870000
6	-1.285953000	1.290097000	1.465453000
26	0.069350000	0.183122000	0.433120000
1	-1.792711000	0.665038000	2.217161000
6	-2.225403000	2.206570000	0.786845000
7	-0.903942000	-1.248484000	-0.273314000
6	-0.350292000	-2.209946000	-1.023817000
1	-1.038160000	-2.995046000	-1.372122000
6	1.007552000	-2.349038000	-1.426610000
6	2.062821000	-1.420870000	-1.066976000
6	1.327729000	-3.486436000	-2.232593000
6	3.383635000	-1.710485000	-1.549019000
7	1.784112000	-0.325718000	-0.296826000
1	4.192355000	-1.022933000	-1.286926000
6	3.647185000	-2.827748000	-2.329907000
1	4.670817000	-3.007615000	-2.677111000
6	2.613576000	-3.734657000	-2.682845000
1	2.829742000	-4.612416000	-3.298724000
1	0.513184000	-4.173554000	-2.492059000
6	-2.331652000	-1.376219000	-0.062227000
6	-3.225025000	-0.993092000	-1.099083000
6	-2.802308000	-1.901937000	1.173562000
6	-4.606339000	-1.161253000	-0.872825000
6	-2.736581000	-0.449918000	-2.444539000
1	-5.311879000	-0.878776000	-1.661629000
6	-5.091241000	-1.676755000	0.333859000
1	-6.168858000	-1.799371000	0.486673000
6	-4.192233000	-2.038193000	1.347637000
1	-4.577239000	-2.446517000	2.287674000
6	-1.827118000	-2.366333000	2.255841000
1	-1.660888000	-0.224151000	-2.343252000
6	-3.442901000	0.861489000	-2.849448000
6	-2.892679000	-1.513137000	-3.558118000
1	-3.009486000	1.246091000	-3.789417000
1	-3.334001000	1.635489000	-2.074127000
1	-4.522032000	0.707830000	-3.027631000

1	-2.339982000	-2.438536000	-3.324138000
1	-2.514065000	-1.125275000	-4.520091000
1	-3.954317000	-1.784635000	-3.697211000
1	-0.894564000	-1.784080000	2.116739000
6	-1.470238000	-3.860709000	2.069206000
6	-2.329208000	-2.108263000	3.690925000
1	-2.371367000	-4.491703000	2.168658000
1	-0.739838000	-4.185748000	2.831216000
1	-1.031898000	-4.050729000	1.075775000
1	-2.630804000	-1.056972000	3.836896000
1	-1.531909000	-2.340373000	4.417878000
1	-3.193895000	-2.745320000	3.948200000
6	2.864113000	0.558861000	0.055669000
6	3.586600000	0.345779000	1.268937000
6	3.172066000	1.668065000	-0.788351000
6	4.607405000	1.253384000	1.610784000
6	3.300621000	-0.853226000	2.175991000
1	5.173199000	1.097282000	2.536095000
6	4.918705000	2.345172000	0.790120000
1	5.719024000	3.037008000	1.073893000
6	4.203155000	2.544136000	-0.397059000
1	4.452707000	3.395675000	-1.040165000
6	2.436428000	1.906461000	-2.108618000
1	2.345438000	-1.295385000	1.839163000
6	4.394794000	-1.937488000	2.029134000
6	3.137072000	-0.453433000	3.658975000
1	4.478041000	-2.283392000	0.986266000
1	4.162381000	-2.811893000	2.662490000
1	5.380175000	-1.546398000	2.340368000
1	4.078066000	-0.064602000	4.086396000
1	2.843576000	-1.331019000	4.260938000
1	2.366476000	0.325273000	3.792929000
1	1.584437000	1.204466000	-2.139319000
6	3.346241000	1.596830000	-3.320853000
6	1.867138000	3.338225000	-2.211809000
1	4.225255000	2.266027000	-3.338058000
1	2.793914000	1.741385000	-4.266130000
1	3.709225000	0.556618000	-3.293895000
1	1.204565000	3.572926000	-1.361367000
1	1.280355000	3.451858000	-3.139778000
1	2.667790000	4.098553000	-2.232078000
6	-1.801604000	3.351571000	0.059699000
6	-3.624235000	1.971830000	0.865947000
6	-2.724838000	4.218139000	-0.541975000
1	-0.732015000	3.573864000	-0.028524000
6	-4.545485000	2.836448000	0.262547000
1	-3.980641000	1.092439000	1.414099000
6	-4.104479000	3.970269000	-0.442867000
1	-2.363102000	5.095930000	-1.089001000
1	-5.617399000	2.626224000	0.347290000
1	-4.824969000	4.650009000	-0.909222000
1	0.654294000	2.213622000	1.080167000
6	0.034397000	2.962766000	3.009499000
1	-0.535572000	3.823668000	2.625401000
1	-0.461118000	2.607204000	3.927858000
1	1.053787000	3.296119000	3.265507000

XII_E (III)
SCF (BP86/SDD/6-31G**) Energy 333 K = -1787.91438826
Thermal correction to Gibbs Free Energy= 0.680981
Lowest Frequency = 9.8900 cm^{-1}
Second Frequency = 17.3129 cm^{-1}
SCF (B3PW91-D3,C6H6/tzvp) Energy 333 K= -2927.65508296

1	1.003966000	1.074277000	1.660442000
6	0.110081000	1.633115000	2.172580000
6	-1.223642000	1.205451000	1.606440000
26	0.050767000	0.070083000	0.546456000
1	-1.745565000	0.493929000	2.272179000
6	-2.162080000	2.196170000	1.031623000
7	-0.956557000	-1.141156000	-0.474987000
6	-0.481755000	-1.790044000	-1.540428000
1	-1.186877000	-2.461804000	-2.052825000
6	0.835890000	-1.739376000	-2.078961000
6	1.921094000	-0.957904000	-1.516159000
6	1.082243000	-2.551872000	-3.229431000
6	3.203737000	-1.083126000	-2.151095000
7	1.723271000	-0.155957000	-0.420044000
1	4.043252000	-0.518712000	-1.737753000
6	3.397119000	-1.885081000	-3.268334000

1	4.393795000	-1.937144000	-3.720821000
6	2.328836000	-2.631895000	-3.827468000
1	2.487919000	-3.261380000	-4.707729000
1	0.241417000	-3.127662000	-3.634868000
6	-2.345494000	-1.383325000	-0.148143000
6	-3.373553000	-0.931591000	-1.022991000
6	-2.649885000	-2.072496000	1.061677000
6	-4.710444000	-1.184436000	-0.651457000
6	-3.095739000	-0.216911000	-2.349864000
1	-5.515772000	-0.845905000	-1.312394000
6	-5.029308000	-1.848697000	0.536548000
1	-6.076030000	-2.030455000	0.802637000
6	-4.001774000	-2.287468000	1.383750000
1	-4.256003000	-2.818869000	2.306176000
6	-1.538001000	-2.637690000	1.947138000
1	-2.016527000	0.005641000	-2.399496000
6	-3.841264000	1.129788000	-2.467684000
6	-3.446549000	-1.128278000	-3.550800000
1	-3.571838000	1.625154000	-3.417072000
1	-3.586513000	1.807045000	-1.638728000
1	-4.937041000	0.991922000	-2.470011000
1	-2.894168000	-2.083094000	-3.523911000
1	-3.207100000	-0.622925000	-4.502706000
1	-4.523920000	-1.371256000	-3.560016000
1	-0.657136000	-1.970098000	1.828335000
6	-1.108358000	-4.039587000	1.452325000
6	-1.890584000	-2.674896000	3.446681000
1	-1.951143000	-4.749801000	1.523979000
1	-0.276918000	-4.432463000	2.063705000
1	-0.776405000	-4.011742000	0.401684000
1	-2.239823000	-1.693562000	3.810748000
1	-1.004003000	-2.965089000	4.036031000
1	-2.678765000	-3.416224000	3.667494000
6	2.886254000	0.500479000	0.127532000
6	3.682227000	-0.172051000	1.102570000
6	3.211128000	1.828329000	-0.282466000
6	4.791805000	0.501332000	1.648543000
6	3.371367000	-1.600635000	1.553744000

1	5.410671000	-0.008767000	2.395812000
6	5.124116000	1.802043000	1.249963000
1	5.994893000	2.306543000	1.682234000
6	4.334990000	2.452917000	0.293485000
1	4.597831000	3.469986000	-0.017538000
6	2.387538000	2.567494000	-1.339151000
1	2.421215000	-1.896750000	1.075416000
6	4.461387000	-2.590770000	1.081964000
6	3.173335000	-1.694526000	3.083167000
1	4.569248000	-2.570720000	-0.014683000
1	4.205145000	-3.622245000	1.382063000
1	5.442511000	-2.345127000	1.526519000
1	4.095778000	-1.433082000	3.631149000
1	2.897508000	-2.723452000	3.374028000
1	2.376430000	-1.014824000	3.430449000
1	1.431153000	2.022935000	-1.441566000
6	3.089568000	2.546713000	-2.718205000
6	2.064096000	4.021089000	-0.930335000
1	4.063973000	3.065650000	-2.669127000
1	2.471041000	3.059333000	-3.476177000
1	3.268593000	1.516096000	-3.064012000
1	1.603104000	4.073061000	0.071133000
1	1.366252000	4.475267000	-1.655105000
1	2.968394000	4.654880000	-0.910813000
6	-1.728259000	3.328380000	0.294979000
6	-3.557808000	2.048340000	1.236759000
6	-2.641110000	4.276716000	-0.185676000
1	-0.659582000	3.466460000	0.096048000
6	-4.470774000	2.997058000	0.755991000
1	-3.921332000	1.176376000	1.792448000
6	-4.019211000	4.122925000	0.046900000
1	-2.273557000	5.143689000	-0.745939000
1	-5.541705000	2.857713000	0.940532000
1	-4.730415000	4.868132000	-0.323944000
1	0.354101000	2.680890000	1.926116000
6	0.317540000	1.358382000	3.674447000
1	-0.355338000	2.002632000	4.264719000
1	0.080350000	0.309568000	3.922239000

1	1.355429000	1.559170000	3.989325000

TS(XII–XI$_E$)(V)
SCF (BP86/SDD/6-31G**) Energy 333 K = -1787.87791019
Thermal correction to Gibbs Free Energy= 0.674409
Lowest Frequency = -722.3485 cm^{-1}
Second Frequency = 13.9242 cm^{-1}
SCF (B3PW91-D3,C6H6/tzvp) Energy 333 K= -2927.62963320

6	0.291070000	1.360081000	-2.400515000
1	0.658617000	-0.272216000	-2.198223000
6	0.054000000	2.158638000	-1.210601000
1	0.947540000	2.661139000	-0.806281000
26	0.283306000	0.108222000	-0.666762000
6	-1.213691000	2.835850000	-0.895364000
7	1.914622000	-0.426589000	0.422359000
6	1.905322000	-1.537440000	1.132495000
1	2.858074000	-1.828463000	1.608737000
6	0.809191000	-2.432643000	1.372483000
6	-0.567550000	-2.228783000	0.943999000
6	1.140636000	-3.595234000	2.132359000
6	-1.510373000	-3.239727000	1.332995000
7	-0.957346000	-1.125511000	0.232423000
1	-2.552281000	-3.110486000	1.030636000
6	-1.137364000	-4.360001000	2.065525000
1	-1.901032000	-5.102006000	2.324848000
6	0.202579000	-4.556016000	2.478556000
1	0.489135000	-5.438353000	3.057091000
1	2.185038000	-3.716596000	2.444347000
6	3.160868000	0.286291000	0.293171000
6	3.340276000	1.482736000	1.044299000
6	4.156982000	-0.192090000	-0.604517000
6	4.550530000	2.183207000	0.889566000
6	2.276789000	1.949531000	2.039126000
1	4.714847000	3.101774000	1.461173000
6	5.550571000	1.723956000	0.021583000
1	6.486119000	2.283690000	-0.081925000
6	5.350527000	0.549911000	-0.712901000
1	6.135652000	0.197886000	-1.390528000
6	3.981317000	-1.476359000	-1.419450000
1	1.295074000	1.621736000	1.645292000
6	2.220673000	3.480642000	2.208285000
6	2.477429000	1.267088000	3.414267000
1	1.341486000	3.759603000	2.813014000
1	2.146390000	3.997687000	1.236646000
1	3.110983000	3.871931000	2.732081000
1	2.438117000	0.168031000	3.335924000
1	1.692833000	1.584399000	4.123115000
1	3.457758000	1.539880000	3.843601000
1	2.914672000	-1.758419000	-1.376782000
6	4.810852000	-2.634846000	-0.815656000
6	4.338297000	-1.286191000	-2.910051000
1	5.889179000	-2.396357000	-0.831504000
1	4.660058000	-3.563133000	-1.393914000
1	4.532750000	-2.838356000	0.232214000
1	3.761326000	-0.462018000	-3.359214000
1	4.112201000	-2.208653000	-3.472280000
1	5.411429000	-1.069917000	-3.054355000
6	-2.360323000	-0.937472000	-0.028920000
6	-2.914344000	-1.366075000	-1.273767000
6	-3.176577000	-0.302943000	0.954638000
6	-4.286391000	-1.145618000	-1.505329000
6	-2.079490000	-2.104201000	-2.323601000
1	-4.727423000	-1.476146000	-2.452130000
6	-5.098277000	-0.526599000	-0.546939000
1	-6.163792000	-0.369672000	-0.746751000
6	-4.541285000	-0.113021000	0.669320000
1	-5.178256000	0.372158000	1.417115000
6	-2.620247000	0.146836000	2.306667000
1	-1.014120000	-1.952388000	-2.068804000
6	-2.358798000	-3.625997000	-2.286387000
6	-2.291496000	-1.557014000	-3.751525000
1	-2.144808000	-4.051853000	-1.292665000
1	-1.730565000	-4.153582000	-3.025910000
1	-3.416175000	-3.837831000	-2.527541000
1	-3.314905000	-1.747164000	-4.120607000
1	-1.595039000	-2.047712000	-4.453331000
1	-2.114672000	-0.469276000	-3.802308000
1	-1.525224000	0.012709000	2.272306000
6	-3.168967000	-0.729707000	3.457541000

6	-2.898731000	1.640268000	2.582949000
1	-4.266701000	-0.634580000	3.538988000
1	-2.733493000	-0.415449000	4.422611000
1	-2.929676000	-1.795027000	3.306223000
1	-2.503890000	2.283017000	1.780481000
1	-2.428696000	1.942440000	3.535852000
1	-3.980788000	1.841510000	2.674287000
6	-2.468287000	2.451442000	-1.440736000
6	-1.201082000	3.974707000	-0.043872000
6	-3.634422000	3.174258000	-1.159368000
1	-2.537647000	1.567156000	-2.083216000
6	-2.367554000	4.698723000	0.230643000
1	-0.246847000	4.300776000	0.386576000
6	-3.597179000	4.306425000	-0.327335000
1	-4.584060000	2.843910000	-1.593825000
1	-2.315286000	5.579399000	0.880882000
1	-4.510444000	4.871477000	-0.114887000
1	-0.595091000	1.175609000	-3.025418000
6	1.553610000	1.623470000	-3.215079000
1	1.459533000	2.596353000	-3.733487000
1	2.443867000	1.676537000	-2.565826000
1	1.726830000	0.839255000	-3.971330000

TS(XII_E–XI_E)(III)
SCF (BP86/SDD/6-31G**) Energy 333 K = -1787.90015911
Thermal correction to Gibbs Free Energy= 0.679548
Lowest Frequency = -481.0200 cm^{-1}
Second Frequency = 8.1928 cm^{-1}
SCF (B3PW91-D3,C6H6/tzvp) Energy 333 K= -2927.63673991

1	-0.970613000	-0.200101000	-2.021300000
6	0.184716000	0.792402000	-2.678932000
6	1.368374000	0.919719000	-1.864517000
26	-0.110029000	-0.058761000	-0.770019000
1	-0.448230000	1.691083000	-2.751025000
6	0.246038000	0.005064000	-3.985846000
1	2.180255000	0.211768000	-2.079748000
6	1.832317000	2.200212000	-1.289310000
7	1.049346000	-1.134754000	0.402495000
7	-1.681128000	-0.286618000	0.333486000
6	0.973151000	3.302578000	-1.045856000
6	3.215790000	2.377035000	-1.029387000
6	0.561209000	-1.896844000	1.381320000
6	2.494250000	-1.177281000	0.295909000
1	1.288400000	-2.531560000	1.912699000
6	-0.780101000	-1.990986000	1.848224000
6	-1.872327000	-1.176179000	1.354449000
6	-1.020855000	-2.922619000	2.905712000
6	-3.145483000	-1.340077000	2.002866000
6	-2.266607000	-3.071001000	3.490966000
1	-0.174116000	-3.526022000	3.254772000
1	-3.980355000	-0.719311000	1.669361000
6	-3.332343000	-2.255032000	3.029359000
6	-2.824001000	0.481609000	-0.105679000
1	-4.323100000	-2.343230000	3.489170000
1	-2.424791000	-3.792225000	4.297789000
6	3.286279000	-0.482478000	1.253408000
6	3.091167000	-1.925528000	-0.758574000
6	4.688421000	-0.532868000	1.110004000
6	2.679746000	0.258659000	2.450434000
6	4.495219000	-1.934396000	-0.855190000
6	2.234315000	-2.759876000	-1.712361000
1	5.315208000	-0.005754000	1.837314000
6	5.292635000	-1.240537000	0.065706000
1	1.597543000	0.369784000	2.266239000
6	3.257792000	1.676336000	2.647104000
6	2.857833000	-0.565667000	3.749265000
1	6.383779000	-1.262445000	-0.026310000
1	4.973748000	-2.502938000	-1.658717000
1	1.257972000	-2.242708000	-1.802788000
6	1.956592000	-4.160887000	-1.114477000
6	2.834186000	-2.898096000	-3.125922000
1	2.734435000	2.181059000	3.477864000
1	3.142903000	2.290247000	1.740814000
1	4.330547000	1.650055000	2.909293000
1	2.395772000	-1.564010000	3.674737000
1	2.398174000	-0.042106000	4.605786000

1	3.929075000	-0.710995000	3.975918000
1	2.900390000	-4.716682000	-0.971758000
1	1.311038000	-4.750625000	-1.789123000
1	1.452016000	-4.093485000	-0.137038000
1	3.101313000	-1.919432000	-3.559818000
1	2.107746000	-3.385488000	-3.798852000
1	3.742884000	-3.526015000	-3.128552000
6	-3.753996000	-0.080239000	-1.031513000
6	-3.000464000	1.810719000	0.386692000
6	-4.847430000	0.704670000	-1.444914000
6	-3.611231000	-1.502684000	-1.578844000
6	-4.113054000	2.549155000	-0.062970000
6	-2.035182000	2.421890000	1.405907000
1	-5.569670000	0.280935000	-2.152082000
6	-5.033552000	2.007340000	-0.968156000
1	-2.654163000	-1.907843000	-1.208161000
6	-4.744543000	-2.426771000	-1.074766000
6	-3.557377000	-1.517400000	-3.123005000
1	-5.892826000	2.599526000	-1.300862000
1	-4.260950000	3.569332000	0.305021000
1	-1.047533000	1.953234000	1.234953000
6	-2.462710000	2.093555000	2.857637000
6	-1.868500000	3.948005000	1.252003000
1	-4.759887000	-2.481518000	0.025459000
1	-4.607654000	-3.450953000	-1.464574000
1	-5.732728000	-2.068054000	-1.414297000
1	-4.505682000	-1.167091000	-3.567384000
1	-3.376112000	-2.541893000	-3.492782000
1	-2.750603000	-0.868068000	-3.502727000
1	-3.453358000	2.532153000	3.075409000
1	-1.739643000	2.516621000	3.577446000
1	-2.526998000	1.008545000	3.033773000
1	-1.664229000	4.239790000	0.208441000
1	-1.028808000	4.299083000	1.875112000
1	-2.769310000	4.494611000	1.584155000
6	1.476308000	4.523553000	-0.577851000
1	-0.101851000	3.205392000	-1.232994000
6	3.718069000	3.602811000	-0.571419000
1	3.899328000	1.539307000	-1.206159000
6	2.852287000	4.685585000	-0.342057000
1	0.787971000	5.357165000	-0.401548000
1	4.793630000	3.712816000	-0.394665000
1	3.244179000	5.643870000	0.013886000
1	0.760895000	0.612051000	-4.752909000
1	0.811268000	-0.932346000	-3.862732000
1	-0.755662000	-0.248840000	-4.370404000

XI_E (V)
SCF (BP86/SDD/6-31G**) Energy 333 K = -1787.88632353
Thermal correction to Gibbs Free Energy= 0.677246
Lowest Frequency = 15.4589 cm^{-1}
Second Frequency = 22.3761 cm^{-1}
SCF (B3PW91-D3,C6H6/tzvp) Energy 333 K= -2927.63746137

6	-0.275677000	1.084002000	2.605483000
1	-0.872563000	-1.282645000	2.015411000
6	-0.244127000	1.951205000	1.482758000
1	-1.213161000	2.355684000	1.154038000
26	-0.314854000	-0.195099000	0.949462000
6	0.929425000	2.728394000	1.032951000
7	-1.840323000	-0.399819000	-0.414556000
6	-1.770361000	-1.430433000	-1.237849000
1	-2.693451000	-1.687600000	-1.786416000
6	-0.649078000	-2.279039000	-1.526509000
6	0.715006000	-2.092132000	-1.049434000
6	-0.934594000	-3.362330000	-2.413552000
6	1.693005000	-3.027856000	-1.532005000
7	1.057769000	-1.076304000	-0.198861000
1	2.724567000	-2.908699000	-1.191760000
6	1.362581000	-4.073745000	-2.383296000
1	2.148149000	-4.769142000	-2.700024000
6	0.034128000	-4.256985000	-2.840091000
1	-0.219481000	-5.080254000	-3.513436000
1	-1.969097000	-3.474792000	-2.759717000
6	-3.125645000	0.242699000	-0.278923000
6	-3.338344000	1.486138000	-0.940856000
6	-4.139308000	-0.360693000	0.519619000

6	-4.585244000	2.117761000	-0.778049000
6	-2.270940000	2.077879000	-1.863096000
1	-4.772650000	3.071521000	-1.280377000
6	-5.593855000	1.545502000	0.008615000
1	-6.556908000	2.054540000	0.121766000
6	-5.368428000	0.319393000	0.642326000
1	-6.163715000	-0.128393000	1.247750000
6	-3.958545000	-1.720628000	1.200196000
1	-1.285157000	1.815928000	-1.433794000
6	-2.345588000	3.613180000	-1.984380000
6	-2.347692000	1.442732000	-3.273848000
1	-1.454567000	3.993228000	-2.511660000
1	-2.398401000	4.104759000	-0.997915000
1	-3.227384000	3.936289000	-2.565968000
1	-2.208270000	0.350104000	-3.242742000
1	-1.566311000	1.863671000	-3.930496000
1	-3.329689000	1.645931000	-3.736578000
1	-2.881922000	-1.964985000	1.190055000
6	-4.718824000	-2.826288000	0.428971000
6	-4.395328000	-1.709300000	2.681261000
1	-5.804808000	-2.624059000	0.415635000
1	-4.562417000	-3.809097000	0.907011000
1	-4.384189000	-2.902210000	-0.619544000
1	-3.864010000	-0.929973000	3.250445000
1	-4.167885000	-2.683943000	3.146455000
1	-5.480341000	-1.537375000	2.794691000
6	2.451306000	-0.809916000	0.024106000
6	3.063293000	-1.204014000	1.254537000
6	3.203999000	-0.118418000	-0.974153000
6	4.420668000	-0.887325000	1.459212000
6	2.303928000	-2.015498000	2.307031000
1	4.903487000	-1.189026000	2.394573000
6	5.168036000	-0.209459000	0.487658000
1	6.223827000	0.021213000	0.666681000
6	4.558258000	0.164506000	-0.715564000
1	5.143747000	0.694939000	-1.475015000
6	2.597822000	0.314334000	-2.311654000
1	1.220544000	-1.830928000	2.162325000
6	2.532982000	-3.532670000	2.103563000
6	2.643840000	-1.612814000	3.756739000
1	2.217292000	-3.858049000	1.099070000
1	1.957463000	-4.113595000	2.845763000
1	3.601714000	-3.786905000	2.224847000
1	3.675264000	-1.891858000	4.037233000
1	1.966885000	-2.129465000	4.458482000
1	2.536326000	-0.526261000	3.918644000
1	1.528683000	0.044399000	-2.298500000
6	3.257694000	-0.427808000	-3.497475000
6	2.684984000	1.842733000	-2.515693000
1	4.332465000	-0.183808000	-3.573040000
1	2.781248000	-0.131757000	-4.448707000
1	3.162284000	-1.520940000	-3.394791000
1	2.188167000	2.389961000	-1.699547000
1	2.204138000	2.126093000	-3.469017000
1	3.733606000	2.186057000	-2.560776000
6	2.259305000	2.444099000	1.436830000
6	0.720291000	3.884194000	0.237319000
6	3.318388000	3.286440000	1.076861000
1	2.473790000	1.551926000	2.033464000
6	1.780159000	4.730037000	-0.115884000
1	-0.298801000	4.132479000	-0.077497000
6	3.088330000	4.438413000	0.304924000
1	4.333420000	3.035474000	1.401154000
1	1.581522000	5.623997000	-0.717190000
1	3.918632000	5.097805000	0.032140000
1	0.674307000	0.887989000	3.124889000
6	-1.509654000	0.985238000	3.483055000
1	-1.520714000	1.824290000	4.208046000
1	-2.434968000	1.054584000	2.886290000
1	-1.533762000	0.039172000	4.045325000

XI_E (III)
SCF (BP86/SDD/6-31G**) Energy 333 K = -1787.89949587
Thermal correction to Gibbs Free Energy= 0.679555
Lowest Frequency = 7.2232 cm^{-1}
Second Frequency = 14.5502 cm^{-1}
SCF (B3PW91-D3,C6H6/tzvp) Energy 333 K= -2927.63210775

6	0.019708000	0.927479000	-2.572013000
1	-0.959553000	-0.808732000	-1.810020000

6	1.276822000	0.998261000	-1.901125000
1	2.038475000	0.278578000	-2.234257000
26	-0.170784000	-0.074506000	-0.787289000
6	1.844298000	2.192975000	-1.242666000
7	1.072222000	-1.106524000	0.401348000
6	0.618843000	-1.781456000	1.453935000
1	1.348934000	-2.411872000	1.987189000
6	-0.700359000	-1.796967000	1.999916000
6	-1.810756000	-1.015533000	1.495928000
6	-0.897520000	-2.633931000	3.140758000
6	-3.054691000	-1.121259000	2.205096000
7	-1.666841000	-0.198495000	0.403418000
1	-3.902024000	-0.527346000	1.854485000
6	-3.198522000	-1.942471000	3.315763000
1	-4.167834000	-1.984372000	3.824888000
6	-2.115403000	-2.720823000	3.795791000
1	-2.236973000	-3.369748000	4.667733000
1	-0.039766000	-3.215963000	3.498605000
6	2.501062000	-1.208591000	0.202268000
6	3.391535000	-0.612373000	1.142537000
6	2.991103000	-1.909234000	-0.938726000
6	4.776153000	-0.702621000	0.887250000
6	2.924127000	0.073242000	2.432840000
1	5.475379000	-0.248011000	1.597565000
6	5.273253000	-1.356588000	-0.243794000
1	6.352737000	-1.409056000	-0.421094000
6	4.381887000	-1.961341000	-1.141790000
1	4.776209000	-2.495363000	-2.011812000
6	2.036452000	-2.665435000	-1.864652000
1	1.827767000	0.183624000	2.387173000
6	3.512448000	1.488670000	2.617084000
6	3.260314000	-0.800024000	3.666617000
1	3.103981000	1.947048000	3.534765000
1	3.270266000	2.141686000	1.765226000
1	4.611338000	1.464942000	2.726320000
1	2.813648000	-1.806426000	3.599079000
1	2.889023000	-0.323550000	4.590780000
1	4.352182000	-0.930770000	3.769112000
1	1.081983000	-2.101556000	-1.879562000
6	1.724945000	-4.068395000	-1.288233000
6	2.538271000	-2.785908000	-3.316744000
1	2.645024000	-4.676222000	-1.223290000
1	1.006324000	-4.603133000	-1.934136000
1	1.289128000	-4.003679000	-0.277877000
1	2.823344000	-1.806866000	-3.738652000
1	1.746489000	-3.215704000	-3.954048000
1	3.413270000	-3.455337000	-3.398553000
6	-2.844548000	0.467856000	-0.104734000
6	-3.779887000	-0.240796000	-0.918951000
6	-3.050535000	1.846560000	0.210279000
6	-4.896152000	0.457437000	-1.418383000
6	-3.632774000	-1.727824000	-1.253456000
1	-5.619101000	-0.076160000	-2.045830000
6	-5.104726000	1.809585000	-1.125775000
1	-5.981091000	2.331990000	-1.524221000
6	-4.187794000	2.490250000	-0.315544000
1	-4.358835000	3.546173000	-0.084896000
6	-2.096672000	2.609451000	1.133987000
1	-2.686278000	-2.081958000	-0.812310000
6	-4.781916000	-2.563138000	-0.641254000
6	-3.550428000	-1.966475000	-2.777936000
1	-4.824421000	-2.448635000	0.453794000
1	-4.637822000	-3.634692000	-0.865660000
1	-5.761185000	-2.263537000	-1.055283000
1	-4.475799000	-1.649412000	-3.290369000
1	-3.402460000	-3.039757000	-2.991295000
1	-2.708969000	-1.410601000	-3.223847000
1	-1.087257000	2.182737000	0.983029000
6	-2.466968000	2.404229000	2.623769000
6	-2.024371000	4.120354000	0.830000000
1	-3.477381000	2.802257000	2.827862000
1	-1.753520000	2.940318000	3.274485000
1	-2.457384000	1.341055000	2.909391000
1	-1.869456000	4.324927000	-0.243146000
1	-1.190394000	4.575062000	1.390731000
1	-2.945809000	4.645204000	1.139680000

6	1.072728000	3.328760000	-0.889094000
6	3.246226000	2.261436000	-1.037426000
6	1.675250000	4.479018000	-0.364109000
1	-0.010089000	3.317070000	-1.045796000
6	3.849043000	3.418835000	-0.525863000
1	3.864031000	1.396283000	-1.302207000
6	3.068255000	4.535761000	-0.183405000
1	1.052843000	5.343127000	-0.107383000
1	4.936122000	3.447531000	-0.393886000
1	3.538703000	5.440497000	0.215091000
1	-0.669643000	1.777823000	-2.437012000
6	-0.119452000	0.241539000	-3.922650000
1	0.211905000	0.928537000	-4.725468000
1	0.503568000	-0.665642000	-3.983979000
1	-1.162399000	-0.043168000	-4.137926000

Allylbenzene 3a
SCF (BP86/SDD/6-31G**) Energy 333 K = -348.954743822
Thermal correction to Gibbs Free Energy= 0.117888
Lowest Frequency = 33.3707 cm^{-1}
Second Frequency = 81.9136 cm^{-1}
SCF (B3PW91-D3,C6H6/tzvp) Energy 333 K= -348.946537529

6	-3.490979000	0.546000000	-0.053113000
1	-3.705929000	0.633765000	1.018528000
1	-4.187230000	1.041435000	-0.737045000
6	-2.429457000	-0.143818000	-0.501742000
1	-2.242833000	-0.204267000	-1.583129000
6	-1.428596000	-0.857998000	0.376265000
1	-1.726377000	-0.736321000	1.435851000
1	-1.463316000	-1.945146000	0.167585000
6	0.006367000	-0.375214000	0.183755000
6	0.309167000	1.001646000	0.207215000
6	1.058989000	-1.291160000	-0.004943000
6	1.629626000	1.448673000	0.055017000
6	2.382301000	-0.847670000	-0.157527000
6	2.671971000	0.524934000	-0.127452000
1	-0.504408000	1.723682000	0.339267000
1	0.838185000	-2.365011000	-0.030653000
1	1.845956000	2.522221000	0.076624000
1	3.186788000	-1.576426000	-0.303534000
1	3.702830000	0.873714000	-0.248355000

(*E*)-prop-1-en-1-ylbenzene (*E*)-4a
SCF (BP86/SDD/6-31G**) Energy 333 K = -348.966435268
Thermal correction to Gibbs Free Energy= 0.117592
Lowest Frequency = 34.8912 cm^{-1}
Second Frequency = 120.5826 cm^{-1}
SCF (B3PW91-D3,C6H6/tzvp) Energy 333 K= -348.955015176

6	-1.386766000	-0.502927000	-0.000200000
6	-2.412839000	0.377864000	0.000137000
1	-2.202408000	1.456445000	0.000429000
6	-3.864573000	-0.007144000	0.000099000
1	-1.635673000	-1.574625000	-0.000502000
6	0.055264000	-0.207118000	-0.000134000
1	-4.389758000	0.400915000	-0.884274000
1	-3.995232000	-1.102002000	-0.000697000
1	-4.389435000	0.399610000	0.885266000
6	0.584510000	1.107011000	-0.000141000
6	0.972108000	-1.285330000	-0.000008000
6	1.966094000	1.326949000	-0.000021000
1	-0.093772000	1.966547000	-0.000306000
6	2.356286000	-1.066428000	0.000107000
1	0.583470000	-2.310505000	0.000002000
6	2.861504000	0.242542000	0.000113000
1	2.349791000	2.352845000	-0.000047000
1	3.041536000	-1.920728000	0.000172000
1	3.941947000	0.418977000	0.000246000

(*Z*)-prop-1-en-1-ylbenzene (*Z*)-4a
SCF (BP86/SDD/6-31G**) Energy 333 K = -348.962146418
Thermal correction to Gibbs Free Energy= 0.118894
Lowest Frequency = 52.5246 cm^{-1}
Second Frequency = 126.0065 cm^{-1}
SCF (B3PW91-D3,C6H6/tzvp) Energy 333 K= -348.951932590

6	1.386440000	-1.031222000	-0.338992000
6	2.597362000	-0.474562000	-0.091624000
1	3.473031000	-1.107198000	-0.290504000
6	2.905034000	0.889019000	0.465948000
1	1.386632000	-2.078871000	-0.670745000
6	0.040340000	-0.448537000	-0.182135000
1	3.232155000	1.592068000	-0.324834000
1	2.038177000	1.338731000	0.975298000
1	3.737852000	0.828364000	1.188774000
6	-0.244604000	0.927304000	-0.361870000
6	-1.041093000	-1.309880000	0.127756000

6	-1.546772000	1.421118000	-0.200193000
1	0.553999000	1.607960000	-0.668897000
6	-2.340840000	-0.816394000	0.293444000
1	-0.846007000	-2.381578000	0.250242000
6	-2.599544000	0.555312000	0.135959000
1	-1.742101000	2.488269000	-0.350890000
1	-3.156026000	-1.504334000	0.541812000
1	-3.615646000	0.943636000	0.259984000

c. DFT Calculations for [(^{Me}BDI^{iPr})Fe-H]$_2$ catalyzed isomerization of allylbenzene 3a

[(^{Me}BDI^{iPr})Fe-H]$_2$ (VII)
SCF (BP86/SDD/6-31G**) Energy 333 K = -2727.87913102
Thermal correction to Gibbs Free Energy= 1.112066
Lowest Frequency = 14.3156 cm^{-1}
Second Frequency = 14.9933 cm^{-1}
SCF (B3PW91-D3,C6H6/tzvp) Energy 333 K= -5007.41813878

26	-1.045842000	0.748339000	-0.083703000
1	-0.518171000	-0.432844000	-1.058521000
1	0.276432000	0.400643000	0.845155000
26	0.999091000	-0.714484000	-0.252549000
7	-2.627799000	1.050653000	-1.199286000
7	-1.162274000	2.450783000	0.902754000
7	1.224170000	-2.667742000	0.250523000
7	2.574906000	-0.643708000	-1.474710000
6	-4.060278000	2.528297000	-2.603085000
1	-3.604165000	2.250201000	-3.568893000
1	-4.357710000	3.586139000	-2.655010000
1	-4.967542000	1.912128000	-2.496945000
6	-3.075948000	2.299925000	-1.465246000
6	-2.710618000	3.430070000	-0.701691000
1	-3.187900000	4.368923000	-0.992482000
6	-1.945996000	3.474751000	0.482999000
6	-2.060171000	4.742749000	1.315230000
1	-2.376246000	4.506018000	2.345437000
1	-2.795293000	5.431321000	0.873205000
1	-1.095789000	5.268806000	1.400202000
6	-3.415086000	-0.049525000	-1.719143000
6	-3.062466000	-0.728847000	-2.920308000
6	-3.900336000	-1.763442000	-3.383421000
1	-3.635955000	-2.282567000	-4.311675000
6	-5.065419000	-2.128440000	-2.701345000
1	-5.708116000	-2.926281000	-3.088739000
6	-5.398404000	-1.462585000	-1.516469000
1	-6.307342000	-1.747969000	-0.974832000
6	-4.591713000	-0.430242000	-1.001062000
6	-1.830848000	-0.370349000	-3.750273000
1	-1.307834000	0.448126000	-3.227162000
6	-0.857931000	-1.566814000	-3.856021000
1	-0.533962000	-1.906364000	-2.858322000
1	0.041045000	-1.285967000	-4.432254000
1	-1.328752000	-2.422271000	-4.372480000
6	-2.219207000	0.123104000	-5.164021000
1	-2.724527000	-0.671168000	-5.741442000
1	-1.320203000	0.422208000	-5.731070000
1	-2.901864000	0.988881000	-5.128601000
6	-5.020007000	0.247050000	0.304719000
1	-4.230517000	0.964077000	0.586938000
6	-5.155760000	-0.777482000	1.453334000
1	-5.951609000	-1.515357000	1.248786000
1	-5.416252000	-0.265150000	2.396073000
1	-4.216216000	-1.331745000	1.609912000
6	-6.337531000	1.040215000	0.139219000
1	-6.251115000	1.823991000	-0.631115000
1	-6.613961000	1.530581000	1.089130000
1	-7.170925000	0.375179000	-0.149648000
6	-0.499076000	2.607726000	2.180847000
6	-1.048287000	1.982224000	3.336825000
6	-0.397523000	2.152931000	4.574799000
1	-0.822792000	1.681944000	5.468146000
6	0.771481000	2.913139000	4.687113000
1	1.261260000	3.036565000	5.658949000
6	1.310302000	3.509899000	3.541812000
1	2.233223000	4.094965000	3.623211000
6	0.697531000	3.376124000	2.280957000
6	-2.333193000	1.154947000	3.290712000
1	-2.622297000	1.046182000	2.228972000
6	-3.489444000	1.881316000	4.016643000

1	-3.251866000	2.035835000	5.084313000
1	-4.419540000	1.288432000	3.962696000
1	-3.690989000	2.870966000	3.572570000
6	-2.127394000	-0.262895000	3.866370000
1	-1.321971000	-0.800677000	3.339793000
1	-3.053850000	-0.854854000	3.772122000
1	-1.864169000	-0.232377000	4.938397000
6	1.376584000	4.006914000	1.064535000
1	0.690523000	3.915031000	0.205013000
6	1.705172000	5.504975000	1.256365000
1	0.816880000	6.101335000	1.527487000
1	2.122742000	5.923974000	0.324409000
1	2.458362000	5.657238000	2.049404000
6	2.661369000	3.224055000	0.720817000
1	3.390871000	3.290361000	1.547783000
1	3.140781000	3.620921000	-0.189443000
1	2.437743000	2.158768000	0.548773000
6	2.103700000	-4.968529000	-0.113237000
1	2.261210000	-5.074998000	0.972152000
1	2.950498000	-5.429364000	-0.643323000
1	1.193685000	-5.542362000	-0.360545000
6	1.955690000	-3.505683000	-0.501698000
6	2.629812000	-3.086534000	-1.681591000
1	3.055365000	-3.895073000	-2.281794000
6	2.955697000	-1.786904000	-2.115325000
6	3.831631000	-1.681974000	-3.355722000
1	3.341475000	-1.055372000	-4.120593000
1	4.022783000	-2.675621000	-3.787076000
1	4.799338000	-1.202285000	-3.134807000
6	0.606087000	-3.141575000	1.460839000
6	1.191047000	-2.808010000	2.718202000
6	0.564269000	-3.262827000	3.894508000
1	1.015085000	-3.023717000	4.863820000
6	-0.616291000	-4.013185000	3.849646000
1	-1.088180000	-4.356841000	4.776242000
6	-1.191297000	-4.316135000	2.609758000
1	-2.121908000	-4.893370000	2.574785000
6	-0.603742000	-3.895654000	1.401414000

6	2.497231000	-2.020110000	2.824135000
1	2.723098000	-1.610879000	1.821500000
6	2.390257000	-0.828146000	3.798922000
1	1.567784000	-0.148806000	3.521572000
1	3.329012000	-0.248210000	3.794634000
1	2.217469000	-1.162606000	4.837141000
6	3.668735000	-2.950568000	3.217225000
1	3.493130000	-3.406968000	4.207786000
1	4.616080000	-2.385605000	3.269345000
1	3.799627000	-3.769354000	2.489531000
6	-1.305014000	-4.220003000	0.081573000
1	-0.625541000	-3.945091000	-0.743302000
6	-1.650046000	-5.719870000	-0.061832000
1	-0.767918000	-6.367661000	0.080335000
1	-2.064952000	-5.921060000	-1.064652000
1	-2.410428000	-6.033586000	0.674876000
6	-2.579000000	-3.362624000	-0.071142000
1	-3.301199000	-3.585935000	0.734107000
1	-3.075954000	-3.554616000	-1.036813000
1	-2.338944000	-2.287656000	-0.027546000
6	3.359994000	0.539795000	-1.735882000
6	2.917553000	1.541250000	-2.645316000
6	3.750865000	2.652073000	-2.887094000
1	3.416517000	3.421811000	-3.591901000
6	4.994398000	2.787344000	-2.261027000
1	5.631170000	3.652590000	-2.474104000
6	5.410632000	1.808404000	-1.350638000
1	6.377601000	1.917703000	-0.846299000
6	4.614250000	0.684078000	-1.064164000
6	1.576778000	1.459086000	-3.371141000
1	1.112034000	0.494472000	-3.101802000
6	0.629616000	2.585425000	-2.899185000
1	0.435452000	2.509696000	-1.814480000
1	-0.340682000	2.529535000	-3.422357000
1	1.062095000	3.582067000	-3.099146000
6	1.746475000	1.497965000	-4.906856000
1	0.769417000	1.377372000	-5.406341000
1	2.414347000	0.696581000	-5.266964000

1	2.171794000	2.459450000	-5.244786000
6	5.121060000	-0.333372000	-0.037277000
1	4.347068000	-1.111260000	0.076247000
6	6.422629000	-1.029395000	-0.498086000
1	6.288666000	-1.557103000	-1.456989000
1	6.749613000	-1.771020000	0.251792000
1	7.243344000	-0.301627000	-0.627655000
6	5.326248000	0.326510000	1.345324000
1	6.106711000	1.107119000	1.309355000
1	5.644552000	-0.426095000	2.088268000
1	4.397375000	0.797732000	1.705851000

XII (V)
SCF (BP86/SDD/6-31G**) Energy 333 K = -1363.92086840
Thermal correction to Gibbs Free Energy= 0.533386
Lowest Frequency = 9.1721 cm^{-1}
Second Frequency = 19.4779 cm^{-1}
SCF (B3PW91-D3,C6H6/tzvp) Energy 333 K= -2503.67580058

1	0.000029000	0.000571000	-2.386758000
26	0.000025000	0.000152000	-0.772297000
7	-1.449355000	-0.000341000	0.559607000
7	1.449336000	0.000175000	0.559644000
6	-2.496704000	0.000893000	2.805660000
1	-3.129691000	0.885260000	2.617071000
1	-2.203030000	0.000240000	3.865676000
1	-3.131267000	-0.882167000	2.616324000
6	-1.276500000	0.000184000	1.901343000
6	-0.000026000	0.000057000	2.509874000
1	-0.000037000	0.000151000	3.602632000
6	1.276458000	-0.000111000	1.901378000
6	2.496648000	-0.000771000	2.805714000
1	3.129458000	-0.885319000	2.617363000
1	2.202961000	0.000208000	3.865726000
1	3.131392000	0.882107000	2.616151000
6	-2.770186000	0.000260000	-0.011804000
6	-3.398685000	1.240290000	-0.325817000
6	-4.659604000	1.211820000	-0.950523000
1	-5.152497000	2.157405000	-1.201950000
6	-5.291488000	0.001627000	-1.262171000
1	-6.272276000	0.002168000	-1.749390000
6	-4.659736000	-1.209201000	-0.952913000
1	-5.152693000	-2.154288000	-1.206162000
6	-3.398774000	-1.239038000	-0.328279000
6	-2.716417000	2.579910000	-0.045137000
1	-1.847668000	2.381279000	0.607047000
6	-2.179389000	3.196731000	-1.358375000
1	-1.488687000	2.504553000	-1.871838000
1	-1.641560000	4.140224000	-1.157957000
1	-3.006371000	3.417557000	-2.056412000
6	-3.634553000	3.576246000	0.695660000
1	-4.499650000	3.874936000	0.078067000
1	-3.077894000	4.496131000	0.945693000
1	-4.026559000	3.149098000	1.634544000
6	-2.717450000	-2.579545000	-0.049649000
1	-1.844957000	-2.381699000	0.597750000
6	-3.634015000	-3.572768000	0.697352000
1	-4.019191000	-3.143813000	1.638240000
1	-3.078641000	-4.494145000	0.944780000
1	-4.503688000	-3.869127000	0.085077000
6	-2.188581000	-3.199375000	-1.364744000
1	-3.019525000	-3.419715000	-2.058215000
1	-1.651700000	-4.143680000	-1.165625000
1	-1.499076000	-2.509392000	-1.882620000
6	2.770163000	-0.000343000	-0.011780000
6	3.398703000	-1.240389000	-0.325729000
6	4.659579000	-1.211925000	-0.950504000
1	5.152495000	-2.157504000	-1.201899000
6	5.291405000	-0.001723000	-1.262278000
1	6.272165000	-0.002260000	-1.749552000
6	4.659623000	1.209094000	-0.953082000
1	5.152519000	2.154191000	-1.206423000
6	3.398683000	1.238938000	-0.328387000
6	2.716446000	-2.579989000	-0.044938000
1	1.848058000	-2.381388000	0.607736000
6	2.178653000	-3.196467000	-1.358026000
1	1.487773000	-2.504091000	-1.870988000
1	1.640811000	-4.139938000	-1.157540000
1	3.005252000	-3.417219000	-2.056541000
6	3.634840000	-3.576590000	0.695165000

1	3.078159000	-4.496405000	0.945407000
1	4.027480000	-3.149634000	1.633872000
1	4.499519000	-3.875387000	0.077036000
6	2.717422000	2.579489000	-0.049802000
1	1.844468000	2.381616000	0.596967000
6	3.633760000	3.572238000	0.698131000
1	4.018061000	3.142964000	1.639231000
1	3.078536000	4.493770000	0.945318000
1	4.504014000	3.868358000	0.086565000
6	2.189560000	3.199908000	-1.365019000
1	3.020995000	3.420362000	-2.057866000
1	1.652758000	4.144259000	-1.165896000
1	1.500238000	2.510274000	-1.883588000

XII (III)
SCF (BP86/SDD/6-31G**) Energy 333 K = -1363.90354666
Thermal correction to Gibbs Free Energy= 0.537114
Lowest Frequency = 8.4659 cm^{-1}
Second Frequency = 19.6033 cm^{-1}
SCF (B3PW91-D3,C6H6/tzvp) Energy 333 K= -2503.64152584

1	-0.526682000	0.525749000	-1.997873000
26	0.100239000	-0.017383000	-0.640972000
7	-1.331131000	0.034527000	0.533414000
7	1.445776000	0.008392000	0.676798000
6	-2.491123000	0.209740000	2.716305000
1	-3.060499000	1.125400000	2.482729000
1	-2.249845000	0.215225000	3.789577000
1	-3.166630000	-0.637748000	2.510850000
6	-1.220403000	0.120619000	1.887560000
6	0.010089000	0.117196000	2.580049000
1	-0.047461000	0.166310000	3.669985000
6	1.300056000	0.056658000	2.015613000
6	2.522244000	0.043609000	2.910772000
1	3.140687000	-0.852772000	2.727569000
1	2.233792000	0.056374000	3.972489000
1	3.168024000	0.917940000	2.715366000
6	-2.641828000	0.028615000	-0.074061000
6	-3.272060000	1.262120000	-0.407956000
6	-4.551890000	1.219237000	-0.990551000
1	-5.047687000	2.159991000	-1.255032000
6	-5.199891000	0.003930000	-1.245350000
1	-6.197287000	-0.004642000	-1.697818000
6	-4.560429000	-1.199460000	-0.926516000
1	-5.062631000	-2.150399000	-1.137540000
6	-3.279249000	-1.215732000	-0.342690000
6	-2.590591000	2.614730000	-0.196030000
1	-1.633976000	2.429449000	0.322348000
6	-2.259488000	3.272154000	-1.555932000
1	-1.627412000	2.603799000	-2.163229000
1	-1.721316000	4.224703000	-1.404014000
1	-3.180106000	3.492201000	-2.125411000
6	-3.430407000	3.569540000	0.682113000
1	-4.388609000	3.828567000	0.198045000
1	-2.882552000	4.512511000	0.854607000
1	-3.664991000	3.129948000	1.666934000
6	-2.607604000	-2.557777000	-0.048933000
1	-1.628841000	-2.345433000	0.416064000
6	-3.423645000	-3.417937000	0.942036000
1	-3.590004000	-2.893239000	1.898277000
1	-2.894286000	-4.361910000	1.161433000
1	-4.413756000	-3.680492000	0.529404000
6	-2.344078000	-3.334560000	-1.359525000
1	-3.289778000	-3.594939000	-1.866974000
1	-1.804795000	-4.276141000	-1.153181000
1	-1.741918000	-2.734468000	-2.062093000
6	2.706508000	-0.049827000	0.001363000
6	3.243528000	-1.323254000	-0.367572000
6	4.429153000	-1.356188000	-1.122646000
1	4.854324000	-2.321891000	-1.414604000
6	5.077112000	-0.174301000	-1.508033000
1	5.998966000	-0.222455000	-2.097355000
6	4.542629000	1.066768000	-1.141544000
1	5.052273000	1.985764000	-1.451243000
6	3.357886000	1.160553000	-0.387159000
6	2.517932000	-2.614580000	0.009924000
1	1.887430000	-2.393771000	0.889888000
6	1.572110000	-3.050943000	-1.133469000
1	0.822326000	-2.264473000	-1.363946000
1	1.015588000	-3.965182000	-0.863648000

1	2.136587000	-3.245996000	-2.062036000
6	3.470530000	-3.765760000	0.394029000
1	2.891184000	-4.626190000	0.770320000
1	4.180003000	-3.460196000	1.181343000
1	4.057866000	-4.124247000	-0.469401000
6	2.793134000	2.533106000	-0.022249000
1	1.944515000	2.371100000	0.664759000
6	3.830476000	3.415652000	0.707686000
1	4.233008000	2.916688000	1.605988000
1	3.367274000	4.366175000	1.024249000
1	4.685145000	3.667259000	0.055362000
6	2.240137000	3.250739000	-1.275604000
1	3.036294000	3.419116000	-2.022608000
1	1.817473000	4.233917000	-1.003518000
1	1.444550000	2.652011000	-1.752073000

XIII (V)
SCF (BP86/SDD/6-31G**) Energy 333 K = -1712.87702695
Thermal correction to Gibbs Free Energy= 0.680172
Lowest Frequency = 9.8711 cm^{-1}
Second Frequency = 11.9802 cm^{-1}
SCF (B3PW91-D3,C6H6/tzvp) Energy 333 K= -2852.63964921

1	-0.173777000	0.151235000	-2.208878000
6	1.041317000	1.519437000	0.060227000
26	-0.158724000	-0.148389000	-0.604797000
6	-0.113740000	1.454630000	0.843352000
1	-0.971516000	2.107471000	0.644082000
1	-0.113850000	0.941369000	1.810142000
7	-2.066134000	-0.747975000	-0.176065000
7	0.679921000	-1.901452000	-0.083364000
1	1.951943000	1.023283000	0.429702000
6	1.271376000	2.571656000	-1.018890000
1	1.749074000	2.113112000	-1.901546000
1	0.295101000	2.965122000	-1.354654000
6	2.148164000	3.708449000	-0.500965000
6	-2.384420000	-2.052934000	-0.267870000
6	-3.100895000	0.238845000	-0.013603000
6	-0.021494000	-3.051450000	-0.148556000
6	2.102052000	-1.912777000	0.125380000
6	-1.427952000	-3.100709000	-0.282028000
6	-3.840105000	-2.480949000	-0.390322000
6	-3.689173000	0.867043000	-1.150129000
6	-3.484989000	0.622691000	1.308621000
1	-1.848798000	-4.106191000	-0.362618000
1	-4.135487000	-2.484366000	-1.455413000
1	-3.987566000	-3.500123000	-0.001315000
1	-4.520186000	-1.791199000	0.130449000
6	0.713452000	-4.380972000	-0.094522000
1	0.015687000	-5.211709000	0.086838000
1	1.234884000	-4.572855000	-1.048986000
1	1.485322000	-4.381693000	0.691627000
6	2.610819000	-1.737481000	1.447711000
6	2.989326000	-2.018931000	-0.987396000
6	-4.654508000	1.871591000	-0.935050000
6	-3.330379000	0.482890000	-2.587543000
6	-4.462779000	1.623501000	1.461517000
6	-2.890377000	-0.050664000	2.549296000
1	-5.112350000	2.359686000	-1.802927000
6	-5.045669000	2.251040000	0.353120000
1	-2.571923000	-0.317749000	-2.544746000
6	-2.691469000	1.665543000	-3.348759000
6	-4.560755000	-0.051554000	-3.357706000
1	-4.772577000	1.917414000	2.470311000
1	-1.974626000	-0.576792000	2.225486000
6	-3.855819000	-1.109258000	3.135139000
6	-2.496302000	0.959448000	3.649887000
1	-5.802224000	3.030222000	0.494552000
1	-2.435031000	1.364204000	-4.379607000
1	-1.761461000	1.991148000	-2.854798000
1	-3.378775000	2.528351000	-3.409548000
1	-5.039821000	-0.896023000	-2.833835000
1	-4.264237000	-0.396610000	-4.363676000
1	-5.327830000	0.732401000	-3.488765000
1	-4.806051000	-0.640820000	3.448038000
1	-3.409787000	-1.594292000	4.021547000
1	-4.094964000	-1.897920000	2.403934000
1	-1.840352000	1.756235000	3.262355000
1	-1.963965000	0.442604000	4.467427000
1	-3.381642000	1.443934000	4.097988000

6	4.005983000	-1.691150000	1.631244000
6	1.685092000	-1.627902000	2.661931000
6	4.375433000	-1.955601000	-0.744472000
6	2.491423000	-2.189603000	-2.424918000
1	4.407616000	-1.568103000	2.643207000
6	4.887537000	-1.796858000	0.548539000
1	0.655638000	-1.509675000	2.279008000
6	1.721568000	-2.917307000	3.516055000
6	2.009575000	-0.397955000	3.539259000
1	5.066759000	-2.034139000	-1.591087000
1	1.394562000	-2.308872000	-2.392304000
6	3.092452000	-3.443249000	-3.102063000
6	2.781787000	-0.929571000	-3.271646000
1	5.969553000	-1.754106000	0.712201000
1	1.426617000	-3.803967000	2.930705000
1	1.032896000	-2.835091000	4.375397000
1	2.736220000	-3.099406000	3.912641000
1	3.004682000	-0.483094000	4.010116000
1	1.270560000	-0.302763000	4.353932000
1	1.997935000	0.537111000	2.954639000
1	4.183565000	-3.345855000	-3.242045000
1	2.644274000	-3.590583000	-4.099961000
1	2.917340000	-4.358209000	-2.510447000
1	2.255537000	-0.053657000	-2.857772000
1	2.428718000	-1.072413000	-4.308110000
1	3.864012000	-0.710507000	-3.307207000
6	3.512232000	3.780744000	-0.845841000
6	1.616683000	4.697533000	0.352559000
6	4.325790000	4.814739000	-0.354637000
1	3.937949000	3.020790000	-1.511830000
6	2.425093000	5.733312000	0.843273000
1	0.557348000	4.653629000	0.631335000
6	3.784214000	5.794935000	0.491479000
1	5.383237000	4.856007000	-0.637584000
1	1.992971000	6.496582000	1.499582000
1	4.415822000	6.604075000	0.873112000

XIII (III)
SCF (BP86/SDD/6-31G**) Energy 333 K = -1712.88462591
Thermal correction to Gibbs Free Energy= 0.684581
Lowest Frequency = 11.6078 cm^{-1}
Second Frequency = 15.8675 cm^{-1}
SCF (B3PW91-D3,C6H6/tzvp) Energy 333 K= -2852.63244936

1	-0.809020000	0.959034000	0.366786000
6	-0.284271000	1.485843000	-1.546342000
6	0.808189000	0.706993000	-1.989977000
1	1.824297000	1.109992000	-1.942130000
1	0.654216000	-0.107390000	-2.711484000
26	0.266953000	0.017384000	-0.131275000
7	2.075297000	-0.392785000	0.547155000
6	2.333141000	-1.357331000	1.464412000
6	1.364545000	-2.282741000	1.905447000
6	3.702125000	-1.441852000	2.124205000
6	0.002345000	-2.360872000	1.556755000
1	1.712873000	-3.029083000	2.623824000
7	-0.589817000	-1.479103000	0.717368000
6	-0.813181000	-3.475564000	2.191408000
6	3.177230000	0.448278000	0.144234000
6	3.426442000	1.663955000	0.843375000
6	3.984043000	0.070619000	-0.972334000
6	4.494219000	2.478016000	0.415172000
6	2.572896000	2.113657000	2.031888000
1	4.692988000	3.416612000	0.944859000
6	5.306016000	2.111168000	-0.663687000
1	6.135571000	2.754870000	-0.975047000
6	5.045289000	0.916566000	-1.347442000
1	5.676775000	0.634925000	-2.197113000
6	3.723150000	-1.216763000	-1.762235000
1	1.866255000	1.297853000	2.262891000
6	1.731373000	3.360604000	1.676403000
6	3.424116000	2.381570000	3.294133000
1	1.113249000	3.670218000	2.537495000
1	1.054187000	3.152483000	0.831714000
1	2.376065000	4.213292000	1.397964000
1	4.040436000	1.508361000	3.568260000
1	2.772509000	2.621895000	4.152285000
1	4.108257000	3.236470000	3.149984000

1	2.708666000	-1.560146000	-1.492723000
6	4.718078000	-2.340321000	-1.384109000
6	3.754694000	-0.992607000	-3.291266000
1	5.758190000	-2.028834000	-1.587958000
1	4.516227000	-3.250096000	-1.976880000
1	4.649197000	-2.613374000	-0.319262000
1	3.084740000	-0.174099000	-3.600678000
1	3.438717000	-1.911897000	-3.814721000
1	4.770650000	-0.749842000	-3.649841000
6	-1.995184000	-1.625970000	0.421563000
6	-2.378012000	-2.304668000	-0.774728000
6	-2.977840000	-1.080753000	1.299288000
6	-3.751311000	-2.422256000	-1.067725000
6	-1.349453000	-2.943183000	-1.713784000
1	-4.059682000	-2.948524000	-1.977842000
6	-4.727054000	-1.885112000	-0.219157000
1	-5.789178000	-1.987118000	-0.466192000
6	-4.334917000	-1.220742000	0.950449000
1	-5.099961000	-0.803437000	1.614934000
6	-2.609451000	-0.349807000	2.593135000
1	-0.355228000	-2.568534000	-1.411468000
6	-1.339661000	-4.483889000	-1.571205000
6	-1.566118000	-2.551573000	-3.192037000
1	-1.109645000	-4.799277000	-0.540331000
1	-0.580518000	-4.930154000	-2.237645000
1	-2.321112000	-4.911954000	-1.842885000
1	-2.521886000	-2.940117000	-3.585795000
1	-0.760576000	-2.972528000	-3.818850000
1	-1.568514000	-1.457622000	-3.327889000
1	-1.516318000	-0.428395000	2.721116000
6	-3.285886000	-0.983365000	3.831386000
6	-2.957957000	1.153746000	2.512821000
1	-4.382984000	-0.860953000	3.798731000
1	-2.927965000	-0.494768000	4.754354000
1	-3.078183000	-2.063718000	3.914801000
1	-2.452740000	1.636732000	1.661141000
1	-2.648508000	1.669617000	3.439090000
1	-4.044958000	1.305132000	2.389994000
1	-0.051322000	2.472508000	-1.123535000
6	-1.690797000	1.386831000	-2.152736000
1	-1.578600000	1.507778000	-3.250592000
1	-2.120423000	0.382476000	-1.997015000
6	-2.665660000	2.439977000	-1.654827000
6	-3.867207000	2.079850000	-1.014203000
6	-2.399851000	3.811520000	-1.859393000
6	-4.777490000	3.061445000	-0.587161000
1	-4.089314000	1.019669000	-0.848115000
6	-3.303514000	4.794109000	-1.429251000
1	-1.477898000	4.111756000	-2.372313000
6	-4.498531000	4.421382000	-0.790769000
1	-5.707460000	2.759955000	-0.092734000
1	-3.078874000	5.852645000	-1.600109000
1	-5.207622000	5.186941000	-0.458214000
1	-1.524656000	-3.922335000	1.479735000
1	-0.150567000	-4.265663000	2.576012000
1	-1.409503000	-3.093438000	3.038669000
1	3.810070000	-0.641004000	2.878149000
1	3.826004000	-2.406201000	2.639866000
1	4.526669000	-1.310057000	1.407959000

TS(XIII-2i) (V)
SCF (BP86/SDD/6-31G**) Energy 333 K = -1712.86067053
Thermal correction to Gibbs Free Energy= 0.679115
Lowest Frequency = -803.5258 cm^{-1}
Second Frequency = 8.9255 cm^{-1}
SCF (B3PW91-D3,C6H6/tzvp) Energy 333 K= -2852.62311440

1	-0.169140000	0.706693000	-1.432114000
6	-0.477626000	1.874953000	-0.374268000
26	0.199603000	-0.164579000	-0.080537000
6	-0.382976000	1.508635000	1.025021000
1	-1.315989000	1.333872000	1.575395000
1	0.436168000	1.916198000	1.627100000
7	-0.726882000	-1.936619000	0.015589000
7	2.068454000	-0.847429000	0.009061000
1	0.337253000	2.511845000	-0.751730000
6	-1.842390000	2.246588000	-0.981045000
1	-1.790098000	2.180855000	-2.083508000
1	-2.595586000	1.508537000	-0.651366000
6	-2.270887000	3.646821000	-0.570696000

6	-0.046566000	-3.093911000	-0.068279000
6	-2.161091000	-1.893098000	0.065132000
6	2.349915000	-2.160697000	-0.098648000
6	3.101136000	0.149096000	0.070753000
6	1.367630000	-3.179579000	-0.127331000
6	-0.802672000	-4.412429000	-0.133687000
6	-2.925983000	-1.995379000	-1.134870000
6	-2.797452000	-1.654393000	1.322801000
1	1.762070000	-4.196234000	-0.201848000
1	-1.025217000	-4.671523000	-1.184380000
1	-0.197977000	-5.229948000	0.288142000
1	-1.763946000	-4.359641000	0.399130000
6	3.797790000	-2.609600000	-0.221090000
1	3.914473000	-3.654303000	0.104729000
1	4.125133000	-2.547991000	-1.274555000
1	4.475016000	-1.969008000	0.364110000
6	3.461102000	0.685583000	1.345821000
6	3.691744000	0.649787000	-1.126968000
6	-4.325957000	-1.866290000	-1.046092000
6	-2.282602000	-2.224353000	-2.504988000
6	-4.199844000	-1.540770000	1.349850000
6	-1.980713000	-1.559748000	2.613958000
1	-4.925839000	-1.940326000	-1.960183000
6	-4.963663000	-1.644179000	0.179413000
1	-1.203653000	-2.394733000	-2.348461000
6	-2.416924000	-0.974819000	-3.405097000
6	-2.865694000	-3.466147000	-3.218563000
1	-4.704020000	-1.364179000	2.305218000
1	-0.987950000	-1.158276000	2.334035000
6	-1.760483000	-2.955600000	3.244948000
6	-2.589812000	-0.601795000	3.657822000
1	-6.053813000	-1.548858000	0.223992000
1	-1.949302000	-1.156027000	-4.389172000
1	-1.918137000	-0.105822000	-2.944889000
1	-3.477102000	-0.716633000	-3.576272000
1	-2.803082000	-4.371021000	-2.590517000
1	-2.319120000	-3.661057000	-4.157720000
1	-3.928278000	-3.320288000	-3.481186000

1	-2.726619000	-3.425027000	3.503453000
1	-1.164990000	-2.871784000	4.171288000
1	-1.224757000	-3.634533000	2.562633000
1	-2.809693000	0.389403000	3.227092000
1	-1.883841000	-0.460213000	4.493897000
1	-3.525629000	-0.998746000	4.090481000
6	4.442186000	1.693316000	1.391950000
6	2.817780000	0.165635000	2.634004000
6	4.661560000	1.665776000	-1.018878000
6	3.296165000	0.139394000	-2.514762000
1	4.736346000	2.109467000	2.360719000
6	5.043409000	2.181791000	0.224230000
1	1.812778000	-0.205313000	2.358214000
6	3.612013000	-1.022955000	3.226644000
6	2.631662000	1.259324000	3.706221000
1	5.123898000	2.059433000	-1.931149000
1	2.598955000	-0.705437000	-2.378564000
6	4.515562000	-0.368884000	-3.318026000
6	2.541764000	1.226825000	-3.314534000
1	5.802501000	2.968976000	0.284585000
1	3.665893000	-1.871410000	2.526038000
1	3.133083000	-1.383893000	4.154079000
1	4.645067000	-0.719443000	3.474339000
1	3.595082000	1.591105000	4.133130000
1	2.028213000	0.866150000	4.542275000
1	2.117290000	2.147349000	3.302383000
1	5.213308000	0.452727000	-3.558026000
1	4.187959000	-0.812276000	-4.274468000
1	5.085944000	-1.133293000	-2.763411000
1	1.619122000	1.530209000	-2.792319000
1	2.258197000	0.846114000	-4.311704000
1	3.169741000	2.123751000	-3.460043000
6	-1.794416000	4.772509000	-1.274376000
6	-3.126929000	3.858508000	0.528933000
6	-2.157854000	6.072075000	-0.889450000
1	-1.134818000	4.625309000	-2.138389000
6	-3.494265000	5.156375000	0.917524000
1	-3.514115000	2.993680000	1.079741000

6	-3.009567000	6.268246000	0.210034000
1	-1.780270000	6.932744000	-1.452304000
1	-4.163820000	5.299257000	1.772777000
1	-3.297667000	7.281300000	0.510252000

TS(XIII-2i) (III)
SCF (BP86/SDD/6-31G**) Energy 333 K = -1712.88396875
Thermal correction to Gibbs Free Energy= 0.684556
Lowest Frequency = -320.3455 cm^{-1}
Second Frequency = 12.3383 cm^{-1}
SCF (B3PW91-D3,C6H6/tzvp) Energy 333 K= -2852.63387088

1	-0.887633000	0.971949000	-0.050583000
6	-0.280759000	1.504246000	-1.583142000
6	0.869147000	0.762659000	-1.991993000
1	1.861184000	1.221121000	-1.936065000
1	0.761366000	-0.027881000	-2.748136000
26	0.321276000	0.034599000	-0.180117000
7	2.097002000	-0.355955000	0.551545000
6	2.341256000	-1.272585000	1.521570000
6	1.358215000	-2.145923000	2.026983000
6	3.718463000	-1.356353000	2.164497000
6	-0.006570000	-2.216309000	1.686102000
1	1.695574000	-2.853104000	2.788631000
7	-0.581733000	-1.378311000	0.792668000
6	-0.842362000	-3.271172000	2.393375000
6	3.220134000	0.435357000	0.103755000
6	3.514426000	1.669102000	0.751395000
6	4.006095000	-0.012948000	-1.000335000
6	4.608537000	2.427361000	0.288667000
6	2.672910000	2.201695000	1.913965000
1	4.841785000	3.379587000	0.778577000
6	5.403430000	1.988026000	-0.775708000
1	6.254993000	2.587977000	-1.113939000
6	5.095924000	0.778220000	-1.411565000
1	5.712769000	0.441351000	-2.251914000
6	3.689009000	-1.315145000	-1.742932000
1	1.947152000	1.416360000	2.187525000
6	1.862512000	3.447326000	1.487380000
6	3.526246000	2.519308000	3.162762000
1	1.239972000	3.812625000	2.323116000
1	1.193591000	3.213674000	0.642443000
1	2.529531000	4.270846000	1.175839000
1	4.120889000	1.648515000	3.487489000
1	2.878020000	2.821588000	4.003725000
1	4.230551000	3.348937000	2.974836000
1	2.662630000	-1.605987000	-1.456985000
6	4.638604000	-2.465263000	-1.330501000
6	3.719173000	-1.143139000	-3.278580000
1	5.689896000	-2.204238000	-1.547727000
1	4.396865000	-3.385479000	-1.891413000
1	4.562707000	-2.699181000	-0.256740000
1	3.084894000	-0.304777000	-3.609447000
1	3.357461000	-2.063396000	-3.769774000
1	4.742204000	-0.958707000	-3.651613000
6	-1.989670000	-1.538694000	0.515497000
6	-2.388872000	-2.312886000	-0.614412000
6	-2.961056000	-0.920445000	1.356599000
6	-3.766173000	-2.448244000	-0.881978000
6	-1.375009000	-3.037598000	-1.505797000
1	-4.086118000	-3.047778000	-1.741801000
6	-4.730892000	-1.844790000	-0.065807000
1	-5.796363000	-1.967097000	-0.288270000
6	-4.322514000	-1.087882000	1.040675000
1	-5.078235000	-0.616303000	1.678894000
6	-2.568919000	-0.079932000	2.574856000
1	-0.369240000	-2.695671000	-1.203550000
6	-1.438004000	-4.569758000	-1.299117000
6	-1.551973000	-2.699651000	-3.002384000
1	-1.249070000	-4.850292000	-0.249827000
1	-0.682297000	-5.076625000	-1.924877000
1	-2.429498000	-4.968067000	-1.579292000
1	-2.533765000	-3.029579000	-3.385441000
1	-0.777295000	-3.207414000	-3.603394000
1	-1.467783000	-1.615837000	-3.186493000
1	-1.478932000	-0.183499000	2.712892000
6	-3.262056000	-0.564072000	3.869327000
6	-2.864680000	1.419371000	2.343142000
1	-4.354829000	-0.410462000	3.826106000
1	-2.885434000	0.002096000	4.739012000

1	-3.087375000	-1.636832000	4.059867000
1	-2.337684000	1.798038000	1.452539000
1	-2.544140000	2.014456000	3.216789000
1	-3.944067000	1.594209000	2.190913000
1	-0.088050000	2.507099000	-1.174330000
6	-1.613928000	1.417111000	-2.343493000
1	-1.395978000	1.720081000	-3.387947000
1	-1.962083000	0.371496000	-2.388634000
6	-2.720100000	2.305628000	-1.803069000
6	-3.915785000	1.758668000	-1.298573000
6	-2.581242000	3.710570000	-1.826616000
6	-4.945112000	2.591641000	-0.828827000
1	-4.040441000	0.670566000	-1.270992000
6	-3.604137000	4.544669000	-1.352081000
1	-1.665274000	4.156212000	-2.233986000
6	-4.792739000	3.986355000	-0.851825000
1	-5.868590000	2.145778000	-0.443691000
1	-3.477797000	5.632417000	-1.381721000
1	-5.595223000	4.636135000	-0.486748000
1	-1.513553000	-3.799257000	1.697884000
1	-0.193152000	-4.008577000	2.889007000
1	-1.486442000	-2.813261000	3.164629000
1	3.879415000	-0.497226000	2.840431000
1	3.810746000	-2.275941000	2.761827000
1	4.532908000	-1.327487000	1.425032000

2i (V)
SCF (BP86/SDD/6-31G**) Energy 333 K = -1712.91047314
Thermal correction to Gibbs Free Energy= 0.680497
Lowest Frequency = 7.4851 cm^{-1}
Second Frequency = 14.0539 cm^{-1}
SCF (B3PW91-D3,C6H6/tzvp) Energy 333 K= -2852.67371501

1	1.668881000	0.266744000	-2.703629000
6	1.812946000	1.043423000	-1.927977000
26	-0.303565000	-0.257363000	-0.139178000
6	0.500631000	1.275316000	-1.163772000
1	-0.284825000	1.655392000	-1.852210000
1	0.641692000	2.076361000	-0.405235000
7	-2.158709000	-0.499321000	0.521202000
7	0.379108000	-1.925046000	0.680095000
1	2.590523000	0.648454000	-1.246302000
6	2.384528000	2.307622000	-2.642955000
1	3.241929000	1.998426000	-3.271340000
1	1.612330000	2.704142000	-3.328828000
6	2.831690000	3.400882000	-1.693180000
6	-2.585421000	-1.539764000	1.272169000
6	-3.067070000	0.547811000	0.136775000
6	-0.357252000	-2.781739000	1.425879000
6	1.766672000	-2.193102000	0.412584000
6	-1.732380000	-2.587898000	1.690164000
6	-4.043121000	-1.603632000	1.695817000
6	-3.732039000	0.473190000	-1.122168000
6	-3.228356000	1.687623000	0.978366000
1	-2.204956000	-3.360412000	2.301859000
1	-4.711724000	-1.592763000	0.817756000
1	-4.245811000	-2.514255000	2.278656000
1	-4.319344000	-0.727972000	2.308192000
6	0.305615000	-4.018698000	2.008382000
1	-0.389813000	-4.569810000	2.658559000
1	0.648175000	-4.700596000	1.210424000
1	1.200709000	-3.750988000	2.595184000
6	2.768886000	-1.585288000	1.223779000
6	2.126158000	-2.996620000	-0.710036000
6	-4.544329000	1.552733000	-1.517851000
6	-3.572541000	-0.732585000	-2.050468000
6	-4.052341000	2.738606000	0.533155000
6	-2.519103000	1.804522000	2.329196000
1	-5.057627000	1.507751000	-2.485158000
6	-4.707102000	2.680081000	-0.703382000
1	-2.980390000	-1.491972000	-1.509982000
6	-2.787888000	-0.352487000	-3.327496000
6	-4.930914000	-1.372478000	-2.415408000
1	-4.180238000	3.621783000	1.169334000
1	-2.014186000	0.842317000	2.525013000
6	-3.508205000	2.059075000	3.489200000
6	-1.431221000	2.902678000	2.294164000
1	-5.342397000	3.509908000	-1.030734000
1	-2.644708000	-1.234988000	-3.975712000
1	-1.794327000	0.057814000	-3.078126000

1	-3.327535000	0.412731000	-3.913167000
1	-5.499133000	-1.663878000	-1.515607000
1	-4.778095000	-2.277171000	-3.029523000
1	-5.563403000	-0.679946000	-2.998224000
1	-4.015553000	3.034198000	3.383994000
1	-2.974008000	2.067147000	4.455324000
1	-4.290197000	1.282203000	3.539539000
1	-0.688332000	2.706942000	1.502853000
1	-0.899521000	2.956368000	3.260571000
1	-1.874412000	3.894937000	2.097446000
6	4.120433000	-1.784299000	0.881895000
6	2.417183000	-0.732172000	2.444027000
6	3.491910000	-3.159090000	-1.008192000
6	1.070418000	-3.654879000	-1.601567000
1	4.899464000	-1.320437000	1.497636000
6	4.486626000	-2.559291000	-0.225107000
1	1.317008000	-0.731579000	2.541484000
6	3.001060000	-1.325287000	3.746906000
6	2.872818000	0.733157000	2.261106000
1	3.781366000	-3.767288000	-1.872502000
1	0.095700000	-3.558608000	-1.092371000
6	1.335622000	-5.161145000	-1.819776000
6	0.954586000	-2.922203000	-2.958183000
1	5.543657000	-2.698817000	-0.475408000
1	2.663890000	-2.363011000	3.911074000
1	2.688380000	-0.724167000	4.618665000
1	4.105193000	-1.333352000	3.726952000
1	3.971143000	0.802955000	2.170754000
1	2.569766000	1.345533000	3.128523000
1	2.436362000	1.182538000	1.353646000
1	2.262832000	-5.335289000	-2.393416000
1	0.507191000	-5.617551000	-2.389011000
1	1.430824000	-5.702283000	-0.862858000
1	0.696535000	-1.858497000	-2.816463000
1	0.174219000	-3.384412000	-3.588370000
1	1.908500000	-2.963250000	-3.513270000
6	4.097100000	3.336107000	-1.070606000
6	1.994970000	4.493152000	-1.382299000
6	4.513158000	4.326815000	-0.168419000
1	4.765562000	2.498653000	-1.305032000
6	2.405896000	5.487571000	-0.479454000
1	1.012221000	4.565260000	-1.862523000
6	3.667286000	5.407948000	0.131369000
1	5.502913000	4.259119000	0.296671000
1	1.740064000	6.328764000	-0.257043000
1	3.991945000	6.184934000	0.831678000

2i (III)
SCF (BP86/SDD/6-31G**) Energy 333 K = -1712.89551199
Thermal correction to Gibbs Free Energy= 0.685598
Lowest Frequency = 9.4226 cm^{-1}
Second Frequency = 12.9805 cm^{-1}
SCF (B3PW91-D3,C6H6/tzvp) Energy 333 K= -2852.65124195

1	-1.138702000	0.956373000	-0.311202000
6	-0.712371000	1.818083000	-0.986450000
6	0.679792000	1.481576000	-1.444500000
1	1.445814000	2.241266000	-1.236367000
1	0.761065000	1.100327000	-2.475282000
26	0.484827000	0.085868000	-0.067997000
7	2.243175000	-0.446184000	0.340428000
6	2.576401000	-1.594467000	0.985204000
6	1.631591000	-2.555267000	1.395553000
6	4.028407000	-1.849945000	1.361398000
6	0.230869000	-2.528074000	1.240557000
1	2.038476000	-3.432441000	1.904681000
7	-0.417369000	-1.495507000	0.646206000
6	-0.548821000	-3.726900000	1.757763000
6	3.309770000	0.459328000	-0.025356000
6	3.740462000	1.445301000	0.906314000
6	3.900389000	0.368705000	-1.319846000
6	4.778455000	2.319287000	0.528437000
6	3.076629000	1.605483000	2.275943000
1	5.115971000	3.085688000	1.235232000
6	5.388796000	2.223972000	-0.727620000
1	6.202191000	2.905312000	-0.999248000
6	4.945179000	1.257183000	-1.639118000
1	5.416461000	1.194005000	-2.625884000
6	3.423323000	-0.654216000	-2.354628000
1	2.435511000	0.722153000	2.440814000

6	2.158362000	2.850121000	2.291188000
6	4.096132000	1.669312000	3.434126000
1	1.646273000	2.949671000	3.264685000
1	1.388402000	2.782787000	1.503025000
1	2.739190000	3.773081000	2.115692000
1	4.769296000	0.795047000	3.434499000
1	3.571849000	1.696355000	4.405290000
1	4.726791000	2.573991000	3.377029000
1	2.420181000	-0.987586000	-2.033137000
6	4.340307000	-1.899338000	-2.409499000
6	3.284783000	-0.046778000	-3.768318000
1	5.375254000	-1.614549000	-2.671198000
1	3.981776000	-2.609480000	-3.175786000
1	4.368442000	-2.433783000	-1.446659000
1	2.679433000	0.874457000	-3.758759000
1	2.800249000	-0.771392000	-4.445751000
1	4.266833000	0.200414000	-4.209450000
6	-1.852369000	-1.585066000	0.539824000
6	-2.436469000	-2.298772000	-0.548912000
6	-2.674070000	-0.952726000	1.518530000
6	-3.841143000	-2.371524000	-0.627582000
6	-1.576128000	-2.963146000	-1.627636000
1	-4.302751000	-2.916270000	-1.458495000
6	-4.658747000	-1.777177000	0.343335000
1	-5.748448000	-1.863406000	0.273475000
6	-4.072327000	-1.071315000	1.401252000
1	-4.712130000	-0.601497000	2.156628000
6	-2.076747000	-0.169736000	2.690025000
1	-0.546636000	-3.030494000	-1.236199000
6	-2.042081000	-4.394462000	-1.972852000
6	-1.518437000	-2.090227000	-2.902692000
1	-2.121019000	-5.028095000	-1.072874000
1	-1.325689000	-4.871321000	-2.664005000
1	-3.027461000	-4.399658000	-2.471300000
1	-2.528067000	-1.933600000	-3.322804000
1	-0.897228000	-2.570891000	-3.679299000
1	-1.079859000	-1.102047000	-2.681045000
1	-0.987862000	-0.100706000	2.513619000
6	-2.288392000	-0.901413000	4.035715000
6	-2.634025000	1.269455000	2.766116000
1	-3.363677000	-1.017793000	4.260416000
1	-1.831987000	-0.330338000	4.863479000
1	-1.835873000	-1.907004000	4.029913000
1	-2.500540000	1.809011000	1.813948000
1	-2.121385000	1.837733000	3.562012000
1	-3.713388000	1.276549000	2.998714000
1	-0.720055000	2.677885000	-0.291628000
6	-1.796072000	2.017498000	-2.084961000
1	-1.392042000	2.766482000	-2.789680000
1	-1.908771000	1.075357000	-2.650084000
6	-3.137339000	2.487566000	-1.558437000
6	-4.132071000	1.567246000	-1.170226000
6	-3.406026000	3.866157000	-1.421406000
6	-5.361712000	2.013574000	-0.660551000
1	-3.944771000	0.491875000	-1.268587000
6	-4.634225000	4.315112000	-0.912617000
1	-2.644606000	4.594272000	-1.727268000
6	-5.617953000	3.387955000	-0.530838000
1	-6.121309000	1.281396000	-0.366877000
1	-4.825980000	5.389726000	-0.820806000
1	-6.579512000	3.735522000	-0.138298000
1	-0.956597000	-4.328249000	0.925561000
1	0.100313000	-4.379567000	2.360672000
1	-1.411482000	-3.421017000	2.372085000
1	4.329598000	-1.206378000	2.207996000
1	4.166228000	-2.897557000	1.669009000
1	4.723346000	-1.626567000	0.537582000

XIV (V)
SCF (BP86/SDD/6-31G**) Energy 333 K = -1712.87775723
Thermal correction to Gibbs Free Energy= 0.681543
Lowest Frequency = 14.4948 cm^{-1}
Second Frequency = 16.9705 cm^{-1}
SCF (B3PW91-D3,C6H6/tzvp) Energy 333 K= -2852.64482677

1	-0.413688000	-0.671426000	-2.368147000
6	-0.347114000	1.883382000	-0.756891000
26	-0.442190000	-0.258969000	-0.779030000
6	0.920532000	1.452126000	-0.373253000
1	-0.545359000	2.167392000	-1.798148000

1	-1.101001000	2.191814000	-0.025848000
1	1.152306000	1.385364000	0.697174000
6	2.118762000	1.429573000	-1.309198000
7	-2.241048000	-0.568343000	0.115339000
7	0.472632000	-1.729258000	0.298094000
1	2.698279000	0.500506000	-1.169179000
1	1.760959000	1.440015000	-2.355060000
6	3.029831000	2.628163000	-1.052942000
6	-2.581600000	-1.790587000	0.572152000
6	-3.224930000	0.478993000	0.065871000
6	-1.645575000	-2.824351000	0.802009000
6	-4.041648000	-2.108536000	0.853083000
6	-0.231741000	-2.797735000	0.720828000
1	-2.071670000	-3.771045000	1.143312000
6	0.484004000	-4.080071000	1.120816000
6	1.909243000	-1.746983000	0.353127000
6	-3.971682000	0.707353000	-1.126937000
6	-3.389532000	1.329608000	1.201523000
6	-4.876569000	1.787037000	-1.153124000
6	-3.827876000	-0.175799000	-2.368839000
6	-4.313329000	2.389119000	1.120056000
6	-2.609261000	1.111496000	2.502179000
1	-5.455962000	1.971121000	-2.064999000
6	-5.053555000	2.623894000	-0.045310000
1	-3.111730000	-0.981367000	-2.129592000
6	-3.236662000	0.613052000	-3.559000000
6	-5.172274000	-0.826542000	-2.769819000
1	-5.764917000	3.455419000	-0.089181000
1	-4.453159000	3.042774000	1.988642000
1	-1.877024000	0.305992000	2.317665000
6	-3.538501000	0.651733000	3.650501000
6	-1.823318000	2.371058000	2.933107000
1	-3.143499000	-0.040471000	-4.443929000
1	-2.229972000	0.991168000	-3.317695000
1	-3.878180000	1.469290000	-3.834300000
1	-5.620965000	-1.395026000	-1.937176000
1	-5.025284000	-1.520102000	-3.615941000
1	-5.910797000	-0.068826000	-3.086722000

1	-4.297051000	1.421113000	3.879677000
1	-2.956969000	0.468479000	4.571171000
1	-4.072930000	-0.279128000	3.398488000
1	-1.121021000	2.706486000	2.151787000
1	-1.239457000	2.164210000	3.847085000
1	-2.498690000	3.215118000	3.158048000
6	2.559113000	-1.157113000	1.480791000
6	2.670939000	-2.288656000	-0.725184000
6	3.966351000	-1.136399000	1.510836000
6	1.758245000	-0.587777000	2.655484000
6	4.077433000	-2.228846000	-0.644975000
6	2.028072000	-2.938898000	-1.953704000
1	4.476312000	-0.698281000	2.375159000
6	4.727029000	-1.664840000	0.458364000
1	0.751696000	-0.342292000	2.268871000
6	1.575261000	-1.637879000	3.777961000
6	2.374027000	0.701062000	3.242735000
1	5.821083000	-1.637872000	0.500907000
1	4.672743000	-2.640101000	-1.467931000
1	0.933740000	-2.927214000	-1.810150000
6	2.482805000	-4.407747000	-2.125357000
6	2.320899000	-2.137881000	-3.242465000
1	1.039076000	-2.531416000	3.421088000
1	0.998236000	-1.211325000	4.617479000
1	2.555057000	-1.963367000	4.170642000
1	3.315486000	0.496734000	3.782872000
1	1.679140000	1.155011000	3.969965000
1	2.594319000	1.450702000	2.463917000
1	3.559998000	-4.471727000	-2.360891000
1	1.934621000	-4.883216000	-2.957227000
1	2.309731000	-5.005728000	-1.214701000
1	1.899634000	-1.122100000	-3.176754000
1	1.860227000	-2.635854000	-4.113694000
1	3.406553000	-2.060248000	-3.431910000
6	2.679589000	3.912592000	-1.517824000
6	4.226677000	2.477120000	-0.324496000
6	3.506825000	5.017082000	-1.266700000
1	1.749977000	4.045014000	-2.083738000

6	5.055537000	3.582170000	-0.069179000
1	4.509222000	1.481275000	0.036966000
6	4.698567000	4.855467000	-0.539647000
1	3.222482000	6.006347000	-1.641598000
1	5.984890000	3.445765000	0.494669000
1	5.345498000	5.717238000	-0.344282000
1	0.654503000	-4.708385000	0.228116000
1	1.468930000	-3.882115000	1.569187000
1	-0.124060000	-4.664837000	1.827853000
1	-4.144405000	-3.066231000	1.384153000
1	-4.521427000	-1.315484000	1.448413000
1	-4.607082000	-2.175377000	-0.093220000

XIV (III)
SCF (BP86/SDD/6-31G**) Energy 333 K = -1712.88193273
Thermal correction to Gibbs Free Energy= 0.684636
Lowest Frequency = 9.2331 cm^{-1}
Second Frequency = 11.5384 cm^{-1}
SCF (B3PW91-D3,C6H6/tzvp) Energy 333 K= -2852.62602601

1	-1.067356000	-0.465853000	-1.735224000
6	0.325025000	0.971859000	-1.944185000
26	-0.311195000	-0.315701000	-0.449984000
1	0.748107000	0.385018000	-2.767320000
6	1.079438000	1.219994000	-0.773028000
1	-0.462889000	1.672129000	-2.248384000
7	0.791985000	-1.713842000	0.422586000
7	-1.932027000	-0.690112000	0.512767000
1	2.086695000	0.788709000	-0.695991000
6	0.833665000	2.482380000	0.051231000
1	-0.237551000	2.745424000	-0.005521000
1	1.060167000	2.304611000	1.118279000
6	1.664570000	3.658973000	-0.455797000
6	0.250632000	-2.686127000	1.196795000
6	2.193204000	-1.794295000	0.089087000
6	-2.135753000	-1.791706000	1.272336000
6	-3.020495000	0.226908000	0.270158000
6	-1.109025000	-2.707866000	1.577409000
6	1.105875000	-3.835667000	1.711030000
6	3.192110000	-1.386925000	1.023322000
6	2.564511000	-2.249392000	-1.214444000
6	-3.526446000	-2.116755000	1.796368000
6	-3.943295000	0.005578000	-0.793848000
6	-3.133406000	1.381234000	1.106725000
1	-1.409549000	-3.559653000	2.193553000
6	4.542901000	-1.426846000	0.624466000
6	2.859677000	-0.905752000	2.439117000
6	3.931258000	-2.280477000	-1.551588000
6	1.522054000	-2.748295000	-2.219476000
6	-4.969232000	0.949046000	-0.996925000
6	-3.862444000	-1.207237000	-1.724120000
6	-4.188300000	2.281868000	0.863928000
6	-2.154385000	1.638286000	2.257403000
1	5.315841000	-1.108508000	1.333283000
6	4.918643000	-1.868106000	-0.648572000
1	1.773781000	-1.028349000	2.592912000
6	3.187904000	0.593322000	2.619513000
6	3.597339000	-1.728177000	3.522501000
1	4.226918000	-2.633712000	-2.544885000
1	0.557887000	-2.279010000	-1.944394000
6	1.340872000	-4.281795000	-2.120366000
6	1.835635000	-2.345500000	-3.675474000
1	5.975151000	-1.893280000	-0.935757000
1	2.911942000	0.935458000	3.632615000
1	2.642783000	1.210354000	1.888967000
1	4.267628000	0.782466000	2.483359000
1	3.428410000	-2.811880000	3.410595000
1	3.257714000	-1.429635000	4.529681000
1	4.687912000	-1.560200000	3.479890000
1	2.287389000	-4.804830000	-2.347550000
1	0.579294000	-4.629884000	-2.840666000
1	1.016884000	-4.590128000	-1.113194000
1	2.036305000	-1.264689000	-3.768939000
1	0.980118000	-2.592905000	-4.327377000
1	2.712985000	-2.883529000	-4.077024000
1	-5.682435000	0.790279000	-1.813755000
6	-5.101786000	2.074798000	-0.177248000
1	-3.027773000	-1.841202000	-1.379263000
6	-5.157524000	-2.052141000	-1.689179000
6	-3.551366000	-0.778478000	-3.176967000

1	-4.291544000	3.167416000	1.498776000
1	-1.193946000	1.172783000	1.966121000
6	-2.614453000	0.962312000	3.572052000
6	-1.898019000	3.137899000	2.517104000
1	-5.912027000	2.791300000	-0.349620000
1	-5.420219000	-2.366168000	-0.665076000
1	-5.040393000	-2.960301000	-2.306036000
1	-6.017753000	-1.488063000	-2.091043000
1	-4.355007000	-0.140866000	-3.586181000
1	-3.458662000	-1.663868000	-3.830499000
1	-2.606576000	-0.212407000	-3.230150000
1	-3.608190000	1.336960000	3.876410000
1	-1.904236000	1.186137000	4.387807000
1	-2.676195000	-0.132281000	3.474237000
1	-1.644492000	3.683761000	1.593517000
1	-1.060702000	3.258737000	3.225600000
1	-2.775256000	3.632481000	2.971099000
6	1.104688000	4.607865000	-1.335207000
6	3.016974000	3.806388000	-0.084522000
6	1.871893000	5.674096000	-1.830357000
1	0.052543000	4.512053000	-1.628785000
6	3.787726000	4.870383000	-0.577356000
1	3.469781000	3.080010000	0.600392000
6	3.217600000	5.808733000	-1.453322000
1	1.415550000	6.403527000	-2.508463000
1	4.835548000	4.969238000	-0.273251000
1	3.817323000	6.641082000	-1.836459000
1	-4.099873000	-2.677920000	1.036769000
1	-3.463083000	-2.747806000	2.696485000
1	-4.105632000	-1.212147000	2.034058000
1	1.594982000	-3.569032000	2.664329000
1	0.480041000	-4.721784000	1.900057000
1	1.903435000	-4.106436000	1.002737000

TS(XIV-2j) (V)
SCF (BP86/SDD/6-31G**) Energy 333 K = -1712.85737217
Thermal correction to Gibbs Free Energy= 0.678121
Lowest Frequency = -820.2820 cm^{-1}
Second Frequency = 6.8693 cm^{-1}
SCF (B3PW91-D3,C6H6/tzvp) Energy 333 K= -2852.62114189

1	-0.439397000	0.381726000	-2.096167000
6	0.136670000	1.757020000	-1.456750000
26	-0.336070000	-0.119143000	-0.536932000
6	0.504829000	1.746794000	-0.052578000
1	0.941997000	1.805142000	-2.202042000
1	-0.746862000	2.331230000	-1.764772000
1	-0.187645000	2.256691000	0.632444000
6	1.972295000	1.763214000	0.346183000
7	-2.184712000	-0.634881000	0.042026000
7	0.503548000	-1.903723000	-0.221359000
1	2.071073000	1.479899000	1.412353000
1	2.526204000	0.975444000	-0.202936000
6	2.723971000	3.085477000	0.140992000
6	-2.564563000	-1.923425000	0.080066000
6	-3.107013000	0.449204000	0.228392000
6	-1.666281000	-3.009972000	-0.077256000
6	-4.026593000	-2.281968000	0.296598000
6	-0.253196000	-3.018144000	-0.159397000
1	-2.132130000	-3.998669000	-0.050967000
6	0.419009000	-4.381818000	-0.109889000
6	1.932628000	-1.970144000	-0.100068000
6	-3.943564000	0.883651000	-0.843106000
6	-3.095220000	1.145409000	1.476465000
6	-4.771205000	2.003763000	-0.628704000
6	-3.971655000	0.191680000	-2.209124000
6	-3.947429000	2.253439000	1.633512000
6	-2.207028000	0.674095000	2.629448000
1	-5.419383000	2.348208000	-1.442433000
6	-4.781831000	2.684494000	0.593427000
1	-3.314742000	-0.693551000	-2.156438000
6	-3.408833000	1.106980000	-3.320914000
6	-5.395353000	-0.282882000	-2.584752000
1	-5.435395000	3.551704000	0.735672000
1	-3.954994000	2.792036000	2.586420000
1	-1.332450000	0.172606000	2.173179000
6	-2.940309000	-0.370934000	3.504055000
6	-1.676286000	1.825074000	3.507642000
1	-3.441948000	0.591352000	-4.296914000
1	-2.360430000	1.378165000	-3.117526000

1	-3.997174000	2.037382000	-3.410566000
1	-5.840730000	-0.918638000	-1.801193000
1	-5.372514000	-0.860815000	-3.525087000
1	-6.076499000	0.572233000	-2.740543000
1	-3.847289000	0.066993000	3.957992000
1	-2.286175000	-0.721230000	4.322019000
1	-3.247303000	-1.251751000	2.917048000
1	-1.190420000	2.609840000	2.904038000
1	-0.932586000	1.437710000	4.224788000
1	-2.478295000	2.300284000	4.100322000
6	2.529525000	-2.130978000	1.186583000
6	2.741462000	-1.791388000	-1.263175000
6	3.933941000	-2.112055000	1.278781000
6	1.696138000	-2.284480000	2.462058000
6	4.141674000	-1.790554000	-1.110879000
6	2.124532000	-1.645710000	-2.656423000
1	4.405137000	-2.226510000	2.261330000
6	4.739705000	-1.946414000	0.145573000
1	0.647338000	-2.455175000	2.165762000
6	2.144282000	-3.490377000	3.318431000
6	1.722346000	-0.986109000	3.301240000
1	5.830650000	-1.938728000	0.241405000
1	4.774168000	-1.664017000	-1.995809000
1	1.067702000	-1.342557000	-2.523096000
6	2.124441000	-2.995346000	-3.412915000
6	2.810752000	-0.556659000	-3.508228000
1	2.159680000	-4.426734000	2.734714000
1	1.458076000	-3.629782000	4.171757000
1	3.156649000	-3.343493000	3.733890000
1	2.752580000	-0.730690000	3.606474000
1	1.116700000	-1.103046000	4.217461000
1	1.316660000	-0.137185000	2.726388000
1	3.155187000	-3.366915000	-3.554793000
1	1.663998000	-2.880555000	-4.410070000
1	1.558708000	-3.769505000	-2.868863000
1	2.869590000	0.404434000	-2.970722000
1	2.243485000	-0.394079000	-4.440820000
1	3.837518000	-0.841990000	-3.798632000

6	2.057185000	4.308741000	-0.059836000
6	4.135032000	3.094151000	0.178777000
6	2.775494000	5.506688000	-0.213034000
1	0.962726000	4.316352000	-0.103027000
6	4.856957000	4.286715000	0.025874000
1	4.670525000	2.148066000	0.328283000
6	4.178039000	5.501561000	-0.170489000
1	2.235114000	6.447234000	-0.369130000
1	5.952295000	4.268304000	0.054977000
1	4.738892000	6.434317000	-0.293391000
1	1.344207000	-4.401409000	-0.705931000
1	0.697546000	-4.640307000	0.927352000
1	-0.264848000	-5.163372000	-0.474615000
1	-4.118589000	-3.252862000	0.807355000
1	-4.550717000	-1.512344000	0.883086000
1	-4.547202000	-2.366279000	-0.673957000

TS(XIV-2j) (III)
SCF (BP86/SDD/6-31G**) Energy 333 K = -1712.88121871
Thermal correction to Gibbs Free Energy= 0.683677
Lowest Frequency = -295.5897 cm^{-1}
Second Frequency = 10.2546 cm^{-1}
SCF (B3PW91-D3,C6H6/tzvp) Energy 333 K= -2852.62663272

1	-1.184218000	0.283004000	-1.790167000
6	0.135947000	1.425277000	-1.775861000
26	-0.429793000	-0.161214000	-0.563314000
1	0.293067000	1.170618000	-2.831265000
6	1.154694000	1.141164000	-0.816344000
1	-0.510483000	2.302434000	-1.631575000
7	0.510828000	-1.772284000	0.098943000
7	-2.135802000	-0.602949000	0.242097000
1	2.024483000	0.567975000	-1.163530000
6	1.460264000	2.089780000	0.339264000
1	0.529214000	2.570053000	0.690453000
1	1.872872000	1.516783000	1.187519000
6	2.466928000	3.163247000	-0.063617000
6	-0.163289000	-2.865293000	0.537657000
6	1.950389000	-1.839346000	0.035836000
6	-2.474894000	-1.820578000	0.714679000
6	-3.114435000	0.457482000	0.215199000
6	-1.549523000	-2.881882000	0.796510000

6	0.572465000	-4.173940000	0.794726000
6	2.732154000	-1.737760000	1.226357000
6	2.585944000	-1.950279000	-1.238999000
6	-3.902351000	-2.108565000	1.152284000
6	-4.037415000	0.571953000	-0.865830000
6	-3.109471000	1.416710000	1.273037000
1	-1.952600000	-3.835844000	1.147129000
6	4.136238000	-1.697772000	1.104065000
6	2.120407000	-1.706341000	2.633382000
6	3.992115000	-1.927086000	-1.297373000
6	1.773922000	-2.130815000	-2.525089000
6	-4.953496000	1.641473000	-0.853528000
6	-4.057609000	-0.407037000	-2.042647000
6	-4.056852000	2.458229000	1.238839000
6	-2.120333000	1.317544000	2.437581000
1	4.745278000	-1.606885000	2.010758000
6	4.769290000	-1.787194000	-0.140535000
1	1.034644000	-1.875730000	2.537702000
6	2.309810000	-0.343868000	3.337409000
6	2.701632000	-2.828538000	3.528454000
1	4.487311000	-2.015820000	-2.270231000
1	0.742155000	-1.795241000	-2.304038000
6	1.703921000	-3.620880000	-2.935311000
6	2.297820000	-1.278702000	-3.700611000
1	5.861996000	-1.759921000	-0.208540000
1	1.913372000	-0.384063000	4.367484000
1	1.781562000	0.463121000	2.806008000
1	3.377561000	-0.068939000	3.399253000
1	2.644168000	-3.818276000	3.045805000
1	2.153362000	-2.879071000	4.485391000
1	3.763346000	-2.642272000	3.768088000
1	2.713557000	-4.020589000	-3.139343000
1	1.098750000	-3.744204000	-3.851019000
1	1.249604000	-4.240822000	-2.145135000
1	2.390061000	-0.214330000	-3.425781000
1	1.607341000	-1.353161000	-4.558775000
1	3.287586000	-1.620077000	-4.052376000
1	-5.666924000	1.739715000	-1.679709000

6	-4.975552000	2.575151000	0.188693000
1	-3.312621000	-1.195106000	-1.837757000
6	-5.438343000	-1.081273000	-2.217817000
6	-3.645166000	0.292712000	-3.359055000
1	-4.070216000	3.195509000	2.048121000
1	-1.248851000	0.745534000	2.067054000
6	-2.720094000	0.532421000	3.628420000
6	-1.611688000	2.693124000	2.917746000
1	-5.702287000	3.394512000	0.180664000
1	-5.778391000	-1.576633000	-1.292988000
1	-5.396906000	-1.841114000	-3.017698000
1	-6.212454000	-0.346073000	-2.500401000
1	-4.359090000	1.091960000	-3.626016000
1	-3.622237000	-0.431448000	-4.192501000
1	-2.644425000	0.748332000	-3.274098000
1	-3.629278000	1.032540000	4.007756000
1	-1.994396000	0.472888000	4.458796000
1	-2.991659000	-0.496814000	3.344807000
1	-1.238148000	3.307367000	2.081237000
1	-0.788216000	2.561083000	3.640333000
1	-2.401021000	3.270574000	3.431212000
6	2.043692000	4.447267000	-0.461238000
6	3.847918000	2.876070000	-0.079269000
6	2.972718000	5.419894000	-0.864175000
1	0.973946000	4.688410000	-0.448059000
6	4.779258000	3.845078000	-0.482227000
1	4.191739000	1.881086000	0.228231000
6	4.344629000	5.121563000	-0.876600000
1	2.623991000	6.414102000	-1.164582000
1	5.848182000	3.604769000	-0.483903000
1	5.070985000	5.879725000	-1.188010000
1	-4.531028000	-2.366563000	0.281462000
1	-3.929088000	-2.961927000	1.847234000
1	-4.368771000	-1.237543000	1.637382000
1	0.922229000	-4.238888000	1.839718000
1	-0.105548000	-5.025000000	0.623890000
1	1.456068000	-4.285492000	0.148830000

2j (V)
SCF (BP86/SDD/6-31G**) Energy 333 K = -1712.90650818
Thermal correction to Gibbs Free Energy= 0.682807
Lowest Frequency = 11.6654 cm^{-1}
Second Frequency = 16.6253 cm^{-1}
SCF (B3PW91-D3,C6H6/tzvp) Energy 333 K= -2852.67375952

1	-0.472308000	2.550213000	-1.906980000
6	-0.301085000	2.757596000	-0.833779000
26	-0.261726000	-0.118894000	-0.003259000
6	0.615016000	1.696940000	-0.197926000
1	0.136023000	3.775387000	-0.774710000
1	-1.291098000	2.794346000	-0.347360000
1	0.745028000	1.951620000	0.882646000
6	2.030684000	1.664443000	-0.839537000
7	-2.154513000	-0.655016000	0.280206000
7	0.466375000	-1.943936000	0.287163000
1	2.621920000	0.840332000	-0.398721000
1	1.933580000	1.437956000	-1.918993000
6	2.810011000	2.964713000	-0.683585000
6	-2.565302000	-1.906462000	0.592136000
6	-3.118211000	0.401252000	0.117373000
6	-1.678384000	-2.997776000	0.738178000
6	-4.047840000	-2.176496000	0.788944000
6	-0.272978000	-3.035186000	0.592970000
1	-2.147876000	-3.951043000	0.993261000
6	0.420827000	-4.372690000	0.788908000
6	1.893738000	-2.047169000	0.142892000
6	-3.711433000	0.620901000	-1.161013000
6	-3.409687000	1.265563000	1.213023000
6	-4.572705000	1.722422000	-1.320593000
6	-3.416815000	-0.290132000	-2.355237000
6	-4.282628000	2.349765000	0.998391000
6	-2.807777000	1.047979000	2.603022000
1	-5.027101000	1.907407000	-2.300420000
6	-4.858810000	2.585988000	-0.255566000
1	-2.868230000	-1.170104000	-1.976729000
6	-2.502212000	0.416246000	-3.382242000
6	-4.704791000	-0.801514000	-3.038623000
1	-5.530881000	3.438119000	-0.402011000
1	-4.512588000	3.022254000	1.832712000
1	-2.167779000	0.149408000	2.550207000
6	-3.899209000	0.784154000	3.666005000
6	-1.915032000	2.235578000	3.029245000
1	-2.273117000	-0.254239000	-4.229345000
1	-1.548896000	0.726080000	-2.920901000
1	-2.986147000	1.322624000	-3.787185000
1	-5.375678000	-1.305400000	-2.321953000
1	-4.455176000	-1.522138000	-3.836796000
1	-5.274955000	0.020958000	-3.505058000
1	-4.563776000	1.657962000	3.785424000
1	-3.439939000	0.577708000	4.648650000
1	-4.531105000	-0.079777000	3.398598000
1	-1.107804000	2.413995000	2.299552000
1	-1.453938000	2.040695000	4.013521000
1	-2.500605000	3.168068000	3.113686000
6	2.736639000	-1.782670000	1.262238000
6	2.452524000	-2.327744000	-1.138894000
6	4.130718000	-1.769443000	1.063827000
6	2.170282000	-1.518486000	2.659245000
6	3.852592000	-2.299784000	-1.281003000
6	1.573192000	-2.643398000	-2.351205000
1	4.788911000	-1.560607000	1.914946000
6	4.691140000	-2.016628000	-0.195485000
1	1.073949000	-1.636223000	2.601111000
6	2.697357000	-2.536916000	3.696213000
6	2.459515000	-0.074220000	3.127193000
1	5.777736000	-1.994700000	-0.329325000
1	4.293824000	-2.502913000	-2.263307000
1	0.536450000	-2.758529000	-1.989642000
6	1.978281000	-3.967596000	-3.037167000
6	1.580513000	-1.480817000	-3.369851000
1	2.487238000	-3.576467000	3.392152000
1	2.223224000	-2.367425000	4.678774000
1	3.789173000	-2.445731000	3.833843000
1	3.545821000	0.111392000	3.201410000
1	2.019391000	0.108983000	4.123313000
1	2.038745000	0.665754000	2.425589000
1	2.987279000	-3.905589000	-3.481191000

1 1.273918000 -4.208929000 -3.852260000

1 1.979844000 -4.811811000 -2.326661000

1 1.223837000 -0.543756000 -2.908806000

1 0.927874000 -1.710346000 -4.230594000

1 2.598679000 -1.295761000 -3.755457000

6 3.502983000 3.245961000 0.513381000

6 2.836541000 3.934292000 -1.707534000

6 4.195693000 4.453801000 0.684111000

1 3.503937000 2.499639000 1.317087000

6 3.526733000 5.146461000 -1.542026000

1 2.313681000 3.730243000 -2.649662000

6 4.208785000 5.411429000 -0.344187000

1 4.732679000 4.646380000 1.619723000

1 3.534628000 5.883241000 -2.353046000

1 4.750801000 6.354185000 -0.213881000

1 0.943031000 -4.687602000 -0.131319000

1 1.189702000 -4.311895000 1.577978000

1 -0.301260000 -5.155744000 1.063550000

1 -4.219745000 -3.212341000 1.116844000

1 -4.482890000 -1.492568000 1.537115000

1 -4.609355000 -2.011532000 -0.147274000

***rot*-2j (V)**
SCF (BP86/SDD/6-31G**) Energy 333 K = -1712.90490118
Thermal correction to Gibbs Free Energy= 0.684671
Lowest Frequency = 10.7439 cm^{-1}
Second Frequency = 12.2240 cm^{-1}
SCF (B3PW91-D3,C6H6/tzvp) Energy 333 K= -2852.66919383

1 0.326342000 2.459644000 1.223382000

6 -0.084023000 2.722291000 0.228632000

6 0.298793000 1.629396000 -0.806845000

1 1.403595000 1.649843000 -0.938156000

26 -0.046405000 -0.249390000 -0.134613000

6 -0.372720000 1.874645000 -2.169801000

7 1.258036000 -1.707159000 0.208523000

6 0.915779000 -2.955032000 0.608661000

6 -0.418705000 -3.352265000 0.848471000

6 -1.617749000 -2.608072000 0.743584000

1 -0.544770000 -4.390073000 1.167989000

7 -1.652541000 -1.312071000 0.357740000

6 2.643460000 -1.365667000 0.021725000

6 3.413311000 -0.902499000 1.129847000

6 3.216113000 -1.429097000 -1.282594000

6 4.747252000 -0.512911000 0.906176000

6 2.821800000 -0.791016000 2.536881000

1 5.345762000 -0.150718000 1.749746000

6 5.323607000 -0.578712000 -0.368507000

1 6.364598000 -0.274168000 -0.519824000

6 4.557897000 -1.034707000 -1.447895000

1 5.009100000 -1.085717000 -2.445354000

6 2.422900000 -1.916887000 -2.496727000

1 1.827410000 -1.270064000 2.520889000

6 2.616703000 0.689756000 2.931596000

6 3.676661000 -1.518735000 3.598598000

1 2.152019000 0.769852000 3.930283000

1 1.966950000 1.208860000 2.206361000

1 3.579824000 1.229423000 2.962293000

1 3.840736000 -2.578060000 3.337162000

1 3.178045000 -1.484090000 4.582923000

1 4.668967000 -1.048768000 3.715473000

1 1.386610000 -2.102805000 -2.161540000

6 2.982330000 -3.247952000 -3.050376000

6 2.373041000 -0.848715000 -3.612318000

1 4.021939000 -3.127093000 -3.403050000

1 2.376754000 -3.597823000 -3.904913000

1 2.979852000 -4.043211000 -2.286040000

1 1.973594000 0.107417000 -3.235829000

1 1.732327000 -1.188338000 -4.445048000

1 3.377138000 -0.650148000 -4.026901000

6 -2.898151000 -0.599660000 0.273845000

6 -3.733915000 -0.738147000 -0.873236000

6 -3.241882000 0.309466000 1.322782000

6 -4.909222000 0.035129000 -0.942945000

6 -3.387283000 -1.676979000 -2.032017000

1 -5.557585000 -0.059446000 -1.821356000

6 -5.264073000 0.918902000 0.081846000

1 -6.184126000 1.508318000 0.008140000

6 -4.432650000 1.049820000 1.202267000

1 -4.712892000 1.747081000 1.997803000

6	-2.356015000	0.457791000	2.563984000
1	-2.504942000	-2.269313000	-1.733665000
6	-4.535676000	-2.662448000	-2.349388000
6	-3.001745000	-0.883643000	-3.301941000
1	-4.852405000	-3.228751000	-1.457390000
1	-4.219304000	-3.385731000	-3.121085000
1	-5.424574000	-2.135251000	-2.738196000
1	-3.845025000	-0.262515000	-3.652616000
1	-2.727035000	-1.571567000	-4.121220000
1	-2.148219000	-0.212606000	-3.114033000
1	-1.309953000	0.291877000	2.233768000
6	-2.672319000	-0.624865000	3.623883000
6	-2.422859000	1.855755000	3.211026000
1	-3.723099000	-0.546548000	3.955381000
1	-2.026469000	-0.497743000	4.510704000
1	-2.511118000	-1.641565000	3.233574000
1	-2.251072000	2.659656000	2.476967000
1	-1.654027000	1.941594000	3.997757000
1	-3.398637000	2.037540000	3.695606000
1	-1.185323000	2.744135000	0.338214000
6	0.407635000	4.116965000	-0.139344000
1	-0.081545000	1.117255000	-2.920355000
1	-1.475290000	1.850066000	-2.084506000
1	-0.106404000	2.864523000	-2.595141000
6	1.755006000	4.479613000	0.073439000
6	-0.450499000	5.066405000	-0.731443000
6	2.229841000	5.747674000	-0.292259000
1	2.436809000	3.755687000	0.536435000
6	0.020344000	6.337121000	-1.102075000
1	-1.502750000	4.805728000	-0.897007000
6	1.362985000	6.682598000	-0.883641000
1	3.278270000	6.009712000	-0.111332000
1	-0.665432000	7.059342000	-1.558901000
1	1.731724000	7.673792000	-1.168345000
6	-2.910768000	-3.348010000	1.054134000
1	-2.720366000	-4.198066000	1.727367000
1	-3.661893000	-2.688007000	1.514690000
1	-3.359725000	-3.751629000	0.128859000
6	2.004542000	-3.989692000	0.842645000
1	2.622112000	-3.722896000	1.718417000
1	1.570201000	-4.984730000	1.020100000
1	2.692892000	-4.050852000	-0.016402000

2j (III)
SCF (BP86/SDD/6-31G**) Energy 333 K = -1712.89501732
Thermal correction to Gibbs Free Energy= 0.687650
Lowest Frequency = 12.7302 cm^{-1}
Second Frequency = 18.8138 cm^{-1}
SCF (B3PW91-D3,C6H6/tzvp) Energy 333 K= -2852.64778550

1	-1.061546000	1.521924000	-0.747514000
6	-0.067667000	2.123614000	-0.702059000
26	-0.343902000	-0.077115000	-0.276267000
1	-0.058030000	2.577297000	-1.708357000
6	1.122910000	1.250532000	-0.377036000
1	-0.287387000	2.896831000	0.051995000
7	0.419130000	-1.786259000	-0.011806000
7	-2.200756000	-0.576151000	0.098065000
1	1.696772000	0.982970000	-1.282939000
6	2.069029000	1.691415000	0.742194000
1	1.482238000	1.978175000	1.635291000
1	2.696519000	0.828281000	1.028023000
6	2.968177000	2.853572000	0.337775000
6	-0.302661000	-2.917595000	0.201815000
6	1.859734000	-1.864963000	-0.035536000
6	-2.603774000	-1.847507000	0.331895000
6	-3.193744000	0.470086000	0.068276000
6	-1.705109000	-2.933623000	0.347294000
6	0.405515000	-4.260836000	0.309753000
6	2.588799000	-1.997438000	1.184120000
6	2.541983000	-1.758490000	-1.285251000
6	-4.071799000	-2.142217000	0.590324000
6	-3.814931000	0.821332000	-1.167190000
6	-3.493740000	1.190882000	1.260643000
1	-2.149734000	-3.917924000	0.515050000
6	3.997163000	-1.994090000	1.123579000
6	1.905321000	-2.143191000	2.549831000
6	3.949546000	-1.768969000	-1.285024000
6	1.768579000	-1.663236000	-2.602937000
6	-4.709625000	1.908652000	-1.184988000

6	-3.531712000	0.058018000	-2.464032000
6	-4.395114000	2.270731000	1.185880000
6	-2.854121000	0.832897000	2.604019000
1	4.569783000	-2.085340000	2.053278000
6	4.678739000	-1.880493000	-0.093238000
1	0.830570000	-2.318001000	2.374549000
6	2.018208000	-0.856185000	3.398499000
6	2.456455000	-3.348073000	3.348150000
1	4.485780000	-1.691289000	-2.236333000
1	0.794680000	-1.188295000	-2.364111000
6	1.460470000	-3.064635000	-3.181568000
6	2.465050000	-0.791296000	-3.667508000
1	5.773679000	-1.884284000	-0.115026000
1	1.546010000	-1.004956000	4.385949000
1	1.519823000	-0.007235000	2.905761000
1	3.074405000	-0.580785000	3.567591000
1	2.437943000	-4.280226000	2.758502000
1	1.857544000	-3.507061000	4.261768000
1	3.500208000	-3.181072000	3.667519000
1	2.394959000	-3.612915000	-3.397363000
1	0.891739000	-2.978365000	-4.124539000
1	0.862883000	-3.671080000	-2.482676000
1	2.738741000	0.201958000	-3.272548000
1	1.794052000	-0.643423000	-4.531198000
1	3.385178000	-1.264936000	-4.053753000
1	-5.189341000	2.188978000	-2.129948000
6	-5.000011000	2.635016000	-0.023233000
1	-2.847941000	-0.772695000	-2.217184000
6	-4.817767000	-0.550791000	-3.068851000
6	-2.822624000	0.951178000	-3.508232000
1	-4.626284000	2.836263000	2.095905000
1	-2.270529000	-0.091961000	2.455731000
6	-3.910783000	0.558234000	3.697882000
6	-1.869311000	1.930576000	3.067126000
1	-5.697788000	3.478369000	-0.059526000
1	-5.334867000	-1.210848000	-2.352130000
1	-4.576999000	-1.147215000	-3.966272000
1	-5.532650000	0.234141000	-3.372903000

1	-3.452481000	1.812170000	-3.794073000
1	-2.602445000	0.376941000	-4.425427000
1	-1.869990000	1.348946000	-3.119664000
1	-4.498282000	1.463058000	3.934135000
1	-3.421580000	0.228874000	4.631209000
1	-4.621930000	-0.227608000	3.391121000
1	-1.062818000	2.076035000	2.328436000
1	-1.403674000	1.656562000	4.030203000
1	-2.383520000	2.898684000	3.203270000
6	2.652683000	4.182027000	0.688342000
6	4.124404000	2.623997000	-0.438803000
6	3.461230000	5.252120000	0.271204000
1	1.766979000	4.378486000	1.305094000
6	4.935184000	3.689501000	-0.857229000
1	4.389321000	1.594075000	-0.707726000
6	4.605266000	5.009554000	-0.505271000
1	3.199698000	6.276527000	0.558812000
1	5.831880000	3.489448000	-1.454382000
1	5.239263000	5.841851000	-0.828913000
1	-4.711141000	-1.778707000	-0.231861000
1	-4.237710000	-3.223195000	0.710438000
1	-4.424534000	-1.634849000	1.505368000
1	0.749439000	-4.454527000	1.341076000
1	-0.285929000	-5.072869000	0.035871000
1	1.292158000	-4.315155000	-0.340147000

2j$_E$(III)
SCF (BP86/SDD/6-31G**) Energy 333 K = -1712.89189475
Thermal correction to Gibbs Free Energy= 0.689053
Lowest Frequency = 18.8835 cm^{-1}
Second Frequency = 20.8193 cm^{-1}
SCF (B3PW91-D3,C6H6/tzvp) Energy 333 K= -2852.64658472

1	-1.149825000	1.009691000	-0.750058000
6	-0.636283000	1.417954000	-1.748367000
26	0.053267000	-0.191223000	-0.375424000
6	0.497029000	0.507127000	-2.155134000
1	-1.514709000	1.340333000	-2.416834000
6	-0.301029000	2.872135000	-1.472683000
1	1.469732000	1.025170000	-2.199607000
6	0.250179000	-0.418837000	-3.339533000
7	1.462957000	-1.234690000	0.312467000

7	-1.457501000	-1.183619000	0.402530000
1	1.036330000	-1.185562000	-3.426788000
1	-0.714428000	-0.945758000	-3.245019000
1	0.226891000	0.134903000	-4.301492000
6	1.278437000	-2.423536000	0.941011000
6	2.759535000	-0.591001000	0.353949000
6	0.001166000	-2.946630000	1.234090000
6	2.470951000	-3.262611000	1.376876000
6	-1.268367000	-2.353176000	1.065357000
1	0.001955000	-3.913316000	1.745731000
6	-2.437333000	-3.046921000	1.750007000
6	-2.798114000	-0.651474000	0.337480000
6	3.045449000	0.269548000	1.460784000
6	3.725918000	-0.778465000	-0.677510000
6	4.292203000	0.922193000	1.506882000
6	2.063191000	0.456176000	2.621234000
6	4.956118000	-0.097993000	-0.579033000
6	3.499342000	-1.713089000	-1.866952000
1	4.520943000	1.575296000	2.356186000
6	5.245871000	0.746792000	0.497554000
1	1.084532000	0.060378000	2.293801000
6	1.860381000	1.937323000	3.006526000
6	2.508962000	-0.361563000	3.857048000
1	6.210813000	1.261915000	0.552259000
1	5.706286000	-0.242481000	-1.364450000
1	2.443695000	-2.036527000	-1.834434000
6	4.396073000	-2.971769000	-1.777751000
6	3.752685000	-1.005213000	-3.218121000
1	1.105251000	2.019419000	3.807471000
1	1.511929000	2.538415000	2.150495000
1	2.789106000	2.396374000	3.388783000
1	2.602945000	-1.434311000	3.623278000
1	1.778963000	-0.254321000	4.678698000
1	3.489205000	-0.010624000	4.226656000
1	5.464232000	-2.696101000	-1.834891000
1	4.182521000	-3.659837000	-2.614745000
1	4.246346000	-3.522062000	-0.835788000
1	3.168036000	-0.076679000	-3.313167000

1	3.483867000	-1.670042000	-4.057837000
1	4.818542000	-0.743316000	-3.339747000
6	-3.653971000	-1.011845000	-0.748099000
6	-3.249588000	0.264407000	1.333326000
6	-4.951723000	-0.464900000	-0.793123000
6	-3.210978000	-1.969742000	-1.858154000
6	-4.551235000	0.791150000	1.230781000
6	-2.360098000	0.704944000	2.497418000
1	-5.618758000	-0.745388000	-1.616128000
6	-5.406824000	0.427502000	0.184405000
1	-2.122925000	-2.122301000	-1.741699000
6	-3.897859000	-3.351950000	-1.747910000
6	-3.465005000	-1.380089000	-3.264646000
1	-6.419786000	0.840055000	0.128330000
1	-4.901490000	1.496624000	1.993130000
1	-1.411328000	0.145028000	2.421223000
6	-2.992117000	0.378142000	3.869563000
6	-2.026006000	2.211479000	2.402054000
1	-3.670695000	-3.852210000	-0.793038000
1	-3.561732000	-4.016765000	-2.563308000
1	-4.995550000	-3.253827000	-1.824407000
1	-4.544553000	-1.288766000	-3.478377000
1	-3.033230000	-2.035735000	-4.041136000
1	-3.018994000	-0.377684000	-3.375965000
1	-3.928343000	0.942062000	4.028175000
1	-2.300921000	0.646914000	4.687585000
1	-3.231667000	-0.694531000	3.964277000
1	-1.514066000	2.455266000	1.455622000
1	-1.367782000	2.519040000	3.233397000
1	-2.941571000	2.826960000	2.454030000
6	0.822671000	3.234016000	-0.698180000
6	-1.111620000	3.896378000	-2.000772000
6	1.129821000	4.583561000	-0.468182000
1	1.470265000	2.451844000	-0.282229000
6	-0.805540000	5.247514000	-1.771310000
1	-1.989491000	3.630750000	-2.601499000
6	0.316301000	5.596310000	-1.003618000
1	2.008699000	4.843510000	0.131427000

1	-1.446992000	6.028007000	-2.194491000
1	0.555603000	6.649328000	-0.822516000
1	-3.358900000	-3.015457000	1.149920000
1	-2.190488000	-4.096585000	1.971940000
1	-2.670764000	-2.549061000	2.709165000
1	2.241936000	-3.809866000	2.305269000
1	2.712778000	-4.017110000	0.606195000
1	3.372899000	-2.654164000	1.539056000

2j$_Z$(III)
SCF (BP86/SDD/6-31G**) Energy 333 K = -1712.89031647
Thermal correction to Gibbs Free Energy= 0.688870
Lowest Frequency = 11.5217 cm^{-1}
Second Frequency = 22.5801 cm^{-1}
SCF (B3PW91-D3,C6H6/tzvp) Energy 333 K= -2852.64801108

1	1.191696000	0.893957000	-0.829988000
6	0.726275000	1.976872000	-0.812508000
26	-0.266732000	0.045846000	-0.248377000
6	-0.549421000	2.001948000	0.011842000
1	0.492321000	2.137176000	-1.882220000
6	1.894252000	2.859491000	-0.400034000
1	-1.424435000	2.318664000	-0.578781000
6	-0.528449000	2.647556000	1.387175000
7	-1.955570000	-0.782865000	-0.031092000
7	0.819119000	-1.583943000	-0.078969000
1	-1.493522000	2.484889000	1.891604000
1	0.269332000	2.243899000	2.034097000
1	-0.367452000	3.743831000	1.331505000
6	-2.147865000	-2.128831000	-0.025247000
6	-3.121310000	0.067312000	0.041775000
6	-1.097037000	-3.066629000	-0.014151000
6	-3.561720000	-2.688177000	-0.108815000
6	0.293619000	-2.829535000	-0.006677000
1	-1.400591000	-4.116422000	0.001141000
6	1.201740000	-4.043665000	0.108309000
6	2.254047000	-1.449192000	-0.062648000
6	-3.769979000	0.484626000	-1.154379000
6	-3.596611000	0.491355000	1.321228000
6	-4.899725000	1.320183000	-1.051260000
6	-3.272532000	0.064386000	-2.540164000
6	-4.738884000	1.314235000	1.363308000
6	-2.885384000	0.084136000	2.618953000
1	-5.405423000	1.650315000	-1.965978000
6	-5.391068000	1.727021000	0.193411000
1	-2.454139000	-0.662326000	-2.393231000
6	-2.690908000	1.267982000	-3.317738000
6	-4.375497000	-0.623559000	-3.376531000
1	-6.276159000	2.369211000	0.255009000
1	-5.122452000	1.648029000	2.332312000
1	-1.804304000	0.046454000	2.381777000
6	-3.293957000	-1.323298000	3.120799000
6	-3.087381000	1.092946000	3.770348000
1	-2.310593000	0.948690000	-4.304313000
1	-1.859384000	1.737785000	-2.765677000
1	-3.459871000	2.042458000	-3.487035000
1	-4.817694000	-1.483173000	-2.845368000
1	-3.962531000	-0.988798000	-4.333131000
1	-5.196958000	0.074415000	-3.616514000
1	-4.387174000	-1.385053000	3.268069000
1	-2.812114000	-1.532012000	4.092611000
1	-2.990145000	-2.118398000	2.424445000
1	-2.887048000	2.132712000	3.462937000
1	-2.406980000	0.847856000	4.603623000
1	-4.115620000	1.053110000	4.172762000
6	2.952369000	-1.412047000	1.180676000
6	2.961242000	-1.311027000	-1.295488000
6	4.350546000	-1.236426000	1.161865000
6	2.228432000	-1.522501000	2.525136000
6	4.358852000	-1.146320000	-1.255557000
6	2.241855000	-1.353272000	-2.646335000
1	4.895406000	-1.200528000	2.112126000
6	5.055879000	-1.108810000	-0.041143000
1	1.174112000	-1.771880000	2.316081000
6	2.814094000	-2.636699000	3.421851000
6	2.244165000	-0.170449000	3.274786000
1	6.143349000	-0.979601000	-0.032996000
1	4.911643000	-1.046042000	-2.196209000
1	1.158074000	-1.279665000	-2.439243000
6	2.487818000	-2.692443000	-3.380344000

6	2.630443000	-0.167233000	-3.557322000
1	2.829402000	-3.613741000	2.909669000
1	2.213593000	-2.743599000	4.341989000
1	3.849873000	-2.408726000	3.729868000
1	3.276691000	0.138516000	3.516889000
1	1.682874000	-0.243591000	4.223003000
1	1.789374000	0.629159000	2.666802000
1	3.562657000	-2.834723000	-3.591959000
1	1.948259000	-2.711992000	-4.343782000
1	2.145158000	-3.554161000	-2.784392000
1	2.494445000	0.801805000	-3.048684000
1	2.011019000	-0.167907000	-4.471249000
1	3.685436000	-0.226260000	-3.878186000
6	3.049170000	2.349195000	0.222442000
6	1.808962000	4.248140000	-0.641200000
6	4.094252000	3.208956000	0.600536000
1	3.136632000	1.272940000	0.406924000
6	2.852641000	5.105157000	-0.265034000
1	0.915286000	4.655468000	-1.128575000
6	4.000435000	4.587786000	0.359990000
1	4.986515000	2.792737000	1.080196000
1	2.770611000	6.179631000	-0.461798000
1	4.816399000	5.256595000	0.653431000
1	-3.936669000	-2.622935000	-1.146405000
1	-3.573783000	-3.749002000	0.184697000
1	-4.275139000	-2.137363000	0.521786000
1	1.602792000	-4.140962000	1.133172000
1	0.645706000	-4.965352000	-0.121496000
1	2.071556000	-3.975450000	-0.564225000

TS_E(*rot*-2j-XV_E) (V)
SCF (BP86/SDD/6-31G**) Energy 333 K = -1712.85829112
Thermal correction to Gibbs Free Energy= 0.682999
Lowest Frequency = -859.2023 cm^{-1}
Second Frequency = 16.5231 cm^{-1}
SCF (B3PW91-D3,C6H6/tzvp) Energy 333 K= -2852.62624132

1	0.530441000	1.608260000	0.480012000
6	0.511821000	2.007966000	-1.127330000
6	0.027991000	0.941894000	-1.993053000
1	0.810525000	0.365473000	-2.509290000
26	-0.190325000	0.218570000	-0.039833000
6	-1.230839000	1.177398000	-2.815758000
7	0.844070000	-1.288271000	0.806393000
6	0.359359000	-1.987423000	1.845192000
6	-0.946059000	-1.806735000	2.371649000
6	-2.023879000	-1.035539000	1.883769000
1	-1.176159000	-2.417826000	3.248780000
7	-1.946822000	-0.224634000	0.805269000
6	2.179287000	-1.482559000	0.317882000
6	3.293815000	-0.935980000	1.021955000
6	2.357828000	-2.153154000	-0.931814000
6	4.575628000	-1.092330000	0.459216000
6	3.149603000	-0.175575000	2.343460000
1	5.440505000	-0.675994000	0.987404000
6	4.767398000	-1.755740000	-0.757695000
1	5.774929000	-1.865752000	-1.172973000
6	3.662469000	-2.278358000	-1.444258000
1	3.817148000	-2.795485000	-2.396450000
6	1.164644000	-2.762045000	-1.671164000
1	2.097400000	-0.250438000	2.666656000
6	3.461383000	1.328308000	2.168299000
6	4.038543000	-0.777958000	3.456832000
1	3.345425000	1.856038000	3.131791000
1	2.783844000	1.794107000	1.435282000
1	4.496772000	1.484517000	1.818298000
1	3.864471000	-1.859167000	3.590331000
1	3.838870000	-0.275740000	4.419479000
1	5.111518000	-0.642155000	3.233829000
1	0.284893000	-2.130765000	-1.435463000
6	0.861125000	-4.189025000	-1.155380000
6	1.326077000	-2.773597000	-3.204110000
1	1.721455000	-4.858990000	-1.332646000
1	-0.014025000	-4.613274000	-1.678580000
1	0.643785000	-4.194360000	-0.075159000
1	1.592139000	-1.777521000	-3.596156000
1	0.379973000	-3.084925000	-3.678729000
1	2.102090000	-3.486919000	-3.534760000
6	-3.132384000	0.348268000	0.234142000
6	-3.993774000	-0.456375000	-0.570962000

6	-3.394075000	1.740684000	0.417967000
6	-5.103696000	0.157407000	-1.181930000
6	-3.737092000	-1.947284000	-0.812057000
1	-5.767937000	-0.446368000	-1.810306000
6	-5.373568000	1.519800000	-1.008458000
1	-6.243332000	1.975622000	-1.493364000
6	-4.524159000	2.297493000	-0.211280000
1	-4.741541000	3.361808000	-0.076068000
6	-2.500387000	2.609021000	1.307625000
1	-2.944356000	-2.273891000	-0.118018000
6	-4.989643000	-2.810064000	-0.535463000
6	-3.214644000	-2.203817000	-2.244274000
1	-5.404530000	-2.624193000	0.469930000
1	-4.739799000	-3.882662000	-0.610701000
1	-5.792987000	-2.609710000	-1.265982000
1	-3.941052000	-1.858031000	-3.000987000
1	-3.043694000	-3.282844000	-2.406878000
1	-2.263472000	-1.674876000	-2.417818000
1	-1.488928000	2.155548000	1.305832000
6	-2.991523000	2.609220000	2.774996000
6	-2.358078000	4.059607000	0.801703000
1	-4.019032000	3.009271000	2.844377000
1	-2.336680000	3.240671000	3.401093000
1	-2.990496000	1.595708000	3.206663000
1	-2.083932000	4.098970000	-0.266279000
1	-1.575896000	4.583176000	1.377656000
1	-3.291689000	4.636469000	0.928474000
1	-0.210251000	2.824314000	-0.972104000
6	1.921349000	2.516975000	-1.154089000
1	-1.635626000	0.238750000	-3.228803000
1	-2.035103000	1.637860000	-2.214422000
1	-1.039560000	1.855230000	-3.674563000
6	3.012073000	1.727208000	-1.580561000
6	2.174787000	3.855364000	-0.776969000
6	4.307118000	2.263412000	-1.633377000
1	2.856180000	0.680589000	-1.861387000
6	3.468174000	4.393144000	-0.834265000
1	1.339359000	4.481522000	-0.441251000
6	4.543208000	3.597931000	-1.264707000
1	5.136535000	1.628817000	-1.962918000
1	3.635702000	5.436665000	-0.545838000
1	5.554900000	4.014507000	-1.311863000
6	-3.354469000	-1.212706000	2.601927000
1	-3.188427000	-1.484495000	3.655825000
1	-3.970932000	-0.302558000	2.555128000
1	-3.940994000	-2.025986000	2.138623000
6	1.217017000	-3.039611000	2.532888000
1	1.744653000	-2.601484000	3.398551000
1	0.589258000	-3.861580000	2.910965000
1	1.980809000	-3.449750000	1.855542000

$TS_E(2j_E-XV_E)$ (III)
SCF (BP86/SDD/6-31G**) Energy 333 K = -1712.88422260
Thermal correction to Gibbs Free Energy= 0.686927
Lowest Frequency = -365.1545 cm^{-1}
Second Frequency = 16.7918 cm^{-1}
SCF (B3PW91-D3,C6H6/tzvp) Energy 333 K= -2852.63662882

1	-1.039333000	1.004413000	-0.699980000
6	-0.084348000	1.177697000	-2.039911000
26	-0.079542000	-0.153346000	-0.383082000
6	0.765697000	0.024415000	-2.200572000
1	-1.010276000	1.176844000	-2.634930000
6	0.440464000	2.567207000	-1.822463000
1	1.845512000	0.185000000	-2.070345000
6	0.388170000	-1.084577000	-3.167125000
7	1.252191000	-1.219660000	0.573153000
7	-1.648939000	-0.976394000	0.456259000
1	0.762812000	-2.069241000	-2.839940000
1	-0.705147000	-1.161064000	-3.278917000
1	0.808639000	-0.896145000	-4.175626000
6	0.955486000	-2.255078000	1.389823000
6	2.604001000	-0.705280000	0.540361000
6	-0.367845000	-2.677531000	1.641308000
6	2.045039000	-3.034305000	2.115248000
6	-1.577938000	-2.060173000	1.269950000
1	-0.464004000	-3.548289000	2.295842000
6	-2.844911000	-2.623996000	1.897848000
6	-2.954224000	-0.380328000	0.266946000
6	2.917612000	0.409149000	1.381529000

6	3.597775000	-1.255205000	-0.323815000
6	4.219748000	0.943119000	1.339649000
6	1.891386000	0.992712000	2.357357000
6	4.883047000	-0.675351000	-0.326573000
6	3.344737000	-2.467665000	-1.224646000
1	4.469010000	1.791311000	1.985390000
6	5.200487000	0.412447000	0.492781000
1	0.887938000	0.736378000	1.964581000
6	1.962418000	2.528550000	2.479579000
6	2.024673000	0.340440000	3.754436000
1	6.207263000	0.843230000	0.474593000
1	5.652380000	-1.094999000	-0.984356000
1	2.268760000	-2.709721000	-1.160765000
6	4.150536000	-3.703420000	-0.753457000
6	3.693555000	-2.182382000	-2.704386000
1	1.125370000	2.892929000	3.099458000
1	1.896588000	3.019663000	1.495157000
1	2.893798000	2.862633000	2.970823000
1	1.892188000	-0.752192000	3.711632000
1	1.266702000	0.747988000	4.446414000
1	3.021784000	0.544326000	4.184614000
1	5.235798000	-3.519702000	-0.845661000
1	3.907367000	-4.583337000	-1.374806000
1	3.946998000	-3.959464000	0.297517000
1	3.177662000	-1.289754000	-3.090315000
1	3.410925000	-3.041728000	-3.337373000
1	4.778459000	-2.023395000	-2.834685000
6	-3.772255000	-0.785978000	-0.828076000
6	-3.409127000	0.622356000	1.172435000
6	-5.042457000	-0.193359000	-0.975838000
6	-3.316704000	-1.830881000	-1.849900000
6	-4.680492000	1.191081000	0.969713000
6	-2.543344000	1.125954000	2.328904000
1	-5.682290000	-0.507599000	-1.808351000
6	-5.503175000	0.784796000	-0.087795000
1	-2.251610000	-2.041819000	-1.647118000
6	-4.098482000	-3.159607000	-1.722875000
6	-3.431595000	-1.297876000	-3.296759000
1	-6.494320000	1.231153000	-0.221620000
1	-5.033267000	1.966019000	1.659978000
1	-1.641100000	0.491574000	2.371342000
6	-3.258243000	1.019836000	3.694538000
6	-2.080540000	2.578323000	2.069230000
1	-3.974922000	-3.618737000	-0.728582000
1	-3.747622000	-3.888913000	-2.474505000
1	-5.179122000	-3.000706000	-1.887729000
1	-4.484645000	-1.137636000	-3.588060000
1	-3.001571000	-2.021269000	-4.011976000
1	-2.905799000	-0.336000000	-3.419508000
1	-4.147952000	1.672421000	3.740473000
1	-2.580294000	1.329464000	4.509174000
1	-3.592095000	-0.010799000	3.904025000
1	-1.514084000	2.654000000	1.125698000
1	-1.432743000	2.935208000	2.889554000
1	-2.944141000	3.263617000	2.000687000
6	1.749719000	2.837008000	-1.364328000
6	-0.400649000	3.663504000	-2.117988000
6	2.202087000	4.158027000	-1.225974000
1	2.424067000	2.013206000	-1.108190000
6	0.050414000	4.983902000	-1.977119000
1	-1.420959000	3.471941000	-2.471354000
6	1.357274000	5.238185000	-1.531757000
1	3.223644000	4.341729000	-0.876196000
1	-0.620554000	5.815000000	-2.219668000
1	1.714695000	6.267444000	-1.423463000
1	-3.668725000	-2.710424000	1.173238000
1	-2.650498000	-3.613790000	2.337924000
1	-3.207865000	-1.959605000	2.702434000
1	1.729151000	-3.270049000	3.144569000
1	2.226689000	-3.997920000	1.606587000
1	2.998051000	-2.488102000	2.154904000

TS_Z(*rot*-2j-XV_Z) (V)
SCF (BP86/SDD/6-31G**) Energy 333 K = -1712.85631073
Thermal correction to Gibbs Free Energy= 0.682862
Lowest Frequency = -770.0242 cm^{-1}
Second Frequency = 13.1006 cm^{-1}
SCF (B3PW91-D3,C6H6/tzvp) Energy 333 K= -2852.62192706

1	-0.236730000	1.379181000	0.566422000

6	0.213837000	1.996765000	-0.969509000
6	0.329393000	0.970350000	-1.994016000
1	1.368085000	0.752934000	-2.281877000
26	0.149671000	-0.045281000	-0.159279000
6	-0.685444000	0.787859000	-3.110396000
7	1.842992000	-0.807001000	0.575904000
6	1.863789000	-1.818883000	1.470576000
6	0.714472000	-2.529823000	1.878177000
6	-0.646362000	-2.354083000	1.514410000
7	-1.074422000	-1.408462000	0.662461000
6	3.043500000	-0.130164000	0.174594000
6	3.580342000	0.923980000	0.972994000
6	3.638636000	-0.473780000	-1.079474000
6	4.712497000	1.613331000	0.494876000
6	2.980782000	1.324706000	2.324478000
1	5.133616000	2.426443000	1.097062000
6	5.309843000	1.281490000	-0.726085000
1	6.190789000	1.830166000	-1.075965000
6	4.774034000	0.243362000	-1.500055000
1	5.245220000	-0.011773000	-2.454738000
6	3.081914000	-1.617182000	-1.933108000
1	2.165094000	0.617941000	2.555179000
6	2.359460000	2.739918000	2.283122000
6	4.026628000	1.240356000	3.461524000
1	1.959634000	3.012458000	3.275760000
1	1.528156000	2.791816000	1.561904000
1	3.111390000	3.499223000	2.003512000
1	4.513388000	0.251420000	3.504500000
1	3.548333000	1.430920000	4.438064000
1	4.823696000	1.993797000	3.332793000
1	2.007128000	-1.704009000	-1.685477000
6	3.754204000	-2.965094000	-1.577733000
6	3.193482000	-1.360882000	-3.450570000
1	4.842771000	-2.919573000	-1.760634000
1	3.339708000	-3.779808000	-2.197621000
1	3.598335000	-3.235233000	-0.521298000
1	2.767311000	-0.384732000	-3.736725000
1	2.650636000	-2.145878000	-4.004671000
1	4.241144000	-1.387638000	-3.799663000
6	-2.467061000	-1.241523000	0.357167000
6	-2.959471000	-1.716536000	-0.899140000
6	-3.318857000	-0.535449000	1.258784000
6	-4.309630000	-1.479946000	-1.218401000
6	-2.048682000	-2.486008000	-1.861191000
1	-4.705679000	-1.838200000	-2.173224000
6	-5.158225000	-0.791884000	-0.340076000
1	-6.205169000	-0.618726000	-0.611091000
6	-4.660392000	-0.326658000	0.881817000
1	-5.325215000	0.214657000	1.564415000
6	-2.831229000	0.002826000	2.606884000
1	-1.046877000	-2.011179000	-1.802987000
6	-1.878753000	-3.964223000	-1.434063000
6	-2.510273000	-2.429270000	-3.331063000
1	-1.424130000	-4.056495000	-0.436306000
1	-1.228553000	-4.497921000	-2.149694000
1	-2.856634000	-4.477732000	-1.416514000
1	-3.430585000	-3.018631000	-3.493560000
1	-1.733267000	-2.862893000	-3.983595000
1	-2.700925000	-1.397336000	-3.667319000
1	-1.783879000	-0.317664000	2.739724000
6	-3.662892000	-0.560249000	3.783437000
6	-2.840496000	1.548047000	2.640538000
1	-4.705599000	-0.198000000	3.748412000
1	-3.234157000	-0.234058000	4.747109000
1	-3.697376000	-1.662715000	3.778159000
1	-2.194544000	1.966999000	1.852112000
1	-2.473064000	1.911431000	3.616682000
1	-3.860814000	1.946084000	2.496427000
1	1.174273000	2.410384000	-0.631208000
6	-0.876279000	3.026721000	-0.984443000
1	-0.472456000	-0.134095000	-3.676544000
1	-1.723391000	0.716537000	-2.745347000
1	-0.662150000	1.630816000	-3.831557000
6	-2.248988000	2.705619000	-0.900383000
6	-0.515833000	4.383925000	-1.136222000
6	-3.227411000	3.707024000	-0.985757000

1	-2.552025000	1.665077000	-0.739105000
6	-1.493859000	5.387235000	-1.215330000
1	0.545461000	4.651701000	-1.199540000
6	-2.855319000	5.052249000	-1.145275000
1	-4.285621000	3.433779000	-0.914592000
1	-1.189280000	6.433226000	-1.330344000
1	-3.620314000	5.833537000	-1.205240000
6	-1.639190000	-3.298930000	2.176857000
1	-2.560747000	-3.410771000	1.587182000
1	-1.185506000	-4.290996000	2.329420000
1	-1.925617000	-2.912427000	3.171254000
6	3.180768000	-2.233464000	2.109525000
1	3.392035000	-1.602515000	2.991256000
1	3.137315000	-3.278813000	2.451525000
1	4.028583000	-2.114734000	1.417725000
1	0.899157000	-3.340835000	2.587713000

$TS_Z(2j_Z\text{-}XV_Z)$ (III)
SCF (BP86/SDD/6-31G**) Energy 333 K = -1712.88211936
Thermal correction to Gibbs Free Energy= 0.685673
Lowest Frequency = -448.9871 cm^{-1}
Second Frequency = 10.8896 cm^{-1}
SCF (B3PW91-D3,C6H6/tzvp) Energy 333 K= -2852.63389046

1	0.751574000	0.752695000	-1.059925000
6	0.146488000	2.144659000	-0.465001000
26	-0.176896000	0.108069000	-0.022475000
6	-0.611607000	1.939161000	0.754802000
1	-0.484951000	2.363375000	-1.340313000
6	1.440770000	2.916468000	-0.507789000
1	-1.697567000	2.077046000	0.666286000
6	-0.035015000	2.239535000	2.124823000
7	-1.904587000	-0.808436000	0.076553000
7	0.906966000	-1.497203000	0.154854000
1	-0.483034000	1.601369000	2.905308000
1	1.056546000	2.099748000	2.154832000
1	-0.233516000	3.291850000	2.412983000
6	-2.049862000	-2.153397000	0.167685000
6	-3.098484000	-0.017451000	-0.107728000
6	-0.962360000	-3.041337000	0.294524000
6	-3.425696000	-2.794913000	0.043511000
6	0.416846000	-2.755102000	0.267730000
1	-1.224197000	-4.099662000	0.374324000
6	1.362278000	-3.947427000	0.304576000
6	2.340724000	-1.333090000	0.135041000
6	-3.575479000	0.238150000	-1.426719000
6	-3.775879000	0.523655000	1.027571000
6	-4.735720000	1.022476000	-1.582512000
6	-2.877319000	-0.309928000	-2.674469000
6	-4.937182000	1.290688000	0.811957000
6	-3.272383000	0.285317000	2.454912000
1	-5.110087000	1.223828000	-2.592777000
6	-5.421058000	1.540661000	-0.478579000
1	-2.026615000	-0.927193000	-2.337448000
6	-2.304364000	0.828102000	-3.549563000
6	-3.815659000	-1.203501000	-3.518651000
1	-6.325743000	2.141174000	-0.621369000
1	-5.471038000	1.704902000	1.673675000
1	-2.202091000	0.021561000	2.373820000
6	-3.993614000	-0.900004000	3.140961000
6	-3.390191000	1.541713000	3.345887000
1	-1.787658000	0.414922000	-4.433615000
1	-1.578021000	1.437654000	-2.987325000
1	-3.103443000	1.499795000	-3.910201000
1	-4.246691000	-2.026252000	-2.924033000
1	-3.265121000	-1.647331000	-4.366523000
1	-4.656945000	-0.623054000	-3.937231000
1	-5.083100000	-0.722431000	3.186210000
1	-3.628539000	-1.024365000	4.175925000
1	-3.825105000	-1.850327000	2.611517000
1	-2.946865000	2.430332000	2.867571000
1	-2.872084000	1.374519000	4.306226000
1	-4.442112000	1.777404000	3.586233000
6	3.022432000	-1.058715000	1.360177000
6	3.058425000	-1.421570000	-1.093111000
6	4.425196000	-0.924133000	1.328785000
6	2.266931000	-0.898640000	2.683879000
6	4.456991000	-1.263670000	-1.066229000
6	2.357901000	-1.649281000	-2.434336000
1	4.963358000	-0.723303000	2.260885000

6	5.143568000	-1.030745000	0.131257000
1	1.235166000	-0.595167000	2.423234000
6	2.173043000	-2.225296000	3.474660000
6	2.869580000	0.201149000	3.584825000
1	6.233539000	-0.923138000	0.131072000
1	5.018106000	-1.328353000	-2.005449000
1	1.285569000	-1.807788000	-2.227213000
6	2.893261000	-2.895660000	-3.175457000
6	2.478864000	-0.399873000	-3.337744000
1	1.619632000	-2.998096000	2.919020000
1	1.650289000	-2.064396000	4.434271000
1	3.180219000	-2.619331000	3.700699000
1	3.840025000	-0.104434000	4.015173000
1	2.193563000	0.407982000	4.432178000
1	3.028549000	1.143289000	3.033997000
1	3.952679000	-2.771879000	-3.461847000
1	2.318750000	-3.067736000	-4.102458000
1	2.822960000	-3.807142000	-2.558003000
1	2.074647000	0.495503000	-2.836789000
1	1.925021000	-0.548208000	-4.281642000
1	3.533302000	-0.194485000	-3.594233000
6	2.675149000	2.401525000	-0.061611000
6	1.402889000	4.242689000	-0.994129000
6	3.833623000	3.195323000	-0.083390000
1	2.733628000	1.364508000	0.285285000
6	2.560099000	5.034348000	-1.019834000
1	0.452005000	4.655964000	-1.351112000
6	3.781536000	4.514104000	-0.560005000
1	4.781431000	2.770082000	0.263129000
1	2.507626000	6.059180000	-1.403230000
1	4.686659000	5.130182000	-0.582173000
1	-3.676947000	-2.951859000	-1.021323000
1	-3.434550000	-3.782028000	0.531406000
1	-4.226655000	-2.176028000	0.472017000
1	2.255916000	-3.757454000	0.917913000
1	0.844979000	-4.837672000	0.693916000
1	1.721259000	-4.186028000	-0.712584000

XV_E (V)
SCF (BP86/SDD/6-31G**) Energy 333 K = -1712.87572264
Thermal correction to Gibbs Free Energy= 0.682683
Lowest Frequency = 9.7556 cm^{-1}
Second Frequency = 13.7455 cm^{-1}
SCF (B3PW91-D3,C6H6/tzvp) Energy 333 K= -2852.64132050

1	0.560080000	1.480713000	1.206604000
6	0.277316000	1.706614000	-1.546492000
6	0.282430000	0.368111000	-1.985463000
1	1.252227000	-0.120847000	-2.145825000
26	-0.052392000	0.407382000	0.149892000
6	-0.890756000	-0.242558000	-2.719199000
7	0.995870000	-1.223368000	0.747379000
6	0.522376000	-1.904743000	1.807479000
6	-0.786564000	-1.747411000	2.326744000
6	-1.905695000	-1.023719000	1.847604000
1	-0.983873000	-2.327668000	3.233700000
7	-1.893237000	-0.241606000	0.748289000
6	2.346195000	-1.403812000	0.287948000
6	3.426163000	-0.724537000	0.928112000
6	2.576668000	-2.212840000	-0.867117000
6	4.715011000	-0.852049000	0.371764000
6	3.252754000	0.109965000	2.200636000
1	5.550915000	-0.330520000	0.851475000
6	4.951572000	-1.626624000	-0.768834000
1	5.963094000	-1.708969000	-1.180865000
6	3.886493000	-2.307131000	-1.373773000
1	4.076405000	-2.927995000	-2.255546000
6	1.443986000	-3.019975000	-1.505785000
1	2.188277000	0.075004000	2.488448000
6	3.603723000	1.596471000	1.971916000
6	4.095148000	-0.462834000	3.365934000
1	3.469463000	2.164978000	2.909264000
1	2.952340000	2.042997000	1.205143000
1	4.653860000	1.719400000	1.651815000
1	3.882982000	-1.529605000	3.548999000
1	3.888239000	0.093484000	4.296966000
1	5.176901000	-0.372634000	3.161670000
1	0.498772000	-2.518775000	-1.229492000
6	1.393975000	-4.454631000	-0.925692000

6	1.523306000	-3.082148000	-3.045605000
1	2.332491000	-4.995423000	-1.142277000
1	0.561209000	-5.026770000	-1.372015000
1	1.253478000	-4.449334000	0.167074000
1	1.609364000	-2.079469000	-3.496719000
1	0.617098000	-3.562963000	-3.452903000
1	2.385662000	-3.680080000	-3.389765000
6	-3.110890000	0.274228000	0.187180000
6	-4.000246000	-0.583965000	-0.534606000
6	-3.390347000	1.673283000	0.297605000
6	-5.151108000	-0.018468000	-1.119338000
6	-3.772855000	-2.092085000	-0.692267000
1	-5.839807000	-0.669358000	-1.669633000
6	-5.431522000	1.347597000	-1.018742000
1	-6.331034000	1.763289000	-1.485046000
6	-4.552549000	2.179402000	-0.314266000
1	-4.777215000	3.247244000	-0.230116000
6	-2.496870000	2.602320000	1.121713000
1	-2.783885000	-2.333479000	-0.265470000
6	-4.843599000	-2.910563000	0.070567000
6	-3.769502000	-2.535212000	-2.174352000
1	-4.895524000	-2.642050000	1.136786000
1	-4.627529000	-3.991099000	-0.001239000
1	-5.845501000	-2.741741000	-0.362661000
1	-4.761955000	-2.395890000	-2.637968000
1	-3.521014000	-3.608392000	-2.250169000
1	-3.039415000	-1.970067000	-2.773069000
1	-1.461575000	2.200244000	1.094440000
6	-2.925903000	2.609218000	2.608140000
6	-2.438580000	4.045328000	0.582459000
1	-3.958467000	2.987656000	2.715927000
1	-2.257587000	3.259565000	3.199097000
1	-2.886917000	1.599559000	3.046276000
1	-2.212889000	4.072817000	-0.497623000
1	-1.652623000	4.609607000	1.112600000
1	-3.387706000	4.588891000	0.739325000
1	-0.680825000	2.247649000	-1.604975000
6	1.465078000	2.582466000	-1.411947000
1	-0.965649000	-1.327939000	-2.543237000
1	-1.847476000	0.219991000	-2.422791000
1	-0.778037000	-0.098529000	-3.812712000
6	2.780519000	2.141469000	-1.694602000
6	1.283276000	3.941256000	-1.057128000
6	3.865390000	3.025602000	-1.619238000
1	2.961668000	1.099241000	-1.975103000
6	2.367526000	4.823589000	-0.983121000
1	0.271844000	4.300075000	-0.836533000
6	3.667930000	4.370315000	-1.264289000
1	4.873006000	2.659549000	-1.843362000
1	2.197453000	5.870316000	-0.708533000
1	4.517552000	5.058917000	-1.210536000
6	-3.166382000	-1.165886000	2.693895000
1	-2.963578000	-0.786471000	3.710848000
1	-4.017585000	-0.609967000	2.278119000
1	-3.450398000	-2.225953000	2.800677000
6	1.405242000	-2.897698000	2.551729000
1	1.783574000	-2.439663000	3.482929000
1	0.826448000	-3.790318000	2.838762000
1	2.275164000	-3.206379000	1.954937000

XV$_Z$ (V)
SCF (BP86/SDD/6-31G**) Energy 333 K = -1712.87227719
Thermal correction to Gibbs Free Energy= 0.684554
Lowest Frequency = 15.3441 cm^{-1}
Second Frequency = 20.1930 cm^{-1}
SCF (B3PW91-D3,C6H6/tzvp) Energy 333 K= -2852.63993892

1	0.130706000	1.691346000	1.221699000
6	-0.012532000	1.857310000	-1.438263000
6	0.377885000	0.610874000	-1.976331000
1	1.460869000	0.483319000	-2.101461000
26	0.149120000	0.428235000	0.191517000
6	-0.476156000	-0.330846000	-2.800438000
7	1.941454000	-0.412730000	0.676704000
6	1.979365000	-1.184397000	1.778957000
6	0.826530000	-1.719471000	2.405387000
6	-0.536497000	-1.702002000	2.017498000
7	-1.009505000	-1.059140000	0.929996000
6	3.151928000	0.039418000	0.049866000
6	3.806908000	1.225033000	0.497250000

6	3.646171000	-0.683012000	-1.080515000
6	4.952674000	1.658366000	-0.200168000
6	3.326734000	2.033542000	1.705619000
1	5.464117000	2.567853000	0.134990000
6	5.452870000	0.954849000	-1.300807000
1	6.347373000	1.310148000	-1.823375000
6	4.800589000	-0.207923000	-1.730627000
1	5.194301000	-0.757177000	-2.592117000
6	2.966328000	-1.968938000	-1.558614000
1	2.449499000	1.518526000	2.133506000
6	2.856426000	3.446705000	1.294449000
6	4.419198000	2.133974000	2.796439000
1	2.524684000	4.013633000	2.181912000
1	2.003500000	3.388134000	0.599308000
1	3.667113000	4.018815000	0.808937000
1	4.793009000	1.141792000	3.100727000
1	4.020581000	2.640045000	3.692967000
1	5.288034000	2.718802000	2.445622000
1	1.917881000	-1.924000000	-1.210457000
6	3.614571000	-3.220699000	-0.919428000
6	2.952939000	-2.114319000	-3.095254000
1	4.681536000	-3.291967000	-1.196561000
1	3.111302000	-4.140101000	-1.267675000
1	3.551732000	-3.198949000	0.180153000
1	2.547949000	-1.216026000	-3.590924000
1	2.331916000	-2.978571000	-3.388463000
1	3.963761000	-2.294477000	-3.502169000
6	-2.389332000	-1.190779000	0.541835000
6	-2.738083000	-2.173278000	-0.437919000
6	-3.377888000	-0.318302000	1.085596000
6	-4.078261000	-2.245404000	-0.863681000
6	-1.701302000	-3.162324000	-0.982913000
1	-4.362939000	-2.992225000	-1.611546000
6	-5.055179000	-1.381827000	-0.350706000
1	-6.090703000	-1.453532000	-0.700365000
6	-4.701163000	-0.434454000	0.615227000
1	-5.468414000	0.232947000	1.023409000
6	-3.066917000	0.706914000	2.179485000

1	-0.720719000	-2.652346000	-0.948290000
6	-1.594998000	-4.425004000	-0.092573000
6	-1.968407000	-3.594524000	-2.440479000
1	-1.275164000	-4.183697000	0.932285000
1	-0.860054000	-5.132845000	-0.515268000
1	-2.569159000	-4.942363000	-0.033654000
1	-2.850806000	-4.254498000	-2.516534000
1	-1.107671000	-4.167739000	-2.825882000
1	-2.132980000	-2.733667000	-3.108764000
1	-1.992705000	0.630528000	2.422594000
6	-3.878942000	0.419556000	3.465402000
6	-3.320148000	2.154880000	1.706551000
1	-4.960989000	0.563813000	3.296536000
1	-3.575431000	1.110049000	4.271619000
1	-3.737153000	-0.612989000	3.826196000
1	-2.699115000	2.407770000	0.833932000
1	-3.074913000	2.865261000	2.515497000
1	-4.378722000	2.309808000	1.431925000
1	0.824556000	2.496313000	-1.125503000
6	-1.276589000	2.618910000	-1.552307000
1	0.066799000	-1.268811000	-2.996656000
1	-1.427230000	-0.600777000	-2.311561000
1	-0.729180000	0.115814000	-3.783152000
6	-2.477610000	2.134335000	-2.129700000
6	-1.268445000	3.961392000	-1.090666000
6	-3.607546000	2.958026000	-2.239370000
1	-2.538866000	1.108770000	-2.495817000
6	-2.395247000	4.782469000	-1.203459000
1	-0.353400000	4.353673000	-0.632834000
6	-3.575953000	4.284398000	-1.781448000
1	-4.521005000	2.555343000	-2.690028000
1	-2.352881000	5.814656000	-0.839461000
1	-4.460451000	4.923292000	-1.872820000
6	-1.489926000	-2.442541000	2.945690000
1	-2.401224000	-2.775701000	2.429053000
1	-0.995702000	-3.311519000	3.407343000
1	-1.801526000	-1.766938000	3.762682000
6	3.309209000	-1.505737000	2.446017000

1	3.554104000	-0.722775000	3.186210000
1	3.259123000	-2.465957000	2.982018000
1	4.136186000	-1.537171000	1.721373000
1	1.028504000	-2.285676000	3.318852000

XV$_{E}$ (III)
SCF (BP86/SDD/6-31G**) Energy 333 K = -1712.87927774
Thermal correction to Gibbs Free Energy= 0.688418
Lowest Frequency = 17.1307 cm^{-1}
Second Frequency = 21.8318 cm^{-1}
SCF (B3PW91-D3,C6H6/tzvp) Energy 333 K= -2852.63506640

1	-1.027447000	1.157248000	-0.570264000
6	0.357534000	0.960151000	-2.205776000
26	-0.198952000	-0.097458000	-0.448474000
6	0.924167000	-0.336431000	-2.175535000
1	-0.570512000	1.085824000	-2.781304000
6	1.085815000	2.236122000	-1.970393000
1	1.971432000	-0.430481000	-1.863366000
6	0.397200000	-1.469517000	-3.032923000
7	1.060310000	-1.185359000	0.715157000
7	-1.794592000	-0.819416000	0.422401000
1	0.489193000	-2.448081000	-2.530562000
1	-0.661458000	-1.318356000	-3.298422000
1	0.962655000	-1.537070000	-3.984503000
6	0.674464000	-2.113593000	1.610454000
6	2.442383000	-0.760747000	0.699838000
6	-0.675786000	-2.488960000	1.794575000
6	1.671004000	-2.826598000	2.515932000
6	-1.827161000	-1.862142000	1.288973000
1	-0.847925000	-3.301301000	2.505698000
6	-3.160029000	-2.361406000	1.826438000
6	-3.060330000	-0.164167000	0.150541000
6	2.762138000	0.481243000	1.332317000
6	3.458339000	-1.515829000	0.038434000
6	4.095733000	0.928997000	1.307455000
6	1.692857000	1.298894000	2.063599000
6	4.775354000	-1.010450000	0.036338000
6	3.199440000	-2.862384000	-0.648155000
1	4.349522000	1.875448000	1.794192000
6	5.100714000	0.195447000	0.664518000
1	0.728190000	1.119860000	1.542435000
6	1.945724000	2.818711000	2.037099000
6	1.521681000	0.810949000	3.521859000
1	6.132101000	0.563794000	0.653025000
1	5.562184000	-1.584562000	-0.465874000
1	2.115518000	-3.067305000	-0.589372000
6	3.956583000	-4.016692000	0.054969000
6	3.605936000	-2.849978000	-2.141341000
1	1.067838000	3.346793000	2.446291000
1	2.120254000	3.185927000	1.013144000
1	2.811880000	3.104156000	2.661275000
1	1.229480000	-0.250125000	3.569838000
1	0.743251000	1.400297000	4.037745000
1	2.465916000	0.930259000	4.083100000
1	5.048403000	-3.889755000	-0.052495000
1	3.689607000	-4.986814000	-0.399914000
1	3.736657000	-4.070449000	1.132097000
1	3.107264000	-2.047376000	-2.705509000
1	3.346316000	-3.812521000	-2.615906000
1	4.695420000	-2.710840000	-2.254983000
6	-3.838426000	-0.547113000	-0.978759000
6	-3.517051000	0.864642000	1.025625000
6	-5.075658000	0.093292000	-1.194009000
6	-3.376904000	-1.614999000	-1.973104000
6	-4.754511000	1.478546000	0.757638000
6	-2.688198000	1.351331000	2.216407000
1	-5.685110000	-0.202838000	-2.055441000
6	-5.540260000	1.095505000	-0.336249000
1	-2.345866000	-1.899910000	-1.696159000
6	-4.250185000	-2.890237000	-1.915269000
6	-3.350189000	-1.063566000	-3.417372000
1	-6.505093000	1.579440000	-0.521667000
1	-5.109149000	2.273556000	1.423606000
1	-1.808620000	0.691089000	2.305195000
6	-3.467264000	1.286112000	3.549282000
6	-2.165936000	2.784433000	1.962665000
1	-4.234829000	-3.356413000	-0.916262000
1	-3.889675000	-3.640727000	-2.640900000
1	-5.302275000	-2.662360000	-2.162541000

1	-4.365515000	-0.816550000	-3.774106000
1	-2.932446000	-1.812892000	-4.113009000
1	-2.743135000	-0.145408000	-3.489474000
1	-4.337909000	1.965478000	3.546600000
1	-2.817169000	1.587586000	4.389299000
1	-3.841362000	0.269423000	3.759711000
1	-1.557173000	2.823349000	1.044026000
1	-1.542590000	3.126714000	2.808185000
1	-3.001408000	3.498471000	1.851008000
6	2.488982000	2.301411000	-1.808703000
6	0.360609000	3.452539000	-1.971186000
6	3.138305000	3.536101000	-1.656046000
1	3.083105000	1.382336000	-1.812334000
6	1.008717000	4.684622000	-1.821557000
1	-0.728482000	3.417654000	-2.091678000
6	2.404948000	4.733922000	-1.663181000
1	4.226949000	3.559271000	-1.537701000
1	0.423422000	5.610564000	-1.830498000
1	2.915310000	5.696140000	-1.550297000
1	-3.899671000	-2.502746000	1.023453000
1	-3.028368000	-3.311986000	2.364641000
1	-3.598973000	-1.627342000	2.524458000
1	1.255228000	-2.927000000	3.531505000
1	1.858635000	-3.848620000	2.142772000
1	2.634725000	-2.302468000	2.576280000

XV$_z$ (III)
SCF (BP86/SDD/6-31G**) Energy 333 K = -1712.88441007
Thermal correction to Gibbs Free Energy= 0.687337
Lowest Frequency = 12.7301 cm^{-1}
Second Frequency = 24.5864 cm^{-1}
SCF (B3PW91-D3,C6H6/tzvp) Energy 333 K= -2852.63452263

1	0.571993000	1.013668000	-0.987483000
6	-0.009993000	2.127327000	0.711128000
26	-0.149082000	0.137975000	-0.006027000
6	-0.636370000	1.279613000	1.669077000
1	-0.708865000	2.679402000	0.065673000
6	1.303356000	2.822153000	0.855127000
1	-1.733933000	1.303353000	1.667557000
6	-0.014115000	0.849558000	2.980455000
7	-1.896320000	-0.740129000	-0.348821000
7	0.938381000	-1.371972000	-0.503278000
1	-0.299747000	-0.180261000	3.257230000
1	1.083809000	0.908938000	2.964203000
1	-0.366386000	1.508568000	3.799270000
6	-2.015882000	-1.959064000	-0.926373000
6	-3.097375000	0.029197000	-0.137914000
6	-0.908151000	-2.782474000	-1.221475000
6	-3.373805000	-2.476209000	-1.382313000
6	0.464904000	-2.516594000	-1.056537000
1	-1.147535000	-3.743099000	-1.685407000
6	1.431129000	-3.564929000	-1.588811000
6	2.368308000	-1.213621000	-0.369491000
6	-3.563591000	0.900906000	-1.165633000
6	-3.787142000	-0.059008000	1.110797000
6	-4.719653000	1.668587000	-0.921177000
6	-2.867611000	1.017577000	-2.524799000
6	-4.943746000	0.723347000	1.293390000
6	-3.307384000	-0.984811000	2.234355000
1	-5.085898000	2.342089000	-1.704422000
6	-5.411968000	1.582982000	0.291494000
1	-2.026820000	0.302808000	-2.532900000
6	-2.272577000	2.426831000	-2.744216000
6	-3.819418000	0.656451000	-3.689353000
1	-6.312061000	2.184314000	0.457724000
1	-5.486188000	0.660707000	2.242625000
1	-2.241523000	-1.201040000	2.037726000
6	-4.064224000	-2.335089000	2.237985000
6	-3.412953000	-0.334965000	3.631894000
1	-1.781986000	2.489011000	-3.731561000
1	-1.517155000	2.663096000	-1.977470000
1	-3.055342000	3.205265000	-2.707771000
1	-4.271475000	-0.341109000	-3.558479000
1	-3.272650000	0.660998000	-4.648471000
1	-4.645806000	1.384045000	-3.776066000
1	-5.149218000	-2.174750000	2.370147000
1	-3.712855000	-2.970707000	3.070073000
1	-3.915341000	-2.896498000	1.302741000
1	-2.939747000	0.660089000	3.662388000

1	-2.917726000	-0.973175000	4.384097000
1	-4.463567000	-0.217873000	3.951500000
6	2.981664000	-1.584149000	0.867622000
6	3.147423000	-0.686714000	-1.440392000
6	4.378102000	-1.441851000	0.990067000
6	2.164521000	-2.131054000	2.043177000
6	4.536632000	-0.552415000	-1.253453000
6	2.535477000	-0.248890000	-2.772868000
1	4.864116000	-1.728943000	1.928018000
6	5.155557000	-0.932898000	-0.057742000
1	1.137515000	-1.738735000	1.924491000
6	2.074777000	-3.676144000	2.026470000
6	2.696270000	-1.662000000	3.414719000
1	6.239524000	-0.829466000	0.060463000
1	5.144762000	-0.144178000	-2.068479000
1	1.456149000	-0.474890000	-2.736507000
6	3.147361000	-1.007794000	-3.973308000
6	2.685472000	1.276635000	-2.977311000
1	1.569236000	-4.050937000	1.123282000
1	1.506458000	-4.037143000	2.902066000
1	3.082422000	-4.127257000	2.068396000
1	3.653039000	-2.149388000	3.673946000
1	1.976756000	-1.926208000	4.208650000
1	2.855338000	-0.571455000	3.446141000
1	4.218283000	-0.768816000	-4.098626000
1	2.634017000	-0.723168000	-4.908406000
1	3.065779000	-2.102077000	-3.859744000
1	2.229913000	1.836795000	-2.144400000
1	2.195279000	1.590004000	-3.916022000
1	3.749121000	1.567523000	-3.039747000
6	2.521518000	2.168485000	1.150046000
6	1.328136000	4.228790000	0.702099000
6	3.709330000	2.896741000	1.314655000
1	2.549253000	1.075850000	1.210161000
6	2.515422000	4.957200000	0.863396000
1	0.396361000	4.754959000	0.462769000
6	3.712910000	4.294122000	1.177344000
1	4.639371000	2.361431000	1.532991000
1	2.504146000	6.045877000	0.741537000
1	4.642387000	4.859944000	1.300659000
1	-3.591353000	-2.115830000	-2.404405000
1	-3.377482000	-3.576943000	-1.414949000
1	-4.197506000	-2.133308000	-0.740337000
1	2.326911000	-3.671047000	-0.959167000
1	0.932498000	-4.543148000	-1.668771000
1	1.781566000	-3.284891000	-2.598323000
